# Genetic alterations of histone lysine methyltransferases and their significance in breast cancer

**DOI:** 10.18632/oncotarget.2967

**Published:** 2014-12-11

**Authors:** Lanxin Liu, Sarah Kimball, Hui Liu, Andreana Holowatyj, Zeng-Quan Yang

**Affiliations:** ^1^ Department of Oncology, Karmanos Cancer Institute, Wayne State University, Detroit, MI, USA

**Keywords:** breast cancer, histone lysine methyltransferase, gene amplification, deletion, mutation

## Abstract

Histone lysine methyltransferases (HMTs), a large class of enzymes that catalyze site-specific methylation of lysine residues on histones and other proteins, play critical roles in controlling transcription, chromatin architecture, and cellular differentiation. However, the genomic landscape and clinical significance of HMTs in breast cancer remain poorly characterized. Here, we conducted a meta-analysis of approximately 50 HMTs in breast cancer and identified associations among recurrent copy number alterations, mutations, gene expression, and clinical outcome. We identified 12 HMTs with the highest frequency of genetic alterations, including 8 with high-level amplification, 2 with putative homozygous deletion, and 2 with somatic mutation. Different subtypes of breast cancer have different patterns of copy number and expression for each HMT gene. In addition, chromosome 1q contains four HMTs that are concurrently or independently amplified or overexpressed in breast cancer. Copy number or mRNA expression of several HMTs was significantly associated with basal-like breast cancer and shorter patient survival. Integrative analysis identified 8 HMTs (SETDB1, SMYD3, ASH1L, SMYD2, WHSC1L1, SUV420H1, SETDB2, and KMT2C) that are dysregulated by genetic alterations, classifying them as candidate therapeutic targets. Together, our findings provide a strong foundation for further mechanistic research and therapeutic options using HMTs to treat breast cancer.

## INTRODUCTION

Breast cancer is the most common cancer among women worldwide, with 1.3 million women diagnosed each year and about 500,000 deaths per year from the disease. Distinct subtypes of breast carcinomas that are associated with different clinical outcomes have been identified by expression analysis using microarray-based technology [1, 2]. Five intrinsic molecular subtypes of human breast cancer include Luminal A, Luminal B, human epidermal growth factor receptor 2 (HER2/ERBB2)-positive, basal-like, and normal-like breast cancer [2, 3]. Both Luminal A and Luminal B breast cancers are estrogen receptor (ER) positive, but Luminal B cancers have poorer outcomes [4]. Basal-like breast cancer is especially aggressive as it includes tumors that lack ER, progesterone receptor (PR), and HER2 expression (hence the name “triple-negative”) [5, 6]. These characteristics render conventional therapies ineffective and lead to poor prognosis. By understanding the genetic and epigenetic abnormalities that are associated with the different types of breast cancer, we can identify new subtype-specific targets for therapy.

Histone lysine methylation, which is controlled by histone lysine methyltransferases (HMTs) and demethylases, is an important player in epigenetic regulation [7, 8]. More than 50 human HMTs have been identified [9]. Structurally, the HMTs are a diverse group of proteins that can be broadly categorized into two functional enzymatic families, the SET (Suppressor of variegation, Enhancer of zeste, Trithorax)-domain-containing methyltransferases and the DOT1-like (DOT1L) lysine methyltransferases [8, 9]. HMTs catalyze the transfer of one to three methyl groups from S-adenosylmethionine to specific lysine residues on histones [7]. Depending on the site and degree of methylation (mono-, di-, or trimethylated), lysine methylation can lead to various biological outcomes, including the regulation of chromatin organization and gene transcription.

Recent studies indicated that dysregulation of HMTs can lead to imbalances in histone methylation pathways and contribute to the pathogenesis of a wide array of human cancers, including breast cancer [10-12]. For example, we demonstrated that the methyltransferase gene *WHSC1L1* (*Wolf-Hirschhorn syndrome candidate 1-like 1*) is significantly amplified and overexpressed in breast cancer. We also demonstrated that *WHSC1L1* acts as a transforming gene: stable WHSC1L1 overexpression in nontumorigenic mammary epithelial MCF10A cells induced transformed phenotypes, whereas WHSC1L1 knockdown inhibited proliferation of *WHSC1L1*-amplified breast cancer cells *in vitro*. EZH2, a histone 3 lysine 27 (H3K27) methyltransferase, is also significantly overexpressed in breast cancers, and elevated expression of EZH2 protein has been associated with poor prognoses for inflammatory basal-like breast cancers [13, 14].

Emerging evidence indicates that genetic alterations of several HMTs that have oncogenic or tumor-suppressor functions play important roles in cancer initiation and progression [10, 11, 15]. Despite the extensive DNA and RNA sequencing data, such as The Cancer Genome Atlas (TCGA), in human cancer, there has been no systematic analysis of genomic anomalies and expression of HMTs in different subtypes of breast cancer. In addition, the clinical relevance of genetic alterations for each HMT in breast cancer has yet to be fully explored. Thus, our goals were to determine the genomic landscape and significance of HMTs in different types of breast cancers and to evaluate their diagnostic and prognostic potentials.

## RESULTS

### Genetic alterations of HMTs in breast cancer

Copy number alteration (CNA) and somatic mutation are important mechanisms that activate oncogenes or inactivate tumor suppressors in human cancers [16, 17]. We hypothesized that HMTs with recurrent CNA or mutation would be more likely to play critical roles in breast cancer. Thus, to systematically investigate genetic alterations of HMTs in breast cancer, we first analyzed the genome sequencing data of 958 breast cancer samples from the TCGA database via cBioPortal [18, 19]. In cBioPortal, copy numbers were computed using a GISTIC (Genomic Identification of Significant Targets in Cancer) algorithm, which identified the putative copy number as high-level amplification (+2), low-level gain (+1), diploid (0), heterozygous deletion (−1), or homozygous deletion (−2) [18, 19]. In 958 breast cancer samples, the average CNA rate was 0.15 (range 5.23*10^−6^ to 0.588), based on the segmented copy number scores of the tumor samples and the paired-normal control.

The human genome encodes 51 proteins with demonstrated or predicted ability to methylate histone lysine residues [9, 20]. Except for DOT1L, the rest of the HMTs contain a characteristic SET enzymatic domain and can be divided into four subgroups according to phylogenetic analysis (Table 1 and Supplementary Figure S1) [7, 9]. We analyzed copy numbers (excluding that of KMT2B, for which data were not available in the cBioPortal database) and mutations of these 51 HMTs compiled from 958 TCGA breast cancer specimens. As shown in Table 2, we discovered distinct patterns of altered copy numbers and mutations of HMTs in breast cancer. Notably, we found that eight HMTs exhibited high-level amplification in more than 5% of breast cancers, and five of these eight HMTs (*SMYD3*, *SETDB1*, *ASH1L*, *WHSC1L1*, and *SMYD2*) had high-level amplification in more than 10% of samples. Two HMT genes, *PRDM7* and *SETDB2*, showed homozygous deletion in more than 2% of breast cancers. In addition, two other HMT genes, *KMT2C* and *KMT2D*, exhibited somatic mutations in more than 2% of the 958 breast cancer samples.

**Table 1:** Summary of identified human HMTs and their substrates

Official Symbol	Other Aliases	Gene ID	Gene Location	Histone Substrates
**EHMT1**	GLP; GLP1; KMT1D; FP13812; EUHMTASE1; Eu-HMTase1; bA188C12.1	79813	9q34.3	H3K9; H1.2K187
**EHMT2**	G9A; BAT8; GAT8; NG36; KMT1C; C6orf30	10919	6p21.31	H3K9; H3K27; H3K56; H1.2K187; H1.4K26;
**SUV39H1**	MG44; KMT1A; SUV39H; H3-K9-HMTase 1	6839	Xp11.23	H3K9
**SUV39H2**	KMT1B	79723	10p13	H3K9
**SETDB1**	ESET; KG1T; KMT1E; TDRD21; H3-K9-HMTase4	9869	1q21.3	H3K9
**SETDB2**	CLLD8; CLLL8; KMT1F; C13orf4	83852	13q14.2	H3K9
**SETD2**	HYPB; SET2; HIF-1; HIP-1; KMT3A; HBP231; HSPC069; p231HBP	29072	3p21.31	H3K36
**KMT2E**	MLL5; NKp44L; HDCMC04P	55904	7q22.3	H3K4
**SETD5**		55209	3p25.3	
**SETMAR**	Mar1; HsMar1; METNASE	6419	3p26.1	H3K36
**EZH1**	KMT6B	2145	17q21.2	H3K27
**EZH2**	WVS; ENX1; EZH1; KMT6; WVS2; ENX-1; EZH2b; KMT6A	2146	7q36.1	H3K27
**SETD7**	KMT7; SET7; SET9; SET7/9	80854	4q31.1	H3K4
**KMT2B**	HRX2; MLL2; MLL4; TRX2; WBP7; MLL1B; WBP-7	9757	19q13.12	H3K4
**KMT2C**	HALR; MLL3	58508	7q36.1	H3K4
**SETD1A**	Set1; KMT2F; Set1A	9739	16p11.2	H3K4
**SETD1B**	KMT2G; Set1B	23067	12q24.31	H3K4
**KMT2A**	HRX; MLL; MLL1; TRX1; ALL-1; CXXC7; HTRX1; MLL1A; WDSTS; MLL/GAS7; TET1-MLL	4297	11q23.3	H3K4
**KMT2D**	ALR; KMS; MLL2; MLL4; AAD10; KABUK1; TNRC21; CAGL114	8085	12q13.12	H3K4
**SETD8**	SET8; KMT5A; SET07; PR-Set7	387893	12q24.31	H4K20
**MECOM**	EVI1; MDS1; PRDM3; MDS1-EVI1; AML1-EVI-1	2122	3q26.2	H3K9me1
**PRDM16**	MEL1; LVNC8; PFM13; CMD1LL	63976	1p36.32	H3K9me1
**PRDM13**	PFM10; MU-MB-20.220	59336	6q16.2	
**PRDM8**	PFM5	56978	4q21.21	H3K9
**PRDM1**	BLIMP1; PRDI-BF1	639	6q21	
**PRDM2**	RIZ; KMT8; RIZ1; RIZ2; MTB-ZF; HUMHOXY1	7799	1p36.21	H3K9
**PRDM10**	PFM7	56980	11q24.3	
**PRDM12**	PFM9	59335	9q34.12	
**PRDM6**		93166	5q23.2	
**PRDM14**	PFM11	63978	8q13.3	
**PRDM4**	PFM1	11108	12q23.3	
**PRDM15**	PFM15; ZNF298; C21orf83	63977	21q22.3	
**PRDM5**	BCS2; PFM2	11107	4q27	
**PRDM7**	PFM4; ZNF910	11105	16q24.3	
**PRDM9**	PFM6; MSBP3; PRMD9; ZNF899; MEISETZ	56979	5p14.2	H3K4
**PRDM11**	PFM8	56981	11p11.2	
**SUV420H1**	CGI85; KMT5B; CGI-85	51111	11q13.2	H4K20
**SUV420H2**	KMT5C	84787	19q13.42	H4K20
**ASH1L**	ASH1; KMT2H; ASH1L1	55870	1q22	H3K4; H3K36
**SETD3**	C14orf154	84193	14q32.2	
**SETD4**	C21orf18; C21orf27	54093	21q22.12	
**SETD6**		79918	16q21	
**SMYD1**	BOP; KMT3D; ZMYND18; ZMYND22	150572	2p11.2	H3K4
**SMYD3**	KMT3E; ZMYND1; ZNFN3A1; bA74P14.1	64754	1q44	H3K4
**SMYD2**	KMT3C; HSKM-B; ZMYND14	56950	1q32.3	H3K4; H3K36
**SMYD4**	ZMYND21	114826	17p13.3	
**SMYD5**	RRG1; RAI15; NN8-4AG; ZMYND23	10322	2p13.2	
**NSD1**	STO; KMT3B; SOTOS; ARA267; SOTOS1	64324	5q35.2	H3K36
**WHSC1**	WHS; NSD2; TRX5; MMSET; REIIBP	7468	4p16.3	H3K36; H4K20
**WHSC1L1**	NSD3; pp14328	54904	8p11.23	H3K4; H3K27
**DOT1L**	DOT1; KMT4	84444	19p13.3	H3K79

**Table 2:** Frequency of HMT copy number alterations and mutations

Gene	Gene Location	Amp	Gain	Diploid	Hetloss	Homdel	Mutation
SMYD3	1q44	14.61	58.87	23.17	3.13	0.21	0.52
SETDB1	1q21.3	14.41	57.83	25.89	1.88	0.00	1.15
ASH1L	1q22	12.84	61.17	24.43	1.57	0.00	1.77
WHSC1L1	8p11.23	12.84	24.53	37.37	23.70	1.57	0.52
SMYD2	1q32.3	12.11	61.59	23.28	2.92	0.10	0.21
PRDM14	8q13.3	9.71	45.30	40.08	4.80	0.10	0.31
SUV420H1	11q13.2	5.64	22.65	57.41	14.20	0.10	0.84
SETD1A	16p11.2	5.01	46.87	39.77	8.35	0.00	0.42
MECOM	3q26.2	4.38	27.56	62.53	5.53	0.00	0.63
PRDM1	6q21	2.92	14.51	51.36	30.90	0.31	0.84
SUV39H2	10p13	2.82	23.90	61.48	11.69	0.10	0.10
SUV420H2	19q13.42	2.71	22.44	60.23	14.51	0.10	0.21
SETMAR	3p26.1	2.19	17.95	63.78	15.66	0.42	0.21
SETD5	3p25.3	2.09	17.85	64.61	15.34	0.10	0.52
PRDM9	5p14.2	1.88	32.05	56.05	9.81	0.21	0.73
EZH1	17q21.2	1.46	18.79	45.09	34.13	0.52	0.31
PRDM11	11p11.2	1.46	16.81	64.20	17.33	0.21	0.10
EHMT2	6p21.31	1.36	23.80	60.23	14.61	0.00	0.42
PRDM13	6q16.2	1.36	14.30	51.57	32.57	0.21	0.31
NSD1	5q35.2	1.25	24.84	55.74	17.85	0.31	1.04
PRDM15	21q22.3	1.25	20.98	61.06	16.49	0.21	0.63
PRDM16	1p36.32	0.94	7.20	52.92	38.00	0.94	0.42
SUV39H1	Xp11.23	0.94	15.34	67.01	16.28	0.42	0.21
EZH2	7q36.1	0.84	24.43	57.52	16.91	0.31	0.31
KMT2C	7q36.1	0.84	23.80	55.53	18.79	1.04	6.99
SETD4	21q22.12	0.84	20.88	62.32	15.66	0.31	0.00
PRDM7	16q24.3	0.73	8.87	26.10	61.90	2.40	0.21
SETD8	12q24.31	0.73	16.49	62.94	19.62	0.21	0.31
EHMT1	9q34.3	0.63	14.41	57.83	26.62	0.52	0.94
KMT2E	7q22.3	0.63	25.37	59.50	14.41	0.10	1.15
PRDM12	9q34.12	0.63	13.26	58.56	27.35	0.21	0.10
PRDM8	4q21.21	0.63	11.69	60.65	26.93	0.10	0.42
SETD1B	12q24.31	0.63	16.70	62.42	20.04	0.21	0.63
SETD3	14q32.2	0.63	14.30	55.74	29.02	0.31	0.31
PRDM10	11q24.3	0.52	9.50	43.63	44.78	1.57	0.73
WHSC1	4p16.3	0.52	10.13	57.41	31.21	0.73	0.42
KMT2D	12q13.12	0.42	19.73	65.76	14.09	0.00	2.40
PRDM5	4q27	0.42	10.02	60.54	29.02	0.00	0.31
DOT1L	19p13.3	0.31	10.75	56.37	31.63	0.94	0.84
PRDM4	12q23.3	0.31	16.49	64.82	18.27	0.10	0.31
PRDM6	5q23.2	0.31	18.27	57.20	23.70	0.52	0.10
SETD6	16q21	0.31	9.08	28.71	61.06	0.84	0.00
SETD7	4q31.1	0.31	9.60	60.65	29.33	0.10	0.52
SMYD4	17p13.3	0.31	6.37	34.34	58.25	0.73	0.52
SMYD5	2p13.2	0.31	16.18	68.27	15.14	0.10	0.21
PRDM2	1p36.21	0.21	5.32	53.13	40.81	0.52	0.73
SMYD1	2p11.2	0.21	15.03	69.10	15.55	0.10	0.52
KMT2A	11q23.3	0.10	9.29	41.65	48.02	0.94	1.67
SETD2	3p21.31	0.10	9.29	59.71	30.48	0.42	1.57
SETDB2	13q14.2	0.10	7.52	46.35	43.95	2.09	0.21

Footnote: Amp=high-level amplification; Gain=low-level gain; Hetloss=heterozygous deletion; Homdel= homozygous deletion. Genes were ranked based on the frequency of high-level amplification.

To determine whether the genetic alteration of each HMT is specific to a breast cancer subtype, we performed an independent analysis of copy number and mutation in different subtypes of breast cancer. Of the 958 breast cancer samples, 492 had subtype data available, including 8 normal-like, 220 Luminal A, 121 Luminal B, 55 HER2+, and 88 basal-like breast cancers [6]. Because the normal-like subtype had such a small sample size (n=8), those samples were excluded from this analysis. As shown in Supplementary Tables S1 and S2, basal-like breast cancer had the highest frequencies of HMT gene amplification, deletion, and mutation, whereas Luminal A had the lowest frequencies among the four subtypes in every category of genetic alteration.

**Figure 1:**
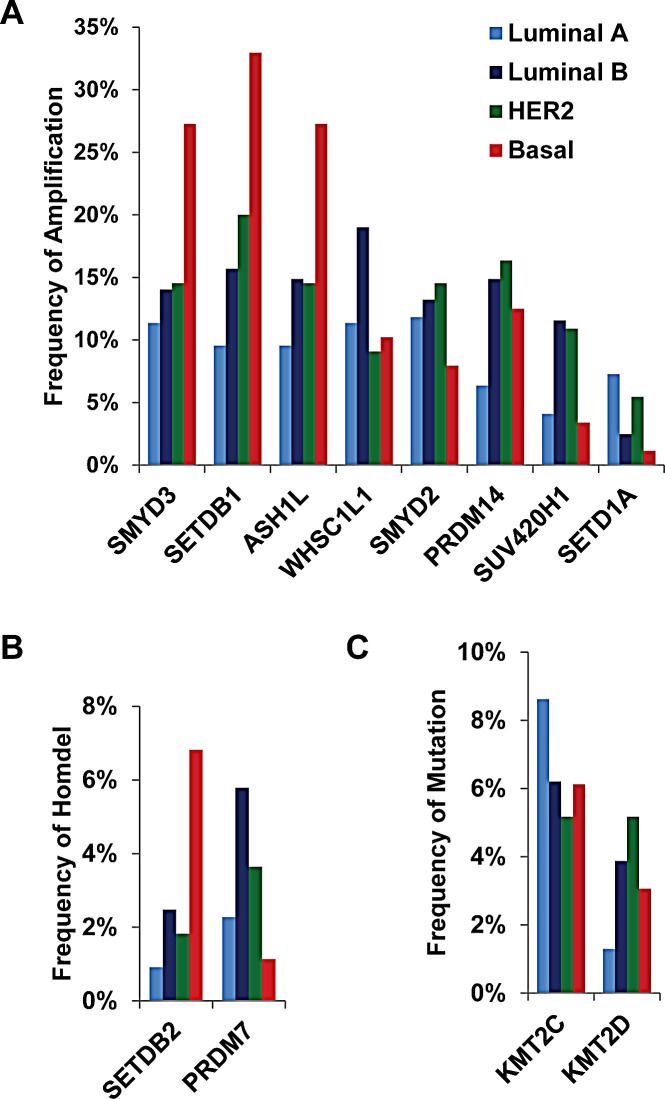
Frequencies of HMTs (A) High-level amplification of eight HMTs, (B) homozygous deletion of two HMTs, and (C) mutation of two HMTs in 492 primary breast cancer specimens from The Cancer Genome Atlas across different types of breast cancer.

In addition, each HMT showed different frequencies of CNA or mutation in different subtypes of breast cancer. Among the eight most frequently amplified HMTs (totaling more than 5% of 958 samples), the frequencies of ASH1L, SETDB1, and SMYD3 amplification were dramatically higher in basal-like breast cancer, with more than 25% of tumors exhibiting high-level amplification compared with the other three subtypes (Figure 1A and Supplementary Table S1). In contrast, WHSC1L1 exhibited the highest frequency of amplification in the Luminal B subtype (19%), and SETD1A had the highest frequency of amplification in the Luminal A subtype (7.27%) (Figure 1A). Of the two most common HMTs with homozygous deletions, SETDB2 exhibited the highest frequency of homozygous deletion (6.82%) in basal-like samples, and PRDM7 was most frequent (5.79%) in Luminal B breast cancer (Figure 1B). Of the most commonly mutated HMTs, KMT2C was most frequently mutated in Luminal A and KMT2D in HER+ breast cancer (Figure 1C). These data indicate that breast cancer, particularly the basal-like subtype, has a high frequency of CNAs and somatic mutations in several HMTs, including amplification of ASH1L, SETDB1, and SMYD3, as well as homozygous deletion of SETDB2.

### Expression profiling of HMTs in breast cancer

Correlation between gene expression and copy number has been used widely to prioritize candidate driver oncogenes in human cancer, because mRNA overexpression can better translate the effect of elevated copy number to cancer initiation and progression. Therefore, we next analyzed the correlation between copy number and mRNA level of 48 HMTs from 958 sequenced breast cancer specimens. Three HMTs (*KMT2B*, *KMT2D*, and *PRDM9*) were excluded from analysis because they lacked RNA sequencing data. To weigh the benefits of different statistical analyses, we compared three different correlation tests—Spearman, Kendall, and Pearson. The rank correlation coefficients among the three statistical tests were similar for the HMTs, specifically by mostly keeping the relative order constant (Table 3). The highest weight was given to the Spearman correlation coefficient, as it measures the relationship between rank-ordered variables and relates the two variables while conserving the order of data points. As shown in Table 3, except for *MECOM*, DNA copy number versus mRNA expression correlations for 47 HMT genes were positive, with 12 of them (*WHSC1L1*, *SETD3*, *SETD6*, *SETDB1*, *SMYD4*, *EZH1*, *SUV420H1*, *EHMT1*, *SETD2*, *SUV39H2*, *PRDM4*, and *SETDB2*) having a Spearman correlation coefficient (r) greater than 0.5. Among the 48 HMTs, WHSC1L1 had the highest correlation by both Spearman (r=0.737) and Kendall (r=0.604) analyses, consistent with our previous findings that *WHSC1L1* is an amplified gene in breast cancer [12, 21].

**Table 3:** Associations between CNA and expression, and comparison of mRNA expression between basal and nonbasal breast cancer subtypes

Gene	DNA/mRNA Correlation			Basal/non-basal Comparison	
	Spearman	Kendall	Pearson	T Stat	P value
WHSC1L1	0.737	0.604	0.460	−1.815	3.65E-02
SETD3	0.663	0.545	0.612	−4.291	2.30E-05
SETD6	0.625	0.503	0.615	4.671	5.41E-06
SETDB1	0.624	0.506	0.560	4.115	4.38E-05
SMYD4	0.614	0.498	0.626	−0.344	3.66E-01
EZH1	0.612	0.485	0.562	−6.282	6.41E-09
SUV420H1	0.553	0.448	0.599	−0.742	2.30E-01
EHMT1	0.543	0.440	0.547	−7.336	5.42E-11
SETD2	0.520	0.417	0.500	−3.102	1.30E-03
SUV39H2	0.507	0.406	0.573	8.950	2.84E-14
PRDM4	0.505	0.409	0.516	−4.631	6.31E-06
SETDB2	0.501	0.398	0.433	−2.541	6.41E-03
SETD1B	0.481	0.385	0.451	−4.121	4.29E-05
EHMT2	0.478	0.386	0.470	5.621	1.13E-07
SETD1A	0.471	0.377	0.451	0.294	3.85E-01
SETD4	0.430	0.347	0.462	0.689	2.46E-01
PRDM2	0.428	0.344	0.422	−1.400	8.25E-02
SETD5	0.418	0.337	0.469	1.931	2.84E-02
SMYD5	0.402	0.324	0.451	3.632	2.38E-04
KMT2A	0.401	0.316	0.404	0.881	1.90E-01
SETMAR	0.388	0.309	0.435	−1.719	4.46E-02
SETD8	0.367	0.292	0.387	3.239	8.50E-04
NSD1	0.358	0.283	0.387	−1.936	2.81E-02
PRDM11	0.355	0.281	0.372	1.557	6.15E-02
WHSC1	0.345	0.278	0.365	3.625	2.43E-04
SMYD2	0.343	0.270	0.336	7.273	7.23E-11
PRDM15	0.339	0.267	0.367	3.895	9.63E-05
ASH1L	0.317	0.248	0.310	−2.722	3.92E-03
SETD7	0.316	0.252	0.325	−9.245	7.08E-15
SMYD3	0.311	0.242	0.213	−6.056	1.73E-08
EZH2	0.300	0.235	0.317	8.004	2.43E-12
PRDM10	0.278	0.220	0.309	−3.070	1.43E-03
SUV420H2	0.267	0.211	0.329	−0.384	3.51E-01
KMT2C	0.261	0.205	0.262	−2.557	6.15E-03
PRDM13	0.252	0.224	0.166	4.209	3.11E-05
DOT1L	0.237	0.188	0.268	2.605	5.40E-03
PRDM7	0.227	0.200	0.177	2.538	6.47E-03
PRDM6	0.197	0.155	0.031	−6.342	4.91E-09
KMT2E	0.194	0.154	0.210	−2.351	1.05E-02
PRDM5	0.154	0.123	0.200	−0.757	2.26E-01
PRDM14	0.123	0.115	0.057	−1.054	1.47E-01
PRDM12	0.110	0.086	0.113	1.168	1.23E-01
PRDM16	0.092	0.074	0.122	0.495	3.11E-01
PRDM1	0.068	0.053	0.086	2.238	1.39E-02
SMYD1	0.063	0.050	−0.002	1.419	7.98E-02
PRDM8	0.035	0.027	0.045	0.380	3.52E-01
SUV39H1	0.016	0.011	0.077	5.415	2.69E-07
MECOM	−0.079	−0.063	−0.034	−0.261	3.97E-01

Footnote: Genes were ranked based on the Spearman correlation coefficient. Significantly higher expression of HMTs in the basal-like subtype is highlighted in dark gray, and significantly lower expression is highlighted in little gray. T stat represents the T statistic, which is equivalent to the number of standard deviations of mRNA expression levels between the basal-like and nonbasal breast cancer samples. A positive value means that the basal-like subtype has a higher value, and a negative value means that the basal-like subtype has a lower value than the nonbasal breast cancer samples.

**Figure 2:**
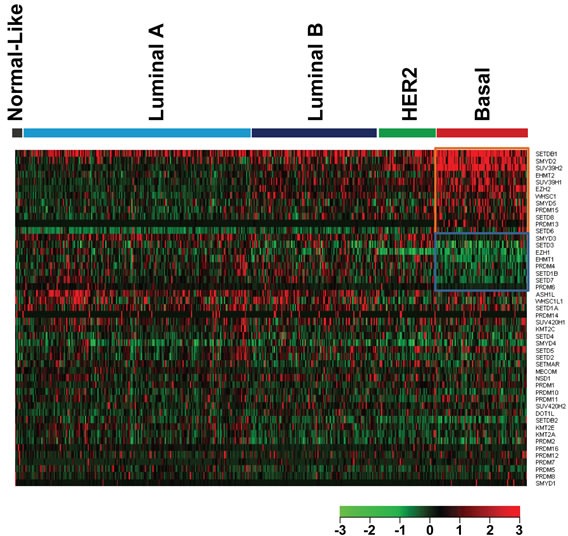
Heatmap of HMT expression profiles in different types of breast cancer The breast cancer samples used in this analysis included 8 normal-like, 220 Luminal A, 121 Luminal B, 55 HER2+, and 88 basal-like breast cancers. Significantly higher-expressed genes (p<0.001) in basal-like tumors are shown at the top, indicated by a red box; and lower-expressed genes (p<0.001) in basal-like tumors are indicated by a blue box.

Basal-like breast cancer, the most aggressive subtype, is associated with higher rates of metastasis and death [22]. We next sought to compare expression levels of the 48 HMTs between basal and non-basal subtypes in the 492 TCGA breast cancer samples with subtype information. The significance of difference for each HMT between the basal-like and the other subtypes was calculated using Student's *t*-test. In the basal-like subtype, compared with non-basal subtypes, we found that the expression levels of 12 HMTs (*SETDB1*, *SMYD2*, *SUV39H2*, *EHMT2*, *SUV39H1*, *EZH2*, *WHSC1*, *SMYD5*, *PRDM15*, *SETD8*, *PRDM13*, and *SETD6*) were significantly higher (p<0.001), and the expression levels of 8 HMTs (*SMYD3*, *SETD3*, *EZH1*, *EHMT1*, *PRDM4*, *SETD1B*, *SETD7*, and *PRDM6*) were significantly lower (p<0.001) (Figure 2 and Table 3). Notably, our analysis validated that *EZH2* exhibits significantly higher expression in basal-like breast cancer, consistent with previous results [14, 23]. Conversely, *WHSC1L1* showed moderately higher expression (t=−1.815, p=0.036) in non-basal subtypes, which supports our other findings that it is amplified more in Luminal subtypes and shows high correlation between copy number and mRNA expression (Figure 1A, Table 2) [12, 21].

### KMT2C and KMT2D mutations in breast cancer

**Figure 3:**
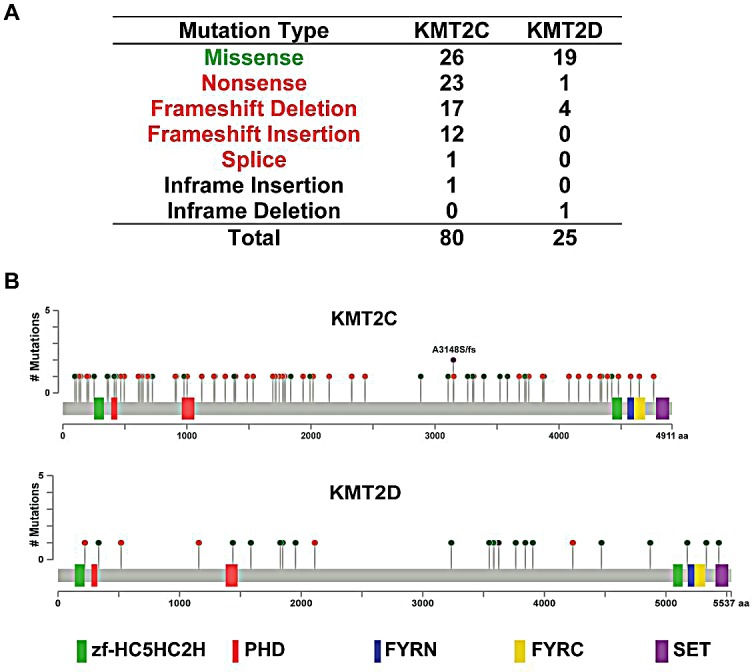
*KMT2C* and *KMT2D* mutational spectrum in breast cancer (A) Frequency of each mutation type for KMT2C from 958 breast cancer samples. The data were obtained from The Cancer Genome Atlas database via cBioPortal. (B) The images show protein domains and the positions of specific mutations of *KMT2C* and *KMT2D*. A red dot indicates a nonsense mutation, frameshift deletion, insertion, or splice; a green dot indicates a missense mutation; and a black dot indicates an inframe insertion or deletion.

Because *KMT2C* and *KMT2D* are the most frequently mutated HMT genes in breast cancers, at rates of 6.99% and 2.40%, respectively (Table 2), we performed a comprehensive analysis of the *KMT2C* and *KMT2D* mutation spectrum in 958 breast cancer samples. As shown in Figure 3, we identified a total of 80 *KMT2C* mutations, consisting of 26 missense mutations, 23 nonsense mutations, 17 frameshift deletions, 12 frameshift insertions, 1 splice, and 1 inframe insertion. Eight tumor samples had two mutations, and two samples had four mutations in the *KMT2C* gene. For example, sample TCGA-AC-A23H contained three missense mutations (D3264N, E3724K, and D4344H) and one nonsense mutation (Q1218*). In the *KMT2D* gene, 25 mutations were identified, most of them missense mutations (Figure 3A). KMT2C and 2D are large proteins (approximately 5000 amino acids) that contain the zf-HC5HC2H, PHD, FYRN, and FYRC domains and the carboxy-terminal SET domain. Figure 3B shows the distribution of KMT2C and KMT2D mutations in 958 breast cancer samples across protein domains; most of the mutations are localized to the amino-terminal end of the SET domain. Previous studies demonstrated that mice lacking the KMT2C catalytic SET domain developed ureteric tumors, which supports its hypothesized role as a tumor suppressor [24]. Therefore, we predict that mutations at the amino terminus of KMT2C and KMT2D SET domains (Supplementary Table S3) might result in the truncation of the SET domain or loss of function of KMT2C and/or KMT2D methyltransferases, subsequently contributing to breast cancer initiation and progression.

### Amplification/overexpression of multiple HMTs from chromosome 1q

**Figure 4:**
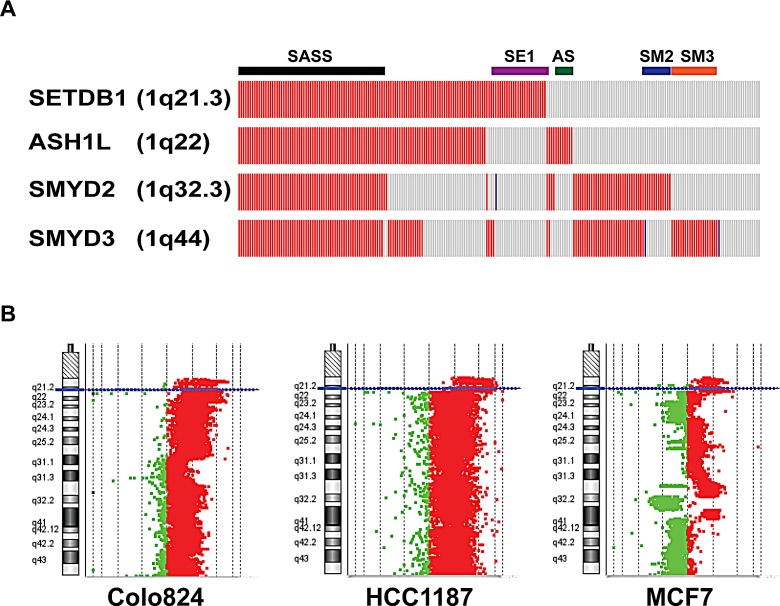
High-level amplification of four HMT genes at chromosome 1q in breast cancer (A) Copy numbers of *SETDB1*, *ASH1*, *SMYD2*, and *SMYD3* were obtained from the Oncoprint output of The Cancer Genome Atlas breast cancer data (cBioPortal). A red rectangle indicates high-level amplification; blue rectangle, homozygous deletion; grey rectangle, unaltered. SASS represents amplification of all four (*SETDB1*, *ASH1*, *SMYD2*, and *SYMD3*) loci; SE1 represents *SETDB1* locus only; AS, *ASH1L* only; SM2, *SMYD2* only; and SM3, *SMYD3* only. (B) Agilent array of comparative genomic hybridization profiles shows amplification/gain of chromosome 1q in three breast cancer cell lines (Colo824, HCC1187, and MCF7, also see Supplementary Table S4).

Among the five most frequently amplified HMT genes (frequency>10%) in breast cancers, four were localized on the long arm of chromosome 1, with *SETDB1* at 1q21.3, *ASH1L* at 1q22, *SMYD2* at 1q32.3, and *SMYD3* at 1q44 (Figure 4). Of the 958 breast cancer samples, 215 (22.44%) contained high-level amplification in at least one locus of these four genes (Figure 4A). Of those 215 samples, 65 had amplification in all four loci, while 23 samples were amplified only at *SETDB1*, 8 only at *ASH1L*, 12 only at *SMYD2*, and 21 only at *SMYD3* (Figure 4A). In addition, 111 of 215 samples were co-amplified at ASH1L and SETDB1, and this amplicon (1q21-22) spans approximately 4 Mb in basal-like breast cancer [6]. We assume that in the 65 samples containing co-amplification of SETDB1, ASH1L, SMYD2, and SMYD3, the whole arm of chromosome 1q is amplified. We found that amplification of the whole arm of 1q is more common in Luminal subtypes (Luminal A, 7.73%, Luminal B, 8.26%) than in HER2+ (5.45%) and basal (5.68%) subtypes.

In breast cancer, cell lines mirror many of the molecular characteristics of the tumors from which they were derived, so we used a panel of breast cancer cell lines to further investigate the genetic landscape of HMTs, particularly the amplification and overexpression of the four HMTs on chromosome 1q. We first analyzed our own genomic array of comparative genomic hybridization (CGH) data as well as publicly available data for 17 breast cancer cell lines [12, 21, 25]. Seven lines (Colo824, SUM229, SUM149, HCC70, HCC1937, HCC1187, and MDB-MA-468) were of the basal-like subtype, three lines (HCC1954, SUM190, and SUM225) were of the HER2+ subtype, and seven (HCC1428, SUM44, SUM52, T47D, SUM185, ZR-75-1, and MCF7) were of the Luminal subtypes. We found that, similar to primary breast cancer samples, 16 of 17 lines showed gain or amplification in at least one locus of *SETDB1*, *ASH1L*, *SMYD2*, and *SMYD3*. Six lines (Colo824, HCC70, HCC1187, MDB-MA-468, HCC1954, and ZR-75-1) showed low-level gain across most of chromosome 1q, whereas Colo824 and HCC1187 showed high-level amplification at the *SETDB1* locus (Figure 4B and Supplementary Table S4). Notably, MCF7 showed moderate amplification at the SETDB1 locus as well as heterozygous deletions at the *ASH1L* and *SMYD2* loci (Figure 4B and Supplementary Table S4). In addition, consistent with our findings in primary breast cancers, we also found that other HMTs, such as *WHSC1L1*, *SUV420H1*, and *SETD1A*, were commonly gained or amplified, and *SETDB2* was commonly lost or deleted in breast cancer cell lines (Supplementary Figure S2).

**Figure 5:**
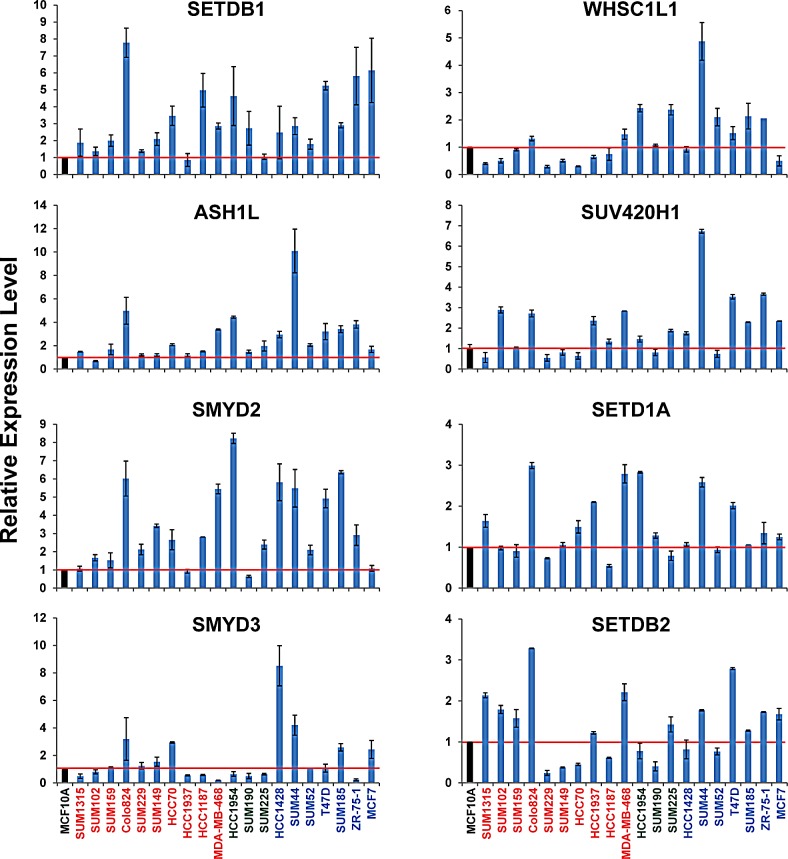
mRNA expression levels of eight HMTs in a panel of 20 breast cancer cell lines measured by qRT-PCR mRNA expression levels in the MCF10A cells, an immortalized but non-tumorigenic breast epithelial cell line, were arbitrarily set as 1. Relative expression levels are shown as fold changes compared with that in MCF10A cells.

Next, we performed quantitative RT-PCR (qRT-PCR) analysis to measure the mRNA expression level of eight HMTs (*SETDB1*, *ASH1L*, *SMYD2*, *SMYD3*, *SETD1A*, *WHSC1L1*, *SUV420H1*, and *SETDB2*) in 20 breast cancer cell lines. MCF10A, an immortalized but non-tumorigenic breast epithelial cell line, was used as the control. These eight HMTs were chosen because seven of them showed the highest frequency of amplification (>5%), and the eighth, *SETDB2*, exhibited homozygous deletion in more than 2% of breast cancers. Although *PRDM14* and *PRDM7* were also among the HMTs with the highest frequency of amplification or homozygous deletion, respectively, in breast cancer, they were excluded from the qRT-PCR analysis because their expression levels in breast cancer cells were too low for detection (data not shown). Figure 5 shows the relative expression of eight HMT genes in 20 breast cancer cell lines compared with MCF10A cells. We found that mRNA levels of *SETDB1* were more than two-fold higher in 14 of 20 breast cancer cell lines. For *ASH1L*, 11 of 20 cell lines; for *SMYD2*, 14 of 20; and for *SMYD3*, 6 of 20 breast cancer cell lines had two-fold higher mRNA levels. Notably, Colo824 showed higher expression of four 1q HMTs, with the highest expression of *SETDB1* among the 20 breast cancer cell lines. In contrast, MCF7 showed higher expression of *SETDB1*, but not *ASH1L* and *SMYD2*, which is consistent with amplification of *SETDB1*, but not *ASH1L* and *SMYD2*, in this breast cancer cell line (Figure 5). We also found that *SMYD3* and *WHSC1L1* were prevalently overexpressed in several Luminal breast cancer cell lines. Breast cancer cell lines with deletion of *SETDB2*, such as SUM229, SUM149, HCC170, and SUM190, showed dramatically lower mRNA levels than MCF10A cells (Figure 5). Similar results were observed in RNA sequencing (RNA-Seq) data from 42 breast cancer cell lines compared with five normal mammary epithelial cell lines (Supplementary Figure S3). These experiments demonstrated that breast cancer cell lines with HMT gene CNAs showed a correlated change in mRNA expression. Thus, these cell lines provide useful preclinical models in which to investigate the biological functions of HMTs and to explore novel inhibitors for targeting HMTs in breast cancer in the future.

### HMT copy number and expression are associated with breast cancer patient survival

**Figure 6:**
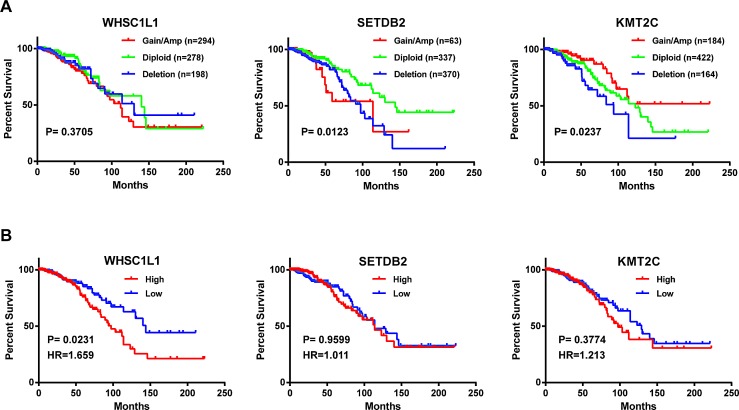
Kaplan-Meier plots of overall survival associated with (A) copy number and (B) mRNA expression levels of three HMTs (WHSC1L1, SETDB2, and KMT2C) in breast cancer

To investigate the clinical relevance of genetic alterations of HMTs in breast cancer, we examined the relationship between HMT copy number, mRNA expression, and overall patient survival in 770 of 958 breast cancer samples for which detailed survival data were available. To investigate DNA copy number, samples were segregated into the following three groups for each HMT: amp/gain (high-level amplification and low-level gain), diploid, or deletion (heterozygous and homozygous deletion). Log-rank statistical analysis was first used to determine whether an increase or decrease in copy number for each HMT was associated with overall patient survival. We found that for seven HMTs (KMT2C, SETDB2, SETD2, SETMAR, PRDM1, PRDM5, and PRDM8), copy number amp/gain or deletion was significantly associated (p<0.05) with shorter survival in breast cancer patients (Figure 6A and Supplementary Table S5). Both deletion and amp/gain of SETDB2 and SETMAR were associated with shorter patient survival, whereas only amp/gain of PRDM1, PRDM5, and PRDM8 was more likely associated with shorter patient survival (Supplementary Table S5). Most importantly, we discovered that deletion of KMT2C was significantly associated with shorter survival, and amp/gain of this gene was significantly associated with longer survival, compared with patients who had no change in copy number (Figure 6A). However, KMT2C, which has the highest genetic mutation rate of HMTs in breast cancer, showed no significant difference in patient survival with regard to mutation (Supplementary Figure S4).

To analyze the relationships between HMT mRNA expression and overall patient survival in breast cancer, samples were divided into low (n=385) and high (n=385) groups based on the mRNA expression Z-scores [RNA-Seq V2 RSEM (RNA-Seq by Expectation-Maximization)] of each HMT. Seven HMTs were excluded from survival analysis of mRNA expression because three of them (KMT2B, KMT2D, and PRDM9) lacked RNA sequencing data, and the expression levels for four of them (PRDM7, PRDM13, PRDM14, and SMYD1) were too low to be convincing, as previously noted. Supplementary Table S6 summarizes the results of a log-rank statistical analysis for 44 HMTs in breast cancer. High mRNA levels of WHSC1L1 (p=0.0231), SETD7 (p=0.0021), and SETD5 (p=0.0456) were significantly associated (p<0.05) with shorter survival in breast cancer patients (Figure 6B, Supplementary Table S6). For WHSC1L1, higher mRNA expression had a hazard ratio (HR), a ratio of the probability of death, of 1.659 (95% confidence interval, 1.074 to 2.571) compared with lower mRNA expression in breast cancer. Additionally, high mRNA levels of SUV39H2 (p=0.0506), KMT2A (p=0.0644), NSD1 (p=0.0701), and ASH1L (p=0.0877) showed moderately significant association (p<0.1) with shorter survival in patients with breast cancer (Supplementary Table S6). In contrast, only two HMTs, SETD8 (p=0.0769) and EHMT2 (p=0.0978), displayed low mRNA levels that were moderately associated with shorter survival in patients. KMT2C, which had the highest genetic mutation rate among HMTs in breast cancer, showed no significant difference (p=0.3774) in patient survival in terms of mRNA expression levels (Figure 6B).

We then performed a multivariate analysis (Cox model, n=468) to investigate whether the copy number and/or expression level of each HMT were predictive of poor prognosis compared with standard prognostic markers, including age at diagnosis, ER status, PR status, HER2 status, tumor size, lymph node status, metastasis status, and molecular subtype (basal vs. non-basal). We found that copy number amp/gain of NSD1 or PRDM1, or loss of SETDB2 or PRDM10 was significantly associated (p<0.05) with shorter survival in breast cancer patients (Supplementary Table S7). In addition, we found that high mRNA levels of SETD4 (p=0.0468, HR=1.92), SETD5 (p=0.00231, HR=2.79), or SETD7 (p=0.0391, HR=1.97) were significantly associated (p<0.05) with shorter survival in breast cancer patients (Supplementary Table S8). However, amp/gain or high expression level of SMYD3 was negatively correlated with shorter survival in breast cancer patients (Supplementary Tables S7 and S8).

### Integrative identification of critical HMTs in breast cancer

The preceding results suggested the possibility of ranking the importance of HMTs in breast cancer according to CNAs, mutation, mRNA expression, and clinical relevance. We calculated a score for each HMT, where every category counted as one point: when an HMT had high-level amplification (frequency >5%), homozygous deletion (frequency >2%), or mutation (frequency >2%); CNA associated with patient survival (log-rank test p<0.1); DNA/mRNA correlation (r > 0.5, p<0.001); altered expression in aggressive basal-like breast cancer (p<0.001); and mRNA associated with shorter patient survival (log-rank test p<0.1). We then ranked the HMTs, as shown in Table 4, observing that three HMTs (SETDB1, WHSC1L1, and SETDB2) had a score of 3, and five HMTs (ASH1L, SMYD2, SMYD3, SUV420H1, and KMT2C) had a score of 2. These results suggest that these eight HMTs act as drivers of oncogenic processing or as tumor suppressors and have critical roles in breast cancer initiation and progression.

**Table 4:** Integrative identification of critical HMTs in breast cancer

Gene	CNA/Mutations	CNA/Survival	DNA/mRNA	Expression	mRNA/Survival	Score
ASH1L	+				+	2
SETDB1	+		+	+		3
SMYD2	+			+		2
SMYD3	+			+		2
WHSC1L1	+		+		+	3
PRDM14	+					1
SUV420H1	+		+			2
SETD1A	+					1
PRDM7	+					1
SETDB2	+	+	+			3
KMT2C	+	+				2
KMT2D	+					1

Footnote: CNA/Mutation: high-level amplification, homozygous deletion, or mutation; CNA/Survival: CNA associated with patient survival; DNA/mRNA: DNA/mRNA correlation; Expression: altered expression in aggressive basal-subtype ; and mRNA/Survival: mRNA associated with patient survival.

## DISCUSSION

We performed comprehensive genomic and transcriptomic analysis of 51 human HMTs in a panel of breast cancer cell lines and in primary breast cancer samples. Our main findings include the following: (1) we identified 12 HMTs with the highest frequency of genetic alterations, including eight with high-level amplification, two with putative homozygous deletion, and two with somatic mutation; (2) there was a correlation between gene expression and copy number: most HMTs had a positive correlation, and WHSC1L1 had the highest correlation coefficient in breast cancer; (3) different subtypes of breast cancer have different patterns of copy number and expression for each HMT gene; several, including SETDB1 and SMYD2, were highly amplified and overexpressed in basal-like breast cancer; (4) the two most mutated HMTs, KMT2C and KMT2D, exhibited various types of mutation across protein domains, possibly resulting in loss of their methyltransferase functions; (5) chromosome 1q contains four HMTs that are concurrently or independently amplified/overexpressed in breast cancer cell lines and primary samples; (6) we identified several HMTs, including WHSC1L1, SETDB2, and KMT2C, whose DNA copy number or mRNA expression level was significantly associated with shorter survival in breast cancer patients; and (7) integrative analysis prioritized SETDB1, WHSC1L1, and SETDB2 as the most critical HMTs in breast cancer.

The HMTs constitute a large class of enzymes that catalyze site-specific methylation of lysine residues on histones and other proteins [7-9]. Previously, oncogenic alterations, including amplification, mutation, and translocation of several HMTs, were identified in various human tumors, including breast cancer [8, 10, 11, 26, 27]. A well-studied example is EZH2, the catalytic subunit of the polycomb repressive complex 2. Hyperactivation of EZH2, by amplification/overexpression or mutation, was documented in breast and prostate cancers, lymphoma, and other types of tumors [28]. Tissue microarray analysis revealed that EZH2 protein levels were strongly associated with breast cancer aggressiveness [13, 29]. Another example is *WHSC1L1*, a commonly amplified gene at 8p11-12 in breast and lung cancers; *WHSC1L1* is fused with NUP98 in acute myeloid leukemia [12, 30-32]. Evidence indicated that the 8p11-12 amplicon contained several candidate oncogenes in breast cancer [12]. As shown in Supplementary Figure S5, we found that most WHSC1L1-amplified breast cancer samples also showed the amplification of other candidate oncogenes at the 8p11-12 amplicon. Notably, our previous study demonstrated the oncogenic potential of WHSC1L1, particularly in ER+ Luminal breast cancer [12]. Although previous studies revealed the dysregulation of individual HMTs in breast cancer, to our knowledge, this is the first report showing comprehensive genomic and transcriptomic analysis of 51 HMTs in different types of breast cancer [10-12]. This study validates previous findings in individual HMTs, namely that WHSC1L1 is highly amplified/overexpressed in Luminal subtypes, and EZH2 is highly expressed in basal-like breast cancer [12, 23, 27]. Importantly, our results reveal the genomic landscape for many HMTs in different subtypes of breast cancer.

Previous studies demonstrated a prevalent gain/amplification of chromosome 1q in breast cancer [33-35]. Of the five HMTs that have high-level amplification in more than 10% of breast cancer samples, four are clustered on chromosome 1q, from 1q21 to 1q44. We found that, of 215 breast cancer samples in which chromosome 1q is amplified, 65 (30%) had concurrent high-level amplification in all four genetic loci; this amplification is more common in Luminal breast cancer. Furthermore, detailed genomic analysis showed that 1q amplifications were heterogeneous in most breast cancer cell lines and primary samples. The *SETDB1* gene (1q21.3) encodes the H3K9 methyltransferase and showed independent amplification in 23 of 215 primary breast cancers as well as the MCF7 breast cancer cell line. We found that amplification or overexpression of SETDB1 is more common in basal-like breast cancer (Figure 1A, Figure 2, and Supplementary Table S9). Recent studies demonstrated that *SETDB1* is frequently amplified in lung and urothelial cancers as well as melanoma [36-38]. The inhibition of SETDB1 expression in *SETDB1*-gene-amplified lung cancer cells reduced tumor growth in cell culture and nude mice models, whereas its overexpression increased tumor invasiveness [36]. SETDB1 significantly accelerated melanoma formation in a zebrafish model [38]. *SMYD2* and *SMYD3*, which share a high degree of sequence homology, are localized at 1q32 and 1q44, respectively. We queried published TCGA breast cancer data and found that the size of the 1q44 (SMYD3) amplicon spans approximately 1.5 Mb in breast cancer [6]. Recent studies demonstrated that SMYD2 and SMYD3 can methylate both histone and nonhistone proteins, such as the tumor suppressors p53 and Rb as well as the ERα protein [39-41]. Enzymatic analysis shows that SMYD2 is a monomethyltransferase that prefers nonmethylated p53 peptide as a substrate among different histones and protein substrates tested *in vitro* [42]. Conversely, another study demonstrated the role of SMYD3 in ER-mediated transcription *via* its histone methyltransferase activity [43]. Furthermore, SMYD3 activity regulates cytoplasmic oncogenic signaling; specifically, SMYD3-mediated MAP3K2 methylation activates RAS signaling and drives carcinogenesis *in vivo* [44]. Additionally, ASH1L is an H3K4 and H3K36 histone methyltransferase that occupies the transcribed region of active genes [45]. Notably, H3K4 demethylase KDM5B (also known as JARID1B), which is localized at 1q32.1, was recently shown to be an oncogene in Luminal breast cancer by regulating lineage-specific genes [46]. Thus, chromosome 1q contains multiple oncogenic histone lysine modifiers that are concurrently or independently amplified or overexpressed in breast cancer. It is necessary to investigate whether or how these five histone lysine modifiers contribute, independently or cooperatively, to breast tumorigenesis.

Two HMT genes demonstrated high (>2%) rates of homozygous deletion among breast cancer samples, implying their potential roles as tumor suppressors. Of the two, *PRDM7* is the more mysterious gene. Most studies of *PRDM7* to date investigated its phylogenetic and evolutionary ancestry [47, 48]. The other gene, *SETDB2,* which was concurrently deleted with another tumor suppressor, RB1, at 13q14 (Supplementary Figure S5A), is a homologue of *SETDB1* and participates in the distribution of trimethylated H3K9 (H3K9me3) marks and contributes to chromosome segregation during mitosis [49]. However, although SETDB1 and SETDB2 are homologues, they seem to have opposing functions with regard to breast cancer. Structurally, the most significant difference between the two proteins is the presence or absence of a Tandem-Tudor domain. The Tandem-Tudor domain functions essentially as the guiding module that allows a protein to recognize and bind to the appropriate histone, where it can regulate methylation reactions. For example, the Tandem-Tudor domain of 53BP1 has a direct role in recognizing and binding H4K20 dimethylation (H4K20me2) to promote DNA repair [50]. In addition, the Tandem-Tudor domain of histone H3K9 and H3K36 demethylase KDM4A recognizes and binds H3K4me3 and H4K20me3/me2 marks [51, 52]. However, the histone site and marks that the SETDB1 Tandem-Tudor domain recognizes are still not known. We still need to investigate how the presence of the Tandem-Tudor domain in SETDB1 differentiates it from SETDB2 so dramatically.

We identified the two most mutated HMT genes in breast cancer. *KMT2C* was mutated in 67 of 958 breast cancer specimens (6.99%), which is almost three times more than *KMT2D* with a rate of 2.40%. *KMT2C* and *KMT2D* belong to a set of genes known as mixed-lineage leukemia (MLL) genes and have the aliases *MLL3* and *MLL4*, respectively. Translocation mutations cause the generated MLL proteins to lose their SET domain and fuse with other proteins to create fusion proteins, which have been identified as direct causes of aggressive leukemia [53]. In terms of function, KMT2C and KMT2D are monomethylases for the lysine residue H3K4 and are required to generate H3K4me1 at various enhancer regions throughout the genome [54]. It has been proposed that cancer-associated mutations in *KMT2C* and *KMT2D* exert their properties through the malfunction of KMT2C/KMT2D-dependent enhancers [55]. In addition, KMT2C/D can associate with activating signal cointegrator-2 (ASC-2), a multifunctional coactivator, to form an ASC complex, which has been identified as a key factor in the DNA damage response and in p53 activation [56]. Our data, which demonstrate that *KMT2C* is commonly mutated and that its deletion is significantly associated with shorter patient survival, suggests that KMT2C might function as a tumor suppressor in breast cancer.

Because epigenetic changes are reversible and HMTs are druggable, targeting HMTs provides a unique opportunity for pharmacologic intervention by means of designing inhibitors that represent a novel class of anti-cancer drugs. Recent evidence shows that aberrant activity of HMTs, due to amplification, deletion, or mutation of their corresponding genes, contributes to cancer initiation and progression. Consequently, a promising strategy could be to target patient populations with those alterations. Selective inhibitors of several HMTs have been reported to show antitumor effects *in vitro* and *in vivo* [57]. Furthermore, an EZH2 inhibitor (EPZ-6438) has entered phase I human clinical trials. Here, we reported a systematic and integrative analysis of HMTs and identified eight HMTs (SETDB1, SMYD3, ASH1L, SMYD2, WHSC1L1, SUV420H1, SETDB2, and KMT2C) that have the highest frequencies of genetic alterations and most clinical relevance. Our findings for these HMTs represent a strong foundation for further mechanistic research and therapeutic advances in breast cancer.

## MATERIALS AND METHODS

### Cell culture

The cultures for the SUM series of breast cancer cell lines and nontransformed human mammary epithelial cell MCF10A line have been described in detail previously [21, 58]. The Colo824 cell line was obtained from DMSZ, and the cell lines HCC70, HCC1187, HCC1428, HCC1937, HCC1954, MDB-MA-468, T47D, and ZR-75-1 were obtained from ATCC. These lines were maintained in RPMI with 10% FBS (Atlanta Biologicals, Flowery Branch, GA, USA) according to DMSZ and ATCC protocols.

### Genomic array CGH

Genomic array CGH experiments were done using the Agilent human genome CGH microarray chip (Agilent Technologies, Palo Alto, CA, USA) as previously described in detail [21]. Agilent's CGH Analytics software was used to calculate various measurement parameters, including log2 ratios of total integrated Cy-5 and Cy-3 intensities for each probe. Array data have been posted in the NCBI GEO database (GEO accession numbers: GSM718287, GSM718288, GSM718290).

### Semiquantitative RT-PCR reactions

mRNA was prepared from human breast cancer cell lines and the MCF10A cell line by using an RNeasy Plus Mini Kit (QIAGEN). mRNA was mixed with qScript cDNA SuperMix (Quanta Biosciences, Gaithersburg, MD, USA), then converted into cDNA through a reverse-transcription reaction for real-time PCR reactions. Primer sets for HMT genes were ordered from Life Technologies (Carlsbad, CA, USA). A PUM1 primer set was used as a control. Semiquantitative RT-PCR was done using the FastStart Universal SYBR Green Master (Rox) (Roche Diagnostics Indianapolis, IN, USA).

### Genomic and clinical data

The DNA copy number, mutation, and overall survival datasets of 958 breast cancer samples used in this research were obtained from the cBio Cancer Genomics Portal at http://www.cbioportal.org. The copy number for each HMT was generated from the copy number analysis algorithms GISTIC and indicates the copy number level per gene. “-2” is a deep loss (possibly a homozygous deletion), “-1” is a heterozygous deletion, “0” is diploid, “1” indicates a low-level gain, and “2” is a high-level amplification. For mRNA expression data, the relative expression of an individual gene and the gene's expression distribution in a reference population were analyzed. The reference population was either all tumors that are diploid for the gene in question, or, when available, normal adjacent tissue. The returned value indicates the number of standard deviations away from the mean of expression in the reference population (Z-score). The breast cancer subtype information was from a previous publication [6].

### Statistical analysis

Statistical analyses were performed using the R software (http://www.r-project.org) and Graphpad Prism (version 6.03). The correlations between copy numbers and mRNA levels of each HMT from 958 sequenced breast cancer specimens were analyzed using Spearman, Kendall, and Pearson correlation tests. The Spearman and Kendall tests are rank correlations—the Spearman coefficient relates the two variables conserving the order of data points, and the Kendall coefficient measures the number of ranks that match in the data set. Although the Pearson correlation coefficient is the most widely used, it was deemed the least relevant to our study, as it measures only the strength of linear relationships and ignores all others. We used the “cor” function in R statistical software for computation, specifying in the code which type of test we wanted (Spearman, Kendall, or Pearson). A systematic approach was used to analyze the correlations between the mRNA levels and DNA copy numbers for each of the 48 HMTs for all three of the methods. The difference in mRNA expression level for each HMT between the basal-like and the other cancer subtypes was calculated using Student's *t*-test. The association between the clinical outcome and individual HMT copy number and expression level was evaluated using a log-rank test. Multivariate survival analysis was conducted using the Cox regression function in R statistical software.

## SUPPLEMENTARY MATERIALS AND METHODS FIGURES AND TABLES




















