# Supplementary material for: Genetic alterations of histone lysine methyltransferases and their significance in breast cancer
Source: Oncotarget. 2014 Dec 11;6(4):2466–82. doi: 10.18632/oncotarget.2967 (PMC4385864; doi:10.18632/oncotarget.2967)
Supplement: Supplementary file 1 [file oncotarget-06-2466-s001.pdf]

Genetic alterations of histone lysine methyltransferases and their significance in breast cancer

Supplementary Materials and Methods

Phylogenetic tree of the HMT superfamily

The phylogeny outlined in the tree is derived from a CLUSTALW multiple sequence alignment of the full-length sequence of the default Swiss-Prot variant (HMT genes). The CLUSTALW uses a "progressive alignment" algorithmic approach, which entails calculating pairwise sequence alignment scores between all the proteins being aligned and then beginning the alignment with the two closest sequences and progressively adding more sequences to the alignment.

GISTIC algorithm

In cBioPortal, the Genomic Identification of Significant Targets in Cancer (GISTIC) algorithm was used to determine the copy number status of each gene in each sample [1, 2]. The GISTIC takes segmented copy number ratios as input, separates chromosome arm-level events from focal events, and then performs two tests: (i) it identifies significantly amplified/deleted chromosome arms; and (ii) it identifies regions that are significantly focally amplified or deleted. Each aberration is assigned a G-score that considers the amplitude of the aberration as well as the frequency of its occurrence across samples. False-discovery rate q-values are then calculated for the aberrant regions, and regions with q-values below a user-defined threshold are considered significant. The putative copy number level is obtained by applying both low- and high-level thresholds to the gene copy levels of all the samples. The entries with value +/- 2 exceed the high-level thresholds for amplifications/deletions, and those with +/- 1 exceed the low-level thresholds but not the high-level thresholds.

Multivariate survival analysis

A Cox proportional hazard model was used for multivariate analysis, and hazard ratio (HR) was calculated according to the cut-off value of a 95% confidence interval (CI). For 468 TCGA breast cancer samples, factors included in the multivariate analysis model were age at diagnosis, ER status (positive vs. negative), PR status (positive vs. negative), HER2 status (positive vs.

negative), tumor size (>20 mm vs. ≤20mm), lymph node status (positive vs. negative), metastasis status (positive vs. negative), and PAM50 subtype (basal vs. non-basal). To investigate DNA copy number associated with survival, samples were segregated into the following three groups for each HMT: amp/gain (high-level amplification and low-level gain), diploid, or deletion (heterozygous and homozygous deletion). Of the altered samples, fewer than 30 were excluded from this analysis in order to prevent skewed data resulting from small sample sizes. To analyze the relationships between HMT mRNA expression and overall patient survival in breast cancer, samples were divided into low (n=385) and high (n=385) groups based on mRNA expression Z-scores [RNA-Seq V2 RSEM (RNA-Seq by Expectation-Maximization)] of each HMT. Multivariate survival analysis was conducted using the Cox regression function ("coxph") in the R statistical programming language.

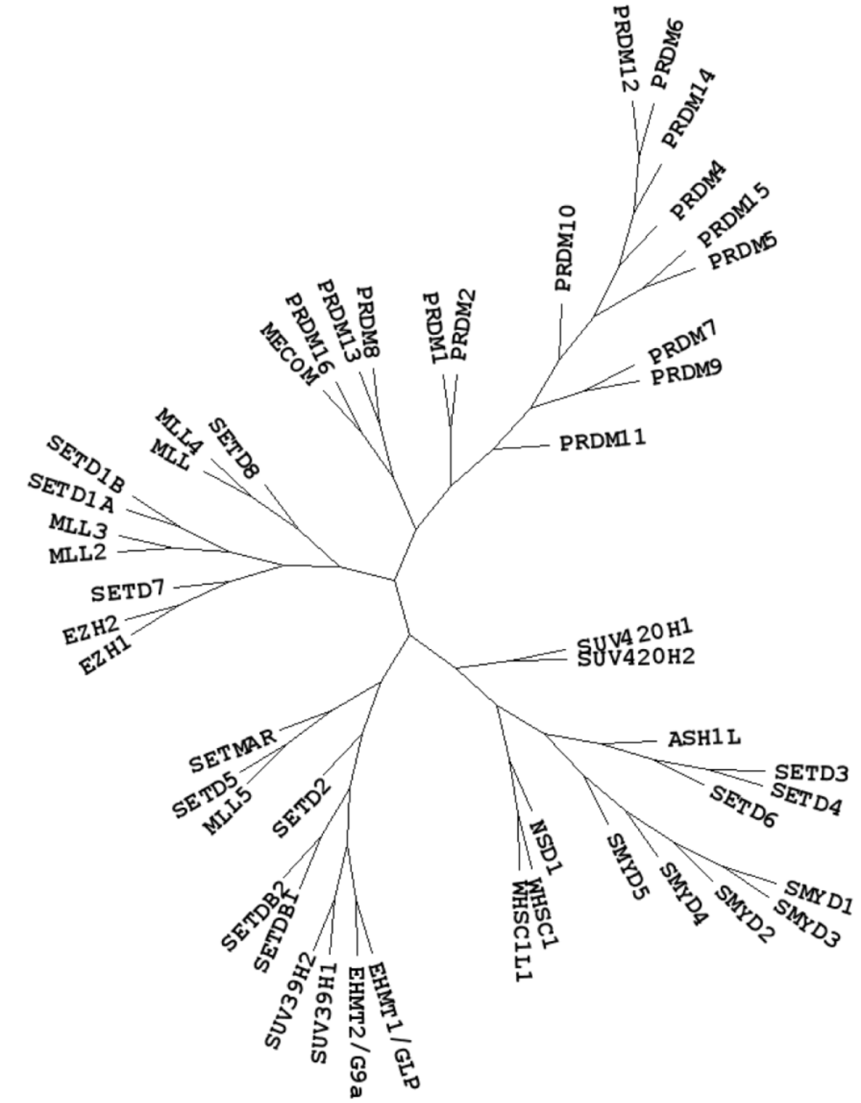

Figure S1: Phylogenetic analysis of histone lysine methyltransferases. The image was obtained from the ChromoHub database (http://www.thesgc.org). The phylogeny outlined in the tree is derived from a CLUSTALW multiple sequence alignment of the full-length sequence of the default Swiss-Prot variant. One HMT, DOT1L, which does not contain a SET domain, was not shown.

Cell Lines	SETDB1 (1q21.3)	ASH1L (1q22)	SMYD2 (1q32.3)	SMYD3 (1q44)	SETD1A (16p11.2)	WHSC1L1 (8p11.23)	SUV420H1 (11q13.2)	SETDB2 (13q14.2)
Colo824	2	1	1	1	0	0	0	-1
SUM229	1	1	0	0	1	-2	-1	-2
SUM149	0	-1	0	0	0	-1	1	-1
HCC70	1	1	1	1	1	-1	0	-1
HCC1937	-1	-1	1	-1	1	-1	1	-1
HCC1187	2	1	1	1	0	1	1	1
MDB-MA-468	1	1	1	1	-1	-1	1	-1
HCC1954	1	1	1	1	-1	-2	-1	-1
SUM190	1	1	0	0	1	0	-1	-2
SUM225	1	0	0	0	1	0	0	-1
HCC1428	1	1	-1	1	1	1	1	-1
SUM44	0	0	1	1	0	2	2	-1
SUM52	0	0	1	0	1	1	1	-1
T47D	1	1	1	0	0	1	1	-1
SUM185	0	0	1	0	1	0	0	0
ZR-75-1	1	1	2	1	1	2	2	0
MCF7	1	-1	-1	0	0	-1	1	-1

Figure S2: CNAs of eight HMTs in 17 breast cancer cell lines. DNA copy number alterations (CNAs) for eight selected HMTs in breast cancer cell lines from comparative genomic hybridization analysis and the TCGA database. Basal-like cell lines are highlighted in red, HER2+ in green, and Luminal in blue. Values represent homozygous deletion (-2), heterozygous loss (-1), diploid (0), low-level gain (+1), and high-level amplification (+2). Deletion/loss is colored in green, and gain/amplification is in red.

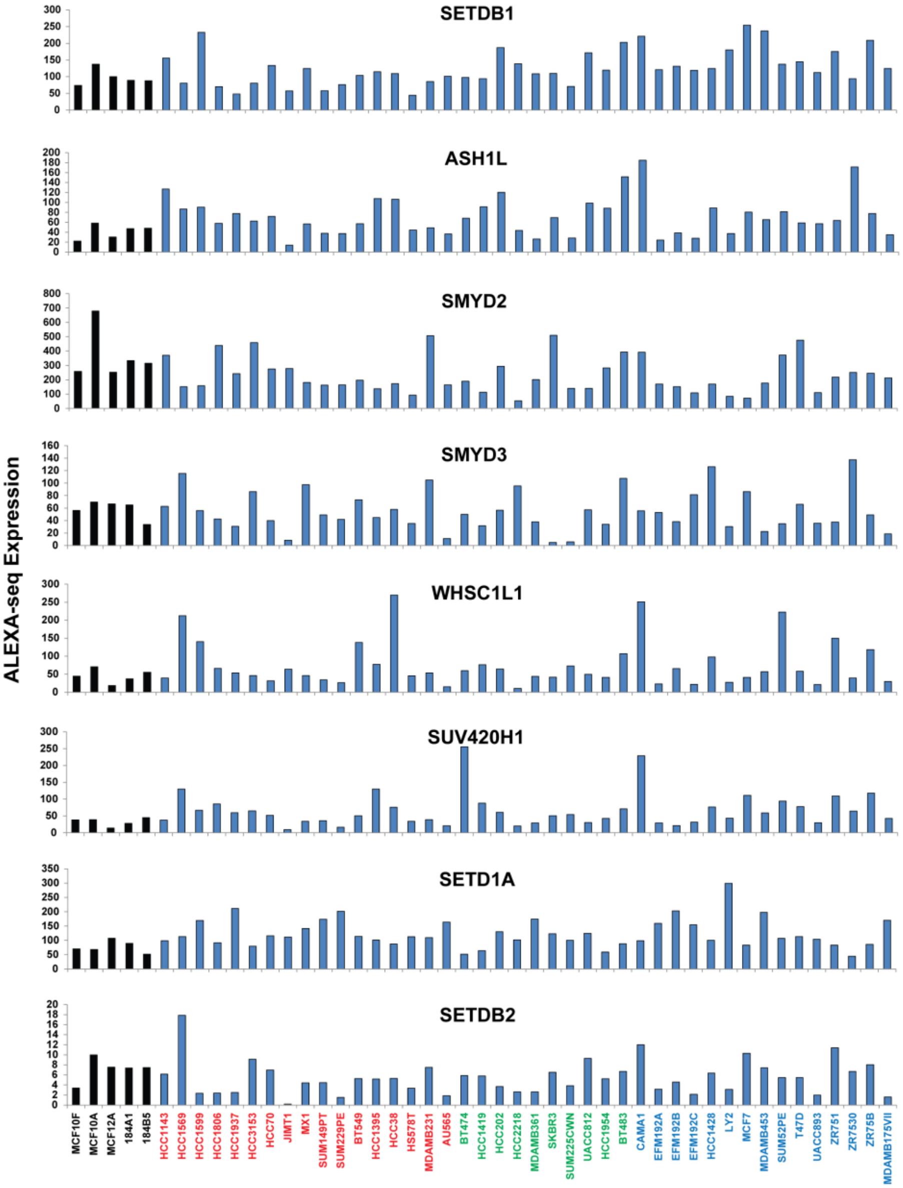

Figure S3: mRNA expression levels from RNA-Seq (GSE48216) of eight HMTs in a panel of 42 breast cancer cell lines compared with 5 normal mammary epithelial cell lines. Cell line names: black indicates normal mammary epithelial cell lines; red, basal-like breast cancer cell lines; green, HER2+ breast cancer cell lines; and blue, Luminal breast cancer cell lines.

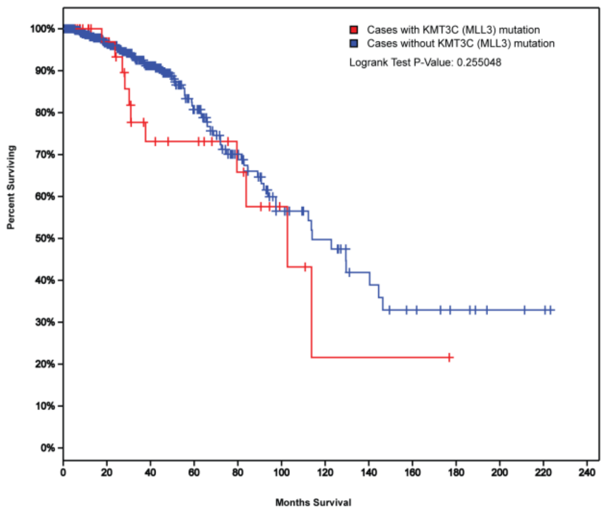

Figure S4: Overall survival associated with KMT2C mutation in breast cancer (Log-rank test p-value = 0.26).

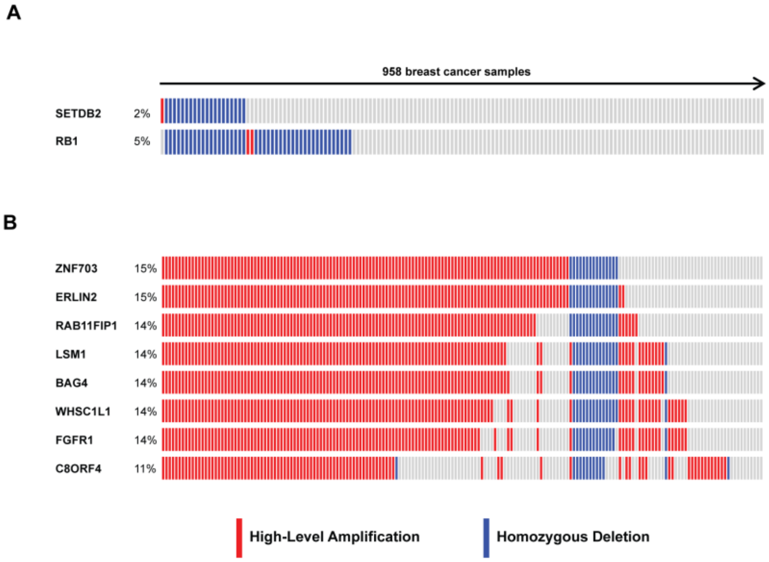

Figure S5: (A) Homozygous deletion of SETDB2 and RB1 at 13q14, and (B) high-level amplification of WHSC1L1 and other 8p11-12 genes (ZNF703, ERLIN2, RAB11FIP1, LSM1, BAG4, FGFR1, and C8ORF4) in the TCGA breast cancer dataset (n=958). Data are displayed using the Oncoprint tool from cBioPortal.

References

1. Mermel CH, Schumacher SE, Hill B, Meyerson ML, Beroukhim R and Getz G. GISTIC2.0 facilitates sensitive and confident localization of the targets of focal somatic copy-number alteration in human cancers. Genome Biol. 2011; 12(4):R41.

2. Beroukhim R, Getz G, Nghiemphu L, Barretina J, Hsueh T, Linhart D, Vivanco I, Lee JC, Huang JH, Alexander S, Du J, Kau T, Thomas RK, Shah K, Soto H, Perner S, et al. Assessing the significance of chromosomal aberrations in cancer: methodology and application to glioma. Proc Natl Acad Sci U S A. 2007; 104(50):20007-20012.
