# Supplementary material for: Genetic alterations of histone lysine methyltransferases and their significance in breast cancer
Source: Oncotarget. 2014 Dec 11;6(4):2466–82. doi: 10.18632/oncotarget.2967 (PMC4385864; doi:10.18632/oncotarget.2967)
Supplement: Supplementary file 2 [file oncotarget-06-2466-s002.pdf]

Table S1. Genetic alterations of HMTs in Luminal A, Luminal B, HER2+ and Basal-like subtypes of breast cancer

Luminal-A	Homdel	Hetloss	Diploid	Gain	Amp
ASH1L	0.00%	1.36%	31.82%	57.27%	9.55%
DOT1L	0.00%	16.36%	71.36%	12.27%	0.00%
EHMT1	0.00%	16.82%	70.91%	12.27%	0.00%
EHMT2	0.00%	14.09%	72.73%	12.27%	0.91%
EZH1	0.00%	20.00%	61.82%	16.82%	1.36%
EZH2	0.00%	13.18%	64.09%	21.82%	0.91%
KMT2A	1.36%	43.18%	48.64%	6.82%	0.00%
KMT2C	0.45%	14.09%	62.73%	22.27%	0.45%
KMT2D	0.00%	5.00%	76.36%	18.18%	0.45%
KMT2E	0.00%	12.27%	65.45%	21.82%	0.45%
MECOM	0.00%	4.55%	73.64%	19.09%	2.73%
NSD1	0.00%	3.64%	71.36%	23.18%	1.82%
PRDM1	0.00%	30.00%	63.18%	5.91%	0.91%
PRDM10	0.91%	39.55%	51.82%	7.27%	0.45%
PRDM11	0.00%	14.55%	70.91%	14.09%	0.45%
PRDM12	0.00%	17.73%	71.82%	10.45%	0.00%
PRDM13	0.00%	31.36%	62.73%	5.45%	0.45%
PRDM14	0.00%	4.09%	48.18%	41.36%	6.36%
PRDM15	0.00%	11.36%	75.91%	12.27%	0.45%
PRDM16	0.45%	30.45%	67.73%	1.36%	0.00%
PRDM2	0.00%	32.27%	67.27%	0.45%	0.00%
PRDM4	0.45%	9.09%	75.00%	15.45%	0.00%
PRDM5	0.00%	17.27%	76.36%	5.91%	0.45%
PRDM6	0.45%	5.91%	70.45%	22.27%	0.91%
PRDM7	2.27%	76.36%	15.45%	5.91%	0.00%
PRDM8	0.00%	17.27%	76.82%	5.91%	0.00%
PRDM9	0.00%	5.00%	66.82%	26.36%	1.82%
SETD1A	0.00%	3.64%	32.27%	56.82%	7.27%
SETD1B	0.91%	10.91%	73.18%	15.00%	0.00%
SETD2	0.45%	17.73%	68.64%	13.18%	0.00%
SETD3	0.00%	15.91%	72.27%	11.36%	0.45%
SETD4	0.00%	12.27%	76.82%	10.91%	0.00%
SETD5	0.00%	6.82%	75.91%	16.82%	0.45%
SETD6	1.82%	72.73%	18.64%	6.82%	0.00%
SETD7	0.00%	18.18%	75.45%	5.91%	0.45%
SETD8	0.45%	10.91%	72.27%	16.36%	0.00%
SETDB1	0.00%	0.91%	33.18%	56.36%	9.55%
SETDB2	0.91%	35.00%	55.00%	9.09%	0.00%
SETMAR	0.00%	7.27%	75.45%	16.82%	0.45%
SMYD1	0.00%	17.27%	78.64%	4.09%	0.00%
SMYD2	0.00%	0.91%	28.64%	58.64%	11.82%
SMYD3	0.45%	2.27%	29.09%	56.82%	11.36%
SMYD4	0.00%	49.09%	45.45%	5.00%	0.45%
SMYD5	0.00%	16.82%	79.09%	4.09%	0.00%
SUV39H1	0.00%	11.36%	74.55%	12.73%	1.36%
SUV39H2	0.45%	12.27%	72.73%	14.09%	0.45%
SUV420H1	0.00%	12.27%	62.73%	20.91%	4.09%
SUV420H2	0.00%	6.82%	73.64%	16.82%	2.73%
WHSC1	0.45%	19.55%	72.73%	6.36%	0.91%
WHSC1L1	1.36%	17.27%	42.27%	27.73%	11.36%

Luminal-B	Homdel	Hetloss	Diploid	Gain	Amp
ASH1L	0.00%	3.31%	15.70%	66.12%	14.88%
DOT1L	0.00%	48.76%	41.32%	9.09%	0.83%
EHMT1	0.00%	35.54%	44.63%	19.01%	0.83%
EHMT2	0.00%	18.18%	52.89%	28.93%	0.00%
EZH1	0.00%	33.06%	33.06%	33.88%	0.00%
EZH2	0.00%	23.14%	54.55%	22.31%	0.00%
KMT2A	0.83%	68.60%	22.31%	8.26%	0.00%
KMT2C	1.65%	23.97%	52.89%	21.49%	0.00%
KMT2D	0.00%	5.79%	60.33%	33.06%	0.83%
KMT2E	0.00%	23.14%	55.37%	20.66%	0.83%
MECOM	0.00%	4.13%	58.68%	35.54%	1.65%
NSD1	0.00%	13.22%	47.93%	38.02%	0.83%
PRDM1	1.65%	45.45%	34.71%	13.22%	4.96%
PRDM10	2.48%	63.64%	24.79%	8.26%	0.83%
PRDM11	0.83%	21.49%	52.07%	24.79%	0.83%
PRDM12	0.00%	36.36%	47.93%	15.70%	0.00%
PRDM13	0.83%	47.11%	38.02%	13.22%	0.83%
PRDM14	0.00%	4.13%	28.10%	52.89%	14.88%
PRDM15	0.00%	24.79%	47.93%	26.45%	0.83%
PRDM16	0.00%	52.89%	42.15%	4.96%	0.00%
PRDM2	0.83%	52.89%	41.32%	4.13%	0.83%
PRDM4	0.00%	14.05%	59.50%	25.62%	0.83%
PRDM5	0.00%	32.23%	50.41%	16.53%	0.83%
PRDM6	0.83%	16.53%	53.72%	28.93%	0.00%
PRDM7	5.79%	66.94%	21.49%	5.79%	0.00%
PRDM8	0.83%	31.40%	45.45%	21.49%	0.83%
PRDM9	0.00%	9.09%	47.93%	42.98%	0.00%
SETD1A	0.00%	4.13%	35.54%	57.85%	2.48%
SETD1B	0.00%	16.53%	56.20%	26.45%	0.83%
SETD2	0.83%	32.23%	51.24%	15.70%	0.00%
SETD3	0.00%	36.36%	47.11%	14.88%	1.65%
SETD4	0.83%	21.49%	52.07%	23.97%	1.65%
SETD5	0.83%	14.05%	52.07%	31.40%	1.65%
SETD6	0.00%	66.94%	23.97%	9.09%	0.00%
SETD7	0.00%	36.36%	47.93%	15.70%	0.00%
SETD8	0.00%	14.88%	56.20%	28.10%	0.83%
SETDB1	0.00%	7.44%	16.53%	60.33%	15.70%
SETDB2	2.48%	52.07%	37.19%	8.26%	0.00%
SETMAR	0.00%	15.70%	52.07%	31.40%	0.83%
SMYD1	0.00%	19.01%	66.12%	14.88%	0.00%
SMYD2	0.00%	4.96%	15.70%	66.12%	13.22%
SMYD3	0.83%	4.13%	17.36%	63.64%	14.05%
SMYD4	1.65%	61.16%	29.75%	6.61%	0.83%
SMYD5	0.00%	19.83%	65.29%	14.05%	0.83%
SUV39H1	0.00%	22.31%	52.07%	25.62%	0.00%
SUV39H2	0.00%	16.53%	59.50%	23.97%	0.00%
SUV420H1	0.00%	15.70%	40.50%	32.23%	11.57%
SUV420H2	0.00%	20.66%	47.11%	29.75%	2.48%
WHSC1	0.00%	30.58%	49.59%	19.01%	0.83%
WHSC1L1	0.83%	23.14%	27.27%	29.75%	19.01%

HER2	Homdel	Hetloss	Diploid	Gain	Amp
ASH1L	0.00%	5.45%	21.82%	58.18%	14.55%
DOT1L	1.82%	40.00%	45.45%	12.73%	0.00%
EHMT1	0.00%	23.64%	52.73%	20.00%	3.64%
EHMT2	0.00%	14.55%	50.91%	34.55%	0.00%
EZH1	3.64%	80.00%	7.27%	5.45%	3.64%
EZH2	0.00%	38.18%	40.00%	21.82%	0.00%
KMT2A	1.82%	54.55%	32.73%	10.91%	0.00%
KMT2C	0.00%	41.82%	38.18%	20.00%	0.00%
KMT2D	0.00%	12.73%	54.55%	30.91%	1.82%
KMT2E	0.00%	36.36%	43.64%	20.00%	0.00%
MECOM	0.00%	12.73%	41.82%	36.36%	9.09%
NSD1	0.00%	25.45%	50.91%	23.64%	0.00%
PRDM1	1.82%	25.45%	41.82%	16.36%	14.55%
PRDM10	3.64%	45.45%	36.36%	14.55%	0.00%
PRDM11	0.00%	27.27%	50.91%	18.18%	3.64%
PRDM12	1.82%	21.82%	43.64%	27.27%	5.45%
PRDM13	1.82%	34.55%	43.64%	18.18%	1.82%
PRDM14	1.82%	10.91%	27.27%	43.64%	16.36%
PRDM15	0.00%	20.00%	34.55%	40.00%	5.45%
PRDM16	1.82%	50.91%	34.55%	10.91%	1.82%
PRDM2	1.82%	47.27%	36.36%	12.73%	1.82%
PRDM4	0.00%	27.27%	45.45%	27.27%	0.00%
PRDM5	0.00%	47.27%	45.45%	7.27%	0.00%
PRDM6	0.00%	32.73%	52.73%	14.55%	0.00%
PRDM7	3.64%	61.82%	23.64%	9.09%	1.82%
PRDM8	0.00%	40.00%	43.64%	14.55%	1.82%
PRDM9	1.82%	10.91%	43.64%	41.82%	1.82%
SETD1A	0.00%	7.27%	34.55%	52.73%	5.45%
SETD1B	0.00%	23.64%	47.27%	29.09%	0.00%
SETD2	0.00%	49.09%	47.27%	3.64%	0.00%
SETD3	0.00%	21.82%	43.64%	32.73%	1.82%
SETD4	1.82%	18.18%	36.36%	41.82%	1.82%
SETD5	0.00%	23.64%	50.91%	20.00%	5.45%
SETD6	0.00%	56.36%	29.09%	12.73%	1.82%
SETD7	1.82%	45.45%	47.27%	3.64%	1.82%
SETD8	0.00%	25.45%	47.27%	27.27%	0.00%
SETDB1	0.00%	5.45%	25.45%	49.09%	20.00%
SETDB2	1.82%	52.73%	32.73%	10.91%	1.82%
SETMAR	0.00%	23.64%	45.45%	20.00%	10.91%
SMYD1	0.00%	16.36%	47.27%	36.36%	0.00%
SMYD2	0.00%	7.27%	20.00%	58.18%	14.55%
SMYD3	0.00%	1.82%	20.00%	63.64%	14.55%
SMYD4	0.00%	89.09%	7.27%	3.64%	0.00%
SMYD5	0.00%	16.36%	49.09%	34.55%	0.00%
SUV39H1	0.00%	18.18%	50.91%	30.91%	0.00%
SUV39H2	0.00%	14.55%	45.45%	40.00%	0.00%
SUV420H1	0.00%	21.82%	38.18%	29.09%	10.91%
SUV420H2	0.00%	20.00%	49.09%	27.27%	3.64%
WHSC1	1.82%	49.09%	40.00%	9.09%	0.00%
WHSC1L1	3.64%	38.18%	38.18%	10.91%	9.09%

Basal	Homdel	Hetloss	Diploid	Gain	Amp
ASH1L	0.00%	1.14%	6.82%	64.77%	27.27%
DOT1L	3.41%	56.82%	28.41%	11.36%	0.00%
EHMT1	2.27%	54.55%	29.55%	12.50%	1.14%
EHMT2	0.00%	10.23%	43.18%	42.05%	4.55%
EZH1	1.14%	51.14%	35.23%	7.95%	4.55%
EZH2	2.27%	12.50%	43.18%	39.77%	2.27%
KMT2A	1.14%	36.36%	40.91%	21.59%	0.00%
KMT2C	4.55%	14.77%	37.50%	40.91%	2.27%
KMT2D	0.00%	55.68%	35.23%	9.09%	0.00%
KMT2E	0.00%	9.09%	38.64%	51.14%	1.14%
MECOM	0.00%	5.68%	32.95%	50.00%	11.36%
NSD1	3.41%	53.41%	28.41%	13.64%	1.14%
PRDM1	0.00%	20.45%	39.77%	35.23%	4.55%
PRDM10	1.14%	45.45%	37.50%	15.91%	0.00%
PRDM11	0.00%	20.45%	54.55%	21.59%	3.41%
PRDM12	0.00%	55.68%	30.68%	13.64%	0.00%
PRDM13	0.00%	21.59%	42.05%	32.95%	3.41%
PRDM14	0.00%	4.55%	21.59%	61.36%	12.50%
PRDM15	1.14%	12.50%	47.73%	35.23%	3.41%
PRDM16	2.27%	31.82%	36.36%	23.86%	5.68%
PRDM2	0.00%	47.73%	38.64%	13.64%	0.00%
PRDM4	0.00%	44.32%	47.73%	7.95%	0.00%
PRDM5	0.00%	44.32%	43.18%	11.36%	1.14%
PRDM6	2.27%	69.32%	25.00%	3.41%	0.00%
PRDM7	1.14%	25.00%	43.18%	25.00%	5.68%
PRDM8	0.00%	40.91%	43.18%	13.64%	2.27%
PRDM9	1.14%	29.55%	30.68%	29.55%	9.09%
SETD1A	0.00%	23.86%	42.05%	32.95%	1.14%
SETD1B	0.00%	44.32%	46.59%	9.09%	0.00%
SETD2	0.00%	53.41%	39.77%	6.82%	0.00%
SETD3	2.27%	55.68%	29.55%	11.36%	1.14%
SETD4	0.00%	12.50%	47.73%	38.64%	1.14%
SETD5	0.00%	26.14%	48.86%	22.73%	2.27%
SETD6	0.00%	34.09%	45.45%	19.32%	1.14%
SETD7	0.00%	44.32%	44.32%	10.23%	1.14%
SETD8	1.14%	40.91%	47.73%	9.09%	1.14%
SETDB1	0.00%	0.00%	7.95%	59.09%	32.95%
SETDB2	6.82%	55.68%	30.68%	6.82%	0.00%
SETMAR	2.27%	23.86%	48.86%	22.73%	2.27%
SMYD1	1.14%	7.95%	46.59%	43.18%	1.14%
SMYD2	1.14%	5.68%	14.77%	70.45%	7.95%
SMYD3	0.00%	3.41%	13.64%	55.68%	27.27%
SMYD4	0.00%	61.36%	28.41%	9.09%	1.14%
SMYD5	1.14%	2.27%	48.86%	46.59%	1.14%
SUV39H1	2.27%	21.59%	47.73%	27.27%	1.14%
SUV39H2	0.00%	3.41%	30.68%	47.73%	18.18%
SUV420H1	0.00%	18.18%	53.41%	25.00%	3.41%
SUV420H2	0.00%	18.18%	42.05%	35.23%	4.55%
WHSC1	2.27%	57.95%	32.95%	6.82%	0.00%
WHSC1L1	0.00%	34.09%	26.14%	29.55%	10.23%
