# Supplementary material for: Genetic alterations of histone lysine methyltransferases and their significance in breast cancer
Source: Oncotarget. 2014 Dec 11;6(4):2466–82. doi: 10.18632/oncotarget.2967 (PMC4385864; doi:10.18632/oncotarget.2967)
Supplement: Supplementary file 3 [file oncotarget-06-2466-s003.pdf]

Table S2. Genetic mutations of HMTs in Luminal A, Luminal B, HER2+ and Basal-like subtypes of breast cancer

Gene	Luminal A (%)	Luminal B (%)	HER2 (%)	Basal (%)
ASH1L	0.431	4.651	1.724	1.020
DOT1L	0.000	3.101	1.724	1.020
EHMT1	0.000	1.550	1.724	2.041
EHMT2	0.431	0.000	0.000	0.000
EZH1	0.431	0.775	0.000	0.000
EZH2	0.000	0.775	0.000	1.020
KMT2A	2.586	0.775	1.724	1.020
KMT2B	0.431	4.651	1.724	3.061
KMT2C	8.621	6.202	5.172	6.122
KMT2D	1.293	3.876	5.172	3.061
KMT2E	0.431	0.000	1.724	2.041
MECOM	0.862	0.000	0.000	1.020
NSD1	0.862	0.775	0.000	0.000
PRDM1	0.431	1.550	1.724	1.020
PRDM10	0.000	0.775	3.448	1.020
PRDM11	0.431	0.000	0.000	0.000
PRDM12	0.431	0.000	0.000	0.000
PRDM13	0.431	0.000	0.000	0.000
PRDM14	0.000	0.000	0.000	1.020
PRDM15	0.862	0.775	0.000	2.041
PRDM16	1.293	0.000	0.000	0.000
PRDM2	0.431	1.550	0.000	0.000
PRDM4	0.431	0.775	0.000	1.020
PRDM5	0.000	0.000	0.000	1.020
PRDM6	0.000	0.000	0.000	0.000
PRDM7	0.000	0.000	0.000	1.020
PRDM8	0.431	0.000	1.724	1.020
PRDM9	0.431	0.000	0.000	0.000
SETD1A	0.431	0.000	0.000	0.000
SETD1B	0.000	1.550	0.000	3.061
SETD2	1.293	0.000	5.172	0.000
SETD3	0.431	0.000	1.724	0.000
SETD4	0.000	0.000	0.000	0.000
SETD5	0.862	0.775	0.000	0.000
SETD6	0.000	0.000	0.000	0.000
SETD7	0.431	0.000	1.724	1.020
SETD8	0.000	1.550	0.000	0.000
SETDB1	0.431	2.326	5.172	1.020
SETDB2	0.000	0.000	0.000	0.000
SETMAR	0.000	1.550	0.000	0.000
SMYD1	0.000	1.550	0.000	1.020
SMYD2	0.431	0.000	0.000	0.000
SMYD3	0.431	0.775	0.000	1.020
SMYD4	0.431	1.550	0.000	0.000
SMYD5	0.431	0.000	1.724	0.000
SUV39H1	0.000	0.000	0.000	0.000
SUV39H2	0.431	0.000	0.000	0.000
SUV420H1	0.431	0.000	1.724	2.041
SUV420H2	0.000	0.000	0.000	1.020
WHSC1	0.000	2.326	0.000	2.041
WHSC1L1	0.000	0.775	0.000	0.000
