# Supplementary material for: Genetic alterations of histone lysine methyltransferases and their significance in breast cancer
Source: Oncotarget. 2014 Dec 11;6(4):2466–82. doi: 10.18632/oncotarget.2967 (PMC4385864; doi:10.18632/oncotarget.2967)
Supplement: Supplementary file 4 [file oncotarget-06-2466-s004.pdf]

Table S3. Predicting the functional impact of KMT2C and KMT2D missense mutations with Mutation Assessor

KMT2C

Sample ID	AA change	Type	Mutation Assessor Score
TCGA-A1-A0SI	W430C	Missense	High
TCGA-A7-A5ZW	H252Y	Missense	High
TCGA-A8-A07E	H414Y	Missense	High
TCGA-AC-A23H	D4344H	Missense	Medium
TCGA-AR-A0U0	C977Y	Missense	Medium
TCGA-B6-A0RP	D4425Y	Missense	Medium
TCGA-BH-A0BW	G3106D	Missense	Medium
TCGA-BH-A1ES	G3584R	Missense	Medium
TCGA-C8-A3M8	C359S	Missense	Medium
TCGA-E9-A1N8	H367D	Missense	Medium
TCGA-A1-A0SF	L1793F	Missense	Low
TCGA-A8-A0A6	V1776G	Missense	Low
TCGA-A8-A0A6	T1837P	Missense	Low
TCGA-AC-A23H	E3724K	Missense	Low
TCGA-AC-A23H	D3264N	Missense	Low
TCGA-AN-A046	E2885K	Missense	Low
TCGA-B6-A0IJ	C134Y	Missense	Low
TCGA-B6-A408	A3148S	Missense	Low
TCGA-BH-A1EY	S3394N	Missense	Low
TCGA-EW-A1J5	E97Q	Missense	Low
TCGA-A2-A0SV	I1381V	Missense	Neutral
TCGA-A8-A07R	V3524A	Missense	Neutral
TCGA-A8-A0A6	S914R	Missense	Neutral
TCGA-A8-A0A6	T3303P	Missense	Neutral
TCGA-AR-A0TX	G722E	Missense	Neutral
TCGA-BH-A18P	E1992Q	Missense	Neutral

KMT2D

Sample ID	AA change	Type	Mutation Assessor Score
TCGA-A8-A0A6	V5435G	Missense	Medium
TCGA-A8-A076	R3582W	Missense	Low
TCGA-BH-A0B9	D1954H	Missense	Low
TCGA-AN-A046	K4470N	Missense	Low
TCGA-A8-A07R	H5176N	Missense	Low
TCGA-C8-A1HK	D1828H	Missense	Low
TCGA-BH-A18J	K3235N	Missense	Low
TCGA-AN-A0AK	R3547H	Missense	Low
TCGA-A7-A26E	R1586L	Missense	Low
TCGA-A2-A0CT	E3587V	Missense	Low
TCGA-C8-A26Y	D1438N	Missense	Low
TCGA-D8-A13Z	M5332I	Missense	Neutral
TCGA-C8-A1HM	G3844V	Missense	Neutral
TCGA-AN-A04C	R3626W	Missense	Neutral
TCGA-BH-A18G	N334I	Missense	Neutral
TCGA-D8-A1XQ	S4871R	Missense	Neutral
TCGA-C8-A26X	T3765R	Missense	Neutral
TCGA-BH-A203	Q3905L	Missense	Neutral
TCGA-GM-A2D9	D1848N	Missense	Neutral
