# Supplementary material for: Genetic alterations of histone lysine methyltransferases and their significance in breast cancer
Source: Oncotarget. 2014 Dec 11;6(4):2466–82. doi: 10.18632/oncotarget.2967 (PMC4385864; doi:10.18632/oncotarget.2967)
Supplement: Supplementary file 5 [file oncotarget-06-2466-s005.pdf]

Table S4. Array CGH data of Colo824, HCC1187 and MCF7 breast cancer cell lines on chromosome 1q using Agilent 244K chip

ProbeName	Start	Stop	Gene	Colo824	HCC1187	MCF7
A_16_P35292719	142116690	142116734	AK130052	1.53	0.90	0.83
A_16_P15281639	142401315	142401374	NBPF1	1.55	1.70	0.63
A_16_P00159259	142409033	142409092	NBPF1	1.42	1.65	0.85
A_14_P125891	142416822	142416881	NBPF1	1.04	1.46	0.77
A_16_P00159281	142423627	142423686	NBPF1	1.23	1.73	0.80
A_16_P00159305	142435868	142435927	NBPF1	1.67	2.21	0.91
A_14_P115693	142444242	142444301	NBPF1	0.45	2.88	1.07
A_16_P00159342	142457879	142457938	NBPF1	1.47	2.02	0.80
A_16_P00159346	142465077	142465135	NBPF1	1.29	1.07	0.73
A_16_P35293442	142475410	142475469	NBPF1	1.45	2.01	1.09
A_14_P131082	142477759	142477818	NBPF1	1.73	2.17	0.74
A_14_P103187	142483991	142484039	NBPF1	1.89	1.98	0.75
A_14_P135298	142484269	142484321	NBPF1	1.27	1.52	0.70
A_14_P139988	142498565	142498624	NBPF1	0.98	1.43	0.92
A_16_P15281911	142504864	142504923	NBPF1	0.73	2.05	0.55
A_16_P15281947	142516496	142516555	NBPF1	0.79	1.62	0.78
A_16_P00159448	142532026	142532085	NBPF1	1.81	2.30	0.93
A_16_P15281992	142536734	142536793	NBPF1	1.38	1.83	0.75
A_16_P15282024	142553595	142553654	NBPF1	1.31	1.66	0.83
A_14_P104604	142565191	142565250	NBPF1	1.99	1.82	1.00
A_16_P35293677	142570866	142570925	NBPF1	1.92	1.61	0.94
A_16_P15282068	142580087	142580146	NBPF1	1.29	1.91	1.01
A_14_P105608	142592920	142592976	NBPF1	1.61	1.50	0.75
A_16_P15282115	142598507	142598566	NBPF1	0.59	1.12	0.97
A_16_P00159544	142610589	142610648	NBPF1	1.77	1.79	1.04
A_16_P00159546	142628370	142628418	NBPF1	1.83	1.39	0.79
A_16_P15282636	142780742	142780799	NBPF1	1.73	1.71	0.90
A_16_P00159573	142871406	142871455	AF379624	1.48	1.33	0.30
A_16_P15282937	142877399	142877458	AF379624	1.73	2.20	1.12
A_16_P15282960	142890482	142890541	AF379624	2.04	2.46	1.21
A_16_P35294639	142898229	142898288	AF379624	1.84	1.60	1.25
A_14_P131300	142902432	142902491	HFE2	1.86	1.32	1.15
A_14_P108657	142905389	142905440	HFE2	1.84	1.45	0.88
A_16_P35294674	142910791	142910850	AF379624	2.31	2.42	1.34
A_16_P15283001	142914439	142914498	AF379624	2.30	2.06	1.26
A_16_P00159620	142923694	142923753	AF379624	2.13	1.89	1.18
A_14_P137169	142929291	142929350	TXNIP	2.14	2.03	1.33
A_16_P35294756	142940373	142940432	AF379624	2.27	2.59	0.94
A_14_P112233	142945720	142945779	POLR3GL	1.79	1.23	1.07
A_16_P00159675	142955707	142955766	POLR3GL	2.41	2.12	1.33
A_16_P35294813	142962986	142963032	ANKRD34	2.33	1.19	0.96
A_14_P122491	142966673	142966732	LIX1L	1.92	2.07	0.97
A_16_P15283155	142974897	142974956	LIX1L	1.70	1.80	0.97
A_16_P00159714	142981327	142981386	LIX1L	2.00	2.44	1.26
A_16_P15283187	142988096	142988155	LIX1L	1.88	2.12	1.17

A_16_P15283195	142993625	142993684	AF379624	1.76	2.59	1.09
A_14_P116802	142997202	142997252	RBM8A	2.13	2.45	1.07
A_16_P35294920	143001341	143001400	AF379624	2.21	1.27	1.21
A_14_P139097	143004427	143004477	AF379624	2.12	2.03	0.93
A_14_P102523	143010599	143010658	PEX11B	2.07	1.95	1.25
A_16_P35294963	143014215	143014274	ITGA10	1.76	2.31	1.25
A_16_P00159764	143018866	143018925	ITGA10	2.10	2.50	1.28
A_14_P111825	143026501	143026557	ITGA10	2.63	2.26	1.19
A_16_P15283310	143032896	143032955	AF379624	2.22	2.13	1.22
A_16_P15283328	143039782	143039841	ANKRD35	2.01	2.18	0.97
A_14_P123955	143045677	143045726	ANKRD35	2.30	1.64	0.98
A_16_P15283358	143049887	143049946	ANKRD35	1.89	2.55	0.92
A_14_P100205	143057538	143057597	AF379624	1.24	1.47	0.39
A_16_P00159850	143062829	143062888	AF379624	1.91	1.88	0.98
A_14_P134227	143066267	143066324	PIAS3	2.32	2.38	1.19
A_16_P15283418	143069715	143069773	PIAS3	2.30	2.05	1.08
A_14_P129358	143072927	143072982	PIAS3	2.26	1.29	1.08
A_16_P00159877	143076162	143076219	NUDT17	2.45	1.49	1.27
A_14_P122423	143085939	143085998	POLR3C	2.40	2.63	1.13
A_16_P35295244	143090498	143090557	POLR3C	2.64	1.80	1.30
A_14_P122217	143098216	143098271	POLR3C	2.32	1.92	1.17
A_16_P35295271	143099984	143100028	AF379624	1.81	1.98	1.12
A_16_P15283520	143115281	143115340	ZNF364	1.87	1.97	1.06
A_14_P133719	143121378	143121437	ZNF364	1.99	1.75	1.00
A_16_P35295334	143131388	143131447	ZNF364	1.60	1.85	0.83
A_16_P15283568	143139200	143139259	ZNF364	2.31	2.34	1.28
A_16_P15283584	143145036	143145095	ZNF364	1.74	1.55	0.91
A_14_P127504	143152233	143152292	ZNF364	1.95	1.82	1.15
A_16_P15283605	143161440	143161499	ZNF364	1.68	2.00	1.06
A_16_P00159966	143171054	143171113	ZNF364	1.52	1.43	0.99
A_16_P00159977	143176008	143176067	ZNF364	2.30	2.62	1.34
A_14_P102237	143185545	143185604	CD160	1.79	1.86	1.11
A_16_P00160006	143193151	143193208	CD160	1.61	1.99	0.94
A_14_P105842	143204646	143204699	AF379624	1.91	1.87	1.23
A_16_P35295539	143218369	143218428	PDZK1	2.41	1.93	1.20
A_14_P118046	143229642	143229701	PDZK1	2.54	2.77	1.28
A_14_P200637	143236258	143236313	PDZK1	2.54	1.34	1.37
A_16_P00160072	143288587	143288646	AF379624	1.77	2.29	0.99
A_14_P138716	143456705	143456764	AF379624	1.35	1.48	0.92
A_16_P35296016	143480571	143480630	AF379624	1.46	1.57	1.17
A_16_P15284207	143488396	143488446	AF379624	1.59	2.51	0.91
A_16_P35296161	143522963	143523020	NBPF11	0.87	1.76	0.60
A_16_P35296175	143526765	143526824	NBPF11	1.76	0.98	0.85
A_16_P00160107	143543580	143543626	NBPF11	0.89	1.85	0.79
A_16_P00160110	143551076	143551133	NBPF11	1.66	1.61	0.50
A_16_P35296280	143565999	143566058	AF379624	1.40	2.54	0.54
A_16_P35296300	143575420	143575479	AF379624	0.74	2.27	0.98
A_16_P00160145	143586636	143586695	AF379624	1.47	1.31	0.67

A_16_P00160158	143603866	143603925	AF379624	1.24	1.64	0.97
A_16_P35296380	143612738	143612797	AF379624	0.78	1.70	0.70
A_16_P15284612	143699032	143699091	AF419616	0.67	1.92	0.56
A_16_P00160179	143725788	143725847	AF419616	1.38	1.31	0.97
A_16_P00160203	143743144	143743203	AF419616	1.15	2.25	0.76
A_16_P35296638	143755639	143755693	AF419616	0.65	1.22	0.60
A_16_P00160215	143767659	143767718	AF419616	1.09	1.48	1.05
A_16_P15284786	143778321	143778380	AF419616	1.42	1.54	0.94
A_14_P133709	143789559	143789618	AF419616	1.25	2.44	0.61
A_16_P15284812	143796120	143796179	AF419616	1.69	2.05	1.00
A_16_P35296727	143803852	143803911	AF419616	1.77	2.54	1.10
A_16_P15284879	143816040	143816099	AF419616	1.63	2.56	1.10
A_16_P15284906	143827533	143827592	AF419616	1.36	1.81	1.09
A_16_P35296804	143834763	143834822	AF419616	1.17	2.04	1.02
A_16_P35296812	143843804	143843863	AF419616	1.54	2.53	1.04
A_16_P35296833	143852936	143852995	PRKAB2	1.55	1.65	0.85
A_14_P134571	143866417	143866476	PRKAB2	1.23	1.88	0.94
A_16_P35296899	143871135	143871194	AF419616	1.29	2.05	0.94
A_16_P35296899	143871135	143871194	AF419616	1.22	2.37	0.95
A_16_P35296899	143871135	143871194	AF419616	1.27	2.26	1.04
A_16_P15285024	143880194	143880253	AF419616	0.95	1.20	0.98
A_16_P15285046	143886533	143886592	FMO5	0.32	1.20	0.75
A_14_P123325	143897646	143897702	FMO5	1.66	1.79	1.09
A_16_P00160403	143912123	143912182	FMO5	1.44	2.35	1.09
A_14_P115847	143921481	143921538	FMO5	1.47	2.28	1.06
A_16_P15285147	143931769	143931828	AF419616	0.95	1.45	0.80
A_16_P00160434	143938711	143938770	AF419616	1.87	2.09	1.12
A_16_P15285183	143952961	143953020	CHD1L	0.48	1.18	0.17
A_14_P101373	143963532	143963591	CHD1L	1.58	1.21	0.84
A_14_P138325	143976658	143976708	CHD1L	1.63	2.14	1.11
A_16_P15285277	143982659	143982718	CHD1L	1.57	1.79	1.06
A_14_P133288	143991900	143991959	CHD1L	1.44	1.73	0.96
A_14_P201223	143994685	143994744	AF419616	1.17	3.07	1.60
A_16_P35297250	144009411	144009470	AF419616	1.15	2.33	1.13
A_16_P15285328	144016750	144016809	AF419616	1.44	1.66	0.82
A_16_P00160549	144032249	144032308	AF419616	1.51	1.25	1.24
A_16_P15285361	144045962	144046021	AF419616	1.45	1.64	1.44
A_16_P15285383	144060264	144060323	AF419616	0.75	1.89	0.77
A_16_P15285429	144088123	144088182	AF419616	1.13	1.52	0.60
A_16_P35297373	144114301	144114360	AF419616	1.62	2.16	0.87
A_16_P35297407	144130787	144130846	AF419616	1.38	2.20	1.03
A_16_P35297428	144145559	144145618	AF419616	1.36	2.21	0.88
A_16_P35297453	144158228	144158287	AF419616	1.29	2.36	0.72
A_16_P00160661	144169114	144169173	AF419616	1.30	1.96	0.98
A_16_P35297482	144178790	144178849	AF419616	1.13	2.02	0.94
A_16_P35297501	144188282	144188341	AF419616	1.27	2.02	0.97
A_16_P00160704	144198177	144198236	AF419616	0.82	1.54	0.99
A_14_P114645	144206506	144206565	AF419616	1.60	1.61	1.19

A_16_P35297582	144215846	144215905	AF419616	1.41	2.00	0.99
A_16_P00160749	144223467	144223526	AF419616	1.56	2.35	0.97
A_16_P35297633	144231819	144231878	AF419616	1.11	1.66	0.94
A_16_P00160778	144238216	144238275	BCL9	1.71	2.16	1.03
A_16_P15285760	144246678	144246737	BCL9	1.28	1.44	0.40
A_14_P133444	144253340	144253399	BCL9	1.46	1.57	0.89
A_16_P15285812	144265274	144265333	BCL9	0.65	1.33	0.95
A_16_P35297751	144270965	144271024	BCL9	1.90	2.33	0.99
A_16_P00160843	144276561	144276620	BCL9	1.07	2.24	0.98
A_16_P00160855	144282695	144282754	BCL9	0.75	0.56	0.73
A_14_P132205	144293947	144294006	BCL9	1.17	2.00	0.69
A_16_P15285921	144301339	144301398	BCL9	1.84	2.22	1.25
A_14_P102990	144309779	144309837	BCL9	1.85	1.80	1.18
A_16_P00160936	144322086	144322145	BCL9	1.37	1.85	0.94
A_14_P119487	144339075	144339134	AF419616	2.03	2.20	1.45
A_16_P15286043	144344019	144344078	ACP6	1.37	2.17	0.82
A_16_P35298000	144350438	144350497	ACP6	-0.10	1.06	0.86
A_16_P15286085	144356263	144356317	ACP6	2.03	2.00	1.25
A_16_P35298039	144362498	144362557	ACP6	1.56	2.37	0.91
A_14_P131065	144367287	144367338	ACP6	1.61	1.69	0.88
A_16_P35298076	144373958	144374017	AF419616	0.97	1.27	0.85
A_16_P35298082	144387167	144387226	AF419616	1.36	1.84	0.95
A_16_P00161047	144400791	144400850	AF419616	0.91	2.21	0.95
A_16_P00161051	144410795	144410854	AF419616	1.33	2.23	1.01
A_16_P15286210	144424777	144424836	AF419616	0.75	1.91	0.80
A_16_P15286228	144435976	144436035	AF419616	1.79	1.98	1.42
A_16_P15286252	144446717	144446776	AF419616	1.91	2.58	1.16
A_16_P00161107	144454591	144454650	GJA5	1.24	1.61	0.93
A_14_P108057	144461803	144461862	GJA5	0.52	1.99	0.95
A_14_P200776	144467604	144467663	GJA5	1.77	1.98	1.13
A_16_P15286343	144473334	144473393	AF419616	1.56	1.28	0.86
A_16_P15286376	144484286	144484345	AF419616	1.02	1.89	0.86
A_16_P00161197	144498894	144498953	AF419616	1.54	1.77	0.91
A_16_P35298394	144511443	144511502	AF419616	1.56	2.38	0.91
A_16_P15286470	144519773	144519832	AF419616	1.75	2.19	1.18
A_16_P00161236	144533949	144534008	AF419616	1.39	2.29	1.13
A_16_P00161253	144550444	144550503	AF419616	1.53	1.81	0.98
A_16_P15286528	144558372	144558419	AF419616	1.33	1.71	0.68
A_16_P15286531	144565205	144565264	AF419616	1.74	2.46	1.08
A_16_P00161262	144572545	144572602	AF419616	1.47	1.33	0.86
A_16_P15286557	144581391	144581450	AF419616	1.14	2.06	1.05
A_16_P00161277	144586762	144586821	AF419616	1.35	2.00	0.99
A_16_P15286588	144591270	144591328	AF419616	1.69	1.97	1.13
A_16_P00161298	144597431	144597490	AF419616	1.53	1.68	1.00
A_16_P15286625	144603439	144603498	GJA8	1.30	1.10	0.93
A_16_P00161322	144609277	144609336	AF419616	1.78	2.11	1.06
A_16_P00161339	144619019	144619078	AF419616	1.55	1.86	1.18
A_16_P35298661	144636350	144636409	GPR89A	1.48	1.97	1.06

A_16_P15287028	144826666	144826725	AF419616	1.38	2.14	1.20
A_16_P15287051	144836722	144836781	AF419616	1.90	2.76	1.13
A_16_P00161412	144860463	144860522	AF419616	1.15	2.02	0.70
A_16_P15287128	144874654	144874710	AF419616	1.96	2.13	1.15
A_16_P00161441	144901559	144901618	AF419616	1.67	1.94	1.08
A_16_P00161456	144921511	144921568	AF419616	0.80	1.56	0.75
A_16_P35299211	144929672	144929727	AF419616	1.87	2.68	1.02
A_16_P00161468	144939060	144939119	AF419616	1.62	1.68	1.13
A_16_P15287275	144942942	144943001	AF419616	1.67	2.40	0.89
A_16_P00161479	144949052	144949102	AF419616	1.54	2.12	0.89
A_16_P00161485	144958928	144958987	FLJ39739	1.08	2.35	0.82
A_16_P15287315	144966156	144966209	FLJ39739	1.35	2.06	1.03
A_16_P00161499	144975464	144975523	FLJ39739	1.58	1.47	1.00
A_16_P15287404	145012579	145012632	FLJ39739	1.59	0.97	0.82
A_16_P00161510	145023947	145024006	FLJ39739	1.60	1.53	1.11
A_16_P00161534	145647143	145647202	chr1:145647143-145647202	0.59	2.65	0.65
A_16_P15288706	145716245	145716304	chr1:145716245-145716304	0.57	1.86	0.47
A_16_P00161539	145726262	145726321	chr1:145726262-145726321	0.47	1.25	0.10
A_16_P00161545	145820605	145820664	chr1:145820605-145820664	0.78	2.40	-1.11
A_16_P15288885	145842787	145842846	chr1:145842787-145842846	0.35	2.74	-0.73
A_16_P00161579	145870722	145870779	chr1:145870722-145870779	1.80	2.52	0.96
A_16_P00161599	145899886	145899945	chr1:145899886-145899945	-0.12	2.86	-0.02
A_16_P00161620	145934582	145934640	chr1:145934582-145934640	0.73	3.15	-0.67
A_16_P00161640	145951439	145951498	chr1:145951439-145951498	0.34	2.48	-0.57
A_14_P136096	145971637	145971696	chr1:145971637-145971696	1.12	2.26	0.49
A_16_P35301246	145988822	145988881	chr1:145988822-145988881	0.29	2.68	-1.14
A_16_P15289248	146012014	146012073	BC062745	0.32	2.35	0.16
A_16_P00161695	146473741	146473785	X14355	1.30	2.67	0.79
A_16_P15290135	146493354	146493413	X14355	0.75	1.79	0.51
A_16_P15290179	146512434	146512493	X14355	0.69	0.83	-0.13
A_16_P00161704	146525071	146525125	X14355	1.94	2.30	1.23
A_16_P15290229	146534262	146534313	X14355	1.12	2.59	0.59
A_16_P00161710	146549143	146549202	X14355	1.30	2.03	0.35
A_16_P00161715	146581869	146581928	chr1:146581869-146581928	0.70	2.45	0.93
A_16_P15290463	146628190	146628238	BC068044	0.85	1.44	0.85
A_14_P100406	146628218	146628264	BC068044	1.01	1.34	0.74
A_14_P128571	146634734	146634790	chr1:146634734-146634790	1.23	1.60	0.87
A_16_P00161734	146634883	146634942	BC071638	1.81	1.64	1.07
A_16_P00161735	146661770	146661829	chr1:146661770-146661829	0.78	1.23	1.03
A_16_P35302700	146665747	146665806	chr1:146665747-146665806	1.80	2.62	1.20
A_14_P112321	146670238	146670297	HIST2H2BE	1.81	1.18	1.18
A_14_P101364	146671976	146672032	HIST2H2AC	1.83	1.18	1.08
A_14_P130835	146672118	146672170	HIST2H2AB	1.93	1.43	1.21
A_16_P35302745	146679306	146679365	chr1:146679306-146679365	1.35	2.26	0.98
A_14_P129235	146684362	146684408	BOLA1	1.13	1.86	0.60
A_14_P126260	146687961	146688020	BC034038	1.80	2.40	1.16
A_14_P136690	146692381	146692434	SV2A	1.88	2.17	1.08
A_16_P35302805	146696728	146696787	SV2A	1.88	2.48	1.25

A_16_P00161794	146700170	146700227	SV2A	1.91	1.85	1.10
A_16_P00161801	146703136	146703195	chr1:146703136-146703195	1.40	1.40	1.00
A_14_P108957	146709632	146709691	SF3B4	1.40	2.11	1.07
A_14_P133628	146713854	146713913	MTMR11	1.07	1.71	0.92
A_16_P15290720	146722434	146722493	chr1:146722434-146722493	1.65	1.87	1.07
A_16_P15290739	146728112	146728171	BC015719	1.75	2.22	1.09
A_14_P129703	146732268	146732327	ZA20D1	1.12	1.61	1.04
A_16_P15290766	146744845	146744904	ZA20D1	1.38	1.61	1.00
A_16_P15290785	146752112	146752171	ZA20D1	1.58	1.92	1.14
A_16_P15290820	146765214	146765273	ZA20D1	1.77	1.70	1.11
A_16_P15290838	146772938	146772997	ZA20D1	0.90	1.29	0.97
A_16_P15290860	146779880	146779939	ZA20D1	1.19	1.22	0.68
A_14_P130162	146786984	146787043	ZA20D1	1.38	2.27	1.03
A_16_P00161964	146803140	146803199	chr1:146803140-146803199	1.43	2.16	0.98
A_16_P00161991	146819417	146819476	chr1:146819417-146819476	1.61	1.75	1.19
A_16_P35303199	146828798	146828857	chr1:146828798-146828857	1.73	1.67	1.11
A_16_P15291032	146845203	146845262	chr1:146845203-146845262	1.83	1.30	1.05
A_16_P00162031	146853748	146853807	VPS45A	1.05	1.23	1.06
A_14_P107801	146866582	146866641	VPS45A	1.88	2.21	1.28
A_16_P00162074	146877233	146877292	VPS45A	1.42	1.17	1.14
A_16_P15291143	146888806	146888865	VPS45A	1.26	2.09	0.92
A_16_P00162089	146895661	146895720	VPS45A	1.41	2.04	1.08
A_16_P15291173	146906824	146906883	VPS45A	1.26	1.49	1.03
A_14_P138756	146913737	146913796	VPS45A	0.62	1.60	0.90
A_16_P00162118	146922605	146922664	VPS45A	1.56	2.15	1.11
A_16_P00162127	146930257	146930316	VPS45A	1.54	2.32	1.24
A_14_P103528	146938814	146938873	PLEKHO1	1.68	1.55	1.13
A_16_P00162167	146949248	146949307	chr1:146949248-146949307	1.57	1.96	1.04
A_16_P00162171	146958918	146958977	chr1:146958918-146958977	1.95	1.89	1.20
A_16_P15291324	146972839	146972898	chr1:146972839-146972898	1.66	2.02	1.15
A_16_P15291352	146994437	146994496	chr1:146994437-146994496	1.47	1.80	1.13
A_16_P15291368	147000230	147000289	chr1:147000230-147000289	1.60	1.11	1.27
A_14_P118277	147005270	147005329	ANP32E	2.03	1.90	1.15
A_14_P110902	147011987	147012045	ANP32E	1.69	2.04	1.15
A_14_P136454	147014516	147014575	ANP32E	1.37	2.18	0.92
A_16_P15291419	147018750	147018809	ANP32E	0.82	1.06	0.85
A_16_P15291443	147029887	147029946	chr1:147029887-147029946	1.45	2.06	1.11
A_16_P15291453	147037238	147037297	chr1:147037238-147037297	0.58	0.72	0.15
A_16_P15291453	147037238	147037297	chr1:147037238-147037297	0.68	1.23	0.25
A_16_P15291453	147037238	147037297	chr1:147037238-147037297	0.75	1.16	0.26
A_16_P15291458	147041227	147041286	chr1:147041227-147041286	1.53	1.46	1.14
A_14_P114014	147047273	147047332	CA14	1.64	1.79	0.90
A_14_P123368	147051327	147051385	APH1A	1.39	2.05	1.06
A_16_P15291511	147054959	147055018	BC068606	1.64	2.05	1.16
A_16_P00162307	147058253	147058312	C1orf54	1.19	1.26	0.85
A_16_P00162313	147061998	147062057	C1orf54	1.89	1.89	0.91
A_14_P120673	147066339	147066398	C1orf54	1.94	2.41	0.94
A_14_P120954	147070449	147070503	C1orf51	1.04	1.97	0.94

A_16_P35303826	147073864	147073923	chr1:147073864-147073923	2.10	2.41	1.18
A_14_P132684	147079620	147079674	MRPS21	2.51	2.93	1.19
A_16_P35303858	147086209	147086268	MRPS21	1.83	2.33	1.15
A_14_P114006	147092292	147092348	MRPS21	1.13	1.32	0.55
A_16_P15291620	147096524	147096583	chr1:147096524-147096583	1.59	1.76	1.16
A_16_P00162367	147105466	147105525	chr1:147105466-147105525	1.36	1.52	1.59
A_14_P132087	147110625	147110683	PRPF3	1.08	1.82	0.82
A_16_P35303941	147120767	147120826	PRPF3	1.92	2.60	1.20
A_16_P35303958	147129345	147129404	PRPF3	-0.16	0.92	0.21
A_14_P109827	147137839	147137898	PRPF3	1.91	1.98	0.86
A_16_P00162420	147149976	147150035	KIAA0460	1.83	1.49	1.10
A_16_P15291743	147156491	147156550	KIAA0460	0.67	0.60	0.98
A_16_P00162441	147167428	147167487	KIAA0460	0.59	1.75	1.12
A_16_P15291784	147182420	147182479	KIAA0460	1.77	1.63	1.34
A_16_P15291795	147192884	147192943	KIAA0460	1.06	1.42	1.27
A_16_P35304083	147199705	147199764	KIAA0460	1.46	2.31	1.18
A_16_P15291821	147205834	147205893	KIAA0460	1.10	2.30	0.89
A_16_P15291835	147214449	147214508	KIAA0460	1.37	2.17	1.19
A_16_P00162491	147231896	147231955	KIAA0460	0.98	1.99	1.18
A_16_P00162499	147244733	147244792	KIAA0460	1.25	1.39	1.16
A_16_P15291891	147250299	147250358	KIAA0460	1.61	1.77	0.98
A_16_P00162516	147256955	147257014	KIAA0460	1.94	2.12	1.11
A_14_P202263	147263264	147263323	chr1:147263264-147263323	0.83	2.91	1.05
A_14_P111509	147272889	147272943	chr1:147272889-147272943	1.47	2.53	0.99
A_16_P15291960	147276676	147276735	TARSL1	0.63	1.70	0.91
A_14_P132197	147281712	147281771	TARSL1	1.65	2.45	1.27
A_16_P35304281	147288275	147288334	TARSL1	0.96	2.54	0.42
A_14_P103598	147294311	147294367	ECM1	1.33	1.98	0.78
A_16_P00162587	147299071	147299130	ECM1	1.63	2.00	1.07
A_16_P00162593	147304779	147304838	chr1:147304779-147304838	1.37	1.88	1.15
A_16_P15292035	147309958	147310017	chr1:147309958-147310017	1.47	1.72	1.10
A_16_P00162601	147318920	147318979	chr1:147318920-147318979	0.97	1.63	1.20
A_16_P15292071	147329655	147329714	chr1:147329655-147329714	0.98	2.01	1.03
A_14_P131648	147335277	147335331	ADAMTSL4	1.97	1.98	1.26
A_14_P131428	147335805	147335852	ADAMTSL4	1.98	1.46	0.85
A_14_P132799	147342461	147342513	ADAMTSL4	1.61	2.00	1.00
A_16_P00162643	147345996	147346053	ADAMTSL4	1.18	1.76	1.00
A_16_P00162653	147350532	147350591	chr1:147350532-147350591	1.35	2.44	1.03
A_14_P101402	147360147	147360206	MCL1	1.16	1.45	1.11
A_16_P00162675	147363965	147364020	MCL1	2.17	2.82	1.15
A_16_P35304528	147368112	147368171	chr1:147368112-147368171	0.74	1.89	0.75
A_16_P15292196	147379259	147379318	AK057901	1.54	2.23	1.12
A_16_P15292211	147389938	147389997	AK057901	1.06	1.28	1.07
A_16_P15292217	147394907	147394966	AK057901	1.20	1.71	1.35
A_16_P00162704	147402207	147402266	AK057901	1.51	1.23	1.07
A_14_P104110	147408815	147408874	ENSA	1.15	1.52	0.54
A_16_P15292266	147412654	147412713	ENSA	1.88	2.10	1.14
A_16_P00162726	147423550	147423609	chr1:147423550-147423609	1.44	2.12	0.83

A_16_P15292292	147428107	147428166	chr1:147428107-147428166	1.29	2.24	0.94
A_16_P35304666	147434325	147434384	GOLPH3L	1.70	1.98	0.88
A_14_P119113	147447380	147447432	GOLPH3L	0.85	2.41	0.53
A_16_P35304696	147456929	147456988	GOLPH3L	1.27	1.68	1.05
A_16_P35304721	147468377	147468436	GOLPH3L	1.20	2.55	1.03
A_16_P35304736	147473938	147473997	GOLPH3L	1.41	1.29	0.99
A_16_P15292391	147480230	147480289	GOLPH3L	1.63	2.15	1.10
A_16_P35304775	147485613	147485672	HORMAD1	1.21	1.54	0.97
A_14_P114946	147493803	147493862	HORMAD1	0.98	1.95	0.65
A_16_P00162816	147499616	147499675	HORMAD1	1.28	1.73	1.01
A_14_P119560	147506206	147506265	HORMAD1	0.69	1.50	0.87
A_16_P15292467	147511797	147511856	chr1:147511797-147511856	0.84	1.37	1.06
A_16_P15292478	147518625	147518684	CTSS	0.64	2.16	0.91
A_16_P00162843	147528905	147528964	CTSS	1.63	2.23	1.14
A_16_P00162847	147535677	147535736	CTSS	1.04	2.50	0.73
A_14_P103986	147551272	147551330	CTSS	1.10	1.62	1.07
A_16_P15292546	147558167	147558226	chr1:147558167-147558226	1.62	1.48	1.22
A_16_P15292559	147565984	147566043	chr1:147565984-147566043	1.49	1.41	1.12
A_16_P00162887	147571011	147571070	chr1:147571011-147571070	1.44	2.12	1.06
A_14_P115591	147584714	147584768	CTSK	1.48	2.45	0.99
A_16_P00162917	147591718	147591777	CTSK	0.95	1.00	0.89
A_16_P00162929	147596737	147596796	ARNT	1.44	1.85	1.25
A_16_P00162943	147602620	147602679	ARNT	1.97	2.16	1.28
A_14_P138897	147612116	147612175	ARNT	0.89	1.43	0.80
A_14_P135828	147622010	147622063	ARNT	1.64	2.34	0.89
A_14_P136306	147631793	147631852	ARNT	1.41	2.77	1.05
A_16_P00162995	147638288	147638347	ARNT	1.27	1.63	0.98
A_16_P15292764	147652348	147652407	ARNT	1.21	1.88	1.17
A_14_P134059	147659658	147659717	ARNT	1.14	1.74	1.10
A_16_P35305212	147666267	147666326	chr1:147666267-147666326	1.75	2.22	1.14
A_16_P15292818	147675267	147675326	chr1:147675267-147675326	1.43	2.10	0.87
A_16_P15292831	147683866	147683925	chr1:147683866-147683925	1.81	1.34	1.25
A_16_P00163050	147692034	147692093	chr1:147692034-147692093	1.54	1.14	1.21
A_16_P00163057	147700452	147700511	chr1:147700452-147700511	1.89	2.51	1.05
A_16_P15292877	147714062	147714121	SETDB1	1.71	2.20	1.26
A_16_P35305316	147728331	147728390	SETDB1	1.81	2.39	0.95
A_14_P100717	147736417	147736476	SETDB1	1.66	2.06	1.16
A_16_P00163094	147744866	147744925	SETDB1	1.64	2.41	1.21
A_16_P35305389	147750204	147750263	SETDB1	1.56	1.71	1.06
A_14_P128132	147754603	147754648	LASS2	2.08	2.13	1.28
A_14_P112869	147757238	147757297	LASS2	1.78	2.34	1.13
A_16_P00163131	147761068	147761127	chr1:147761068-147761127	1.87	1.55	1.27
A_16_P00163139	147767223	147767277	chr1:147767223-147767277	1.45	1.31	0.85
A_16_P00163146	147771070	147771125	ANXA9	1.46	2.27	0.77
A_16_P15293024	147774882	147774941	ANXA9	1.49	2.08	0.90
A_14_P128620	147779483	147779542	ANXA9	1.49	0.91	1.01
A_14_P108204	147782393	147782452	FAM63A	1.32	1.71	1.13
A_14_P130483	147783778	147783823	FAM63A	1.14	1.56	1.00

A_14_P130500	147791827	147791886	FAM63A	1.57	1.65	1.09
A_14_P130500	147791827	147791886	FAM63A	1.49	1.72	1.14
A_14_P130500	147791827	147791886	FAM63A	1.46	1.35	1.11
A_16_P00163196	147799028	147799087	PRUNE	0.93	1.66	0.93
A_16_P35305585	147803967	147804026	PRUNE	1.55	2.03	0.97
A_14_P100336	147812113	147812172	PRUNE	1.43	2.08	0.82
A_16_P00163222	147819305	147819364	PRUNE	1.01	1.59	1.18
A_16_P00163231	147823026	147823085	BNIPL	1.72	2.31	1.06
A_14_P102385	147826899	147826957	BNIPL	1.80	1.58	1.18
A_16_P00163240	147831588	147831647	BNIPL	1.30	1.56	1.08
A_14_P119371	147836482	147836541	C1orf56	1.25	1.19	0.53
A_14_P200031	147836751	147836810	C1orf56	1.33	1.50	0.93
A_14_P136601	147840673	147840718	CDC42SE1	1.47	2.17	0.96
A_14_P102041	147845819	147845873	MLLT11	1.96	0.60	1.22
A_14_P114453	147850106	147850165	MLLT11	0.96	1.84	0.84
A_14_P108384	147853680	147853739	MLLT11	0.90	2.44	0.70
A_16_P15293268	147864527	147864586	MGC29891	1.62	1.33	0.95
A_16_P15293283	147878699	147878758	MGC29891	1.14	1.65	1.02
A_14_P114070	147892583	147892642	MGC29891	1.68	2.46	1.20
A_16_P15293333	147903945	147904004	MGC29891	1.15	2.22	1.12
A_16_P00163343	147914814	147914873	chr1:147914814-147914873	1.60	1.51	1.01
A_16_P35305887	147920206	147920255	SEMA6C	0.81	1.34	0.69
A_16_P00163364	147924248	147924307	SEMA6C	2.14	2.34	1.31
A_14_P106988	147930006	147930065	SEMA6C	1.88	2.26	1.01
A_16_P15293429	147934502	147934561	chr1:147934502-147934561	1.60	2.04	1.08
A_16_P15293432	147939539	147939598	chr1:147939539-147939598	1.58	1.83	1.09
A_14_P110629	147942498	147942555	TNFAIP8L2	1.53	2.18	1.02
A_16_P35305969	147946165	147946224	LYSMD1	1.43	-0.18	1.14
A_16_P15293459	147949840	147949899	LYSMD1	1.56	1.89	0.99
A_14_P116662	147953020	147953079	SCNM1	1.63	2.09	1.26
A_14_P137815	147958917	147958967	TMOD4	1.35	2.32	1.11
A_16_P00163430	147961995	147962054	VPS72	1.63	2.36	1.01
A_14_P110081	147965885	147965941	VPS72	1.39	2.15	1.03
A_16_P15293513	147972464	147972523	VPS72	1.66	1.60	1.01
A_16_P15293537	147983390	147983449	chr1:147983390-147983449	1.21	1.99	1.25
A_14_P136624	147990060	147990119	PIP5K1A	0.88	2.50	1.05
A_16_P15293568	147999328	147999387	PIP5K1A	1.21	1.95	1.08
A_16_P15293579	148007028	148007087	PIP5K1A	1.53	2.65	1.11
A_14_P138128	148012908	148012966	PIP5K1A	1.37	1.35	0.67
A_16_P00163495	148025554	148025613	PIP5K1A	1.87	2.03	0.88
A_16_P00163501	148033355	148033414	PIP5K1A	1.41	2.35	0.90
A_16_P35306206	148037312	148037371	chr1:148037312-148037371	1.33	1.98	1.13
A_16_P15293657	148047648	148047706	PSMD4	1.58	2.32	1.06
A_14_P137809	148052253	148052312	PSMD4	1.52	1.57	1.14
A_16_P15293678	148057398	148057457	chr1:148057398-148057457	1.89	1.94	1.01
A_16_P00163531	148066774	148066829	BC021024	2.06	0.98	1.23
A_14_P120078	148071561	148071613	ZNF687	1.44	1.73	0.85
A_16_P35306308	148076830	148076889	ZNF687	0.97	1.89	0.72

A_16_P15293744	148081128	148081185	PIK4CB	1.06	2.09	1.22
A_16_P00163571	148086513	148086572	PIK4CB	1.05	1.70	1.00
A_16_P15293771	148092018	148092077	PIK4CB	1.60	2.20	1.12
A_16_P35306389	148100241	148100300	PIK4CB	1.97	1.95	1.09
A_14_P113673	148111268	148111327	PIK4CB	1.95	3.00	1.18
A_16_P00163623	148118106	148118165	chr1:148118106-148118165	1.31	1.59	0.94
A_16_P15293857	148122275	148122328	chr1:148122275-148122328	1.35	1.84	1.17
A_14_P123584	148130607	148130666	RFX5	1.55	2.07	1.03
A_16_P35306490	148135976	148136035	chr1:148135976-148136035	1.77	2.34	1.18
A_16_P35306495	148142293	148142352	chr1:148142293-148142352	1.45	1.74	1.05
A_16_P15293897	148146197	148146256	chr1:148146197-148146256	1.36	2.17	1.41
A_14_P127389	148149896	148149950	SELENBP1	1.02	0.97	0.28
A_14_P128845	148154178	148154234	SELENBP1	1.62	1.86	0.91
A_14_P108679	148157189	148157248	SELENBP1	1.14	1.29	1.16
A_16_P00163676	148162017	148162076	chr1:148162017-148162076	1.48	2.47	0.93
A_16_P15293955	148167701	148167760	chr1:148167701-148167760	1.42	1.66	0.85
A_16_P00163683	148175893	148175952	chr1:148175893-148175952	1.68	1.88	0.94
A_16_P35306583	148181680	148181739	chr1:148181680-148181739	1.66	1.87	1.08
A_14_P133049	148186104	148186157	PSMB4	1.30	2.28	0.88
A_16_P00163711	148192760	148192819	POGZ	1.63	1.80	1.30
A_16_P35306647	148197943	148198002	POGZ	1.83	1.50	0.93
A_16_P15294040	148209443	148209502	POGZ	1.08	2.02	1.10
A_16_P15294040	148209443	148209502	POGZ	1.05	1.60	0.98
A_16_P15294040	148209443	148209502	POGZ	1.06	1.80	1.16
A_14_P112506	148215123	148215182	POGZ	0.65	1.88	0.99
A_16_P00163760	148227644	148227703	POGZ	1.84	2.04	1.26
A_16_P15294095	148233788	148233847	POGZ	1.42	1.82	0.93
A_16_P15294110	148240982	148241041	POGZ	1.37	2.31	1.10
A_16_P35306771	148246425	148246484	chr1:148246425-148246484	1.92	2.02	1.30
A_16_P15294154	148257814	148257873	chr1:148257814-148257873	0.90	1.28	0.89
A_14_P112780	148280933	148280992	chr1:148280933-148280992	1.45	1.96	0.88
A_16_P15294221	148294190	148294249	chr1:148294190-148294249	1.42	1.81	1.00
A_14_P118209	148302866	148302925	CGN	1.19	2.64	1.14
A_16_P15294269	148312324	148312382	CGN	1.60	2.14	0.92
A_16_P00163885	148317311	148317370	CGN	0.95	1.80	0.86
A_16_P00163900	148324127	148324186	CGN	1.76	2.01	1.21
A_14_P112451	148335580	148335639	TUFT1	1.07	1.85	0.78
A_16_P15294364	148351632	148351691	TUFT1	1.41	2.68	1.15
A_16_P15294387	148359750	148359809	TUFT1	1.19	1.96	1.05
A_14_P117207	148368050	148368109	TUFT1	1.34	2.25	0.46
A_16_P00163979	148374743	148374802	chr1:148374743-148374802	1.75	2.61	1.11
A_16_P15294454	148382536	148382595	chr1:148382536-148382595	1.28	1.86	0.75
A_16_P35307134	148390052	148390111	chr1:148390052-148390111	1.23	1.82	0.97
A_14_P123804	148397409	148397454	chr1:148397409-148397454	2.30	1.57	1.28
A_16_P15294510	148409413	148409472	SNX27	1.45	2.37	1.09
A_14_P132995	148424401	148424452	SNX27	1.56	2.23	1.11
A_16_P15294557	148441298	148441357	SNX27	1.43	2.56	1.16
A_14_P111429	148446507	148446566	SNX27	0.95	1.95	0.75

A_16_P00164063	148454106	148454165	SNX27	1.72	1.91	1.12
A_16_P15294595	148462845	148462904	SNX27	1.03	2.31	1.05
A_16_P35307277	148468971	148469030	SNX27	1.44	1.71	1.27
A_16_P15294626	148476622	148476681	SNX27	1.38	2.06	1.16
A_14_P124099	148482652	148482711	SNX27	1.74	1.31	1.21
A_14_P105478	148491343	148491401	TNRC4	1.36	2.08	1.02
A_14_P122874	148493678	148493732	TNRC4	1.05	1.83	1.19
A_16_P00164125	148497871	148497930	TNRC4	1.71	0.56	1.09
A_16_P35307422	148504390	148504449	AK093351	2.04	2.52	1.10
A_16_P00164159	148513095	148513154	AK093351	1.35	2.14	0.94
A_16_P15294776	148524642	148524701	chr1:148524642-148524701	1.21	0.97	0.85
A_16_P35307483	148536355	148536414	chr1:148536355-148536414	0.76	2.21	0.93
A_16_P00164178	148540789	148540836	chr1:148540789-148540836	1.06	2.06	0.93
A_14_P116957	148545402	148545448	MRPL9	0.80	0.92	0.28
A_16_P00164193	148549344	148549403	BC073949	1.53	1.74	1.11
A_14_P200032	148554071	148554130	OAZ3	1.77	2.10	1.12
A_14_P120948	148556830	148556885	OAZ3	1.56	1.75	1.04
A_14_P139391	148557393	148557452	TDRKH	1.40	2.00	1.14
A_16_P00164220	148564692	148564751	TDRKH	1.84	2.04	1.49
A_16_P00164232	148574005	148574064	TDRKH	1.89	2.13	1.40
A_16_P00164241	148577324	148577383	BC038201	0.79	2.32	0.99
A_16_P00164245	148582215	148582274	chr1:148582215-148582274	1.35	1.99	1.23
A_16_P35307630	148585774	148585833	chr1:148585774-148585833	1.80	2.62	1.40
A_14_P110233	148593427	148593486	RORC	2.24	2.67	1.40
A_14_P108848	148597632	148597691	RORC	1.67	1.74	1.28
A_14_P113616	148601189	148601248	RORC	1.38	1.71	1.16
A_14_P113616	148601189	148601248	RORC	1.33	1.33	1.10
A_14_P113616	148601189	148601248	RORC	1.34	1.71	1.18
A_16_P00164291	148604656	148604715	RORC	1.28	2.69	1.55
A_16_P15294985	148609931	148609980	RORC	1.78	2.01	1.44
A_16_P15295026	148621638	148621695	chr1:148621638-148621695	1.60	2.32	1.29
A_16_P00164335	148627451	148627510	chr1:148627451-148627510	1.73	1.75	1.21
A_14_P110983	148634829	148634888	THEM5	0.84	2.21	0.55
A_16_P15295098	148644345	148644404	chr1:148644345-148644404	1.56	2.01	1.04
A_16_P35307849	148648618	148648677	chr1:148648618-148648677	2.26	1.91	1.57
A_16_P35307863	148656482	148656541	AK130716	1.24	1.85	1.30
A_16_P15295140	148662372	148662431	THEM4	1.51	2.01	1.16
A_16_P35307893	148669922	148669981	THEM4	1.30	1.59	1.22
A_14_P128983	148680539	148680595	THEM4	1.42	1.52	1.18
A_16_P00164415	148688559	148688615	THEM4	1.72	1.98	1.31
A_16_P00164422	148696302	148696361	chr1:148696302-148696361	2.23	2.22	1.65
A_16_P00164432	148710468	148710527	chr1:148710468-148710527	1.37	3.95	1.32
A_16_P35307996	148732810	148732869	chr1:148732810-148732869	1.71	2.36	1.46
A_16_P15295274	148751441	148751500	chr1:148751441-148751500	0.93	1.97	1.32
A_16_P00164481	148757339	148757398	chr1:148757339-148757398	1.82	2.03	1.28
A_16_P15295302	148763956	148764015	chr1:148763956-148764015	1.10	1.45	1.41
A_14_P128097	148769828	148769887	S100A10	1.46	2.00	1.26
A_16_P35308098	148776941	148777000	S100A10	1.14	0.91	1.39

A_16_P15295370	148785288	148785347	AK125884	1.73	1.79	1.85
A_16_P15295389	148791855	148791914	AK125884	1.69	2.16	1.50
A_16_P15295399	148796548	148796607	AK125884	0.86	2.20	0.69
A_16_P00164557	148802951	148803005	AK125884	1.80	1.97	1.27
A_16_P15295442	148810268	148810327	AK125884	1.11	1.94	1.13
A_14_P129524	148820622	148820681	S100A11	1.74	2.07	1.58
A_16_P35308245	148825398	148825457	AK125884	1.62	1.78	1.66
A_16_P00164601	148830425	148830484	chr1:148830425-148830484	1.62	2.05	1.45
A_16_P35308273	148836726	148836785	chr1:148836726-148836785	1.84	1.73	1.41
A_16_P15295552	148849263	148849322	chr1:148849263-148849322	1.08	2.15	1.41
A_16_P35308332	148857604	148857663	chr1:148857604-148857663	1.42	1.83	1.35
A_16_P15295584	148862321	148862380	chr1:148862321-148862380	1.56	2.11	1.51
A_16_P35308382	148872969	148873028	TCHHL1	1.17	2.17	1.38
A_16_P35308399	148878193	148878252	chr1:148878193-148878252	1.82	1.58	1.17
A_16_P00164682	148882526	148882585	chr1:148882526-148882585	1.27	1.34	1.19
A_16_P00164682	148882526	148882585	chr1:148882526-148882585	1.26	1.15	1.19
A_16_P00164682	148882526	148882585	chr1:148882526-148882585	1.25	1.62	1.12
A_16_P00164696	148892561	148892620	CR602107	1.58	1.49	1.32
A_14_P101186	148900575	148900634	AY827491	1.72	2.66	1.39
A_16_P15295732	148913041	148913100	chr1:148913041-148913100	1.25	2.36	1.11
A_16_P00164752	148927292	148927351	chr1:148927292-148927351	1.39	2.01	1.21
A_14_P111291	148940204	148940263	AY396742	1.21	2.29	1.23
A_14_P201770	148940626	148940681	AY396742	1.40	2.41	1.38
A_16_P00164791	148953623	148953682	chr1:148953623-148953682	1.43	2.05	1.09
A_16_P15295847	148964780	148964839	chr1:148964780-148964839	1.24	1.50	0.98
A_16_P15295888	148977484	148977543	CR933683	1.61	1.36	0.85
A_16_P15295901	148984579	148984638	CR933683	0.99	1.75	1.24
A_16_P15295920	148993070	148993129	CR933683	1.67	2.69	1.27
A_16_P15295925	148997700	148997759	HRNR	1.29	2.47	1.25
A_16_P15295958	149006682	149006740	HRNR	1.94	2.25	1.29
A_16_P00164870	149013903	149013962	chr1:149013903-149013962	1.15	1.07	0.83
A_16_P00164874	149021592	149021651	chr1:149021592-149021651	1.31	2.59	1.12
A_16_P15295998	149027733	149027791	chr1:149027733-149027791	1.99	2.18	1.46
A_16_P15296030	149040203	149040260	BC014333	1.18	2.55	1.32
A_16_P15296036	149086269	149086328	chr1:149086269-149086328	1.13	1.35	1.13
A_16_P00164904	149093824	149093877	FLG	1.45	1.35	1.23
A_14_P109748	149100220	149100279	FLG	1.42	0.88	1.15
A_16_P00164914	149112042	149112101	AK056431	1.35	0.87	1.29
A_16_P35308883	149117616	149117675	AK056431	1.65	0.90	1.20
A_16_P15296135	149124516	149124575	AK056431	1.02	0.42	1.30
A_16_P15296152	149129614	149129672	AK056431	1.32	1.34	1.32
A_16_P00164952	149135090	149135149	FLG2	1.21	0.82	0.79
A_16_P35308973	149145267	149145326	FLG2	1.48	0.85	1.15
A_16_P15296229	149150889	149150948	AK056431	1.30	0.90	1.19
A_16_P35309009	149161192	149161251	chr1:149161192-149161251	1.17	1.31	0.53
A_16_P00164994	149173414	149173473	chr1:149173414-149173473	1.56	1.30	1.27
A_16_P00165003	149179668	149179727	chr1:149179668-149179727	1.45	1.04	1.25
A_16_P00165009	149185409	149185468	chr1:149185409-149185468	0.58	1.11	1.04

A_16_P00165011	149189880	149189939	chr1:149189880-149189939	1.99	0.91	1.28
A_14_P115963	149197748	149197806	CRNN	1.54	1.10	1.29
A_16_P35309093	149206382	149206441	chr1:149206382-149206441	1.40	1.64	1.23
A_16_P00165043	149210692	149210748	chr1:149210692-149210748	1.45	0.31	1.30
A_16_P35309107	149215643	149215702	chr1:149215643-149215702	1.44	2.14	1.30
A_16_P15296352	149222930	149222989	chr1:149222930-149222989	0.84	0.41	0.91
A_16_P00165089	149247137	149247196	chr1:149247137-149247196	1.88	1.50	1.27
A_16_P15296455	149278545	149278604	chr1:149278545-149278604	1.56	1.41	-0.13
A_16_P35309244	149288712	149288771	chr1:149288712-149288771	1.45	1.38	-0.06
A_16_P35309258	149293067	149293126	chr1:149293067-149293126	1.40	0.44	0.04
A_14_P200033	149297512	149297562	LCE5A	1.49	0.53	0.10
A_14_P137720	149300646	149300705	C1orf42	1.89	1.48	-0.02
A_16_P15296547	149307353	149307412	chr1:149307353-149307412	1.62	0.49	0.07
A_16_P15296557	149312253	149312312	chr1:149312253-149312312	1.61	0.89	0.19
A_16_P15296568	149321549	149321608	chr1:149321549-149321608	0.99	1.39	-0.23
A_16_P15296591	149328355	149328414	chr1:149328355-149328414	1.49	1.59	0.00
A_16_P00165206	149344354	149344413	chr1:149344354-149344413	1.84	0.66	-0.10
A_14_P138487	149352010	149352067	LCE3E	1.31	1.12	0.15
A_16_P15296646	149358185	149358244	chr1:149358185-149358244	0.74	0.03	-0.15
A_16_P00165237	149362629	149362688	chr1:149362629-149362688	1.93	1.27	0.11
A_14_P121322	149365900	149365944	LCE3D	0.90	0.75	-0.04
A_16_P15296678	149369522	149369575	chr1:149369522-149369575	-0.89	-1.63	-0.75
A_16_P15296679	149382933	149382978	chr1:149382933-149382978	-1.62	0.10	-0.93
A_14_P100633	149386203	149386262	chr1:149386203-149386262	-2.34	-2.92	-0.69
A_16_P15296703	149390893	149390952	chr1:149390893-149390952	-5.23	-2.67	-3.38
A_16_P00165273	149394958	149395017	chr1:149394958-149395017	-3.49	-2.36	-2.13
A_14_P112866	149399307	149399354	chr1:149399307-149399354	0.06	1.72	-0.27
A_16_P15296737	149403519	149403578	chr1:149403519-149403578	0.93	0.50	-0.03
A_14_P112404	149408173	149408229	chr1:149408173-149408229	1.43	1.31	0.17
A_16_P35309540	149413396	149413455	chr1:149413396-149413455	1.74	1.18	-0.03
A_16_P00165314	149424144	149424203	chr1:149424144-149424203	1.05	0.92	-0.04
A_16_P00165327	149432379	149432438	chr1:149432379-149432438	1.94	1.46	0.24
A_16_P35309581	149437459	149437518	chr1:149437459-149437518	0.97	1.32	0.03
A_16_P35309611	149445428	149445487	chr1:149445428-149445487	1.70	1.38	0.03
A_14_P200034	149450117	149450161	LCE2D	1.08	1.01	0.16
A_16_P35309639	149453552	149453611	chr1:149453552-149453611	1.35	1.35	0.01
A_14_P200035	149461953	149462002	LCE2C	1.19	1.17	-0.11
A_14_P114063	149462743	149462802	chr1:149462743-149462802	1.54	0.60	-0.02
A_16_P15296915	149466277	149466336	chr1:149466277-149466336	0.97	1.36	-0.06
A_14_P133620	149472740	149472793	LCE2B	1.72	1.02	0.16
A_16_P15296942	149476933	149476992	chr1:149476933-149476992	0.99	1.02	-0.16
A_14_P100722	149484402	149484458	LCE2A	1.00	0.67	0.26
A_14_P200036	149484785	149484829	LCE2A	1.97	0.95	0.20
A_16_P15296965	149491029	149491088	chr1:149491029-149491088	1.18	0.98	-0.02
A_14_P132523	149494562	149494621	chr1:149494562-149494621	1.35	1.14	-0.25
A_16_P00165427	149502562	149502618	chr1:149502562-149502618	0.81	0.19	0.28
A_16_P35309785	149507122	149507181	chr1:149507122-149507181	1.59	1.05	0.20
A_16_P15297045	149515265	149515324	chr1:149515265-149515324	1.32	1.57	-0.02

A_16_P15297076	149525311	149525370	chr1:149525311-149525370	1.09	0.07	0.04
A_16_P00165486	149532002	149532061	chr1:149532002-149532061	0.95	0.32	-0.38
A_16_P15297115	149538785	149538844	chr1:149538785-149538844	1.09	1.14	0.16
A_16_P00165508	149545523	149545582	C1orf45	1.00	0.20	0.24
A_16_P00165520	149552115	149552174	chr1:149552115-149552174	1.23	1.23	-0.02
A_16_P00165526	149555853	149555912	chr1:149555853-149555912	1.63	1.02	0.06
A_14_P137710	149561702	149561761	chr1:149561702-149561761	0.99	1.02	-0.17
A_16_P00165545	149568628	149568687	chr1:149568628-149568687	1.51	0.23	0.16
A_14_P131413	149572677	149572734	LCE1E	0.98	0.99	0.10
A_14_P200037	149573806	149573854	LCE1E	1.09	0.97	0.02
A_16_P35309989	149579637	149579696	chr1:149579637-149579696	1.50	2.40	-0.33
A_16_P35310000	149582884	149582943	chr1:149582884-149582943	2.58	2.69	0.13
A_16_P15297242	149586912	149586971	chr1:149586912-149586971	0.95	0.60	-0.24
A_14_P138003	149591108	149591167	LCE1C	1.20	0.94	-0.06
A_14_P134645	149598429	149598476	LCE1B	1.52	0.81	0.24
A_16_P15297284	149604744	149604803	chr1:149604744-149604803	1.39	1.33	-0.15
A_16_P15297284	149604744	149604803	chr1:149604744-149604803	1.39	1.67	-0.15
A_16_P15297284	149604744	149604803	chr1:149604744-149604803	1.47	1.60	-0.07
A_14_P127411	149612897	149612956	chr1:149612897-149612956	1.14	1.06	-0.25
A_16_P00165611	149624783	149624842	chr1:149624783-149624842	1.30	1.40	-0.04
A_16_P00165623	149629633	149629689	chr1:149629633-149629689	0.95	-0.21	-0.09
A_14_P110026	149635840	149635899	chr1:149635840-149635899	1.67	1.01	0.04
A_16_P15297375	149645065	149645123	chr1:149645065-149645123	1.77	0.86	-0.06
A_16_P15297404	149655843	149655902	chr1:149655843-149655902	1.21	1.01	-0.30
A_16_P15297419	149660287	149660346	chr1:149660287-149660346	1.57	0.41	0.12
A_14_P131912	149668094	149668153	SMCP	1.03	0.11	0.18
A_16_P00165690	149670525	149670584	SMCP	1.35	0.42	-0.04
A_16_P00165697	149681928	149681987	chr1:149681928-149681987	1.93	1.35	-0.05
A_16_P00165706	149689476	149689535	chr1:149689476-149689535	1.41	0.74	0.15
A_14_P120925	149695407	149695454	IVL	1.17	0.52	-0.02
A_16_P35310274	149705527	149705586	chr1:149705527-149705586	1.11	0.52	0.00
A_16_P00165725	149709712	149709771	chr1:149709712-149709771	0.98	1.06	0.05
A_16_P35310301	149719706	149719765	BC042056	0.61	1.41	0.11
A_16_P15297558	149730587	149730646	BC042056	1.54	1.44	0.12
A_16_P15297578	149736718	149736777	chr1:149736718-149736777	1.20	0.80	0.00
A_16_P00165776	149743524	149743583	chr1:149743524-149743583	1.62	1.01	0.05
A_16_P35310382	149751935	149751994	chr1:149751935-149751994	1.36	1.02	0.17
A_14_P122655	149757296	149757355	SPRR4	1.04	-0.19	0.26
A_16_P15297647	149761486	149761545	chr1:149761486-149761545	1.05	1.64	-0.28
A_14_P124410	149770646	149770705	SPRR1A	0.72	1.64	0.25
A_16_P00165836	149777743	149777802	chr1:149777743-149777802	1.82	0.72	0.23
A_16_P00165841	149784226	149784285	chr1:149784226-149784285	1.61	0.93	0.13
A_14_P116391	149788459	149788518	SPRR3	1.35	0.50	0.03
A_16_P15297722	149794660	149794717	chr1:149794660-149794717	1.89	1.27	0.20
A_16_P35310500	149798238	149798297	chr1:149798238-149798297	2.03	1.32	0.09
A_16_P00165860	149807388	149807447	chr1:149807388-149807447	1.06	-0.03	-0.03
A_16_P00165868	149811209	149811268	chr1:149811209-149811268	1.86	1.34	0.24
A_14_P120073	149817483	149817537	SPRR1B	2.01	1.18	0.26

A_16_P35310549	149823137	149823196	chr1:149823137-149823196	1.05	1.74	0.15
A_16_P15297784	149827259	149827318	SPRR2D	1.06	0.59	-0.04
A_16_P15297798	149830943	149831002	chr1:149830943-149831002	1.50	1.04	0.17
A_14_P119955	149841534	149841593	chr1:149841534-149841593	1.39	1.41	0.07
A_16_P15297818	149846200	149846259	chr1:149846200-149846259	1.35	0.54	0.04
A_16_P00165914	149855551	149855610	chr1:149855551-149855610	1.23	0.91	-0.09
A_16_P00165925	149861919	149861978	chr1:149861919-149861978	1.24	1.32	-0.14
A_16_P35310630	149878373	149878432	chr1:149878373-149878432	1.40	1.31	0.34
A_16_P35310653	149884863	149884922	BC058018	1.12	0.61	-0.18
A_16_P00165939	149897150	149897209	chr1:149897150-149897209	0.91	0.56	-0.05
A_16_P35310670	149901510	149901569	chr1:149901510-149901569	1.03	1.19	-0.13
A_16_P15297903	149908208	149908267	chr1:149908208-149908267	0.95	0.87	-0.18
A_16_P00165957	149912696	149912755	chr1:149912696-149912755	1.58	1.13	0.05
A_16_P15297917	149916836	149916895	chr1:149916836-149916895	0.96	0.62	0.21
A_14_P106802	149926487	149926546	SPRR2C	0.95	0.93	-0.32
A_16_P35310714	149931147	149931206	chr1:149931147-149931206	1.40	1.34	0.04
A_16_P35310720	149935051	149935110	chr1:149935051-149935110	1.13	1.32	0.02
A_16_P15297956	149941355	149941414	chr1:149941355-149941414	1.27	0.61	-0.13
A_16_P00165990	149948489	149948548	chr1:149948489-149948548	1.11	-0.28	-0.13
A_16_P00166018	149962017	149962076	BC038426	1.25	0.80	0.01
A_16_P35310800	149976168	149976227	BC038426	1.36	0.87	-0.25
A_16_P00166034	149980807	149980866	chr1:149980807-149980866	1.31	1.46	0.04
A_16_P00166054	149990231	149990290	LELP1	1.66	0.44	0.28
A_16_P00166067	149996932	149996991	chr1:149996932-149996991	1.27	0.73	0.02
A_16_P15298100	150002450	150002509	chr1:150002450-150002509	1.89	1.15	0.24
A_16_P15298133	150014564	150014623	chr1:150014564-150014623	1.40	1.45	-0.11
A_16_P00166115	150025515	150025574	chr1:150025515-150025574	1.56	0.87	0.18
A_16_P00166123	150033412	150033471	chr1:150033412-150033471	0.88	-1.02	-0.03
A_16_P35310986	150039114	150039173	chr1:150039114-150039173	1.79	0.59	0.14
A_14_P110433	150046473	150046521	LOR	0.76	1.74	-1.19
A_16_P15298234	150051411	150051470	chr1:150051411-150051470	0.44	0.35	0.24
A_16_P00166156	150063480	150063539	chr1:150063480-150063539	1.53	1.59	0.12
A_16_P00166162	150069066	150069125	chr1:150069066-150069125	1.72	0.95	0.21
A_16_P15298258	150075228	150075287	chr1:150075228-150075287	1.70	1.33	0.19
A_16_P00166174	150080788	150080847	chr1:150080788-150080847	1.86	1.14	0.24
A_16_P15298291	150087910	150087968	PGLYRP3	1.49	0.64	0.21
A_14_P125444	150094542	150094601	PGLYRP3	0.85	0.59	-0.39
A_16_P15298333	150099274	150099333	chr1:150099274-150099333	2.67	-0.14	-3.06
A_16_P15298337	150107727	150107786	chr1:150107727-150107786	1.22	0.98	0.29
A_16_P15298343	150115613	150115671	chr1:150115613-150115671	1.99	1.39	0.31
A_14_P102942	150122939	150122998	PGLYRP4	1.11	0.72	-0.16
A_14_P103610	150127788	150127847	PGLYRP4	1.08	0.56	0.00
A_16_P00166238	150132038	150132097	PGLYRP4	1.58	1.08	0.04
A_16_P00166244	150135850	150135909	chr1:150135850-150135909	1.61	-0.23	0.24
A_16_P15298409	150141522	150141581	chr1:150141522-150141581	0.87	0.86	-0.10
A_14_P100749	150145628	150145687	S100A9	1.81	0.81	0.22
A_16_P00166275	150151738	150151797	chr1:150151738-150151797	2.05	1.19	0.32
A_14_P124338	150159434	150159491	S100A12	1.34	0.15	0.14

A_16_P00166293	150165125	150165184	chr1:150165125-150165184	1.61	1.38	0.21
A_16_P15298482	150171444	150171503	chr1:150171444-150171503	1.59	0.44	0.20
A_14_P105363	150175715	150175768	S100A8	1.85	1.00	0.16
A_16_P00166318	150181314	150181373	chr1:150181314-150181373	1.22	1.06	0.13
A_16_P00166332	150190196	150190255	chr1:150190196-150190255	1.35	1.70	0.13
A_16_P00166335	150198752	150198811	chr1:150198752-150198811	0.86	1.33	0.09
A_14_P106240	150203466	150203525	S100A7L1	1.62	1.19	0.12
A_16_P00166363	150217004	150217063	chr1:150217004-150217063	1.47	1.59	0.29
A_16_P15298617	150223298	150223357	chr1:150223298-150223357	1.43	1.65	0.16
A_16_P00166385	150232309	150232368	chr1:150232309-150232368	0.83	1.16	0.04
A_16_P35311450	150236347	150236406	chr1:150236347-150236406	1.22	-0.01	0.17
A_14_P125828	150244667	150244726	S100A7	1.94	1.18	0.49
A_16_P00166403	150249371	150249430	chr1:150249371-150249430	1.36	1.43	-0.04
A_16_P00166406	150254335	150254394	chr1:150254335-150254394	1.36	0.17	0.00
A_16_P15298710	150260693	150260752	chr1:150260693-150260752	1.47	1.13	0.00
A_16_P00166438	150281145	150281204	chr1:150281145-150281204	2.03	1.26	0.26
A_16_P15298818	150303528	150303587	chr1:150303528-150303587	0.65	1.05	-0.03
A_16_P00166481	150316093	150316152	chr1:150316093-150316152	1.58	0.54	0.12
A_16_P00166502	150327194	150327253	S100A5	1.47	0.45	0.03
A_16_P15298879	150332314	150332366	chr1:150332314-150332366	2.22	1.40	0.46
A_16_P00166522	150335661	150335709	chr1:150335661-150335709	1.49	1.17	0.19
A_16_P00166531	150342090	150342149	chr1:150342090-150342149	1.57	0.68	0.23
A_16_P15298906	150346701	150346760	S100A2	1.17	-0.04	0.17
A_16_P00166542	150351657	150351716	chr1:150351657-150351716	1.99	0.87	0.20
A_16_P15298937	150358315	150358374	chr1:150358315-150358374	1.36	0.93	0.16
A_16_P15298950	150365475	150365534	chr1:150365475-150365534	1.49	0.80	0.06
A_16_P00166563	150374011	150374070	chr1:150374011-150374070	1.36	0.95	0.11
A_16_P15298966	150381696	150381754	chr1:150381696-150381754	1.60	1.68	0.22
A_16_P00166572	150389160	150389208	chr1:150389160-150389208	1.62	1.03	0.03
A_14_P118231	150398345	150398396	S100A16	1.45	1.09	0.14
A_14_P107098	150400873	150400920	S100A14	0.75	0.87	0.18
A_14_P105005	150405478	150405537	S100A13	2.04	1.42	0.25
A_16_P15299029	150411809	150411863	S100A13	0.84	0.65	0.11
A_16_P00166617	150417657	150417716	S100A13	1.39	1.11	0.19
A_16_P15299064	150423528	150423587	C1orf77	1.27	1.47	-0.01
A_14_P138311	150426666	150426725	C1orf77	1.45	1.16	0.19
A_14_P123600	150428709	150428768	C1orf77	1.39	1.17	0.04
A_14_P134907	150431078	150431129	AF318362	1.96	0.76	0.26
A_16_P00166648	150435908	150435967	chr1:150435908-150435967	1.68	0.38	0.28
A_16_P00166652	150441251	150441310	chr1:150441251-150441310	1.49	0.95	0.03
A_14_P123709	150447303	150447362	SNAPAP	1.14	0.73	-0.20
A_14_P123851	150448356	150448415	ILF2	1.17	1.59	0.32
A_16_P15299141	150454103	150454162	ILF2	1.70	0.90	0.21
A_16_P15299152	150459454	150459502	chr1:150459454-150459502	1.36	-0.13	0.05
A_16_P00166679	150462960	150463019	chr1:150462960-150463019	1.31	-0.08	0.15
A_14_P125388	150466778	150466829	NPR1	1.61	0.96	0.23
A_16_P15299184	150472058	150472115	NPR1	1.56	0.84	0.32
A_16_P35312089	150479591	150479650	chr1:150479591-150479650	1.22	1.01	0.23

A_16_P15299220	150484983	150485042	chr1:150484983-150485042	1.84	1.26	0.19
A_16_P00166717	150493806	150493865	chr1:150493806-150493865	1.42	0.77	1.05
A_16_P35312111	150503227	150503284	chr1:150503227-150503284	1.63	-0.01	0.81
A_16_P35312114	150509121	150509180	chr1:150509121-150509180	1.47	1.05	0.24
A_16_P00166729	150514577	150514636	INTS3	1.55	0.27	0.05
A_16_P15299269	150522632	150522691	INTS3	1.76	0.53	0.13
A_16_P15299278	150528431	150528488	INTS3	1.57	0.58	-0.08
A_14_P129616	150537880	150537939	INTS3	1.67	1.61	0.23
A_16_P00166778	150545112	150545171	INTS3	2.01	1.46	0.32
A_16_P00166798	150555348	150555407	INTS3	1.31	0.46	-0.12
A_16_P00166809	150559568	150559627	INTS3	1.16	0.42	0.17
A_14_P120830	150564538	150564589	SLC27A3	1.43	0.55	0.09
A_16_P00166831	150573192	150573251	chr1:150573192-150573251	1.34	1.51	0.20
A_16_P00166837	150580301	150580358	chr1:150580301-150580358	2.04	1.92	0.24
A_16_P15299429	150584270	150584326	chr1:150584270-150584326	1.49	0.21	0.19
A_14_P106047	150593514	150593573	GATAD2B	0.69	0.47	0.69
A_16_P35312435	150605831	150605890	GATAD2B	1.77	1.20	0.20
A_16_P00166902	150610827	150610886	GATAD2B	1.64	0.73	0.16
A_16_P15299523	150617300	150617359	AK094665	1.24	0.83	0.12
A_16_P15299526	150622565	150622624	AK094665	1.60	1.16	0.03
A_16_P15299536	150627954	150628013	AK094665	1.50	1.10	0.18
A_16_P00166920	150638137	150638192	AK094665	1.31	1.44	-0.02
A_16_P15299561	150650667	150650726	AK094665	1.75	0.76	0.58
A_14_P134132	150668871	150668930	AK094665	0.79	1.22	0.41
A_16_P35312566	150683019	150683078	AK094665	0.87	0.68	0.36
A_16_P15299647	150689917	150689976	AK094665	1.34	1.23	0.34
A_16_P15299649	150696525	150696584	AK094665	0.73	1.90	0.24
A_16_P00166984	150706383	150706442	AK094665	0.79	0.82	0.25
A_16_P00167007	150720169	150720226	CR613795	1.64	0.70	0.53
A_14_P101801	150724437	150724488	AB007945	1.82	0.96	0.55
A_16_P00167026	150730251	150730310	AB007945	1.90	0.80	0.57
A_14_P129483	150738116	150738167	CRTC2	1.02	1.25	0.34
A_14_P129438	150741505	150741559	CRTC2	1.86	1.28	0.55
A_16_P00167061	150747194	150747251	SLC39A1	1.91	0.47	0.44
A_14_P103242	150752653	150752712	SLC39A1	1.69	1.39	0.50
A_14_P122524	150757886	150757945	CREB3L4	1.86	1.28	0.48
A_14_P116859	150759837	150759896	CREB3L4	0.56	1.29	0.42
A_14_P138500	150760053	150760112	JTB	1.42	1.04	0.40
A_14_P127661	150762434	150762486	JTB	1.43	0.86	0.43
A_16_P15299855	150767104	150767163	chr1:150767104-150767163	1.57	0.83	0.32
A_14_P134188	150769927	150769986	RAB13	1.43	0.87	0.55
A_16_P00167104	150773155	150773214	chr1:150773155-150773214	1.44	1.13	0.16
A_14_P116884	150776992	150777051	RPS27	1.63	1.79	0.42
A_16_P15299888	150780578	150780637	NUP210L	1.15	0.68	0.59
A_16_P35312911	150787228	150787287	NUP210L	1.00	0.30	0.37
A_16_P00167130	150795505	150795564	NUP210L	1.09	0.49	0.21
A_16_P15299943	150808620	150808679	NUP210L	0.80	-0.39	0.18
A_16_P15299957	150815602	150815661	NUP210L	1.23	0.96	0.48

A_16_P15299958	150826059	150826118	NUP210L	1.60	1.17	0.33
A_16_P15299970	150831758	150831817	NUP210L	0.81	0.68	-0.23
A_16_P00167165	150844193	150844252	NUP210L	1.93	0.57	0.23
A_16_P15300003	150855929	150855988	NUP210L	1.05	-0.16	0.10
A_16_P00167177	150865093	150865152	NUP210L	1.69	1.02	0.09
A_16_P15300010	150874123	150874182	NUP210L	0.98	0.99	-0.32
A_14_P112900	150889560	150889619	NUP210L	1.29	0.53	-0.07
A_16_P00167198	150903355	150903411	NUP210L	1.53	0.82	0.06
A_16_P00167205	150912847	150912906	NUP210L	1.40	0.79	-0.04
A_16_P00167211	150921341	150921400	NUP210L	1.16	0.86	0.09
A_16_P35313145	150927091	150927150	AK128000	1.73	1.61	0.27
A_16_P15300117	150938237	150938296	AK128000	1.36	1.25	0.22
A_16_P15300146	150945653	150945712	TPM3	1.56	1.35	0.04
A_14_P105411	150953456	150953515	TPM3	2.06	1.48	0.38
A_16_P00167277	150962590	150962649	TPM3	1.69	0.68	0.05
A_14_P135435	150967081	150967140	TPM3	1.12	1.20	0.16
A_14_P132594	150976096	150976155	TPM3	1.99	0.86	0.03
A_16_P00167312	150984133	150984192	chr1:150984133-150984192	0.88	0.34	0.40
A_16_P00167322	150992398	150992457	C1orf43	1.07	1.36	0.29
A_14_P130581	151000091	151000150	C1orf43	1.33	1.08	0.01
A_14_P118648	151005293	151005352	C1orf43	1.82	1.24	0.15
A_16_P15300323	151010369	151010428	D63478	1.01	0.67	0.05
A_16_P15300336	151014355	151014414	UBAP2L	1.23	0.96	0.14
A_14_P125938	151022590	151022644	UBAP2L	1.58	1.22	0.13
A_16_P15300380	151029414	151029473	UBAP2L	1.63	1.12	0.18
A_16_P00167397	151036526	151036585	UBAP2L	1.76	0.61	0.20
A_16_P15300426	151044472	151044531	UBAP2L	1.55	1.44	0.05
A_16_P15300447	151050438	151050497	UBAP2L	1.51	1.41	0.13
A_14_P109760	151058042	151058094	BC005240	2.05	0.64	0.18
A_14_P109760	151058042	151058094	BC005240	2.07	0.79	0.20
A_14_P109760	151058042	151058094	BC005240	2.04	0.75	0.26
A_14_P110047	151061118	151061177	HAX1	1.91	1.53	0.10
A_16_P15300489	151070714	151070773	chr1:151070714-151070773	1.41	1.25	0.19
A_16_P00167451	151076963	151077022	chr1:151076963-151077022	1.67	1.79	0.08
A_16_P15300501	151085299	151085358	chr1:151085299-151085358	0.82	0.79	-0.37
A_16_P15300506	151091138	151091197	chr1:151091138-151091197	1.56	0.59	0.02
A_16_P00167454	151097291	151097345	chr1:151097291-151097345	1.99	0.84	0.15
A_16_P15300517	151105168	151105227	chr1:151105168-151105227	1.72	0.73	0.20
A_14_P118053	151109453	151109502	AQP10	1.85	1.46	0.17
A_14_P200038	151115557	151115616	ATP8B2	1.39	0.58	0.21
A_16_P00167487	151123145	151123204	ATP8B2	0.99	0.73	0.07
A_16_P15300595	151130394	151130451	ATP8B2	1.22	0.14	-0.10
A_16_P00167515	151135918	151135977	ATP8B2	1.46	0.83	0.39
A_16_P00167527	151145806	151145865	chr1:151145806-151145865	1.84	1.37	0.19
A_16_P15300637	151157555	151157611	chr1:151157555-151157611	0.81	0.47	-0.29
A_16_P15300657	151171112	151171171	chr1:151171112-151171171	1.60	0.19	0.11
A_14_P123543	151192363	151192422	IL6R	1.56	1.45	0.26
A_16_P35313851	151200943	151201002	IL6R	1.29	1.08	-0.04

A_16_P35313874	151209144	151209203	IL6R	1.76	1.79	0.19
A_14_P137954	151220662	151220720	IL6R	1.11	0.67	-0.02
A_16_P15300789	151232310	151232369	IL6R	1.39	1.80	0.11
A_16_P15300820	151245404	151245463	IL6R	0.78	1.42	-0.03
A_14_P131110	151251940	151251999	IL6R	1.28	0.75	0.17
A_16_P15300852	151257160	151257219	X52229	1.18	0.83	0.25
A_16_P35314015	151265307	151265366	SHE	1.70	0.74	0.12
A_14_P139937	151275654	151275713	SHE	1.50	1.52	0.07
A_16_P35314078	151284627	151284686	SHE	1.64	0.96	0.18
A_16_P35314108	151293924	151293983	TDRD10	1.76	1.59	0.21
A_16_P35314116	151299400	151299459	TDRD10	1.77	1.36	0.14
A_14_P102787	151307885	151307944	TDRD10	1.75	1.28	0.22
A_16_P00167719	151320588	151320647	TDRD10	1.64	1.11	0.39
A_16_P35314156	151328951	151329009	TDRD10	1.94	1.47	0.42
A_16_P15301019	151333464	151333520	TDRD10	1.17	0.59	0.08
A_16_P15301042	151339467	151339526	UBE2Q1	2.25	1.76	0.20
A_14_P111131	151342676	151342735	UBE2Q1	1.28	0.86	0.03
A_16_P35314229	151347284	151347343	X52229	1.71	1.18	0.28
A_16_P00167764	151350712	151350770	X52229	2.33	1.56	0.34
A_14_P135667	151354482	151354536	CHRNB2	1.22	0.79	-0.19
A_16_P00167783	151360531	151360582	CHRNB2	1.67	0.98	0.15
A_16_P15301112	151363822	151363881	X52229	0.67	2.41	0.12
A_14_P108088	151369382	151369441	ADAR	1.57	0.99	0.25
A_16_P00167813	151374181	151374240	ADAR	1.73	1.73	0.18
A_16_P15301174	151384110	151384169	ADAR	1.93	1.18	0.18
A_16_P00167846	151391015	151391074	ADAR	0.98	0.93	-0.02
A_16_P35314401	151398288	151398347	ADAR	1.49	1.57	0.01
A_16_P15301237	151406399	151406458	ADAR	1.00	1.39	0.02
A_16_P15301250	151412066	151412125	ADAR	0.79	0.19	-0.02
A_16_P15301264	151422059	151422118	X52229	2.25	0.61	0.21
A_16_P15301270	151431771	151431830	X52229	1.40	1.39	-0.04
A_16_P15301279	151448149	151448208	X52229	1.62	2.04	0.26
A_16_P00167901	151467441	151467499	X52229	1.87	1.02	0.12
A_14_P109889	151481956	151482015	X52229	1.75	1.11	0.37
A_16_P15301364	151491890	151491949	X52229	1.70	1.30	0.04
A_16_P35314580	151501768	151501827	KCNN3	1.95	1.22	0.12
A_16_P00167973	151510253	151510312	KCNN3	1.16	1.29	0.00
A_16_P00167995	151519109	151519168	KCNN3	1.36	0.26	-0.05
A_16_P15301480	151530733	151530792	KCNN3	1.76	0.56	0.31
A_16_P00168029	151539583	151539642	KCNN3	1.64	1.47	0.21
A_16_P15301525	151545372	151545431	KCNN3	1.58	1.77	-0.02
A_14_P119592	151555890	151555949	KCNN3	1.89	0.61	0.16
A_16_P15301570	151561438	151561497	KCNN3	1.39	0.90	0.15
A_16_P15301599	151571738	151571797	KCNN3	1.81	1.18	0.23
A_14_P116117	151581694	151581753	KCNN3	1.56	0.31	-0.12
A_16_P00168103	151589041	151589099	KCNN3	1.17	1.68	0.28
A_16_P15301672	151595014	151595073	KCNN3	0.81	0.83	-0.15
A_16_P15301695	151601563	151601622	KCNN3	1.74	1.64	0.31

A_16_P15301719	151609205	151609264	KCNN3	1.35	0.71	0.18
A_16_P35314953	151625755	151625814	KCNN3	1.68	1.70	0.21
A_16_P00168194	151635077	151635136	KCNN3	1.87	1.27	0.11
A_14_P127061	151654608	151654664	KCNN3	1.28	1.63	0.24
A_16_P00168255	151670728	151670787	X52229	1.44	0.26	-0.10
A_14_P123036	151687895	151687954	X52229	2.03	0.96	0.32
A_16_P00168283	151700258	151700317	X52229	1.97	1.39	0.13
A_16_P15301959	151706303	151706362	X52229	1.25	0.71	-0.05
A_14_P124631	151718294	151718353	PMVK	1.44	0.89	0.10
A_16_P00168304	151723252	151723311	X52229	1.89	1.80	0.30
A_16_P15302016	151729155	151729214	X52229	0.95	2.33	1.81
A_16_P00168323	151735132	151735179	PBXIP1	1.65	1.19	0.13
A_14_P200039	151738128	151738187	PBXIP1	2.24	1.09	0.33
A_14_P135389	151740974	151741033	PBXIP1	1.58	0.97	0.09
A_14_P112902	151745366	151745411	PYGO2	1.77	1.06	0.37
A_14_P109651	151747904	151747949	SHC1	2.05	0.94	0.28
A_14_P136319	151753562	151753607	SHC1	1.25	0.71	0.24
A_14_P109823	151757884	151757943	SHC1	1.14	1.03	-0.08
A_14_P119683	151760912	151760971	CKS1B	1.59	0.99	0.06
A_14_P127745	151763153	151763212	CKS1B	1.60	1.22	0.02
A_14_P121173	151763867	151763926	CKS1B	1.47	0.22	0.13
A_14_P137481	151769284	151769332	FLAD1	1.92	0.89	0.31
A_14_P121932	151773969	151774015	FLAD1	1.71	1.44	0.39
A_14_P122449	151778332	151778377	FLAD1	1.70	1.46	0.15
A_14_P137988	151779518	151779570	LENEP	0.97	0.82	0.16
A_16_P00168392	151785517	151785570	X52229	1.54	0.82	0.14
A_16_P15302189	151788646	151788693	X52229	1.28	1.58	0.22
A_16_P00168406	151792095	151792154	X52229	1.99	0.96	0.37
A_16_P15302215	151795695	151795744	X52229	1.69	1.33	0.15
A_16_P00168422	151799543	151799602	X52229	1.76	1.75	0.52
A_14_P136715	151802511	151802564	X52229	1.40	0.48	0.23
A_16_P00168432	151805695	151805754	DCST2	1.21	0.40	0.21
A_16_P15302254	151809154	151809213	DCST2	0.90	1.50	0.07
A_14_P106381	151815729	151815788	DCST2	1.84	0.00	0.40
A_16_P15302289	151822184	151822243	DCST1	2.32	0.59	0.18
A_16_P35315563	151829191	151829250	DCST1	1.68	0.66	-0.23
A_14_P109688	151835167	151835226	DCST1	1.13	0.64	0.12
A_14_P114199	151838039	151838095	ADAM15	1.73	0.98	0.32
A_16_P00168492	151841982	151842031	ADAM15	1.84	1.35	0.17
A_16_P15302367	151846194	151846242	ADAM15	2.07	1.17	0.07
A_16_P15302384	151851759	151851816	EFNA4	1.52	0.87	-0.11
A_14_P118932	151855007	151855066	EFNA4	1.94	1.16	0.15
A_16_P15302402	151857827	151857886	X52229	1.77	1.60	0.21
A_14_P135793	151865010	151865054	EFNA3	0.81	0.96	0.25
A_14_P102872	151866955	151867008	EFNA3	0.65	-0.70	0.16
A_14_P134411	151873010	151873068	EFNA3	1.56	1.49	0.18
A_16_P35315754	151878132	151878191	X52229	1.72	-0.86	0.22
A_16_P35315764	151890549	151890606	X52229	1.77	1.26	0.08

A_16_P00168571	151896227	151896286	X52229	1.59	1.11	0.37
A_16_P15302513	151904505	151904564	X52229	1.11	1.54	-0.04
A_16_P15302520	151908256	151908315	X52229	1.27	0.75	0.40
A_14_P122370	151915533	151915592	EFNA1	1.08	0.98	0.38
A_14_P133452	151916569	151916628	EFNA1	0.55	0.66	0.04
A_14_P108832	151918996	151919051	EFNA1	1.47	0.90	0.14
A_14_P138772	151924337	151924396	RAG1AP1	1.42	1.83	0.09
A_14_P102538	151925424	151925482	DPM3	1.37	1.29	0.26
A_16_P15302578	151928881	151928940	X52229	0.96	0.65	0.27
A_16_P35315884	151934063	151934107	X52229	0.99	0.63	0.27
A_16_P15302591	151944911	151944966	X52229	0.45	2.03	0.15
A_16_P35315911	151954323	151954382	X52229	1.56	0.50	0.09
A_14_P113322	151958100	151958156	KRTCAP2	1.17	2.06	0.10
A_14_P126551	151963229	151963281	TRIM46	1.80	1.28	0.24
A_16_P00168655	151968006	151968063	TRIM46	1.77	0.84	0.20
A_14_P134330	151973557	151973614	MUC1	1.31	1.37	0.29
A_16_P00168677	151978440	151978495	THBS3	2.06	0.64	0.14
A_14_P131350	151981602	151981656	THBS3	1.11	1.02	0.21
A_16_P35316054	151987501	151987560	THBS3	1.42	1.77	0.40
A_14_P121890	151992836	151992895	MTX1	1.50	1.40	0.11
A_16_P00168711	151998352	151998402	BC000349	0.82	1.93	0.45
A_16_P35316109	151998685	151998734	BC000349	1.55	1.40	0.11
A_16_P00168715	152002641	152002699	BC000349	1.23	0.75	0.26
A_16_P00168720	152006259	152006318	BC000349	0.96	1.13	0.13
A_16_P15302786	152010139	152010198	BC000349	0.60	-0.24	-0.04
A_16_P00168730	152014680	152014736	AK056250	1.18	0.28	0.22
A_14_P104473	152018577	152018631	GBA	1.54	1.26	0.31
A_16_P35316200	152025389	152025448	GBA	1.90	0.54	0.34
A_16_P00168742	152030358	152030408	C1orf2	1.30	0.75	0.02
A_14_P125581	152036329	152036384	C1orf2	1.50	1.60	0.33
A_14_P115285	152036987	152037031	C1orf2	1.39	0.73	0.12
A_14_P133588	152040188	152040246	SCAMP3	1.33	0.85	0.09
A_14_P138183	152045920	152045979	CLK2	0.97	1.47	-0.10
A_14_P109697	152049242	152049301	CLK2	0.97	1.52	0.08
A_14_P109697	152049242	152049301	CLK2	0.99	0.95	-0.05
A_14_P109697	152049242	152049301	CLK2	0.92	1.29	0.14
A_14_P116165	152050861	152050917	CLK2	1.09	0.48	0.11
A_16_P35316316	152055627	152055686	CLK2	1.59	0.69	0.18
A_16_P00168784	152058726	152058785	chr1:152058726-152058785	1.65	1.57	0.28
A_16_P00168792	152062637	152062696	HCN3	0.45	-0.56	0.10
A_16_P00168793	152065375	152065430	HCN3	1.59	1.37	0.43
A_16_P15302941	152069377	152069427	HCN3	1.43	0.55	0.26
A_16_P00168810	152074440	152074499	PKLR	1.49	0.79	0.40
A_14_P114022	152077981	152078026	PKLR	1.46	-0.23	0.35
A_16_P15302986	152084439	152084498	chr1:152084439-152084498	1.02	1.36	0.27
A_16_P00168827	152088340	152088399	chr1:152088340-152088399	0.95	0.59	0.09
A_14_P109453	152093751	152093810	FDPS	1.54	1.17	0.25
A_16_P00168840	152097841	152097900	FDPS	1.72	1.50	0.28

A_14_P118865	152102091	152102146	FDPS	1.31	1.26	0.27
A_14_P110134	152110037	152110088	RUSC1	0.84	1.12	-0.03
A_14_P110595	152114155	152114214	chr1:152114155-152114214	1.09	1.13	0.24
A_16_P00168872	152120282	152120341	ASH1L	0.70	1.20	0.03
A_16_P35316535	152124725	152124784	ASH1L	0.99	0.88	0.22
A_14_P138544	152132262	152132321	ASH1L	1.05	1.15	0.22
A_16_P15303128	152143250	152143309	ASH1L	1.36	0.71	0.27
A_16_P00168913	152153470	152153529	ASH1L	1.20	0.17	0.08
A_16_P00168921	152161220	152161279	ASH1L	1.00	0.75	-0.04
A_16_P15303160	152171035	152171094	ASH1L	0.90	0.42	0.07
A_14_P131183	152178381	152178440	ASH1L	0.81	1.76	-0.11
A_16_P15303186	152185206	152185265	ASH1L	1.24	1.03	0.09
A_16_P15303196	152195943	152196002	ASH1L	0.87	1.00	-0.01
A_16_P35316673	152202135	152202194	ASH1L	1.09	0.56	0.07
A_16_P15303207	152215543	152215602	ASH1L	0.95	1.37	0.00
A_14_P108780	152221179	152221238	ASH1L	0.99	1.08	-0.13
A_16_P15303237	152235779	152235838	ASH1L	1.34	1.15	0.25
A_16_P15303273	152247127	152247186	ASH1L	1.44	0.69	0.14
A_16_P15303293	152252895	152252954	ASH1L	0.93	0.79	0.02
A_14_P125721	152265068	152265127	ASH1L	1.00	1.40	0.15
A_16_P15303343	152274032	152274091	ASH1L	0.85	1.23	-0.05
A_16_P15303357	152282610	152282669	ASH1L	1.05	1.47	0.14
A_16_P15303379	152290303	152290362	ASH1L	1.18	0.46	0.02
A_14_P132131	152303948	152304007	ASH1L	1.37	1.28	0.08
A_16_P15303416	152314507	152314566	ASH1L	0.77	1.04	-0.19
A_16_P00169068	152329283	152329342	ASH1L	1.41	0.92	0.21
A_16_P15303459	152335528	152335587	ASH1L	1.28	1.68	0.16
A_14_P104104	152346796	152346855	AK126559	1.47	1.38	0.30
A_16_P15303503	152354514	152354573	AY339211	1.15	1.10	0.17
A_16_P35316985	152365218	152365277	chr1:152365218-152365277	1.48	1.07	0.12
A_16_P00169104	152373294	152373353	chr1:152373294-152373353	1.09	0.91	0.26
A_16_P15303545	152382340	152382399	chr1:152382340-152382399	1.41	1.39	0.36
A_16_P15303553	152386803	152386862	chr1:152386803-152386862	0.76	0.80	-0.23
A_14_P112204	152396245	152396301	MSTO1	0.31	1.55	0.05
A_16_P15303582	152400371	152400430	chr1:152400371-152400430	1.65	0.93	0.23
A_16_P15303591	152429240	152429299	BC092439	0.84	0.65	0.15
A_14_P104263	152431711	152431770	chr1:152431711-152431770	1.29	0.93	-0.02
A_16_P00169131	152441303	152441362	chr1:152441303-152441362	1.60	1.20	0.23
A_14_P114709	152452977	152453036	YY1AP1	1.61	0.90	0.11
A_16_P00169154	152461289	152461348	YY1AP1	1.27	1.21	0.12
A_14_P131921	152472370	152472429	DAP3	0.69	1.51	0.24
A_14_P117601	152489294	152489353	DAP3	1.48	1.23	0.27
A_16_P35317227	152498523	152498582	DAP3	0.65	1.36	-0.35
A_16_P15303725	152504317	152504376	DAP3	1.76	1.43	0.13
A_14_P118949	152521242	152521297	DAP3	1.25	1.40	0.11
A_16_P00169202	152530779	152530837	BC070067	1.78	1.06	0.28
A_14_P100817	152538493	152538552	GON4L	1.43	-0.36	0.08
A_14_P108771	152546963	152547010	GON4L	1.06	0.47	0.13

A_16_P00169239	152563006	152563065	GON4L	1.77	1.31	0.33
A_16_P00169246	152570234	152570292	GON4L	2.11	1.09	0.33
A_14_P122547	152577959	152578018	GON4L	1.73	1.33	0.28
A_16_P35317466	152587758	152587817	GON4L	1.52	1.49	0.11
A_14_P136562	152597177	152597234	GON4L	1.48	0.38	0.15
A_16_P15303947	152605084	152605143	GON4L	1.55	1.00	0.11
A_16_P15303962	152617283	152617342	GON4L	1.48	0.87	0.06
A_16_P35317530	152628629	152628688	GON4L	1.24	0.87	-0.01
A_14_P104089	152636626	152636685	GON4L	1.54	1.59	-0.20
A_16_P00169315	152641947	152642006	AY335491	1.33	0.07	0.14
A_14_P121308	152647439	152647498	SYT11	1.64	0.84	0.21
A_16_P00169336	152655306	152655365	SYT11	1.76	1.48	0.33
A_14_P100980	152667934	152667993	AK026178	0.80	2.10	-0.03
A_16_P00169360	152674448	152674507	chr1:152674448-152674507	0.97	1.31	-0.09
A_14_P128370	152682951	152683010	RIT1	1.36	1.46	0.11
A_16_P35317681	152687540	152687599	RIT1	1.34	0.99	-0.02
A_14_P200041	152691897	152691956	RIT1	2.02	1.48	0.30
A_16_P15304134	152696527	152696586	KIAA0907	1.97	1.36	0.13
A_14_P122600	152704736	152704795	KIAA0907	0.65	1.83	0.06
A_14_P120161	152709481	152709540	KIAA0907	1.79	-0.21	0.13
A_16_P15304183	152714659	152714718	KIAA0907	1.45	0.68	0.13
A_16_P35317777	152720890	152720949	chr1:152720890-152720949	1.08	0.58	0.24
A_14_P111354	152725417	152725469	RXFP4	0.08	1.74	-0.09
A_16_P00169440	152730034	152730093	ARHGEF2	1.43	0.65	-0.02
A_16_P15304241	152737484	152737543	ARHGEF2	1.68	1.72	0.15
A_16_P15304249	152742739	152742798	ARHGEF2	-4.07	-0.16	0.01
A_14_P133916	152752929	152752988	ARHGEF2	1.36	1.77	0.26
A_14_P105474	152762755	152762814	AB014551	2.08	1.71	0.11
A_16_P00169508	152770201	152770260	AB014551	1.19	0.47	0.24
A_16_P15304366	152782576	152782635	chr1:152782576-152782635	1.80	0.81	0.07
A_14_P111500	152791922	152791981	SSR2	1.69	1.10	0.38
A_16_P00169541	152796807	152796866	SSR2	0.59	0.81	-0.17
A_16_P00169551	152804128	152804187	chr1:152804128-152804187	1.08	1.19	0.10
A_16_P00169557	152812157	152812216	chr1:152812157-152812216	1.21	1.02	0.19
A_16_P00169570	152820532	152820591	UBQLN4	1.80	0.95	0.00
A_16_P00169574	152826079	152826138	UBQLN4	0.84	1.21	-0.23
A_14_P103897	152829804	152829863	UBQLN4	1.50	0.41	0.19
A_16_P00169585	152835073	152835132	UBQLN4	1.68	0.07	0.16
A_14_P127074	152840490	152840549	MAPBPIP	1.32	1.11	0.02
A_16_P00169602	152845057	152845116	RAB25	1.76	1.17	0.12
A_14_P127546	152848350	152848409	RAB25	1.42	2.33	0.46
A_16_P00169619	152855057	152855116	chr1:152855057-152855116	1.87	0.58	0.50
A_16_P15304555	152859169	152859228	AK125482	1.95	1.56	0.50
A_16_P15304591	152869137	152869196	AK056143	1.54	1.37	0.15
A_14_P136879	152878225	152878284	AK056143	1.59	1.03	0.05
A_16_P00169683	152894845	152894904	AK056143	1.68	0.62	0.34
A_14_P132140	152900987	152901046	LMNA	1.77	1.40	0.16
A_16_P15304685	152905356	152905415	LMNA	1.50	0.47	-0.29

A_14_P128617	152915380	152915439	LMNA	0.81	1.00	0.30
A_16_P00169727	152920942	152920999	LMNA	1.44	1.74	0.07
A_16_P15304749	152929189	152929246	chr1:152929189-152929246	1.59	1.07	0.25
A_16_P00169742	152934762	152934820	AY358531	1.22	-0.74	0.35
A_16_P15304785	152939944	152940003	SEMA4A	1.57	0.61	-0.12
A_14_P107291	152945859	152945915	SEMA4A	1.78	1.09	0.25
A_16_P15304805	152950566	152950617	SEMA4A	1.99	1.58	0.29
A_16_P35318499	152965330	152965379	chr1:152965330-152965379	1.73	0.92	0.26
A_16_P15304828	152976027	152976085	chr1:152976027-152976085	1.63	0.89	0.24
A_16_P00169785	152980278	152980337	KIAA0446	1.59	0.84	0.05
A_16_P00169793	152984349	152984408	KIAA0446	1.11	-0.41	0.20
A_16_P00169800	152991470	152991529	KIAA0446	2.01	1.15	0.17
A_16_P00169811	152995543	152995602	KIAA0446	1.60	0.87	0.13
A_14_P114220	153001127	153001186	PMF1	1.77	0.45	0.09
A_16_P00169822	153008068	153008127	PMF1	1.45	1.51	-0.04
A_16_P15304926	153016345	153016404	PMF1	1.83	0.64	0.24
A_14_P100083	153019706	153019765	PMF1	1.42	1.40	0.27
A_14_P131501	153026574	153026623	PAQR6	2.43	1.54	0.65
A_16_P00169861	153033448	153033502	SMG5	2.06	1.24	0.26
A_14_P109368	153039359	153039418	SMG5	1.44	1.35	0.28
A_16_P15305020	153051020	153051079	SMG5	2.04	0.65	0.18
A_14_P117036	153057876	153057935	SMG5	1.49	1.82	0.15
A_16_P00169909	153061801	153061860	SMG5	1.06	1.01	0.08
A_14_P126203	153066461	153066520	TMEM79	0.78	0.69	0.22
A_14_P125256	153067663	153067716	TMEM79	1.40	0.80	0.16
A_16_P00169926	153071659	153071718	TMEM79	1.56	1.25	0.09
A_14_P113243	153077328	153077376	C1orf85	2.46	1.25	0.48
A_16_P15305109	153085702	153085761	chr1:153085702-153085761	1.70	1.79	0.16
A_16_P00169954	153091823	153091882	CCT3	1.54	1.54	0.18
A_14_P108302	153099304	153099363	CCT3	1.39	1.35	0.26
A_16_P15305156	153106192	153106251	CCT3	1.44	1.25	0.30
A_16_P15305170	153116491	153116550	CCT3	1.20	0.45	0.10
A_14_P200042	153120420	153120478	CCT3	1.43	1.17	0.08
A_14_P113791	153129794	153129852	C1orf182	1.35	1.00	-0.01
A_16_P35318951	153133869	153133928	chr1:153133869-153133928	1.79	1.59	0.10
A_16_P35318954	153139079	153139138	chr1:153139079-153139138	1.59	0.75	0.16
A_16_P00170019	153150675	153150734	chr1:153150675-153150734	1.82	0.98	0.37
A_14_P117615	153158528	153158587	RHBG	1.31	0.90	0.02
A_16_P00170041	153166163	153166219	RHBG	1.86	1.47	0.12
A_16_P00170053	153171937	153171996	AB007971	2.10	0.72	0.13
A_16_P35319056	153175336	153175390	AB007971	2.20	1.23	0.20
A_16_P35319068	153180041	153180087	chr1:153180041-153180087	2.07	0.69	0.42
A_16_P15305336	153187085	153187144	chr1:153187085-153187144	1.69	1.04	0.27
A_16_P00170086	153189996	153190054	C1orf61	1.80	1.38	0.32
A_14_P123928	153193559	153193603	C1orf61	1.50	2.08	-0.17
A_16_P15305368	153197241	153197300	C1orf61	1.66	0.68	0.03
A_16_P00170109	153199754	153199813	C1orf61	1.39	0.73	0.09
A_16_P15305386	153202146	153202205	C1orf61	1.52	0.91	0.10

A_16_P00170125	153206606	153206664	C1orf61	1.68	0.23	0.33
A_14_P119703	153212346	153212398	BC054045	1.38	1.52	0.09
A_16_P15305455	153221349	153221408	chr1:153221349-153221408	1.43	1.13	-0.08
A_16_P00170171	153229444	153229503	chr1:153229444-153229503	0.86	1.22	-0.26
A_16_P15305487	153233708	153233767	chr1:153233708-153233767	1.06	0.88	0.05
A_16_P35319278	153246683	153246742	MEF2D	1.20	1.25	0.29
A_14_P106403	153264534	153264593	MEF2D	1.82	1.07	0.23
A_16_P00170250	153275326	153275383	MEF2D	1.59	1.45	0.21
A_16_P00170263	153280994	153281052	MEF2D	2.14	0.91	0.27
A_16_P15305653	153287573	153287632	chr1:153287573-153287632	1.72	0.89	0.12
A_16_P00170291	153302232	153302291	chr1:153302232-153302291	1.15	-0.20	0.02
A_14_P136060	153308526	153308585	IQGAP3	1.71	0.97	0.18
A_16_P15305724	153318857	153318916	IQGAP3	0.18	0.74	-0.05
A_14_P120100	153327250	153327307	IQGAP3	1.48	1.20	0.16
A_16_P15305795	153343013	153343072	IQGAP3	2.01	1.21	0.27
A_14_P104634	153354414	153354473	IQGAP3	0.94	0.74	-0.15
A_16_P00170381	153360948	153361001	chr1:153360948-153361001	2.30	1.90	0.32
A_16_P00170394	153369769	153369828	chr1:153369769-153369828	1.51	1.11	-0.05
A_14_P124908	153376126	153376171	APOA1BP	1.29	1.01	0.33
A_14_P113561	153381918	153381977	GPATC4	1.71	0.69	-0.15
A_16_P15305912	153385908	153385967	chr1:153385908-153385967	1.95	1.48	0.19
A_16_P15305921	153390986	153391045	chr1:153390986-153391045	1.19	0.06	-0.18
A_16_P00170432	153395677	153395735	chr1:153395677-153395735	1.53	1.97	0.31
A_16_P15305944	153399174	153399233	chr1:153399174-153399233	1.89	0.45	0.22
A_14_P100389	153403383	153403442	HAPLN2	1.75	1.43	0.29
A_16_P15305970	153409399	153409458	chr1:153409399-153409458	1.78	1.85	0.10
A_16_P15305974	153413243	153413302	chr1:153413243-153413302	1.49	0.96	0.43
A_16_P15305978	153418656	153418709	chr1:153418656-153418709	1.79	1.74	0.43
A_16_P35319827	153426066	153426125	BCAN	1.76	1.00	0.14
A_14_P131260	153432150	153432206	BCAN	0.89	1.43	-0.11
A_14_P113766	153436237	153436293	BCAN	1.30	1.68	0.12
A_16_P00170496	153440271	153440329	BCAN	1.36	0.71	0.17
A_16_P00170512	153447548	153447607	chr1:153447548-153447607	1.44	0.16	0.23
A_14_P133525	153456508	153456556	NES	1.45	1.14	0.05
A_16_P00170538	153460942	153460998	chr1:153460942-153460998	2.41	1.37	0.25
A_16_P00170544	153464670	153464728	chr1:153464670-153464728	1.47	1.00	0.37
A_16_P00170554	153473477	153473535	chr1:153473477-153473535	1.25	1.27	0.07
A_14_P116196	153483487	153483534	CRABP2	0.67	4.27	-0.14
A_16_P15306172	153487031	153487090	CRABP2	1.77	1.33	0.19
A_16_P00170593	153492979	153493038	chr1:153492979-153493038	1.92	0.24	0.27
A_16_P35320069	153502405	153502464	chr1:153502405-153502464	1.71	1.27	0.16
A_14_P120697	153508659	153508714	ISG20L2	1.83	0.99	0.20
A_16_P00170620	153512389	153512448	C1orf66	1.09	0.89	0.17
A_14_P135490	153518461	153518520	C1orf66	1.31	1.21	-0.10
A_14_P105949	153522163	153522222	MRPL24	1.59	0.72	0.31
A_14_P111109	153531129	153531187	HDGF	0.95	1.48	0.33
A_16_P00170670	153536278	153536337	AL833592	1.43	-0.58	0.25
A_16_P00170678	153542865	153542924	AL833592	0.74	4.05	0.07

A_16_P00170681	153547828	153547887	AL833592	1.62	0.68	0.21
A_16_P15306351	153552836	153552895	PRCC	1.45	0.87	0.00
A_16_P00170701	153565158	153565217	PRCC	1.62	1.40	0.04
A_14_P134118	153577635	153577693	PRCC	1.11	0.79	0.28
A_16_P00170729	153583631	153583690	PRCC	1.65	1.39	0.04
A_14_P117305	153589125	153589184	SH2D2A	2.22	0.64	0.32
A_16_P00170745	153593263	153593322	SH2D2A	1.10	0.58	0.11
A_14_P200043	153599612	153599658	SH2D2A	2.55	1.02	0.49
A_16_P15306468	153604974	153605033	NTRK1	1.75	0.65	0.28
A_16_P15306474	153609809	153609868	NTRK1	1.11	0.72	0.00
A_16_P00170782	153621467	153621526	NTRK1	1.11	0.88	0.09
A_16_P15306525	153625540	153625593	NTRK1	1.48	1.55	0.22
A_16_P15306540	153629940	153629997	NTRK1	2.01	1.39	0.16
A_14_P118101	153639269	153639328	NTRK1	1.59	1.24	0.07
A_14_P131170	153644843	153644902	NTRK1	1.48	1.51	0.25
A_14_P133584	153648735	153648793	NTRK1	1.99	0.93	0.39
A_16_P35320530	153653702	153653761	NTRK1	1.27	1.18	0.04
A_14_P101526	153660147	153660206	NTRK1	1.01	1.61	-0.10
A_14_P101526	153660147	153660206	NTRK1	0.89	1.38	-0.20
A_14_P101526	153660147	153660206	NTRK1	0.94	1.72	-0.18
A_16_P00170869	153664930	153664986	chr1:153664930-153664986	1.77	0.87	0.24
A_16_P15306678	153673289	153673348	chr1:153673289-153673348	1.13	0.94	0.04
A_16_P00170893	153678532	153678586	chr1:153678532-153678586	2.03	1.72	0.20
A_16_P35320685	153694661	153694718	AK098809	1.12	0.62	0.03
A_16_P00170936	153698993	153699052	AK098809	1.86	1.43	0.30
A_14_P104300	153703922	153703981	C1orf92	1.24	1.18	0.03
A_16_P15306781	153708902	153708961	C1orf92	2.06	1.11	0.32
A_16_P15306803	153715927	153715986	chr1:153715927-153715986	1.47	1.28	0.19
A_16_P15306820	153720348	153720394	ARHGEF11	1.25	0.48	-0.08
A_16_P15306847	153728725	153728784	ARHGEF11	1.57	1.21	0.17
A_16_P35320853	153737702	153737761	ARHGEF11	1.62	1.21	0.15
A_14_P128432	153744514	153744573	ARHGEF11	1.22	0.96	0.10
A_16_P15306956	153761333	153761392	ARHGEF11	1.07	0.44	0.21
A_14_P122439	153776041	153776100	ARHGEF11	1.71	1.07	0.41
A_16_P15307022	153785370	153785429	ARHGEF11	1.80	0.54	0.13
A_16_P00171114	153792501	153792560	ARHGEF11	0.98	0.88	0.06
A_16_P15307070	153804112	153804171	ARHGEF11	1.24	0.87	0.07
A_14_P133141	153809613	153809672	ARHGEF11	0.80	1.29	0.17
A_16_P00171145	153819611	153819670	ARHGEF11	1.40	1.06	0.28
A_14_P122851	153827498	153827557	ARHGEF11	1.42	0.93	0.20
A_16_P15307150	153833333	153833392	chr1:153833333-153833392	1.16	0.83	0.11
A_16_P15307158	153842714	153842773	chr1:153842714-153842773	0.22	1.37	-0.18
A_16_P35321154	153852918	153852977	chr1:153852918-153852977	0.39	1.06	0.13
A_16_P00171189	153861573	153861632	chr1:153861573-153861632	1.37	1.19	0.26
A_16_P00171196	153867579	153867638	chr1:153867579-153867638	1.01	0.94	0.19
A_16_P00171204	153875226	153875285	FLJ16478	0.56	1.44	0.06
A_16_P15307232	153880438	153880482	FLJ16478	0.88	0.95	0.36
A_16_P15307249	153886717	153886776	chr1:153886717-153886776	1.36	0.76	0.13

A_16_P15307274	153893892	153893951	chr1:153893892-153893951	1.15	1.34	0.09
A_16_P00171250	153898774	153898833	chr1:153898774-153898833	1.02	0.26	-0.05
A_16_P15307304	153903020	153903079	chr1:153903020-153903079	1.36	1.31	0.11
A_14_P134934	153908534	153908580	L16464	1.22	1.40	0.12
A_14_P110038	153916248	153916307	ETV3	1.00	1.00	-0.05
A_14_P132906	153917935	153917994	ETV3	0.73	0.52	0.06
A_14_P110400	153919453	153919512	ETV3	0.73	0.27	-0.11
A_16_P15307375	153923379	153923438	chr1:153923379-153923438	1.12	0.98	0.18
A_16_P35321410	153933356	153933415	chr1:153933356-153933415	1.02	0.77	0.03
A_14_P115493	153949670	153949729	chr1:153949670-153949729	1.51	1.45	0.25
A_16_P15307559	153984142	153984201	chr1:153984142-153984201	1.85	0.98	0.44
A_16_P00171430	154007986	154008045	chr1:154007986-154008045	1.30	1.34	-0.18
A_16_P15307652	154043128	154043186	chr1:154043128-154043186	1.09	0.98	-0.01
A_16_P00171477	154066453	154066512	chr1:154066453-154066512	1.04	1.01	0.14
A_14_P102194	154093308	154093367	chr1:154093308-154093367	1.02	0.93	0.02
A_16_P15307780	154137750	154137809	chr1:154137750-154137809	1.30	1.47	-0.10
A_16_P00171550	154164588	154164647	chr1:154164588-154164647	0.91	0.58	0.04
A_16_P15307951	154224268	154224327	chr1:154224268-154224327	0.94	1.02	0.03
A_16_P15308009	154267049	154267108	chr1:154267049-154267108	0.79	0.40	-0.08
A_16_P00171661	154281251	154281310	chr1:154281251-154281310	0.69	0.79	-0.14
A_16_P00171673	154297084	154297143	FCRL5	0.61	0.66	-0.04
A_14_P201237	154305018	154305077	FCRL5	0.81	1.67	0.20
A_14_P136877	154305597	154305656	FCRL5	0.70	1.66	0.01
A_14_P117839	154310748	154310807	FCRL5	0.83	0.87	0.08
A_16_P35322171	154328953	154329012	FCRL5	0.98	0.55	0.10
A_14_P120324	154335245	154335299	FCRL5	0.49	0.35	-0.09
A_16_P35322195	154341405	154341464	chr1:154341405-154341464	1.00	1.12	-0.09
A_16_P15308198	154348165	154348224	chr1:154348165-154348224	1.42	0.70	-0.17
A_14_P107366	154357933	154357992	FCRL4	1.50	1.55	0.19
A_14_P119473	154361466	154361524	FCRL4	0.67	0.96	-0.02
A_14_P137420	154364133	154364192	FCRL4	0.86	0.88	-0.02
A_16_P00171773	154370765	154370824	FCRL4	0.62	1.25	-0.22
A_14_P117753	154376768	154376827	FCRL4	0.57	0.89	-0.07
A_16_P15308295	154385148	154385207	chr1:154385148-154385207	0.69	0.84	-0.09
A_16_P00171805	154401121	154401180	chr1:154401121-154401180	1.31	1.56	0.26
A_16_P35322375	154414185	154414244	chr1:154414185-154414244	0.96	1.25	-0.09
A_16_P35322421	154429345	154429404	chr1:154429345-154429404	0.78	1.04	-0.12
A_16_P15308410	154437098	154437157	chr1:154437098-154437157	0.85	0.81	0.02
A_16_P15308415	154453154	154453213	chr1:154453154-154453213	1.01	1.12	-0.09
A_14_P102186	154459554	154459613	FCRL3	0.73	1.45	-0.19
A_16_P15308448	154465577	154465636	FCRL3	0.71	0.60	-0.15
A_16_P35322501	154472876	154472935	FCRL3	1.02	0.73	0.28
A_14_P118917	154478128	154478187	FCRL3	0.78	1.72	-0.03
A_16_P15308506	154487391	154487450	chr1:154487391-154487450	0.78	0.52	-0.72
A_16_P15308526	154495504	154495563	chr1:154495504-154495563	0.42	1.09	-0.84
A_16_P00171951	154510132	154510191	chr1:154510132-154510191	0.91	1.23	-0.61
A_16_P15308565	154517644	154517703	chr1:154517644-154517703	0.26	0.81	-0.64
A_16_P15308570	154523678	154523737	chr1:154523678-154523737	0.74	1.37	-0.37

A_16_P00171971	154532458	154532517	FCRL2	0.29	0.35	0.40
A_16_P00171979	154539199	154539258	FCRL2	0.99	0.47	-0.63
A_14_P139667	154544532	154544591	FCRL2	0.63	1.01	-0.65
A_16_P35322681	154553348	154553407	FCRL2	0.82	1.00	-0.58
A_16_P00172019	154565787	154565846	chr1:154565787-154565846	0.65	0.38	-0.59
A_16_P00172029	154578929	154578988	FCRL1	1.60	1.25	-0.44
A_14_P130712	154584533	154584592	FCRL1	0.90	1.48	-0.25
A_16_P15308723	154593941	154594000	FCRL1	1.41	0.74	-0.79
A_16_P15308725	154602672	154602731	FCRL1	1.17	0.80	-0.59
A_16_P15308742	154614158	154614217	CD5L	1.24	1.63	-0.44
A_14_P116044	154618725	154618783	CD5L	0.88	0.93	-0.54
A_16_P35322852	154624361	154624420	CD5L	0.83	1.08	-0.64
A_16_P35322871	154629474	154629533	chr1:154629474-154629533	1.00	0.69	-0.55
A_16_P00172110	154635277	154635336	chr1:154635277-154635336	1.17	1.30	-0.32
A_16_P15308823	154643174	154643233	chr1:154643174-154643233	0.70	1.18	-0.64
A_16_P15308840	154649238	154649297	chr1:154649238-154649297	0.57	1.23	-0.80
A_16_P15308878	154662907	154662966	chr1:154662907-154662966	0.81	1.05	-0.34
A_16_P00172157	154676979	154677038	chr1:154676979-154677038	1.36	1.37	-0.34
A_16_P15308899	154691897	154691956	chr1:154691897-154691956	0.18	0.57	-0.43
A_14_P108814	154699522	154699581	chr1:154699522-154699581	1.15	0.69	-0.47
A_16_P15308938	154712938	154712997	AK057438	0.58	0.86	-0.63
A_16_P00172203	154727610	154727669	AK057438	0.97	0.76	-0.71
A_16_P15309003	154739450	154739509	chr1:154739450-154739509	0.81	0.15	-0.35
A_16_P15309048	154759440	154759497	chr1:154759440-154759497	1.61	1.49	-0.37
A_16_P00172270	154772754	154772813	chr1:154772754-154772813	1.21	0.36	-0.33
A_14_P136081	154783542	154783601	KIRREL	1.14	0.25	-0.52
A_14_P107173	154802759	154802818	KIRREL	1.54	0.98	-0.30
A_16_P35323303	154813219	154813278	KIRREL	0.75	0.17	-0.71
A_16_P00172369	154825265	154825324	KIRREL	0.57	0.83	-0.54
A_16_P00172377	154831698	154831757	KIRREL	0.97	0.55	-0.39
A_14_P129570	154840297	154840356	KIRREL	-0.02	1.16	-0.27
A_16_P15309315	154847268	154847327	KIRREL	1.13	1.06	-0.65
A_16_P00172430	154856154	154856213	KIRREL	1.41	1.38	-0.35
A_14_P103551	154869890	154869949	KIRREL	1.24	0.17	-0.47
A_16_P15309436	154881794	154881853	AK090554	1.58	1.25	-0.31
A_16_P15309456	154890708	154890767	chr1:154890708-154890767	1.33	0.99	-0.35
A_16_P35323586	154903613	154903672	chr1:154903613-154903672	1.19	0.97	0.23
A_16_P15309507	154913961	154914020	chr1:154913961-154914020	1.28	0.90	0.38
A_14_P129351	154935099	154935158	chr1:154935099-154935158	1.25	0.71	0.19
A_16_P15309562	154942406	154942465	chr1:154942406-154942465	0.75	1.49	0.18
A_16_P15309580	154949502	154949561	chr1:154949502-154949561	0.99	0.93	0.04
A_16_P00172578	154956894	154956953	chr1:154956894-154956953	1.02	-0.66	0.36
A_16_P35323706	154961793	154961852	chr1:154961793-154961852	0.93	0.79	0.24
A_14_P103137	154966651	154966710	CD1D	1.25	0.97	0.42
A_16_P00172607	154972699	154972758	chr1:154972699-154972758	1.32	0.68	0.39
A_16_P15309652	154977628	154977687	chr1:154977628-154977687	0.89	0.92	0.14
A_16_P15309664	154982890	154982946	AK097921	1.13	1.23	0.36
A_16_P15309674	154997391	154997450	CR603267	1.29	0.19	0.49

A_16_P00172628	155008486	155008545	CR603267	0.84	-0.10	-0.04
A_16_P15309681	155027576	155027635	CR603267	0.65	0.82	0.35
A_16_P00172635	155031399	155031458	CR603267	0.44	1.57	0.05
A_14_P127974	155037466	155037525	CD1A	1.00	1.00	0.41
A_16_P15309724	155043905	155043964	chr1:155043905-155043964	1.04	1.77	0.33
A_16_P35323838	155048641	155048700	chr1:155048641-155048700	0.40	0.79	0.01
A_16_P35323848	155055324	155055383	chr1:155055324-155055383	1.24	1.12	0.10
A_16_P15309751	155059803	155059862	chr1:155059803-155059862	0.85	1.58	0.22
A_16_P15309752	155063741	155063800	chr1:155063741-155063800	0.54	0.78	0.14
A_16_P15309754	155067659	155067718	chr1:155067659-155067718	0.98	1.19	0.25
A_14_P103654	155073202	155073253	CD1C	1.15	1.30	0.36
A_16_P15309786	155076968	155077027	chr1:155076968-155077027	1.31	1.35	0.21
A_16_P35323904	155093953	155094012	chr1:155093953-155094012	0.75	1.95	0.38
A_16_P15309790	155108721	155108780	AK130822	0.76	0.81	0.13
A_14_P137670	155113723	155113780	CD1B	1.14	0.50	0.11
A_16_P15309819	155125180	155125239	chr1:155125180-155125239	0.40	0.96	0.45
A_16_P15309820	155128475	155128522	chr1:155128475-155128522	1.20	0.72	0.30
A_14_P129272	155139399	155139456	CD1E	0.67	1.21	0.18
A_14_P201239	155139562	155139620	CR608653	1.07	0.42	0.22
A_16_P00172730	155145482	155145541	AK128347	0.76	0.52	0.36
A_16_P15309874	155152973	155153032	chr1:155152973-155153032	0.95	0.88	0.22
A_16_P15309883	155165961	155166020	chr1:155165961-155166020	-0.10	1.44	0.19
A_16_P15309897	155175914	155175973	chr1:155175914-155175973	0.90	0.45	0.24
A_16_P15309927	155184761	155184820	chr1:155184761-155184820	1.10	0.93	0.24
A_16_P15309938	155190229	155190288	chr1:155190229-155190288	0.77	0.04	0.11
A_16_P15309950	155194500	155194558	chr1:155194500-155194558	1.25	0.74	0.29
A_16_P35324084	155197751	155197810	chr1:155197751-155197810	1.04	1.03	0.12
A_16_P15309983	155203912	155203971	chr1:155203912-155203971	1.25	0.91	0.45
A_16_P15310005	155209645	155209704	chr1:155209645-155209704	1.29	1.35	0.36
A_16_P35324134	155218006	155218065	chr1:155218006-155218065	1.20	1.04	0.27
A_16_P35324157	155227871	155227930	chr1:155227871-155227930	1.19	1.31	-0.50
A_16_P15310058	155241559	155241618	chr1:155241559-155241618	0.45	0.91	-0.68
A_16_P15310073	155246017	155246076	chr1:155246017-155246076	0.40	0.43	-0.65
A_16_P35324219	155254043	155254102	chr1:155254043-155254102	1.08	0.68	-0.42
A_16_P15310106	155258414	155258473	AK057554	0.48	0.89	-0.73
A_16_P15310123	155264947	155265006	AK057554	1.01	0.10	-0.23
A_16_P35324269	155271904	155271963	AK057554	0.83	1.00	-0.57
A_16_P15310156	155277817	155277876	chr1:155277817-155277876	1.51	0.73	-0.41
A_16_P15310169	155283096	155283155	chr1:155283096-155283155	0.40	1.01	-0.85
A_16_P15310201	155301162	155301221	chr1:155301162-155301221	0.59	1.12	-0.64
A_16_P00172942	155308594	155308649	chr1:155308594-155308649	1.16	1.30	-0.18
A_16_P35324363	155320311	155320370	chr1:155320311-155320370	0.66	0.93	-0.60
A_16_P00172955	155330914	155330973	OR6Y1	1.43	0.84	-0.33
A_16_P15310264	155337761	155337820	chr1:155337761-155337820	0.21	1.32	-1.01
A_16_P15310277	155343096	155343155	chr1:155343096-155343155	0.70	0.42	-0.86
A_14_P101832	155351619	155351678	chr1:155351619-155351678	0.28	-0.71	-1.22
A_16_P15310318	155358400	155358459	chr1:155358400-155358459	0.79	0.89	-0.61
A_16_P00173015	155365088	155365147	chr1:155365088-155365147	0.75	1.81	-0.36

A_16_P35324491	155371526	155371585	chr1:155371526-155371585	0.10	0.43	-0.93
A_16_P00173024	155377949	155378008	chr1:155377949-155378008	0.55	0.23	-0.49
A_16_P00173027	155387295	155387354	chr1:155387295-155387354	0.89	1.63	-0.58
A_14_P124018	155393833	155393892	SPTA1	0.83	0.71	-0.13
A_16_P15310401	155400441	155400500	SPTA1	0.74	0.84	-0.36
A_16_P15310416	155404598	155404657	SPTA1	0.73	0.31	-0.64
A_16_P15310430	155410712	155410771	SPTA1	1.02	1.60	-0.46
A_14_P113425	155420978	155421037	SPTA1	0.34	1.10	-0.65
A_16_P15310470	155427983	155428042	SPTA1	0.84	0.45	-0.54
A_16_P15310494	155434317	155434376	SPTA1	1.31	0.74	-0.54
A_16_P35324685	155440267	155440326	SPTA1	0.41	1.22	-0.56
A_16_P00173145	155452599	155452658	SPTA1	0.78	0.55	-0.80
A_16_P35324761	155461200	155461259	SPTA1	0.81	0.62	-0.41
A_14_P133002	155466261	155466319	SPTA1	1.24	1.17	-0.56
A_16_P15310611	155470780	155470839	chr1:155470780-155470839	0.91	1.32	-0.60
A_14_P201999	155474583	155474642	chr1:155474583-155474642	0.94	1.44	-0.40
A_16_P35324823	155480826	155480885	chr1:155480826-155480885	0.61	1.53	-0.51
A_16_P15310648	155486890	155486949	chr1:155486890-155486949	0.46	0.54	-0.62
A_16_P15310660	155490258	155490317	chr1:155490258-155490317	0.57	0.64	-0.65
A_16_P15310680	155497076	155497135	chr1:155497076-155497135	0.19	0.36	-0.85
A_16_P00173241	155503259	155503318	chr1:155503259-155503318	0.60	0.17	-0.47
A_16_P15310730	155509896	155509955	chr1:155509896-155509955	0.52	0.70	-0.76
A_16_P00173265	155518597	155518656	chr1:155518597-155518656	0.77	0.60	-0.93
A_16_P35324947	155523541	155523600	chr1:155523541-155523600	1.09	1.45	-0.55
A_16_P00173282	155529363	155529422	chr1:155529363-155529422	0.96	0.52	-0.55
A_16_P15310792	155533813	155533872	chr1:155533813-155533872	1.05	1.14	-0.31
A_16_P00173300	155537856	155537915	OR6K6	1.22	1.23	-0.63
A_16_P15310823	155543559	155543618	chr1:155543559-155543618	0.89	-0.15	-0.64
A_16_P15310828	155547659	155547718	chr1:155547659-155547718	0.60	0.88	-0.45
A_16_P00173326	155554088	155554147	chr1:155554088-155554147	0.26	-0.01	-0.94
A_16_P00173337	155559839	155559898	OR6N2	0.83	0.78	-0.93
A_16_P00173344	155563149	155563208	chr1:155563149-155563208	1.33	1.44	-0.49
A_16_P15310887	155571105	155571164	chr1:155571105-155571164	0.52	1.11	-0.53
A_16_P15310891	155578924	155578976	chr1:155578924-155578976	0.49	0.97	-0.15
A_16_P15310907	155585577	155585636	chr1:155585577-155585636	0.69	0.71	-0.70
A_16_P15310952	155600759	155600818	chr1:155600759-155600818	1.44	1.25	-0.26
A_16_P15310964	155609456	155609515	chr1:155609456-155609515	0.44	1.58	-0.59
A_14_P111064	155614345	155614398	MNDA	0.50	1.04	-0.30
A_16_P35325183	155625218	155625277	MNDA	0.71	-0.31	-0.62
A_16_P35325204	155630512	155630571	MNDA	0.37	0.31	-1.22
A_16_P00173439	155646494	155646553	chr1:155646494-155646553	1.09	1.47	-0.61
A_16_P00173446	155657234	155657293	chr1:155657234-155657293	0.78	0.34	-0.58
A_14_P118356	155685017	155685076	chr1:155685017-155685076	0.61	0.63	-0.69
A_16_P15311115	155698043	155698102	chr1:155698043-155698102	0.46	0.94	-0.43
A_14_P135015	155716690	155716749	PYHIN1	0.51	0.82	-0.73
A_16_P15311179	155724801	155724860	PYHIN1	1.20	1.05	-0.40
A_16_P15311207	155743845	155743904	PYHIN1	0.82	0.86	-0.52
A_16_P35325414	155752861	155752920	PYHIN1	1.20	1.11	-0.64

A_16_P35325442	155759582	155759641	PYHIN1	0.74	1.30	-0.59
A_16_P35325451	155772924	155772983	chr1:155772924-155772983	0.51	0.52	-0.97
A_14_P137822	155782978	155783028	AK094968	1.10	1.09	0.12
A_16_P35325491	155789952	155790011	AK094968	0.89	1.25	-0.48
A_16_P35325530	155801045	155801104	IFI16	1.01	1.37	-0.10
A_16_P35325545	155815317	155815376	IFI16	0.96	0.79	-0.54
A_14_P102996	155821830	155821889	IFI16	0.92	1.05	-0.55
A_14_P102996	155821830	155821889	IFI16	1.03	1.09	-0.52
A_14_P102996	155821830	155821889	IFI16	1.03	0.97	-0.54
A_16_P00173661	155829388	155829447	IFI16	0.80	1.33	-2.39
A_14_P133897	155835853	155835912	IFI16	0.66	1.06	-0.48
A_14_P105264	155846409	155846468	AIM2	0.25	-0.11	-1.03
A_16_P15311442	155851243	155851302	AIM2	0.37	1.35	-0.62
A_16_P00173706	155856129	155856188	AIM2	0.97	1.00	-0.48
A_16_P35325696	155863646	155863705	chr1:155863646-155863705	1.07	0.88	-0.52
A_16_P15311500	155875239	155875298	chr1:155875239-155875298	1.15	0.70	-0.55
A_16_P15311555	155904168	155904227	chr1:155904168-155904227	0.63	0.54	-0.55
A_16_P15311595	155922831	155922890	chr1:155922831-155922890	1.48	0.85	-0.44
A_16_P35325853	155934212	155934271	chr1:155934212-155934271	1.28	1.37	-0.49
A_16_P15311642	155943219	155943278	chr1:155943219-155943278	1.20	0.81	-0.44
A_16_P00173832	155951514	155951573	chr1:155951514-155951573	1.24	1.14	-0.40
A_16_P35325927	155959795	155959854	IGSF4B	1.01	0.90	-0.64
A_14_P119140	155966914	155966973	IGSF4B	0.43	0.86	-0.54
A_16_P15311746	155973726	155973785	IGSF4B	1.37	1.28	-0.23
A_16_P00173889	155978478	155978537	IGSF4B	0.88	1.09	-0.59
A_14_P129627	155984008	155984067	IGSF4B	1.06	1.01	-0.08
A_14_P132596	155988363	155988417	DARC	0.31	1.63	-0.77
A_16_P15311826	155997370	155997429	chr1:155997370-155997429	1.01	0.95	-0.50
A_16_P00173937	156004802	156004861	chr1:156004802-156004861	0.67	0.71	-0.56
A_16_P15311873	156012180	156012239	chr1:156012180-156012239	1.35	1.02	-0.62
A_16_P15311896	156030948	156031007	chr1:156030948-156031007	0.74	0.72	-0.68
A_16_P00173975	156051012	156051071	chr1:156051012-156051071	1.35	0.88	-0.38
A_14_P120844	156058130	156058189	chr1:156058130-156058189	0.37	1.33	-0.35
A_16_P00173993	156064995	156065054	chr1:156064995-156065054	0.78	0.93	-0.63
A_16_P15311976	156071506	156071565	chr1:156071506-156071565	0.97	1.64	-0.42
A_16_P15311987	156075425	156075484	FCER1A	0.52	0.88	-0.70
A_14_P133250	156085141	156085200	FCER1A	0.90	0.81	-0.62
A_16_P15312027	156089087	156089146	FCER1A	1.04	1.08	-0.29
A_16_P35326290	156096463	156096522	chr1:156096463-156096522	1.04	0.51	-0.36
A_16_P15312072	156104594	156104653	chr1:156104594-156104653	0.42	0.79	-0.58
A_16_P00174072	156120866	156120925	chr1:156120866-156120925	0.59	0.77	-0.64
A_16_P15312124	156145177	156145236	BC038194	0.55	1.06	-0.39
A_16_P00174124	156156511	156156570	BC038194	0.60	0.74	-0.56
A_16_P00174127	156178180	156178237	BC038194	1.23	1.56	-0.36
A_14_P138414	156187938	156187997	BC038194	1.08	0.64	-0.58
A_16_P35326434	156205977	156206036	BC038194	0.97	0.79	-0.39
A_16_P15312199	156211136	156211195	BC038194	0.59	0.60	-0.61
A_16_P15312227	156218970	156219029	BC038194	0.81	0.81	-0.67

A_14_P130266	156223043	156223094	OR10J1	1.84	0.99	-0.34
A_16_P35326502	156228164	156228223	BC038194	0.81	1.09	-0.38
A_16_P15312265	156242627	156242686	BC038194	0.83	1.60	-0.42
A_16_P15312282	156251615	156251674	BC038194	0.99	0.80	-0.42
A_16_P15312310	156262750	156262809	chr1:156262750-156262809	1.52	1.95	-0.35
A_16_P35326613	156293807	156293866	chr1:156293807-156293866	0.49	1.20	-0.62
A_16_P35326614	156301814	156301873	chr1:156301814-156301873	0.25	1.22	-0.69
A_16_P15312381	156313212	156313271	chr1:156313212-156313271	0.52	0.61	-0.56
A_16_P35326638	156318862	156318921	chr1:156318862-156318921	0.43	1.46	-0.47
A_16_P15312405	156328483	156328542	chr1:156328483-156328542	1.34	1.69	-0.89
A_16_P15312419	156334478	156334537	chr1:156334478-156334537	1.03	0.73	-0.27
A_16_P35326686	156344464	156344523	chr1:156344464-156344523	0.79	0.72	-0.33
A_16_P15312469	156361524	156361583	chr1:156361524-156361583	0.79	1.40	-0.40
A_14_P201988	156365328	156365387	chr1:156365328-156365387	0.61	0.89	-0.67
A_14_P200044	156371167	156371226	APCS	0.88	1.69	-0.38
A_16_P15312520	156376414	156376473	chr1:156376414-156376473	0.36	0.93	-0.74
A_16_P15312535	156380518	156380577	chr1:156380518-156380577	1.36	0.68	-0.45
A_16_P00174350	156385994	156386053	chr1:156385994-156386053	1.20	0.96	-0.36
A_16_P15312563	156395434	156395493	chr1:156395434-156395493	0.78	0.91	-0.24
A_16_P15312582	156408790	156408849	chr1:156408790-156408849	1.17	1.20	-0.37
A_16_P00174384	156425071	156425130	chr1:156425071-156425130	1.12	0.74	-0.29
A_16_P00174415	156460966	156461025	chr1:156460966-156461025	1.06	0.55	-0.32
A_16_P15312716	156473358	156473417	chr1:156473358-156473417	0.90	1.09	-0.46
A_16_P15312735	156479676	156479735	chr1:156479676-156479735	0.85	1.27	-0.39
A_16_P00174463	156486124	156486182	chr1:156486124-156486182	1.60	1.40	-0.25
A_14_P134453	156495638	156495697	CRP	1.89	0.68	-0.20
A_14_P202503	156495668	156495727	CRP	1.60	1.39	-0.18
A_14_P134745	156497370	156497422	CRP	1.13	0.92	0.48
A_16_P35327049	156504203	156504262	chr1:156504203-156504262	1.38	1.84	-0.27
A_16_P15312818	156510826	156510885	chr1:156510826-156510885	1.61	1.50	-0.33
A_14_P100050	156535732	156535791	chr1:156535732-156535791	-0.39	1.59	0.05
A_16_P00174541	156548418	156548477	chr1:156548418-156548477	1.29	1.16	-0.33
A_16_P15312896	156555744	156555803	chr1:156555744-156555803	0.83	1.39	-0.24
A_14_P120630	156565153	156565202	DUSP23	1.10	0.80	-0.18
A_16_P15312944	156569473	156569532	chr1:156569473-156569532	1.30	0.96	-0.26
A_16_P35327216	156579067	156579126	chr1:156579067-156579126	1.67	0.92	-0.32
A_16_P15312979	156583660	156583719	AK131201	1.17	1.18	-0.43
A_16_P00174610	156589934	156589993	FCRL6	0.83	0.87	-0.19
A_16_P00174618	156593393	156593452	FCRL6	1.15	1.05	-0.38
A_16_P35327278	156597191	156597250	FCRL6	1.25	1.20	-0.33
A_16_P00174637	156603241	156603300	chr1:156603241-156603300	1.05	0.65	-0.32
A_16_P00174645	156606525	156606580	chr1:156606525-156606580	1.47	1.03	-0.13
A_14_P103440	156610594	156610653	SLAMF8	1.03	1.66	-0.30
A_16_P15313070	156614730	156614789	SLAMF8	1.25	1.74	-0.23
A_16_P00174673	156620085	156620144	SLAMF8	1.57	0.89	-0.24
A_16_P15313100	156624464	156624508	AK096506	0.98	0.57	0.17
A_16_P15313109	156629035	156629093	AK096506	1.40	1.19	-0.20
A_16_P35327400	156636183	156636242	AK096506	0.73	0.35	-0.46

A_16_P00174707	156641347	156641406	LOC391123	1.13	1.85	-0.08
A_16_P15313171	156647474	156647533	chr1:156647474-156647533	1.34	1.12	-0.22
A_14_P126931	156655250	156655302	CCDC19	1.25	1.76	-0.14
A_16_P15313230	156665132	156665191	CCDC19	0.80	1.35	-0.53
A_14_P107443	156670812	156670865	CCDC19	0.76	1.12	-0.14
A_16_P15313275	156680502	156680561	CCDC19	0.73	-0.15	-0.42
A_16_P00174803	156691735	156691794	CR604695	1.61	0.21	-0.11
A_16_P35327610	156699183	156699242	CR604695	1.50	0.77	-0.02
A_14_P137054	156706427	156706472	TAGLN2	1.12	0.44	-0.16
A_16_P00174834	156709878	156709937	IGSF9	0.65	1.32	-0.32
A_14_P135664	156715120	156715171	IGSF9	1.38	1.29	-0.48
A_16_P00174861	156722904	156722963	IGSF9	0.99	0.32	0.00
A_16_P15313409	156727647	156727705	IGSF9	1.56	1.60	-0.15
A_16_P15313420	156730685	156730744	chr1:156730685-156730744	0.71	0.60	-0.43
A_14_P100200	156735779	156735838	SLAMF9	1.18	1.02	-0.30
A_14_P116050	156737077	156737124	AY034613	1.70	0.38	-0.15
A_16_P15313452	156741131	156741190	chr1:156741131-156741190	1.49	-0.02	-0.34
A_16_P00174908	156747801	156747860	AF088076	1.56	1.41	-0.24
A_16_P35327843	156762264	156762323	chr1:156762264-156762323	0.74	1.12	-0.41
A_14_P108642	156779304	156779363	chr1:156779304-156779363	1.29	1.54	-0.41
A_16_P15313586	156792513	156792572	chr1:156792513-156792572	0.94	-0.09	-0.42
A_16_P15313600	156799397	156799456	chr1:156799397-156799456	1.42	0.82	-0.41
A_16_P15313613	156804533	156804592	chr1:156804533-156804592	1.05	1.48	-0.47
A_16_P15313626	156808967	156809026	BC039426	1.24	1.28	-0.41
A_14_P119545	156812968	156813027	PIGM	0.85	1.49	-0.20
A_16_P00175012	156817431	156817490	chr1:156817431-156817490	1.67	1.79	-0.38
A_14_P113825	156822392	156822451	KCNJ10	1.17	1.52	-0.16
A_16_P15313695	156829822	156829881	KCNJ10	1.21	0.95	-0.26
A_16_P00175049	156834825	156834884	KCNJ10	1.45	1.19	-0.28
A_16_P15313737	156843179	156843238	KCNJ10	0.77	0.78	-0.17
A_16_P00175082	156850306	156850365	KCNJ10	1.27	0.88	-0.40
A_16_P15313786	156859418	156859477	chr1:156859418-156859477	0.98	0.89	-0.19
A_14_P138828	156864814	156864871	KCNJ9	1.55	0.77	-0.29
A_16_P15313814	156868465	156868516	KCNJ9	0.94	0.70	-0.10
A_14_P137800	156874678	156874724	IGSF8	0.94	0.63	-0.10
A_16_P35328201	156882550	156882609	chr1:156882550-156882609	1.22	0.31	-0.32
A_16_P15313888	156890001	156890058	chr1:156890001-156890058	0.93	1.14	-0.40
A_16_P15313909	156897704	156897763	Y07494	1.10	1.18	-0.34
A_16_P35328262	156902182	156902241	ATP1A2	1.30	1.78	-0.25
A_16_P00175197	156910772	156910831	ATP1A2	1.02	1.72	-0.07
A_16_P00175205	156916504	156916563	ATP1A2	1.66	0.83	-0.27
A_14_P117927	156921294	156921353	ATP1A2	0.72	1.04	-0.27
A_16_P35328362	156928572	156928631	chr1:156928572-156928631	1.43	1.53	-0.26
A_14_P136770	156934962	156935021	ATP1A4	1.45	1.27	-0.22
A_16_P00175246	156944099	156944158	ATP1A4	1.29	1.43	-0.43
A_16_P15314054	156949785	156949844	ATP1A4	0.66	0.49	-0.32
A_16_P15314098	156962460	156962519	ATP1A4	1.00	0.78	-0.69
A_14_P114704	156965866	156965924	ATP1A4	1.32	1.68	-0.22

A_16_P00175290	156969618	156969677	ATP1A4	1.01	1.24	-0.08
A_16_P00175300	156973757	156973815	CASQ1	1.04	-0.59	-0.46
A_14_P100750	156977921	156977976	CASQ1	1.30	1.53	-0.15
A_16_P00175318	156984468	156984527	CASQ1	0.86	0.79	-0.42
A_14_P136774	156990900	156990959	PEA15	1.62	1.39	-0.24
A_16_P35328602	156999499	156999558	WDR42A	1.18	1.59	-0.19
A_16_P15314229	157003138	157003197	WDR42A	1.10	0.02	-0.34
A_16_P35328659	157014258	157014317	WDR42A	1.21	1.15	-0.42
A_14_P100040	157027336	157027395	WDR42A	0.49	1.35	-0.19
A_16_P00175422	157038049	157038108	WDR42A	0.96	1.29	-0.38
A_14_P106757	157044186	157044245	WDR42A	1.12	1.64	-0.39
A_16_P15314369	157051062	157051121	chr1:157051062-157051121	0.72	0.23	-0.33
A_14_P126604	157063009	157063064	PEX19	1.25	1.15	-0.19
A_16_P00175462	157067614	157067673	PEX19	1.34	1.05	-0.37
A_16_P00175471	157072826	157072885	COPA	1.00	1.26	-0.42
A_16_P00175490	157080419	157080478	COPA	0.81	1.70	-0.47
A_14_P111715	157088418	157088466	COPA	1.71	1.40	-0.08
A_16_P00175508	157093070	157093129	COPA	1.16	1.64	-0.37
A_14_P132433	157108413	157108464	COPA	0.90	1.13	-0.07
A_16_P15314505	157113962	157114021	COPA	0.75	1.34	-0.43
A_16_P35328957	157122958	157123017	COPA	1.07	1.31	-0.38
A_14_P106717	157127111	157127170	NCSTN	1.40	1.33	-0.11
A_14_P101608	157127321	157127378	NCSTN	0.86	1.24	-0.50
A_16_P15314553	157131976	157132035	NCSTN	1.30	1.30	-0.32
A_16_P15314573	157137306	157137365	NCSTN	1.10	1.34	-0.40
A_16_P00175588	157143417	157143476	chr1:157143417-157143476	1.05	0.76	-0.38
A_16_P35329054	157146589	157146648	chr1:157146589-157146648	1.62	2.03	-0.08
A_14_P123209	157153399	157153443	NHLH1	0.89	1.16	0.17
A_16_P00175616	157160213	157160272	chr1:157160213-157160272	1.19	1.90	-0.28
A_16_P15314658	157165595	157165654	chr1:157165595-157165654	1.56	1.25	-0.66
A_14_P129959	157177917	157177976	chr1:157177917-157177976	1.41	1.92	-0.15
A_16_P35329154	157189966	157190025	VANGL2	1.19	0.44	-0.09
A_16_P00175662	157195597	157195656	VANGL2	0.92	0.63	-0.54
A_16_P35329208	157205300	157205359	VANGL2	1.17	1.41	-0.43
A_16_P35329224	157210261	157210320	VANGL2	0.93	0.55	-0.10
A_16_P15314814	157228626	157228685	chr1:157228626-157228685	1.10	0.84	-0.39
A_16_P00175741	157243417	157243475	chr1:157243417-157243475	1.48	1.07	-0.23
A_16_P15314866	157254146	157254205	chr1:157254146-157254205	1.04	1.59	-0.42
A_16_P00175754	157265154	157265213	chr1:157265154-157265213	0.84	0.83	-0.12
A_14_P130512	157271852	157271905	SLAMF6	1.34	0.84	-0.01
A_14_P125769	157277820	157277879	SLAMF6	1.36	1.52	-0.09
A_16_P15314914	157284218	157284277	SLAMF6	1.09	1.25	-0.69
A_14_P138911	157300891	157300950	SLAMF6	0.62	-0.44	-0.08
A_16_P15314937	157313235	157313294	chr1:157313235-157313294	0.81	0.80	-0.44
A_16_P15314939	157319654	157319713	chr1:157319654-157319713	0.66	0.69	-0.52
A_16_P35329430	157328075	157328134	chr1:157328075-157328134	1.12	0.22	-0.37
A_14_P122883	157336252	157336310	CD84	0.95	0.77	-0.48
A_16_P35329490	157352275	157352334	CD84	1.30	0.54	-0.41

A_14_P138687	157361836	157361895	CD84	0.84	0.56	-0.28
A_16_P00175865	157371462	157371521	chr1:157371462-157371521	1.18	1.09	-0.17
A_16_P15315069	157383937	157383996	chr1:157383937-157383996	1.16	1.35	-0.25
A_14_P129334	157393416	157393474	SLAMF1	0.20	-0.36	-0.22
A_16_P15315107	157402582	157402641	SLAMF1	1.84	1.17	-0.21
A_16_P15315126	157408073	157408132	SLAMF1	0.91	1.24	-0.09
A_16_P35329651	157424057	157424116	SLAMF1	0.88	1.16	-0.43
A_14_P137324	157429972	157430027	BC012602	0.92	0.77	-0.33
A_16_P35329703	157446463	157446522	chr1:157446463-157446522	0.63	0.98	-0.47
A_16_P35329730	157457777	157457836	chr1:157457777-157457836	1.27	0.46	-0.31
A_14_P136511	157466860	157466919	CD48	0.68	-0.47	-0.52
A_16_P35329769	157469290	157469345	CD48	0.92	0.51	-0.32
A_16_P00175998	157487772	157487831	CD48	0.04	1.53	-0.38
A_14_P130805	157489641	157489700	CD48	1.20	1.68	-0.40
A_16_P15315320	157505345	157505404	chr1:157505345-157505404	1.35	0.99	-0.13
A_16_P00176022	157510074	157510133	chr1:157510074-157510133	1.44	1.27	-0.33
A_16_P00176026	157517168	157517227	chr1:157517168-157517227	0.56	0.84	-0.60
A_16_P15315349	157522719	157522778	SLAMF7	1.08	0.85	-0.27
A_16_P15315364	157527728	157527787	SLAMF7	0.77	1.34	-0.50
A_14_P118540	157532269	157532328	SLAMF7	1.18	-0.08	-0.42
A_16_P15315391	157536330	157536389	SLAMF7	1.42	0.81	-0.22
A_16_P15315401	157540807	157540866	chr1:157540807-157540866	0.83	0.83	-0.38
A_16_P35329896	157550406	157550465	chr1:157550406-157550465	1.18	1.26	-0.29
A_16_P15315425	157557058	157557117	AK097200	1.11	1.68	0.08
A_14_P126727	157564589	157564648	chr1:157564589-157564648	1.71	1.20	-0.09
A_16_P35329954	157572795	157572854	chr1:157572795-157572854	1.07	0.91	-0.22
A_16_P15315486	157580520	157580579	LY9	1.28	0.96	-0.39
A_16_P15315500	157585372	157585426	LY9	0.39	1.13	-0.13
A_16_P15315502	157591760	157591819	LY9	0.76	1.13	-0.56
A_16_P15315513	157598836	157598895	LY9	1.85	1.30	-0.13
A_14_P101269	157610623	157610682	LY9	0.93	-0.61	-0.45
A_16_P15315560	157616108	157616167	CD244	1.93	1.55	-0.22
A_14_P108616	157624332	157624384	CD244	1.78	1.68	-0.35
A_16_P15315599	157630850	157630907	CD244	1.44	0.31	-0.17
A_14_P137877	157639041	157639100	CD244	0.94	1.29	-0.37
A_16_P15315619	157644158	157644204	CD244	1.07	0.87	0.08
A_16_P35330144	157649521	157649580	chr1:157649521-157649580	1.77	1.12	-0.30
A_14_P132417	157661583	157661642	ITLN1	1.42	1.20	-0.31
A_16_P35330185	157666359	157666418	ITLN1	1.81	1.01	-0.08
A_16_P35330203	157673410	157673469	AK123144	1.59	1.47	-0.44
A_16_P00176228	157684183	157684242	AK123144	1.41	1.22	-0.35
A_16_P15315729	157695344	157695403	AK123144	0.99	1.14	-0.26
A_16_P15315731	157715206	157715265	AK123144	1.73	0.34	-0.55
A_16_P35330249	157725041	157725100	AK123144	1.43	0.42	-0.22
A_14_P138580	157730768	157730821	ITLN2	1.93	1.31	-0.16
A_16_P15315773	157740597	157740656	AK123144	1.47	1.22	-0.14
A_16_P15315780	157744494	157744553	AK123144	2.19	1.58	-0.09
A_16_P15315790	157750752	157750811	AK123144	1.38	1.00	-0.31

A_16_P15315796	157755845	157755904	AK123144	1.60	1.49	-0.29
A_14_P119407	157761912	157761971	AK123144	1.54	0.86	-0.29
A_16_P15315812	157769567	157769626	AK123144	1.60	1.11	-0.14
A_14_P125771	157779753	157779812	F11R	1.08	0.80	-0.13
A_16_P15315847	157784355	157784414	F11R	1.18	1.37	-0.10
A_16_P15315857	157793455	157793514	F11R	1.32	0.24	-0.39
A_16_P15315876	157803185	157803244	F11R	2.02	0.84	-0.14
A_16_P15315884	157807668	157807727	F11R	1.25	1.39	-0.39
A_14_P105237	157815324	157815383	F11R	1.07	-0.60	-0.41
A_16_P15315897	157819203	157819262	F11R	0.94	1.28	-0.33
A_16_P15315909	157823177	157823236	USF1	1.35	1.17	-0.39
A_14_P110659	157827760	157827813	USF1	1.23	0.06	-0.48
A_16_P15315951	157836376	157836435	ARHGAP30	1.79	0.91	-0.13
A_14_P103285	157845066	157845125	ARHGAP30	1.68	1.36	-0.33
A_16_P15315977	157850625	157850684	ARHGAP30	1.47	0.60	-0.30
A_14_P136786	157854498	157854555	AK123144	1.96	1.01	-0.16
A_14_P138232	157859000	157859059	PVRL4	1.50	1.11	-0.25
A_16_P00176392	157862323	157862369	PVRL4	1.52	0.45	0.02
A_16_P35330580	157865784	157865843	PVRL4	1.77	1.02	-0.28
A_16_P15316040	157871153	157871212	PVRL4	1.96	1.44	-0.14
A_16_P15316053	157874540	157874599	AK123144	1.67	0.42	-0.26
A_16_P00176430	157879078	157879137	AK123144	1.78	0.63	-0.40
A_14_P127353	157882386	157882435	KARCA1	1.14	1.03	0.18
A_16_P00176447	157886581	157886640	PFDN2	1.46	0.74	-0.24
A_14_P103751	157893423	157893482	PFDN2	1.13	1.17	-0.53
A_16_P15316115	157897752	157897811	PFDN2	1.23	0.26	-0.18
A_16_P15316125	157901934	157901992	NIT1	1.72	1.28	-0.17
A_14_P123090	157907161	157907210	DEDD	1.59	0.95	-0.24
A_16_P00176487	157913496	157913555	DEDD	1.96	1.13	-0.24
A_16_P00176494	157918083	157918142	chr1:157918083-157918142	1.31	0.87	-0.03
A_16_P00176501	157921854	157921913	chr1:157921854-157921913	2.00	1.23	-0.29
A_16_P00176506	157926386	157926445	chr1:157926386-157926445	0.93	0.80	-0.17
A_16_P35330775	157930005	157930064	chr1:157930005-157930064	1.11	0.70	-0.19
A_14_P114523	157936701	157936760	UFC1	1.78	0.23	-0.36
A_14_P118597	157946403	157946453	USP21	1.85	1.40	-0.01
A_14_P121393	157953690	157953747	PPOX	1.70	1.63	-0.20
A_14_P111122	157954424	157954481	B4GALT3	1.81	0.56	-0.24
A_16_P15316289	157959208	157959267	B4GALT3	1.39	0.40	-0.12
A_16_P35330911	157964179	157964238	chr1:157964179-157964238	1.82	0.18	-0.28
A_16_P15316306	157968440	157968499	chr1:157968440-157968499	1.51	-0.07	-0.05
A_16_P00176576	157971518	157971573	chr1:157971518-157971573	1.55	1.50	-0.13
A_16_P15316327	157975110	157975165	ADAMTS4	1.95	0.90	-0.25
A_16_P15316335	157978138	157978195	ADAMTS4	1.35	1.66	-0.16
A_14_P122881	157981603	157981649	ADAMTS4	1.92	1.09	0.14
A_16_P15316370	157989087	157989146	NDUFS2	1.64	1.42	-0.18
A_14_P118933	157992317	157992370	NDUFS2	1.28	1.22	0.00
A_14_P128844	157998181	157998230	FCER1G	1.50	0.05	-0.01
A_16_P00176637	158001533	158001592	FCER1G	1.58	0.53	0.11

A_14_P103272	158005361	158005417	APOA2	1.48	1.35	0.29
A_14_P118993	158011746	158011804	TOMM40L	1.90	1.07	-0.25
A_14_P200045	158012873	158012928	TOMM40L	1.79	1.65	0.02
A_14_P135807	158014032	158014085	NR1I3	1.78	1.65	-0.22
A_16_P15316458	158017303	158017362	NR1I3	0.54	0.32	-0.44
A_16_P15316466	158024044	158024102	BX537951	1.77	1.41	-0.15
A_16_P15316474	158028823	158028882	AK127099	1.21	0.31	-0.46
A_16_P15316485	158032840	158032899	AK127099	1.57	1.55	0.22
A_16_P35331161	158043021	158043080	BC028905	0.96	0.31	-0.36
A_16_P00176704	158051249	158051308	BC028905	1.66	1.06	-0.21
A_14_P124808	158066898	158066957	BC028905	0.89	2.04	0.12
A_16_P35331234	158076532	158076591	chr1:158076532-158076591	1.25	0.82	-0.47
A_16_P15316567	158080538	158080597	chr1:158080538-158080597	1.30	1.30	-0.44
A_14_P134814	158088248	158088293	MPZ	1.93	0.10	-0.01
A_16_P00176764	158097918	158097977	SDHC	1.53	0.84	-0.39
A_14_P110882	158104138	158104197	SDHC	1.00	1.16	-0.43
A_14_P121601	158111315	158111368	SDHC	1.19	1.02	0.15
A_16_P15316658	158123386	158123445	SDHC	1.49	1.46	-0.25
A_16_P15316674	158129102	158129161	SDHC	0.28	0.96	-0.56
A_16_P15316684	158133466	158133525	SDHC	1.23	1.27	-0.06
A_16_P15316698	158138945	158139004	SDHC	0.93	0.81	-0.45
A_14_P121105	158144970	158145029	SDHC	1.32	2.23	-0.15
A_16_P35331419	158150653	158150712	C1orf192	1.73	1.04	-0.15
A_16_P15316749	158157025	158157084	chr1:158157025-158157084	1.13	1.03	-0.33
A_16_P00176840	158161633	158161682	chr1:158161633-158161682	1.32	1.51	-0.36
A_14_P113667	158168691	158168750	chr1:158168691-158168750	1.50	0.05	-0.16
A_16_P15316795	158173886	158173943	chr1:158173886-158173943	1.01	1.60	-0.39
A_16_P15316802	158179915	158179974	chr1:158179915-158179974	0.72	0.91	-0.56
A_16_P15316815	158186153	158186212	chr1:158186153-158186212	1.38	1.10	-0.14
A_16_P00176866	158194488	158194547	chr1:158194488-158194547	1.05	1.00	-0.51
A_16_P15316833	158201943	158202002	chr1:158201943-158202002	1.27	0.36	-0.18
A_16_P15316849	158211115	158211174	chr1:158211115-158211174	1.59	1.27	-0.26
A_16_P00176887	158222895	158222939	chr1:158222895-158222939	1.28	0.40	0.15
A_16_P00176889	158255568	158255612	chr1:158255568-158255612	0.45	0.90	0.06
A_16_P00176893	158265365	158265424	chr1:158265365-158265424	1.33	0.80	-0.23
A_16_P00176893	158265365	158265424	chr1:158265365-158265424	1.33	1.06	-0.21
A_16_P00176893	158265365	158265424	chr1:158265365-158265424	1.33	0.75	-0.23
A_16_P00176894	158271485	158271529	chr1:158271485-158271529	1.28	1.15	-0.27
A_16_P00176909	158283824	158283883	chr1:158283824-158283883	1.03	0.94	-0.34
A_16_P00176917	158288151	158288210	chr1:158288151-158288210	0.25	0.33	-0.14
A_14_P138859	158292447	158292506	FCGR2A	0.65	1.00	-0.10
A_14_P110141	158296040	158296099	FCGR2A	1.45	0.61	-0.28
A_14_P123651	158296770	158296814	FCGR2A	1.59	0.58	-0.25
A_16_P00176938	158305161	158305220	CR624955	1.38	0.77	-0.37
A_14_P107820	158309145	158309195	HSPA6	1.41	1.20	-0.20
A_16_P15317086	158314033	158314088	chr1:158314033-158314088	1.06	1.40	-0.10
A_16_P15317088	158314837	158314890	chr1:158314837-158314890	0.63	0.95	-0.27
A_16_P15317094	158319014	158319071	chr1:158319014-158319071	1.40	0.48	-0.32

A_16_P00176967	158325153	158325212	FCGR3A	1.24	1.23	-0.30
A_14_P122986	158332696	158332749	FCGR3A	1.35	0.88	-0.38
A_16_P35331852	158337876	158337935	chr1:158337876-158337935	1.13	1.32	-0.33
A_16_P00176997	158341848	158341907	chr1:158341848-158341907	0.47	0.76	-0.52
A_16_P35331882	158350539	158350598	chr1:158350539-158350598	0.87	-0.12	-0.70
A_16_P00177012	158355900	158355958	chr1:158355900-158355958	1.33	0.77	-0.13
A_16_P15317205	158361010	158361069	chr1:158361010-158361069	1.08	0.99	-0.64
A_16_P00177029	158365270	158365328	FCGR2B	1.32	1.34	-0.17
A_14_P109934	158372551	158372610	FCGR2B	1.83	1.29	-0.09
A_16_P00177047	158378189	158378248	FCGR2B	1.36	1.18	-0.19
A_16_P15317263	158382079	158382138	chr1:158382079-158382138	0.91	0.82	0.00
A_16_P15317281	158392593	158392652	chr1:158392593-158392652	1.08	1.29	-0.39
A_16_P00177067	158405952	158406011	chr1:158405952-158406011	0.79	1.44	-0.44
A_14_P127243	158408551	158408609	FCRLM1	1.43	0.92	-0.29
A_14_P137646	158408640	158408694	FCRLM1	0.88	0.90	-0.01
A_16_P35332033	158416185	158416244	chr1:158416185-158416244	1.03	0.41	-0.40
A_16_P00177099	158422808	158422867	chr1:158422808-158422867	1.13	1.24	-0.40
A_16_P35332067	158426019	158426078	FCRLM2	1.28	1.33	-0.27
A_14_P113624	158429347	158429405	FCRLM2	0.85	1.55	-0.24
A_16_P35332095	158433313	158433372	chr1:158433313-158433372	1.09	0.50	-0.66
A_16_P00177126	158438147	158438206	chr1:158438147-158438206	1.10	0.79	-0.33
A_16_P15317405	158442355	158442414	chr1:158442355-158442414	1.01	0.79	-0.44
A_16_P15317411	158446792	158446841	chr1:158446792-158446841	1.24	1.28	0.06
A_14_P117685	158453867	158453926	DUSP12	0.63	1.16	-0.41
A_16_P35332164	158461910	158461969	chr1:158461910-158461969	1.21	1.33	-0.30
A_16_P15317446	158466990	158467049	chr1:158466990-158467049	1.00	1.05	-0.28
A_16_P35332193	158479651	158479710	ATF6	0.49	1.07	-0.73
A_16_P15317486	158485395	158485454	ATF6	1.41	0.72	-0.25
A_14_P101293	158493555	158493614	ATF6	0.89	0.78	-0.33
A_16_P15317524	158501233	158501292	ATF6	0.69	0.60	-0.39
A_16_P00177215	158508070	158508129	ATF6	1.32	1.15	-0.22
A_16_P35332289	158514957	158515016	ATF6	0.68	1.19	-0.46
A_16_P35332315	158521612	158521671	ATF6	1.17	1.35	-0.37
A_16_P15317601	158528772	158528831	ATF6	0.71	0.83	-0.27
A_16_P15317606	158536985	158537044	ATF6	0.47	0.28	-0.32
A_14_P103158	158547870	158547929	ATF6	0.56	0.49	-0.31
A_16_P15317667	158558813	158558872	ATF6	0.74	1.34	-0.37
A_16_P35332425	158567949	158568008	ATF6	0.15	1.02	-0.21
A_16_P15317723	158575724	158575783	ATF6	0.50	1.27	-0.31
A_16_P35332459	158583600	158583659	ATF6	0.56	1.29	-0.48
A_14_P107548	158592897	158592943	ATF6	0.81	0.58	0.13
A_16_P00177364	158600528	158600587	ATF6	0.40	0.31	-0.49
A_16_P15317809	158608072	158608131	ATF6	0.46	0.91	-0.65
A_14_P112649	158613763	158613821	ATF6	0.30	-0.28	-0.27
A_16_P35332586	158624841	158624900	ATF6	1.16	1.20	-0.44
A_16_P35332616	158636182	158636241	ATF6	0.84	0.95	-0.30
A_16_P00177425	158642719	158642778	ATF6	0.48	1.09	-0.50
A_16_P15317915	158650103	158650162	ATF6	0.91	0.44	-0.30

A_14_P139281	158660382	158660441	ATF6	0.03	0.68	-0.45
A_16_P15317973	158667176	158667235	chr1:158667176-158667235	0.67	0.49	-0.40
A_16_P00177495	158676220	158676279	chr1:158676220-158676279	1.52	1.65	-0.24
A_16_P35332766	158684673	158684732	OLFML2B	1.02	1.92	-0.34
A_16_P00177532	158699084	158699143	OLFML2B	1.41	1.08	-0.26
A_16_P00177551	158707862	158707921	OLFML2B	0.71	1.03	-0.34
A_16_P15318129	158718775	158718834	OLFML2B	0.62	1.53	-0.60
A_14_P129968	158736756	158736815	chr1:158736756-158736815	0.85	0.55	-0.11
A_16_P15318207	158747329	158747388	chr1:158747329-158747388	1.70	1.35	-0.28
A_16_P00177643	158758691	158758750	chr1:158758691-158758750	1.74	2.38	0.00
A_16_P15318274	158768771	158768830	chr1:158768771-158768830	1.21	1.13	-0.42
A_16_P00177671	158774419	158774478	NOS1AP	1.65	1.61	-0.06
A_14_P131151	158785146	158785205	NOS1AP	0.96	0.39	-0.05
A_16_P00177703	158795387	158795446	NOS1AP	1.34	1.19	-0.33
A_16_P15318362	158811956	158812015	NOS1AP	1.06	0.39	-0.34
A_16_P00177723	158817285	158817344	NOS1AP	0.95	0.49	-0.02
A_16_P15318412	158832416	158832475	NOS1AP	0.82	1.13	-0.41
A_16_P00177757	158838649	158838708	NOS1AP	1.31	1.25	-0.40
A_16_P00177771	158846387	158846446	NOS1AP	1.49	1.02	-0.24
A_16_P15318479	158855336	158855395	NOS1AP	0.85	1.00	-0.32
A_16_P35333240	158860816	158860875	NOS1AP	0.77	1.55	-0.25
A_16_P15318517	158868657	158868716	NOS1AP	0.99	0.71	-0.33
A_16_P00177818	158880275	158880334	NOS1AP	1.51	0.96	-0.24
A_16_P15318566	158890879	158890938	NOS1AP	0.75	1.15	-0.52
A_16_P35333340	158900061	158900120	NOS1AP	0.98	0.88	-0.17
A_14_P100664	158907101	158907160	NOS1AP	1.06	0.87	-0.35
A_16_P00177873	158913733	158913792	NOS1AP	1.71	0.30	-0.16
A_16_P15318666	158921180	158921239	NOS1AP	1.07	0.80	-0.41
A_16_P15318699	158931135	158931194	NOS1AP	0.74	0.25	-0.23
A_16_P15318720	158939032	158939091	NOS1AP	0.89	0.82	-0.52
A_16_P00177935	158945769	158945828	NOS1AP	0.64	0.91	-0.48
A_16_P15318765	158952801	158952860	NOS1AP	0.95	1.23	-0.47
A_16_P00177968	158963529	158963588	NOS1AP	1.35	1.02	-0.22
A_16_P00177988	158979465	158979524	NOS1AP	1.37	1.84	-0.31
A_14_P107214	158988785	158988844	NOS1AP	1.13	1.20	-0.47
A_16_P15318899	159000513	159000572	NOS1AP	1.06	0.93	-0.29
A_16_P00178048	159012148	159012207	NOS1AP	1.14	1.29	-0.29
A_16_P00178068	159024007	159024066	NOS1AP	1.46	1.53	-0.24
A_16_P35333730	159030457	159030516	NOS1AP	1.09	1.26	-0.22
A_16_P15319011	159039288	159039347	NOS1AP	0.95	0.80	-0.38
A_16_P00178126	159054975	159055034	NOS1AP	0.90	0.80	-0.25
A_16_P15319079	159060390	159060449	NOS1AP	1.30	1.06	-0.40
A_14_P127720	159069522	159069576	NOS1AP	0.21	1.16	0.24
A_14_P138496	159077369	159077428	C1orf111	0.83	1.35	-0.12
A_16_P15319155	159083076	159083135	chr1:159083076-159083135	0.93	1.13	-0.28
A_16_P15319176	159089481	159089540	chr1:159089481-159089540	1.11	1.69	-0.19
A_16_P15319191	159093749	159093808	chr1:159093749-159093808	1.01	1.46	-0.17
A_14_P130380	159097767	159097826	SH2D1B	1.11	0.45	-0.31

A_16_P15319213	159104248	159104307	SH2D1B	1.13	1.18	-0.31
A_14_P137647	159110162	159110221	SH2D1B	1.22	1.08	-0.34
A_16_P00178240	159118547	159118606	chr1:159118547-159118606	1.04	1.76	0.03
A_16_P15319261	159133034	159133093	chr1:159133034-159133093	0.49	1.27	-0.69
A_16_P15319276	159143946	159144005	chr1:159143946-159144005	0.83	0.83	-0.30
A_16_P15319302	159164580	159164639	chr1:159164580-159164639	1.31	0.50	-0.65
A_16_P00178289	159190037	159190096	chr1:159190037-159190096	1.72	0.90	-0.15
A_16_P15319341	159197987	159198046	chr1:159197987-159198046	1.18	0.60	-0.35
A_16_P15319365	159205158	159205217	UHMK1	0.86	1.27	-0.53
A_16_P35334157	159213872	159213931	UHMK1	1.03	1.05	-0.59
A_16_P00178328	159219520	159219579	UHMK1	0.94	1.06	-0.25
A_14_P109472	159224611	159224670	UHMK1	0.41	1.88	-0.51
A_16_P15319422	159229544	159229603	AL137257	1.20	1.05	-0.41
A_16_P35334231	159238154	159238213	chr1:159238154-159238213	0.48	1.17	0.14
A_16_P15319470	159249719	159249778	chr1:159249719-159249778	1.40	0.44	-0.39
A_16_P00178389	159258771	159258830	chr1:159258771-159258830	1.41	1.93	-0.47
A_14_P129276	159265291	159265350	UAP1	1.08	1.23	-0.25
A_16_P35334326	159272081	159272140	UAP1	0.61	1.16	-0.43
A_16_P00178428	159278205	159278264	UAP1	0.71	0.92	-0.37
A_16_P15319588	159289757	159289816	UAP1	0.14	-0.54	-0.62
A_14_P124172	159301387	159301446	chr1:159301387-159301446	0.86	1.05	-0.19
A_16_P15319639	159313967	159314026	chr1:159313967-159314026	1.36	0.99	-0.55
A_16_P35334459	159322161	159322220	chr1:159322161-159322220	0.74	0.70	-0.73
A_14_P138813	159334038	159334091	DDR2	0.46	0.54	0.13
A_16_P15319749	159353237	159353296	DDR2	0.62	1.10	-0.45
A_16_P15319808	159370546	159370605	DDR2	0.69	1.22	-0.53
A_16_P00178612	159383921	159383980	DDR2	0.89	0.68	-0.23
A_16_P15319866	159391095	159391154	DDR2	1.30	0.69	-0.30
A_16_P15319881	159397516	159397575	DDR2	0.81	1.28	1.52
A_16_P00178646	159403988	159404047	DDR2	0.87	0.64	-0.46
A_14_P114165	159412679	159412738	DDR2	0.32	0.51	-0.53
A_16_P00178683	159422935	159422994	DDR2	0.27	0.86	-0.61
A_14_P133627	159435026	159435085	DDR2	1.37	1.16	-0.56
A_16_P15320009	159442431	159442490	DDR2	0.62	0.87	-0.41
A_16_P00178716	159455772	159455831	DDR2	1.12	0.58	-0.15
A_16_P00178727	159461368	159461426	DDR2	1.56	1.21	-0.18
A_16_P15320059	159468597	159468655	DDR2	1.55	1.27	-0.19
A_14_P119649	159474894	159474951	DDR2	0.69	0.72	-0.14
A_16_P15320118	159485725	159485784	BX537651	0.65	0.94	-0.65
A_16_P00178776	159498830	159498889	HSD17B7	0.02	0.62	-0.50
A_16_P35335034	159521117	159521167	chr1:159521117-159521167	0.65	1.34	-0.31
A_16_P15320226	159526746	159526805	chr1:159526746-159526805	1.37	1.58	-0.18
A_16_P00178798	159534224	159534283	chr1:159534224-159534283	0.89	1.00	-0.24
A_16_P35335089	159542786	159542845	chr1:159542786-159542845	1.37	1.36	-0.52
A_16_P35335106	159549079	159549138	chr1:159549079-159549138	1.08	1.38	-0.47
A_14_P122322	159556181	159556239	C1orf110	1.31	1.21	-0.27
A_14_P202502	159564101	159564160	C1orf110	1.13	1.18	-0.42
A_16_P35335166	159569825	159569884	C1orf110	0.00	0.65	-0.68

A_16_P00178859	159575716	159575775	chr1:159575716-159575775	1.30	1.12	-0.39
A_16_P15320398	159591480	159591539	chr1:159591480-159591539	0.55	0.66	-0.48
A_16_P15320461	159626693	159626752	chr1:159626693-159626752	0.80	1.30	-0.48
A_14_P128323	159681985	159682044	chr1:159681985-159682044	0.83	1.29	-0.64
A_16_P35335523	159711883	159711942	chr1:159711883-159711942	0.57	0.96	-0.42
A_16_P15320746	159741075	159741134	chr1:159741075-159741134	1.39	1.36	-0.32
A_16_P15320768	159752017	159752076	chr1:159752017-159752076	0.90	0.46	-0.48
A_16_P15320800	159765266	159765325	chr1:159765266-159765325	1.06	0.91	-0.31
A_16_P00179131	159769553	159769612	chr1:159769553-159769612	0.84	1.37	-0.47
A_14_P112139	159773838	159773891	RGS4	0.85	0.35	-0.40
A_16_P15320836	159777875	159777934	RGS4	0.73	0.91	-0.51
A_16_P35335686	159785006	159785065	chr1:159785006-159785065	1.27	0.92	-0.48
A_16_P15320871	159796604	159796663	chr1:159796604-159796663	0.35	0.79	-0.84
A_16_P35335733	159812385	159812444	chr1:159812385-159812444	0.58	0.95	-0.48
A_16_P15320916	159831119	159831178	chr1:159831119-159831178	0.69	0.93	-0.45
A_14_P129400	159848729	159848788	RGS5	1.06	1.30	-0.54
A_16_P35335842	159859075	159859134	RGS5	0.96	1.68	-0.33
A_14_P130292	159867947	159868006	RGS5	1.29	1.57	-0.37
A_16_P15321061	159879217	159879276	RGS5	0.71	2.42	0.17
A_16_P15321093	159893951	159894010	RGS5	0.25	1.23	-0.53
A_16_P35335977	159904175	159904234	RGS5	0.92	0.82	0.01
A_14_P133102	159916336	159916395	chr1:159916336-159916395	0.63	0.87	-0.29
A_16_P15321182	159932056	159932115	chr1:159932056-159932115	0.35	1.63	-0.04
A_16_P15321212	159945839	159945898	chr1:159945839-159945898	0.59	0.54	-0.13
A_16_P15321261	159965263	159965322	chr1:159965263-159965322	1.10	0.94	-0.54
A_16_P35336138	159988648	159988707	chr1:159988648-159988707	0.86	0.77	-0.63
A_16_P00179427	160006618	160006677	BC032239	1.64	1.65	-0.55
A_16_P35336192	160014933	160014992	BC032239	1.56	1.41	-0.38
A_14_P108397	160023784	160023843	CDCA1	0.24	2.00	-0.24
A_14_P129102	160029683	160029742	CDCA1	0.93	1.08	-0.40
A_16_P00179465	160039510	160039569	CDCA1	1.28	1.20	-0.39
A_16_P00179483	160049262	160049321	CDCA1	1.32	0.93	-0.57
A_14_P106162	160056784	160056843	CDCA1	0.49	0.91	-0.81
A_16_P15321480	160068408	160068467	chr1:160068408-160068467	0.46	0.26	-0.99
A_16_P15321518	160082260	160082319	chr1:160082260-160082319	0.66	1.68	-0.48
A_16_P15321649	160126739	160126798	chr1:160126739-160126798	0.95	1.33	-0.36
A_14_P122018	160169878	160169937	chr1:160169878-160169937	0.64	0.52	-0.81
A_16_P35336730	160210484	160210543	chr1:160210484-160210543	0.94	0.55	-0.59
A_16_P35336873	160282506	160282565	chr1:160282506-160282565	1.61	1.48	-0.29
A_14_P103374	160305744	160305803	chr1:160305744-160305803	1.17	0.98	-0.56
A_16_P15322099	160338560	160338619	chr1:160338560-160338619	0.88	0.88	-0.43
A_16_P15322198	160378521	160378580	chr1:160378521-160378580	1.53	1.05	-0.47
A_16_P00179990	160403472	160403531	chr1:160403472-160403531	1.03	0.69	-0.57
A_16_P35337201	160447913	160447972	chr1:160447913-160447972	1.42	1.31	-0.40
A_16_P15322374	160475353	160475412	chr1:160475353-160475412	1.19	1.00	-0.39
A_16_P15322452	160507620	160507679	chr1:160507620-160507679	0.86	1.04	-0.45
A_14_P137166	160543089	160543148	chr1:160543089-160543148	1.34	1.13	-0.49
A_16_P00180235	160583591	160583650	chr1:160583591-160583650	1.48	0.66	-0.33

A_16_P15322736	160618604	160618663	chr1:160618604-160618663	0.77	0.92	-0.45
A_14_P133772	160648751	160648810	chr1:160648751-160648810	0.72	0.94	-0.66
A_16_P15322855	160679352	160679411	chr1:160679352-160679411	0.64	1.01	-0.04
A_16_P15322937	160737494	160737553	chr1:160737494-160737553	0.67	0.20	-0.58
A_16_P15323004	160761015	160761074	chr1:160761015-160761074	0.76	0.71	-0.39
A_16_P15323058	160803346	160803405	chr1:160803346-160803405	1.16	1.71	-0.46
A_16_P00180491	160833791	160833850	chr1:160833791-160833850	1.02	1.18	-0.52
A_14_P127174	160884424	160884483	chr1:160884424-160884483	0.48	0.55	-0.34
A_16_P15323284	160928237	160928296	chr1:160928237-160928296	0.71	0.74	-0.48
A_16_P35338224	160961991	160962050	chr1:160961991-160962050	1.12	0.96	-0.12
A_14_P139339	161010813	161010872	chr1:161010813-161010872	0.80	1.23	-0.08
A_16_P00180734	161038556	161038615	chr1:161038556-161038615	1.07	1.90	-0.14
A_16_P15323587	161062631	161062690	chr1:161062631-161062690	1.48	1.14	-0.43
A_16_P00180806	161090023	161090082	chr1:161090023-161090082	1.48	1.14	-0.35
A_14_P111092	161125721	161125780	chr1:161125721-161125780	0.94	0.96	-0.31
A_16_P00180872	161166958	161167017	chr1:161166958-161167017	1.63	1.27	-0.31
A_16_P15323837	161192303	161192362	chr1:161192303-161192362	1.36	0.99	0.17
A_14_P116778	161232661	161232720	chr1:161232661-161232720	1.12	-0.27	-0.19
A_16_P15323950	161248441	161248500	chr1:161248441-161248500	1.14	0.73	-0.08
A_16_P00180985	161257134	161257193	chr1:161257134-161257193	1.43	0.69	-0.14
A_14_P109901	161264108	161264167	PBX1	0.49	0.99	-0.24
A_16_P00181014	161270997	161271056	PBX1	1.34	1.08	-0.29
A_16_P15324036	161280979	161281038	PBX1	0.64	0.45	-0.37
A_16_P00181044	161288118	161288177	PBX1	1.17	0.62	-0.50
A_16_P15324102	161301035	161301094	PBX1	1.45	0.80	0.07
A_16_P00181086	161310324	161310383	PBX1	1.48	0.96	-0.25
A_16_P15324152	161316375	161316434	PBX1	0.77	0.82	-0.37
A_16_P35339040	161325002	161325061	PBX1	0.73	0.22	-0.32
A_16_P15324219	161335452	161335511	PBX1	0.37	1.36	-0.50
A_14_P108826	161342831	161342890	PBX1	0.58	1.00	-0.23
A_16_P15324287	161354923	161354982	PBX1	1.10	0.60	-0.42
A_16_P15324320	161364564	161364623	PBX1	0.79	1.01	-0.45
A_14_P135647	161375600	161375653	PBX1	0.89	1.35	-0.60
A_16_P00181266	161388017	161388076	PBX1	1.44	0.59	-0.18
A_16_P35339288	161398240	161398299	PBX1	0.88	0.74	-0.14
A_16_P00181302	161403755	161403814	PBX1	0.90	1.37	-0.39
A_16_P15324487	161414142	161414201	PBX1	0.63	-1.01	-0.25
A_16_P15324508	161419655	161419714	PBX1	0.90	1.38	-0.25
A_14_P131780	161428857	161428916	PBX1	1.51	0.57	-0.22
A_16_P15324561	161434931	161434990	PBX1	0.55	1.61	-0.45
A_16_P15324583	161441966	161442025	PBX1	0.90	1.01	-0.54
A_16_P00181399	161448797	161448856	PBX1	0.76	0.59	-0.10
A_16_P35339515	161463306	161463365	PBX1	1.28	0.98	-0.40
A_16_P15324683	161472106	161472165	PBX1	1.06	0.95	-0.29
A_16_P00181464	161478405	161478464	PBX1	0.89	1.28	-0.20
A_14_P118233	161493539	161493587	PBX1	1.42	0.68	-0.07
A_16_P00181517	161501375	161501434	PBX1	1.56	1.39	-0.25
A_16_P35339673	161509672	161509731	PBX1	1.21	1.01	-0.37

A_16_P35339720	161522384	161522443	PBX1	1.01	1.22	-0.56
A_16_P00181592	161537451	161537510	PBX1	0.38	1.00	-0.49
A_14_P119839	161547713	161547771	PBX1	1.15	0.86	-0.35
A_16_P00181631	161554683	161554742	chr1:161554683-161554742	1.23	0.50	-0.39
A_16_P15324991	161562256	161562315	chr1:161562256-161562315	1.07	0.92	-0.36
A_16_P00181661	161570130	161570189	chr1:161570130-161570189	1.33	0.55	-0.39
A_16_P00181688	161585330	161585389	chr1:161585330-161585389	1.14	1.82	-0.42
A_16_P15325100	161603304	161603363	chr1:161603304-161603363	1.35	0.32	-0.23
A_16_P00181767	161634388	161634447	chr1:161634388-161634447	1.54	1.38	-0.12
A_16_P35340100	161662913	161662972	chr1:161662913-161662972	0.77	0.95	-0.73
A_14_P111452	161713992	161714051	chr1:161713992-161714051	0.99	1.18	-0.31
A_16_P00181911	161750990	161751049	chr1:161750990-161751049	1.20	0.84	-0.43
A_16_P15325542	161792779	161792838	chr1:161792779-161792838	0.51	1.24	-0.17
A_14_P116931	161824028	161824087	chr1:161824028-161824087	0.92	1.04	-0.58
A_16_P15325720	161873732	161873790	chr1:161873732-161873790	1.40	1.06	-0.22
A_16_P00182104	161886530	161886589	chr1:161886530-161886589	0.85	1.23	0.18
A_16_P15325771	161894623	161894682	chr1:161894623-161894682	1.82	1.37	-0.32
A_16_P15325795	161902955	161903014	LMX1A	1.24	1.02	-0.33
A_14_P113064	161908484	161908543	LMX1A	1.58	0.99	-0.25
A_16_P00182153	161914673	161914732	LMX1A	1.28	0.81	-0.46
A_16_P00182172	161923060	161923119	LMX1A	0.80	0.68	-0.46
A_14_P126771	161931366	161931425	LMX1A	1.06	1.05	-0.09
A_16_P15325913	161938696	161938755	LMX1A	1.40	1.32	-0.56
A_16_P15325939	161948041	161948100	LMX1A	1.31	0.41	-0.25
A_16_P00182242	161956479	161956538	LMX1A	1.09	1.45	-0.30
A_16_P00182260	161966723	161966782	LMX1A	1.04	1.21	-0.35
A_16_P35340884	161974577	161974636	LMX1A	0.93	1.05	-0.54
A_16_P15326038	161981587	161981646	LMX1A	1.04	0.67	-0.49
A_14_P105457	161995213	161995272	LMX1A	1.53	0.93	-0.09
A_16_P35340975	162003580	162003639	LMX1A	1.19	1.07	-0.36
A_14_P118938	162012100	162012159	LMX1A	1.10	1.46	-0.27
A_16_P00182365	162023716	162023775	LMX1A	0.89	1.26	-0.57
A_16_P15326196	162030743	162030802	LMX1A	0.65	-0.31	-0.49
A_16_P35341079	162038224	162038283	LMX1A	1.00	1.17	-0.57
A_16_P00182403	162044806	162044865	LMX1A	0.90	1.08	-0.56
A_14_P123583	162051217	162051276	LMX1A	0.37	1.98	-0.62
A_16_P15326281	162059489	162059548	chr1:162059489-162059548	1.26	0.75	-0.46
A_16_P15326308	162067212	162067271	chr1:162067212-162067271	1.24	1.57	-0.27
A_14_P121046	162073311	162073370	chr1:162073311-162073370	0.85	1.52	-0.30
A_16_P15326348	162080501	162080560	chr1:162080501-162080560	1.15	0.52	-0.32
A_16_P15326388	162094900	162094959	chr1:162094900-162094959	0.90	1.44	-0.42
A_14_P134940	162101990	162102049	RXRG	1.55	1.50	-0.27
A_16_P15326435	162111520	162111578	RXRG	1.20	0.54	-0.48
A_16_P35341350	162121108	162121167	RXRG	0.92	0.70	-0.31
A_16_P00182566	162127399	162127458	RXRG	0.85	1.10	-0.37
A_16_P00182574	162131302	162131361	RXRG	0.94	1.26	-0.24
A_14_P110181	162137430	162137489	RXRG	1.04	1.04	-0.45
A_16_P15326535	162141844	162141903	RXRG	1.01	0.85	-0.48

A_16_P00182599	162145753	162145812	RXRG	0.65	1.24	-0.43
A_16_P00182610	162151827	162151886	chr1:162151827-162151886	0.90	1.34	-0.46
A_16_P00182627	162160637	162160695	chr1:162160637-162160695	1.54	0.88	-0.39
A_16_P00182641	162169082	162169141	chr1:162169082-162169141	0.89	1.31	-0.22
A_16_P15326668	162186474	162186533	AK093132	1.03	0.63	-0.57
A_14_P104564	162204641	162204700	AK093132	0.95	0.94	-0.53
A_16_P15326762	162225577	162225636	AK093132	0.55	0.95	-0.65
A_16_P00182749	162237278	162237337	AK093132	1.36	0.96	-0.30
A_16_P15326824	162243680	162243739	AK093132	0.37	0.51	-0.97
A_16_P00182777	162249318	162249377	LRRC52	0.77	0.59	-0.19
A_16_P35341762	162255728	162255787	LRRC52	0.85	1.01	-0.63
A_16_P00182805	162265752	162265811	AK093132	0.94	0.53	-0.39
A_16_P15326909	162273399	162273458	AK093132	0.44	1.22	-0.44
A_16_P15326921	162279423	162279482	AK093132	0.86	0.95	-0.47
A_16_P15326959	162294144	162294203	chr1:162294144-162294203	1.19	0.93	-0.36
A_16_P15326994	162304982	162305041	chr1:162304982-162305041	1.04	0.83	-0.55
A_16_P00182882	162313774	162313833	chr1:162313774-162313833	0.42	1.28	-0.52
A_16_P00182893	162322547	162322606	chr1:162322547-162322606	1.52	1.39	-0.14
A_16_P15327044	162330294	162330353	chr1:162330294-162330353	1.34	1.35	-0.34
A_16_P15327070	162337898	162337957	MGST3	1.30	1.23	-0.29
A_14_P201942	162342675	162342734	MGST3	0.42	1.47	-0.52
A_16_P15327101	162350162	162350221	MGST3	1.35	1.27	-0.09
A_14_P122838	162355211	162355270	MGST3	1.24	1.35	-0.29
A_16_P00182951	162363668	162363727	ALDH9A1	0.83	0.98	-0.52
A_16_P00182962	162369793	162369852	ALDH9A1	0.60	1.54	-0.53
A_16_P15327172	162382633	162382692	ALDH9A1	0.62	0.84	-0.43
A_16_P00182985	162390239	162390298	ALDH9A1	1.70	1.61	-0.64
A_14_P115810	162396192	162396251	ALDH9A1	0.97	0.50	-0.34
A_16_P35342148	162412665	162412724	chr1:162412665-162412724	1.87	1.16	-0.19
A_16_P00183018	162420490	162420549	chr1:162420490-162420549	0.93	0.82	-0.34
A_14_P128524	162428374	162428433	TMCO1	0.54	0.92	-0.63
A_14_P135811	162428599	162428654	TMCO1	1.19	0.45	-0.16
A_16_P35342222	162442515	162442574	TMCO1	1.26	0.78	-0.28
A_16_P15327349	162459706	162459765	TMCO1	1.11	1.16	-0.49
A_14_P138648	162471527	162471586	AK093302	0.88	0.75	-0.35
A_16_P15327397	162489647	162489706	AK093302	1.19	0.68	-0.20
A_16_P15327420	162498972	162499031	AK093302	0.37	1.34	-0.41
A_16_P15327424	162508132	162508191	AK093302	0.68	1.15	-0.30
A_16_P15327440	162515983	162516042	AK093302	1.12	0.34	-0.19
A_16_P00183131	162522271	162522330	AK093302	0.67	0.13	-0.36
A_14_P138122	162532994	162533053	UCK2	0.34	0.33	-0.31
A_16_P00183156	162538172	162538231	UCK2	-0.14	0.58	-0.35
A_14_P108960	162554869	162554928	UCK2	0.21	0.95	-0.16
A_16_P15327565	162564307	162564366	UCK2	0.66	1.47	-0.36
A_16_P00183212	162575875	162575934	UCK2	0.58	0.79	-0.49
A_16_P15327597	162581533	162581592	UCK2	0.83	0.18	-0.34
A_14_P100997	162593555	162593606	UCK2	1.25	0.49	0.27
A_16_P15327673	162604037	162604095	UCK2	0.41	0.30	-0.41

A_16_P00183277	162610595	162610654	BX640859	1.15	1.26	-0.31
A_16_P00183292	162619315	162619374	AK055795	0.96	0.46	-0.50
A_14_P119990	162636473	162636532	AK055795	1.23	0.43	0.44
A_16_P15327793	162645417	162645476	AK055795	1.43	1.02	-0.35
A_16_P35342765	162660960	162661019	AK055795	1.47	1.59	-0.14
A_16_P00183359	162671176	162671235	AK055795	1.17	0.88	-0.61
A_16_P15327854	162685387	162685446	AK055795	0.74	1.54	-0.21
A_16_P00183375	162697294	162697353	AK055795	0.82	0.94	-0.25
A_16_P00183393	162718789	162718848	AK055795	0.67	1.01	-0.43
A_16_P35342857	162726969	162727028	AK055795	0.66	1.08	-0.58
A_16_P00183415	162732441	162732500	AK055795	0.73	0.41	-0.35
A_16_P15327977	162741925	162741984	AK055795	1.16	0.93	-0.46
A_16_P15328009	162752984	162753043	AK055795	0.94	0.93	-0.45
A_16_P00183483	162763405	162763464	AK055795	1.01	1.35	-0.21
A_14_P112595	162779364	162779423	FAM78B	0.85	1.68	-0.60
A_16_P15328122	162790075	162790134	FAM78B	1.53	2.09	-0.27
A_16_P00183544	162795543	162795602	FAM78B	1.61	1.54	-0.44
A_16_P15328154	162812512	162812571	FAM78B	1.64	1.26	-0.34
A_16_P00183575	162823422	162823481	FAM78B	1.04	1.49	-0.55
A_16_P15328244	162841356	162841415	FAM78B	1.33	0.73	-0.35
A_16_P15328281	162853315	162853374	FAM78B	0.45	1.14	-0.61
A_16_P00183659	162865519	162865578	FAM78B	1.20	1.07	-0.41
A_16_P15328340	162871602	162871660	chr1:162871602-162871660	0.31	1.28	-0.61
A_16_P15328379	162884355	162884414	chr1:162884355-162884414	1.20	1.25	-0.30
A_16_P15328392	162891303	162891362	chr1:162891303-162891362	1.14	1.88	-0.38
A_16_P35343345	162897556	162897615	chr1:162897556-162897615	0.62	0.48	-0.72
A_16_P35343363	162903727	162903786	chr1:162903727-162903786	0.30	0.83	-0.76
A_16_P00183734	162909239	162909298	chr1:162909239-162909298	0.44	0.85	-0.52
A_16_P15328456	162955388	162955447	chr1:162955388-162955447	1.29	1.00	-0.52
A_16_P15328460	162963280	162963339	chr1:162963280-162963339	1.00	0.55	-0.67
A_16_P15328470	162972462	162972521	chr1:162972462-162972521	0.93	0.89	-0.62
A_16_P15328486	162981149	162981208	chr1:162981149-162981208	0.69	0.61	-0.50
A_16_P15328503	162987158	162987217	chr1:162987158-162987217	0.33	1.06	-0.37
A_14_P137974	163002236	163002295	chr1:163002236-163002295	0.68	0.62	-0.53
A_16_P15328567	163008737	163008796	chr1:163008737-163008796	1.20	1.35	-0.40
A_16_P00183801	163027195	163027254	chr1:163027195-163027254	1.60	1.30	-0.35
A_16_P15328607	163038769	163038828	chr1:163038769-163038828	0.84	0.95	-0.45
A_16_P15328638	163050076	163050135	chr1:163050076-163050135	0.86	1.29	-0.73
A_16_P15328660	163056705	163056763	chr1:163056705-163056763	1.07	2.07	-0.37
A_16_P00183857	163062536	163062595	chr1:163062536-163062595	1.06	0.45	-0.54
A_16_P15328695	163068284	163068343	chr1:163068284-163068343	1.40	1.26	-0.40
A_16_P15328710	163078565	163078624	chr1:163078565-163078624	0.73	0.05	-0.61
A_16_P15328738	163087105	163087164	chr1:163087105-163087164	1.21	1.03	-0.39
A_16_P00183910	163095939	163095998	chr1:163095939-163095998	1.08	1.19	-0.36
A_16_P15328778	163102864	163102923	chr1:163102864-163102923	0.66	0.55	-0.50
A_16_P00183919	163118738	163118797	chr1:163118738-163118797	1.41	0.73	-0.50
A_14_P126954	163122697	163122756	chr1:163122697-163122756	1.15	1.73	-0.16
A_16_P15328802	163131153	163131212	chr1:163131153-163131212	1.40	0.87	-0.28

A_16_P00183942	163139367	163139426	chr1:163139367-163139426	0.23	0.22	-1.02
A_16_P15328847	163153652	163153711	chr1:163153652-163153711	0.54	0.59	-0.68
A_16_P15328875	163170407	163170466	chr1:163170407-163170466	0.84	1.22	-0.55
A_16_P35343833	163182144	163182203	chr1:163182144-163182203	0.88	-1.33	-0.68
A_16_P35343859	163192779	163192838	chr1:163192779-163192838	0.91	0.97	-0.32
A_16_P15328940	163207425	163207484	chr1:163207425-163207484	0.82	1.04	-0.55
A_16_P00184023	163214102	163214161	chr1:163214102-163214161	1.33	0.96	-0.36
A_16_P35343909	163223701	163223760	chr1:163223701-163223760	0.99	1.12	-0.56
A_16_P15328996	163243055	163243114	chr1:163243055-163243114	0.75	0.43	-0.58
A_16_P15329004	163246937	163246996	chr1:163246937-163246996	-0.20	-0.04	0.00
A_16_P15329017	163264162	163264221	chr1:163264162-163264221	0.86	0.96	-0.54
A_16_P35343980	163274862	163274921	chr1:163274862-163274921	0.26	1.28	0.10
A_16_P15329075	163282030	163282089	chr1:163282030-163282089	0.77	0.70	0.28
A_16_P15329090	163288444	163288503	chr1:163288444-163288503	1.29	1.34	0.32
A_16_P00184107	163294790	163294849	chr1:163294790-163294849	0.20	1.00	0.39
A_14_P201495	163300876	163300935	chr1:163300876-163300935	0.15	1.56	0.51
A_14_P126330	163305648	163305707	BC014341	0.58	0.95	0.26
A_16_P15329143	163313783	163313842	BC014341	1.02	1.32	0.48
A_14_P114497	163322798	163322856	BC014341	1.12	1.61	0.36
A_16_P15329203	163337361	163337420	chr1:163337361-163337420	0.61	1.51	0.06
A_16_P00184165	163347316	163347375	chr1:163347316-163347375	0.94	0.46	0.38
A_16_P35344167	163357223	163357282	chr1:163357223-163357282	1.16	0.32	0.20
A_16_P15329252	163366729	163366788	chr1:163366729-163366788	0.71	0.24	0.28
A_16_P15329275	163375565	163375624	chr1:163375565-163375624	0.66	-0.20	0.22
A_16_P35344231	163382534	163382593	chr1:163382534-163382593	1.21	0.64	0.30
A_16_P00184234	163395627	163395686	chr1:163395627-163395686	1.30	1.42	0.35
A_16_P15329358	163406261	163406320	chr1:163406261-163406320	0.65	0.96	0.24
A_16_P15329359	163437609	163437668	chr1:163437609-163437668	0.39	0.23	0.21
A_16_P15329390	163450554	163450613	chr1:163450554-163450613	0.79	1.31	0.17
A_16_P35344334	163457614	163457673	chr1:163457614-163457673	0.00	1.57	0.19
A_16_P00184281	163463727	163463786	chr1:163463727-163463786	0.69	0.55	0.37
A_16_P35344366	163470434	163470493	chr1:163470434-163470493	0.70	0.57	0.14
A_16_P35344372	163477705	163477764	chr1:163477705-163477764	1.03	0.56	0.21
A_16_P00184295	163486746	163486805	chr1:163486746-163486805	1.02	0.88	0.25
A_16_P00184304	163492243	163492302	chr1:163492243-163492302	0.85	1.13	0.33
A_16_P15329491	163507380	163507439	chr1:163507380-163507439	0.88	0.93	0.32
A_16_P35344449	163518910	163518969	chr1:163518910-163518969	0.29	-0.44	0.22
A_16_P35344468	163534298	163534357	chr1:163534298-163534357	0.76	1.12	0.23
A_14_P201180	163538774	163538833	chr1:163538774-163538833	0.66	0.75	-0.08
A_14_P136156	163545379	163545438	POGK	1.31	1.00	0.38
A_16_P15329576	163549777	163549836	POGK	1.06	1.06	0.37
A_14_P114293	163555737	163555796	AB040946	1.00	0.98	0.43
A_16_P15329628	163565712	163565771	TADA1L	0.16	0.00	0.36
A_16_P15329640	163570380	163570439	TADA1L	0.88	1.16	0.23
A_16_P00184428	163576490	163576549	TADA1L	0.07	0.53	0.16
A_16_P35344621	163580634	163580693	chr1:163580634-163580693	0.55	0.96	0.02
A_16_P35344640	163589271	163589330	AY726559	0.82	-0.22	0.06
A_16_P15329706	163595922	163595981	chr1:163595922-163595981	1.04	0.89	0.23

A_16_P15329750	163611992	163612051	chr1:163611992-163612051	1.10	1.10	0.22
A_16_P00184497	163619835	163619894	C1orf32	0.89	0.87	0.34
A_16_P15329805	163627906	163627965	C1orf32	1.15	0.93	0.33
A_16_P15329826	163635753	163635812	C1orf32	1.34	1.77	0.42
A_14_P130847	163653273	163653332	C1orf32	1.15	1.08	0.16
A_16_P35344862	163658627	163658685	C1orf32	1.36	0.99	0.33
A_16_P00184600	163673756	163673813	C1orf32	1.52	0.49	0.30
A_16_P35344925	163680112	163680171	chr1:163680112-163680171	1.02	1.27	0.10
A_16_P00184631	163693690	163693749	MAEL	1.41	1.12	0.61
A_14_P123522	163699466	163699525	MAEL	0.75	0.75	0.37
A_16_P00184648	163706136	163706195	MAEL	1.39	0.54	0.41
A_16_P00184652	163714684	163714743	MAEL	1.18	0.41	0.47
A_14_P121669	163722788	163722847	MAEL	0.88	1.08	0.08
A_16_P15330064	163733232	163733291	chr1:163733232-163733291	0.62	1.12	0.09
A_16_P00184674	163744253	163744312	chr1:163744253-163744312	1.13	1.39	0.47
A_16_P15330109	163753732	163753791	GPA33	0.58	1.01	0.10
A_16_P15330126	163759645	163759704	GPA33	1.10	1.17	0.39
A_16_P15330153	163768876	163768935	GPA33	1.07	1.37	0.25
A_14_P111103	163779246	163779305	GPA33	0.91	1.70	0.39
A_14_P201952	163790648	163790707	GPA33	1.31	1.43	0.48
A_16_P00184775	163797683	163797742	chr1:163797683-163797742	0.83	0.20	0.24
A_16_P15330251	163809278	163809337	chr1:163809278-163809337	1.22	0.21	0.39
A_16_P00184807	163819843	163819902	chr1:163819843-163819902	1.51	1.36	0.46
A_16_P00184829	163832659	163832718	chr1:163832659-163832718	1.48	1.21	-0.24
A_16_P15330347	163843159	163843218	chr1:163843159-163843218	0.86	0.52	-0.43
A_16_P15330393	163865026	163865085	chr1:163865026-163865085	1.23	0.93	-0.50
A_16_P15330445	163886156	163886215	chr1:163886156-163886215	1.32	1.28	-0.45
A_16_P00184926	163897918	163897977	chr1:163897918-163897977	1.04	0.91	-0.34
A_16_P35345485	163908294	163908353	chr1:163908294-163908353	1.53	1.76	-0.39
A_16_P15330513	163915774	163915833	chr1:163915774-163915833	0.42	0.29	-0.34
A_14_P132037	163929508	163929567	POU2F1	0.82	0.61	-0.26
A_16_P35345575	163940010	163940069	POU2F1	1.09	0.74	-0.44
A_16_P15330625	163951588	163951647	POU2F1	0.86	0.87	-0.58
A_16_P35345642	163959249	163959308	POU2F1	1.05	0.93	-0.49
A_14_P120545	163969912	163969971	POU2F1	0.90	0.91	-0.32
A_16_P15330693	163975712	163975771	POU2F1	0.62	0.38	-0.58
A_16_P00185071	163984403	163984462	POU2F1	1.11	1.67	-0.25
A_16_P15330746	163997408	163997467	POU2F1	0.69	1.36	-0.57
A_16_P15330776	164008278	164008337	POU2F1	0.51	1.23	-0.37
A_16_P15330782	164017377	164017436	POU2F1	0.84	1.61	-0.37
A_14_P129359	164030127	164030178	POU2F1	0.35	0.08	-0.27
A_14_P120608	164030233	164030292	POU2F1	0.41	1.49	-0.09
A_16_P00185153	164046261	164046320	POU2F1	1.42	1.21	-0.22
A_16_P15330877	164056721	164056780	POU2F1	0.99	1.28	-0.13
A_14_P122393	164064117	164064176	POU2F1	1.19	1.45	-0.16
A_16_P15330900	164070997	164071056	POU2F1	1.01	0.18	-0.44
A_16_P35345923	164080149	164080208	POU2F1	1.24	0.83	-0.22
A_16_P00185210	164088360	164088419	POU2F1	1.26	0.65	-0.39

A_16_P15330974	164097318	164097377	POU2F1	0.91	1.47	-0.13
A_16_P15330997	164103943	164104002	POU2F1	1.27	0.93	-0.28
A_14_P101718	164116897	164116956	POU2F1	1.27	1.66	-0.34
A_14_P101718	164116897	164116956	POU2F1	1.22	1.44	-0.30
A_14_P101718	164116897	164116956	POU2F1	1.28	1.66	-0.32
A_16_P15331048	164123299	164123358	BC037864	0.80	1.02	-0.43
A_14_P133409	164136314	164136358	CD247	0.89	1.41	-0.16
A_16_P00185324	164146332	164146391	CD247	1.88	0.89	-0.14
A_14_P119578	164157332	164157391	CD247	1.30	1.71	0.09
A_16_P35346205	164170453	164170512	CD247	1.40	1.41	-0.29
A_16_P15331218	164179938	164179997	CD247	0.90	1.32	-0.48
A_16_P15331243	164186677	164186736	CD247	0.59	-0.28	-0.42
A_14_P117337	164193343	164193402	CD247	1.32	0.96	-0.34
A_16_P00185423	164206247	164206306	CD247	1.19	0.37	-0.61
A_16_P00185444	164216118	164216177	CD247	1.46	1.19	-0.07
A_16_P35346355	164222219	164222278	chr1:164222219-164222278	1.33	1.72	-0.41
A_16_P00185464	164229035	164229094	chr1:164229035-164229094	1.16	1.22	-0.29
A_16_P00185472	164235155	164235212	chr1:164235155-164235212	1.42	1.03	-0.52
A_14_P116451	164242059	164242118	CREG1	1.12	1.64	-0.95
A_16_P15331410	164247118	164247177	CREG1	1.34	0.97	-0.31
A_16_P00185505	164253171	164253230	CREG1	0.89	0.99	-0.29
A_16_P35346459	164259935	164259994	chr1:164259935-164259994	1.48	1.75	-0.34
A_16_P15331458	164265476	164265535	chr1:164265476-164265535	0.83	0.65	-0.38
A_16_P35346494	164279195	164279254	chr1:164279195-164279254	1.40	1.03	-0.18
A_14_P117231	164294192	164294251	chr1:164294192-164294251	1.28	1.81	-0.39
A_16_P00185576	164318124	164318183	chr1:164318124-164318183	1.01	0.73	-0.60
A_16_P15331594	164334027	164334086	RCSD1	0.77	0.39	-0.41
A_14_P122259	164346476	164346535	RCSD1	1.14	1.66	-0.34
A_16_P15331657	164354144	164354203	RCSD1	0.78	1.71	-0.28
A_16_P15331685	164363806	164363865	RCSD1	1.04	1.10	-0.60
A_14_P139918	164370799	164370850	RCSD1	1.09	0.88	0.03
A_16_P15331720	164378026	164378085	RCSD1	1.15	1.51	-0.52
A_16_P35346787	164394949	164395008	RCSD1	0.58	1.17	-0.47
A_14_P107584	164406139	164406198	RCSD1	0.80	0.66	-0.45
A_16_P15331828	164411358	164411417	chr1:164411358-164411417	1.27	0.93	-0.32
A_14_P123552	164427619	164427678	MPZL1	0.73	0.83	-0.37
A_16_P00185782	164436885	164436944	MPZL1	1.48	1.50	-0.41
A_16_P15331897	164443369	164443428	MPZL1	1.35	1.37	-0.45
A_16_P15331914	164449907	164449966	MPZL1	1.02	0.69	-0.49
A_16_P35346958	164460164	164460223	MPZL1	0.73	1.18	-0.33
A_14_P135623	164466252	164466311	MPZL1	0.91	0.26	-0.36
A_16_P15331994	164478064	164478123	MPZL1	1.20	1.28	-0.47
A_16_P15332001	164485350	164485409	MPZL1	1.48	1.10	-0.32
A_14_P100965	164490984	164491043	MPZL1	0.69	0.61	-0.23
A_16_P15332049	164501961	164502020	chr1:164501961-164502020	1.59	1.09	-0.29
A_16_P00185894	164510534	164510593	SAC	0.71	1.21	-0.53
A_14_P101731	164523847	164523892	SAC	0.75	0.99	-0.26
A_16_P35347156	164537351	164537410	SAC	1.30	1.14	-0.27

A_16_P15332154	164546382	164546441	SAC	0.93	0.83	-0.36
A_14_P121666	164557285	164557344	SAC	0.81	-0.30	-0.34
A_16_P35347232	164571185	164571244	SAC	0.50	0.56	-0.48
A_14_P104416	164581377	164581436	SAC	1.00	1.79	-0.31
A_16_P35347286	164594716	164594775	SAC	0.69	0.81	-0.41
A_14_P118117	164602590	164602640	SAC	0.93	0.43	-0.21
A_16_P00186025	164611447	164611506	SAC	1.13	1.54	-0.13
A_16_P15332293	164616098	164616157	chr1:164616098-164616157	1.11	1.18	-0.38
A_16_P35347358	164620929	164620988	BRP44	1.14	1.36	-0.24
A_14_P100123	164632887	164632946	BRP44	0.89	0.74	0.10
A_16_P15332373	164641891	164641950	IQWD1	0.80	0.53	-0.43
A_14_P113350	164652719	164652778	IQWD1	0.63	1.13	-0.06
A_16_P35347451	164659539	164659598	IQWD1	0.71	1.46	-0.63
A_16_P15332408	164672673	164672732	IQWD1	0.96	0.84	-0.36
A_16_P15332422	164680133	164680192	IQWD1	0.94	0.89	-0.21
A_16_P15332429	164688332	164688391	IQWD1	0.70	0.92	0.07
A_16_P35347519	164695976	164696035	IQWD1	0.65	1.14	-0.57
A_14_P108330	164704851	164704910	IQWD1	1.17	0.02	-0.32
A_16_P35347553	164710804	164710863	IQWD1	0.59	0.35	-0.69
A_16_P15332514	164721318	164721377	IQWD1	0.62	-0.51	-0.34
A_16_P15332539	164728978	164729037	IQWD1	0.89	0.57	-0.28
A_16_P15332553	164734866	164734925	IQWD1	1.01	0.71	-0.53
A_16_P00186194	164743897	164743956	IQWD1	1.01	1.08	-0.34
A_14_P131597	164751758	164751817	IQWD1	0.89	-0.12	-0.49
A_16_P15332633	164761410	164761469	IQWD1	0.88	0.70	-0.42
A_16_P00186236	164767299	164767358	IQWD1	1.28	0.01	-0.55
A_14_P125737	164779111	164779170	chr1:164779111-164779170	1.63	1.05	-0.21
A_16_P15332705	164784114	164784173	AK091271	1.18	1.11	-0.21
A_16_P15332721	164788417	164788474	GPR161	1.43	-0.49	-0.16
A_16_P00186288	164792852	164792911	GPR161	1.60	1.79	-0.40
A_14_P123215	164800436	164800495	GPR161	1.05	0.41	-0.14
A_16_P00186308	164805092	164805151	GPR161	0.91	1.30	-0.39
A_16_P15332778	164810985	164811044	GPR161	0.65	1.20	-0.40
A_16_P15332792	164818083	164818142	GPR161	0.83	0.98	-0.21
A_14_P117410	164826411	164826470	GPR161	0.69	0.56	-0.40
A_16_P00186340	164834786	164834844	GPR161	0.95	0.71	-0.54
A_16_P15332862	164849477	164849536	chr1:164849477-164849536	0.97	1.53	-0.44
A_16_P35347980	164864380	164864439	chr1:164864380-164864439	1.31	0.50	-0.40
A_16_P35347997	164870923	164870982	chr1:164870923-164870982	1.35	1.24	-0.38
A_16_P15332914	164875086	164875145	chr1:164875086-164875145	1.24	0.71	-0.25
A_16_P00186392	164880536	164880595	TIPRL	1.45	0.30	-0.04
A_14_P101478	164884788	164884847	TIPRL	1.76	0.86	-0.22
A_16_P00186417	164897488	164897547	TIPRL	1.28	1.10	-0.19
A_14_P130224	164902883	164902942	TIPRL	0.64	0.94	-0.31
A_16_P15332985	164915191	164915250	chr1:164915191-164915250	1.60	0.79	-0.25
A_16_P15332991	164921853	164921912	chr1:164921853-164921912	0.80	0.61	-0.80
A_16_P15333013	164929569	164929628	SFT2D2	1.12	1.10	-0.41
A_16_P15333037	164937438	164937497	SFT2D2	1.25	1.05	-0.23

A_14_P111113	164946817	164946876	AK091404	0.53	1.17	-0.50
A_16_P15333077	164952465	164952524	AK091404	0.95	0.58	-0.31
A_16_P35348205	164962306	164962365	AK091404	1.00	1.50	-0.45
A_14_P123765	164972509	164972568	AK091404	1.34	0.79	-0.16
A_16_P00186499	164978948	164979007	AK091404	1.13	0.97	-0.46
A_16_P00186513	164986062	164986121	TBX19	1.11	1.24	-0.59
A_16_P00186526	164994587	164994646	TBX19	1.27	1.41	-0.38
A_14_P124740	165003836	165003895	TBX19	1.09	1.14	-0.21
A_16_P15333184	165009575	165009634	TBX19	0.91	0.72	-0.55
A_16_P15333215	165021016	165021075	chr1:165021016-165021075	1.22	1.23	-0.37
A_16_P35348354	165028129	165028188	chr1:165028129-165028188	1.24	0.92	-0.30
A_16_P00186580	165045117	165045176	chr1:165045117-165045176	1.66	1.25	-0.23
A_16_P15333281	165065912	165065971	chr1:165065912-165065971	1.37	1.74	-0.43
A_16_P00186639	165085938	165085997	chr1:165085938-165085997	1.44	0.29	0.36
A_14_P103934	165100386	165100445	chr1:165100386-165100445	0.55	1.29	0.18
A_16_P00186692	165120827	165120886	AK097492	0.23	1.63	0.07
A_16_P15333507	165139569	165139628	chr1:165139569-165139628	0.89	0.89	0.29
A_16_P15333529	165145780	165145839	chr1:165145780-165145839	1.22	1.62	0.61
A_16_P00186763	165156759	165156818	chr1:165156759-165156818	0.58	0.62	0.22
A_16_P15333569	165163186	165163245	chr1:165163186-165163245	0.99	0.17	0.60
A_16_P00186783	165173147	165173206	AF085892	1.31	0.50	0.30
A_16_P15333616	165184691	165184750	AF085892	0.64	0.76	0.13
A_16_P15333655	165198942	165199001	chr1:165198942-165199001	0.87	-0.20	0.38
A_16_P00186836	165210204	165210263	chr1:165210204-165210263	1.22	0.59	0.44
A_16_P00186850	165219242	165219301	chr1:165219242-165219301	1.10	1.18	0.28
A_16_P00186866	165232486	165232545	chr1:165232486-165232545	1.66	0.75	0.25
A_16_P15333750	165237097	165237156	chr1:165237097-165237156	0.70	1.17	0.21
A_16_P00186881	165245732	165245791	chr1:165245732-165245791	0.61	1.33	0.29
A_16_P00186888	165254604	165254663	chr1:165254604-165254663	1.36	0.31	0.33
A_16_P15333791	165258476	165258535	chr1:165258476-165258535	0.86	0.58	0.31
A_16_P35348925	165263038	165263097	chr1:165263038-165263097	1.10	0.93	0.28
A_16_P15333813	165269098	165269157	chr1:165269098-165269157	0.81	1.33	0.35
A_14_P102758	165279288	165279347	XCL1	0.66	1.09	0.22
A_16_P15333870	165287462	165287521	chr1:165287462-165287521	0.57	0.19	0.01
A_16_P15333886	165292699	165292758	chr1:165292699-165292758	0.93	1.16	0.23
A_16_P35349035	165303106	165303165	chr1:165303106-165303165	1.35	1.04	0.34
A_16_P15333957	165318190	165318249	chr1:165318190-165318249	0.96	-0.46	0.30
A_16_P35349154	165341132	165341191	chr1:165341132-165341191	0.98	1.39	0.19
A_14_P109703	165374190	165374249	chr1:165374190-165374249	0.97	0.55	0.50
A_16_P15334132	165389975	165390034	chr1:165389975-165390034	1.32	1.56	0.38
A_14_P114904	165397443	165397502	DPT	1.25	1.01	0.37
A_16_P15334184	165405093	165405152	DPT	0.83	1.12	0.31
A_16_P35349334	165410814	165410873	DPT	0.92	1.39	0.26
A_16_P35349356	165416945	165417004	DPT	0.45	0.77	0.14
A_16_P00187171	165422572	165422631	DPT	1.11	0.10	0.22
A_14_P138483	165429075	165429134	DPT	1.12	0.59	0.35
A_16_P35349433	165444802	165444861	chr1:165444802-165444861	1.07	1.72	0.35
A_16_P00187223	165455980	165456039	chr1:165455980-165456039	0.45	0.29	0.34

A_16_P15334376	165472716	165472775	chr1:165472716-165472775	0.62	1.00	0.21
A_14_P125441	165489450	165489506	BC009319	1.00	1.45	0.35
A_14_P202365	165493318	165493377	BC009319	1.44	1.23	0.45
A_16_P15334549	165536157	165536216	chr1:165536157-165536216	0.83	1.14	0.29
A_16_P00187413	165581599	165581658	chr1:165581599-165581658	0.41	0.94	0.35
A_16_P15334684	165603316	165603375	chr1:165603316-165603375	0.97	0.58	0.28
A_14_P111383	165620422	165620481	BC041008	0.69	1.41	0.35
A_16_P00187483	165634461	165634520	BC041008	0.49	0.66	0.48
A_16_P15334786	165644168	165644227	BC041008	0.62	0.81	0.11
A_16_P15334805	165653377	165653436	BC041008	1.07	1.58	0.49
A_16_P00187537	165674444	165674503	BC041008	1.58	1.14	0.59
A_16_P15334878	165684246	165684305	BC041008	1.36	1.52	0.44
A_16_P15334923	165708780	165708839	BC041008	0.99	0.61	0.21
A_16_P35350079	165720001	165720060	BC041008	1.33	1.74	0.61
A_16_P35350107	165730856	165730915	BC041008	1.15	1.08	0.38
A_16_P00187634	165750850	165750909	BC041008	1.13	1.18	0.36
A_14_P115199	165778527	165778586	BC041008	1.02	0.75	0.11
A_16_P15335134	165793762	165793821	chr1:165793762-165793821	0.86	1.01	0.36
A_16_P15335168	165804062	165804121	chr1:165804062-165804121	1.74	1.40	0.62
A_16_P15335185	165809168	165809227	ATP1B1	0.28	0.73	0.65
A_14_P115648	165818351	165818410	ATP1B1	0.88	1.04	0.35
A_16_P00187761	165825195	165825254	ATP1B1	1.02	1.05	0.38
A_16_P00187779	165833665	165833724	NME7	1.01	1.19	0.28
A_16_P15335301	165842438	165842497	NME7	1.17	1.37	0.40
A_16_P35350460	165848575	165848634	NME7	0.34	0.66	0.31
A_16_P15335347	165857691	165857750	NME7	0.71	1.07	0.35
A_16_P35350507	165865891	165865950	NME7	0.46	0.92	0.30
A_16_P15335389	165873779	165873838	NME7	1.11	0.66	0.50
A_16_P15335418	165885462	165885521	NME7	1.23	0.75	0.35
A_16_P35350594	165897116	165897175	NME7	1.17	1.27	0.42
A_16_P15335469	165905634	165905693	NME7	0.44	1.20	0.31
A_16_P00187911	165919132	165919191	NME7	0.74	1.02	0.14
A_16_P00187932	165931623	165931682	NME7	0.80	1.46	0.17
A_14_P115553	165938533	165938581	NME7	0.36	0.62	0.32
A_16_P15335576	165948301	165948360	NME7	0.78	1.18	0.47
A_16_P15335577	165958802	165958861	NME7	2.02	-4.34	-0.41
A_16_P15335602	165972916	165972975	NME7	1.93	-2.37	-0.31
A_14_P114627	165988174	165988233	NME7	0.61	0.80	0.08
A_16_P35350789	166003996	166004055	NME7	1.14	1.63	0.34
A_16_P15335658	166010822	166010881	NME7	0.72	0.28	0.27
A_16_P15335672	166018172	166018231	NME7	0.75	2.34	0.49
A_14_P118472	166024101	166024160	NME7	1.04	1.30	0.55
A_16_P15335730	166039993	166040052	NME7	0.88	0.37	0.40
A_16_P15335735	166048689	166048748	NME7	1.03	0.40	0.50
A_16_P35350907	166059425	166059484	NME7	0.57	1.28	0.37
A_14_P116551	166064793	166064852	NME7	1.41	1.53	0.49
A_14_P103069	166077906	166077965	BLZF1	0.11	1.70	-0.08
A_16_P15335826	166083166	166083225	BLZF1	0.82	1.01	0.21

A_14_P133801	166089142	166089201	BLZF1	0.77	0.95	0.46
A_14_P200046	166097020	166097079	BLZF1	0.40	1.42	0.12
A_16_P35351036	166108313	166108372	C1orf114	0.73	1.20	0.31
A_16_P15335874	166115643	166115702	C1orf114	0.67	0.79	0.10
A_16_P15335891	166121595	166121654	C1orf114	0.73	1.02	0.22
A_16_P15335923	166136826	166136885	chr1:166136826-166136885	0.53	0.78	0.09
A_14_P125328	166145538	166145597	BC041451	0.72	0.73	0.30
A_16_P15335949	166155150	166155209	BC041451	0.67	0.88	-0.14
A_14_P108355	166164405	166164464	AJ237724	1.08	1.16	0.43
A_16_P00188203	166169540	166169599	SLC19A2	1.18	0.24	0.38
A_16_P15336009	166173878	166173937	SLC19A2	1.09	1.00	0.37
A_14_P120038	166178428	166178487	SLC19A2	0.92	0.65	0.28
A_16_P15336038	166183924	166183983	SLC19A2	1.13	0.72	0.40
A_16_P15336064	166193233	166193292	chr1:166193233-166193292	1.21	1.29	0.30
A_16_P35351274	166205387	166205446	chr1:166205387-166205446	0.52	0.70	0.16
A_14_P108008	166215211	166215270	F5	1.34	0.97	0.48
A_16_P00188295	166224721	166224780	F5	1.25	1.57	0.61
A_16_P15336162	166230693	166230752	F5	0.76	1.04	0.28
A_16_P15336171	166236578	166236637	F5	0.66	0.94	0.29
A_14_P138837	166243152	166243211	F5	0.98	0.24	0.30
A_16_P00188339	166250653	166250712	F5	1.05	0.82	0.34
A_16_P00188356	166259024	166259083	F5	0.94	0.36	0.37
A_16_P15336249	166265754	166265813	F5	0.94	1.93	0.79
A_14_P121509	166273195	166273254	F5	1.18	1.19	0.39
A_16_P15336283	166279488	166279547	F5	0.40	0.50	0.40
A_14_P122687	166290369	166290428	SELP	1.33	1.00	0.59
A_16_P15336347	166299967	166300026	SELP	1.45	0.82	0.47
A_16_P35351596	166307782	166307841	SELP	1.22	0.23	0.37
A_16_P35351633	166316129	166316188	SELP	0.53	0.66	0.20
A_14_P111462	166324880	166324939	SELP	1.04	0.74	0.40
A_16_P00188487	166340492	166340551	chr1:166340492-166340551	1.05	1.05	0.32
A_16_P35351717	166348520	166348579	chr1:166348520-166348579	0.66	0.39	0.24
A_16_P15336498	166356526	166356582	chr1:166356526-166356582	0.59	1.21	0.07
A_16_P35351732	166372743	166372802	AK127098	1.18	1.12	0.59
A_16_P00188519	166381927	166381986	AK127098	1.18	0.95	0.44
A_16_P15336536	166392135	166392194	SELL	0.73	0.37	0.37
A_14_P104065	166397317	166397376	SELL	0.96	1.58	0.38
A_14_P101289	166404041	166404100	SELL	0.99	1.12	0.28
A_16_P15336603	166413132	166413191	AK127098	0.89	1.11	0.40
A_16_P35351853	166422436	166422495	AK127098	1.07	1.15	0.31
A_14_P132506	166428512	166428563	SELE	0.50	1.15	0.01
A_14_P139486	166434972	166435026	AK127098	1.18	0.95	0.45
A_16_P35351924	166441904	166441963	AK127098	0.25	1.10	0.17
A_16_P35351939	166451048	166451107	AK127098	0.99	1.54	0.72
A_16_P15336716	166464570	166464629	AK127098	1.13	1.39	0.56
A_16_P15336733	166472953	166473012	AK127098	0.88	-0.10	0.14
A_16_P00188656	166481230	166481289	AK127098	0.58	1.15	0.29
A_16_P15336771	166489679	166489738	AK127098	0.81	0.44	0.08

A_14_P116543	166495325	166495384	C1orf156	1.61	0.87	0.56
A_16_P35352051	166499580	166499639	C1orf112	0.71	1.51	0.38
A_16_P00188696	166504988	166505047	C1orf112	1.39	1.07	0.38
A_16_P35352098	166514183	166514242	C1orf112	1.01	1.07	0.36
A_14_P133078	166523597	166523656	C1orf112	1.04	0.88	0.32
A_16_P00188738	166533234	166533293	C1orf112	1.01	1.20	0.35
A_14_P110548	166544486	166544545	C1orf112	1.06	1.27	0.41
A_14_P117149	166553877	166553936	SCYL3	0.24	1.10	0.28
A_16_P35352239	166565120	166565179	SCYL3	1.05	1.03	0.35
A_16_P15336982	166570996	166571055	SCYL3	0.99	1.58	0.28
A_14_P122254	166579411	166579470	SCYL3	0.95	1.53	0.21
A_16_P15337030	166591491	166591550	SCYL3	0.75	1.26	0.17
A_14_P114035	166603388	166603447	chr1:166603388-166603447	1.19	1.10	0.41
A_16_P35352364	166610113	166610172	chr1:166610113-166610172	1.09	1.63	0.35
A_14_P100440	166622212	166622268	KIFAP3	0.20	1.45	0.33
A_16_P35352429	166632293	166632352	KIFAP3	-0.25	-2.00	-1.32
A_16_P15337159	166638516	166638575	KIFAP3	1.00	0.69	0.39
A_14_P137714	166654876	166654935	KIFAP3	1.14	0.86	0.35
A_16_P15337266	166673336	166673395	KIFAP3	0.58	1.02	0.21
A_16_P35352583	166678852	166678911	KIFAP3	0.48	0.82	0.05
A_16_P00189001	166685445	166685504	KIFAP3	0.95	0.68	0.47
A_14_P132002	166692970	166693029	KIFAP3	0.56	0.43	0.00
A_16_P15337344	166700147	166700206	KIFAP3	0.92	0.51	0.41
A_16_P00189034	166706675	166706734	KIFAP3	0.64	1.15	0.35
A_16_P35352695	166717370	166717429	KIFAP3	1.01	1.37	0.28
A_16_P15337401	166723772	166723831	KIFAP3	0.93	1.21	0.41
A_16_P35352724	166732189	166732248	KIFAP3	0.80	-0.77	0.23
A_16_P35352760	166741364	166741423	KIFAP3	0.80	1.40	0.33
A_16_P00189097	166747620	166747679	KIFAP3	0.49	1.24	0.30
A_16_P15337463	166756319	166756378	KIFAP3	0.73	1.23	0.32
A_16_P15337475	166763614	166763673	KIFAP3	0.78	0.95	0.18
A_14_P102376	166773414	166773473	KIFAP3	0.69	1.77	0.54
A_16_P15337501	166784871	166784930	chr1:166784871-166784930	0.99	0.75	0.36
A_14_P111146	166803474	166803533	chr1:166803474-166803533	0.47	0.53	0.15
A_16_P15337584	166836025	166836084	chr1:166836025-166836084	0.31	0.97	0.33
A_16_P15337648	166861789	166861848	chr1:166861789-166861848	1.03	0.76	0.14
A_16_P15337695	166882982	166883041	chr1:166882982-166883041	0.47	0.89	0.40
A_16_P15337719	166901404	166901463	chr1:166901404-166901463	0.73	1.39	0.23
A_16_P15337830	166940205	166940264	chr1:166940205-166940264	1.35	1.40	0.40
A_16_P15337884	166963233	166963292	chr1:166963233-166963292	0.90	1.56	0.42
A_16_P15337942	166990424	166990483	chr1:166990424-166990483	0.43	1.21	0.13
A_16_P15338011	167014611	167014670	chr1:167014611-167014670	1.23	0.50	0.19
A_14_P103461	167047223	167047282	chr1:167047223-167047282	0.38	0.75	0.05
A_16_P15338149	167077293	167077351	chr1:167077293-167077351	1.05	0.97	0.17
A_16_P15338176	167105965	167106024	chr1:167105965-167106024	0.94	0.80	0.16
A_16_P15338217	167125744	167125803	chr1:167125744-167125803	0.77	1.58	0.36
A_16_P00189566	167145179	167145238	chr1:167145179-167145238	0.98	1.04	0.13
A_16_P15338282	167171669	167171728	AK096329	0.62	0.78	0.08

A_16_P00189604	167184619	167184678	AK096329	0.65	0.44	0.19
A_14_P108316	167195184	167195243	AK096329	1.21	1.42	0.18
A_16_P15338352	167205146	167205205	AK096329	0.35	0.16	0.16
A_16_P35353682	167215443	167215502	AK096329	0.83	0.72	0.25
A_16_P00189668	167231762	167231821	chr1:167231762-167231821	1.06	1.26	0.28
A_16_P35353735	167239463	167239522	SCYL1BP1	0.63	0.13	0.18
A_14_P106032	167245470	167245527	SCYL1BP1	0.87	0.30	0.18
A_14_P128387	167248299	167248358	SCYL1BP1	1.52	1.14	0.29
A_16_P15338470	167253784	167253843	SCYL1BP1	0.95	1.48	0.23
A_16_P00189726	167265579	167265638	chr1:167265579-167265638	1.32	1.42	0.33
A_16_P15338522	167279487	167279546	chr1:167279487-167279546	0.64	0.74	0.06
A_16_P15338564	167293370	167293429	chr1:167293370-167293429	0.57	1.17	0.25
A_16_P15338587	167307479	167307538	AK056067	0.82	1.13	0.23
A_16_P35353928	167320025	167320084	AK056067	0.94	0.50	0.24
A_16_P15338640	167338596	167338655	AK056067	0.61	0.99	0.06
A_16_P15338664	167348378	167348437	chr1:167348378-167348437	0.40	0.90	0.04
A_16_P35353999	167356596	167356655	chr1:167356596-167356655	0.91	0.95	0.12
A_16_P35353999	167356596	167356655	chr1:167356596-167356655	0.83	0.82	0.13
A_16_P35353999	167356596	167356655	chr1:167356596-167356655	0.87	1.03	0.04
A_14_P126724	167364312	167364371	chr1:167364312-167364371	1.38	1.46	0.18
A_16_P00189866	167370794	167370853	PRRX1	0.66	0.67	0.36
A_14_P126419	167380048	167380107	PRRX1	1.33	1.39	0.24
A_16_P35354102	167386811	167386870	PRRX1	1.13	1.23	0.30
A_16_P15338804	167392576	167392635	PRRX1	1.21	1.49	0.27
A_14_P133283	167404408	167404467	PRRX1	0.86	0.87	0.21
A_16_P15338885	167415891	167415950	PRRX1	0.86	1.15	0.19
A_16_P15338910	167422886	167422945	PRRX1	1.24	1.53	0.01
A_16_P35354246	167430766	167430825	PRRX1	1.35	1.26	0.35
A_16_P15338957	167438250	167438309	PRRX1	0.77	1.18	0.17
A_16_P00190016	167446069	167446128	chr1:167446069-167446128	0.02	1.17	-0.09
A_16_P00190037	167456895	167456954	chr1:167456895-167456954	0.91	0.60	0.21
A_16_P15339049	167477699	167477758	chr1:167477699-167477758	0.81	1.04	0.26
A_14_P136098	167504573	167504632	chr1:167504573-167504632	1.12	1.26	0.26
A_16_P15339164	167527737	167527796	chr1:167527737-167527796	0.99	1.34	0.22
A_16_P00190148	167562093	167562152	chr1:167562093-167562152	0.96	0.80	0.24
A_16_P00190188	167586035	167586094	chr1:167586035-167586094	1.10	0.92	0.26
A_16_P15339359	167619723	167619782	chr1:167619723-167619782	0.91	1.46	0.21
A_16_P15339378	167630519	167630577	chr1:167630519-167630577	0.44	1.10	0.10
A_16_P15339386	167636341	167636400	C1orf129	0.57	0.01	0.04
A_16_P35354705	167646367	167646426	C1orf129	0.87	1.35	0.28
A_16_P00190269	167652578	167652637	C1orf129	0.78	0.11	0.16
A_16_P00190300	167672511	167672570	C1orf129	0.76	1.18	0.17
A_16_P00190316	167690695	167690754	C1orf129	1.11	1.01	-0.05
A_16_P00190316	167690695	167690754	C1orf129	1.12	1.27	-0.01
A_16_P00190316	167690695	167690754	C1orf129	1.09	1.36	-0.04
A_16_P00190329	167698927	167698986	C1orf129	0.70	1.28	-0.08
A_16_P35354838	167708934	167708993	chr1:167708934-167708993	0.31	1.62	0.20
A_16_P15339530	167715809	167715868	chr1:167715809-167715868	1.04	1.28	0.26

A_16_P35354866	167725730	167725789	chr1:167725730-167725789	0.41	1.25	0.20
A_16_P15339563	167740774	167740833	chr1:167740774-167740833	0.77	0.76	0.05
A_16_P35354910	167768349	167768408	chr1:167768349-167768408	0.69	0.57	0.25
A_16_P00190382	167777293	167777352	chr1:167777293-167777352	1.08	0.92	0.32
A_16_P15339632	167783927	167783986	chr1:167783927-167783986	0.80	1.96	0.36
A_14_P200905	167792143	167792202	FMO3	0.54	0.71	0.29
A_14_P120355	167796122	167796181	FMO3	0.99	0.47	0.27
A_16_P00190426	167811557	167811616	FMO3	1.08	0.78	0.27
A_14_P130322	167816847	167816906	FMO3	0.30	0.95	-0.25
A_16_P00190446	167823739	167823798	chr1:167823739-167823798	0.98	0.41	0.22
A_16_P00190456	167836196	167836255	chr1:167836196-167836255	0.68	0.63	0.21
A_16_P15339793	167848264	167848323	AK130511	0.67	1.02	0.21
A_16_P35355150	167854298	167854357	AK130511	0.15	0.67	0.16
A_16_P15339824	167862431	167862490	chr1:167862431-167862490	1.14	0.73	0.34
A_16_P35355177	167871597	167871656	chr1:167871597-167871656	0.89	0.43	0.10
A_16_P35355194	167877422	167877481	chr1:167877422-167877481	1.34	1.21	0.44
A_16_P15339879	167884235	167884294	chr1:167884235-167884294	0.63	1.44	0.19
A_16_P35355246	167894209	167894268	FMO2	1.04	1.08	0.30
A_14_P122882	167906465	167906524	FMO2	1.18	0.94	0.75
A_16_P00190593	167919607	167919666	chr1:167919607-167919666	1.49	0.80	0.40
A_16_P15339982	167927265	167927324	chr1:167927265-167927324	0.80	1.59	0.28
A_16_P15339988	167934430	167934489	chr1:167934430-167934489	0.88	0.85	0.16
A_16_P35355345	167946743	167946802	chr1:167946743-167946802	0.80	0.39	0.10
A_16_P15340013	167952815	167952874	FMO1	1.09	1.21	0.42
A_16_P00190627	167959796	167959855	FMO1	0.74	0.48	0.22
A_14_P119086	167967577	167967636	FMO1	1.11	1.50	0.35
A_14_P101997	167986514	167986573	FMO1	0.28	-0.16	0.21
A_16_P00190682	168000006	168000065	chr1:168000006-168000065	1.45	0.61	0.29
A_14_P100966	168015038	168015097	BC002780	0.69	1.54	0.34
A_14_P127176	168017024	168017079	FMO4	0.84	0.78	0.37
A_16_P15340170	168027733	168027792	FMO4	1.09	1.23	0.32
A_16_P15340194	168035419	168035478	FMO4	0.52	1.78	0.47
A_14_P120987	168042551	168042610	FMO4	1.27	1.81	0.23
A_16_P15340245	168056073	168056132	chr1:168056073-168056132	1.29	1.58	0.38
A_16_P35355644	168086053	168086112	chr1:168086053-168086112	1.14	1.03	0.15
A_16_P00190790	168130539	168130598	chr1:168130539-168130598	1.22	0.44	0.13
A_14_P126263	168156194	168156253	chr1:168156194-168156253	0.89	0.71	0.10
A_16_P15340415	168180225	168180284	chr1:168180225-168180284	0.71	0.82	0.32
A_14_P129006	168191110	168191169	BAT2D1	0.11	0.30	0.02
A_16_P35355858	168207748	168207807	BAT2D1	1.16	0.22	0.42
A_14_P116227	168212909	168212968	BAT2D1	1.51	1.79	0.43
A_16_P35355908	168224147	168224206	BAT2D1	1.13	1.13	0.57
A_16_P00190920	168233673	168233732	BAT2D1	0.77	1.57	0.44
A_16_P00190938	168242184	168242243	BAT2D1	1.02	1.38	0.46
A_14_P104929	168250905	168250964	BAT2D1	1.08	0.93	0.44
A_16_P35356014	168266976	168267035	BAT2D1	0.80	1.90	0.18
A_16_P00190985	168275895	168275954	BAT2D1	1.17	1.06	0.45
A_14_P133961	168284871	168284930	BAT2D1	1.12	0.93	0.39

A_16_P15340696	168294873	168294932	chr1:168294873-168294932	1.03	0.43	0.19
A_16_P15340712	168308519	168308578	chr1:168308519-168308578	0.66	0.58	0.28
A_16_P15340718	168319151	168319210	chr1:168319151-168319210	0.46	1.29	0.35
A_16_P00191037	168325888	168325947	chr1:168325888-168325947	1.59	1.56	0.43
A_14_P105609	168336253	168336312	MYOC	0.91	1.01	0.41
A_16_P35356195	168341140	168341199	MYOC	1.40	0.88	0.51
A_16_P15340792	168347624	168347683	MYOC	1.11	1.15	0.27
A_14_P113207	168353615	168353674	chr1:168353615-168353674	1.21	0.61	0.33
A_16_P15340824	168357734	168357793	chr1:168357734-168357793	0.91	1.85	0.43
A_16_P15340846	168367108	168367166	chr1:168367108-168367166	1.16	0.17	0.19
A_16_P35356291	168376427	168376486	chr1:168376427-168376486	0.68	0.81	0.25
A_16_P15340885	168385458	168385517	chr1:168385458-168385517	0.54	0.95	0.14
A_16_P35356333	168404635	168404694	VAMP4	0.54	0.72	-0.09
A_16_P00191145	168411555	168411614	VAMP4	0.88	0.84	0.26
A_16_P35356385	168420353	168420412	VAMP4	0.67	1.52	0.26
A_14_P101743	168429316	168429375	VAMP4	0.05	1.26	-0.40
A_16_P00191171	168439198	168439257	VAMP4	0.91	0.79	0.38
A_16_P15341017	168451188	168451247	chr1:168451188-168451247	1.31	0.84	0.19
A_16_P35356468	168457304	168457362	chr1:168457304-168457362	0.67	0.78	0.57
A_16_P00191203	168463722	168463781	chr1:168463722-168463781	0.29	0.30	0.17
A_16_P15341050	168468818	168468877	chr1:168468818-168468877	1.26	0.89	0.51
A_16_P15341070	168479861	168479920	chr1:168479861-168479920	1.32	0.15	0.31
A_14_P138416	168486491	168486541	KIAA0859	0.21	1.27	0.05
A_14_P111690	168489828	168489887	KIAA0859	1.37	1.04	0.48
A_16_P15341131	168497862	168497921	KIAA0859	1.47	1.61	0.52
A_16_P00191278	168506236	168506295	chr1:168506236-168506295	1.21	0.75	0.18
A_14_P101241	168515472	168515531	chr1:168515472-168515531	1.34	1.21	0.26
A_16_P15341197	168522764	168522823	chr1:168522764-168522823	1.77	0.82	0.55
A_16_P15341207	168530558	168530617	chr1:168530558-168530617	0.70	0.33	0.12
A_16_P00191323	168540453	168540512	chr1:168540453-168540512	1.18	0.90	0.22
A_16_P15341280	168554582	168554641	DNM3	1.02	1.40	0.32
A_16_P15341313	168570458	168570517	DNM3	1.33	1.50	0.34
A_16_P15341334	168577703	168577762	DNM3	1.02	1.33	0.40
A_16_P15341377	168590830	168590889	DNM3	1.32	1.15	0.33
A_16_P15341410	168605141	168605200	DNM3	1.29	0.59	0.29
A_16_P15341437	168615327	168615386	DNM3	0.94	1.02	-0.03
A_14_P104759	168622525	168622576	DNM3	0.37	1.36	0.04
A_16_P15341465	168632425	168632484	DNM3	0.21	0.95	0.24
A_16_P35356915	168640257	168640316	DNM3	0.86	0.81	0.19
A_16_P15341505	168646117	168646176	DNM3	0.71	1.38	0.32
A_16_P15341521	168653894	168653953	DNM3	1.17	1.01	0.35
A_16_P00191500	168660110	168660169	DNM3	0.91	0.88	0.31
A_16_P15341539	168667946	168668005	DNM3	0.94	1.39	0.40
A_16_P15341557	168676060	168676119	DNM3	0.89	1.22	0.30
A_16_P15341592	168689945	168690004	DNM3	0.83	1.42	0.40
A_16_P35357041	168702232	168702291	DNM3	1.16	0.55	0.20
A_16_P15341637	168714094	168714153	DNM3	0.73	1.01	0.23
A_16_P15341641	168723128	168723187	DNM3	0.46	0.59	0.18

A_16_P35357071	168733124	168733183	DNM3	1.17	2.10	0.37
A_14_P113743	168742778	168742837	DNM3	1.12	1.08	0.37
A_16_P00191594	168751043	168751102	DNM3	1.33	0.66	0.38
A_16_P15341708	168762659	168762718	DNM3	0.81	1.04	0.23
A_16_P15341731	168769984	168770043	DNM3	1.04	0.80	0.25
A_16_P35357175	168782593	168782652	DNM3	0.99	0.95	0.31
A_16_P00191643	168793643	168793702	DNM3	1.49	1.48	0.45
A_16_P00191654	168800810	168800869	DNM3	1.40	1.43	0.41
A_16_P35357242	168808626	168808685	DNM3	0.77	0.87	0.09
A_16_P35357256	168812476	168812535	DNM3	1.24	1.17	0.44
A_16_P15341847	168818982	168819041	DNM3	1.08	1.35	0.43
A_16_P35357284	168822863	168822922	DNM3	0.69	1.00	0.21
A_16_P00191704	168828118	168828177	DNM3	1.07	1.38	0.37
A_16_P15341886	168832476	168832535	DNM3	1.33	1.29	0.45
A_16_P15341894	168835124	168835183	DNM3	1.11	0.11	0.46
A_16_P15341907	168838760	168838819	DNM3	1.38	0.71	0.30
A_16_P15341916	168841328	168841387	DNM3	0.58	1.42	0.25
A_16_P00191740	168845243	168845302	DNM3	1.44	1.23	0.29
A_16_P15341940	168847819	168847878	DNM3	0.74	0.92	0.36
A_16_P15341952	168850945	168851004	DNM3	0.52	1.31	0.41
A_16_P15341970	168855977	168856036	DNM3	0.95	0.98	0.12
A_14_P120239	168862261	168862320	DNM3	1.07	0.56	0.24
A_16_P00191779	168865383	168865442	DNM3	1.04	0.68	0.32
A_16_P15342001	168869889	168869948	DNM3	0.86	0.97	0.36
A_16_P35357452	168875492	168875551	DNM3	0.87	1.01	0.37
A_16_P35357479	168883245	168883304	DNM3	0.92	0.51	0.19
A_16_P00191828	168888800	168888859	DNM3	1.15	1.29	0.35
A_16_P15342067	168903611	168903670	DNM3	1.11	2.01	0.42
A_16_P15342099	168913275	168913334	DNM3	0.52	1.57	0.12
A_16_P15342136	168924824	168924883	DNM3	1.48	1.09	0.54
A_16_P15342148	168930289	168930348	DNM3	0.71	1.47	0.65
A_16_P00191881	168936898	168936957	DNM3	1.37	0.80	0.46
A_16_P00191894	168942783	168942842	DNM3	1.23	1.23	0.50
A_14_P105281	168954432	168954491	DNM3	0.49	0.34	0.36
A_16_P35357669	168967700	168967759	DNM3	1.07	1.23	0.30
A_16_P15342256	168976284	168976343	DNM3	0.97	0.55	0.32
A_16_P35357720	168985515	168985574	DNM3	1.11	1.25	0.30
A_16_P15342303	168993597	168993656	DNM3	0.76	1.38	0.21
A_16_P35357773	169013010	169013069	DNM3	1.08	1.31	0.48
A_16_P35357783	169022561	169022620	DNM3	1.15	2.05	0.51
A_16_P00192015	169029100	169029159	DNM3	0.55	0.99	0.31
A_16_P15342401	169039979	169040038	DNM3	0.89	1.61	0.57
A_16_P15342414	169045503	169045562	DNM3	0.45	0.22	0.48
A_16_P35357880	169054722	169054781	DNM3	0.79	1.12	0.16
A_16_P00192075	169064997	169065056	DNM3	0.96	1.11	0.46
A_16_P15342477	169072037	169072096	DNM3	1.16	0.27	0.41
A_16_P15342504	169079895	169079954	DNM3	1.55	1.09	0.42
A_16_P35357966	169087893	169087952	DNM3	1.00	0.82	0.27

A_14_P202086	169094039	169094098	DNM3	1.25	1.79	0.42
A_14_P111508	169098021	169098080	DNM3	0.77	0.96	0.31
A_16_P15342607	169109616	169109675	DNM3	1.06	1.12	0.45
A_16_P15342648	169121487	169121546	C1orf105	1.33	1.00	0.45
A_16_P15342663	169130169	169130228	C1orf105	1.40	1.11	0.31
A_16_P35358114	169142211	169142270	C1orf105	0.97	1.77	0.14
A_14_P125792	169144031	169144090	C1orf105	1.64	1.76	0.56
A_16_P35358147	169149254	169149313	C1orf105	0.70	0.65	0.27
A_16_P35358169	169155878	169155937	C1orf105	1.20	1.06	0.43
A_14_P108915	169164446	169164505	C1orf105	0.58	0.48	0.19
A_16_P15342767	169169470	169169529	C1orf105	0.81	0.70	0.23
A_16_P15342805	169182646	169182705	chr1:169182646-169182705	1.35	0.65	0.49
A_16_P35358279	169192281	169192340	chr1:169192281-169192340	1.19	1.05	0.42
A_16_P35358327	169206279	169206338	chr1:169206279-169206338	1.19	1.28	0.40
A_16_P35358368	169219011	169219070	chr1:169219011-169219070	1.58	0.95	0.28
A_16_P15342931	169227268	169227327	chr1:169227268-169227327	0.31	0.65	0.41
A_14_P109246	169233474	169233528	C1orf9	1.22	0.95	0.43
A_16_P35358439	169244891	169244950	C1orf9	0.99	0.77	0.34
A_16_P15342995	169250994	169251053	C1orf9	0.75	0.48	0.24
A_14_P112373	169258441	169258500	C1orf9	0.84	1.03	0.45
A_16_P00192444	169276383	169276442	C1orf9	0.78	1.20	0.33
A_16_P35358543	169282822	169282881	C1orf9	0.88	0.61	0.21
A_14_P102429	169291783	169291842	C1orf9	0.74	1.60	0.14
A_16_P35358591	169302980	169303039	C1orf9	1.10	0.73	0.37
A_14_P137383	169311583	169311642	C1orf9	0.92	1.57	0.48
A_16_P00192515	169329561	169329620	chr1:169329561-169329620	1.03	0.91	0.62
A_16_P00192533	169342049	169342108	chr1:169342049-169342108	1.19	1.87	0.47
A_16_P15343223	169348853	169348912	chr1:169348853-169348912	1.23	1.49	0.31
A_16_P15343238	169355793	169355852	chr1:169355793-169355852	1.20	2.42	0.28
A_14_P119518	169360958	169361017	FASLG	1.14	0.67	0.49
A_14_P100759	169366186	169366245	FASLG	1.08	1.36	0.30
A_14_P105867	169366838	169366897	FASLG	0.81	0.99	0.03
A_16_P15343298	169374867	169374926	chr1:169374867-169374926	1.11	0.39	-0.09
A_16_P00192605	169384000	169384059	chr1:169384000-169384059	0.96	0.98	0.44
A_14_P125959	169399217	169399276	chr1:169399217-169399276	1.01	0.44	0.38
A_16_P35358890	169424590	169424649	chr1:169424590-169424649	0.70	1.17	0.20
A_16_P15343497	169460567	169460626	chr1:169460567-169460626	0.98	0.99	0.49
A_16_P15343572	169491281	169491340	chr1:169491281-169491340	0.77	1.37	0.17
A_16_P15343583	169518266	169518325	chr1:169518266-169518325	0.74	0.58	0.23
A_16_P15343655	169544660	169544719	chr1:169544660-169544719	0.88	1.23	0.27
A_16_P00192819	169568157	169568216	chr1:169568157-169568216	1.09	-0.26	0.41
A_16_P00192831	169582882	169582941	chr1:169582882-169582941	1.24	1.25	0.46
A_16_P35359236	169600440	169600499	chr1:169600440-169600499	1.12	1.44	0.28
A_14_P108213	169619285	169619344	chr1:169619285-169619344	0.86	1.16	0.32
A_16_P00192941	169649165	169649224	chr1:169649165-169649224	1.42	1.52	0.31
A_16_P00192976	169670992	169671051	chr1:169670992-169671051	1.20	1.50	0.37
A_16_P35359476	169698887	169698946	chr1:169698887-169698946	1.13	1.58	0.38
A_16_P35359512	169721280	169721339	chr1:169721280-169721339	0.80	1.10	0.44

A_16_P35359518	169730632	169730691	chr1:169730632-169730691	0.78	0.21	0.37
A_16_P00193035	169737341	169737400	chr1:169737341-169737400	1.30	0.89	0.32
A_14_P202512	169742367	169742426	TNFSF18	0.16	1.20	0.24
A_14_P135207	169744675	169744734	TNFSF18	0.62	1.42	-0.52
A_14_P202112	169749119	169749178	TNFSF18	1.22	0.64	0.28
A_16_P15344116	169756461	169756520	chr1:169756461-169756520	0.91	0.58	0.15
A_16_P15344135	169762343	169762402	chr1:169762343-169762402	1.55	1.66	0.49
A_16_P15344144	169787705	169787764	chr1:169787705-169787764	0.75	1.18	0.10
A_16_P00193119	169812994	169813053	chr1:169812994-169813053	1.52	1.76	0.34
A_16_P15344246	169839736	169839795	chr1:169839736-169839795	0.81	0.83	0.07
A_16_P00193172	169864853	169864912	chr1:169864853-169864912	0.86	1.02	0.37
A_16_P15344302	169881617	169881676	chr1:169881617-169881676	0.72	1.18	0.30
A_14_P139797	169886582	169886641	TNFSF4	1.25	0.66	0.36
A_16_P15344349	169894871	169894930	TNFSF4	0.95	0.20	0.28
A_14_P112470	169905193	169905252	TNFSF4	1.09	0.88	0.30
A_14_P138781	169906082	169906133	TNFSF4	0.38	0.32	0.66
A_16_P35359885	169910668	169910727	chr1:169910668-169910727	1.33	1.14	0.35
A_16_P00193257	169919078	169919137	chr1:169919078-169919137	0.77	0.94	0.19
A_14_P139652	169927231	169927290	chr1:169927231-169927290	0.34	0.73	0.24
A_16_P15344480	169947656	169947715	AK127238	1.27	0.88	0.20
A_16_P35359993	169957891	169957950	AK127238	1.28	0.03	0.28
A_16_P35360003	169969757	169969816	AK127238	0.61	0.83	0.27
A_16_P00193336	169984157	169984216	AK127238	1.51	1.18	0.33
A_16_P15344585	170003678	170003737	AK127238	0.72	0.82	0.27
A_16_P15344607	170014212	170014271	AK127238	0.61	1.47	0.16
A_16_P15344634	170033794	170033853	AK127238	1.19	1.16	0.38
A_16_P35360129	170045626	170045685	AK127238	0.73	1.28	0.25
A_16_P15344697	170062713	170062772	AK127238	0.82	0.61	0.28
A_16_P15344712	170083441	170083500	AK127238	1.20	1.17	0.26
A_16_P00193431	170092422	170092481	AK127238	0.97	0.42	0.20
A_16_P15344747	170104291	170104350	AK127238	1.39	0.79	0.36
A_14_P115379	170114673	170114732	AK127238	1.36	0.99	0.44
A_16_P15344813	170131267	170131325	LOC646870	1.64	2.00	0.66
A_16_P15344865	170153129	170153188	LOC646870	0.48	0.21	0.44
A_16_P00193517	170168193	170168252	AK127238	1.01	1.10	0.22
A_16_P15344889	170173043	170173102	AK127238	0.51	0.86	0.18
A_16_P00193532	170180783	170180842	PRDX6	0.97	0.54	0.19
A_14_P114905	170188979	170189038	PRDX6	0.76	1.65	0.01
A_16_P00193554	170193277	170193335	chr1:170193277-170193335	1.34	1.07	0.37
A_16_P35360428	170201829	170201888	SLC9A11	0.86	0.04	0.19
A_16_P35360451	170207691	170207750	SLC9A11	1.27	0.99	0.52
A_14_P129071	170222106	170222165	SLC9A11	1.07	1.11	0.36
A_16_P00193605	170234491	170234550	SLC9A11	0.94	0.72	0.43
A_16_P35360544	170249181	170249240	SLC9A11	1.02	0.72	0.57
A_14_P124569	170258206	170258265	SLC9A11	1.09	0.57	0.34
A_14_P138744	170277475	170277534	SLC9A11	1.38	0.81	0.36
A_16_P35360641	170287018	170287077	SLC9A11	0.59	0.37	0.36
A_14_P134179	170302599	170302658	SLC9A11	1.35	1.10	0.35

A_16_P35360697	170310839	170310898	ANKRD45	0.52	1.38	0.39
A_16_P35360705	170317428	170317487	ANKRD45	0.97	0.96	0.22
A_16_P35360707	170325556	170325615	ANKRD45	0.48	0.89	0.29
A_16_P15345208	170337640	170337699	ANKRD45	1.14	0.76	0.59
A_14_P134250	170347522	170347581	ANKRD45	0.93	1.12	0.20
A_16_P35360758	170359844	170359903	ANKRD45	0.30	0.41	-0.06
A_16_P15345278	170374614	170374673	chr1:170374614-170374673	0.86	0.88	0.27
A_16_P00193754	170394580	170394639	chr1:170394580-170394639	0.92	1.63	0.60
A_16_P15345290	170402746	170402805	chr1:170402746-170402805	0.89	1.31	0.36
A_16_P35360815	170412575	170412634	chr1:170412575-170412634	1.34	1.58	0.40
A_16_P35360836	170421247	170421306	KLHL20	1.31	1.26	0.54
A_14_P114892	170433259	170433318	KLHL20	0.02	1.78	0.43
A_16_P00193787	170443189	170443248	KLHL20	1.36	0.76	0.43
A_16_P15345347	170449811	170449870	KLHL20	1.10	0.99	0.46
A_14_P114833	170456689	170456748	KLHL20	1.03	1.25	0.23
A_16_P35360901	170467058	170467117	KLHL20	0.17	-0.65	0.31
A_16_P15345389	170475037	170475096	KLHL20	0.84	0.84	0.07
A_14_P128697	170486013	170486072	KLHL20	0.94	0.48	0.32
A_16_P15345422	170492693	170492752	chr1:170492693-170492752	1.19	1.42	0.45
A_16_P35360976	170504248	170504307	C1orf155	0.90	1.10	0.29
A_16_P15345460	170519011	170519070	C1orf155	0.90	0.52	0.52
A_16_P00193883	170529119	170529178	DARS2	1.10	1.21	0.51
A_14_P118568	170537563	170537622	DARS2	1.04	1.53	0.34
A_16_P35361095	170551091	170551150	DARS2	0.70	1.26	0.17
A_14_P133617	170558282	170558341	DARS2	1.25	0.79	0.29
A_16_P00193930	170564301	170564360	AK025846	0.84	0.69	0.33
A_14_P105924	170569584	170569630	ZBTB37	1.26	0.62	0.51
A_14_P200047	170572463	170572522	ZBTB37	1.25	1.36	0.38
A_16_P15345621	170578091	170578150	AK057310	1.22	1.44	0.42
A_14_P104276	170586683	170586727	AK057310	1.32	1.23	0.46
A_16_P15345659	170591421	170591480	chr1:170591421-170591480	1.45	1.31	0.93
A_16_P15345675	170596575	170596634	chr1:170596575-170596634	1.19	0.84	0.98
A_16_P35361267	170604704	170604763	SERPINC1	1.31	1.00	0.26
A_14_P128174	170615914	170615973	SERPINC1	1.12	1.13	0.55
A_16_P15345758	170626731	170626790	AF130100	1.08	1.05	0.40
A_16_P15345792	170639473	170639532	RC3H1	1.22	1.38	0.44
A_16_P15345813	170647498	170647557	RC3H1	1.14	0.25	0.21
A_16_P15345842	170658908	170658967	RC3H1	0.46	0.72	0.22
A_16_P15345856	170665890	170665949	RC3H1	0.66	1.17	0.19
A_16_P35361485	170681571	170681630	RC3H1	1.32	1.03	0.49
A_16_P15345921	170693994	170694053	AF130100	0.95	1.08	0.38
A_16_P15345937	170700377	170700436	AF130100	0.71	0.53	0.40
A_16_P15345953	170707064	170707123	AF130100	0.91	1.24	0.40
A_16_P00194184	170718676	170718735	AF130100	1.11	1.52	0.38
A_14_P123624	170733820	170733879	AF130100	1.00	1.32	0.30
A_16_P15346057	170747850	170747909	AF130100	1.17	0.14	0.51
A_16_P15346075	170764908	170764967	AF130100	0.64	0.72	0.24
A_16_P15346084	170774410	170774469	AF130100	0.82	0.86	0.25

A_16_P00194245	170791204	170791263	AF130100	0.85	1.24	0.24
A_16_P15346113	170804181	170804240	AF130100	2.01	1.94	0.94
A_16_P15346131	170817323	170817382	AF130100	0.66	1.29	0.25
A_16_P15346153	170839482	170839541	AF130100	1.03	1.20	0.49
A_14_P101071	170856572	170856631	AF130100	1.20	1.38	0.32
A_16_P35361795	170868384	170868443	RABGAP1L	1.27	1.60	0.53
A_16_P15346221	170874262	170874321	RABGAP1L	1.24	1.14	0.45
A_16_P15346244	170887424	170887483	RABGAP1L	0.75	1.77	0.32
A_16_P15346262	170895668	170895727	RABGAP1L	0.96	0.86	0.45
A_16_P15346276	170901480	170901539	RABGAP1L	0.88	1.27	0.28
A_16_P15346283	170907329	170907388	RABGAP1L	0.87	0.52	0.30
A_14_P125466	170919996	170920055	RABGAP1L	0.84	0.83	0.42
A_16_P15346308	170929168	170929227	RABGAP1L	0.75	0.66	0.37
A_16_P35361915	170942261	170942320	RABGAP1L	0.99	1.22	0.38
A_16_P15346352	170951426	170951485	RABGAP1L	1.43	1.58	0.86
A_16_P15346383	170964579	170964638	RABGAP1L	0.63	0.61	0.20
A_16_P35361998	170973254	170973313	RABGAP1L	0.92	1.12	0.54
A_16_P00194435	170979540	170979599	RABGAP1L	0.95	0.90	0.42
A_16_P00194444	170997289	170997348	RABGAP1L	0.73	1.14	0.39
A_16_P35362059	171007717	171007776	RABGAP1L	0.34	0.64	0.11
A_16_P35362064	171024684	171024743	RABGAP1L	1.03	1.48	0.48
A_16_P35362076	171036509	171036568	RABGAP1L	0.80	0.62	0.22
A_16_P15346496	171042620	171042679	RABGAP1L	1.39	0.95	0.36
A_16_P00194484	171058029	171058088	RABGAP1L	0.96	0.32	0.37
A_14_P127684	171071823	171071882	RABGAP1L	0.99	1.12	0.55
A_16_P15346564	171085459	171085518	RABGAP1L	0.76	1.44	0.14
A_16_P35362178	171092695	171092754	RABGAP1L	0.78	0.73	0.30
A_16_P00194532	171098371	171098430	RABGAP1L	1.03	1.14	0.27
A_16_P00194546	171105678	171105737	RABGAP1L	0.65	0.98	0.16
A_16_P15346635	171117146	171117205	RABGAP1L	0.65	1.11	0.26
A_16_P15346684	171132144	171132203	RABGAP1L	1.00	0.37	0.39
A_16_P15346703	171143264	171143323	RABGAP1L	0.25	1.54	0.24
A_14_P109034	171149245	171149304	RABGAP1L	1.40	0.79	0.42
A_14_P201439	171149893	171149941	RABGAP1L	1.41	0.14	0.46
A_16_P15346731	171153711	171153770	RABGAP1L	0.73	1.32	0.16
A_16_P00194631	171159200	171159259	RABGAP1L	0.83	0.89	0.32
A_16_P00194650	171170814	171170873	RABGAP1L	0.67	-0.77	0.12
A_16_P35362405	171179539	171179598	RABGAP1L	0.99	1.63	0.37
A_16_P15346826	171194179	171194238	RABGAP1L	0.55	0.78	0.31
A_16_P15346846	171199825	171199884	RABGAP1L	0.49	1.57	0.11
A_16_P35362458	171208887	171208946	RABGAP1L	1.03	1.49	0.31
A_16_P15346906	171221287	171221346	RABGAP1L	0.63	0.71	0.04
A_16_P35362518	171239194	171239253	RABGAP1L	0.85	0.86	0.27
A_16_P15346957	171255759	171255818	RABGAP1L	0.86	0.93	0.35
A_16_P15346983	171264496	171264555	RABGAP1L	0.50	0.58	0.20
A_16_P15347012	171275189	171275248	RABGAP1L	0.49	0.04	-0.01
A_16_P15347041	171288288	171288347	RABGAP1L	0.01	0.75	0.04
A_16_P15347044	171307144	171307203	RABGAP1L	0.96	0.69	0.29

A_16_P35362655	171318141	171318200	RABGAP1L	0.97	1.16	0.46
A_16_P15347083	171329967	171330026	RABGAP1L	0.93	1.40	0.30
A_14_P115147	171338146	171338205	RABGAP1L	0.65	0.59	0.42
A_16_P00194841	171344073	171344132	RABGAP1L	0.64	0.90	0.32
A_16_P15347119	171354536	171354595	RABGAP1L	0.04	-1.34	-0.06
A_16_P15347131	171362163	171362222	RABGAP1L	0.33	0.53	0.13
A_16_P15347139	171367657	171367716	RABGAP1L	1.03	0.45	0.53
A_16_P15347153	171377306	171377365	RABGAP1L	0.63	1.06	0.20
A_16_P15347174	171388394	171388453	RABGAP1L	0.68	0.85	0.32
A_16_P35362797	171400421	171400480	RABGAP1L	0.84	1.14	0.12
A_16_P00194906	171408072	171408131	RABGAP1L	0.73	0.59	0.30
A_16_P00194911	171415766	171415825	RABGAP1L	0.54	0.84	0.09
A_16_P15347262	171424260	171424319	RABGAP1L	0.85	0.54	0.54
A_16_P35362884	171435359	171435418	RABGAP1L	1.11	1.17	0.35
A_16_P15347299	171441146	171441203	RABGAP1L	0.51	0.67	0.02
A_16_P15347324	171451859	171451918	RABGAP1L	0.64	1.17	0.22
A_16_P00194975	171458927	171458986	RABGAP1L	0.99	0.54	0.32
A_16_P15347362	171474253	171474312	RABGAP1L	1.02	-0.06	0.43
A_16_P35362964	171479772	171479831	RABGAP1L	0.56	0.62	0.16
A_16_P15347381	171486245	171486304	RABGAP1L	0.29	0.12	0.27
A_16_P15347400	171492810	171492869	RABGAP1L	1.47	0.66	0.29
A_14_P201699	171500908	171500966	RABGAP1L	-0.10	0.34	0.09
A_14_P105147	171503945	171504004	RABGAP1L	1.31	0.54	0.34
A_16_P15347470	171516082	171516141	RABGAP1L	0.98	1.05	0.36
A_16_P15347498	171526031	171526090	RABGAP1L	1.18	0.97	0.44
A_14_P134427	171537634	171537693	RABGAP1L	1.47	1.08	0.39
A_16_P15347560	171552956	171553015	RABGAP1L	0.64	1.86	0.18
A_16_P15347608	171569720	171569779	RABGAP1L	0.99	1.17	0.41
A_16_P15347630	171577284	171577343	RABGAP1L	0.37	0.60	0.06
A_16_P00195155	171585654	171585713	RABGAP1L	1.10	0.86	0.41
A_14_P131958	171594570	171594629	RABGAP1L	0.61	0.96	0.19
A_16_P35363288	171600472	171600531	RABGAP1L	0.75	1.69	0.25
A_16_P15347705	171617106	171617165	RABGAP1L	0.96	0.82	0.30
A_14_P133470	171629698	171629757	RABGAP1L	0.61	1.11	0.07
A_16_P15347771	171645995	171646054	RABGAP1L	0.50	0.23	0.12
A_14_P112458	171658140	171658199	RABGAP1L	1.15	1.14	0.32
A_16_P00195259	171666229	171666288	AK095838	0.80	0.67	0.25
A_16_P15347870	171678274	171678333	AK095838	1.54	1.22	0.38
A_16_P15347905	171689389	171689448	AB019493	1.08	1.11	0.32
A_14_P125407	171693506	171693565	AB007940	0.25	0.33	0.26
A_16_P15347942	171702040	171702099	CACYBP	0.69	1.29	0.46
A_14_P125159	171707517	171707575	CACYBP	0.84	1.04	0.23
A_16_P35363590	171711612	171711671	CACYBP	0.91	0.89	0.26
A_14_P137189	171714876	171714935	MRPS14	0.41	0.39	0.04
A_14_P201931	171719206	171719265	MRPS14	0.44	0.62	0.12
A_16_P15347997	171724200	171724254	chr1:171724200-171724254	1.57	0.17	0.53
A_16_P15348008	171730022	171730081	chr1:171730022-171730081	0.55	0.90	0.22
A_16_P00195382	171735833	171735892	chr1:171735833-171735892	1.49	1.11	0.46

A_16_P15348054	171745140	171745199	chr1:171745140-171745199	1.22	1.03	0.38
A_16_P15348075	171753438	171753497	chr1:171753438-171753497	0.93	0.63	0.46
A_16_P15348087	171761566	171761625	chr1:171761566-171761625	0.88	1.13	0.00
A_16_P00195425	171767801	171767858	chr1:171767801-171767858	1.59	1.63	0.32
A_16_P15348134	171776459	171776518	TNN	1.45	1.24	0.62
A_16_P00195471	171789229	171789288	TNN	0.61	1.07	0.21
A_16_P00195480	171794757	171794816	TNN	0.71	0.55	0.34
A_16_P35363841	171801447	171801506	TNN	0.93	1.25	0.36
A_16_P15348215	171817572	171817631	TNN	1.00	0.38	0.33
A_16_P15348232	171825865	171825924	TNN	0.94	0.68	0.48
A_16_P35363883	171832102	171832161	TNN	1.03	1.14	0.20
A_16_P00195528	171837667	171837726	TNN	1.02	0.58	0.13
A_16_P00195539	171845188	171845247	TNN	1.36	1.05	0.18
A_16_P00195553	171852649	171852708	chr1:171852649-171852708	1.33	1.18	0.48
A_16_P00195564	171858274	171858333	KIAA0040	1.03	1.50	0.61
A_14_P123553	171865764	171865823	KIAA0040	0.68	0.11	0.41
A_16_P00195597	171873047	171873106	KIAA0040	1.04	0.69	0.39
A_16_P00195612	171880456	171880515	KIAA0040	1.13	1.05	0.38
A_14_P106401	171890938	171890997	KIAA0040	1.25	1.47	0.32
A_14_P125573	171908030	171908089	chr1:171908030-171908089	0.94	0.79	0.28
A_16_P35364158	171917087	171917146	chr1:171917087-171917146	0.97	1.31	0.30
A_16_P00195710	171950250	171950309	chr1:171950250-171950309	1.08	0.87	0.17
A_16_P35364260	172000033	172000092	chr1:172000033-172000092	0.96	1.21	0.32
A_16_P35364273	172011319	172011378	chr1:172011319-172011378	1.22	0.98	0.33
A_16_P15348660	172019216	172019275	AB007979	0.98	0.75	0.35
A_16_P35364331	172028157	172028216	TNR	1.27	0.49	0.16
A_16_P00195794	172035817	172035876	TNR	1.28	1.19	0.40
A_14_P134510	172042030	172042089	TNR	0.63	2.13	0.56
A_16_P15348773	172057246	172057305	TNR	1.48	1.03	0.41
A_14_P111048	172064472	172064531	TNR	1.19	1.21	0.27
A_16_P15348822	172073555	172073611	TNR	0.83	0.47	0.27
A_16_P00195877	172079143	172079202	TNR	0.70	1.09	0.29
A_16_P15348863	172087089	172087148	TNR	0.73	0.50	0.35
A_16_P00195924	172103712	172103771	TNR	0.22	-0.68	-0.02
A_16_P00195949	172116526	172116585	X98085	1.27	1.32	0.11
A_16_P15349003	172127437	172127496	X98085	0.73	0.50	0.00
A_16_P00195996	172144879	172144938	X98085	1.16	-0.19	0.27
A_14_P125667	172161337	172161396	X98085	0.79	1.25	0.02
A_16_P00196069	172180504	172180562	X98085	0.99	0.60	0.19
A_16_P35364851	172193034	172193093	X98085	0.99	1.12	0.27
A_16_P00196121	172210539	172210598	X98085	0.52	0.67	0.35
A_14_P122300	172229058	172229117	X98085	1.33	1.41	0.27
A_14_P125639	172229243	172229302	Y13359	0.60	1.53	0.20
A_16_P15349354	172243459	172243518	Y13359	0.66	1.51	0.22
A_16_P00196207	172256102	172256161	Y13359	1.13	1.04	0.30
A_16_P00196223	172267099	172267158	Y13359	1.26	0.71	0.35
A_16_P00196250	172278587	172278646	Y13359	0.92	0.76	0.41
A_16_P00196284	172296413	172296472	Y13359	0.82	0.70	0.23

A_16_P00196323	172316629	172316688	Y13359	1.06	1.17	0.14
A_16_P00196384	172347031	172347090	Y13359	0.80	0.86	0.20
A_16_P15349689	172356033	172356092	Y13359	0.99	1.12	0.39
A_16_P00196427	172370347	172370406	Y13359	1.64	1.54	0.35
A_16_P15349781	172382944	172383003	Y13359	0.99	0.85	0.30
A_16_P15349812	172392724	172392783	Y13359	0.68	1.00	0.19
A_16_P15349837	172403759	172403818	Y13359	1.17	1.21	0.27
A_14_P110029	172414492	172414551	Y13359	0.58	0.71	0.32
A_16_P35365564	172423933	172423992	Y13359	1.03	1.17	0.37
A_16_P15349927	172436400	172436459	Y13359	0.39	0.59	0.05
A_16_P00196620	172474835	172474894	chr1:172474835-172474894	1.23	0.76	0.43
A_16_P00196657	172497794	172497853	chr1:172497794-172497853	0.84	1.44	0.20
A_16_P35365851	172527923	172527982	chr1:172527923-172527982	0.90	0.81	0.19
A_16_P35365930	172559307	172559366	chr1:172559307-172559366	1.16	1.48	0.09
A_16_P15350302	172575470	172575529	chr1:172575470-172575529	1.53	0.81	0.24
A_14_P102532	172582282	172582341	chr1:172582282-172582341	1.01	1.92	0.25
A_16_P35366075	172606415	172606474	chr1:172606415-172606474	0.48	0.43	0.17
A_16_P00196855	172621484	172621543	chr1:172621484-172621543	1.51	1.14	0.36
A_16_P00196863	172641629	172641688	chr1:172641629-172641688	0.30	1.47	0.10
A_16_P15350466	172652087	172652146	RFWD2	0.96	-0.22	0.04
A_16_P00196894	172662588	172662647	RFWD2	0.91	0.90	0.66
A_16_P15350515	172672613	172672672	RFWD2	0.82	0.97	0.32
A_16_P35366228	172681763	172681822	RFWD2	0.77	1.14	0.09
A_14_P122124	172690135	172690186	RFWD2	0.99	0.85	0.32
A_16_P35366248	172690365	172690424	RFWD2	0.47	0.66	-0.05
A_16_P15350568	172710100	172710159	RFWD2	0.34	-0.17	-0.98
A_16_P15350568	172710100	172710159	RFWD2	-0.11	-0.05	0.34
A_16_P15350568	172710100	172710159	RFWD2	-0.40	0.59	-0.78
A_16_P35366276	172718053	172718112	RFWD2	0.70	0.80	0.32
A_14_P114536	172726886	172726945	RFWD2	0.32	1.34	0.18
A_16_P35366327	172741926	172741985	RFWD2	0.82	1.16	0.23
A_14_P122616	172746944	172747003	RFWD2	0.51	0.04	0.01
A_16_P15350673	172753341	172753400	RFWD2	0.81	0.06	0.08
A_14_P139604	172765296	172765355	RFWD2	0.43	-0.44	0.03
A_16_P00197016	172770819	172770878	RFWD2	0.60	0.53	0.27
A_16_P35366414	172781908	172781967	RFWD2	0.95	0.84	0.31
A_16_P15350738	172789422	172789481	RFWD2	0.39	0.59	-0.03
A_14_P131930	172795079	172795138	RFWD2	0.41	1.28	0.13
A_16_P15350751	172804793	172804852	RFWD2	0.58	0.64	0.47
A_16_P15350769	172815051	172815110	RFWD2	0.71	-0.09	0.21
A_16_P15350795	172823664	172823723	RFWD2	0.44	0.58	0.06
A_16_P15350808	172830637	172830696	RFWD2	0.47	0.99	0.16
A_14_P105291	172837254	172837313	RFWD2	0.20	0.04	-0.03
A_16_P15350855	172848842	172848901	RFWD2	0.69	0.86	-0.01
A_16_P15350882	172863771	172863830	RFWD2	0.83	0.27	-0.02
A_16_P15350900	172870164	172870223	RFWD2	0.26	1.12	0.04
A_14_P132450	172876687	172876746	RFWD2	0.67	1.41	0.09
A_16_P15350950	172891813	172891872	RFWD2	1.18	0.81	0.35

A_16_P35366668	172902267	172902326	RFWD2	0.99	0.81	0.26
A_14_P108480	172910467	172910526	chr1:172910467-172910526	1.19	1.80	0.50
A_16_P35366713	172917155	172917214	chr1:172917155-172917214	0.52	1.22	-0.09
A_16_P15351021	172924495	172924554	chr1:172924495-172924554	0.38	0.01	0.13
A_16_P15351032	172950455	172950514	chr1:172950455-172950514	0.49	1.35	0.31
A_16_P00197233	172975031	172975090	chr1:172975031-172975090	0.64	0.78	0.07
A_16_P00197245	173000565	173000624	chr1:173000565-173000624	1.36	0.90	0.24
A_16_P35366840	173029246	173029305	chr1:173029246-173029305	0.70	0.62	0.18
A_14_P125295	173075728	173075787	chr1:173075728-173075787	1.07	0.75	0.26
A_16_P00197346	173110462	173110521	chr1:173110462-173110521	0.98	0.70	0.29
A_16_P35366987	173134250	173134309	chr1:173134250-173134309	1.32	0.81	0.29
A_16_P15351334	173148073	173148132	chr1:173148073-173148132	1.26	0.73	0.26
A_14_P121406	173164164	173164222	PAPPA2	1.58	0.98	0.44
A_16_P00197426	173176993	173177052	PAPPA2	1.69	2.07	0.46
A_16_P15351423	173184591	173184639	PAPPA2	1.00	1.41	0.44
A_16_P15351436	173191098	173191157	PAPPA2	0.90	1.04	0.05
A_16_P00197465	173200502	173200561	PAPPA2	1.08	1.42	0.34
A_16_P15351490	173215463	173215522	PAPPA2	0.96	0.81	0.27
A_16_P00197505	173226072	173226131	PAPPA2	0.60	1.08	0.05
A_16_P00197518	173231739	173231798	PAPPA2	0.71	0.51	0.26
A_16_P15351567	173238175	173238234	PAPPA2	0.89	0.82	0.38
A_16_P15351591	173245006	173245065	PAPPA2	0.63	1.25	0.37
A_14_P104369	173256544	173256603	PAPPA2	0.91	1.74	0.28
A_16_P15351683	173274495	173274554	PAPPA2	0.66	1.37	0.41
A_16_P15351701	173280340	173280399	PAPPA2	1.56	1.05	0.41
A_16_P15351707	173290884	173290943	PAPPA2	0.95	0.85	0.40
A_16_P00197632	173296755	173296814	PAPPA2	1.38	1.43	0.40
A_16_P00197642	173304010	173304069	PAPPA2	0.58	0.15	0.13
A_16_P35367471	173310677	173310736	PAPPA2	0.97	1.18	0.29
A_14_P113662	173318864	173318923	PAPPA2	0.46	0.52	0.39
A_16_P00197689	173333089	173333148	PAPPA2	0.83	0.94	0.20
A_16_P00197702	173342767	173342826	PAPPA2	0.52	1.21	0.05
A_16_P15351864	173353988	173354047	PAPPA2	1.50	1.52	0.47
A_16_P15351883	173362476	173362535	PAPPA2	1.33	1.00	0.19
A_16_P15351897	173368489	173368548	PAPPA2	0.88	0.60	0.40
A_16_P15351921	173377694	173377753	PAPPA2	0.73	0.90	0.22
A_16_P00197755	173384687	173384746	PAPPA2	0.09	0.83	-0.11
A_16_P35367680	173391546	173391605	PAPPA2	0.74	0.80	0.07
A_14_P122670	173407125	173407184	PAPPA2	0.17	1.16	0.06
A_16_P15352040	173412590	173412649	PAPPA2	1.00	0.99	0.24
A_16_P15352058	173426175	173426234	PAPPA2	0.41	-0.09	0.15
A_16_P15352099	173440731	173440790	PAPPA2	0.99	1.35	0.19
A_16_P35367846	173449222	173449281	PAPPA2	1.01	0.99	0.12
A_16_P00197876	173455887	173455946	PAPPA2	1.11	0.45	0.28
A_16_P35367881	173462045	173462104	PAPPA2	0.97	0.89	0.42
A_16_P35367908	173470328	173470387	PAPPA2	1.31	1.31	0.36
A_16_P15352208	173479842	173479901	PAPPA2	0.94	1.11	0.31
A_16_P35367956	173486664	173486723	PAPPA2	1.48	0.98	0.38

A_16_P15352242	173492256	173492315	PAPPA2	0.25	1.19	0.09
A_16_P00197971	173505314	173505373	PAPPA2	1.43	0.97	0.39
A_16_P00197985	173512773	173512832	PAPPA2	0.95	-1.40	0.41
A_16_P15352325	173520048	173520107	PAPPA2	0.80	1.06	0.29
A_16_P15352348	173527775	173527834	PAPPA2	1.46	1.02	0.28
A_16_P00198025	173537527	173537586	PAPPA2	0.35	0.95	0.09
A_14_P114420	173543324	173543383	PAPPA2	1.90	1.37	0.10
A_16_P15352449	173556337	173556396	chr1:173556337-173556396	1.30	1.56	0.19
A_16_P00198075	173562303	173562360	ASTN	0.78	0.56	0.46
A_16_P00198092	173569595	173569654	ASTN	0.79	0.98	0.17
A_16_P15352542	173585522	173585581	ASTN	1.12	0.98	0.43
A_16_P15352574	173595624	173595683	ASTN	1.37	1.47	0.24
A_14_P127222	173613621	173613680	ASTN	-0.50	1.33	0.10
A_16_P00198196	173623938	173623997	ASTN	1.04	1.72	0.18
A_16_P15352682	173632862	173632921	ASTN	0.67	0.66	0.28
A_16_P35368469	173644431	173644490	ASTN	0.58	1.04	0.34
A_16_P00198250	173651621	173651680	ASTN	1.26	1.06	0.25
A_16_P15352768	173658280	173658339	ASTN	0.49	0.87	0.09
A_16_P15352796	173666625	173666684	ASTN	0.66	0.93	0.22
A_16_P00198307	173679350	173679409	ASTN	1.12	0.97	0.47
A_16_P35368621	173689264	173689323	ASTN	0.88	1.01	0.36
A_16_P15352880	173694658	173694717	ASTN	0.48	0.87	0.08
A_16_P00198346	173703025	173703084	ASTN	1.07	0.47	0.33
A_16_P35368663	173707228	173707287	ASTN	0.70	0.64	0.18
A_16_P15352920	173712000	173712059	ASTN	0.97	0.56	0.29
A_16_P15352935	173715770	173715829	ASTN	1.29	1.51	0.39
A_16_P15352950	173720104	173720163	ASTN	0.80	1.07	0.09
A_16_P00198385	173725209	173725268	ASTN	1.52	1.00	0.43
A_16_P35368749	173731884	173731943	ASTN	1.14	1.08	0.35
A_16_P00198406	173734728	173734787	ASTN	0.75	1.13	0.12
A_16_P15353009	173738554	173738613	ASTN	0.55	1.29	0.03
A_16_P15353023	173742458	173742517	ASTN	1.17	1.64	0.25
A_16_P00198430	173746180	173746238	ASTN	1.32	0.61	0.35
A_16_P00198430	173746180	173746238	ASTN	1.15	0.84	0.38
A_16_P00198430	173746180	173746238	ASTN	1.21	0.73	0.31
A_16_P00198441	173751219	173751278	ASTN	0.92	1.17	0.15
A_16_P35368835	173757633	173757692	ASTN	1.06	1.25	0.16
A_16_P00198475	173766543	173766602	ASTN	1.38	0.94	0.45
A_16_P15353133	173775335	173775394	ASTN	0.66	1.73	0.14
A_14_P118806	173788941	173789000	ASTN	1.00	1.32	0.35
A_16_P35368965	173799883	173799942	ASTN	0.53	1.70	0.16
A_16_P15353221	173807391	173807450	ASTN	1.09	0.66	0.25
A_16_P35369013	173816848	173816907	ASTN	1.16	0.93	0.23
A_16_P35369060	173829825	173829884	ASTN	1.13	0.40	0.25
A_16_P00198626	173844879	173844938	ASTN	0.88	0.54	0.40
A_16_P15353361	173851327	173851386	ASTN	0.79	0.90	0.30
A_16_P00198642	173860085	173860144	ASTN	0.75	1.02	0.17
A_14_P104389	173872424	173872472	FAM5B	0.05	0.64	0.35

A_16_P15353443	173878197	173878256	FAM5B	0.56	0.40	0.09
A_16_P15353479	173889665	173889724	FAM5B	1.02	0.13	0.14
A_16_P15353498	173895493	173895552	FAM5B	0.96	1.05	0.34
A_14_P125925	173907882	173907941	FAM5B	1.04	1.25	0.47
A_16_P15353546	173915284	173915343	FAM5B	0.83	0.53	0.31
A_14_P102687	173926779	173926838	FAM5B	0.87	1.07	0.45
A_16_P35369374	173935358	173935417	FAM5B	1.13	1.07	0.12
A_16_P35369394	173941043	173941102	FAM5B	1.10	1.03	0.43
A_14_P110958	173956811	173956861	FAM5B	0.64	0.29	0.46
A_16_P15353697	173974463	173974522	FAM5B	0.80	0.69	0.24
A_14_P121334	173983060	173983119	FAM5B	0.28	1.67	-0.01
A_16_P35369528	173989039	173989098	chr1:173989039-173989098	1.12	0.93	0.29
A_16_P15353762	174004970	174005029	chr1:174004970-174005029	0.90	0.81	0.23
A_16_P00198902	174019834	174019893	chr1:174019834-174019893	0.73	1.21	0.51
A_14_P102940	174060399	174060458	chr1:174060399-174060458	1.19	0.53	0.41
A_16_P00199015	174098156	174098214	chr1:174098156-174098214	1.48	1.51	0.42
A_16_P15354035	174122895	174122954	chr1:174122895-174122954	1.42	1.83	0.60
A_16_P15354109	174144061	174144120	chr1:174144061-174144120	1.07	0.99	0.35
A_16_P35369938	174167752	174167811	chr1:174167752-174167811	0.44	0.81	0.22
A_14_P114418	174187840	174187899	chr1:174187840-174187899	0.78	1.30	0.31
A_16_P00199173	174208778	174208837	chr1:174208778-174208837	1.06	1.54	0.43
A_16_P15354309	174229679	174229738	chr1:174229679-174229738	0.77	0.95	0.43
A_16_P35370098	174246303	174246362	chr1:174246303-174246362	1.33	1.20	0.35
A_16_P00199225	174268881	174268940	chr1:174268881-174268940	0.96	0.66	0.48
A_16_P15354397	174288520	174288579	chr1:174288520-174288579	1.08	0.55	0.36
A_16_P35370255	174317286	174317345	chr1:174317286-174317345	0.54	-0.32	0.22
A_16_P35370309	174347635	174347694	chr1:174347635-174347694	1.40	1.57	0.45
A_16_P15354593	174369277	174369336	chr1:174369277-174369336	0.92	0.80	0.28
A_14_P110303	174387073	174387132	chr1:174387073-174387132	0.71	1.20	0.43
A_16_P15354664	174409340	174409399	BC031234	1.06	1.21	0.47
A_16_P15354752	174453118	174453177	chr1:174453118-174453177	0.96	1.24	0.31
A_16_P00199536	174497772	174497831	chr1:174497772-174497831	1.04	0.65	0.52
A_14_P127594	174532015	174532074	chr1:174532015-174532074	0.19	0.36	0.17
A_16_P00199649	174559322	174559381	chr1:174559322-174559381	1.24	0.35	0.42
A_16_P15355098	174583731	174583790	chr1:174583731-174583790	1.18	1.57	0.40
A_16_P00199732	174613850	174613909	chr1:174613850-174613909	1.63	1.34	0.47
A_14_P134177	174629668	174629727	AK074451	0.20	0.83	0.17
A_16_P15355251	174639654	174639711	LZTR2	1.08	0.87	0.34
A_14_P137759	174649170	174649227	LZTR2	1.31	0.80	0.45
A_16_P00199815	174654791	174654850	LZTR2	0.59	1.46	0.41
A_14_P124053	174661750	174661809	LZTR2	1.26	1.62	0.26
A_16_P00199837	174668464	174668522	LZTR2	1.44	0.14	0.46
A_14_P116023	174680320	174680379	AB067515	1.40	1.05	0.37
A_16_P35371204	174695366	174695425	AB067515	1.16	0.55	0.29
A_14_P108182	174706517	174706576	AB067515	0.75	0.79	0.02
A_16_P35371243	174712703	174712762	AB067515	0.81	0.54	0.46
A_16_P35371262	174722713	174722772	AB067515	1.16	0.17	0.48
A_16_P15355486	174734016	174734075	AB067515	1.07	1.04	-0.26

A_14_P118008	174740553	174740612	chr1:174740553-174740612	1.00	1.32	0.23
A_16_P00199941	174753660	174753719	chr1:174753660-174753719	1.64	0.70	0.34
A_16_P15355552	174762664	174762723	chr1:174762664-174762723	1.22	0.67	0.36
A_16_P15355584	174776567	174776626	chr1:174776567-174776626	1.52	1.74	0.34
A_16_P15355597	174786089	174786148	chr1:174786089-174786148	0.58	-0.74	0.31
A_14_P125245	174796985	174797044	RASAL2	1.02	1.35	0.37
A_16_P35371463	174802707	174802766	RASAL2	1.13	0.99	0.29
A_16_P35371483	174809789	174809848	RASAL2	0.74	0.84	-0.44
A_16_P15355670	174817192	174817251	RASAL2	0.85	0.29	-0.44
A_16_P15355681	174823305	174823364	RASAL2	0.68	0.52	-0.59
A_16_P00200053	174832692	174832751	RASAL2	1.38	0.65	-0.32
A_16_P35371547	174839215	174839274	RASAL2	0.54	0.98	-0.44
A_16_P15355750	174849849	174849908	RASAL2	1.27	0.59	-0.29
A_16_P15355776	174861673	174861732	RASAL2	1.72	1.32	-0.15
A_16_P15355801	174872267	174872326	RASAL2	0.95	1.12	-0.25
A_16_P15355815	174877958	174878017	RASAL2	1.07	1.32	-0.03
A_16_P15355842	174893987	174894046	RASAL2	1.04	1.75	-0.42
A_16_P35371679	174905433	174905492	RASAL2	0.86	-0.06	-0.34
A_16_P15355872	174911993	174912051	RASAL2	0.25	0.16	-0.55
A_14_P105860	174928596	174928655	RASAL2	0.79	1.00	-0.38
A_16_P35371750	174937214	174937273	RASAL2	1.03	1.05	-0.05
A_16_P00200203	174944758	174944817	RASAL2	1.08	1.38	-0.34
A_16_P15355948	174952381	174952440	RASAL2	1.02	0.58	-0.15
A_16_P15355952	174958263	174958322	RASAL2	1.14	0.34	-0.39
A_16_P15355967	174963987	174964046	RASAL2	0.46	0.99	-0.51
A_16_P15355972	174969550	174969608	RASAL2	1.71	0.65	-0.24
A_16_P15356013	174984308	174984367	RASAL2	1.02	0.74	-0.29
A_16_P35371856	174993172	174993231	RASAL2	1.02	1.48	-0.48
A_16_P15356059	175002966	175003025	RASAL2	0.58	1.17	-0.29
A_16_P15356076	175011361	175011420	RASAL2	1.03	1.14	-0.24
A_16_P00200292	175024967	175025026	RASAL2	0.62	0.28	-0.58
A_16_P35371938	175030966	175031025	RASAL2	0.62	0.30	-0.62
A_16_P15356134	175039942	175040001	RASAL2	1.03	1.30	-0.43
A_16_P15356165	175049509	175049568	RASAL2	1.04	1.30	-0.30
A_16_P15356171	175058041	175058100	RASAL2	0.43	1.35	-0.40
A_16_P35372010	175064944	175065003	RASAL2	1.07	0.94	-0.51
A_16_P15356202	175071991	175072050	RASAL2	0.87	0.46	-0.29
A_16_P15356242	175084964	175085023	RASAL2	0.87	1.44	-0.29
A_16_P15356262	175091571	175091630	RASAL2	0.47	0.90	-0.35
A_14_P100729	175103681	175103740	RASAL2	1.01	0.76	-0.27
A_16_P15356320	175113349	175113408	RASAL2	0.89	1.51	-0.56
A_16_P15356328	175120416	175120475	RASAL2	1.35	1.27	-0.19
A_16_P15356351	175130233	175130292	RASAL2	0.91	1.18	-0.37
A_16_P00200472	175143678	175143737	RASAL2	1.06	1.22	-0.36
A_16_P15356415	175151668	175151727	RASAL2	1.20	2.13	-0.19
A_16_P15356436	175157682	175157741	RASAL2	1.33	1.20	-0.12
A_16_P35372301	175165194	175165248	RASAL2	1.00	0.55	-0.13
A_14_P128242	175174711	175174770	RASAL2	1.18	0.77	-0.48

A_16_P15356515	175179448	175179507	chr1:175179448-175179507	0.82	0.48	-0.29
A_16_P00200561	175183958	175184017	chr1:175183958-175184017	0.90	0.83	-0.22
A_16_P15356544	175188599	175188658	chr1:175188599-175188658	0.84	0.69	-0.30
A_16_P00200581	175194706	175194765	FLJ44005	1.27	1.24	-0.41
A_16_P00200591	175202389	175202448	chr1:175202389-175202448	1.20	1.36	-0.24
A_16_P35372463	175210761	175210820	chr1:175210761-175210820	1.14	0.76	-0.26
A_14_P131393	175220001	175220060	C1orf49	1.14	0.98	-0.15
A_14_P122374	175222626	175222685	C1orf49	1.31	1.28	0.27
A_16_P00200637	175227043	175227102	AK097518	0.51	1.54	0.53
A_16_P00200648	175233625	175233681	AK097518	1.06	1.19	-0.11
A_16_P15356694	175236913	175236972	AK097518	0.50	0.31	-0.50
A_16_P00200666	175244063	175244115	FLJ35530	0.76	0.52	-0.54
A_14_P200048	175249343	175249402	FLJ35530	0.80	1.65	-0.55
A_14_P127089	175252409	175252468	chr1:175252409-175252468	0.86	1.09	-0.30
A_16_P35372619	175265482	175265541	chr1:175265482-175265541	1.07	0.96	0.08
A_16_P00200702	175280275	175280334	chr1:175280275-175280334	1.39	1.24	-0.33
A_16_P15356826	175297055	175297114	chr1:175297055-175297114	0.93	0.97	-0.40
A_16_P00200774	175325078	175325137	chr1:175325078-175325137	1.48	0.74	-0.23
A_16_P35372803	175341216	175341275	chr1:175341216-175341275	1.15	1.15	-0.19
A_16_P15356975	175356774	175356833	chr1:175356774-175356833	1.20	1.29	-0.18
A_14_P129595	175403354	175403413	chr1:175403354-175403413	1.01	0.66	-0.15
A_16_P15357077	175414547	175414606	chr1:175414547-175414606	0.87	1.40	-0.43
A_16_P15357102	175424639	175424698	chr1:175424639-175424698	1.08	1.72	-0.32
A_14_P104217	175440261	175440320	RALGPS2	1.12	0.35	-0.30
A_16_P15357161	175454405	175454464	RALGPS2	0.46	3.01	-0.36
A_16_P35373045	175473177	175473236	RALGPS2	0.73	0.75	-0.65
A_16_P35373066	175481382	175481441	RALGPS2	0.78	0.22	-0.28
A_16_P15357202	175487800	175487859	RALGPS2	1.11	1.30	-0.39
A_16_P35373097	175494530	175494589	RALGPS2	1.05	0.44	-0.49
A_14_P129938	175508870	175508929	RALGPS2	0.79	1.35	-0.44
A_16_P15357258	175516262	175516321	RALGPS2	0.55	0.30	-0.77
A_16_P00200996	175522466	175522525	RALGPS2	1.11	1.34	-0.04
A_14_P136537	175534311	175534370	RALGPS2	0.63	1.65	-0.34
A_16_P15357302	175545085	175545144	RALGPS2	1.14	0.99	-0.37
A_14_P132749	175551630	175551689	RALGPS2	0.13	1.03	-0.51
A_14_P138710	175558793	175558852	RALGPS2	0.34	0.77	-0.14
A_16_P00201059	175566133	175566192	RALGPS2	0.76	0.35	-0.47
A_16_P15357384	175570566	175570625	RALGPS2	0.91	0.90	-0.25
A_16_P35373315	175579749	175579808	RALGPS2	1.24	1.44	-0.09
A_14_P131017	175586877	175586936	RALGPS2	0.52	1.54	-0.13
A_16_P15357486	175602821	175602880	RALGPS2	1.32	0.67	-0.55
A_14_P101158	175617403	175617462	RALGPS2	1.18	0.74	-0.33
A_16_P00201164	175628103	175628162	chr1:175628103-175628162	1.21	0.89	-0.33
A_16_P35373490	175640903	175640962	chr1:175640903-175640962	0.83	0.23	-0.31
A_16_P00201196	175648727	175648786	chr1:175648727-175648786	1.29	1.25	0.50
A_16_P15357613	175656254	175656313	chr1:175656254-175656313	1.19	0.45	0.88
A_16_P15357628	175662135	175662194	chr1:175662135-175662194	0.73	1.46	0.24
A_16_P15357639	175668133	175668192	chr1:175668133-175668192	1.16	0.75	0.42

A_16_P15357655	175675129	175675188	chr1:175675129-175675188	1.20	0.09	0.32
A_16_P35373573	175695071	175695130	chr1:175695071-175695130	1.07	0.94	0.43
A_14_P139155	175696530	175696589	chr1:175696530-175696589	1.45	1.04	0.39
A_16_P00201241	175704309	175704368	chr1:175704309-175704368	1.10	0.23	0.41
A_16_P00201251	175712089	175712148	chr1:175712089-175712148	1.23	0.93	0.33
A_16_P15357705	175718991	175719050	chr1:175718991-175719050	1.01	0.28	0.34
A_16_P00201269	175724663	175724722	chr1:175724663-175724722	0.90	0.98	0.48
A_16_P15357764	175737199	175737258	FAM20B	0.64	0.88	0.25
A_16_P35373702	175746014	175746073	FAM20B	0.97	0.70	0.69
A_16_P15357813	175755411	175755470	FAM20B	0.75	1.20	0.53
A_14_P139267	175767022	175767081	FAM20B	1.09	1.40	0.47
A_16_P35373779	175773677	175773736	FAM20B	1.10	1.81	0.43
A_16_P00201361	175782169	175782228	chr1:175782169-175782228	0.24	0.88	0.02
A_14_P102230	175789376	175789434	TOR3A	0.96	-0.22	0.35
A_14_P116182	175794871	175794926	TOR3A	1.38	1.20	0.66
A_16_P15357945	175801328	175801387	chr1:175801328-175801387	0.99	1.37	0.43
A_14_P123471	175807155	175807214	BX538317	0.69	0.47	0.39
A_16_P15357996	175816750	175816809	ABL2	0.92	0.92	0.43
A_16_P35373941	175822323	175822382	ABL2	1.72	0.46	0.55
A_16_P15358044	175834873	175834932	ABL2	0.52	0.45	0.02
A_14_P106955	175843655	175843714	ABL2	1.22	0.85	0.33
A_16_P35374021	175857110	175857169	ABL2	0.67	1.53	0.48
A_14_P115783	175865256	175865315	ABL2	0.96	0.71	0.23
A_16_P15358111	175872678	175872737	ABL2	0.87	0.24	0.30
A_16_P15358131	175879755	175879814	ABL2	0.91	1.36	0.26
A_16_P15358158	175890300	175890359	ABL2	0.99	0.59	0.48
A_14_P127543	175899513	175899572	ABL2	0.79	0.44	0.46
A_16_P00201543	175908101	175908160	ABL2	1.27	1.17	0.48
A_14_P125430	175916784	175916843	ABL2	0.61	1.72	0.21
A_14_P125430	175916784	175916843	ABL2	0.56	1.33	0.10
A_14_P125430	175916784	175916843	ABL2	0.54	-0.46	0.32
A_16_P00201564	175927859	175927918	ABL2	1.48	0.89	0.50
A_16_P15358265	175933831	175933890	chr1:175933831-175933890	1.04	0.87	-0.16
A_14_P134224	175947269	175947328	chr1:175947269-175947328	1.44	1.17	0.28
A_16_P15358352	175973820	175973878	chr1:175973820-175973878	1.14	0.83	0.55
A_16_P35374299	175988417	175988476	chr1:175988417-175988476	1.32	1.17	0.58
A_16_P15358393	176002673	176002732	SOAT1	1.27	1.59	0.30
A_16_P15358416	176011385	176011444	SOAT1	0.94	0.51	0.23
A_16_P15358441	176019147	176019206	SOAT1	0.86	0.80	0.23
A_16_P15358457	176027097	176027156	SOAT1	1.31	1.22	0.38
A_14_P132241	176036300	176036359	SOAT1	1.14	1.12	0.31
A_16_P15358502	176044405	176044464	SOAT1	1.03	0.74	0.26
A_16_P35374484	176055029	176055088	SOAT1	0.49	1.45	0.21
A_14_P127568	176069386	176069445	C1orf125	0.73	1.01	0.33
A_16_P00201764	176080170	176080229	C1orf125	0.79	1.16	0.01
A_16_P15358603	176086056	176086115	C1orf125	1.15	0.29	0.22
A_14_P120915	176091526	176091585	C1orf125	0.87	0.61	0.59
A_16_P15358629	176102188	176102247	C1orf125	1.00	0.45	0.18

A_16_P15358632	176110908	176110967	C1orf125	0.70	0.68	0.20
A_14_P108466	176128016	176128075	C1orf125	0.51	0.40	0.07
A_14_P109770	176145728	176145787	C1orf125	-0.27	1.90	0.38
A_16_P35374657	176158529	176158588	C1orf125	1.24	1.50	0.10
A_14_P107451	176167712	176167771	C1orf125	1.15	0.92	0.43
A_16_P00201823	176183951	176184010	C1orf125	0.16	1.39	0.34
A_16_P00201823	176183951	176184010	C1orf125	0.13	0.65	0.26
A_16_P00201823	176183951	176184010	C1orf125	0.24	0.75	0.30
A_16_P15358707	176192347	176192406	C1orf125	1.24	1.03	0.35
A_16_P15358713	176201051	176201110	C1orf125	-0.33	3.04	-0.73
A_14_P122722	176207659	176207718	C1orf125	1.17	0.85	0.52
A_14_P122635	176226195	176226254	C1orf125	1.13	0.76	0.29
A_16_P15358769	176235732	176235791	C1orf125	1.10	1.61	0.34
A_16_P00201866	176243774	176243833	C1orf125	1.64	1.29	0.57
A_14_P111645	176255407	176255466	C1orf125	0.68	1.62	0.21
A_16_P15358829	176261982	176262041	NPHS2	1.09	0.90	0.32
A_14_P108691	176275366	176275425	NPHS2	1.39	1.39	0.43
A_16_P15358872	176284347	176284406	chr1:176284347-176284406	0.87	0.97	0.61
A_14_P102727	176294477	176294536	TDRD5	1.12	1.15	0.54
A_16_P15358924	176312604	176312663	TDRD5	0.97	1.86	0.68
A_16_P35374949	176321772	176321831	TDRD5	1.01	1.78	0.33
A_14_P109102	176336575	176336632	TDRD5	1.39	1.68	0.46
A_16_P15359006	176345325	176345384	TDRD5	0.19	0.28	-0.18
A_16_P15359015	176352922	176352981	TDRD5	1.55	1.40	0.38
A_16_P15359028	176363036	176363095	TDRD5	1.66	1.15	0.45
A_16_P35375053	176369924	176369983	TDRD5	0.74	0.96	0.34
A_16_P00202008	176384326	176384385	TDRD5	1.05	1.55	0.34
A_14_P126409	176391797	176391856	TDRD5	0.18	-0.80	0.45
A_16_P15359098	176413006	176413065	chr1:176413006-176413065	1.02	1.03	0.24
A_14_P117504	176429493	176429552	chr1:176429493-176429552	0.96	1.24	0.40
A_16_P00202079	176446701	176446760	C1orf76	0.91	1.14	0.55
A_16_P00202090	176455950	176456009	C1orf76	1.05	2.37	0.56
A_16_P35375225	176462398	176462457	C1orf76	1.15	1.32	0.51
A_16_P15359232	176473402	176473461	C1orf76	0.77	0.48	0.41
A_16_P15359252	176480426	176480485	C1orf76	0.87	0.67	0.51
A_16_P15359273	176486353	176486412	C1orf76	1.24	0.90	0.54
A_14_P119082	176495872	176495931	C1orf76	1.68	0.60	0.63
A_16_P15359331	176505769	176505828	C1orf76	1.12	1.40	0.45
A_14_P130487	176516945	176517004	C1orf76	1.23	1.37	0.33
A_16_P15359390	176524885	176524944	chr1:176524885-176524944	0.79	0.95	0.32
A_14_P139312	176534338	176534397	chr1:176534338-176534397	1.26	0.45	0.53
A_16_P15359445	176546452	176546511	TOR1AIP2	0.96	1.23	0.44
A_14_P118015	176553563	176553622	TOR1AIP2	1.08	1.71	0.25
A_16_P35375529	176561901	176561960	TOR1AIP2	0.81	0.60	0.28
A_14_P120230	176565927	176565982	TOR1AIP2	1.13	1.66	0.43
A_16_P15359521	176573604	176573663	TOR1AIP2	0.94	1.03	0.51
A_16_P00202303	176577965	176578024	TOR1AIP2	1.04	0.47	0.36
A_14_P112346	176587612	176587671	TOR1AIP1	1.24	1.14	0.31

A_14_P122023	176595795	176595854	TOR1AIP1	1.06	0.75	0.57
A_16_P35375683	176609451	176609510	TOR1AIP1	0.97	0.99	0.32
A_16_P15359653	176619044	176619103	TOR1AIP1	1.16	1.63	0.62
A_16_P15359685	176630867	176630926	chr1:176630867-176630926	0.76	1.35	0.65
A_16_P35375758	176640780	176640839	chr1:176640780-176640839	1.23	1.28	0.35
A_16_P00202416	176647834	176647893	chr1:176647834-176647893	1.44	1.72	0.49
A_14_P138343	176656603	176656662	CEP350	1.13	0.72	0.38
A_16_P00202437	176662262	176662321	CEP350	1.01	0.15	0.46
A_16_P35375814	176669302	176669361	CEP350	1.34	0.98	0.48
A_16_P00202447	176675231	176675288	CEP350	0.72	1.45	0.33
A_16_P15359780	176681617	176681676	CEP350	0.83	0.33	0.31
A_16_P00202469	176691347	176691406	CEP350	1.04	0.36	0.39
A_16_P00202484	176697498	176697557	CEP350	0.20	2.23	0.32
A_16_P00202499	176707234	176707293	CEP350	0.44	0.50	0.02
A_16_P00202515	176715204	176715261	CEP350	1.25	0.92	0.39
A_16_P15359894	176720983	176721042	CEP350	0.60	0.96	0.48
A_16_P00202538	176732207	176732266	CEP350	1.18	1.27	0.47
A_14_P115508	176742429	176742488	CEP350	1.02	0.98	0.47
A_16_P00202561	176753788	176753847	CEP350	0.91	0.97	0.33
A_16_P00202573	176762985	176763044	CEP350	0.85	1.03	0.30
A_16_P00202604	176781404	176781463	CEP350	1.36	1.42	0.60
A_14_P110201	176793384	176793443	CEP350	0.55	1.44	0.40
A_16_P15360106	176805091	176805150	CEP350	1.38	1.53	0.44
A_16_P00202652	176812354	176812413	CEP350	0.87	1.33	0.55
A_14_P111896	176822919	176822978	chr1:176822919-176822978	0.97	0.82	0.58
A_16_P00202681	176834537	176834596	chr1:176834537-176834596	1.75	1.05	0.71
A_16_P15360199	176846281	176846340	chr1:176846281-176846340	1.07	0.83	0.48
A_16_P00202704	176857786	176857845	QSCN6	1.62	1.94	0.75
A_16_P00202715	176867023	176867082	QSCN6	1.47	0.80	0.69
A_14_P119855	176873805	176873864	QSCN6	1.46	1.96	0.61
A_16_P35376390	176884208	176884267	QSCN6	1.00	0.92	0.60
A_14_P138336	176899251	176899297	CR597598	1.01	0.91	0.27
A_16_P15360377	176911098	176911157	chr1:176911098-176911157	1.24	1.15	0.66
A_16_P15360416	176925931	176925990	chr1:176925931-176925990	1.37	0.35	0.58
A_16_P00202835	176932722	176932781	LHX4	1.30	2.15	0.70
A_16_P35376558	176938712	176938771	LHX4	1.24	2.02	1.27
A_14_P116231	176949224	176949269	LHX4	1.34	0.60	0.58
A_16_P15360524	176956299	176956358	LHX4	1.43	1.11	0.59
A_16_P15360548	176964358	176964417	LHX4	1.06	1.03	0.52
A_14_P116523	176972225	176972269	LHX4	0.88	0.85	0.35
A_16_P15360612	176982380	176982439	chr1:176982380-176982439	1.03	0.79	0.26
A_16_P15360636	176989104	176989163	ACBD6	1.37	0.81	0.35
A_16_P00202973	177000667	177000726	ACBD6	0.77	0.81	0.29
A_16_P15360661	177010142	177010201	ACBD6	0.92	1.40	0.33
A_16_P35376783	177018511	177018570	ACBD6	1.48	1.18	0.52
A_14_P118707	177030603	177030662	ACBD6	1.13	1.07	0.35
A_16_P15360701	177042927	177042986	ACBD6	1.14	1.14	0.38
A_16_P00203014	177053486	177053545	ACBD6	1.04	0.80	0.59

A_16_P35376867	177062505	177062564	ACBD6	1.05	0.22	0.43
A_14_P113554	177071709	177071768	ACBD6	1.05	1.27	0.46
A_16_P15360809	177089199	177089258	ACBD6	0.78	0.85	0.32
A_16_P35376925	177099221	177099280	ACBD6	0.44	0.45	0.35
A_16_P15360838	177106541	177106600	ACBD6	0.63	0.48	0.26
A_16_P00203093	177116993	177117052	ACBD6	0.24	1.18	0.04
A_14_P136182	177130990	177131049	ACBD6	0.90	1.19	0.31
A_16_P15360909	177139157	177139216	ACBD6	0.79	1.01	0.35
A_16_P15360913	177145609	177145668	ACBD6	1.25	1.32	0.16
A_16_P35377040	177156478	177156537	ACBD6	0.72	0.95	0.32
A_16_P15360949	177168481	177168540	ACBD6	0.97	0.53	0.43
A_16_P15360974	177176930	177176989	ACBD6	1.56	1.25	0.57
A_16_P15360993	177184964	177185023	ACBD6	1.34	1.11	0.46
A_14_P120887	177196283	177196342	ACBD6	0.53	1.18	0.45
A_16_P35377152	177204047	177204106	chr1:177204047-177204106	0.82	0.98	0.37
A_16_P15361046	177214750	177214809	chr1:177214750-177214809	1.02	0.52	0.52
A_16_P15361117	177238815	177238874	chr1:177238815-177238874	0.99	0.91	0.47
A_16_P00203268	177253230	177253289	chr1:177253230-177253289	1.20	1.61	0.80
A_16_P35377328	177275561	177275620	chr1:177275561-177275620	1.23	1.20	0.43
A_16_P00203328	177299080	177299139	chr1:177299080-177299139	1.63	0.98	0.18
A_16_P15361304	177312587	177312646	chr1:177312587-177312646	0.67	1.23	0.47
A_14_P135537	177330369	177330428	chr1:177330369-177330428	1.16	1.29	0.52
A_16_P15361378	177342796	177342855	XPR1	0.90	0.71	0.17
A_16_P15361389	177348302	177348361	XPR1	0.81	0.61	0.14
A_16_P15361399	177354030	177354089	XPR1	0.73	1.17	0.51
A_14_P139777	177371715	177371774	XPR1	1.11	1.55	0.47
A_16_P35377539	177376701	177376760	XPR1	1.09	1.82	0.40
A_16_P35377574	177393411	177393470	XPR1	0.39	0.51	0.36
A_16_P35377587	177406796	177406855	XPR1	1.16	0.85	0.57
A_16_P15361480	177417448	177417507	XPR1	1.02	0.71	0.78
A_16_P35377628	177426247	177426306	XPR1	0.76	0.40	0.47
A_14_P134666	177434157	177434216	XPR1	0.22	0.18	0.38
A_16_P15361534	177450564	177450623	XPR1	1.12	1.39	0.77
A_16_P15361553	177456515	177456574	XPR1	1.03	1.31	0.88
A_16_P35377684	177467718	177467777	XPR1	0.46	1.08	0.65
A_16_P15361586	177477705	177477764	XPR1	1.43	0.89	0.90
A_14_P127338	177488522	177488581	XPR1	1.02	1.24	0.84
A_16_P15361637	177498460	177498519	XPR1	0.81	1.27	0.79
A_16_P00203547	177504331	177504390	XPR1	0.80	0.84	0.85
A_16_P15361666	177512800	177512859	XPR1	0.94	1.09	0.76
A_16_P35377807	177519621	177519680	XPR1	0.93	1.03	0.96
A_14_P125725	177525682	177525741	XPR1	0.35	0.39	0.58
A_16_P00203594	177537293	177537352	XPR1	1.09	0.94	0.80
A_16_P15361770	177552377	177552436	XPR1	0.88	1.26	0.69
A_16_P00203632	177564518	177564577	XPR1	0.93	1.17	0.74
A_16_P35377952	177575582	177575641	XPR1	0.97	0.94	0.87
A_14_P132482	177586263	177586322	XPR1	1.12	0.66	0.73
A_16_P15361867	177594274	177594333	chr1:177594274-177594333	1.27	0.62	0.92

A_16_P35378029	177606768	177606827	chr1:177606768-177606827	0.84	0.21	0.82
A_16_P15361915	177614691	177614750	AB046834	0.96	1.03	1.01
A_16_P00203709	177621345	177621404	AB046834	1.08	0.63	0.97
A_16_P00203728	177632401	177632460	AB046834	1.60	0.75	0.97
A_16_P00203739	177637859	177637915	AB046834	1.15	1.00	0.80
A_14_P131176	177645629	177645676	AB046834	1.06	1.27	0.34
A_16_P15362058	177662019	177662078	AK056657	1.46	1.51	0.90
A_16_P35378261	177675345	177675404	STX6	0.59	1.30	0.99
A_16_P00203814	177682402	177682461	STX6	1.30	1.47	0.94
A_14_P123433	177694176	177694235	STX6	1.06	1.87	0.95
A_16_P35378344	177703478	177703537	STX6	1.44	1.29	0.89
A_16_P35378363	177712227	177712286	STX6	0.86	0.86	1.10
A_14_P103599	177722499	177722558	STX6	1.07	0.92	0.91
A_16_P35378410	177729017	177729076	chr1:177729017-177729076	0.50	1.14	0.57
A_16_P35378422	177734484	177734543	chr1:177734484-177734543	0.80	1.64	0.65
A_16_P00203905	177739194	177739253	MR1	1.60	1.07	0.98
A_14_P131517	177745086	177745145	MR1	1.34	1.41	0.79
A_14_P120754	177751975	177752026	MR1	0.40	0.97	-0.16
A_14_P109497	177756772	177756831	CR602405	1.00	0.95	1.05
A_16_P15362340	177764098	177764157	chr1:177764098-177764157	1.04	0.76	0.87
A_16_P15362346	177770063	177770122	chr1:177770063-177770122	0.82	-0.78	0.81
A_16_P00203960	177777834	177777893	chr1:177777834-177777893	1.22	0.65	0.85
A_16_P00203975	177785385	177785444	chr1:177785385-177785444	1.46	1.88	0.81
A_14_P113088	177791410	177791468	IER5	0.92	1.32	1.01
A_16_P35378602	177796855	177796913	chr1:177796855-177796913	0.88	1.01	0.82
A_14_P201253	177801508	177801567	chr1:177801508-177801567	0.83	0.60	0.72
A_16_P00204018	177808232	177808291	chr1:177808232-177808291	1.39	0.72	0.90
A_16_P35378665	177816510	177816569	chr1:177816510-177816569	0.99	0.00	0.80
A_16_P15362524	177830924	177830983	chr1:177830924-177830983	1.47	0.74	1.20
A_16_P15362613	177867618	177867677	chr1:177867618-177867677	1.50	1.01	1.07
A_16_P00204130	177888353	177888412	chr1:177888353-177888412	1.13	0.97	0.71
A_16_P15362690	177906433	177906492	chr1:177906433-177906492	1.07	1.21	0.98
A_16_P00204166	177918356	177918414	chr1:177918356-177918414	2.65	1.17	3.46
A_16_P15362750	177926052	177926111	chr1:177926052-177926111	1.09	1.04	0.61
A_16_P35378937	177937349	177937408	AF387616	1.36	0.80	1.39
A_16_P00204197	177944625	177944684	chr1:177944625-177944684	1.40	1.44	0.88
A_16_P00204216	177966505	177966564	chr1:177966505-177966564	0.47	1.49	1.00
A_14_P130489	178001310	178001369	chr1:178001310-178001369	1.04	1.10	0.95
A_16_P00204312	178047308	178047367	chr1:178047308-178047367	1.58	1.20	0.94
A_16_P15363071	178086134	178086193	chr1:178086134-178086193	0.78	0.01	0.57
A_16_P15363192	178132569	178132628	chr1:178132569-178132628	0.82	0.61	0.78
A_16_P00204483	178153518	178153577	chr1:178153518-178153577	0.87	0.46	0.89
A_14_P116167	178167694	178167753	chr1:178167694-178167753	1.20	1.00	0.88
A_16_P00204526	178181770	178181829	chr1:178181770-178181829	0.88	0.57	0.74
A_16_P15363350	178187872	178187931	CACNA1E	0.85	0.85	0.81
A_16_P35379559	178198152	178198211	CACNA1E	0.74	0.36	0.83
A_14_P128630	178205629	178205688	CACNA1E	1.13	0.70	0.80
A_16_P35379614	178213429	178213488	CACNA1E	0.96	0.94	0.70

A_16_P15363466	178224502	178224561	CACNA1E	1.16	1.03	0.84
A_16_P00204627	178233851	178233910	CACNA1E	0.69	0.70	0.89
A_16_P15363502	178239566	178239625	CACNA1E	1.34	1.22	1.00
A_16_P15363518	178246138	178246197	CACNA1E	0.91	0.55	0.30
A_16_P35379724	178255912	178255971	CACNA1E	0.65	0.74	0.71
A_16_P00204670	178266391	178266450	CACNA1E	0.88	1.11	1.06
A_14_P113074	178279969	178280021	CACNA1E	1.75	1.73	0.90
A_16_P15363623	178285886	178285945	CACNA1E	0.96	0.68	0.58
A_16_P15363649	178295786	178295845	CACNA1E	1.05	1.72	0.30
A_16_P35379878	178311321	178311380	CACNA1E	0.72	1.04	0.16
A_16_P00204770	178321454	178321513	CACNA1E	1.32	0.93	0.30
A_16_P00204777	178327650	178327709	CACNA1E	1.04	1.15	0.55
A_16_P15363762	178339051	178339110	CACNA1E	1.31	1.65	0.54
A_16_P00204813	178344981	178345040	CACNA1E	1.40	1.14	0.65
A_14_P137706	178352110	178352161	CACNA1E	0.65	0.89	0.21
A_16_P15363833	178364525	178364584	CACNA1E	0.66	1.04	0.21
A_16_P15363883	178379899	178379958	CACNA1E	0.51	1.55	0.34
A_16_P35380096	178388730	178388789	CACNA1E	0.57	0.33	0.35
A_16_P15363911	178397273	178397332	CACNA1E	0.87	1.15	0.20
A_16_P00204910	178406819	178406878	CACNA1E	1.59	0.15	-0.31
A_14_P138422	178416107	178416166	CACNA1E	0.70	1.17	-0.37
A_16_P00204943	178423773	178423832	CACNA1E	0.83	1.56	-0.19
A_16_P15364013	178431752	178431811	CACNA1E	1.14	1.46	-0.19
A_14_P102064	178440582	178440641	CACNA1E	0.56	0.06	-0.54
A_16_P00204998	178449372	178449431	CACNA1E	0.82	0.56	-0.03
A_14_P101261	178461310	178461369	CACNA1E	0.75	1.33	-0.36
A_16_P00205040	178468926	178468985	CACNA1E	0.60	0.34	-0.70
A_14_P109807	178478566	178478625	CACNA1E	1.22	1.24	-0.41
A_16_P15364203	178484662	178484721	CACNA1E	0.86	0.73	-0.47
A_16_P15364224	178490850	178490909	CACNA1E	1.20	0.92	-0.55
A_16_P00205110	178498121	178498180	CACNA1E	0.52	-0.20	-0.38
A_14_P135753	178505991	178506050	chr1:178505991-178506050	0.87	1.36	0.40
A_16_P15364322	178522405	178522464	chr1:178522405-178522464	1.07	1.11	-0.39
A_16_P15364332	178546094	178546153	chr1:178546094-178546153	1.44	1.26	-0.39
A_16_P00205178	178595087	178595146	chr1:178595087-178595146	0.82	0.30	-0.39
A_16_P15364453	178636666	178636725	chr1:178636666-178636725	1.22	0.57	-0.24
A_16_P15364555	178701913	178701972	chr1:178701913-178701972	1.02	0.70	-0.49
A_16_P35380810	178724653	178724712	chr1:178724653-178724712	0.66	1.05	-0.34
A_16_P15364608	178735449	178735508	chr1:178735449-178735508	-0.32	0.39	-0.47
A_16_P35380842	178743245	178743304	chr1:178743245-178743304	0.82	0.68	-0.45
A_16_P00205313	178749078	178749137	chr1:178749078-178749137	1.06	1.17	-0.48
A_16_P00205325	178755989	178756048	ZNF648	1.28	0.68	-0.42
A_16_P15364669	178761588	178761647	ZNF648	1.73	0.87	-0.21
A_16_P00205343	178766849	178766908	AK055155	0.98	1.49	-0.27
A_16_P00205353	178776644	178776703	chr1:178776644-178776703	1.41	0.79	-0.25
A_16_P15364699	178782869	178782928	chr1:178782869-178782928	1.02	0.93	-0.18
A_16_P00205386	178799116	178799175	chr1:178799116-178799175	1.47	1.59	-0.33
A_16_P00205446	178838518	178838577	AK128015	1.55	0.48	-0.28

A_16_P15364887	178848650	178848709	AK128015	0.57	0.46	-0.41
A_14_P139794	178866095	178866154	AK128015	0.89	1.51	-0.19
A_16_P35381192	178883154	178883213	AK128015	0.81	0.92	-0.54
A_16_P15365000	178894458	178894517	AK128015	1.20	1.28	-0.29
A_16_P15365028	178904952	178905011	AK128015	1.36	0.28	-0.34
A_16_P00205571	178914363	178914422	AK128015	0.83	0.77	-0.51
A_16_P35381315	178934461	178934520	AK128015	1.71	1.68	-0.22
A_16_P15365126	178945542	178945600	chr1:178945542-178945600	1.36	1.09	-0.50
A_16_P15365167	178963849	178963908	chr1:178963849-178963908	1.09	1.03	-0.25
A_16_P00205697	179004945	179005004	chr1:179004945-179005004	1.42	0.79	-0.17
A_16_P35381526	179016396	179016455	chr1:179016396-179016455	0.84	1.01	-0.44
A_16_P00205724	179024667	179024726	chr1:179024667-179024726	1.41	0.88	-0.25
A_16_P00205749	179040395	179040454	chr1:179040395-179040454	1.01	0.95	-0.22
A_16_P00205770	179056159	179056218	chr1:179056159-179056218	0.94	0.37	-0.65
A_16_P15365421	179065996	179066055	chr1:179065996-179066055	0.97	0.97	-0.49
A_16_P15365433	179074367	179074426	chr1:179074367-179074426	1.01	0.26	-0.41
A_16_P15365445	179078869	179078928	chr1:179078869-179078928	1.14	1.28	-0.42
A_14_P201170	179087571	179087630	GLUL	1.36	1.46	0.04
A_14_P137662	179089661	179089720	GLUL	1.53	1.40	-0.09
A_16_P15365495	179094406	179094465	chr1:179094406-179094465	1.15	1.60	-0.38
A_14_P124300	179099122	179099181	TEDDM1	0.12	1.25	-0.32
A_16_P15365523	179102304	179102363	chr1:179102304-179102363	0.35	0.07	-0.81
A_16_P15365526	179105828	179105887	chr1:179105828-179105887	0.86	1.20	-0.13
A_16_P15365543	179112799	179112858	C1orf120	0.98	1.29	0.25
A_16_P00205868	179116999	179117058	chr1:179116999-179117058	1.54	1.35	0.69
A_16_P15365564	179120515	179120574	chr1:179120515-179120574	0.37	0.99	0.10
A_14_P122161	179125540	179125599	chr1:179125540-179125599	1.34	1.39	0.41
A_16_P00205892	179135979	179136038	chr1:179135979-179136038	1.47	2.18	0.53
A_16_P15365635	179148341	179148400	chr1:179148341-179148400	1.21	0.60	0.49
A_16_P15365650	179154770	179154829	RGSL2	1.13	1.03	0.34
A_14_P118636	179160774	179160833	RGSL2	1.32	1.79	0.39
A_16_P00205934	179174803	179174862	RGSL2	1.23	1.32	0.45
A_14_P116531	179189153	179189212	RGSL2	1.19	1.39	0.43
A_16_P15365716	179198166	179198225	RGSL1	0.44	1.08	-0.33
A_14_P119818	179207077	179207136	RGSL1	0.52	0.98	0.20
A_16_P15365726	179222620	179222677	RGSL1	1.23	0.96	0.40
A_16_P35381997	179228297	179228356	RGSL1	1.36	1.04	0.38
A_16_P35382024	179241137	179241196	RGSL1	1.74	1.54	0.56
A_16_P35382031	179247787	179247846	RGSL1	0.52	0.23	0.38
A_16_P00205996	179254643	179254702	RGSL1	1.36	1.04	0.45
A_14_P123634	179261044	179261103	RGSL1	1.16	0.66	0.28
A_16_P15365818	179265492	179265551	chr1:179265492-179265551	1.05	1.24	0.34
A_16_P15365827	179271777	179271836	chr1:179271777-179271836	1.18	0.95	0.42
A_16_P35382110	179276346	179276405	RNASEL	1.06	0.89	0.46
A_14_P103014	179282068	179282124	RNASEL	1.48	1.17	0.61
A_14_P118074	179284850	179284907	RNASEL	0.88	0.55	0.23
A_14_P123752	179287118	179287177	RNASEL	1.00	0.97	0.29
A_16_P15365881	179293293	179293352	chr1:179293293-179293352	1.71	1.23	0.76

A_14_P100951	179299508	179299567	RGS16	0.79	0.92	0.26
A_16_P15365913	179304112	179304171	RGS16	0.88	1.10	0.27
A_16_P00206084	179308485	179308544	chr1:179308485-179308544	0.61	1.17	0.50
A_16_P15365942	179313351	179313407	AK023174	0.55	0.99	0.38
A_16_P35382235	179318312	179318371	chr1:179318312-179318371	1.40	1.53	0.53
A_16_P35382252	179325186	179325245	chr1:179325186-179325245	1.45	1.32	0.57
A_16_P35382279	179336644	179336703	chr1:179336644-179336703	0.95	0.43	0.35
A_16_P15366018	179342655	179342714	AF297014	0.59	-0.12	0.15
A_14_P129392	179350555	179350614	RGS8	0.83	1.05	0.03
A_16_P15366057	179355508	179355567	RGS8	1.36	0.84	0.20
A_16_P15366090	179365086	179365145	RGS8	1.23	1.36	0.50
A_16_P15366122	179373551	179373610	RGS8	1.06	1.34	0.23
A_16_P35382428	179382667	179382726	chr1:179382667-179382726	1.38	1.67	0.28
A_14_P136125	179396111	179396170	chr1:179396111-179396170	1.24	0.97	0.44
A_16_P15366247	179420685	179420744	chr1:179420685-179420744	0.94	1.26	0.94
A_16_P15366302	179443743	179443802	chr1:179443743-179443802	1.30	1.73	0.50
A_16_P15366381	179473217	179473276	chr1:179473217-179473276	1.07	0.53	0.25
A_14_P112408	179495071	179495130	NPL	0.39	1.01	0.25
A_16_P00206418	179504465	179504524	NPL	1.04	1.65	0.30
A_14_P116122	179514383	179514442	NPL	1.00	1.32	0.18
A_16_P00206456	179526566	179526625	NPL	0.92	0.80	0.17
A_16_P15366558	179532895	179532953	chr1:179532895-179532953	1.42	1.75	0.57
A_14_P121408	179543307	179543366	DHX9	0.94	0.88	0.26
A_16_P15366595	179549889	179549948	DHX9	0.74	1.49	0.25
A_16_P15366634	179560729	179560788	DHX9	1.12	0.59	0.70
A_14_P138409	179568716	179568775	DHX9	1.24	0.71	0.32
A_16_P35382983	179575821	179575880	DHX9	0.72	1.09	0.26
A_16_P15366693	179581225	179581284	DHX9	0.42	-0.57	0.26
A_16_P00206554	179590270	179590329	chr1:179590270-179590329	1.42	0.53	0.53
A_16_P15366738	179600924	179600983	C1orf14	0.79	0.95	0.47
A_16_P00206569	179605179	179605238	C1orf14	1.08	1.58	0.45
A_14_P121553	179620513	179620572	C1orf14	1.05	0.92	0.60
A_16_P15366773	179631967	179632026	C1orf14	0.76	1.44	0.18
A_16_P15366780	179641211	179641270	C1orf14	1.02	1.51	0.43
A_14_P139468	179652142	179652201	C1orf14	0.64	1.96	0.71
A_16_P00206605	179658849	179658908	chr1:179658849-179658908	1.15	1.29	0.38
A_14_P117373	179672654	179672713	chr1:179672654-179672713	0.88	0.36	0.36
A_16_P35383202	179684073	179684132	chr1:179684073-179684132	0.97	0.75	0.58
A_16_P15366900	179703319	179703378	chr1:179703319-179703378	0.94	1.26	0.44
A_16_P35383254	179713037	179713096	chr1:179713037-179713096	1.14	1.91	0.37
A_16_P15366937	179720111	179720170	chr1:179720111-179720170	1.12	0.17	0.22
A_16_P15366961	179727384	179727443	LAMC1	0.82	1.06	0.36
A_16_P35383328	179738239	179738298	LAMC1	0.79	1.14	0.35
A_14_P102452	179753901	179753960	LAMC1	0.81	1.12	0.60
A_16_P15367080	179763993	179764052	LAMC1	0.71	1.73	0.33
A_16_P00206752	179770037	179770096	LAMC1	0.99	0.75	0.29
A_14_P135169	179775739	179775798	LAMC1	1.05	0.72	0.32
A_16_P15367125	179780523	179780582	LAMC1	0.80	1.10	0.28

A_14_P123053	179796025	179796084	LAMC1	0.66	1.12	0.47
A_16_P00206819	179809134	179809193	LAMC1	1.09	0.75	0.58
A_16_P15367231	179817322	179817381	LAMC1	1.36	0.69	0.71
A_16_P35383598	179825356	179825415	LAMC1	1.03	-0.31	0.51
A_16_P35383620	179831088	179831147	LAMC1	1.10	0.00	0.56
A_14_P118994	179845814	179845873	LAMC1	1.56	0.49	0.68
A_16_P00206915	179860416	179860475	chr1:179860416-179860475	1.50	0.27	0.55
A_16_P00206931	179870684	179870743	chr1:179870684-179870743	0.89	0.97	0.54
A_14_P135136	179879654	179879713	chr1:179879654-179879713	1.17	1.11	0.50
A_16_P00206963	179887626	179887685	LAMC2	1.38	0.87	0.35
A_16_P35383813	179894570	179894628	LAMC2	1.70	0.19	0.62
A_16_P00206986	179900378	179900437	LAMC2	0.73	1.41	0.38
A_16_P15367507	179913127	179913186	LAMC2	0.95	0.67	0.42
A_14_P123336	179921710	179921766	LAMC2	0.60	0.71	0.52
A_16_P35383933	179931685	179931744	LAMC2	0.84	1.30	0.28
A_16_P15367586	179937524	179937583	LAMC2	1.07	0.75	0.58
A_14_P121573	179944930	179944989	LAMC2	1.16	1.17	0.32
A_16_P00207099	179952549	179952608	NMNAT2	1.07	1.16	0.58
A_16_P35384037	179961718	179961777	NMNAT2	0.73	1.09	0.28
A_16_P15367682	179972027	179972086	NMNAT2	0.81	1.49	0.42
A_16_P00207134	179978972	179979031	NMNAT2	1.04	0.72	0.17
A_16_P15367720	179986803	179986862	NMNAT2	1.33	0.88	0.45
A_14_P102303	179994538	179994597	NMNAT2	1.59	1.47	0.43
A_16_P15367776	180010457	180010516	NMNAT2	0.92	1.11	0.38
A_16_P00207201	180017791	180017850	NMNAT2	1.16	1.24	0.27
A_14_P136131	180031528	180031587	NMNAT2	0.82	1.28	0.20
A_16_P00207240	180038963	180039022	NMNAT2	1.26	1.14	1.44
A_16_P15367880	180047671	180047730	NMNAT2	1.27	0.53	0.49
A_16_P15367906	180055093	180055152	NMNAT2	1.46	0.97	0.46
A_14_P122561	180065149	180065208	NMNAT2	1.19	1.33	0.37
A_16_P35384371	180080550	180080609	NMNAT2	1.28	0.70	0.38
A_16_P00207322	180090011	180090070	NMNAT2	0.79	1.32	0.45
A_14_P139813	180108185	180108244	NMNAT2	1.04	1.33	0.42
A_16_P15368055	180116181	180116240	NMNAT2	1.15	1.10	0.51
A_16_P00207363	180123773	180123832	chr1:180123773-180123832	0.85	1.29	0.50
A_16_P15368108	180136059	180136118	chr1:180136059-180136118	1.04	1.46	0.56
A_16_P15368119	180144476	180144535	chr1:180144476-180144535	0.94	0.68	0.26
A_14_P110947	180163365	180163424	BC032873	0.53	1.28	0.51
A_16_P15368197	180174972	180175031	SMG7	0.66	1.16	0.53
A_16_P15368217	180181132	180181191	SMG7	1.02	0.73	0.38
A_14_P125606	180186890	180186949	SMG7	0.93	0.72	0.22
A_16_P15368250	180197926	180197985	SMG7	0.88	1.45	0.38
A_16_P00207475	180205637	180205696	SMG7	1.22	1.09	0.34
A_14_P119364	180216723	180216782	SMG7	0.82	1.71	0.20
A_16_P35384727	180222232	180222291	SMG7	1.07	1.55	0.74
A_16_P15368351	180230121	180230180	SMG7	0.86	0.16	0.59
A_14_P137949	180237464	180237523	SMG7	0.42	1.87	-0.03
A_16_P35384831	180250517	180250576	SMG7	0.66	0.82	0.04

A_14_P102200	180256827	180256886	NCF2	0.36	1.28	0.15
A_16_P00207607	180269887	180269946	NCF2	0.84	1.00	0.22
A_16_P00207617	180276335	180276394	NCF2	1.13	1.00	0.58
A_14_P102742	180285845	180285904	NCF2	0.90	0.31	0.37
A_16_P00207659	180298120	180298179	chr1:180298120-180298179	1.28	0.92	0.36
A_16_P15368583	180304116	180304174	chr1:180304116-180304174	0.71	-1.13	0.40
A_16_P00207679	180309688	180309747	chr1:180309688-180309747	1.52	1.32	0.43
A_16_P15368622	180323137	180323196	chr1:180323137-180323196	0.66	0.93	0.27
A_14_P127894	180327378	180327437	ARPC5	0.98	0.75	0.05
A_16_P15368648	180331428	180331487	ARPC5	1.09	1.64	0.40
A_16_P15368659	180335230	180335289	ARPC5	0.96	0.57	0.23
A_16_P15368681	180342815	180342874	RGL1	0.61	0.79	0.19
A_16_P15368688	180345836	180345895	RGL1	1.14	1.06	0.42
A_16_P35385139	180349225	180349284	RGL1	1.08	1.71	0.27
A_14_P122344	180355538	180355597	RGL1	1.33	0.96	0.30
A_16_P15368725	180365380	180365439	RGL1	0.57	1.14	0.22
A_16_P15368734	180375737	180375795	RGL1	0.80	1.02	0.37
A_16_P15368750	180386491	180386550	RGL1	0.95	1.15	0.28
A_16_P15368771	180393173	180393232	RGL1	1.12	0.41	0.40
A_16_P35385240	180402924	180402983	RGL1	1.01	1.69	0.48
A_16_P15368824	180417000	180417059	RGL1	0.97	1.00	0.33
A_16_P15368842	180431766	180431825	RGL1	0.61	0.60	0.46
A_14_P122634	180443048	180443107	RGL1	1.44	1.24	0.41
A_16_P15368913	180460515	180460574	RGL1	0.80	1.03	0.31
A_16_P00207869	180471876	180471935	RGL1	1.27	0.85	0.32
A_16_P35385393	180481532	180481591	RGL1	1.31	0.93	0.37
A_16_P15368977	180493040	180493099	RGL1	0.71	0.78	0.11
A_16_P35385439	180500874	180500933	RGL1	0.12	1.06	0.02
A_14_P112725	180507209	180507259	RGL1	0.70	0.97	0.47
A_16_P15369035	180515497	180515556	RGL1	1.11	0.87	0.36
A_16_P35385527	180529595	180529654	RGL1	1.23	3.43	0.23
A_16_P00207982	180539409	180539468	RGL1	1.12	0.25	0.33
A_16_P15369136	180552388	180552447	RGL1	0.60	1.07	0.27
A_16_P15369160	180558931	180558990	RGL1	1.15	0.85	0.34
A_16_P00208025	180564742	180564801	RGL1	1.23	0.48	0.43
A_16_P15369217	180578211	180578270	RGL1	0.85	1.02	0.15
A_16_P35385697	180585722	180585781	RGL1	1.17	1.57	0.62
A_14_P102108	180592861	180592920	RGL1	0.89	1.00	0.37
A_16_P35385757	180602627	180602686	RGL1	0.82	1.05	0.29
A_16_P35385783	180612794	180612853	RGL1	1.49	1.20	0.43
A_16_P00208135	180619235	180619294	RGL1	1.25	1.20	0.19
A_14_P129733	180627867	180627926	RGL1	1.48	1.20	0.37
A_16_P35385881	180639091	180639150	GLT25D2	1.04	-0.39	0.02
A_14_P124293	180655544	180655597	GLT25D2	1.22	2.06	0.25
A_16_P15369478	180666055	180666114	GLT25D2	1.14	1.32	0.44
A_16_P00208232	180671895	180671954	GLT25D2	1.49	1.07	0.39
A_14_P118021	180679249	180679308	GLT25D2	0.88	1.18	0.33
A_16_P15369565	180693152	180693211	GLT25D2	0.92	0.54	0.40

A_16_P15369583	180699193	180699252	GLT25D2	0.96	1.91	0.14
A_16_P00208290	180710729	180710788	GLT25D2	0.46	1.32	0.23
A_14_P134373	180723924	180723983	GLT25D2	0.91	0.76	0.19
A_16_P35386151	180731239	180731298	GLT25D2	1.31	0.17	0.15
A_16_P35386179	180738643	180738702	chr1:180738643-180738702	1.33	1.66	0.55
A_16_P15369712	180746456	180746515	chr1:180746456-180746515	0.94	1.24	0.16
A_14_P120406	180753109	180753168	C1orf19	0.58	0.42	0.28
A_16_P15369743	180758689	180758748	C1orf19	0.63	-0.56	0.20
A_16_P15369757	180763219	180763278	C1orf19	1.12	0.59	0.28
A_14_P107275	180769734	180769793	C1orf19	0.55	1.19	0.19
A_16_P00208391	180774200	180774259	C1orf19	0.27	0.10	0.20
A_16_P15369803	180781569	180781628	chr1:180781569-180781628	0.94	0.81	0.10
A_16_P15369835	180793359	180793418	chr1:180793359-180793418	1.07	0.19	0.61
A_16_P35386357	180803380	180803439	chr1:180803380-180803439	1.07	0.61	0.29
A_14_P108929	180822870	180822929	chr1:180822870-180822929	1.40	1.33	0.53
A_16_P15369956	180849843	180849902	chr1:180849843-180849902	0.85	1.50	0.25
A_16_P00208558	180902925	180902984	chr1:180902925-180902984	0.35	1.63	0.54
A_16_P15370156	180940607	180940666	chr1:180940607-180940666	0.80	0.82	0.23
A_16_P35386777	180998800	180998859	chr1:180998800-180998859	1.00	0.35	0.51
A_14_P102637	181050668	181050727	chr1:181050668-181050727	0.94	1.10	0.27
A_16_P15370471	181081550	181081609	chr1:181081550-181081609	1.01	0.85	0.13
A_16_P15370517	181094245	181094304	C1orf21	0.78	0.26	0.08
A_16_P15370542	181104636	181104695	C1orf21	1.03	0.67	0.27
A_14_P106649	181114712	181114771	C1orf21	1.17	1.52	0.39
A_16_P15370598	181121490	181121549	C1orf21	0.60	0.70	0.33
A_16_P15370617	181131058	181131117	C1orf21	1.25	1.02	0.39
A_16_P15370663	181148267	181148326	C1orf21	0.78	0.82	0.53
A_16_P35387176	181157591	181157650	C1orf21	1.25	1.32	0.23
A_14_P114265	181163419	181163478	C1orf21	0.72	0.86	0.22
A_16_P35387220	181170231	181170290	C1orf21	0.84	1.08	0.48
A_16_P15370755	181175946	181176005	C1orf21	0.81	0.56	0.29
A_16_P15370783	181184592	181184651	C1orf21	1.33	0.09	0.36
A_16_P35387313	181198479	181198538	C1orf21	0.71	0.92	0.34
A_14_P133475	181208353	181208409	C1orf21	1.14	1.88	0.27
A_16_P00209042	181214663	181214722	C1orf21	1.01	1.35	0.37
A_16_P15370899	181220607	181220666	C1orf21	0.71	1.37	0.33
A_16_P35387400	181227474	181227533	C1orf21	0.82	1.47	0.31
A_16_P15370946	181237704	181237763	C1orf21	0.36	0.66	0.35
A_14_P115660	181244508	181244567	C1orf21	0.86	1.49	0.35
A_16_P00209114	181257077	181257136	C1orf21	1.33	0.61	0.43
A_16_P15371027	181266311	181266370	C1orf21	1.08	1.11	0.39
A_16_P15371058	181276057	181276116	C1orf21	0.85	0.71	0.36
A_16_P15371075	181284046	181284105	C1orf21	1.14	1.19	0.49
A_14_P127917	181291544	181291603	C1orf21	0.90	1.47	0.63
A_16_P35387595	181299149	181299208	C1orf21	1.12	0.98	0.34
A_16_P15371130	181305266	181305325	C1orf21	1.25	1.12	0.44
A_16_P00209213	181315906	181315965	C1orf21	1.04	1.84	0.11
A_14_P139662	181324853	181324912	C1orf21	1.18	0.61	0.15

A_16_P00209257	181336872	181336931	chr1:181336872-181336931	1.30	0.64	0.45
A_16_P15371253	181344738	181344797	chr1:181344738-181344797	1.06	0.93	0.58
A_16_P15371292	181359492	181359551	chr1:181359492-181359551	0.80	1.08	0.21
A_14_P109512	181371569	181371628	chr1:181371569-181371628	1.76	1.24	0.44
A_16_P00209326	181387023	181387082	chr1:181387023-181387082	0.85	1.02	0.27
A_14_P136298	181393732	181393791	EDEM3	0.75	0.96	0.51
A_16_P35387896	181403550	181403609	EDEM3	0.98	1.61	0.29
A_14_P101136	181417759	181417818	EDEM3	0.29	0.36	0.42
A_16_P00209400	181424570	181424629	EDEM3	0.30	1.93	0.40
A_16_P15371489	181432075	181432134	EDEM3	0.70	1.16	0.35
A_16_P00209425	181437816	181437875	EDEM3	1.21	0.93	0.49
A_16_P00209438	181448821	181448880	EDEM3	1.42	0.87	0.33
A_14_P101734	181454768	181454827	EDEM3	1.01	1.03	0.32
A_16_P35388115	181469735	181469794	chr1:181469735-181469794	1.24	1.79	0.46
A_16_P15371611	181477453	181477512	chr1:181477453-181477512	1.23	1.01	0.63
A_14_P133074	181492030	181492089	C1orf24	1.26	1.62	0.45
A_16_P35388202	181503290	181503349	C1orf24	1.10	0.62	0.39
A_16_P15371706	181520223	181520282	C1orf24	0.97	0.15	0.39
A_14_P115527	181532709	181532768	C1orf24	0.54	2.45	0.44
A_16_P00209581	181543699	181543758	C1orf24	1.69	0.76	0.33
A_16_P15371794	181557218	181557277	C1orf24	1.12	1.42	0.18
A_14_P136518	181564183	181564242	C1orf24	1.21	1.77	0.18
A_16_P35388387	181580252	181580311	C1orf24	0.60	-0.03	0.19
A_16_P00209645	181589219	181589278	C1orf24	0.95	1.55	0.39
A_14_P112372	181599943	181600002	C1orf24	1.10	0.95	0.33
A_16_P15371943	181610967	181611026	C1orf24	1.33	1.20	0.34
A_16_P15371957	181616440	181616499	C1orf24	0.93	0.69	0.43
A_16_P35388511	181622530	181622589	C1orf24	1.14	0.19	0.49
A_16_P15371989	181633871	181633930	C1orf24	1.05	1.01	0.30
A_16_P15372009	181641085	181641144	C1orf24	1.17	0.04	0.37
A_14_P134020	181651527	181651586	C1orf24	0.85	0.86	0.12
A_16_P15372057	181659349	181659408	C1orf24	0.67	0.39	0.09
A_16_P00209756	181673420	181673479	C1orf24	0.13	-0.45	0.03
A_14_P125556	181681985	181682044	chr1:181681985-181682044	0.87	1.22	0.26
A_16_P15372129	181690863	181690922	chr1:181690863-181690922	0.90	1.07	0.37
A_16_P15372154	181701959	181702018	chr1:181701959-181702018	1.24	1.10	0.40
A_16_P15372174	181715248	181715307	chr1:181715248-181715307	1.18	1.32	0.38
A_16_P15372203	181739427	181739486	chr1:181739427-181739486	0.83	0.36	0.19
A_16_P00209854	181755853	181755912	RNF2	0.95	1.05	0.18
A_14_P113596	181760667	181760726	RNF2	0.84	0.41	0.32
A_16_P15372303	181771356	181771415	RNF2	1.04	1.18	0.49
A_16_P15372330	181781509	181781568	RNF2	0.67	1.45	0.28
A_14_P133195	181792489	181792541	RNF2	1.32	0.46	0.20
A_16_P35388912	181798793	181798852	RNF2	1.10	1.07	0.39
A_16_P35388932	181814744	181814803	chr1:181814744-181814803	1.14	1.17	0.31
A_16_P00209945	181825839	181825898	C1orf25	0.58	1.16	0.44
A_14_P127856	181838229	181838288	C1orf25	1.09	1.34	0.44
A_16_P15372454	181844706	181844765	C1orf25	0.19	0.67	0.19

A_16_P00209986	181851147	181851206	C1orf25	0.42	0.51	-0.02
A_14_P139076	181857631	181857687	C1orf25	1.84	1.07	0.50
A_16_P35389088	181867380	181867439	C1orf26	0.59	0.15	0.19
A_16_P00210023	181875555	181875614	C1orf26	1.04	0.94	0.37
A_14_P106091	181881156	181881215	C1orf26	0.56	1.26	0.31
A_16_P15372587	181890445	181890504	C1orf26	1.21	0.78	0.41
A_14_P108809	181905541	181905600	C1orf26	0.62	0.96	0.23
A_16_P15372620	181913083	181913142	C1orf26	1.14	1.27	0.56
A_16_P15372644	181921250	181921309	C1orf26	0.87	1.46	0.23
A_16_P35389241	181929329	181929388	C1orf26	1.01	1.37	0.42
A_16_P15372672	181938680	181938739	C1orf26	0.81	0.62	0.29
A_14_P125333	181944391	181944450	C1orf26	0.81	1.03	0.29
A_16_P35389292	181960296	181960355	C1orf26	0.70	0.53	0.27
A_14_P101338	181972116	181972175	C1orf26	0.82	0.97	0.14
A_16_P15372764	181977406	181977465	C1orf26	0.46	1.77	0.37
A_14_P129398	181992515	181992574	AK000128	0.27	1.93	0.39
A_14_P138240	181998825	181998884	IVNS1ABP	1.62	1.38	0.53
A_14_P131093	182007875	182007934	IVNS1ABP	0.77	0.97	0.03
A_14_P133068	182009923	182009981	IVNS1ABP	-0.19	1.26	-0.20
A_14_P201575	182014657	182014716	IVNS1ABP	0.84	0.83	0.26
A_16_P15372889	182019437	182019496	chr1:182019437-182019496	1.52	0.82	0.46
A_16_P15372897	182024454	182024513	AF508904	1.07	0.68	0.30
A_16_P35389532	182035656	182035715	AF508905	1.15	0.75	0.26
A_16_P35389589	182057044	182057103	chr1:182057044-182057103	0.78	1.53	0.46
A_16_P35389693	182109897	182109956	chr1:182109897-182109956	0.97	1.07	0.47
A_14_P119610	182185208	182185267	chr1:182185208-182185267	1.19	1.12	0.35
A_16_P15373391	182233661	182233720	chr1:182233661-182233720	0.00	1.37	0.34
A_16_P15373449	182256203	182256262	chr1:182256203-182256262	0.54	1.64	0.14
A_16_P15373517	182276713	182276772	AF508906	1.43	0.79	0.44
A_16_P35390108	182294766	182294825	AF508906	1.23	0.54	0.33
A_14_P136218	182308072	182308131	AF508906	0.87	0.45	0.44
A_16_P00210655	182323563	182323622	AF508906	0.95	0.68	0.38
A_16_P00210664	182334676	182334735	chr1:182334676-182334735	1.28	1.02	0.50
A_16_P15373617	182352605	182352664	chr1:182352605-182352664	0.90	0.96	0.37
A_16_P35390291	182379947	182380006	chr1:182379947-182380006	1.08	1.10	0.38
A_16_P15373768	182404118	182404177	chr1:182404118-182404177	0.53	1.37	0.07
A_14_P202455	182425317	182425376	chr1:182425317-182425376	1.31	1.20	0.35
A_14_P103255	182435693	182435752	HMCN1	1.09	1.30	0.41
A_16_P35390447	182446367	182446426	HMCN1	1.34	1.08	0.45
A_16_P15373888	182456869	182456928	HMCN1	0.67	1.47	0.30
A_16_P00210856	182463440	182463499	HMCN1	1.23	0.43	0.63
A_16_P15373923	182469382	182469441	HMCN1	1.38	1.47	0.52
A_16_P35390528	182477788	182477847	HMCN1	0.87	1.36	0.31
A_16_P15373951	182485308	182485367	HMCN1	0.36	1.72	0.43
A_14_P136509	182491162	182491221	HMCN1	0.97	1.56	0.20
A_16_P15373998	182504691	182504750	HMCN1	0.60	0.84	0.34
A_16_P15374020	182510894	182510953	HMCN1	0.94	1.54	0.42
A_16_P15374039	182516447	182516506	HMCN1	0.85	0.32	0.53

A_16_P15374063	182524448	182524507	HMCN1	0.91	0.93	0.22
A_16_P35390668	182536791	182536850	HMCN1	0.71	1.65	0.76
A_16_P00210982	182546830	182546889	HMCN1	1.05	0.41	0.41
A_16_P15374153	182557393	182557452	HMCN1	0.65	1.34	0.31
A_14_P125851	182566511	182566570	HMCN1	0.63	1.38	0.19
A_16_P15374209	182576064	182576123	HMCN1	0.89	1.47	0.25
A_16_P35390805	182586584	182586643	HMCN1	0.84	1.17	0.33
A_16_P35390814	182596372	182596431	HMCN1	0.61	0.62	0.16
A_16_P15374259	182602559	182602618	HMCN1	0.70	1.35	0.34
A_16_P15374288	182610169	182610228	HMCN1	1.01	0.67	0.44
A_16_P15374313	182616542	182616601	HMCN1	1.24	0.72	0.53
A_16_P15374339	182624384	182624443	HMCN1	1.20	1.03	0.81
A_16_P35390955	182634281	182634340	HMCN1	0.90	1.20	0.23
A_16_P15374395	182641159	182641218	HMCN1	0.74	0.05	0.38
A_14_P113852	182657845	182657904	HMCN1	0.54	0.71	0.19
A_16_P35391034	182664685	182664744	HMCN1	0.77	1.20	0.12
A_16_P00211197	182671146	182671205	HMCN1	0.93	0.75	0.25
A_16_P15374495	182678688	182678747	HMCN1	0.61	0.68	0.45
A_14_P105247	182688319	182688378	HMCN1	1.18	0.70	0.50
A_14_P201826	182694119	182694170	HMCN1	1.22	1.13	0.33
A_16_P00211255	182702175	182702234	HMCN1	0.65	1.06	0.36
A_16_P00211265	182707934	182707993	HMCN1	0.77	0.44	0.43
A_16_P15374600	182715895	182715954	HMCN1	0.55	0.29	0.36
A_16_P15374621	182722345	182722404	HMCN1	0.63	0.70	0.47
A_16_P35391253	182728087	182728146	HMCN1	1.30	-0.02	0.62
A_16_P35391291	182743325	182743384	HMCN1	0.72	0.87	0.30
A_16_P15374710	182754547	182754606	HMCN1	1.06	1.70	0.40
A_16_P15374750	182768771	182768830	HMCN1	0.66	-0.02	0.24
A_16_P00211383	182777380	182777439	HMCN1	0.37	1.48	0.48
A_16_P35391443	182788802	182788861	HMCN1	1.00	-0.43	0.17
A_16_P35391475	182795981	182796040	HMCN1	0.57	1.21	0.37
A_16_P35391509	182807738	182807797	HMCN1	1.05	0.89	0.51
A_16_P15374889	182814728	182814787	HMCN1	0.75	0.47	0.44
A_16_P00211487	182830758	182830817	HMCN1	1.40	0.24	0.38
A_16_P00211499	182837630	182837689	HMCN1	0.96	1.31	0.57
A_14_P112274	182845174	182845233	HMCN1	1.00	0.39	0.23
A_14_P202083	182854702	182854761	HMCN1	1.06	1.44	0.26
A_16_P00211546	182872777	182872836	HMCN1	0.76	1.18	0.29
A_16_P15375091	182888158	182888217	HMCN1	0.87	1.49	0.24
A_16_P15375135	182904607	182904666	chr1:182904607-182904666	0.44	0.24	0.25
A_16_P00211618	182913341	182913400	chr1:182913341-182913400	0.93	0.49	0.47
A_16_P00211667	182963555	182963614	chr1:182963555-182963614	1.40	1.32	0.50
A_16_P35391943	182981265	182981324	chr1:182981265-182981324	0.83	1.37	0.32
A_14_P106242	182988885	182988944	chr1:182988885-182988944	0.84	0.34	0.32
A_16_P15375309	182993731	182993790	chr1:182993731-182993790	0.98	0.40	0.53
A_16_P15375324	182998573	182998632	PRG4	0.81	1.02	0.48
A_14_P132240	183004965	183005024	PRG4	1.42	0.48	0.47
A_14_P124075	183007383	183007442	PRG4	0.79	1.27	0.30

A_14_P137921	183009892	183009951	PRG4	0.05	1.08	0.20
A_16_P15375384	183015383	183015442	TPR	0.86	0.98	0.36
A_16_P15375408	183023359	183023418	TPR	0.90	1.66	0.30
A_14_P108950	183028175	183028234	TPR	1.04	0.96	0.37
A_16_P35392163	183037783	183037842	TPR	0.82	0.53	0.55
A_16_P00211815	183044782	183044841	TPR	0.86	0.99	0.42
A_14_P111281	183054522	183054581	TPR	1.07	1.73	0.53
A_14_P124077	183054582	183054641	TPR	0.74	0.56	0.50
A_14_P109857	183061572	183061631	TPR	1.08	0.92	0.41
A_14_P120768	183068655	183068714	TPR	0.27	2.23	0.80
A_16_P00211870	183074215	183074274	TPR	0.46	1.07	0.18
A_16_P15375588	183080448	183080507	C1orf27	1.00	1.64	0.50
A_16_P00211897	183086809	183086868	C1orf27	0.49	0.68	0.15
A_16_P15375629	183092439	183092498	C1orf27	0.94	1.04	0.74
A_14_P112995	183101730	183101784	C1orf27	0.56	0.88	0.38
A_16_P00211936	183106975	183107034	C1orf27	0.25	-0.38	0.22
A_16_P15375701	183116291	183116350	C1orf27	0.78	1.09	0.46
A_16_P15375719	183123680	183123739	chr1:183123680-183123739	0.84	0.98	0.37
A_16_P00211975	183132719	183132778	chr1:183132719-183132778	1.07	1.03	0.59
A_16_P15375741	183136915	183136974	chr1:183136915-183136974	0.52	0.69	0.31
A_14_P126927	183144874	183144933	PDC	0.97	1.34	0.51
A_14_P201150	183153541	183153600	PDC	0.75	1.01	0.33
A_16_P15375808	183158605	183158664	PDC	0.39	1.54	0.28
A_16_P15375838	183167716	183167775	chr1:183167716-183167775	0.90	0.93	0.33
A_16_P15375846	183174066	183174125	chr1:183174066-183174125	0.90	0.63	0.48
A_16_P15375873	183182470	183182529	chr1:183182470-183182529	1.23	0.86	0.47
A_16_P15375956	183220924	183220983	chr1:183220924-183220983	0.80	1.17	0.36
A_16_P00212172	183281421	183281480	chr1:183281421-183281480	0.47	0.73	0.49
A_16_P15376120	183323060	183323119	chr1:183323060-183323119	0.81	0.73	0.15
A_16_P00212235	183352447	183352506	chr1:183352447-183352506	1.47	1.18	0.53
A_16_P00212249	183366714	183366773	chr1:183366714-183366773	0.15	1.43	0.46
A_14_P100478	183373041	183373100	PTGS2	1.10	1.01	0.40
A_14_P109141	183374785	183374844	PTGS2	0.70	1.27	0.24
A_14_P119570	183376869	183376928	PTGS2	0.93	0.69	0.48
A_14_P125781	183377195	183377254	PTGS2	0.98	1.05	0.35
A_14_P125781	183377195	183377254	PTGS2	1.07	1.04	0.41
A_14_P125781	183377195	183377254	PTGS2	1.04	0.93	0.43
A_14_P202164	183379931	183379990	PTGS2	0.82	1.37	0.45
A_16_P15376274	183384502	183384561	chr1:183384502-183384561	1.18	0.69	0.24
A_16_P15376276	183392520	183392579	chr1:183392520-183392579	1.01	1.27	1.00
A_16_P00212290	183405846	183405905	chr1:183405846-183405905	0.85	1.13	0.33
A_16_P15376324	183420953	183421012	chr1:183420953-183421012	1.15	0.89	0.33
A_16_P00212382	183472133	183472192	chr1:183472133-183472192	0.66	0.86	-0.17
A_16_P15376485	183493723	183493782	chr1:183493723-183493782	1.36	1.53	0.41
A_16_P15376534	183508517	183508576	chr1:183508517-183508576	0.44	0.14	0.35
A_14_P126406	183516735	183516794	chr1:183516735-183516794	0.21	-0.13	0.15
A_16_P15376592	183529089	183529148	chr1:183529089-183529148	0.38	1.31	0.15
A_16_P15376621	183537393	183537452	PLA2G4A	0.74	1.67	0.19

A_16_P15376644	183545013	183545072	PLA2G4A	2.48	-0.02	0.24
A_14_P134547	183555076	183555135	PLA2G4A	0.68	0.52	0.34
A_16_P15376706	183567019	183567078	PLA2G4A	0.83	1.04	0.32
A_16_P00212551	183576687	183576746	PLA2G4A	1.07	1.75	0.41
A_14_P112455	183594885	183594944	PLA2G4A	0.84	1.27	0.25
A_16_P15376813	183601782	183601841	PLA2G4A	0.89	1.00	0.37
A_16_P00212608	183607741	183607800	PLA2G4A	0.49	0.89	0.10
A_16_P15376845	183613656	183613715	PLA2G4A	0.07	1.29	-0.01
A_16_P15376851	183623971	183624030	PLA2G4A	1.00	0.95	0.34
A_16_P15376872	183631855	183631914	PLA2G4A	-0.34	-0.98	0.01
A_14_P107053	183640756	183640815	PLA2G4A	0.74	0.72	0.24
A_16_P00212665	183647492	183647551	PLA2G4A	0.75	1.46	0.38
A_16_P00212683	183656901	183656960	PLA2G4A	0.49	0.91	0.23
A_14_P121124	183666209	183666268	PLA2G4A	1.41	0.41	0.26
A_16_P15376989	183672158	183672217	PLA2G4A	0.48	1.00	0.03
A_16_P35393804	183680215	183680274	PLA2G4A	0.96	1.31	0.30
A_14_P118647	183689150	183689209	PLA2G4A	1.19	1.60	0.23
A_16_P15377084	183701431	183701490	chr1:183701431-183701490	0.77	0.75	0.30
A_16_P00212770	183711465	183711524	chr1:183711465-183711524	0.63	1.08	0.27
A_16_P15377171	183743278	183743337	chr1:183743278-183743337	0.63	1.06	0.24
A_16_P35394015	183764010	183764069	chr1:183764010-183764069	0.89	0.78	0.35
A_16_P35394069	183781508	183781567	chr1:183781508-183781567	0.90	0.90	0.38
A_14_P124332	183806342	183806401	chr1:183806342-183806401	0.85	0.65	0.34
A_16_P15377426	183844770	183844829	chr1:183844770-183844829	0.64	0.79	0.22
A_16_P35394232	183864192	183864251	chr1:183864192-183864251	1.01	1.06	0.38
A_16_P15377461	183888875	183888934	chr1:183888875-183888934	0.38	0.63	0.13
A_16_P15377518	183909580	183909639	chr1:183909580-183909639	0.95	0.80	0.12
A_16_P35394330	183926154	183926213	chr1:183926154-183926213	0.70	0.97	0.22
A_14_P127626	183950075	183950134	chr1:183950075-183950134	0.78	0.82	0.07
A_16_P15377723	183985366	183985425	chr1:183985366-183985425	0.67	1.69	0.32
A_16_P15377771	184009739	184009798	chr1:184009739-184009798	0.45	0.72	0.47
A_16_P00213179	184026940	184026999	chr1:184026940-184026999	0.47	1.05	0.29
A_16_P35394650	184052579	184052638	chr1:184052579-184052638	0.55	1.22	0.22
A_16_P15377897	184068309	184068368	chr1:184068309-184068368	0.21	0.74	0.20
A_16_P00213299	184114435	184114494	chr1:184114435-184114494	0.51	0.11	0.24
A_16_P35394917	184158977	184159036	chr1:184158977-184159036	0.40	-0.04	0.11
A_14_P103970	184182830	184182889	chr1:184182830-184182889	-0.14	1.19	0.21
A_16_P15378274	184204300	184204359	chr1:184204300-184204359	0.64	0.63	0.30
A_16_P15378362	184237559	184237618	chr1:184237559-184237618	0.21	0.36	0.10
A_16_P15378399	184255038	184255097	chr1:184255038-184255097	-0.12	0.05	0.01
A_16_P15378435	184271649	184271708	chr1:184271649-184271708	-0.18	0.46	0.04
A_16_P15378487	184290905	184290964	chr1:184290905-184290964	0.03	1.24	0.02
A_16_P15378497	184305851	184305910	chr1:184305851-184305910	-0.11	0.48	0.04
A_16_P15378528	184321886	184321945	chr1:184321886-184321945	0.35	0.19	0.23
A_16_P15378553	184330442	184330501	chr1:184330442-184330501	0.24	0.33	0.22
A_16_P00213622	184336245	184336289	chr1:184336245-184336289	0.46	0.77	0.01
A_16_P15378569	184340679	184340738	chr1:184340679-184340738	0.77	0.50	0.03
A_16_P35395346	184344524	184344583	C1orf99	0.37	0.94	0.21

A_16_P00213643	184353399	184353458	chr1:184353399-184353458	0.67	1.31	0.06
A_16_P15378621	184359471	184359530	chr1:184359471-184359530	0.51	0.75	0.31
A_16_P15378672	184375684	184375743	chr1:184375684-184375743	0.45	0.22	0.09
A_16_P15378721	184396107	184396166	chr1:184396107-184396166	0.02	0.82	0.11
A_16_P15378776	184413463	184413522	chr1:184413463-184413522	0.27	0.76	0.15
A_16_P15378837	184444620	184444679	chr1:184444620-184444679	-0.11	0.27	0.35
A_16_P00213817	184477355	184477414	chr1:184477355-184477414	0.28	1.04	0.17
A_16_P15378988	184510591	184510650	chr1:184510591-184510650	0.42	0.46	0.31
A_14_P118086	184545320	184545379	chr1:184545320-184545379	0.36	1.57	0.29
A_16_P15379129	184581665	184581724	chr1:184581665-184581724	0.23	0.37	0.19
A_16_P15379202	184604844	184604903	chr1:184604844-184604903	0.54	1.72	0.23
A_16_P15379302	184635834	184635893	chr1:184635834-184635893	0.41	0.89	0.24
A_16_P00214134	184688297	184688356	chr1:184688297-184688356	0.51	0.30	0.41
A_16_P15379483	184709653	184709712	chr1:184709653-184709712	-0.03	0.71	0.02
A_16_P15379535	184728954	184729013	chr1:184728954-184729013	0.37	1.21	0.23
A_16_P15379593	184755672	184755731	chr1:184755672-184755731	0.40	0.75	0.16
A_14_P104768	184780831	184780890	chr1:184780831-184780890	0.29	1.02	0.27
A_16_P35396433	184795591	184795650	chr1:184795591-184795650	-0.16	0.19	0.04
A_16_P15379683	184811249	184811308	chr1:184811249-184811308	0.61	0.97	0.32
A_16_P00214317	184837072	184837131	chr1:184837072-184837131	0.17	1.22	0.25
A_16_P35396597	184864241	184864300	chr1:184864241-184864300	0.81	1.30	0.36
A_16_P15379929	184898083	184898142	chr1:184898083-184898142	0.41	0.40	0.33
A_14_P102748	184937606	184937665	chr1:184937606-184937665	-0.08	0.45	0.19
A_16_P15380069	184981041	184981100	chr1:184981041-184981100	0.46	0.68	0.48
A_16_P15380167	185021731	185021790	chr1:185021731-185021790	0.54	1.16	0.36
A_16_P15380239	185067485	185067544	chr1:185067485-185067544	-0.71	1.17	0.32
A_14_P118719	185123050	185123109	chr1:185123050-185123109	-0.40	1.56	0.24
A_16_P15380568	185195479	185195538	chr1:185195479-185195538	0.07	1.21	0.33
A_16_P00214843	185216722	185216781	chr1:185216722-185216781	0.54	0.77	0.51
A_16_P35397442	185250551	185250610	chr1:185250551-185250610	-0.11	0.50	0.16
A_16_P15380825	185306100	185306159	chr1:185306100-185306159	0.27	1.23	0.27
A_16_P35397681	185351791	185351850	chr1:185351791-185351850	0.44	1.44	0.26
A_14_P110417	185390738	185390797	chr1:185390738-185390797	0.58	0.85	0.53
A_16_P15381063	185417276	185417335	chr1:185417276-185417335	-0.31	1.37	0.28
A_16_P35397869	185439640	185439699	chr1:185439640-185439699	0.62	0.47	0.43
A_16_P15381204	185494641	185494700	chr1:185494641-185494700	0.37	1.04	0.54
A_14_P133560	185518310	185518369	chr1:185518310-185518369	0.19	1.05	0.21
A_16_P15381285	185540351	185540410	chr1:185540351-185540410	0.09	0.78	0.20
A_16_P00215295	185580661	185580720	chr1:185580661-185580720	0.23	0.99	0.30
A_16_P15381402	185603368	185603427	chr1:185603368-185603427	0.75	0.53	0.42
A_16_P15381450	185621030	185621089	chr1:185621030-185621089	0.29	0.49	0.35
A_16_P35398266	185642042	185642101	chr1:185642042-185642101	0.53	0.57	0.16
A_16_P00215405	185673755	185673814	chr1:185673755-185673814	0.59	1.27	0.33
A_14_P134533	185702847	185702906	chr1:185702847-185702906	0.17	0.93	0.40
A_16_P15381710	185735271	185735330	chr1:185735271-185735330	0.19	1.28	0.20
A_16_P15381771	185763769	185763828	chr1:185763769-185763828	0.16	0.89	0.27
A_16_P15381842	185787967	185788026	chr1:185787967-185788026	0.19	-0.19	0.06
A_16_P15381885	185812249	185812308	chr1:185812249-185812308	0.63	0.69	0.39

A_14_P112540	185832842	185832901	chr1:185832842-185832901	-0.03	0.70	0.14
A_16_P35398746	185860989	185861048	chr1:185860989-185861048	0.48	1.08	0.18
A_16_P35398850	185906490	185906549	chr1:185906490-185906549	0.48	1.33	0.26
A_16_P35398883	185922040	185922099	chr1:185922040-185922099	0.17	0.89	0.20
A_16_P15382147	185947809	185947868	chr1:185947809-185947868	-0.05	0.16	0.25
A_16_P15382262	185996662	185996721	chr1:185996662-185996721	0.15	0.81	0.26
A_16_P35399063	186011436	186011495	chr1:186011436-186011495	0.30	0.77	0.22
A_14_P126992	186034550	186034609	chr1:186034550-186034609	-0.33	1.03	0.13
A_16_P15382386	186062909	186062968	chr1:186062909-186062968	0.66	0.39	0.43
A_16_P15382426	186079062	186079121	chr1:186079062-186079121	0.31	0.76	0.24
A_16_P15382474	186098372	186098431	chr1:186098372-186098431	-0.04	0.95	0.09
A_16_P15382531	186127742	186127801	chr1:186127742-186127801	-0.14	0.22	0.14
A_16_P15382584	186145776	186145835	chr1:186145776-186145835	0.79	1.33	0.28
A_16_P35399396	186161759	186161818	chr1:186161759-186161818	-0.05	0.29	0.19
A_16_P35399434	186180177	186180236	chr1:186180177-186180236	0.65	0.60	0.60
A_16_P00216092	186202054	186202113	chr1:186202054-186202113	0.29	0.62	0.10
A_16_P00216146	186244486	186244545	chr1:186244486-186244545	-0.29	0.74	0.09
A_16_P15382857	186274119	186274178	chr1:186274119-186274178	0.46	0.67	0.40
A_16_P15382915	186293872	186293931	chr1:186293872-186293931	0.48	0.15	0.08
A_14_P116320	186306358	186306417	chr1:186306358-186306417	-0.46	0.42	0.16
A_16_P15383032	186337104	186337163	chr1:186337104-186337163	0.21	1.17	0.31
A_16_P35399871	186360339	186360398	chr1:186360339-186360398	0.00	1.22	0.07
A_16_P35399966	186394515	186394574	chr1:186394515-186394574	0.04	0.88	0.20
A_16_P15383233	186417335	186417394	chr1:186417335-186417394	0.27	0.85	0.25
A_16_P15383354	186476579	186476638	chr1:186476579-186476638	0.11	0.69	0.38
A_14_P109148	186502356	186502415	chr1:186502356-186502415	-0.13	-0.22	0.15
A_14_P109148	186502356	186502415	chr1:186502356-186502415	-0.15	0.28	0.30
A_14_P109148	186502356	186502415	chr1:186502356-186502415	-0.21	0.97	0.24
A_16_P35400320	186565378	186565437	chr1:186565378-186565437	0.20	1.22	0.26
A_16_P15383587	186587164	186587223	chr1:186587164-186587223	0.05	1.13	0.49
A_16_P35400507	186655987	186656046	chr1:186655987-186656046	0.70	0.97	0.33
A_16_P00216723	186688932	186688991	chr1:186688932-186688991	0.31	1.35	0.22
A_14_P132515	186736991	186737050	chr1:186736991-186737050	0.07	0.79	0.15
A_16_P15383957	186760496	186760555	chr1:186760496-186760555	0.69	1.28	0.48
A_16_P15384005	186783621	186783680	chr1:186783621-186783680	0.04	1.07	0.26
A_16_P35400806	186791473	186791532	chr1:186791473-186791532	0.13	0.81	0.30
A_16_P00216876	186798591	186798650	FAM5C	-0.12	1.07	0.29
A_16_P00216889	186804354	186804413	FAM5C	0.26	0.70	0.39
A_16_P15384092	186811651	186811710	FAM5C	0.29	0.58	0.03
A_16_P15384110	186817791	186817850	FAM5C	0.19	1.54	0.31
A_16_P15384134	186824557	186824616	FAM5C	0.25	0.44	0.18
A_16_P15384157	186832030	186832088	FAM5C	0.02	0.58	0.32
A_16_P15384177	186838656	186838715	FAM5C	0.11	0.91	0.17
A_16_P35400992	186847100	186847159	FAM5C	0.19	0.39	-0.03
A_16_P15384234	186854915	186854974	FAM5C	0.01	1.10	0.30
A_16_P15384247	186861579	186861638	FAM5C	0.56	0.73	0.35
A_16_P15384288	186873156	186873215	FAM5C	0.50	1.28	-0.03
A_14_P108334	186879996	186880055	FAM5C	0.13	1.53	0.55

A_16_P15384336	186895841	186895900	FAM5C	0.16	1.25	0.24
A_16_P15384366	186909141	186909200	FAM5C	0.35	1.16	-0.22
A_16_P15384388	186916468	186916527	FAM5C	0.31	1.13	0.40
A_16_P35401185	186928941	186929000	FAM5C	0.20	1.17	0.38
A_16_P35401203	186935263	186935322	FAM5C	0.56	1.35	0.27
A_16_P15384452	186943463	186943522	FAM5C	0.35	1.55	0.15
A_16_P15384473	186949978	186950037	FAM5C	0.70	1.25	0.48
A_16_P15384488	186959556	186959605	FAM5C	0.58	0.11	0.40
A_16_P35401274	186965602	186965661	FAM5C	0.51	1.37	0.24
A_16_P15384531	186973669	186973728	FAM5C	0.25	1.27	0.31
A_16_P00217178	186982361	186982420	FAM5C	0.29	1.74	0.40
A_16_P15384592	186990972	186991031	FAM5C	0.32	0.24	0.09
A_16_P15384614	187008757	187008816	FAM5C	0.47	0.66	0.39
A_16_P35401421	187019907	187019966	FAM5C	0.28	0.85	0.11
A_16_P15384675	187035366	187035425	FAM5C	0.12	0.52	0.21
A_16_P15384696	187041429	187041488	FAM5C	0.24	0.27	0.22
A_14_P136363	187051151	187051210	FAM5C	-0.49	-0.14	0.08
A_16_P15384753	187060875	187060934	FAM5C	0.60	0.72	0.38
A_16_P15384773	187070521	187070580	FAM5C	0.32	0.91	0.40
A_16_P15384786	187076445	187076504	FAM5C	0.19	1.03	0.20
A_16_P15384806	187084123	187084182	FAM5C	0.01	-0.01	0.04
A_16_P00217341	187092733	187092792	FAM5C	0.04	0.99	0.16
A_16_P15384847	187100570	187100629	FAM5C	-0.16	0.44	0.20
A_16_P15384870	187107909	187107968	FAM5C	0.32	1.17	0.22
A_16_P15384906	187119506	187119565	FAM5C	0.21	1.36	0.19
A_16_P15384948	187130836	187130895	FAM5C	0.23	0.58	0.10
A_16_P00217441	187141425	187141484	FAM5C	0.01	0.29	0.17
A_16_P15385003	187148696	187148755	FAM5C	-0.09	0.66	0.02
A_16_P00217467	187155414	187155473	FAM5C	0.55	1.39	0.22
A_16_P15385066	187167428	187167487	FAM5C	0.04	1.01	0.09
A_16_P00217520	187178260	187178319	FAM5C	0.30	0.79	0.23
A_14_P103178	187191289	187191348	chr1:187191289-187191348	-0.21	0.06	-0.03
A_16_P15385212	187216480	187216539	chr1:187216480-187216539	0.18	0.89	0.25
A_16_P15385274	187241129	187241188	chr1:187241129-187241188	0.19	1.20	0.32
A_16_P15385330	187266790	187266849	chr1:187266790-187266849	0.14	0.58	0.18
A_16_P15385426	187303579	187303638	chr1:187303579-187303638	0.30	0.35	0.02
A_16_P15385474	187324071	187324130	chr1:187324071-187324130	-0.05	-0.48	0.23
A_16_P35402286	187342912	187342971	BC042042	0.38	0.39	0.26
A_14_P125984	187356080	187356139	BC042042	-0.75	-0.38	0.00
A_16_P15385570	187376321	187376380	BC042042	0.55	1.44	0.33
A_16_P15385608	187388911	187388970	BC042042	-0.08	0.50	0.32
A_16_P35402431	187406850	187406909	BC042042	0.22	0.76	0.27
A_16_P35402455	187418367	187418426	BC042042	0.62	0.48	0.12
A_16_P35402474	187437244	187437303	BC042042	0.02	0.57	0.00
A_16_P15385721	187448755	187448814	BC042042	0.30	0.64	0.20
A_16_P35402565	187466198	187466257	BC042042	0.28	0.29	0.35
A_16_P15385807	187477406	187477465	BC042042	0.35	0.77	0.33
A_14_P102910	187502078	187502137	BC042042	0.74	0.99	0.37

A_16_P15385879	187517062	187517121	chr1:187517062-187517121	-0.03	0.52	0.39
A_16_P15385925	187544487	187544546	chr1:187544487-187544546	0.75	0.61	0.44
A_16_P35402747	187567971	187568030	chr1:187567971-187568030	0.31	0.26	0.44
A_16_P00218091	187598457	187598516	chr1:187598457-187598516	-0.15	0.91	0.21
A_16_P35402910	187646178	187646237	chr1:187646178-187646237	0.67	1.20	0.36
A_14_P111181	187700885	187700944	chr1:187700885-187700944	0.35	0.70	0.39
A_16_P15386272	187722611	187722670	chr1:187722611-187722670	0.47	1.66	0.43
A_16_P35403156	187766137	187766196	chr1:187766137-187766196	0.21	0.28	0.39
A_16_P15386435	187833816	187833875	chr1:187833816-187833875	0.49	1.37	0.25
A_14_P137255	187895718	187895777	chr1:187895718-187895777	0.03	0.59	0.29
A_16_P00218460	187929188	187929247	chr1:187929188-187929247	0.73	0.98	0.41
A_16_P15386651	187958899	187958958	chr1:187958899-187958958	0.32	0.48	0.38
A_16_P15386802	188029323	188029382	chr1:188029323-188029382	0.27	1.64	0.08
A_16_P35403701	188064786	188064845	chr1:188064786-188064845	-0.08	1.35	0.38
A_16_P15386930	188096764	188096823	chr1:188096764-188096823	0.10	0.57	0.15
A_14_P127699	188120217	188120276	chr1:188120217-188120276	-0.26	1.15	0.37
A_16_P35403865	188157102	188157161	chr1:188157102-188157161	0.66	1.14	0.28
A_16_P15387180	188214835	188214894	chr1:188214835-188214894	0.66	0.73	0.39
A_16_P15387232	188240631	188240690	chr1:188240631-188240690	0.78	1.45	0.43
A_16_P15387335	188274625	188274684	chr1:188274625-188274684	0.59	0.52	0.43
A_14_P105473	188299952	188300011	chr1:188299952-188300011	-0.01	1.05	0.36
A_16_P35404273	188321880	188321939	chr1:188321880-188321939	0.74	1.66	0.43
A_16_P15387581	188376200	188376259	chr1:188376200-188376259	0.33	0.94	0.44
A_16_P15387656	188421294	188421353	chr1:188421294-188421353	0.48	1.42	0.52
A_16_P35404545	188448909	188448968	chr1:188448909-188448968	0.32	0.60	0.10
A_14_P112830	188491263	188491322	chr1:188491263-188491322	0.08	0.61	0.18
A_16_P35404716	188523096	188523155	chr1:188523096-188523155	0.00	0.50	0.13
A_16_P15387950	188558282	188558341	chr1:188558282-188558341	0.06	0.67	0.12
A_16_P15387994	188583466	188583525	BC042094	0.44	1.04	0.43
A_16_P35404865	188601885	188601944	BC042094	0.50	1.14	0.40
A_16_P15388064	188614363	188614422	BC042094	-0.07	0.66	0.27
A_16_P15388104	188642909	188642968	BC042094	-0.28	1.18	0.30
A_16_P15388113	188654750	188654809	BC042094	0.45	1.02	0.36
A_16_P15388151	188666902	188666961	BC042094	-0.30	0.39	-0.06
A_16_P35405005	188684853	188684912	BC042094	-0.12	1.11	0.22
A_14_P138574	188695069	188695128	BC042094	0.25	1.21	-0.04
A_16_P15388232	188707871	188707930	BC042094	0.28	0.96	0.25
A_16_P15388267	188723595	188723654	chr1:188723595-188723654	0.11	0.83	0.12
A_16_P35405183	188764199	188764258	chr1:188764199-188764258	0.59	0.78	0.24
A_16_P15388422	188787730	188787789	chr1:188787730-188787789	0.56	1.04	0.28
A_14_P122716	188824080	188824139	chr1:188824080-188824139	0.38	0.73	0.24
A_16_P15388560	188838874	188838933	chr1:188838874-188838933	0.30	1.18	0.22
A_16_P35405409	188849134	188849193	chr1:188849134-188849193	0.14	0.82	0.19
A_16_P15388609	188858990	188859049	chr1:188858990-188859049	0.40	1.26	0.13
A_16_P15388646	188870524	188870583	RGS18	0.11	0.32	0.16
A_16_P35405480	188876293	188876352	RGS18	0.52	0.89	0.36
A_14_P119196	188882142	188882197	RGS18	0.11	0.44	0.27
A_14_P202283	188885210	188885269	RGS18	0.48	0.93	0.20

A_16_P15388708	188895139	188895198	chr1:188895139-188895198	0.50	1.45	0.26
A_16_P15388725	188904113	188904172	chr1:188904113-188904172	0.14	1.13	0.17
A_16_P15388784	188937007	188937066	chr1:188937007-188937066	0.45	1.09	0.16
A_16_P15388823	188959544	188959603	chr1:188959544-188959603	0.00	1.06	0.05
A_16_P15388893	188984152	188984211	chr1:188984152-188984211	0.63	1.94	0.26
A_16_P35405759	189000074	189000133	chr1:189000074-189000133	0.57	1.06	0.31
A_14_P133864	189016546	189016605	chr1:189016546-189016605	0.36	0.91	0.05
A_16_P15388992	189023351	189023410	RGS21	0.81	0.87	0.34
A_16_P15389013	189030021	189030080	RGS21	0.23	0.74	0.33
A_16_P15389042	189040810	189040869	RGS21	0.30	0.51	0.23
A_16_P15389042	189040810	189040869	RGS21	0.36	1.02	0.16
A_16_P15389042	189040810	189040869	RGS21	0.37	-0.92	0.21
A_16_P15389067	189048061	189048120	RGS21	0.71	1.02	0.18
A_16_P15389103	189058693	189058752	RGS21	0.23	1.32	0.11
A_16_P15389131	189067678	189067737	RGS21	0.36	1.87	0.18
A_16_P15389162	189077292	189077351	chr1:189077292-189077351	0.44	1.40	0.16
A_16_P35406028	189090016	189090075	chr1:189090016-189090075	0.26	1.77	0.19
A_16_P35406065	189103542	189103601	chr1:189103542-189103601	0.48	2.03	0.05
A_14_P129986	189154524	189154583	chr1:189154524-189154583	0.56	1.58	0.37
A_16_P15389419	189190192	189190251	chr1:189190192-189190251	0.60	1.25	0.43
A_16_P00220138	189213213	189213272	chr1:189213213-189213272	0.10	0.83	0.12
A_16_P15389501	189231443	189231502	chr1:189231443-189231502	0.44	1.58	0.47
A_16_P00220206	189253672	189253731	chr1:189253672-189253731	0.52	0.58	0.13
A_16_P15389579	189266235	189266294	chr1:189266235-189266294	0.10	0.42	0.11
A_16_P15389598	189273710	189273769	chr1:189273710-189273769	0.92	1.18	0.40
A_14_P202511	189279099	189279158	RGS1	0.69	0.53	0.26
A_14_P123371	189279992	189280051	RGS1	0.50	0.75	0.28
A_16_P35406485	189286469	189286528	chr1:189286469-189286528	0.27	0.60	0.16
A_16_P15389676	189295927	189295986	chr1:189295927-189295986	0.36	1.37	0.30
A_16_P15389700	189302805	189302864	chr1:189302805-189302864	0.73	0.55	0.32
A_16_P00220310	189310486	189310545	chr1:189310486-189310545	0.93	1.05	0.27
A_16_P15389736	189318468	189318527	chr1:189318468-189318527	0.07	1.69	0.35
A_16_P15389760	189329179	189329238	chr1:189329179-189329238	0.28	0.12	0.32
A_14_P138017	189337051	189337110	RGS13	0.94	1.27	0.31
A_16_P35406677	189350527	189350586	RGS13	0.52	1.58	0.58
A_14_P113910	189355740	189355799	RGS13	0.44	1.44	0.20
A_16_P35406713	189360977	189361036	RGS13	0.17	1.08	0.07
A_16_P35406724	189370559	189370618	chr1:189370559-189370618	0.78	1.07	0.41
A_16_P15389899	189380840	189380899	chr1:189380840-189380899	0.68	2.08	0.52
A_16_P35406768	189392062	189392121	chr1:189392062-189392121	0.55	1.69	0.33
A_16_P00220447	189410243	189410302	chr1:189410243-189410302	0.34	1.31	0.22
A_14_P130798	189428816	189428875	chr1:189428816-189428875	-0.43	1.68	0.03
A_16_P35406910	189447420	189447479	chr1:189447420-189447479	0.60	0.95	-0.01
A_16_P15390103	189468289	189468348	chr1:189468289-189468348	0.78	1.32	0.39
A_16_P15390139	189487827	189487886	chr1:189487827-189487886	0.57	0.28	0.43
A_16_P00220569	189495456	189495515	chr1:189495456-189495515	1.00	1.34	0.51
A_16_P15390173	189500657	189500716	chr1:189500657-189500716	0.66	0.44	0.41
A_14_P131833	189511954	189512013	RGS2	0.06	0.77	0.22

A_16_P00220605	189515914	189515973	chr1:189515914-189515973	0.40	0.97	0.35
A_16_P15390242	189521211	189521270	chr1:189521211-189521270	0.40	1.31	0.19
A_16_P15390250	189527530	189527589	chr1:189527530-189527589	0.14	0.77	0.28
A_16_P00220636	189536931	189536990	chr1:189536931-189536990	0.56	0.85	0.36
A_14_P113547	189575028	189575087	chr1:189575028-189575087	0.85	3.77	-0.19
A_16_P00220786	189631543	189631602	chr1:189631543-189631602	0.82	1.01	0.40
A_16_P35407491	189675680	189675739	chr1:189675680-189675739	0.21	1.96	0.54
A_16_P35407531	189697253	189697312	chr1:189697253-189697312	0.27	0.62	0.24
A_16_P15390690	189708969	189709028	chr1:189708969-189709028	0.42	0.66	0.14
A_14_P100344	189716940	189716999	UCHL5	0.52	0.85	0.22
A_14_P100536	189721094	189721153	UCHL5	0.33	0.95	0.29
A_16_P15390753	189730018	189730077	UCHL5	0.46	1.39	0.20
A_14_P130683	189748066	189748125	UCHL5	0.89	1.66	0.82
A_16_P00220951	189759138	189759197	UCHL5	0.03	0.78	0.03
A_14_P112927	189766169	189766228	TROVE2	0.54	1.02	0.26
A_16_P15390857	189777699	189777758	TROVE2	0.59	1.52	0.42
A_16_P15390872	189785596	189785655	TROVE2	0.13	1.29	0.48
A_14_P129953	189792428	189792487	AY205314	0.49	0.86	0.33
A_16_P15390910	189797191	189797250	chr1:189797191-189797250	0.61	0.44	0.16
A_14_P121761	189801952	189802011	GLRX2	0.36	1.15	0.25
A_16_P35407808	189806574	189806633	GLRX2	0.63	0.38	0.40
A_16_P15390944	189810865	189810924	chr1:189810865-189810924	0.24	1.81	0.82
A_14_P115098	189823021	189823068	CDC73	0.84	1.46	0.48
A_16_P15390996	189836227	189836286	CDC73	0.74	1.05	0.33
A_16_P35407886	189842653	189842712	CDC73	0.38	1.23	0.33
A_14_P138936	189851061	189851120	CDC73	0.37	0.23	0.33
A_16_P15391075	189862186	189862245	CDC73	0.21	0.43	0.41
A_16_P15391101	189873425	189873484	CDC73	0.91	1.29	0.41
A_14_P118550	189883489	189883548	CDC73	0.65	1.10	0.44
A_16_P35408036	189890120	189890179	CDC73	0.05	0.04	0.12
A_16_P15391167	189895354	189895413	CDC73	0.53	0.66	0.35
A_16_P15391172	189900011	189900070	CDC73	0.25	1.20	0.29
A_14_P104197	189904555	189904612	CDC73	0.35	0.76	0.38
A_16_P00221185	189913149	189913208	CDC73	0.35	0.27	0.53
A_16_P00221196	189922557	189922616	CDC73	0.32	0.32	0.16
A_14_P101440	189933762	189933821	CDC73	0.39	0.91	0.07
A_14_P106218	189951899	189951958	CDC73	0.24	0.69	0.07
A_16_P00221245	189963418	189963477	chr1:189963418-189963477	0.43	0.56	0.22
A_16_P35408213	189970828	189970887	chr1:189970828-189970887	0.50	0.89	0.22
A_16_P15391362	189992186	189992245	chr1:189992186-189992245	0.59	0.70	0.59
A_14_P130498	190009274	190009333	chr1:190009274-190009333	1.19	1.82	0.30
A_16_P00221325	190036226	190036285	chr1:190036226-190036285	0.51	0.14	0.50
A_16_P15391561	190091014	190091073	chr1:190091014-190091073	0.61	1.40	0.35
A_16_P15391674	190136125	190136184	chr1:190136125-190136184	0.57	1.56	0.46
A_16_P15391726	190162779	190162838	chr1:190162779-190162838	1.05	0.80	0.43
A_16_P15391801	190192211	190192270	chr1:190192211-190192270	-0.02	1.48	0.00
A_14_P116046	190221121	190221180	chr1:190221121-190221180	-0.20	0.33	0.30
A_16_P00221591	190243158	190243217	chr1:190243158-190243217	0.76	0.76	0.26

A_16_P15391947	190255019	190255078	chr1:190255019-190255078	-0.04	1.05	0.06
A_16_P15391985	190269146	190269205	chr1:190269146-190269205	0.55	0.87	0.33
A_16_P35408883	190279913	190279972	chr1:190279913-190279972	-0.19	0.81	0.21
A_16_P15392058	190295933	190295992	chr1:190295933-190295992	-0.09	0.68	0.25
A_16_P35408990	190325451	190325510	chr1:190325451-190325510	0.60	0.93	0.15
A_16_P15392184	190365890	190365949	chr1:190365890-190365949	0.34	0.99	0.18
A_16_P35409138	190397297	190397356	chr1:190397297-190397356	0.95	1.41	0.26
A_14_P123369	190431171	190431230	chr1:190431171-190431230	0.31	0.79	0.25
A_16_P15392397	190447703	190447762	chr1:190447703-190447762	0.63	0.94	0.46
A_16_P00221914	190457595	190457654	chr1:190457595-190457654	-0.21	0.64	-0.02
A_16_P15392425	190464773	190464832	chr1:190464773-190464832	-0.02	1.43	1.23
A_16_P00221931	190470615	190470674	chr1:190470615-190470674	0.46	0.91	0.32
A_16_P35409335	190480229	190480288	chr1:190480229-190480288	0.35	-0.03	0.14
A_16_P15392509	190498171	190498230	chr1:190498171-190498230	0.42	0.89	0.42
A_16_P35409401	190507649	190507708	chr1:190507649-190507708	0.59	0.40	0.16
A_16_P35409425	190515374	190515433	chr1:190515374-190515433	0.36	0.72	0.22
A_16_P15392577	190532905	190532964	chr1:190532905-190532964	0.16	0.95	0.04
A_16_P15392599	190540519	190540578	chr1:190540519-190540578	0.28	0.09	0.24
A_16_P15392636	190554983	190555042	chr1:190554983-190555042	0.16	0.87	0.20
A_14_P121909	190610504	190610563	chr1:190610504-190610563	1.23	1.93	0.67
A_16_P35409679	190638750	190638809	chr1:190638750-190638809	0.26	-0.78	0.29
A_16_P15392861	190663528	190663587	chr1:190663528-190663587	0.47	1.09	0.27
A_16_P15392962	190714904	190714963	chr1:190714904-190714963	0.09	0.76	0.33
A_16_P15393020	190744586	190744645	chr1:190744586-190744645	0.07	0.98	0.14
A_16_P15393092	190774085	190774144	chr1:190774085-190774144	0.44	0.57	0.20
A_16_P15393158	190798792	190798851	chr1:190798792-190798851	0.26	1.57	0.16
A_16_P15393219	190828310	190828369	chr1:190828310-190828369	0.21	0.87	0.09
A_14_P111802	190865439	190865498	chr1:190865439-190865498	0.45	1.14	0.25
A_16_P15393391	190898577	190898636	chr1:190898577-190898636	0.07	0.72	0.05
A_16_P15393465	190937446	190937505	chr1:190937446-190937505	-0.11	1.28	0.07
A_16_P35410429	190989056	190989115	chr1:190989056-190989115	0.31	0.88	0.18
A_16_P15393732	191041702	191041761	chr1:191041702-191041761	0.17	1.00	0.06
A_16_P15393812	191073675	191073734	chr1:191073675-191073734	0.56	0.92	0.24
A_14_P133903	191102381	191102440	chr1:191102381-191102440	0.32	2.45	0.23
A_16_P15393931	191133804	191133863	chr1:191133804-191133863	0.63	1.65	0.29
A_16_P15393992	191161542	191161601	chr1:191161542-191161601	0.26	1.04	-0.11
A_16_P15394100	191204237	191204296	chr1:191204237-191204296	0.55	0.61	0.13
A_16_P15394167	191228197	191228256	chr1:191228197-191228256	-0.56	1.12	0.02
A_16_P15394223	191261475	191261534	chr1:191261475-191261534	0.20	1.24	0.11
A_14_P126531	191314937	191314996	chr1:191314937-191314996	0.44	1.16	0.16
A_16_P15394399	191350026	191350085	chr1:191350026-191350085	0.01	1.05	-0.05
A_16_P00223100	191376714	191376772	chr1:191376714-191376772	0.68	0.66	0.10
A_16_P15394522	191401434	191401493	chr1:191401434-191401493	0.53	0.64	0.12
A_14_P134949	191435219	191435278	chr1:191435219-191435278	0.22	-0.04	0.11
A_16_P15394725	191487816	191487875	chr1:191487816-191487875	0.07	1.79	0.25
A_16_P00223310	191510690	191510749	chr1:191510690-191510749	0.50	0.82	0.37
A_16_P15394864	191540168	191540227	chr1:191540168-191540227	-0.01	1.58	-0.16
A_14_P130129	191571308	191571367	chr1:191571308-191571367	0.45	1.08	0.50

A_16_P35411815	191594400	191594459	chr1:191594400-191594459	0.23	-0.25	0.32
A_16_P15395037	191619487	191619546	chr1:191619487-191619546	0.31	0.72	0.26
A_16_P15395077	191641567	191641626	chr1:191641567-191641626	0.42	0.34	0.21
A_16_P15395125	191666750	191666809	chr1:191666750-191666809	0.05	0.93	0.44
A_16_P35412040	191699476	191699535	chr1:191699476-191699535	0.41	0.94	0.59
A_16_P15395253	191721683	191721742	chr1:191721683-191721742	-0.02	-1.06	-0.06
A_16_P15395313	191750249	191750308	chr1:191750249-191750308	0.19	0.62	0.32
A_14_P134297	191782973	191783032	chr1:191782973-191783032	0.08	1.19	0.31
A_16_P15395485	191830928	191830987	chr1:191830928-191830987	-0.16	0.79	0.35
A_16_P15395651	191904130	191904189	chr1:191904130-191904189	0.48	1.66	0.51
A_16_P15395719	191932213	191932272	chr1:191932213-191932272	0.26	1.17	0.38
A_14_P101944	191955321	191955380	chr1:191955321-191955380	0.85	1.60	0.33
A_16_P15395884	191998391	191998450	chr1:191998391-191998450	0.00	0.61	0.44
A_16_P00224038	192049070	192049129	chr1:192049070-192049129	0.24	0.54	0.28
A_16_P15396037	192075730	192075789	chr1:192075730-192075789	0.20	0.14	0.48
A_16_P15396121	192111050	192111109	chr1:192111050-192111109	0.17	-0.20	0.46
A_16_P15396195	192137769	192137828	chr1:192137769-192137828	0.24	1.59	0.25
A_14_P122448	192165283	192165342	chr1:192165283-192165342	0.45	0.93	0.36
A_16_P15396300	192190080	192190139	chr1:192190080-192190139	0.46	1.14	0.83
A_16_P35413199	192215096	192215155	chr1:192215096-192215155	0.48	1.68	0.25
A_16_P15396407	192245930	192245989	chr1:192245930-192245989	-0.10	1.25	0.71
A_16_P15396479	192297279	192297338	chr1:192297279-192297338	-0.33	1.08	0.01
A_16_P15396547	192335441	192335500	chr1:192335441-192335500	0.10	1.04	0.26
A_16_P35413441	192358835	192358894	chr1:192358835-192358894	0.12	1.02	0.28
A_14_P100783	192419316	192419375	chr1:192419316-192419375	1.03	1.20	0.65
A_16_P15396782	192441486	192441545	chr1:192441486-192441545	0.32	1.41	0.48
A_16_P15396889	192483933	192483992	chr1:192483933-192483992	0.19	0.65	0.36
A_16_P15397009	192534673	192534732	chr1:192534673-192534732	0.37	1.00	0.44
A_16_P15397052	192553342	192553401	chr1:192553342-192553401	-0.25	1.02	0.03
A_16_P15397107	192583172	192583231	chr1:192583172-192583231	0.57	1.21	0.48
A_16_P35414034	192616066	192616125	chr1:192616066-192616125	0.16	0.80	0.32
A_14_P105138	192645771	192645830	chr1:192645771-192645830	0.32	0.72	0.25
A_16_P15397408	192714776	192714835	chr1:192714776-192714835	0.44	0.32	0.45
A_16_P15397487	192752216	192752275	chr1:192752216-192752275	-0.03	0.69	0.26
A_16_P15397569	192790100	192790159	chr1:192790100-192790159	-0.01	0.63	0.63
A_14_P104171	192833855	192833914	chr1:192833855-192833914	0.46	0.76	0.42
A_16_P15397710	192870420	192870479	chr1:192870420-192870479	0.04	0.64	0.33
A_16_P00225084	192914626	192914685	chr1:192914626-192914685	0.19	0.97	0.31
A_16_P15397802	192926931	192926990	KCNT2	-0.19	1.10	0.25
A_16_P35414686	192939681	192939740	KCNT2	0.81	0.82	0.56
A_16_P35414717	192958958	192959017	KCNT2	0.40	0.84	0.25
A_16_P15397914	192976206	192976265	KCNT2	0.31	1.26	0.26
A_16_P00225174	192981729	192981788	KCNT2	0.63	0.97	0.43
A_16_P00225191	192990967	192991026	KCNT2	0.22	1.29	0.29
A_16_P35414814	192996746	192996805	KCNT2	-0.03	0.60	0.22
A_16_P15397974	193002416	193002475	KCNT2	-0.03	1.04	0.34
A_16_P15398003	193011049	193011108	KCNT2	0.25	0.91	0.38
A_16_P35414888	193020329	193020388	KCNT2	0.64	1.14	0.42

A_14_P125788	193026311	193026370	KCNT2	0.52	0.54	0.32
A_16_P35414928	193032027	193032086	KCNT2	0.42	0.95	0.28
A_16_P15398086	193041188	193041247	KCNT2	0.73	1.26	0.34
A_16_P00225293	193050045	193050104	KCNT2	0.35	0.09	0.54
A_16_P15398121	193056008	193056067	KCNT2	0.66	0.67	0.27
A_16_P35415011	193065148	193065207	KCNT2	0.03	1.28	0.31
A_16_P15398168	193072839	193072898	KCNT2	0.22	0.89	0.36
A_16_P15398189	193079894	193079953	KCNT2	-0.02	0.40	0.31
A_16_P15398221	193091827	193091886	KCNT2	0.38	0.87	0.41
A_16_P00225379	193099323	193099382	KCNT2	0.59	1.07	0.48
A_16_P15398260	193107717	193107776	KCNT2	0.04	0.58	0.33
A_16_P00225404	193118097	193118156	KCNT2	0.38	0.68	0.48
A_16_P15398312	193128811	193128870	KCNT2	0.43	0.63	0.49
A_16_P15398336	193138680	193138739	KCNT2	-0.08	1.42	0.79
A_16_P15398358	193145100	193145159	KCNT2	0.49	1.22	0.32
A_16_P15398380	193150957	193151016	KCNT2	0.36	0.88	0.35
A_16_P15398411	193162526	193162585	KCNT2	0.07	-0.77	0.45
A_16_P00225496	193169787	193169846	KCNT2	0.20	1.08	0.33
A_16_P15398472	193180001	193180060	KCNT2	0.31	1.01	0.34
A_16_P15398488	193186597	193186656	KCNT2	-0.07	0.99	0.19
A_16_P15398516	193194214	193194273	KCNT2	0.09	1.24	0.53
A_16_P15398537	193203046	193203105	KCNT2	0.46	1.23	0.31
A_16_P35415437	193213573	193213632	KCNT2	0.60	0.03	0.35
A_16_P15398590	193230094	193230153	KCNT2	-0.11	-1.12	0.32
A_16_P15398629	193242916	193242975	KCNT2	0.15	1.01	0.24
A_14_P107342	193250043	193250102	KCNT2	0.36	0.98	0.39
A_16_P35415532	193258301	193258360	KCNT2	0.14	0.87	0.27
A_16_P15398685	193268630	193268688	KCNT2	-0.09	1.17	0.15
A_16_P15398709	193275974	193276033	KCNT2	0.44	1.15	0.34
A_16_P35415619	193290533	193290592	KCNT2	0.13	0.57	0.36
A_16_P35415640	193299177	193299236	KCNT2	0.50	0.98	0.39
A_16_P15398810	193308702	193308761	KCNT2	0.37	1.30	0.46
A_16_P15398843	193320828	193320887	chr1:193320828-193320887	0.39	0.98	0.40
A_16_P35415747	193333838	193333897	chr1:193333838-193333897	0.22	1.16	0.34
A_16_P35415754	193339795	193339854	chr1:193339795-193339854	-0.32	1.09	0.14
A_16_P15398904	193348280	193348339	chr1:193348280-193348339	-0.15	0.66	0.44
A_14_P137451	193354776	193354835	CFH	0.13	0.09	0.34
A_16_P35415806	193360643	193360702	CFH	0.51	0.55	0.44
A_16_P35415842	193376701	193376760	CFH	0.64	1.26	0.41
A_14_P109763	193389323	193389382	CFH	0.12	0.85	0.36
A_16_P15399032	193402874	193402933	CFH	0.27	0.91	0.39
A_16_P15399039	193408807	193408866	CFH	0.39	0.74	0.54
A_14_P126644	193416358	193416417	CFH	0.21	0.39	0.41
A_16_P00225893	193427624	193427683	CFH	0.54	0.83	0.22
A_16_P00225903	193436658	193436717	CFH	-0.41	-0.04	0.54
A_14_P102601	193442747	193442806	CFH	0.23	0.58	0.32
A_16_P35415994	193443252	193443311	CFH	0.30	0.23	0.16
A_16_P00225921	193470554	193470613	chr1:193470554-193470613	-0.55	1.37	0.67

A_16_P15399174	193480150	193480209	CFHR3	-0.63	0.78	0.28
A_16_P00225941	193491737	193491796	CFHR3	-0.96	0.78	0.06
A_16_P00225945	193498586	193498645	chr1:193498586-193498645	-1.45	0.35	0.47
A_16_P15399241	193510905	193510954	chr1:193510905-193510954	0.09	0.72	0.20
A_16_P35416144	193513242	193513301	chr1:193513242-193513301	0.31	1.53	0.54
A_16_P35416170	193527058	193527117	CFHR1	-1.29	0.10	0.12
A_16_P35416187	193532798	193532857	CFHR1	0.15	0.13	0.03
A_16_P00225973	193556792	193556851	chr1:193556792-193556851	0.62	1.38	0.47
A_16_P15399344	193569241	193569300	chr1:193569241-193569300	0.29	0.51	0.40
A_16_P15399365	193583559	193583618	chr1:193583559-193583618	0.36	0.66	0.55
A_16_P00226000	193585863	193585922	chr1:193585863-193585922	-0.03	1.02	0.54
A_14_P136242	193594693	193594752	CFHR4	0.02	0.95	0.29
A_16_P35416312	193601733	193601792	CFHR4	0.43	0.85	0.44
A_16_P15399437	193609096	193609155	CFHR4	-0.25	1.41	0.46
A_14_P113247	193614996	193615055	CFHR4	0.28	0.87	0.34
A_16_P35416386	193623266	193623325	chr1:193623266-193623325	0.45	1.18	0.65
A_16_P35416401	193630262	193630321	chr1:193630262-193630321	-0.18	0.75	0.24
A_16_P35416442	193652057	193652116	CFHR2	0.67	1.54	0.57
A_14_P134359	193653717	193653776	CFHR2	0.49	1.48	0.58
A_14_P108283	193659769	193659828	CFHR2	-0.30	0.80	0.64
A_16_P15399569	193666777	193666836	chr1:193666777-193666836	0.41	1.31	0.53
A_16_P35416500	193671114	193671173	chr1:193671114-193671173	0.33	0.83	0.42
A_16_P15399595	193677043	193677102	chr1:193677043-193677102	0.40	0.77	0.36
A_16_P15399620	193686350	193686409	CFHR5	0.65	0.42	0.74
A_14_P125089	193696878	193696937	CFHR5	0.42	0.64	0.20
A_16_P00226137	193703383	193703442	CFHR5	-0.10	0.87	0.49
A_16_P15399673	193710137	193710196	CFHR5	0.44	0.53	0.36
A_16_P35416619	193719413	193719472	chr1:193719413-193719472	0.40	0.84	0.45
A_16_P15399698	193725645	193725704	chr1:193725645-193725704	0.14	0.66	0.36
A_14_P127569	193730939	193730998	chr1:193730939-193730998	0.60	1.59	0.67
A_16_P35416661	193740022	193740081	F13B	0.06	1.25	0.22
A_16_P00226191	193751553	193751612	F13B	0.52	0.61	0.36
A_14_P102418	193756527	193756586	F13B	-0.01	0.84	0.27
A_16_P15399771	193761243	193761302	F13B	0.35	0.67	0.40
A_16_P35416734	193767950	193768009	F13B	-0.07	1.24	0.26
A_16_P15399795	193777367	193777426	chr1:193777367-193777426	0.08	1.50	0.20
A_14_P139792	193785053	193785112	BX648804	-0.80	0.39	-0.07
A_14_P121775	193785182	193785241	ASPM	0.12	0.65	0.37
A_16_P15399825	193792825	193792884	ASPM	0.53	0.54	0.49
A_16_P35416819	193805851	193805910	ASPM	0.20	0.50	0.46
A_16_P35416826	193814505	193814564	ASPM	0.75	1.15	0.52
A_16_P00226286	193823163	193823222	ASPM	0.74	0.88	0.47
A_14_P128896	193829976	193830035	ASPM	0.51	1.39	0.40
A_14_P116236	193843232	193843291	ASPM	0.31	1.03	0.31
A_14_P117907	193860493	193860552	ZBTB41	0.22	1.01	0.38
A_16_P15400028	193875745	193875804	ZBTB41	0.82	1.10	0.52
A_16_P15400057	193884928	193884987	ZBTB41	0.22	0.64	0.38
A_14_P108365	193892811	193892870	ZBTB41	0.38	0.96	0.37

A_14_P105959	193901152	193901210	ZBTB41	0.48	1.19	0.08
A_16_P35417094	193911956	193912015	chr1:193911956-193912015	0.36	0.57	0.57
A_16_P15400115	193918991	193919050	chr1:193918991-193919050	-0.15	0.77	0.53
A_16_P15400145	193931284	193931343	chr1:193931284-193931343	0.43	1.21	0.50
A_16_P15400160	193948178	193948237	chr1:193948178-193948237	0.30	0.85	0.54
A_16_P15400190	193958465	193958524	chr1:193958465-193958524	0.90	0.78	0.53
A_14_P100727	193969184	193969243	CRB1	0.87	1.11	0.55
A_16_P35417223	193976329	193976388	CRB1	0.47	0.98	0.34
A_16_P00226513	193987018	193987077	CRB1	0.70	0.92	0.68
A_16_P35417275	193997257	193997316	CRB1	0.45	0.88	0.44
A_14_P137546	194003668	194003727	CRB1	0.67	1.09	0.51
A_16_P15400330	194016789	194016848	CRB1	0.30	1.57	0.48
A_16_P35417354	194029260	194029319	CRB1	0.55	1.33	0.24
A_16_P15400390	194036620	194036679	CRB1	0.71	1.18	0.51
A_14_P122860	194045059	194045118	CRB1	0.90	1.19	0.66
A_16_P35417446	194058273	194058332	CRB1	0.59	1.46	0.40
A_16_P00226631	194068772	194068831	CRB1	0.32	0.69	0.42
A_16_P35417477	194078271	194078330	CRB1	-0.16	1.12	0.48
A_14_P133019	194088574	194088633	CRB1	0.04	1.75	0.19
A_16_P35417532	194095606	194095665	CRB1	0.34	0.84	0.45
A_16_P35417562	194108974	194109033	CRB1	0.16	1.52	0.44
A_16_P15400586	194115491	194115550	CRB1	0.05	-0.12	0.49
A_16_P00226718	194122232	194122291	CRB1	0.38	1.94	0.58
A_16_P15400625	194128699	194128758	CRB1	0.56	0.57	0.54
A_14_P103941	194135522	194135581	CRB1	0.50	1.67	0.57
A_16_P15400670	194143236	194143295	CRB1	0.39	0.93	0.50
A_16_P35417695	194152445	194152504	CRB1	0.68	0.53	0.36
A_16_P15400734	194166046	194166105	CRB1	0.45	0.46	0.45
A_16_P35417756	194171696	194171755	CRB1	0.89	1.37	0.57
A_14_P112401	194178811	194178870	CRB1	0.59	1.40	0.35
A_16_P15400801	194186658	194186717	chr1:194186658-194186717	0.80	0.73	0.39
A_16_P35417814	194194978	194195037	chr1:194194978-194195037	0.93	1.22	0.55
A_16_P15400848	194210484	194210543	FLJ20054	0.28	0.86	0.41
A_16_P15400886	194221754	194221813	FLJ20054	0.23	1.79	0.45
A_14_P121930	194230349	194230408	FLJ20054	0.31	0.83	0.25
A_14_P121930	194230349	194230408	FLJ20054	0.36	0.42	0.17
A_14_P121930	194230349	194230408	FLJ20054	0.43	0.81	0.16
A_16_P35417942	194238941	194239000	FLJ20054	0.01	0.62	0.29
A_14_P113746	194253779	194253838	FLJ20054	0.62	0.81	0.46
A_16_P35418016	194265818	194265877	FLJ20054	-0.01	0.23	0.29
A_16_P15401009	194271643	194271702	FLJ20054	0.50	1.39	0.48
A_14_P114088	194278504	194278563	FLJ20054	0.28	1.02	0.32
A_14_P114088	194278504	194278563	FLJ20054	0.13	1.31	0.65
A_14_P114088	194278504	194278563	FLJ20054	0.19	1.19	0.63
A_16_P15401040	194286726	194286785	DENND1B	0.19	1.24	0.48
A_16_P15401073	194297868	194297927	DENND1B	0.13	1.75	0.47
A_14_P126009	194306329	194306388	DENND1B	-0.28	1.19	0.35
A_16_P35418139	194312034	194312093	DENND1B	0.40	1.16	0.60

A_16_P35418185	194323955	194324014	DENND1B	0.28	1.05	0.41
A_16_P35418200	194335287	194335346	DENND1B	0.32	0.75	0.56
A_16_P15401199	194340831	194340890	DENND1B	0.52	0.46	0.48
A_16_P15401221	194347403	194347462	DENND1B	0.32	1.17	0.34
A_16_P00227122	194356630	194356689	DENND1B	0.30	0.88	0.65
A_16_P35418287	194362207	194362266	DENND1B	0.11	0.94	0.43
A_14_P107093	194372905	194372964	DENND1B	0.47	0.95	0.37
A_16_P15401330	194382787	194382846	DENND1B	0.42	1.01	0.56
A_16_P00227198	194394638	194394697	DENND1B	0.53	0.81	0.45
A_16_P35418417	194405135	194405194	DENND1B	0.38	0.51	0.49
A_14_P119281	194417978	194418037	DENND1B	0.50	1.30	0.26
A_16_P15401449	194431391	194431450	DENND1B	0.14	1.22	0.60
A_16_P35418504	194438315	194438374	DENND1B	0.30	0.97	0.53
A_16_P00227278	194447772	194447831	DENND1B	0.19	0.85	0.42
A_16_P00227282	194455840	194455899	DENND1B	0.23	1.00	0.50
A_16_P00227308	194468644	194468703	DENND1B	0.77	1.49	0.60
A_14_P133694	194475449	194475508	DENND1B	0.29	1.16	0.63
A_14_P202100	194477521	194477580	chr1:194477521-194477580	0.34	0.71	0.59
A_16_P35418639	194488228	194488287	chr1:194488228-194488287	0.78	1.64	0.47
A_16_P35418678	194526605	194526664	chr1:194526605-194526664	0.97	1.06	0.49
A_16_P15401735	194570793	194570852	chr1:194570793-194570852	0.12	0.68	0.35
A_16_P35418780	194586541	194586600	chr1:194586541-194586600	0.27	0.98	0.40
A_16_P15401756	194597222	194597281	chr1:194597222-194597281	0.92	1.29	0.46
A_16_P15401771	194602301	194602360	chr1:194602301-194602360	0.40	1.06	0.66
A_16_P15401792	194607927	194607986	C1orf53	0.31	1.02	0.70
A_16_P15401816	194614623	194614682	LHX9	1.06	1.19	0.65
A_14_P120904	194618206	194618265	LHX9	0.82	1.02	0.71
A_14_P124525	194621847	194621906	LHX9	0.66	1.18	0.63
A_16_P15401861	194626230	194626289	LHX9	0.71	1.54	0.72
A_16_P15401877	194630289	194630348	LHX9	0.29	1.57	0.62
A_16_P15401891	194634160	194634219	chr1:194634160-194634219	0.70	1.07	0.45
A_16_P15401927	194644391	194644450	chr1:194644391-194644450	0.47	1.09	0.59
A_16_P15401973	194663129	194663188	chr1:194663129-194663188	-0.01	1.45	0.59
A_14_P133778	194691494	194691553	chr1:194691494-194691553	0.24	0.63	0.46
A_16_P00227638	194725169	194725228	chr1:194725169-194725228	0.39	1.11	0.57
A_16_P35419243	194776804	194776863	chr1:194776804-194776863	-0.04	0.97	0.26
A_14_P135029	194844902	194844961	chr1:194844902-194844961	0.19	0.43	0.47
A_16_P15402446	194874391	194874450	CR614163	0.45	1.00	0.63
A_16_P15402500	194889876	194889935	CR614163	0.19	1.97	0.59
A_16_P35419581	194904265	194904324	CR614163	0.09	0.12	0.35
A_16_P35419605	194921340	194921399	CR614163	0.15	0.77	0.33
A_14_P118715	194933332	194933386	NEK7	0.42	1.03	0.49
A_16_P15402602	194940965	194941024	NEK7	0.48	0.43	0.32
A_14_P103773	194953867	194953926	NEK7	0.07	0.85	0.37
A_16_P35419704	194964854	194964913	NEK7	0.19	1.44	0.47
A_16_P15402696	194975448	194975507	NEK7	0.50	1.65	0.39
A_16_P00228004	194988635	194988694	NEK7	0.27	0.46	0.41
A_14_P117789	194997907	194997966	NEK7	0.71	1.48	0.57

A_16_P15402810	195009173	195009232	NEK7	0.91	0.84	0.58
A_14_P118223	195020336	195020395	NEK7	-2.23	0.79	0.21
A_16_P15402861	195031539	195031598	Y00062	0.46	0.82	0.54
A_16_P15402893	195041238	195041297	Y00062	0.89	0.62	0.62
A_16_P15402919	195051162	195051221	Y00062	0.45	0.61	0.68
A_16_P35420004	195063431	195063490	Y00062	0.51	0.50	0.27
A_16_P00228161	195073251	195073310	Y00062	0.58	0.79	0.69
A_16_P15403015	195083070	195083129	Y00062	0.07	0.59	0.52
A_16_P00228213	195097031	195097090	Y00062	0.47	1.81	0.69
A_16_P00228262	195125201	195125260	Y00062	0.41	1.37	0.53
A_14_P122196	195137450	195137509	Y00062	0.43	1.02	0.51
A_16_P15403232	195157163	195157222	Y00062	0.77	1.34	0.72
A_16_P35420313	195166921	195166980	Y00062	0.43	1.57	0.67
A_16_P15403292	195186425	195186484	Y00062	0.13	1.18	0.58
A_14_P114997	195196784	195196843	Y00062	0.36	0.81	0.91
A_16_P00228387	195215951	195216010	Y00062	0.53	0.67	0.60
A_14_P126903	195227586	195227645	ATP6V1G3	0.71	0.49	0.66
A_14_P109323	195230412	195230471	ATP6V1G3	0.85	1.47	0.86
A_16_P15403408	195237419	195237478	ATP6V1G3	0.08	0.20	0.75
A_16_P15403423	195241600	195241659	ATP6V1G3	0.30	0.99	0.43
A_16_P15403465	195255009	195255068	Y00062	0.66	0.89	0.60
A_16_P15403496	195265179	195265238	Y00062	0.77	1.33	0.62
A_16_P15403522	195275532	195275591	Y00062	0.04	0.65	0.55
A_16_P00228496	195297976	195298035	Y00062	0.77	0.34	0.71
A_14_P126533	195309301	195309360	Y00062	0.63	0.24	0.62
A_16_P15403581	195319867	195319926	Y00062	0.27	1.25	0.60
A_16_P15403621	195333446	195333505	Y00062	0.46	1.44	0.60
A_14_P101884	195339884	195339943	PTPRC	0.53	1.07	0.70
A_16_P35420735	195349816	195349875	PTPRC	0.27	1.52	0.75
A_16_P15403695	195356697	195356755	PTPRC	0.39	1.03	0.65
A_14_P133241	195363428	195363487	PTPRC	0.47	0.64	0.57
A_16_P00228627	195371427	195371486	PTPRC	0.27	1.00	0.70
A_16_P15403762	195377868	195377927	PTPRC	0.27	1.07	0.51
A_14_P138870	195384653	195384712	PTPRC	-0.04	0.82	0.72
A_16_P15403814	195393378	195393437	PTPRC	-0.14	0.22	0.45
A_16_P35420892	195398939	195398998	PTPRC	0.45	1.37	0.69
A_16_P15403854	195404690	195404749	PTPRC	0.46	1.20	0.62
A_16_P35420941	195410562	195410621	PTPRC	0.48	1.29	0.63
A_14_P114440	195423187	195423246	PTPRC	0.19	1.04	0.62
A_16_P00228757	195433146	195433205	PTPRC	0.47	1.11	0.85
A_16_P00228772	195444862	195444921	PTPRC	0.46	0.89	0.56
A_14_P136733	195453081	195453140	PTPRC	0.41	0.74	0.52
A_16_P15404019	195459873	195459932	chr1:195459873-195459932	0.06	0.84	0.78
A_16_P15404032	195466603	195466662	chr1:195466603-195466662	0.28	-0.70	0.53
A_16_P35421140	195474917	195474976	chr1:195474917-195474976	0.75	1.08	0.62
A_14_P138293	195487682	195487741	chr1:195487682-195487741	0.41	1.20	0.62
A_16_P15404165	195508526	195508585	chr1:195508526-195508585	0.64	1.01	0.75
A_16_P35421271	195523353	195523412	chr1:195523353-195523412	0.22	1.23	0.64

A_16_P15404230	195536440	195536499	chr1:195536440-195536499	0.88	0.97	0.84
A_16_P15404249	195543555	195543614	chr1:195543555-195543614	0.48	0.29	0.68
A_16_P15404269	195549473	195549532	chr1:195549473-195549532	0.34	0.75	0.61
A_16_P35421356	195556855	195556914	chr1:195556855-195556914	0.14	1.02	0.73
A_16_P00228965	195561400	195561459	chr1:195561400-195561459	0.62	1.87	0.68
A_16_P35421379	195563699	195563758	chr1:195563699-195563758	0.64	1.15	0.84
A_16_P15404334	195570594	195570653	chr1:195570594-195570653	0.14	1.40	0.61
A_16_P15404354	195578062	195578121	chr1:195578062-195578121	0.00	1.09	0.73
A_16_P35421440	195584216	195584275	chr1:195584216-195584275	0.42	0.76	0.77
A_16_P15404430	195599954	195600013	chr1:195599954-195600013	0.38	1.46	0.68
A_16_P15404526	195628532	195628591	chr1:195628532-195628591	0.20	0.77	0.52
A_14_P101188	195649583	195649642	chr1:195649583-195649642	0.16	1.00	0.45
A_16_P15404684	195689957	195690016	chr1:195689957-195690016	0.30	1.17	0.71
A_16_P35421827	195716545	195716604	BC040869	0.20	0.94	0.68
A_16_P15404799	195729104	195729163	chr1:195729104-195729163	0.17	1.08	0.69
A_16_P15404840	195744858	195744917	chr1:195744858-195744917	0.91	1.28	0.71
A_14_P107198	195767198	195767257	chr1:195767198-195767257	-0.71	0.40	0.55
A_16_P15404955	195801491	195801550	chr1:195801491-195801550	0.28	1.31	0.68
A_16_P35422062	195824969	195825028	chr1:195824969-195825028	0.48	0.56	0.52
A_16_P15405093	195855945	195856004	AK125573	0.75	1.42	0.72
A_16_P15405122	195866110	195866169	AK125573	0.50	1.02	0.74
A_16_P35422243	195885700	195885759	AK125573	-0.01	0.43	0.50
A_16_P35422305	195911979	195912038	AK125573	0.33	1.09	0.69
A_16_P15405263	195923583	195923642	AK125573	0.55	0.31	0.74
A_16_P15405331	195948982	195949041	AK125573	0.44	0.50	0.60
A_14_P115232	195961263	195961322	AK125573	0.53	1.50	0.63
A_16_P15405369	195972475	195972534	AK125573	-0.05	0.66	0.47
A_16_P00229628	195985017	195985076	AK125573	0.77	0.77	0.70
A_16_P15405442	195994631	195994690	AK125573	0.82	0.83	0.48
A_16_P15405486	196007998	196008056	AK125573	0.98	1.34	0.81
A_16_P15405518	196017788	196017847	AK125573	0.60	1.30	0.89
A_16_P00229709	196033910	196033969	AK125573	0.43	1.23	0.80
A_16_P35422611	196044238	196044297	AK125573	0.53	0.81	0.73
A_16_P15405588	196058536	196058595	AK125573	1.06	0.89	0.76
A_16_P15405609	196071353	196071412	AK125573	-0.58	0.21	0.60
A_16_P35422676	196080735	196080794	AK125573	0.15	0.72	0.61
A_14_P112474	196101103	196101162	chr1:196101103-196101162	0.13	0.87	0.57
A_16_P15405725	196149376	196149435	chr1:196149376-196149435	0.52	1.31	0.67
A_16_P00229883	196190499	196190558	chr1:196190499-196190558	0.29	0.74	0.65
A_14_P111321	196213688	196213747	chr1:196213688-196213747	0.19	0.71	0.48
A_16_P35422977	196252180	196252239	chr1:196252180-196252239	0.59	1.21	0.25
A_16_P15406031	196316592	196316651	chr1:196316592-196316651	0.40	0.56	0.71
A_14_P104038	196347444	196347503	chr1:196347444-196347503	0.40	0.75	0.57
A_16_P35423216	196375888	196375947	chr1:196375888-196375947	0.27	1.32	0.52
A_16_P35423517	196492343	196492402	chr1:196492343-196492402	0.55	0.96	0.81
A_14_P103851	196511064	196511123	chr1:196511064-196511123	0.26	0.31	0.55
A_16_P00230180	196556910	196556969	chr1:196556910-196556969	0.61	0.94	0.61
A_16_P15406695	196590447	196590506	chr1:196590447-196590506	0.70	1.34	0.65

A_16_P15406751	196616864	196616923	chr1:196616864-196616923	0.90	0.79	0.74
A_16_P00230275	196643920	196643979	chr1:196643920-196643979	1.01	1.10	0.95
A_14_P105424	196683843	196683902	chr1:196683843-196683902	0.59	0.20	0.68
A_16_P00230345	196703921	196703980	chr1:196703921-196703980	0.33	0.71	0.60
A_16_P15406936	196716876	196716935	chr1:196716876-196716935	0.47	0.29	0.66
A_14_P126842	196728434	196728493	NR5A2	0.42	1.50	0.83
A_16_P00230395	196734848	196734906	NR5A2	1.14	1.50	1.02
A_16_P00230421	196744548	196744607	NR5A2	0.12	0.86	0.59
A_16_P15407048	196750912	196750971	NR5A2	0.65	1.44	0.91
A_14_P131751	196759119	196759178	NR5A2	-0.23	0.39	0.38
A_16_P35424184	196773194	196773253	NR5A2	0.57	0.95	0.82
A_16_P35424202	196779235	196779294	NR5A2	0.81	1.16	0.81
A_16_P00230511	196790628	196790687	NR5A2	0.86	1.73	0.71
A_16_P15407210	196802417	196802476	NR5A2	0.53	0.96	0.95
A_16_P15407233	196809450	196809509	NR5A2	0.60	0.40	0.73
A_16_P35424340	196821526	196821585	NR5A2	0.51	0.70	0.77
A_14_P134233	196830061	196830120	NR5A2	0.58	1.20	0.68
A_16_P35424391	196838773	196838832	NR5A2	-0.17	1.28	0.64
A_16_P15407339	196845388	196845447	NR5A2	0.05	0.44	0.66
A_14_P116960	196856901	196856960	NR5A2	0.70	0.78	0.79
A_14_P109851	196876188	196876247	NR5A2	0.60	1.09	0.95
A_14_P202308	196876553	196876612	NR5A2	0.46	0.55	0.64
A_16_P35424529	196885719	196885778	chr1:196885719-196885778	0.60	0.48	0.70
A_16_P15407486	196899931	196899990	chr1:196899931-196899990	0.50	0.73	0.75
A_16_P00230719	196909158	196909217	chr1:196909158-196909217	0.98	0.27	0.87
A_16_P15407543	196923512	196923571	chr1:196923512-196923571	0.18	0.78	0.58
A_16_P00230761	196935953	196936012	chr1:196935953-196936012	-0.56	-0.70	0.63
A_16_P15407614	196947718	196947777	chr1:196947718-196947777	0.40	0.63	0.85
A_16_P35424766	196977244	196977303	chr1:196977244-196977303	0.48	0.69	0.77
A_14_P111077	197009284	197009343	chr1:197009284-197009343	0.99	0.32	0.88
A_16_P15407866	197049889	197049948	BC040731	0.65	0.12	0.79
A_16_P00230952	197061629	197061688	BC040731	0.66	0.64	0.79
A_16_P15407924	197074345	197074404	BC040731	0.57	0.51	0.82
A_16_P15407958	197084971	197085030	chr1:197084971-197085030	0.46	0.58	0.80
A_16_P15407982	197092804	197092863	chr1:197092804-197092863	1.11	0.84	0.84
A_16_P15407997	197099038	197099097	chr1:197099038-197099097	0.49	0.68	0.75
A_14_P119977	197107842	197107901	ZNF281	0.54	1.12	0.89
A_16_P35425115	197112191	197112250	chr1:197112191-197112250	0.53	0.78	0.84
A_16_P15408074	197122020	197122079	chr1:197122020-197122079	0.77	1.01	0.95
A_16_P00231064	197129097	197129156	chr1:197129097-197129156	0.47	0.98	0.77
A_16_P15408144	197153643	197153702	chr1:197153643-197153702	0.61	0.56	0.82
A_14_P118527	197191483	197191542	chr1:197191483-197191542	0.55	1.11	0.85
A_16_P15408303	197212863	197212922	chr1:197212863-197212922	0.54	0.50	0.52
A_16_P00231224	197228750	197228809	chr1:197228750-197228809	0.77	1.04	0.91
A_16_P15408387	197244131	197244190	chr1:197244131-197244190	0.96	1.25	1.04
A_16_P15408404	197253033	197253092	KIF14	0.58	0.98	0.74
A_16_P00231273	197260146	197260205	KIF14	0.84	1.61	1.05
A_14_P106756	197266217	197266276	KIF14	0.62	0.50	0.71

A_16_P35425543	197276358	197276417	KIF14	0.60	1.61	0.84
A_16_P15408493	197287009	197287068	KIF14	0.83	1.04	0.92
A_16_P00231324	197292882	197292941	KIF14	0.70	1.35	0.84
A_14_P118962	197299161	197299220	KIF14	0.47	0.95	0.72
A_16_P15408541	197304591	197304650	KIF14	1.15	1.44	0.88
A_14_P130355	197318869	197318928	KIF14	0.23	1.21	0.27
A_16_P15408610	197326808	197326867	DDX59	0.50	0.80	0.66
A_16_P35425725	197333483	197333542	DDX59	0.49	0.60	0.81
A_14_P110687	197345092	197345149	DDX59	0.25	1.66	0.51
A_16_P35425773	197351155	197351214	DDX59	0.53	1.12	0.72
A_16_P35425797	197359769	197359828	DDX59	-0.25	0.55	0.85
A_14_P134447	197367112	197367171	DDX59	0.92	-0.24	0.77
A_16_P00231451	197374191	197374250	BC042016	1.10	1.60	1.11
A_16_P35425840	197379844	197379903	BC042016	0.78	0.73	0.87
A_16_P15408770	197396731	197396790	chr1:197396731-197396790	0.27	1.05	0.85
A_16_P15408804	197412309	197412368	chr1:197412309-197412368	0.38	1.26	0.85
A_16_P15408851	197431325	197431384	chr1:197431325-197431384	0.84	0.95	0.89
A_16_P00231535	197439125	197439184	chr1:197439125-197439184	1.14	0.94	0.89
A_16_P00231554	197454337	197454396	CAMSAP1L1	0.72	0.95	0.74
A_16_P00231566	197461839	197461898	CAMSAP1L1	0.75	0.91	0.99
A_16_P35426043	197468433	197468492	CAMSAP1L1	0.45	0.92	0.91
A_16_P15408978	197481153	197481212	CAMSAP1L1	0.44	1.14	1.04
A_16_P00231611	197496177	197496236	CAMSAP1L1	0.59	1.12	0.97
A_14_P110992	197506412	197506471	CAMSAP1L1	-0.02	0.82	0.87
A_16_P00231625	197513125	197513184	CAMSAP1L1	0.38	0.76	0.99
A_16_P35426147	197519273	197519332	CAMSAP1L1	0.21	1.06	0.83
A_16_P35426184	197533580	197533639	CAMSAP1L1	0.17	1.31	0.62
A_16_P15409095	197540808	197540867	CAMSAP1L1	0.65	1.14	0.91
A_16_P00231684	197549463	197549522	CAMSAP1L1	-0.48	0.57	0.71
A_16_P15409152	197558801	197558860	CAMSAP1L1	0.91	1.35	0.86
A_16_P00231724	197567893	197567952	chr1:197567893-197567952	1.45	1.25	1.18
A_16_P15409191	197572542	197572601	chr1:197572542-197572601	0.99	0.56	0.91
A_16_P00231740	197577718	197577777	chr1:197577718-197577777	0.27	1.32	0.79
A_16_P00231752	197586556	197586615	chr1:197586556-197586615	0.75	1.64	0.92
A_14_P138242	197592310	197592363	C1orf106	0.76	0.94	0.75
A_14_P118757	197596962	197597021	C1orf106	0.71	0.02	0.85
A_16_P00231781	197602165	197602224	C1orf106	0.45	0.72	0.79
A_16_P15409286	197607355	197607414	C1orf106	0.89	0.69	0.90
A_14_P125493	197613189	197613248	C1orf106	0.64	0.66	0.82
A_16_P00231805	197618255	197618314	chr1:197618255-197618314	0.41	-0.57	0.94
A_16_P35426464	197623700	197623759	chr1:197623700-197623759	0.61	0.74	0.97
A_16_P00231827	197633607	197633666	chr1:197633607-197633666	0.80	0.92	0.74
A_16_P00231833	197648594	197648653	chr1:197648594-197648653	0.58	1.39	1.04
A_16_P35426515	197657123	197657182	chr1:197657123-197657182	0.35	1.05	0.82
A_16_P15409402	197664549	197664608	chr1:197664549-197664608	0.50	1.01	0.85
A_14_P137457	197672274	197672326	KIF21B	0.71	1.00	0.94
A_14_P136888	197681337	197681396	KIF21B	0.64	0.69	0.90
A_16_P00231916	197692202	197692257	KIF21B	0.92	0.79	0.93

A_16_P15409513	197698946	197699005	KIF21B	0.84	0.43	1.01
A_16_P00231945	197707400	197707456	KIF21B	0.87	1.27	0.85
A_16_P00231960	197715665	197715724	KIF21B	1.06	1.53	0.78
A_16_P00231987	197727360	197727419	AK022893	0.87	0.91	0.98
A_16_P00231997	197733715	197733774	chr1:197733715-197733774	0.87	1.54	0.85
A_16_P00231997	197733715	197733774	chr1:197733715-197733774	0.93	1.41	0.88
A_16_P00231997	197733715	197733774	chr1:197733715-197733774	0.89	1.67	0.85
A_16_P15409658	197747619	197747678	CACNA1S	0.95	1.01	0.98
A_16_P00232032	197753574	197753633	CACNA1S	0.47	1.10	1.02
A_14_P107001	197760942	197761001	CACNA1S	1.33	0.90	1.07
A_16_P00232066	197770871	197770930	CACNA1S	0.27	1.50	0.86
A_14_P134717	197785725	197785781	CACNA1S	0.58	0.88	1.01
A_16_P00232110	197796377	197796436	CACNA1S	0.43	0.30	0.98
A_16_P35427052	197810465	197810524	CACNA1S	0.94	1.27	1.03
A_14_P106884	197817878	197817937	chr1:197817878-197817937	0.45	0.21	0.70
A_16_P00232167	197824575	197824634	chr1:197824575-197824634	0.67	0.33	0.93
A_16_P15409909	197829580	197829639	chr1:197829580-197829639	0.37	0.53	0.76
A_14_P133574	197839039	197839098	TMEM9	0.88	-0.08	1.04
A_16_P00232222	197852558	197852616	TMEM9	1.33	1.14	0.81
A_16_P00232228	197856998	197857057	AY359012	0.30	0.85	0.95
A_16_P15410009	197865778	197865837	AY359012	0.51	0.68	0.93
A_16_P15410017	197870944	197871003	AY359012	0.87	0.34	0.48
A_16_P35427276	197883833	197883892	chr1:197883833-197883892	1.08	1.35	1.24
A_16_P00232285	197892030	197892089	BC033185	1.04	0.64	1.06
A_16_P00232306	197904077	197904136	BC033185	0.71	0.64	0.97
A_16_P15410160	197915159	197915218	DKFZp434B1231	1.21	1.02	1.07
A_14_P103397	197921734	197921793	DKFZp434B1231	0.58	1.71	0.64
A_16_P00232360	197929690	197929749	DKFZp434B1231	0.69	0.66	1.15
A_16_P15410222	197944302	197944361	chr1:197944302-197944361	0.04	1.01	0.97
A_16_P15410271	197965607	197965666	chr1:197965607-197965666	0.98	1.18	0.94
A_16_P00232408	197974794	197974853	chr1:197974794-197974853	1.19	1.37	1.01
A_14_P126075	197982932	197982991	chr1:197982932-197982991	0.35	0.98	0.82
A_16_P15410364	197995102	197995161	PKP1	0.97	1.58	0.81
A_16_P35427618	198002552	198002611	PKP1	0.61	0.64	0.96
A_16_P00232486	198012579	198012638	PKP1	0.72	0.83	1.04
A_14_P111437	198024769	198024828	PKP1	1.12	1.22	0.93
A_16_P15410502	198035307	198035366	chr1:198035307-198035366	0.28	1.17	0.67
A_16_P15410523	198047583	198047642	chr1:198047583-198047642	0.77	1.48	0.88
A_16_P35427778	198052898	198052957	chr1:198052898-198052957	0.77	0.81	0.87
A_14_P129845	198060406	198060461	TNNT2	0.14	1.13	0.75
A_16_P15410577	198064361	198064410	TNNT2	0.57	0.75	0.94
A_16_P00232585	198068196	198068248	TNNT2	0.60	0.40	0.76
A_14_P135734	198074022	198074068	TNNT2	-0.03	0.80	0.39
A_16_P15410643	198083724	198083783	LAD1	0.96	0.11	1.07
A_16_P00232626	198087968	198088023	LAD1	1.29	1.09	0.97
A_16_P00232629	198091841	198091900	LAD1	0.47	1.20	0.90
A_14_P110906	198098460	198098518	LAD1	-0.16	1.25	0.94
A_16_P00232651	198105228	198105287	TNNI1	0.66	1.20	1.20

A_16_P35427984	198111839	198111898	TNNI1	0.74	0.72	0.86
A_14_P111016	198119669	198119728	TNNI1	-0.06	1.21	0.77
A_16_P35428031	198124311	198124370	AK123965	0.77	1.56	0.97
A_16_P00232704	198131042	198131097	chr1:198131042-198131097	0.66	1.05	0.88
A_14_P108308	198138480	198138539	chr1:198138480-198138539	1.14	1.60	0.74
A_16_P00232713	198144403	198144462	chr1:198144403-198144462	1.06	0.40	0.87
A_16_P15410817	198151258	198151317	chr1:198151258-198151317	0.67	1.16	0.80
A_16_P00232725	198155307	198155366	chr1:198155307-198155366	0.91	0.64	1.03
A_16_P00232733	198160432	198160489	chr1:198160432-198160489	0.50	0.77	0.77
A_14_P123682	198168603	198168662	PHLDA3	0.60	0.81	1.01
A_16_P15410876	198172387	198172446	chr1:198172387-198172446	0.91	1.42	0.92
A_16_P00232755	198176102	198176161	AK130802	0.75	1.24	0.89
A_16_P00232762	198179954	198180013	AK130802	1.14	1.34	0.85
A_14_P110603	198184752	198184811	CSRP1	1.13	0.49	1.03
A_14_P139396	198192408	198192467	CSRP1	0.43	1.49	1.01
A_16_P00232808	198205191	198205250	CSRP1	0.81	0.60	0.85
A_16_P15411001	198214585	198214644	AK097932	0.15	0.90	0.95
A_16_P15411012	198222534	198222593	AK055590	0.90	0.90	1.23
A_16_P15411022	198233201	198233260	chr1:198233201-198233260	1.19	0.88	1.17
A_14_P132677	198241513	198241572	chr1:198241513-198241572	0.52	1.10	1.23
A_16_P00232896	198268339	198268398	chr1:198268339-198268398	0.73	1.44	0.81
A_16_P00232951	198306465	198306524	chr1:198306465-198306524	0.81	0.45	1.01
A_16_P00232990	198330979	198331038	chr1:198330979-198331038	0.32	0.56	0.79
A_16_P35428571	198338399	198338458	chr1:198338399-198338458	1.20	1.65	0.99
A_16_P00233016	198345394	198345453	chr1:198345394-198345453	1.51	1.39	1.08
A_16_P00233032	198354330	198354389	NAV1	1.46	1.23	1.20
A_16_P00233051	198363087	198363146	NAV1	1.19	1.85	0.88
A_16_P15411434	198377651	198377710	NAV1	1.08	0.71	1.03
A_14_P101960	198387204	198387263	NAV1	1.00	0.40	0.65
A_16_P15411492	198397801	198397860	NAV1	0.37	1.30	0.89
A_16_P00233123	198404133	198404192	NAV1	1.69	0.49	1.08
A_14_P102984	198411778	198411837	NAV1	0.91	1.83	1.06
A_16_P00233164	198422145	198422204	NAV1	0.85	1.54	1.15
A_14_P101978	198432497	198432551	NAV1	1.17	0.98	0.87
A_16_P15411610	198439618	198439677	NAV1	1.23	1.04	1.08
A_16_P00233201	198445625	198445684	NAV1	0.71	1.32	0.87
A_16_P15411663	198454171	198454230	NAV1	0.85	1.81	0.97
A_16_P15411700	198463771	198463830	NAV1	0.71	1.16	1.00
A_16_P00233261	198475159	198475218	NAV1	0.50	-0.77	-0.92
A_16_P00233268	198481516	198481575	NAV1	0.22	0.92	-0.66
A_16_P15411775	198489993	198490052	NAV1	0.99	0.97	-1.02
A_14_P137152	198495667	198495726	NAV1	0.59	0.16	-0.25
A_14_P107666	198504457	198504513	NAV1	1.03	1.31	-0.52
A_16_P00233347	198522687	198522746	NAV1	1.25	1.02	-0.85
A_16_P35429183	198531310	198531369	IPO9	0.30	0.42	-0.80
A_14_P112000	198539303	198539362	IPO9	0.64	0.69	-0.34
A_16_P00233389	198546977	198547036	IPO9	0.36	0.99	-0.94
A_14_P111979	198556611	198556670	IPO9	0.24	0.66	-1.07

A_16_P00233433	198567488	198567547	IPO9	0.33	0.37	-0.80
A_14_P129280	198577472	198577531	AK074740	0.34	0.91	-0.06
A_16_P00233463	198583971	198584030	AK055940	0.69	1.48	-0.63
A_16_P00233479	198591501	198591560	TMEM58	1.35	1.33	-0.50
A_16_P00233500	198600958	198601017	LMOD1	0.73	1.78	-0.61
A_14_P115312	198608295	198608354	LMOD1	0.49	0.35	-0.26
A_16_P35429472	198624169	198624228	LMOD1	0.79	0.64	-0.68
A_16_P35429483	198630269	198630328	LMOD1	1.09	1.42	-0.43
A_14_P116691	198638711	198638770	LMOD1	1.14	1.43	-0.58
A_16_P00233561	198645843	198645902	LMOD1	0.72	1.34	-0.28
A_16_P00233569	198650022	198650081	AF017307	0.26	1.12	-0.79
A_16_P15412217	198654101	198654160	AF017307	0.98	1.63	-0.78
A_16_P15412230	198658168	198658227	TIMM17A	0.17	1.02	-0.42
A_14_P104070	198664500	198664554	TIMM17A	1.29	1.43	-0.15
A_16_P35429587	198670975	198671034	TIMM17A	0.00	0.60	-0.88
A_16_P15412265	198678669	198678728	AF017307	0.20	1.86	-0.48
A_16_P00233610	198684446	198684505	RNPEP	1.25	1.40	-0.42
A_16_P15412286	198689950	198690009	RNPEP	0.76	1.52	-0.63
A_14_P106935	198696067	198696126	RNPEP	0.11	-0.03	-0.37
A_16_P00233637	198702454	198702513	RNPEP	0.71	1.78	-0.58
A_16_P00233655	198710905	198710958	ELF3	0.36	1.07	-0.47
A_16_P00233672	198717830	198717889	AK129946	0.51	0.65	-0.52
A_16_P00233681	198723672	198723727	AK129946	1.16	1.61	0.97
A_16_P15412390	198729283	198729342	AK129946	0.94	0.48	1.37
A_16_P15412402	198736744	198736803	AK129946	0.77	1.01	0.83
A_14_P108979	198748694	198748753	AK129946	0.75	0.97	0.89
A_16_P15412447	198767395	198767454	AK129946	1.37	1.10	1.09
A_16_P15412467	198780953	198781012	AK129946	0.80	-0.44	1.05
A_16_P15412488	198790912	198790971	AK129946	1.08	0.27	1.10
A_16_P15412501	198801836	198801895	AK129946	0.52	1.12	0.85
A_16_P00233761	198809705	198809764	AK129946	0.42	1.36	1.33
A_16_P00233772	198816324	198816383	AK129946	1.27	0.62	1.11
A_14_P200049	198825332	198825391	GPR37L1	0.54	0.61	0.96
A_14_P127784	198830249	198830300	GPR37L1	1.30	1.11	0.96
A_16_P00233797	198834310	198834369	ARL8A	0.45	1.15	0.92
A_14_P130083	198839858	198839917	ARL8A	1.12	1.24	0.97
A_16_P15412617	198846499	198846558	chr1:198846499-198846558	1.32	0.72	1.24
A_16_P00233823	198851395	198851450	PTPN7	1.34	1.18	1.10
A_14_P113453	198857078	198857136	PTPN7	0.29	0.73	0.97
A_14_P201961	198860435	198860490	PTPN7	0.91	1.48	1.26
A_16_P00233855	198870264	198870322	AJ629456	1.30	0.66	0.96
A_16_P15412718	198880213	198880272	AJ629456	0.54	0.92	1.02
A_16_P00233881	198886760	198886805	AJ629456	1.00	0.91	0.56
A_16_P35430169	198895988	198896032	LGR6	0.56	0.21	0.72
A_16_P00233908	198904789	198904846	LGR6	0.13	1.11	0.95
A_16_P15412808	198910356	198910415	LGR6	1.40	0.96	1.17
A_16_P00233924	198916165	198916223	LGR6	1.18	0.59	0.99
A_16_P00233933	198921939	198921994	LGR6	0.74	1.01	0.68

A_16_P00233946	198928164	198928223	LGR6	1.04	1.40	1.03
A_16_P35430290	198933704	198933763	LGR6	0.84	1.10	0.90
A_16_P00233967	198941470	198941529	LGR6	1.32	1.31	1.02
A_16_P00233978	198947703	198947762	LGR6	0.90	0.43	0.90
A_16_P15412940	198960345	198960404	LGR6	0.91	1.69	1.16
A_16_P35430373	198970899	198970958	LGR6	1.11	1.32	0.86
A_16_P15412986	198976554	198976613	LGR6	1.24	1.28	1.03
A_16_P00234025	198982183	198982242	LGR6	1.00	1.41	1.01
A_16_P00234046	198994576	198994635	LGR6	0.95	1.50	0.88
A_14_P125698	199006992	199007051	LGR6	0.51	0.81	0.76
A_16_P00234090	199016372	199016431	LGR6	0.92	1.65	0.80
A_16_P00234099	199022799	199022858	chr1:199022799-199022858	0.81	1.50	0.92
A_14_P109654	199032584	199032643	UBE2T	0.85	0.58	0.90
A_16_P15413171	199041703	199041762	UBE2T	1.04	0.59	1.06
A_16_P15413185	199047681	199047740	chr1:199047681-199047740	0.52	0.95	0.89
A_16_P15413205	199054142	199054201	PPP1R12B	0.67	1.48	0.78
A_14_P136403	199065829	199065888	PPP1R12B	-0.41	0.33	0.53
A_16_P15413253	199078488	199078547	PPP1R12B	0.80	1.53	0.95
A_16_P00234173	199087690	199087749	PPP1R12B	0.87	1.36	0.62
A_16_P15413287	199097312	199097371	PPP1R12B	0.87	-0.15	0.60
A_16_P15413298	199103486	199103538	PPP1R12B	1.13	1.43	0.90
A_16_P00234184	199111062	199111118	PPP1R12B	0.19	0.52	0.66
A_16_P15413341	199119748	199119807	PPP1R12B	0.66	0.78	0.73
A_14_P116999	199135441	199135500	PPP1R12B	0.28	0.53	0.95
A_16_P15413420	199143566	199143625	PPP1R12B	0.02	0.19	0.80
A_16_P15413443	199154489	199154548	PPP1R12B	0.51	1.29	1.48
A_16_P35430901	199163722	199163768	PPP1R12B	0.96	1.06	0.65
A_16_P00234242	199172908	199172967	PPP1R12B	0.82	0.68	0.24
A_16_P00234248	199194296	199194355	PPP1R12B	0.13	0.79	0.91
A_16_P00234256	199208949	199209008	PPP1R12B	0.95	0.68	1.02
A_16_P15413614	199222301	199222360	PPP1R12B	-0.38	2.12	0.91
A_16_P00234277	199251316	199251375	PPP1R12B	0.39	0.71	0.76
A_16_P35431180	199267238	199267297	PPP1R12B	1.07	0.44	0.91
A_16_P00234318	199273751	199273810	PPP1R12B	0.52	1.26	0.92
A_14_P113014	199286459	199286518	PPP1R12B	0.40	0.33	0.79
A_16_P00234365	199294243	199294302	AF093822	1.24	1.24	0.74
A_16_P15413863	199304996	199305055	SYT2	0.90	0.55	0.80
A_16_P15413882	199316962	199317021	SYT2	1.02	0.58	0.69
A_16_P00234397	199322524	199322569	SYT2	0.48	0.92	0.30
A_14_P110709	199329886	199329944	SYT2	1.00	0.47	0.64
A_16_P00234404	199336123	199336175	SYT2	1.19	1.30	0.60
A_16_P00234414	199345641	199345685	SYT2	0.93	0.90	0.28
A_14_P111530	199356111	199356170	SYT2	0.86	0.95	0.82
A_16_P00234442	199363774	199363832	SYT2	0.79	1.66	1.08
A_14_P127003	199374574	199374633	SYT2	0.57	-0.03	0.75
A_16_P15414082	199386490	199386549	SYT2	0.74	1.00	0.83
A_16_P00234493	199394085	199394144	SYT2	0.78	0.61	0.97
A_14_P108227	199407549	199407608	SYT2	1.13	1.43	1.14

A_16_P00234532	199413484	199413543	AF093822	0.94	0.56	1.01
A_16_P00234547	199420653	199420712	AF093822	1.09	2.08	0.94
A_14_P106017	199428155	199428214	AF093822	0.55	1.57	0.70
A_14_P108869	199430442	199430486	JARID1B	0.48	1.37	0.86
A_16_P00234593	199442474	199442533	JARID1B	0.47	1.16	0.82
A_16_P00234604	199449768	199449827	JARID1B	0.27	1.05	1.31
A_16_P15414308	199459272	199459331	JARID1B	0.33	0.93	0.83
A_14_P121081	199467368	199467424	JARID1B	1.03	0.76	1.02
A_16_P15414346	199472730	199472789	JARID1B	0.69	1.41	0.97
A_16_P15414382	199488996	199489055	JARID1B	0.67	0.61	1.08
A_16_P15414399	199495164	199495223	JARID1B	0.89	1.01	0.96
A_16_P15414423	199503055	199503114	JARID1B	0.86	0.79	0.90
A_14_P113742	199509421	199509480	AF093822	0.92	1.02	0.81
A_16_P15414468	199517013	199517072	AF093822	0.80	1.96	0.98
A_16_P15414474	199522498	199522557	AF093822	0.85	0.50	0.93
A_16_P00234737	199535319	199535378	AF093822	0.74	1.37	1.26
A_16_P15414521	199541352	199541410	AF093822	0.52	0.81	0.29
A_14_P125109	199552834	199552893	AF093822	0.41	1.40	0.84
A_16_P15414565	199558964	199559023	AF093822	0.93	1.22	0.99
A_16_P15414598	199573722	199573781	AF093822	0.88	0.04	0.93
A_14_P125914	199581092	199581151	RABIF	0.75	0.99	0.02
A_14_P102023	199586051	199586110	RABIF	0.84	1.07	0.92
A_14_P120503	199589065	199589124	RABIF	0.58	1.51	0.95
A_16_P15414662	199596800	199596859	KLHL12	1.15	1.04	1.11
A_16_P00234834	199601963	199602022	KLHL12	0.45	1.42	1.24
A_14_P101184	199609705	199609764	KLHL12	-0.03	2.49	0.66
A_14_P109659	199609881	199609936	KLHL12	0.63	0.56	0.80
A_16_P15414727	199620333	199620392	KLHL12	0.32	1.11	0.99
A_16_P35432249	199625983	199626042	KLHL12	0.48	1.12	0.89
A_16_P15414756	199631129	199631188	AF093822	0.75	0.75	0.99
A_16_P15414757	199636069	199636128	AF093822	0.73	1.33	0.83
A_14_P110783	199645747	199645806	ADIPOR1	0.11	0.39	0.59
A_16_P35432322	199654390	199654449	ADIPOR1	0.71	1.18	0.80
A_16_P00234925	199662866	199662925	CYB5R1	0.92	1.31	0.84
A_14_P109192	199666576	199666635	CYB5R1	0.51	0.47	0.80
A_16_P15414864	199675671	199675730	chr1:199675671-199675730	0.42	0.54	0.84
A_16_P35432401	199687669	199687728	chr1:199687669-199687728	1.35	1.34	1.16
A_16_P15414901	199694053	199694112	chr1:199694053-199694112	0.52	0.79	0.79
A_16_P15414915	199703206	199703265	chr1:199703206-199703265	0.59	1.07	1.06
A_14_P136920	199709391	199709450	C1orf37	0.23	0.70	0.48
A_16_P00234990	199717225	199717284	C1orf37	0.56	0.61	0.95
A_16_P35432495	199721512	199721571	C1orf37	0.23	0.45	0.95
A_16_P35432513	199726416	199726475	chr1:199726416-199726475	0.86	0.81	0.81
A_16_P15414999	199730552	199730611	chr1:199730552-199730611	0.52	0.84	0.99
A_16_P15415019	199735576	199735629	chr1:199735576-199735629	1.02	1.08	0.93
A_16_P15415035	199740126	199740185	chr1:199740126-199740185	0.66	1.24	0.62
A_14_P124066	199747522	199747581	chr1:199747522-199747581	0.38	0.56	0.74
A_16_P00235067	199758885	199758944	PPFIA4	1.09	0.66	0.99

A_16_P15415114	199764354	199764413	PPFIA4	0.41	0.76	0.92
A_16_P00235095	199772861	199772919	PPFIA4	0.23	0.28	0.52
A_16_P00235105	199778179	199778238	PPFIA4	0.36	1.19	0.70
A_14_P113133	199783993	199784049	MYOG	0.89	1.10	0.97
A_16_P15415192	199787662	199787719	chr1:199787662-199787719	0.67	0.26	0.69
A_16_P15415209	199792404	199792461	ADORA1	0.72	0.57	0.66
A_16_P15415216	199797913	199797965	ADORA1	1.05	0.49	1.04
A_14_P111607	199802584	199802643	ADORA1	1.16	0.74	0.93
A_16_P15415242	199812804	199812863	ADORA1	0.81	1.19	1.15
A_16_P00235165	199819406	199819462	ADORA1	1.00	0.88	1.15
A_16_P00235178	199826754	199826813	ADORA1	1.00	1.02	1.04
A_16_P00235197	199836546	199836605	ADORA1	1.15	-0.05	1.17
A_16_P35432883	199844534	199844593	ADORA1	0.91	1.17	1.14
A_14_P114626	199852256	199852315	ADORA1	1.16	1.08	1.09
A_16_P00235232	199856699	199856758	ADORA1	0.46	-0.37	0.92
A_16_P00235238	199863393	199863452	ADORA1	0.53	0.71	1.03
A_16_P00235258	199870640	199870699	MYBPH	1.10	0.85	1.14
A_14_P113229	199874862	199874917	MYBPH	1.44	1.43	1.05
A_14_P138133	199879753	199879803	CHI3L1	0.40	1.33	0.44
A_16_P00235292	199886365	199886424	CHI3L1	1.14	1.07	0.98
A_14_P130552	199892004	199892063	X75546	0.97	1.59	1.08
A_16_P00235313	199898856	199898915	X75546	0.90	0.44	0.67
A_16_P00235323	199904886	199904945	X75546	0.61	1.14	1.01
A_16_P00235332	199913061	199913120	X75546	1.24	1.70	0.90
A_14_P126010	199921952	199922011	CHIT1	0.26	0.30	0.66
A_14_P107125	199929623	199929682	CHIT1	1.02	0.90	1.02
A_16_P15415597	199940101	199940160	X75546	0.21	1.41	0.91
A_16_P00235386	199954049	199954108	X75546	0.22	0.23	1.05
A_16_P15415619	199969473	199969532	X75546	0.75	1.14	0.85
A_16_P00235422	199985730	199985789	X75546	0.92	0.70	0.97
A_16_P00235438	199993013	199993072	X75546	1.00	0.33	1.10
A_16_P35433302	199999436	199999495	X75546	0.76	0.57	0.98
A_14_P135005	200007708	200007763	BTG2	0.79	0.66	1.02
A_14_P118298	200008808	200008864	BTG2	0.24	1.07	0.76
A_14_P138354	200009843	200009902	BTG2	0.84	1.25	1.00
A_16_P15415771	200018829	200018888	X75546	0.68	0.76	0.84
A_16_P15415806	200029367	200029426	X75546	0.92	1.66	1.11
A_16_P00235509	200036683	200036742	X75546	0.79	1.42	1.07
A_14_P103608	200042740	200042799	FMOD	0.80	1.41	0.45
A_16_P00235531	200047977	200048036	FMOD	0.80	1.41	0.89
A_16_P00235540	200051782	200051840	FMOD	1.21	1.08	1.19
A_16_P00235540	200051782	200051840	FMOD	1.18	1.11	1.11
A_16_P00235540	200051782	200051840	FMOD	0.87	1.09	1.03
A_16_P00235553	200060574	200060633	chr1:200060574-200060633	0.99	1.27	1.08
A_16_P00235583	200079312	200079371	chr1:200079312-200079371	1.00	0.76	0.89
A_16_P15415969	200101162	200101221	chr1:200101162-200101221	0.55	1.26	0.82
A_14_P118261	200122652	200122711	chr1:200122652-200122711	0.05	0.12	0.94
A_16_P15416050	200142134	200142193	chr1:200142134-200142193	0.91	0.66	1.03

A_16_P00235674	200160182	200160241	chr1:200160182-200160241	0.67	0.13	0.92
A_16_P15416122	200169713	200169772	chr1:200169713-200169772	0.47	0.70	0.99
A_16_P15416146	200176848	200176907	PRELP	0.48	0.00	0.76
A_16_P35433752	200182082	200182141	PRELP	0.14	0.75	0.96
A_16_P00235732	200190816	200190875	PRELP	0.83	0.75	0.97
A_16_P00235738	200194259	200194317	chr1:200194259-200194317	1.12	1.59	1.01
A_16_P15416233	200201777	200201836	OPTC	0.99	1.08	0.91
A_16_P35433841	200206810	200206866	OPTC	0.57	1.29	0.75
A_16_P00235782	200213215	200213274	chr1:200213215-200213274	1.11	1.23	1.07
A_16_P35433893	200221713	200221772	chr1:200221713-200221772	0.84	0.53	0.99
A_16_P00235833	200245222	200245281	chr1:200245222-200245281	0.79	-0.58	0.78
A_16_P00235877	200271304	200271363	chr1:200271304-200271363	0.83	1.40	1.48
A_16_P15416532	200310054	200310113	chr1:200310054-200310113	0.76	0.71	0.88
A_16_P00235960	200325428	200325487	chr1:200325428-200325487	1.38	0.96	1.08
A_14_P104923	200337579	200337638	ATP2B4	1.01	1.42	1.06
A_16_P00235998	200349843	200349902	ATP2B4	0.39	1.09	0.61
A_16_P00236004	200356948	200357007	ATP2B4	0.60	-0.22	0.81
A_14_P130289	200364956	200365015	ATP2B4	0.27	0.82	0.85
A_16_P00236035	200374683	200374742	ATP2B4	0.35	0.84	0.83
A_14_P100473	200382149	200382208	ATP2B4	0.35	0.73	0.95
A_16_P35434337	200390401	200390460	ATP2B4	0.89	1.35	1.20
A_16_P15416774	200400056	200400115	ATP2B4	0.98	1.13	0.87
A_14_P138032	200407816	200407875	ATP2B4	0.27	0.47	0.80
A_16_P15416827	200419668	200419727	ATP2B4	1.31	1.28	0.96
A_16_P15416841	200425392	200425451	ATP2B4	0.51	0.61	0.99
A_14_P134210	200431768	200431821	ATP2B4	0.68	1.63	0.69
A_14_P115882	200434051	200434100	ATP2B4	1.52	1.55	1.15
A_16_P35434506	200442490	200442549	ATP2B4	0.85	1.01	0.82
A_14_P125773	200451559	200451618	chr1:200451559-200451618	0.97	1.25	0.91
A_16_P15416932	200456516	200456570	chr1:200456516-200456570	0.90	1.47	0.88
A_16_P00236180	200461236	200461295	chr1:200461236-200461295	0.52	0.92	0.74
A_14_P123051	200466184	200466231	LAX1	0.81	1.41	0.99
A_14_P108847	200475751	200475810	LAX1	1.15	1.08	1.07
A_16_P00236201	200486482	200486541	chr1:200486482-200486541	1.14	1.51	0.89
A_16_P15416991	200495245	200495304	chr1:200495245-200495304	0.77	0.77	0.93
A_14_P109305	200500677	200500736	ZC3H11A	0.53	1.11	0.82
A_16_P15417031	200507490	200507549	ZC3H11A	0.34	1.01	0.83
A_14_P131701	200517648	200517707	ZC3H11A	0.54	0.99	0.75
A_16_P00236243	200523398	200523457	ZC3H11A	0.86	1.22	0.71
A_16_P35434714	200532542	200532601	ZC3H11A	0.82	0.76	1.00
A_16_P15417096	200538693	200538752	ZC3H11A	0.71	1.05	0.61
A_16_P15417105	200543897	200543956	ZC3H11A	0.65	1.17	0.85
A_14_P112115	200552021	200552080	ZC3H11A	0.95	1.39	0.92
A_16_P35434798	200557220	200557279	chr1:200557220-200557279	1.17	1.61	1.02
A_16_P00236292	200561749	200561808	chr1:200561749-200561808	0.89	0.93	0.88
A_14_P124920	200569199	200569258	SNRPE	1.15	0.93	0.88
A_16_P35434855	200581689	200581748	chr1:200581689-200581748	0.84	1.22	0.95
A_16_P00236322	200589862	200589921	chr1:200589862-200589921	0.11	0.99	0.71

A_16_P15417218	200599204	200599263	chr1:200599204-200599263	0.68	0.86	0.95
A_16_P00236334	200610072	200610131	chr1:200610072-200610131	0.25	0.77	0.89
A_16_P35434903	200624073	200624132	chr1:200624073-200624132	0.53	1.10	0.91
A_16_P00236358	200637253	200637312	chr1:200637253-200637312	1.16	0.97	0.87
A_16_P15417310	200660803	200660862	chr1:200660803-200660862	0.65	1.68	0.76
A_16_P00236389	200686381	200686440	chr1:200686381-200686440	0.51	-0.02	0.82
A_16_P35435018	200704316	200704375	chr1:200704316-200704375	1.08	0.93	0.56
A_16_P35435036	200718721	200718780	chr1:200718721-200718780	0.45	0.72	0.70
A_14_P201653	200734101	200734160	C1orf157	0.65	1.58	0.83
A_14_P103801	200737992	200738051	C1orf157	1.03	0.95	0.67
A_16_P00236454	200747062	200747121	chr1:200747062-200747121	0.37	1.23	0.90
A_16_P15417452	200757448	200757507	chr1:200757448-200757507	0.31	-0.53	0.59
A_16_P00236463	200764333	200764392	chr1:200764333-200764392	0.15	0.15	0.81
A_16_P00236486	200782021	200782080	SOX13	0.24	1.94	1.04
A_14_P107182	200792135	200792194	SOX13	0.22	0.89	0.95
A_16_P00236516	200799345	200799404	SOX13	0.70	0.69	0.87
A_14_P107029	200812629	200812688	SOX13	0.76	0.41	0.88
A_14_P125431	200823412	200823471	SOX13	-0.30	0.79	0.76
A_16_P15417644	200829087	200829146	chr1:200829087-200829146	1.03	0.20	0.87
A_16_P00236579	200833550	200833609	ETNK2	0.84	0.43	0.80
A_16_P00236592	200842095	200842154	ETNK2	0.68	0.58	0.98
A_16_P00236601	200845797	200845856	ETNK2	0.86	1.04	0.84
A_14_P120854	200851511	200851564	ETNK2	1.55	1.09	1.13
A_16_P15417739	200860056	200860115	REN	0.65	0.55	0.90
A_14_P101388	200864080	200864139	REN	0.80	1.48	0.77
A_14_P104722	200873587	200873638	chr1:200873587-200873638	1.23	0.68	0.64
A_16_P00236659	200880641	200880700	chr1:200880641-200880700	1.23	0.32	1.20
A_16_P35435467	200884134	200884193	chr1:200884134-200884193	0.79	1.10	0.91
A_16_P00236669	200889449	200889503	chr1:200889449-200889503	1.06	1.25	0.89
A_14_P115566	200893182	200893241	KISS1	0.48	1.18	0.90
A_14_P119774	200895173	200895229	KISS1	1.07	0.88	1.12
A_14_P117623	200896856	200896912	KISS1	1.19	1.16	0.95
A_16_P00236692	200901429	200901488	GOLT1A	1.19	0.54	1.05
A_16_P35435541	200907569	200907628	GOLT1A	1.30	1.14	0.97
A_16_P00236706	200912360	200912419	GOLT1A	1.08	1.36	1.02
A_14_P137927	200919873	200919932	PLEKHA6	0.26	1.89	0.51
A_16_P15417900	200926062	200926121	PLEKHA6	0.60	0.75	0.96
A_16_P15417914	200930982	200931041	PLEKHA6	0.24	0.44	0.75
A_16_P00236750	200944463	200944519	PLEKHA6	1.31	0.66	0.83
A_16_P00236775	200956408	200956467	PLEKHA6	0.73	1.02	0.85
A_16_P15418035	200974526	200974581	PLEKHA6	1.44	0.58	0.99
A_16_P15418057	200980200	200980259	PLEKHA6	1.06	1.03	0.98
A_16_P15418092	200989917	200989976	PLEKHA6	0.88	0.54	1.08
A_16_P35435816	200997962	200998021	PLEKHA6	0.64	0.79	0.79
A_14_P132951	201011102	201011161	PLEKHA6	0.68	1.43	0.73
A_16_P00236913	201019395	201019454	PLEKHA6	0.68	1.36	1.08
A_16_P00236933	201029892	201029951	PLEKHA6	0.45	0.67	0.86
A_16_P00236946	201036666	201036725	PLEKHA6	1.18	0.86	1.05

A_16_P00236959	201043593	201043652	PLEKHA6	0.80	1.59	0.83
A_16_P00236972	201050760	201050819	PLEKHA6	0.99	1.41	1.03
A_16_P15418304	201061757	201061816	chr1:201061757-201061816	1.08	1.21	0.77
A_16_P00237013	201077622	201077681	chr1:201077622-201077681	1.07	0.66	1.20
A_16_P15418363	201083912	201083971	chr1:201083912-201083971	1.31	1.10	1.09
A_16_P00237028	201089292	201089351	chr1:201089292-201089351	0.73	1.54	0.98
A_16_P35436089	201098834	201098893	chr1:201098834-201098893	1.05	0.67	1.02
A_14_P126454	201106886	201106945	PPP1R15B	1.04	1.38	0.79
A_16_P00237058	201110709	201110768	PPP1R15B	1.08	0.94	0.98
A_16_P15418440	201114841	201114900	chr1:201114841-201114900	-6.24	0.73	1.06
A_14_P111843	201123810	201123869	PIK3C2B	0.98	0.87	0.98
A_16_P15418493	201136093	201136152	PIK3C2B	0.93	1.35	0.78
A_16_P15418510	201141742	201141801	PIK3C2B	1.08	1.61	1.04
A_14_P107166	201157571	201157630	PIK3C2B	0.92	0.55	0.61
A_16_P00237168	201169853	201169912	PIK3C2B	1.09	1.84	1.11
A_16_P15418631	201181733	201181792	PIK3C2B	0.99	1.05	1.05
A_16_P00237206	201190888	201190946	PIK3C2B	1.14	0.44	0.97
A_16_P00237221	201200238	201200297	chr1:201200238-201200297	1.22	1.02	0.96
A_16_P00237228	201205833	201205892	chr1:201205833-201205892	0.95	1.39	0.85
A_14_P124092	201217187	201217231	MDM4	1.05	1.30	0.86
A_16_P00237249	201226263	201226322	MDM4	0.87	0.93	0.83
A_16_P00237259	201232961	201233020	MDM4	0.60	1.18	0.84
A_14_P127624	201239051	201239110	MDM4	0.74	0.59	0.86
A_16_P00237292	201252599	201252658	CR749470	0.91	0.61	0.84
A_14_P108985	201267016	201267075	CR749470	0.12	0.96	0.52
A_16_P00237338	201279302	201279361	CR749470	0.46	0.95	0.66
A_16_P00237348	201284819	201284878	CR749470	0.77	1.22	0.91
A_16_P15418926	201293431	201293490	CR749470	0.54	1.31	0.93
A_16_P00237370	201303004	201303063	CR749470	1.55	0.95	1.08
A_14_P118079	201310532	201310591	CR749470	0.42	1.24	0.63
A_16_P00237390	201318569	201318627	LRRN5	1.34	1.10	1.08
A_14_P116727	201329549	201329606	LRRN5	0.19	-0.93	0.53
A_16_P00237424	201337049	201337108	LRRN5	1.28	0.89	0.70
A_16_P00237450	201350731	201350790	LRRN5	0.81	-0.24	0.80
A_16_P35436857	201360598	201360657	LRRN5	0.52	0.95	0.85
A_16_P00237477	201367046	201367105	LRRN5	0.83	0.46	0.93
A_16_P00237503	201383602	201383658	LRRN5	-0.23	1.40	0.68
A_14_P123949	201393157	201393216	AF007111	0.82	1.36	0.74
A_16_P15419219	201402236	201402295	AF007111	1.12	1.16	1.21
A_16_P15419258	201414170	201414229	chr1:201414170-201414229	0.98	0.79	0.89
A_16_P00237580	201429296	201429355	chr1:201429296-201429355	-0.13	1.06	0.68
A_16_P35437155	201473561	201473620	chr1:201473561-201473620	0.98	1.57	1.04
A_14_P121731	201505681	201505740	chr1:201505681-201505740	0.54	0.83	0.92
A_16_P35437245	201519752	201519811	chr1:201519752-201519811	0.83	0.82	0.89
A_16_P00237708	201533530	201533589	NFASC	1.37	1.26	1.04
A_16_P00237724	201539904	201539963	NFASC	1.14	1.29	0.97
A_16_P00237743	201549711	201549770	NFASC	0.79	1.20	0.70
A_16_P00237753	201555949	201556008	NFASC	0.89	1.44	1.02

A_16_P00237767	201567574	201567633	NFASC	0.96	1.06	0.85
A_16_P00237785	201576770	201576829	NFASC	0.92	1.09	0.96
A_16_P00237804	201587655	201587714	NFASC	-0.61	1.35	0.86
A_16_P00237818	201595856	201595915	NFASC	0.74	1.35	0.95
A_16_P00237833	201605422	201605481	NFASC	0.23	0.31	0.65
A_16_P15419740	201621273	201621332	NFASC	0.41	1.19	0.81
A_16_P15419776	201632922	201632981	NFASC	0.80	0.18	1.06
A_16_P00237876	201639441	201639500	NFASC	1.59	1.16	1.00
A_16_P00237887	201644945	201645004	NFASC	0.88	0.83	0.87
A_16_P00237906	201654529	201654588	NFASC	0.97	1.17	1.04
A_16_P00237919	201661615	201661674	NFASC	0.91	1.25	1.00
A_16_P00237933	201667366	201667425	NFASC	1.04	1.19	0.95
A_16_P00237958	201678403	201678457	NFASC	0.65	0.68	0.83
A_16_P00237973	201684985	201685044	NFASC	0.50	0.67	0.84
A_16_P00237988	201691114	201691173	NFASC	1.02	0.72	1.02
A_14_P138873	201704018	201704077	NFASC	0.40	0.61	0.89
A_16_P00238029	201710754	201710811	NFASC	0.63	0.96	0.77
A_16_P15420045	201717807	201717866	NFASC	1.14	0.60	0.96
A_16_P00238074	201731296	201731355	chr1:201731296-201731355	0.72	0.18	0.85
A_16_P15420104	201737486	201737530	chr1:201737486-201737530	0.66	-0.36	0.60
A_16_P15420120	201743208	201743265	chr1:201743208-201743265	0.44	1.12	0.92
A_14_P110453	201754154	201754213	CNTN2	0.61	-1.34	0.92
A_16_P00238132	201761531	201761590	CNTN2	0.79	1.37	0.74
A_16_P15420202	201769542	201769600	CNTN2	-0.27	1.70	0.64
A_16_P15420232	201777681	201777740	CNTN2	0.80	0.63	1.03
A_16_P15420242	201783601	201783655	chr1:201783601-201783655	1.19	1.08	1.17
A_14_P117858	201787922	201787981	RBBP5	0.99	0.61	0.87
A_16_P15420278	201794699	201794758	RBBP5	0.61	0.80	0.88
A_16_P00238208	201800913	201800972	RBBP5	0.75	1.33	0.70
A_16_P00238214	201805803	201805862	RBBP5	0.69	1.65	0.95
A_16_P15420320	201811962	201812021	RBBP5	0.38	0.71	0.83
A_14_P110699	201820856	201820915	RBBP5	0.45	0.93	0.64
A_16_P15420360	201827697	201827756	chr1:201827697-201827756	0.22	0.42	0.77
A_16_P00238252	201843268	201843327	RIPK5	1.09	1.47	1.00
A_16_P35438264	201858837	201858896	RIPK5	0.14	0.42	0.61
A_16_P00238292	201864491	201864550	RIPK5	1.02	1.16	0.79
A_16_P00238298	201869969	201870028	RIPK5	0.90	1.12	0.96
A_16_P15420483	201879023	201879082	RIPK5	0.49	1.02	0.81
A_14_P115808	201890869	201890928	RIPK5	0.74	0.76	0.82
A_16_P00238346	201900567	201900626	RIPK5	0.41	0.43	0.84
A_16_P15420567	201910522	201910581	RIPK5	1.05	1.50	1.00
A_16_P00238368	201919095	201919154	chr1:201919095-201919154	0.63	0.67	0.77
A_16_P35438442	201925238	201925297	chr1:201925238-201925297	0.81	0.57	0.79
A_16_P00238398	201934203	201934262	TMCC2	1.09	1.60	0.82
A_16_P15420660	201943040	201943099	TMCC2	0.98	1.82	0.99
A_14_P119770	201949237	201949296	TMCC2	1.23	1.24	1.09
A_16_P35438537	201956522	201956581	TMCC2	0.93	0.20	0.85
A_14_P125724	201966170	201966229	TMCC2	0.34	0.96	0.71

A_16_P15420745	201974094	201974153	TMCC2	0.81	1.19	0.84
A_16_P00238484	201982481	201982540	chr1:201982481-201982540	0.70	0.79	0.92
A_16_P00238494	201988931	201988990	chr1:201988931-201988990	0.43	2.06	0.49
A_16_P00238501	201995465	201995522	chr1:201995465-201995522	1.05	0.70	0.97
A_16_P35438659	202003036	202003095	NUAK2	0.57	1.05	0.84
A_14_P107350	202008019	202008078	NUAK2	1.25	1.27	1.07
A_16_P00238534	202015643	202015700	NUAK2	1.18	1.69	1.05
A_16_P00238542	202019539	202019598	NUAK2	1.00	1.35	0.92
A_16_P00238556	202028160	202028218	chr1:202028160-202028218	0.66	1.04	1.30
A_16_P00238572	202037353	202037411	FLJ10748	1.01	0.80	0.95
A_14_P124157	202045508	202045567	FLJ10748	0.74	0.64	0.72
A_16_P00238609	202056237	202056296	FLJ10748	0.49	2.37	1.04
A_16_P00238617	202060500	202060559	chr1:202060500-202060559	0.89	0.53	0.99
A_16_P15421013	202069149	202069208	chr1:202069149-202069208	0.27	2.12	-1.67
A_16_P15421024	202074370	202074429	AK090497	0.83	0.99	0.85
A_16_P00238644	202082435	202082494	LEMD1	0.79	0.85	0.95
A_16_P15421059	202089626	202089685	LEMD1	0.58	1.40	0.81
A_16_P15421065	202097804	202097861	LEMD1	0.81	1.43	0.69
A_16_P15421088	202114983	202115042	LEMD1	0.98	1.57	1.01
A_14_P127276	202123440	202123499	AK026628	1.04	1.53	0.97
A_16_P00238698	202128651	202128710	AK026628	0.71	0.33	0.96
A_16_P00238705	202133282	202133340	AK026628	1.08	1.50	0.98
A_16_P00238712	202136423	202136482	AK026628	0.56	0.07	0.81
A_16_P15421167	202139956	202140008	AK026628	1.12	0.80	0.95
A_16_P00238729	202144103	202144162	AK026628	0.60	0.54	0.58
A_16_P15421191	202146719	202146778	AK026628	1.15	0.85	1.00
A_16_P00238751	202153599	202153658	chr1:202153599-202153658	0.82	0.97	0.89
A_16_P15421227	202156995	202157054	AK095633	1.40	1.43	1.12
A_16_P15421237	202160431	202160489	AK095633	1.08	0.52	0.90
A_16_P35439131	202171381	202171440	chr1:202171381-202171440	0.19	0.91	0.68
A_16_P00238780	202177340	202177399	chr1:202177340-202177399	1.12	0.79	0.95
A_16_P00238795	202184466	202184525	chr1:202184466-202184525	0.96	0.91	1.02
A_16_P15421332	202200281	202200340	chr1:202200281-202200340	0.25	0.54	0.92
A_16_P00238841	202214252	202214311	PCTK3	0.77	0.79	1.04
A_16_P00238853	202222542	202222600	PCTK3	1.52	0.69	0.97
A_16_P15421415	202229082	202229141	PCTK3	0.71	1.00	0.67
A_16_P00238892	202242015	202242074	chr1:202242015-202242074	0.45	0.87	1.06
A_16_P00238900	202247607	202247666	chr1:202247607-202247666	1.14	-0.10	1.03
A_16_P15421485	202254008	202254067	chr1:202254008-202254067	0.75	0.11	0.87
A_14_P201827	202271354	202271412	MFSD4	0.20	0.35	0.83
A_14_P114267	202280259	202280318	MFSD4	0.95	1.37	0.79
A_16_P15421560	202286238	202286297	MFSD4	0.67	0.96	0.79
A_16_P00238963	202294390	202294449	MFSD4	0.86	1.44	0.96
A_14_P136612	202303674	202303733	BC040145	0.05	0.44	0.76
A_16_P15421636	202310306	202310365	BC040145	0.61	1.10	0.83
A_14_P127696	202317162	202317221	ELK4	0.94	1.60	0.93
A_14_P127572	202320577	202320636	ELK4	0.47	0.74	0.83
A_16_P15421687	202325754	202325813	ELK4	0.92	1.43	0.90

A_14_P136625	202331236	202331295	ELK4	0.50	1.44	0.91
A_14_P136625	202331236	202331295	ELK4	0.46	1.39	0.86
A_14_P136625	202331236	202331295	ELK4	0.54	1.59	0.92
A_16_P35439635	202338578	202338637	chr1:202338578-202338637	1.06	1.68	0.63
A_16_P00239049	202346626	202346685	chr1:202346626-202346685	0.63	1.18	1.05
A_16_P00239059	202358631	202358690	SLC45A3	0.94	1.13	0.65
A_16_P00239077	202366885	202366944	SLC45A3	0.86	0.84	1.02
A_14_P118252	202373737	202373796	SLC45A3	0.99	1.17	0.77
A_16_P00239103	202382325	202382384	chr1:202382325-202382384	1.16	1.14	1.03
A_16_P00239110	202389333	202389392	chr1:202389333-202389392	1.17	0.60	0.97
A_16_P15421869	202397699	202397758	chr1:202397699-202397758	1.14	0.34	1.01
A_16_P35439822	202409434	202409493	chr1:202409434-202409493	1.10	1.49	1.50
A_14_P139395	202424578	202424628	NUCKS1	0.53	1.14	0.78
A_16_P35439903	202430378	202430437	NUCKS1	0.52	0.64	0.92
A_14_P133811	202447092	202447151	NUCKS1	1.02	1.59	1.05
A_16_P15422049	202455628	202455687	chr1:202455628-202455687	0.10	0.71	0.87
A_16_P00239232	202463607	202463666	chr1:202463607-202463666	0.98	0.38	0.79
A_14_P118188	202470788	202470847	RAB7L1	0.75	1.55	0.91
A_16_P00239262	202479414	202479473	chr1:202479414-202479473	0.80	0.47	0.94
A_16_P00239269	202485608	202485667	chr1:202485608-202485667	0.92	0.17	0.96
A_16_P15422133	202489972	202490031	SLC41A1	0.23	0.81	0.69
A_16_P35440098	202498533	202498592	SLC41A1	1.12	1.15	0.91
A_16_P00239301	202502860	202502916	SLC41A1	1.08	0.97	1.01
A_14_P115088	202508339	202508398	SLC41A1	1.32	1.03	1.07
A_16_P00239317	202513009	202513068	SLC41A1	0.97	1.34	0.97
A_16_P15422221	202518126	202518185	chr1:202518126-202518185	0.96	-0.05	1.02
A_16_P15422228	202522885	202522944	chr1:202522885-202522944	0.94	1.08	0.92
A_16_P15422228	202522885	202522944	chr1:202522885-202522944	0.93	0.94	0.82
A_16_P15422228	202522885	202522944	chr1:202522885-202522944	1.00	0.66	0.96
A_14_P137731	202528806	202528865	FLJ32569	0.49	1.10	1.01
A_16_P35440208	202535380	202535439	FLJ32569	1.08	1.68	1.23
A_16_P35440228	202542652	202542711	FLJ32569	0.34	0.79	0.96
A_14_P127966	202549082	202549141	FLJ32569	0.23	0.39	0.34
A_16_P00239377	202554518	202554577	chr1:202554518-202554577	0.69	0.74	0.84
A_16_P15422324	202599960	202600019	chr1:202599960-202600019	0.10	1.15	0.67
A_16_P15422326	202608438	202608492	chr1:202608438-202608492	0.42	0.81	0.48
A_16_P15422333	202613935	202613994	SLC26A9	0.76	0.86	0.84
A_16_P00239406	202624316	202624375	SLC26A9	0.27	1.00	0.79
A_14_P111373	202633937	202633996	SLC26A9	0.47	0.58	0.81
A_16_P00239441	202640563	202640620	SLC26A9	1.04	1.15	1.13
A_14_P200819	202645984	202646041	chr1:202645984-202646041	1.06	1.13	0.98
A_16_P00239470	202653435	202653494	chr1:202653435-202653494	1.08	1.54	1.01
A_16_P00239473	202732905	202732964	chr1:202732905-202732964	0.46	1.13	0.74
A_16_P00239495	202745047	202745106	chr1:202745047-202745106	0.55	1.49	0.80
A_16_P15422606	202756620	202756679	chr1:202756620-202756679	0.52	0.95	0.86
A_16_P00239519	202765185	202765244	chr1:202765185-202765244	0.93	1.12	1.09
A_16_P00239541	202776694	202776753	chr1:202776694-202776753	0.90	1.01	0.92
A_16_P15422671	202782397	202782456	C1orf186	1.12	1.13	1.00

A_16_P15422685	202786791	202786850	C1orf186	0.95	1.53	0.92
A_16_P15422713	202796621	202796680	C1orf186	0.72	0.88	0.92
A_16_P15422731	202802693	202802752	C1orf186	0.08	0.78	0.77
A_16_P15422744	202810170	202810229	C1orf186	0.84	1.40	0.79
A_16_P15422774	202823062	202823121	C1orf186	1.23	0.70	0.87
A_16_P00239622	202836108	202836167	chr1:202836108-202836167	0.44	0.90	0.90
A_16_P15422810	202846888	202846947	chr1:202846888-202846947	0.57	0.27	0.67
A_16_P35440798	202852155	202852214	chr1:202852155-202852214	0.71	0.76	0.96
A_16_P35440809	202858813	202858872	chr1:202858813-202858872	0.68	1.27	0.91
A_16_P15422853	202864766	202864825	CTSE	0.22	0.63	0.48
A_16_P15422857	202868692	202868751	CTSE	0.05	1.69	0.80
A_16_P00239675	202874290	202874349	CTSE	0.45	0.77	0.66
A_16_P35440872	202928411	202928465	chr1:202928411-202928465	1.47	1.85	0.83
A_16_P00239679	202959653	202959712	SRGAP2	0.73	1.81	0.93
A_16_P15423014	202972092	202972151	SRGAP2	0.58	-0.56	0.61
A_14_P116938	202982019	202982078	SRGAP2	0.50	0.09	0.72
A_16_P15423081	202996571	202996630	SRGAP2	1.06	1.03	1.04
A_16_P00239741	203005995	203006054	SRGAP2	0.51	0.97	0.87
A_16_P00239769	203018181	203018240	SRGAP2	0.60	0.82	0.99
A_16_P00239784	203025149	203025208	SRGAP2	0.05	-0.07	0.84
A_16_P00239804	203034077	203034136	IKBKE	0.46	1.74	0.64
A_14_P131795	203040278	203040337	IKBKE	0.37	1.00	0.33
A_16_P00239830	203046065	203046124	IKBKE	0.88	0.36	0.87
A_16_P15423268	203051603	203051662	IKBKE	0.91	0.39	1.12
A_16_P15423297	203059734	203059793	chr1:203059734-203059793	0.84	0.59	0.90
A_16_P00239865	203064508	203064567	chr1:203064508-203064567	1.18	1.06	1.04
A_16_P15423332	203071535	203071594	RASSF5	1.03	1.08	0.91
A_14_P132661	203077571	203077630	RASSF5	0.58	0.72	0.66
A_16_P00239906	203086751	203086803	RASSF5	0.92	1.58	0.89
A_16_P00239919	203094267	203094326	RASSF5	1.07	1.13	1.12
A_16_P00239935	203103385	203103444	RASSF5	0.87	1.03	0.95
A_14_P111221	203119359	203119416	RASSF5	0.66	0.74	0.98
A_16_P35441493	203125438	203125497	RASSF5	0.82	1.16	0.84
A_16_P00239989	203136345	203136404	RASSF5	0.87	1.46	0.85
A_16_P00239998	203141155	203141214	RASSF5	0.72	0.08	0.87
A_16_P35441575	203149521	203149580	RASSF5	0.72	1.28	1.03
A_16_P35441608	203157563	203157622	LGTN	0.66	0.14	0.93
A_14_P113946	203163843	203163902	LGTN	0.75	1.10	0.93
A_16_P15423620	203171033	203171092	LGTN	0.88	1.28	0.90
A_16_P15423633	203175617	203175676	chr1:203175617-203175676	0.57	0.84	0.79
A_16_P15423642	203182867	203182926	chr1:203182867-203182926	0.98	1.81	1.17
A_16_P15423652	203190083	203190142	chr1:203190083-203190142	0.68	1.43	0.65
A_16_P00240086	203194466	203194525	chr1:203194466-203194525	0.39	0.58	1.07
A_16_P00240095	203198626	203198685	DYRK3	0.54	0.66	0.88
A_16_P15423693	203202753	203202812	DYRK3	0.94	1.79	1.01
A_14_P109525	203210762	203210821	DYRK3	0.47	1.62	0.85
A_16_P35441766	203215076	203215135	BC037269	0.60	-0.19	0.88
A_16_P00240146	203225083	203225142	BC037269	0.33	0.31	0.83

A_16_P15423781	203231761	203231820	BC037269	0.83	1.05	0.98
A_16_P35441842	203240156	203240215	BC037269	0.81	0.89	1.03
A_14_P100433	203249430	203249489	MAPKAPK2	0.95	0.75	1.02
A_16_P00240209	203257426	203257485	MAPKAPK2	1.02	1.47	0.94
A_16_P00240231	203266794	203266853	MAPKAPK2	0.80	1.24	0.68
A_16_P35441951	203272906	203272965	MAPKAPK2	0.55	1.46	0.70
A_16_P15423925	203280934	203280993	MAPKAPK2	0.59	0.72	0.98
A_14_P103877	203286569	203286628	MAPKAPK2	0.85	0.71	1.04
A_14_P129763	203294484	203294543	MAPKAPK2	0.63	1.35	0.78
A_16_P35442053	203308038	203308097	chr1:203308038-203308097	0.58	0.91	0.79
A_16_P00240296	203312799	203312858	chr1:203312799-203312858	0.47	0.80	0.84
A_16_P00240301	203322138	203322197	chr1:203322138-203322197	1.16	1.18	0.96
A_14_P116893	203329383	203329442	IL10	0.40	0.28	0.80
A_14_P130790	203330388	203330447	IL10	0.66	1.11	0.93
A_14_P128324	203333990	203334041	IL10	1.21	0.96	1.02
A_16_P00240345	203344634	203344693	chr1:203344634-203344693	0.91	1.24	1.07
A_16_P15424126	203354117	203354176	chr1:203354117-203354176	0.63	0.40	0.96
A_14_P138822	203359253	203359312	chr1:203359253-203359312	0.79	1.60	0.36
A_16_P00240389	203366096	203366155	IL19	0.68	1.13	0.98
A_16_P35442224	203371501	203371560	IL19	0.95	1.41	0.82
A_14_P103017	203376467	203376526	IL19	0.74	1.30	0.80
A_16_P00240398	203382070	203382129	IL19	1.06	0.28	0.97
A_16_P00240410	203388424	203388483	IL19	1.00	1.81	0.47
A_14_P126830	203395813	203395872	IL19	0.10	1.24	0.49
A_16_P00240439	203402113	203402172	IL19	0.73	1.48	0.98
A_16_P00240449	203407187	203407246	chr1:203407187-203407246	0.76	1.05	0.72
A_16_P15424280	203419075	203419134	chr1:203419075-203419134	0.92	1.62	0.91
A_14_P109657	203428036	203428091	IL20	1.09	1.12	0.82
A_16_P15424327	203435339	203435398	chr1:203435339-203435398	0.53	1.39	0.89
A_16_P00240491	203446486	203446544	chr1:203446486-203446544	1.04	1.17	0.87
A_16_P00240503	203454297	203454356	chr1:203454297-203454356	0.32	1.47	0.71
A_14_P105163	203459164	203459215	IL24	0.96	0.64	0.82
A_14_P200050	203461732	203461791	IL24	1.02	0.47	0.82
A_16_P00240524	203465750	203465809	IL24	0.45	0.78	0.93
A_14_P131026	203474696	203474746	FAIM3	1.27	1.25	0.82
A_16_P35442518	203479053	203479112	FAIM3	0.28	2.40	0.66
A_16_P35442523	203482758	203482817	FAIM3	0.48	1.35	0.94
A_16_P15424460	203488055	203488114	chr1:203488055-203488114	0.66	0.48	0.89
A_16_P00240568	203492557	203492616	PIGR	0.94	0.66	0.98
A_14_P105678	203495902	203495961	PIGR	1.03	0.65	0.95
A_16_P15424519	203506505	203506564	PIGR	0.57	0.49	1.08
A_16_P35442619	203509744	203509803	chr1:203509744-203509803	1.09	1.52	0.96
A_16_P00240618	203515356	203515415	chr1:203515356-203515415	0.18	0.15	0.85
A_16_P00240627	203518918	203518977	chr1:203518918-203518977	0.31	1.30	0.62
A_16_P00240638	203523953	203524008	FCAMR	1.08	1.32	0.88
A_14_P103597	203527991	203528050	FCAMR	0.92	1.47	0.55
A_16_P00240658	203532522	203532581	chr1:203532522-203532581	0.79	0.54	0.94
A_16_P15424621	203539204	203539263	chr1:203539204-203539263	0.81	0.94	0.71

A_16_P00240678	203548521	203548580	chr1:203548521-203548580	0.25	0.60	0.75
A_16_P35442755	203557938	203557997	chr1:203557938-203557997	1.00	0.75	0.93
A_16_P00240698	203569276	203569335	chr1:203569276-203569335	0.47	0.83	0.81
A_16_P35442798	203576418	203576477	chr1:203576418-203576477	0.46	1.19	0.87
A_14_P114002	203582015	203582068	C1orf116	0.52	1.17	0.71
A_16_P35442845	203588521	203588580	C1orf116	0.43	0.09	0.82
A_16_P00240746	203592928	203592987	C1orf116	1.47	0.50	0.71
A_16_P15424772	203596263	203596322	chr1:203596263-203596322	0.71	1.32	0.82
A_16_P15424780	203601256	203601315	chr1:203601256-203601315	0.57	1.51	0.91
A_16_P15424789	203605560	203605619	YOD1	0.45	1.01	0.39
A_16_P00240777	203611092	203611151	YOD1	0.52	1.29	0.64
A_16_P35442950	203619371	203619430	PFKFB2	0.53	1.14	0.63
A_16_P00240810	203625124	203625181	PFKFB2	0.82	1.02	1.03
A_14_P105616	203629913	203629967	PFKFB2	0.61	1.12	0.81
A_16_P00240830	203634227	203634286	PFKFB2	0.11	-1.01	0.80
A_14_P127529	203642082	203642141	PFKFB2	-0.52	1.20	0.76
A_16_P35443064	203646994	203647053	chr1:203646994-203647053	1.02	0.76	0.86
A_14_P107701	203650624	203650668	C4BPB	0.25	1.85	0.47
A_14_P118745	203650954	203651008	C4BPB	0.08	1.32	0.79
A_14_P103462	203651084	203651143	C4BPB	-0.02	1.13	0.68
A_16_P00240879	203658310	203658369	C4BPB	0.89	0.77	0.81
A_14_P121791	203666102	203666161	C4BPA	1.22	1.31	0.99
A_16_P15425006	203671520	203671579	C4BPA	0.73	1.46	0.86
A_16_P00240916	203677173	203677232	C4BPA	0.31	0.88	0.95
A_16_P15425030	203682445	203682504	C4BPA	0.39	1.29	0.84
A_14_P117643	203688471	203688526	C4BPA	0.95	1.58	0.68
A_16_P15425060	203696314	203696373	C4BPA	0.96	1.62	0.91
A_14_P134479	203706623	203706682	C4BPA	-0.04	0.70	0.52
A_16_P15425105	203719061	203719120	chr1:203719061-203719120	0.50	1.35	0.81
A_16_P15425128	203728657	203728716	chr1:203728657-203728716	0.54	1.31	0.76
A_14_P111708	203740433	203740492	chr1:203740433-203740492	0.98	1.42	0.90
A_16_P15425234	203783862	203783921	chr1:203783862-203783921	0.19	0.87	0.70
A_16_P15425273	203804010	203804069	chr1:203804010-203804069	0.54	1.86	0.68
A_16_P15425326	203833303	203833362	chr1:203833303-203833362	0.68	1.16	1.20
A_16_P35443511	203850093	203850152	chr1:203850093-203850152	0.85	1.87	1.07
A_16_P00241138	203873165	203873224	chr1:203873165-203873224	0.82	1.37	0.94
A_14_P201810	203886409	203886468	CD55	0.49	1.84	0.89
A_16_P00241174	203892920	203892979	CD55	0.74	1.48	1.00
A_14_P119189	203898434	203898488	CD55	0.77	0.27	0.71
A_14_P129167	203898896	203898945	CD55	0.62	0.97	0.44
A_14_P136375	203899311	203899370	CD55	0.22	1.37	0.77
A_16_P15425494	203915568	203915627	CD55	0.88	1.14	0.94
A_16_P35443696	203921571	203921630	CD55	0.63	1.34	0.77
A_14_P137203	203934702	203934761	chr1:203934702-203934761	0.12	1.04	0.57
A_16_P00241245	203953117	203953176	chr1:203953117-203953176	0.43	0.89	0.91
A_16_P35443802	203967528	203967587	chr1:203967528-203967587	0.94	1.03	0.95
A_14_P118290	203979657	203979716	chr1:203979657-203979716	0.21	1.29	0.78
A_16_P15425657	203992260	203992319	chr1:203992260-203992319	0.87	1.63	0.76

A_16_P00241309	204006633	204006692	chr1:204006633-204006692	0.96	0.74	1.04
A_14_P126654	204015795	204015854	chr1:204015795-204015854	0.60	-0.12	1.08
A_16_P35443923	204023403	204023462	CR2	1.07	-0.34	1.15
A_16_P15425766	204032688	204032747	CR2	0.31	0.41	0.87
A_16_P35443999	204041716	204041775	CR2	0.79	1.56	0.70
A_14_P133087	204050784	204050843	CR2	0.50	0.60	0.93
A_16_P00241380	204056257	204056316	chr1:204056257-204056316	0.34	0.62	0.66
A_16_P00241391	204064776	204064834	CR1	0.88	0.96	0.86
A_16_P00241398	204071558	204071617	CR1	0.18	1.16	0.67
A_14_P116939	204084389	204084448	CR1	0.78	1.06	0.36
A_16_P15425881	204085282	204085341	CR1	0.49	0.31	0.72
A_16_P00241411	204128081	204128140	CR1	0.92	0.23	0.61
A_16_P15426042	204145267	204145326	CR1	0.17	0.04	0.67
A_16_P15426062	204156441	204156497	CR1	0.96	1.49	0.88
A_14_P104682	204168964	204169023	CR1	0.73	1.57	0.83
A_16_P35444362	204179961	204180020	CR1	0.70	1.32	0.92
A_14_P139964	204192238	204192297	CR1	0.21	1.31	0.64
A_16_P15426190	204202407	204202466	CR1	0.14	0.85	0.48
A_16_P15426224	204212141	204212200	BC109190	0.57	2.23	1.05
A_16_P00241544	204228620	204228679	BC109190	0.17	1.11	0.49
A_16_P35444514	204236242	204236301	BC109190	0.10	-0.20	0.49
A_14_P106089	204245435	204245494	BC109190	0.73	1.28	0.92
A_16_P35444550	204255421	204255480	BC109190	0.35	1.07	0.57
A_16_P00241579	204268180	204268239	BC109190	0.79	1.10	0.89
A_16_P35444603	204274990	204275049	BC109190	-0.03	0.97	0.73
A_16_P15426376	204281795	204281854	AY114160	0.51	0.79	0.76
A_16_P35444651	204292040	204292099	chr1:204292040-204292099	0.11	1.34	0.68
A_16_P35444654	204298431	204298490	chr1:204298431-204298490	-0.09	0.97	0.61
A_16_P00241620	204311956	204312015	chr1:204311956-204312015	0.55	-0.10	-0.40
A_16_P00241640	204322239	204322298	CD46	0.25	1.00	-0.73
A_16_P00241647	204328811	204328870	CD46	0.57	1.08	-0.93
A_14_P132063	204341536	204341595	CD46	0.54	1.16	-0.54
A_16_P35444749	204346840	204346899	CD46	0.27	-0.03	-1.24
A_16_P15426516	204352294	204352353	CD46	-0.08	1.45	-0.85
A_16_P15426531	204356257	204356316	CD46	0.36	0.71	-0.91
A_16_P00241689	204359744	204359803	chr1:204359744-204359803	0.79	1.62	-0.75
A_16_P15426561	204364919	204364978	AK123264	0.21	0.10	-0.95
A_16_P00241704	204367189	204367248	chr1:204367189-204367248	0.89	0.85	-0.48
A_16_P00241712	204370370	204370429	chr1:204370370-204370429	0.47	0.08	-1.10
A_16_P00241722	204374870	204374929	chr1:204374870-204374929	1.02	1.65	-0.75
A_16_P15426613	204378931	204378990	AK092969	1.09	0.66	-0.87
A_16_P00241743	204383231	204383290	LOC148696	1.16	0.50	-0.84
A_14_P104071	204389659	204389718	chr1:204389659-204389718	0.73	1.39	-0.53
A_16_P15426702	204404977	204405036	chr1:204404977-204405036	-0.04	1.15	-0.73
A_16_P00241811	204418397	204418456	chr1:204418397-204418456	0.72	0.72	-0.82
A_16_P00241844	204438278	204438337	chr1:204438278-204438337	0.10	0.27	-1.20
A_14_P108567	204452006	204452065	CD34	0.75	0.71	-0.93
A_16_P00241878	204460902	204460961	CD34	0.87	1.47	-0.78

A_16_P00241887	204465357	204465416	CD34	0.87	-0.23	-1.30
A_14_P125046	204472923	204472972	CD34	0.03	0.58	-0.43
A_16_P15426927	204486728	204486787	chr1:204486728-204486787	0.22	1.52	-0.55
A_16_P15426947	204503761	204503820	chr1:204503761-204503820	0.19	1.57	-0.11
A_16_P35445276	204524250	204524309	chr1:204524250-204524309	0.64	1.21	-1.07
A_16_P00242008	204548957	204549016	chr1:204548957-204549016	1.03	1.18	-0.82
A_16_P15427127	204571538	204571597	chr1:204571538-204571597	0.76	1.06	-0.86
A_14_P109074	204584768	204584825	AK125154	1.25	1.59	-0.68
A_14_P201303	204585980	204586038	AK125154	0.69	1.35	-0.82
A_16_P00242081	204591515	204591574	PLXNA2	0.58	0.51	-0.76
A_16_P15427225	204600873	204600926	PLXNA2	0.41	0.97	-0.78
A_16_P35445535	204607706	204607765	PLXNA2	1.21	1.06	-0.50
A_16_P00242153	204625201	204625260	PLXNA2	0.16	0.98	-0.87
A_16_P15427331	204633405	204633464	PLXNA2	-0.26	0.16	-0.81
A_16_P35445640	204640415	204640474	PLXNA2	0.83	1.95	-0.93
A_16_P35445690	204653087	204653146	PLXNA2	0.91	1.44	-0.78
A_16_P15427420	204659990	204660049	PLXNA2	0.17	-1.76	-1.39
A_16_P00242232	204665507	204665566	PLXNA2	0.31	0.98	-1.10
A_16_P15427462	204673497	204673556	PLXNA2	0.94	0.40	-0.77
A_16_P15427503	204685573	204685632	PLXNA2	-0.02	0.04	-1.04
A_16_P00242294	204695717	204695776	PLXNA2	0.91	0.34	-0.91
A_16_P00242308	204702859	204702918	PLXNA2	1.47	1.12	-0.80
A_16_P00242320	204709838	204709897	PLXNA2	0.56	1.44	-0.96
A_16_P15427615	204719201	204719260	PLXNA2	0.94	1.89	-0.98
A_16_P00242363	204727817	204727876	PLXNA2	0.81	1.08	-1.09
A_16_P00242386	204738168	204738227	PLXNA2	0.44	0.87	-0.93
A_16_P00242406	204747188	204747247	PLXNA2	0.31	1.15	-0.97
A_16_P35446059	204759947	204760006	PLXNA2	0.82	0.28	-0.76
A_16_P00242442	204765719	204765777	PLXNA2	0.57	0.45	-0.71
A_16_P15427789	204771778	204771837	PLXNA2	-0.40	1.08	-1.20
A_16_P00242468	204781336	204781395	PLXNA2	1.13	0.94	-0.77
A_14_P107008	204789214	204789273	PLXNA2	0.69	0.59	-0.81
A_16_P15427861	204795011	204795070	PLXNA2	0.44	0.61	-0.89
A_16_P15427899	204805487	204805538	AK074960	1.81	0.63	-0.56
A_16_P15427938	204816263	204816322	chr1:204816263-204816322	0.14	0.56	-1.29
A_16_P00242560	204829026	204829085	chr1:204829026-204829085	0.05	0.81	-1.15
A_16_P15428025	204846554	204846613	chr1:204846554-204846613	-0.41	0.75	-1.20
A_16_P15428116	204877552	204877611	chr1:204877552-204877611	0.72	0.46	-1.12
A_16_P15428222	204909708	204909767	chr1:204909708-204909767	0.11	1.00	-0.97
A_16_P00242761	204935996	204936055	chr1:204935996-204936055	0.47	0.66	-1.12
A_14_P100583	204973751	204973810	chr1:204973751-204973810	0.04	1.27	-0.40
A_16_P35446767	204996127	204996186	chr1:204996127-204996186	0.60	0.89	-1.01
A_16_P15428507	205020321	205020380	chr1:205020321-205020380	0.43	0.58	-1.11
A_16_P00242971	205070208	205070267	chr1:205070208-205070267	0.92	1.16	-1.29
A_16_P00243002	205095000	205095059	chr1:205095000-205095059	0.54	1.25	-1.14
A_16_P15428823	205145255	205145314	chr1:205145255-205145314	0.63	0.64	-1.01
A_16_P15428912	205174835	205174894	chr1:205174835-205174894	0.06	0.97	-1.51
A_16_P15428995	205209759	205209818	chr1:205209759-205209818	0.51	0.44	-0.78

A_16_P15429086	205241709	205241768	chr1:205241709-205241768	0.73	1.22	-1.05
A_16_P35447463	205277377	205277436	chr1:205277377-205277436	0.16	0.49	-1.21
A_14_P120556	205316497	205316556	chr1:205316497-205316556	0.15	0.43	-1.03
A_16_P15429371	205368930	205368989	chr1:205368930-205368989	0.75	1.46	-1.16
A_16_P15429434	205392955	205393014	chr1:205392955-205393014	0.59	1.31	-0.94
A_16_P00243505	205417127	205417186	chr1:205417127-205417186	0.99	0.63	-0.82
A_16_P15429560	205448113	205448172	chr1:205448113-205448172	0.27	0.38	-1.13
A_16_P15429627	205479257	205479316	chr1:205479257-205479316	1.05	1.11	-0.90
A_14_P127366	205507621	205507680	chr1:205507621-205507680	0.46	0.94	-1.05
A_16_P15429773	205538635	205538694	chr1:205538635-205538694	0.13	0.79	-1.13
A_16_P15429870	205592264	205592323	chr1:205592264-205592323	1.07	1.15	-1.20
A_14_P129960	205657066	205657125	chr1:205657066-205657125	0.21	1.17	-2.17
A_16_P15430138	205710878	205710937	chr1:205710878-205710937	0.66	0.95	-1.01
A_16_P15430229	205754022	205754081	chr1:205754022-205754081	0.47	1.33	-0.99
A_16_P15430347	205800420	205800479	chr1:205800420-205800479	0.66	0.89	-1.08
A_16_P00244064	205853861	205853920	chr1:205853861-205853920	0.49	1.08	-0.90
A_14_P106495	205885022	205885081	chr1:205885022-205885081	1.18	1.47	-0.87
A_16_P00244176	205913859	205913918	chr1:205913859-205913918	0.55	0.70	-0.92
A_16_P15430694	205930979	205931038	chr1:205930979-205931038	0.80	0.70	-1.08
A_16_P00244254	205954108	205954167	chr1:205954108-205954167	0.59	1.07	-1.11
A_16_P35449129	205966175	205966234	chr1:205966175-205966234	-0.24	3.20	-1.12
A_16_P00244290	205975915	205975974	chr1:205975915-205975974	0.71	0.61	-0.96
A_16_P15430851	205982151	205982210	chr1:205982151-205982210	0.97	1.08	-1.06
A_16_P35449196	205987565	205987624	chr1:205987565-205987624	0.92	1.04	-0.53
A_16_P00244324	205990580	205990639	AK091113	0.09	0.97	-0.21
A_16_P15430893	205994580	205994639	chr1:205994580-205994639	0.79	1.50	-1.11
A_16_P15430919	206002885	206002944	chr1:206002885-206002944	0.51	1.14	-0.66
A_16_P15430926	206005833	206005892	chr1:206005833-206005892	1.28	0.88	-0.96
A_16_P00244363	206019111	206019170	chr1:206019111-206019170	0.72	0.75	-1.11
A_14_P102283	206034300	206034359	chr1:206034300-206034359	0.10	0.54	-0.30
A_16_P15431042	206074039	206074098	chr1:206074039-206074098	0.18	1.29	-1.02
A_16_P35449452	206102064	206102123	chr1:206102064-206102123	0.63	0.73	-1.03
A_16_P15431159	206117014	206117073	chr1:206117014-206117073	1.51	1.57	-1.46
A_16_P15431195	206131117	206131176	chr1:206131117-206131176	0.75	1.31	-0.91
A_16_P00244533	206141826	206141885	chr1:206141826-206141885	0.66	1.23	-0.87
A_14_P120532	206148667	206148726	CAMK1G	1.00	1.37	-0.81
A_16_P15431272	206154999	206155058	CAMK1G	0.43	0.84	-0.99
A_16_P35449645	206167973	206168032	CAMK1G	0.97	1.02	-0.54
A_14_P109728	206173989	206174039	CAMK1G	0.24	2.00	-0.80
A_16_P15431364	206184485	206184535	LAMB3	0.58	1.12	-0.74
A_16_P15431397	206194774	206194833	LAMB3	1.27	1.11	-0.56
A_16_P00244656	206200649	206200708	LAMB3	0.47	1.38	-0.74
A_16_P00244669	206207048	206207107	LAMB3	0.44	1.07	-0.86
A_14_P125424	206212778	206212837	LAMB3	0.40	0.73	-1.01
A_16_P15431487	206225115	206225174	chr1:206225115-206225174	0.72	1.41	-0.67
A_16_P00244715	206232449	206232508	chr1:206232449-206232508	0.47	-0.25	-0.81
A_14_P127588	206237980	206238038	G0S2	0.92	1.32	-0.88
A_16_P15431529	206244124	206244183	chr1:206244124-206244183	0.52	0.96	-0.99

A_16_P35449904	206248605	206248664	HSD11B1	0.83	0.62	-0.69
A_16_P00244749	206254863	206254922	HSD11B1	1.18	1.23	-0.92
A_16_P15431564	206260416	206260475	HSD11B1	0.88	1.43	-0.82
A_14_P112241	206268708	206268766	HSD11B1	0.44	0.07	-0.44
A_16_P15431619	206285308	206285367	HSD11B1	0.74	0.85	-0.63
A_14_P121171	206296446	206296504	HSD11B1	0.47	1.35	-0.31
A_16_P00244812	206305345	206305404	chr1:206305345-206305404	1.30	0.95	-0.82
A_16_P15431687	206313791	206313850	chr1:206313791-206313850	0.69	0.34	-1.07
A_14_P118353	206324795	206324851	TRAF3IP3	0.33	1.32	-1.07
A_16_P15431732	206328740	206328799	TRAF3IP3	0.90	0.66	-0.65
A_14_P110893	206334874	206334933	TRAF3IP3	1.17	1.32	-0.95
A_16_P15431767	206341015	206341074	TRAF3IP3	0.27	0.16	-1.19
A_14_P130537	206344270	206344329	C1orf74	0.56	0.97	-1.12
A_16_P35450175	206351367	206351424	IRF6	1.04	0.40	-0.80
A_14_P127584	206355229	206355287	IRF6	1.17	0.18	-1.11
A_16_P15431836	206360698	206360757	IRF6	0.98	0.68	-0.66
A_16_P35450228	206366195	206366254	IRF6	1.12	0.41	-0.70
A_16_P15431877	206375212	206375271	chr1:206375212-206375271	0.62	0.50	-0.83
A_16_P35450279	206382537	206382596	chr1:206382537-206382596	0.65	1.65	-0.92
A_16_P00244981	206392521	206392580	C1orf107	0.05	0.42	-1.12
A_16_P00244997	206400724	206400783	C1orf107	0.80	1.40	-0.73
A_14_P139185	206413255	206413314	C1orf107	0.86	1.18	-1.02
A_14_P139450	206416791	206416850	C1orf107	1.11	0.68	-0.81
A_16_P15432045	206436374	206436433	chr1:206436374-206436433	0.52	0.92	-0.95
A_16_P15432116	206463710	206463769	chr1:206463710-206463769	0.37	1.11	-0.99
A_16_P15432142	206485808	206485867	chr1:206485808-206485867	0.56	0.56	-1.06
A_16_P15432160	206494347	206494406	chr1:206494347-206494406	0.59	0.83	-0.92
A_14_P100003	206501334	206501393	SYT14	-0.11	-0.12	-0.87
A_16_P15432211	206512121	206512180	SYT14	0.43	1.45	-0.74
A_16_P15432225	206518387	206518446	SYT14	0.83	0.65	-0.84
A_16_P15432245	206530155	206530214	SYT14	-0.33	-0.02	-1.38
A_16_P35450660	206551090	206551149	SYT14	0.53	1.20	-0.91
A_16_P15432252	206559617	206559676	SYT14	0.04	1.07	-0.90
A_16_P15432262	206566161	206566220	SYT14	0.45	1.01	-1.00
A_16_P35450697	206571795	206571854	SYT14	0.47	1.17	-1.02
A_14_P111404	206582775	206582834	SYT14	0.89	1.19	-0.98
A_16_P35450766	206595476	206595535	SYT14	0.50	1.36	-0.21
A_16_P15432364	206606224	206606283	SYT14	0.27	0.66	-1.11
A_14_P104138	206615456	206615515	SYT14	0.52	0.04	-0.79
A_16_P15432402	206623268	206623327	SYT14	0.41	0.78	-1.03
A_16_P15432429	206634430	206634489	SYT14	0.03	0.51	-1.08
A_16_P35450864	206640407	206640466	SYT14	0.30	1.89	-1.06
A_16_P00245307	206649354	206649413	SYT14	0.95	1.14	-0.79
A_16_P15432476	206656008	206656067	SYT14	0.63	1.06	-0.87
A_14_P120767	206662148	206662206	SYT14	0.72	1.57	-0.90
A_16_P35450937	206672020	206672079	SYT14	0.24	1.63	-0.99
A_16_P15432533	206682685	206682744	SYT14	1.30	0.98	-1.00
A_16_P15432558	206692723	206692782	SYT14	0.10	1.48	-1.16

A_16_P15432580	206704345	206704404	SYT14	0.27	1.74	-1.06
A_16_P15432616	206714029	206714088	SYT14	0.38	1.10	-0.76
A_16_P15432628	206720862	206720921	SYT14	0.25	1.13	-1.28
A_14_P136248	206729737	206729796	chr1:206729737-206729796	0.57	1.62	-0.90
A_16_P15432696	206745787	206745846	chr1:206745787-206745846	0.31	1.55	-0.88
A_16_P00245464	206760386	206760445	chr1:206760386-206760445	1.44	0.80	-0.67
A_16_P15432737	206773133	206773192	chr1:206773133-206773192	1.03	1.34	-0.85
A_16_P35451174	206780909	206780968	chr1:206780909-206780968	0.70	1.65	-0.80
A_16_P15432776	206791876	206791935	chr1:206791876-206791935	0.19	0.87	-0.98
A_16_P00245507	206796604	206796663	SERTAD4	0.49	1.34	-0.79
A_14_P128677	206803380	206803439	SERTAD4	0.55	1.13	-1.14
A_16_P00245552	206816328	206816387	chr1:206816328-206816387	0.90	0.36	-0.84
A_16_P00245567	206824387	206824446	chr1:206824387-206824446	0.87	1.20	-0.82
A_16_P00245577	206833947	206834006	chr1:206833947-206834006	0.26	0.88	-0.89
A_16_P00245611	206862481	206862540	chr1:206862481-206862540	0.15	2.01	-0.77
A_16_P15433009	206874189	206874248	chr1:206874189-206874248	0.76	1.19	-0.76
A_14_P131124	206887767	206887826	chr1:206887767-206887826	0.75	0.49	-0.43
A_16_P00245651	206896351	206896410	HHAT	0.68	1.23	-0.73
A_16_P15433050	206903639	206903698	HHAT	0.56	0.99	-1.17
A_14_P100808	206910753	206910809	HHAT	0.05	1.03	-1.07
A_16_P00245683	206922952	206923011	HHAT	0.90	1.19	-1.02
A_16_P35451543	206929842	206929901	HHAT	0.63	0.53	-0.91
A_16_P15433133	206935749	206935808	HHAT	0.19	1.75	-1.10
A_16_P35451589	206945858	206945917	HHAT	1.01	1.24	-0.80
A_16_P15433179	206952955	206953014	HHAT	-0.31	0.54	-1.56
A_16_P15433198	206960500	206960559	HHAT	0.86	1.50	-0.89
A_16_P15433216	206965948	206966007	HHAT	-0.08	0.83	-0.86
A_16_P35451665	206971574	206971633	HHAT	0.78	0.98	-0.75
A_16_P35451674	206977172	206977231	HHAT	1.06	1.29	-1.00
A_16_P00245787	206989940	206989999	HHAT	-0.71	0.76	-0.83
A_16_P35451703	206995740	206995799	HHAT	0.74	1.44	-0.96
A_16_P00245797	207007032	207007091	HHAT	0.45	0.81	-1.10
A_16_P35451744	207016646	207016705	HHAT	1.13	1.49	-1.06
A_16_P00245839	207029434	207029493	HHAT	0.96	0.50	-0.92
A_16_P15433390	207040914	207040973	HHAT	0.33	1.14	-1.11
A_16_P00245873	207048046	207048105	HHAT	0.28	-0.18	-0.99
A_16_P15433435	207058484	207058543	HHAT	0.42	1.10	-0.35
A_16_P00245904	207065993	207066052	HHAT	-0.27	1.50	-0.85
A_14_P138787	207074866	207074925	HHAT	0.48	1.44	-1.08
A_16_P15433500	207082694	207082753	HHAT	1.02	1.18	-1.06
A_16_P00245937	207089256	207089315	HHAT	0.57	1.33	-0.90
A_16_P00245965	207102951	207103010	HHAT	1.02	1.49	-0.76
A_16_P35452035	207113267	207113326	HHAT	0.54	0.55	-0.96
A_16_P00246003	207121170	207121229	HHAT	0.19	1.13	-0.96
A_14_P115803	207131410	207131469	HHAT	1.06	1.13	-0.99
A_16_P15433666	207137477	207137536	HHAT	-0.19	1.07	-1.08
A_16_P15433675	207143677	207143736	HHAT	-0.18	-0.07	-1.39
A_16_P15433701	207151946	207152005	HHAT	0.70	0.95	-0.38

A_16_P15433708	207162968	207163027	HHAT	0.79	1.18	-0.94
A_16_P15433729	207173770	207173829	HHAT	0.63	1.64	-0.47
A_16_P00246073	207179645	207179704	HHAT	0.15	1.20	-0.75
A_16_P00246088	207185367	207185423	HHAT	0.93	1.23	-0.86
A_16_P00246102	207192896	207192955	HHAT	0.72	1.60	-0.50
A_16_P35452241	207198547	207198606	HHAT	0.99	1.24	-1.13
A_16_P00246127	207210299	207210358	HHAT	0.34	0.82	-1.00
A_16_P00246144	207218727	207218786	HHAT	-0.23	-0.55	-0.73
A_16_P15433868	207231150	207231209	HHAT	0.59	1.11	-1.02
A_14_P131922	207237768	207237827	HHAT	0.91	1.25	-0.79
A_16_P00246196	207248465	207248524	KCNH1	0.62	0.66	-1.11
A_16_P00246220	207261989	207262048	KCNH1	0.58	0.54	-0.95
A_16_P35452449	207270240	207270299	KCNH1	0.60	0.81	-0.82
A_16_P00246247	207278186	207278245	KCNH1	0.89	1.30	-0.81
A_16_P35452502	207288837	207288896	KCNH1	0.56	0.81	-0.96
A_16_P15434068	207295021	207295080	KCNH1	0.23	0.95	-0.66
A_16_P00246295	207302101	207302160	KCNH1	0.65	1.18	-1.05
A_16_P00246319	207313849	207313908	KCNH1	0.77	0.84	-0.99
A_14_P134308	207328744	207328803	KCNH1	0.86	0.84	-0.66
A_16_P15434186	207339164	207339223	KCNH1	0.56	0.53	-0.84
A_16_P35452666	207349729	207349788	KCNH1	0.94	0.83	-0.95
A_16_P15434233	207356686	207356745	KCNH1	0.77	1.25	-0.52
A_16_P35452707	207364118	207364177	KCNH1	-0.22	0.54	-1.04
A_16_P15434266	207374272	207374331	KCNH1	0.08	0.31	-1.02
A_16_P00246423	207384974	207385033	KCNH1	0.36	0.14	-1.01
A_14_P123459	207392434	207392493	KCNH1	0.94	1.30	-0.80
A_16_P15434326	207401581	207401640	KCNH1	0.16	1.47	-1.03
A_16_P15434347	207410954	207411013	KCNH1	0.16	0.67	-1.09
A_16_P00246473	207417912	207417971	KCNH1	0.61	0.90	-0.70
A_16_P15434395	207425267	207425326	KCNH1	0.09	1.12	-1.01
A_16_P35452876	207436540	207436599	KCNH1	0.24	0.88	-1.21
A_16_P00246505	207444184	207444243	KCNH1	0.78	0.71	-0.80
A_16_P35452898	207454342	207454401	KCNH1	0.57	1.34	-1.01
A_16_P00246522	207460754	207460813	KCNH1	1.06	0.84	-0.56
A_16_P15434491	207471590	207471649	KCNH1	0.47	0.20	-0.80
A_16_P15434530	207483159	207483218	KCNH1	0.43	1.05	-0.79
A_16_P15434543	207497885	207497942	KCNH1	1.13	0.52	-0.71
A_16_P15434569	207507854	207507913	KCNH1	0.00	0.16	-0.95
A_16_P00246604	207514442	207514501	KCNH1	0.42	0.42	-0.75
A_16_P15434589	207521779	207521838	KCNH1	0.41	0.70	-0.74
A_16_P00246608	207534543	207534602	KCNH1	0.90	-0.08	-0.87
A_16_P15434613	207543850	207543909	KCNH1	0.26	0.59	-0.90
A_16_P00246621	207550789	207550848	KCNH1	0.06	1.13	-1.12
A_16_P00246637	207569149	207569208	KCNH1	0.08	0.48	-0.87
A_14_P201447	207580430	207580489	KCNH1	0.67	0.99	-0.74
A_14_P129795	207580550	207580600	KCNH1	0.91	1.16	-0.86
A_16_P15434671	207587142	207587201	KCNH1	0.16	1.30	-0.90
A_16_P15434680	207598795	207598854	KCNH1	0.46	1.52	-0.90

A_16_P15434685	207608092	207608151	KCNH1	0.44	0.40	-0.84
A_16_P15434701	207615037	207615096	KCNH1	0.45	0.72	-0.98
A_16_P00246678	207629333	207629392	KCNH1	0.92	0.33	0.20
A_16_P15434713	207635775	207635834	KCNH1	0.79	0.78	-0.94
A_16_P15434720	207644058	207644117	KCNH1	0.41	0.96	-0.94
A_16_P00246702	207652358	207652417	KCNH1	0.25	1.25	-0.96
A_14_P110041	207665245	207665304	KCNH1	-0.03	1.94	-0.84
A_16_P35453300	207681199	207681258	KCNH1	0.72	0.70	-0.87
A_16_P35453317	207688610	207688669	KCNH1	0.65	0.84	-0.78
A_14_P113582	207696776	207696835	chr1:207696776-207696835	0.53	0.20	-0.84
A_16_P35453371	207710441	207710500	chr1:207710441-207710500	0.43	0.19	-0.84
A_16_P15434906	207728659	207728718	chr1:207728659-207728718	0.26	0.90	-0.86
A_16_P15434922	207739453	207739512	chr1:207739453-207739512	0.54	1.00	-0.88
A_16_P15434946	207750864	207750923	chr1:207750864-207750923	0.63	1.35	-0.80
A_16_P35453426	207760195	207760254	chr1:207760195-207760254	0.83	1.22	-0.81
A_16_P15434975	207767243	207767302	chr1:207767243-207767302	0.68	1.40	-0.92
A_16_P15434991	207773706	207773765	chr1:207773706-207773765	1.05	0.47	-0.79
A_16_P35453476	207781084	207781143	chr1:207781084-207781143	0.42	1.28	-0.98
A_16_P00246856	207797474	207797533	chr1:207797474-207797533	0.95	1.10	-0.66
A_16_P00246871	207815179	207815238	chr1:207815179-207815238	0.59	1.13	-0.97
A_14_P135295	207821878	207821936	RCOR3	0.58	0.10	-0.68
A_14_P128936	207833011	207833070	RCOR3	0.58	1.06	-0.92
A_16_P00246920	207842334	207842393	RCOR3	0.86	0.83	-0.92
A_16_P00246925	207850937	207850996	RCOR3	1.13	0.81	-0.83
A_14_P120059	207857405	207857463	RCOR3	0.84	1.25	-0.75
A_16_P35453666	207865758	207865817	RCOR3	0.39	1.43	-0.98
A_16_P35453685	207871334	207871393	RCOR3	0.59	1.03	-0.98
A_16_P15435224	207876940	207876999	RCOR3	0.76	1.15	-0.87
A_16_P15435243	207886326	207886385	chr1:207886326-207886385	0.89	1.02	-0.90
A_16_P15435270	207894755	207894814	TRAF5	0.97	1.18	-0.87
A_16_P15435284	207903115	207903174	TRAF5	1.31	1.02	-0.66
A_14_P115646	207908473	207908532	TRAF5	-0.33	1.18	-1.25
A_16_P35453797	207916112	207916171	TRAF5	0.57	0.51	-1.03
A_16_P00247036	207921783	207921842	TRAF5	0.47	1.14	-1.04
A_14_P126851	207931169	207931228	TRAF5	0.37	0.93	-1.00
A_16_P35453866	207936661	207936720	chr1:207936661-207936720	-0.02	1.96	-1.35
A_16_P15435386	207943971	207944030	chr1:207943971-207944030	0.58	0.19	-1.02
A_16_P00247091	207953619	207953678	C1orf97	0.66	1.09	-1.05
A_16_P15435423	207959127	207959186	C1orf97	0.48	0.94	-0.90
A_16_P35453930	207978751	207978810	C1orf97	0.54	1.24	-0.85
A_16_P15435439	207987983	207988042	C1orf97	0.55	0.96	-0.84
A_14_P135951	207994183	207994242	C1orf97	1.01	0.69	-0.91
A_16_P00247126	208004789	208004848	chr1:208004789-208004848	-0.31	0.78	-0.86
A_16_P00247127	208026723	208026782	chr1:208026723-208026782	0.42	0.51	-0.82
A_16_P35453997	208035509	208035568	chr1:208035509-208035568	0.73	1.38	-0.85
A_14_P104021	208039357	208039416	C1orf36	0.48	0.44	-1.12
A_16_P00247158	208045548	208045607	C1orf36	0.69	1.50	-0.96
A_16_P00247158	208045548	208045607	C1orf36	0.66	1.55	-1.01

A_16_P00247158	208045548	208045607	C1orf36	0.69	1.50	-0.94
A_16_P35454043	208050307	208050363	C1orf36	1.04	0.48	-0.67
A_16_P00247172	208054396	208054452	C1orf36	1.55	1.42	-0.71
A_16_P00247179	208060497	208060555	chr1:208060497-208060555	1.30	1.23	-0.43
A_16_P00247188	208065196	208065255	chr1:208065196-208065255	1.05	1.24	-0.71
A_16_P15435592	208073724	208073783	chr1:208073724-208073783	-0.14	0.55	-0.76
A_16_P00247240	208104637	208104696	chr1:208104637-208104696	0.66	1.23	-0.91
A_16_P15435688	208116942	208117001	chr1:208116942-208117001	0.81	0.15	-0.97
A_16_P35454229	208128463	208128522	chr1:208128463-208128522	0.54	0.79	-0.69
A_14_P124186	208137749	208137808	SLC30A1	0.74	0.88	0.30
A_16_P15435769	208143743	208143802	chr1:208143743-208143802	0.56	1.66	-0.67
A_16_P15435782	208148352	208148411	chr1:208148352-208148411	0.22	1.01	-0.93
A_16_P00247315	208155951	208156010	chr1:208155951-208156010	1.31	1.88	-0.58
A_16_P15435825	208166700	208166759	chr1:208166700-208166759	1.12	0.77	-0.84
A_16_P00247365	208183669	208183728	chr1:208183669-208183728	0.99	1.11	-0.69
A_16_P15435938	208206076	208206135	CR605189	1.14	0.61	-0.54
A_16_P00247405	208212248	208212307	CR605189	0.64	0.12	-0.88
A_16_P00247420	208217999	208218058	chr1:208217999-208218058	1.07	0.03	-0.65
A_16_P00247424	208224508	208224567	NEK2	0.35	1.16	-0.90
A_14_P136417	208228616	208228675	NEK2	0.53	0.72	-0.77
A_14_P108980	208228706	208228765	NEK2	0.35	0.66	-1.45
A_14_P101182	208228820	208228871	NEK2	-0.12	0.80	-0.73
A_16_P15436007	208233008	208233067	NEK2	0.43	1.10	-0.94
A_16_P15436027	208238318	208238377	chr1:208238318-208238377	0.28	1.03	-1.37
A_16_P15436037	208243279	208243338	chr1:208243279-208243338	0.89	0.79	-1.00
A_16_P00247479	208265005	208265064	chr1:208265005-208265064	0.85	0.59	-0.56
A_16_P00247508	208293331	208293390	chr1:208293331-208293390	0.88	0.97	-0.63
A_14_P136437	208307829	208307888	LPGAT1	0.69	1.22	-0.94
A_16_P15436212	208324634	208324693	LPGAT1	0.58	1.09	-0.81
A_16_P15436244	208337932	208337991	LPGAT1	0.42	1.63	-0.64
A_14_P108003	208344950	208345009	LPGAT1	0.56	0.86	-0.40
A_16_P15436303	208359155	208359214	LPGAT1	0.79	1.21	-0.72
A_16_P35454842	208366551	208366610	LPGAT1	0.21	1.00	-0.92
A_16_P15436327	208374216	208374275	LPGAT1	0.30	0.32	-0.81
A_16_P35454871	208380574	208380633	LPGAT1	0.91	1.38	-0.97
A_14_P110503	208390826	208390885	LPGAT1	0.65	1.57	-0.97
A_14_P124471	208410005	208410064	chr1:208410005-208410064	0.20	0.30	-0.95
A_16_P15436435	208433353	208433412	chr1:208433353-208433412	1.06	1.23	-0.88
A_16_P15436510	208469490	208469549	chr1:208469490-208469549	0.51	1.30	-0.87
A_16_P15436554	208485730	208485789	chr1:208485730-208485789	0.92	0.96	-1.00
A_16_P15436581	208494899	208494956	chr1:208494899-208494956	1.19	0.99	-0.64
A_14_P131903	208502918	208502973	BC009918	0.75	0.87	-0.50
A_14_P113487	208508330	208508389	INTS7	0.80	1.67	-0.74
A_16_P15436649	208514312	208514371	INTS7	0.86	0.96	-0.76
A_16_P15436654	208520509	208520568	INTS7	0.59	1.29	-0.76
A_16_P00247813	208528156	208528215	INTS7	0.32	1.28	-1.06
A_14_P105003	208544494	208544553	INTS7	0.70	1.02	-0.79
A_16_P15436747	208562160	208562219	INTS7	0.48	0.62	-0.88

A_14_P112821	208568418	208568477	INTS7	0.20	0.87	-0.74
A_16_P00247892	208581874	208581933	INTS7	0.38	0.57	-1.01
A_16_P15436796	208587513	208587572	INTS7	0.73	1.16	-0.83
A_16_P00247914	208595874	208595933	INTS7	0.67	1.16	-0.93
A_14_P139799	208606416	208606475	DTL	0.37	1.48	-0.88
A_16_P15436866	208616594	208616651	DTL	0.97	1.14	-0.76
A_14_P118274	208630338	208630397	DTL	0.67	1.02	-0.81
A_14_P112385	208642342	208642399	DTL	0.95	1.05	-0.64
A_16_P15436932	208648086	208648145	DTL	-0.03	1.17	-0.83
A_14_P108506	208666099	208666158	DTL	0.90	1.35	-0.77
A_16_P35455574	208675984	208676043	chr1:208675984-208676043	1.04	0.58	-0.67
A_16_P15437036	208697206	208697265	chr1:208697206-208697265	0.63	0.32	-0.80
A_16_P00248053	208715373	208715432	chr1:208715373-208715432	0.89	1.34	-0.92
A_16_P15437111	208738639	208738698	chr1:208738639-208738698	1.10	1.10	-0.72
A_14_P113447	208774600	208774659	chr1:208774600-208774659	0.60	1.27	-0.73
A_16_P15437235	208792885	208792944	chr1:208792885-208792944	0.71	1.70	-0.94
A_16_P35455850	208808730	208808789	chr1:208808730-208808789	0.69	0.99	-0.79
A_16_P15437308	208822292	208822351	chr1:208822292-208822351	1.19	1.63	-0.57
A_16_P15437332	208836457	208836516	chr1:208836457-208836516	1.22	1.32	-0.86
A_16_P15437347	208843274	208843333	chr1:208843274-208843333	0.38	1.48	-0.78
A_14_P134318	208855394	208855453	PPP2R5A	0.55	1.96	-0.50
A_16_P15437399	208861281	208861340	PPP2R5A	0.56	0.44	-0.54
A_16_P15437412	208875040	208875099	PPP2R5A	0.48	0.87	-0.55
A_16_P15437423	208880817	208880876	PPP2R5A	0.59	0.70	-0.98
A_14_P126109	208890958	208891017	PPP2R5A	1.01	0.88	-0.77
A_16_P15437461	208897233	208897292	PPP2R5A	0.73	0.88	-0.95
A_16_P35456078	208907654	208907713	PPP2R5A	0.63	0.97	-0.80
A_16_P15437521	208918759	208918818	PPP2R5A	0.66	1.41	-0.68
A_14_P124982	208926403	208926462	C1orf75	0.94	0.69	-0.46
A_16_P00248345	208936937	208936996	C1orf75	0.57	0.39	-0.96
A_16_P15437580	208942980	208943039	C1orf75	0.65	1.09	-0.69
A_16_P15437604	208951805	208951864	C1orf75	0.95	0.89	-0.92
A_14_P137557	208960116	208960175	C1orf75	0.82	1.71	-0.91
A_16_P15437650	208977797	208977856	chr1:208977797-208977856	0.36	1.36	-1.07
A_16_P00248403	208981953	208982012	chr1:208981953-208982012	1.37	1.10	0.10
A_16_P15437659	208994393	208994450	chr1:208994393-208994450	0.84	0.87	-0.90
A_14_P137805	208996575	208996634	NENF	0.64	1.19	-0.69
A_16_P15437679	209002824	209002883	NENF	0.74	1.04	-0.74
A_16_P00248423	209007420	209007479	NENF	1.30	1.50	-0.68
A_14_P130479	209017422	209017481	chr1:209017422-209017481	0.85	0.88	-0.89
A_16_P15437781	209037102	209037161	chr1:209037102-209037161	0.94	1.39	-0.63
A_16_P00248505	209053451	209053510	chr1:209053451-209053510	1.02	1.44	-0.83
A_16_P00248524	209072811	209072870	BC028700	1.11	0.75	-0.89
A_16_P00248566	209093509	209093568	chr1:209093509-209093568	0.77	1.05	-0.93
A_16_P15437972	209108295	209108354	chr1:209108295-209108354	0.80	1.60	-0.92
A_16_P15437997	209118029	209118088	chr1:209118029-209118088	0.32	0.77	-1.22
A_16_P15438038	209131340	209131399	ATF3	0.33	0.40	-1.08
A_16_P35456647	209142376	209142435	ATF3	0.89	0.46	-0.87

A_16_P00248650	209149399	209149458	ATF3	0.72	1.14	-1.04
A_16_P35456684	209156188	209156247	ATF3	1.09	1.58	-0.93
A_16_P00248668	209161213	209161272	ATF3	0.52	0.68	-0.30
A_16_P15438122	209165597	209165656	ATF3	0.18	1.09	-1.00
A_16_P15438150	209173039	209173098	ATF3	1.02	1.15	-0.72
A_16_P15438164	209176472	209176531	ATF3	0.84	0.18	-0.79
A_14_P201724	209180639	209180698	ATF3	0.76	1.05	-0.49
A_14_P108482	209180794	209180840	ATF3	0.45	0.01	-0.28
A_14_P200051	209187361	209187412	FAM71A	1.09	0.97	-0.41
A_14_P128969	209188468	209188524	FAM71A	0.84	0.45	-0.80
A_16_P00248743	209200339	209200398	chr1:209200339-209200398	1.48	1.22	-1.06
A_16_P15438265	209210912	209210971	chr1:209210912-209210971	0.45	0.58	-0.72
A_16_P15438310	209230959	209231018	chr1:209230959-209231018	0.80	1.24	-0.65
A_16_P15438330	209239553	209239611	chr1:209239553-209239611	1.00	1.00	-0.91
A_16_P00248811	209244086	209244145	chr1:209244086-209244145	0.87	1.48	-0.61
A_14_P101656	209256216	209256275	SNFT	1.03	0.60	-1.07
A_16_P35457007	209265045	209265104	chr1:209265045-209265104	0.65	1.30	-0.77
A_16_P00248847	209270361	209270420	chr1:209270361-209270420	0.28	1.03	-1.22
A_16_P35457024	209275098	209275157	chr1:209275098-209275157	0.82	1.42	-0.81
A_16_P35457026	209280169	209280228	chr1:209280169-209280228	1.04	0.90	-0.92
A_16_P35457045	209289206	209289265	AK124596	0.89	1.58	-0.92
A_14_P117379	209299315	209299374	chr1:209299315-209299374	0.09	0.88	-0.72
A_16_P35457094	209311437	209311496	C1orf48	0.41	0.35	-0.79
A_16_P15438512	209319996	209320055	C1orf48	0.70	1.02	-0.83
A_16_P15438513	209339498	209339557	C1orf48	0.56	1.24	-0.79
A_14_P137130	209352154	209352213	C1orf48	0.74	0.82	-0.65
A_16_P15438567	209359643	209359702	AL832248	0.75	1.11	-0.77
A_16_P00248946	209366118	209366177	AL832248	0.74	0.26	-0.80
A_16_P15438607	209377782	209377841	AL832248	0.63	0.84	-0.89
A_16_P15438626	209384863	209384922	chr1:209384863-209384922	1.18	0.64	-0.90
A_16_P15438641	209391814	209391873	chr1:209391814-209391873	0.59	-0.13	-0.96
A_16_P35457267	209397863	209397922	LOC149643	1.22	0.02	-0.90
A_16_P15438659	209404191	209404250	LOC149643	0.17	-0.91	-0.81
A_16_P35457276	209409386	209409445	chr1:209409386-209409445	1.01	1.19	-0.95
A_16_P15438672	209415513	209415572	AK092887	0.47	1.10	-0.75
A_16_P15438687	209421547	209421606	FLVCR	0.74	0.94	-0.81
A_16_P35457318	209428493	209428552	FLVCR	0.46	1.55	-0.81
A_16_P15438719	209434384	209434443	FLVCR	0.01	0.40	-1.03
A_16_P15438727	209440981	209441040	FLVCR	0.73	0.91	-0.89
A_14_P109954	209457020	209457078	FLVCR	1.42	1.05	-0.69
A_14_P130306	209458019	209458078	AK001419	0.27	0.29	-1.02
A_16_P15438773	209468318	209468377	chr1:209468318-209468377	0.99	1.30	-0.80
A_16_P00249061	209476954	209477013	chr1:209476954-209477013	1.03	0.70	-0.97
A_16_P15438814	209486752	209486811	chr1:209486752-209486811	1.59	1.06	-0.80
A_16_P15438828	209496394	209496453	chr1:209496394-209496453	0.45	0.99	-0.68
A_16_P15438844	209508619	209508678	chr1:209508619-209508678	0.87	1.28	-0.98
A_16_P00249103	209517406	209517465	FLJ12505	0.51	1.17	-0.76
A_14_P137823	209524848	209524907	FLJ12505	0.88	1.17	-0.85

A_16_P15438922	209536347	209536405	FLJ12505	1.40	0.47	-0.85
A_16_P00249157	209544199	209544258	FLJ12505	0.41	0.71	-0.72
A_16_P15438968	209549414	209549473	FLJ12505	0.57	-0.32	-1.04
A_14_P106718	209556238	209556297	ANGEL2	0.30	1.66	-0.90
A_16_P35457650	209562108	209562167	ANGEL2	0.71	2.03	-1.02
A_16_P15439024	209570135	209570194	ANGEL2	0.58	1.81	-0.91
A_14_P136174	209574698	209574757	ANGEL2	0.36	0.30	-0.40
A_16_P15439054	209579787	209579846	chr1:209579787-209579846	0.25	1.26	-1.30
A_16_P15439061	209584970	209585029	chr1:209584970-209585029	0.91	1.09	-0.79
A_16_P00249227	209597478	209597537	chr1:209597478-209597537	0.22	0.36	-0.85
A_16_P15439080	209604944	209605003	chr1:209604944-209605003	1.10	-0.05	0.06
A_16_P00249233	209612288	209612347	chr1:209612288-209612347	0.66	1.01	-0.80
A_16_P15439103	209618224	209618283	RPS6KC1	0.71	1.32	-0.65
A_16_P35457771	209627210	209627269	RPS6KC1	0.75	1.50	-1.00
A_16_P15439138	209633470	209633529	RPS6KC1	0.38	1.05	-0.90
A_14_P125983	209639478	209639537	RPS6KC1	0.46	1.40	-0.87
A_16_P15439187	209655727	209655786	RPS6KC1	0.10	0.81	-0.95
A_16_P35457853	209666117	209666176	RPS6KC1	0.74	1.33	-0.91
A_16_P00249307	209672681	209672740	RPS6KC1	0.51	0.44	-0.29
A_16_P15439236	209683671	209683730	RPS6KC1	0.50	1.47	-0.89
A_14_P126669	209691247	209691306	RPS6KC1	1.05	1.16	-0.66
A_16_P00249351	209698278	209698337	RPS6KC1	0.14	1.31	-0.92
A_16_P15439287	209706337	209706396	RPS6KC1	-0.13	1.47	-1.16
A_16_P00249365	209724831	209724890	RPS6KC1	0.43	0.84	-0.82
A_16_P15439349	209738219	209738278	RPS6KC1	0.82	0.30	-0.83
A_16_P15439362	209756865	209756921	RPS6KC1	0.71	0.80	-0.80
A_16_P15439362	209756865	209756921	RPS6KC1	0.55	1.15	-0.85
A_16_P15439362	209756865	209756921	RPS6KC1	0.64	1.07	-0.72
A_16_P00249407	209766869	209766928	RPS6KC1	0.24	0.74	-0.89
A_14_P113731	209773007	209773066	RPS6KC1	0.59	0.72	-1.02
A_16_P15439392	209782469	209782528	RPS6KC1	0.17	1.16	-1.19
A_16_P00249446	209796850	209796909	RPS6KC1	-0.05	1.18	-1.00
A_14_P120484	209803845	209803904	RPS6KC1	0.75	1.31	-0.93
A_16_P15439481	209811557	209811616	RPS6KC1	0.33	0.37	-1.20
A_16_P15439505	209817780	209817839	RPS6KC1	-0.16	-0.13	-1.17
A_16_P00249501	209824475	209824532	RPS6KC1	0.74	1.00	-0.82
A_16_P15439561	209838726	209838785	chr1:209838726-209838785	0.45	0.67	-0.98
A_16_P00249538	209847815	209847874	chr1:209847815-209847874	0.63	1.14	-0.83
A_14_P123070	209857100	209857159	chr1:209857100-209857159	0.41	0.54	-0.91
A_16_P15439651	209871089	209871148	chr1:209871089-209871148	0.63	1.22	-0.92
A_16_P00249645	209911617	209911676	chr1:209911617-209911676	1.00	1.31	-0.88
A_16_P15439915	209960599	209960658	chr1:209960599-209960658	0.62	1.43	-0.80
A_14_P100115	209984874	209984933	chr1:209984874-209984933	-0.16	0.92	-1.04
A_16_P00249797	209992587	209992646	chr1:209992587-209992646	0.90	1.83	-0.87
A_16_P00249804	210002841	210002900	chr1:210002841-210002900	0.06	0.24	-1.02
A_16_P15440058	210014660	210014717	chr1:210014660-210014717	0.76	1.15	-0.82
A_16_P35458765	210035444	210035503	chr1:210035444-210035503	0.71	1.13	-1.19
A_16_P15440170	210065317	210065376	chr1:210065317-210065376	0.34	1.12	-0.85

A_16_P00249916	210081793	210081852	chr1:210081793-210081852	0.71	0.99	-0.76
A_16_P00249953	210105673	210105732	chr1:210105673-210105732	0.70	0.72	-0.74
A_16_P00249985	210132304	210132363	chr1:210132304-210132363	0.82	0.91	-1.15
A_16_P00250012	210151766	210151825	chr1:210151766-210151825	0.33	1.73	-0.81
A_16_P00250073	210183306	210183365	chr1:210183306-210183365	0.55	1.10	-0.98
A_14_P135215	210208000	210208059	chr1:210208000-210208059	0.43	0.76	-0.79
A_16_P15440557	210228371	210228430	chr1:210228371-210228430	0.22	0.71	-0.70
A_16_P00250153	210248196	210248255	chr1:210248196-210248255	0.88	1.47	-0.73
A_16_P00250193	210283506	210283565	chr1:210283506-210283565	0.40	0.45	-1.10
A_16_P00250220	210299803	210299861	chr1:210299803-210299861	0.85	0.38	-0.85
A_16_P00250260	210325806	210325865	chr1:210325806-210325865	0.87	0.81	-0.88
A_16_P00250293	210342888	210342947	chr1:210342888-210342947	1.01	0.76	-0.77
A_16_P15440892	210364065	210364124	chr1:210364065-210364124	0.30	0.63	-1.11
A_16_P00250360	210381025	210381084	AK092251	0.64	1.44	-1.04
A_16_P00250390	210395603	210395662	AK092251	0.30	0.75	-0.73
A_16_P15441032	210412303	210412362	AK092251	0.58	0.59	-1.10
A_16_P35459775	210433780	210433839	AK092251	0.54	0.72	-1.25
A_16_P00250484	210444241	210444300	AK092251	0.15	1.01	-1.05
A_16_P15441150	210454769	210454828	AK092251	0.41	0.93	-1.12
A_16_P00250529	210469979	210470038	AK092251	0.95	0.82	-0.85
A_16_P00250579	210496015	210496074	AK092251	-0.09	1.35	-1.18
A_16_P15441326	210512178	210512237	AK092251	0.69	0.71	-1.05
A_14_P128404	210526599	210526658	AK092251	0.28	1.04	-0.72
A_16_P35460088	210538318	210538377	chr1:210538318-210538377	0.35	1.24	-1.06
A_14_P132471	210550049	210550099	U44060	0.97	0.97	-0.39
A_14_P134886	210559030	210559087	PROX1	0.38	0.76	-0.87
A_16_P35460224	210573438	210573497	PROX1	0.30	0.86	-1.20
A_16_P15441584	210585550	210585609	PROX1	0.33	0.25	-1.18
A_16_P00250786	210591141	210591200	PROX1	0.55	-0.79	-0.94
A_14_P104533	210597588	210597647	PROX1	0.73	1.01	-0.63
A_16_P15441672	210612791	210612850	chr1:210612791-210612850	0.62	2.02	-0.99
A_14_P121141	210635452	210635511	chr1:210635452-210635511	1.19	1.70	-0.74
A_16_P15441785	210661522	210661581	chr1:210661522-210661581	0.09	-0.11	-0.87
A_16_P15441896	210704763	210704822	chr1:210704763-210704822	0.79	0.71	-0.80
A_16_P15442001	210748499	210748558	chr1:210748499-210748558	0.45	0.73	-0.95
A_16_P15442147	210796516	210796575	chr1:210796516-210796575	1.05	1.41	-0.99
A_16_P15442200	210813920	210813979	chr1:210813920-210813979	0.12	1.22	-1.00
A_14_P125275	210824739	210824798	chr1:210824739-210824798	0.56	0.85	-0.75
A_16_P15442248	210833255	210833314	chr1:210833255-210833314	0.64	0.59	-0.94
A_16_P35460945	210841971	210842030	chr1:210841971-210842030	0.47	0.83	-1.06
A_16_P15442299	210852191	210852250	SMYD2	0.42	1.61	-0.44
A_16_P15442326	210861894	210861953	SMYD2	0.47	0.87	-0.94
A_16_P00251236	210868316	210868375	SMYD2	0.74	0.80	-0.92
A_16_P15442378	210879754	210879813	SMYD2	0.36	0.90	-0.98
A_14_P126738	210891934	210891991	SMYD2	-0.02	0.88	-1.07
A_14_P108715	210898554	210898613	SMYD2	0.43	0.44	-0.91
A_16_P35461140	210909618	210909677	chr1:210909618-210909677	0.64	1.61	-1.00
A_16_P15442477	210916186	210916245	chr1:210916186-210916245	0.57	0.58	-0.90

A_16_P00251341	210925791	210925850	PTPN14	1.16	1.34	-0.95
A_16_P00251350	210931575	210931634	PTPN14	0.62	0.41	-0.86
A_16_P35461230	210937099	210937158	PTPN14	0.76	0.75	-0.96
A_16_P00251384	210946487	210946546	PTPN14	1.05	1.10	-0.75
A_14_P125243	210955436	210955495	PTPN14	1.01	1.27	-0.25
A_16_P35461345	210969327	210969386	PTPN14	1.02	1.08	-0.62
A_16_P00251447	210978226	210978285	PTPN14	0.34	0.82	-0.87
A_16_P15442696	210984591	210984650	PTPN14	1.29	1.29	-0.77
A_16_P35461406	210990334	210990393	PTPN14	0.92	1.70	-1.06
A_16_P00251490	211001602	211001661	PTPN14	0.70	1.46	-0.80
A_14_P134274	211013581	211013639	PTPN14	0.32	0.69	-0.91
A_16_P15442807	211021625	211021684	PTPN14	0.78	1.11	-0.84
A_16_P15442824	211028119	211028178	PTPN14	0.57	0.85	-1.11
A_14_P116682	211043376	211043435	PTPN14	-0.34	0.41	-0.76
A_16_P35461589	211050284	211050343	PTPN14	0.79	0.97	-0.97
A_16_P15442932	211062089	211062148	PTPN14	0.86	1.49	-0.52
A_16_P00251619	211068325	211068384	PTPN14	1.05	1.34	-0.84
A_16_P15442971	211078515	211078574	PTPN14	0.88	1.25	-0.71
A_16_P15443000	211088071	211088130	PTPN14	0.41	0.37	-1.03
A_16_P00251683	211104710	211104769	PTPN14	-0.23	1.08	-0.25
A_16_P15443071	211110542	211110601	PTPN14	0.09	0.18	-0.71
A_14_P111073	211118685	211118744	chr1:211118685-211118744	0.17	2.11	-0.86
A_16_P15443113	211126341	211126400	chr1:211126341-211126400	0.18	1.67	-0.93
A_16_P15443141	211136030	211136089	chr1:211136030-211136089	0.45	0.73	-1.01
A_16_P15443154	211144937	211144996	chr1:211144937-211144996	0.66	0.50	-0.71
A_16_P35461858	211152169	211152217	chr1:211152169-211152217	1.29	1.03	-0.63
A_14_P123214	211165333	211165392	CENPF	0.87	1.04	-0.81
A_16_P15443202	211175187	211175246	CENPF	0.89	1.11	-0.97
A_14_P119136	211182633	211182692	CENPF	0.58	0.83	-0.59
A_16_P35461942	211190781	211190840	CENPF	0.25	1.74	-1.09
A_16_P35461979	211201667	211201726	CENPF	0.46	0.80	0.94
A_14_P103816	211214572	211214631	CENPF	0.11	0.66	1.02
A_14_P135064	211215490	211215549	CENPF	-0.16	1.99	0.72
A_14_P129226	211215610	211215669	CENPF	0.60	1.04	1.11
A_14_P129226	211215610	211215669	CENPF	0.62	1.11	1.16
A_14_P129226	211215610	211215669	CENPF	0.46	0.94	1.26
A_14_P201983	211226166	211226225	CENPF	0.92	1.23	1.12
A_16_P15443379	211232667	211232726	chr1:211232667-211232726	0.60	0.70	0.88
A_16_P15443404	211242946	211243005	chr1:211242946-211243005	0.54	1.00	1.09
A_16_P00251903	211252013	211252072	chr1:211252013-211252072	0.30	1.55	0.81
A_16_P15443457	211278765	211278824	chr1:211278765-211278824	-0.11	1.04	0.77
A_16_P15443516	211309244	211309303	chr1:211309244-211309303	0.97	1.69	0.67
A_14_P100718	211364313	211364372	chr1:211364313-211364372	0.66	1.90	0.87
A_16_P35462384	211395327	211395386	chr1:211395327-211395386	0.34	1.20	1.22
A_16_P15443763	211438238	211438297	chr1:211438238-211438297	0.71	1.33	1.05
A_16_P15443822	211466210	211466269	chr1:211466210-211466269	0.65	1.32	0.92
A_16_P15443953	211509249	211509308	chr1:211509249-211509308	0.33	0.86	0.97
A_16_P15444070	211549000	211549059	chr1:211549000-211549059	0.53	0.90	0.96

A_16_P00252307	211564531	211564590	chr1:211564531-211564590	-0.19	0.34	0.98
A_16_P00252327	211572389	211572448	KCNK2	1.20	1.01	1.03
A_16_P15444154	211582562	211582621	KCNK2	0.55	1.48	1.01
A_16_P15444173	211589523	211589582	KCNK2	0.56	0.81	0.86
A_16_P35462921	211601168	211601227	KCNK2	0.69	1.00	0.94
A_16_P35462945	211609688	211609747	KCNK2	0.43	1.32	1.11
A_16_P15444215	211615908	211615967	KCNK2	0.33	1.39	1.23
A_16_P00252386	211622233	211622292	KCNK2	1.16	1.15	1.28
A_14_P200938	211630525	211630584	KCNK2	0.34	0.16	0.76
A_14_P136977	211645077	211645131	KCNK2	-0.13	0.98	1.13
A_16_P15444342	211656012	211656071	KCNK2	0.25	0.97	1.13
A_16_P15444375	211666421	211666480	KCNK2	0.56	1.14	0.95
A_14_P112819	211675783	211675842	KCNK2	0.61	1.21	0.84
A_16_P15444416	211686244	211686303	KCNK2	0.21	0.18	0.73
A_16_P00252511	211695625	211695684	KCNK2	0.82	1.29	1.10
A_14_P109222	211709630	211709689	KCNK2	0.71	1.26	0.95
A_16_P00252558	211722825	211722884	KCNK2	0.75	1.15	0.98
A_16_P15444547	211733931	211733990	KCNK2	0.19	1.09	1.23
A_14_P112884	211747497	211747556	KCNK2	0.91	0.70	1.11
A_16_P15444595	211757652	211757711	KCNK2	0.01	0.83	1.09
A_16_P15444629	211768498	211768557	KCNK2	0.52	0.53	1.00
A_16_P35463410	211779290	211779349	KCNK2	0.37	0.65	0.94
A_14_P109121	211795590	211795649	KCNK2	0.74	1.83	1.15
A_16_P15444748	211814071	211814130	chr1:211814071-211814130	0.95	1.89	1.07
A_16_P15444811	211841191	211841250	chr1:211841191-211841250	0.60	0.64	1.24
A_16_P15444931	211893732	211893791	chr1:211893732-211893791	0.69	0.84	1.08
A_16_P15445023	211934411	211934470	chr1:211934411-211934470	0.56	0.98	1.22
A_14_P138659	211969342	211969401	chr1:211969342-211969401	1.07	1.39	1.12
A_16_P15445142	212013723	212013782	chr1:212013723-212013782	0.51	1.48	0.99
A_16_P15445256	212056488	212056547	chr1:212056488-212056547	0.66	1.23	1.09
A_14_P132391	212090320	212090379	chr1:212090320-212090379	0.47	0.69	0.84
A_16_P35464108	212108514	212108573	chr1:212108514-212108573	0.37	1.22	0.91
A_16_P15445376	212128315	212128374	chr1:212128315-212128374	0.26	1.36	1.12
A_14_P130613	212135789	212135848	KCTD3	0.33	1.33	0.96
A_16_P15445438	212152890	212152949	KCTD3	0.45	-0.09	0.90
A_14_P112215	212163869	212163928	KCTD3	0.20	0.33	1.03
A_16_P00253129	212173612	212173671	KCTD3	1.00	1.17	1.21
A_14_P127442	212183435	212183494	KCTD3	0.40	1.58	1.17
A_16_P35464358	212200849	212200908	USH2A	0.90	1.13	1.06
A_16_P00253200	212211926	212211985	USH2A	0.38	0.75	1.09
A_16_P15445636	212217858	212217917	USH2A	0.54	1.08	0.98
A_16_P15445655	212223877	212223936	USH2A	0.32	-1.08	0.87
A_16_P15445686	212233029	212233088	USH2A	0.21	0.92	0.76
A_16_P00253261	212241859	212241918	USH2A	1.10	1.00	1.17
A_14_P128440	212249269	212249328	USH2A	0.32	1.12	1.18
A_16_P00253282	212258261	212258320	USH2A	0.93	0.62	0.89
A_16_P15445778	212272351	212272410	USH2A	0.70	0.87	1.09
A_16_P15445801	212278856	212278915	USH2A	0.80	0.53	1.00

A_16_P00253332	212288654	212288713	USH2A	0.55	1.17	0.91
A_16_P15445860	212297950	212298008	USH2A	0.63	0.65	1.13
A_16_P00253362	212304894	212304953	USH2A	0.34	1.40	1.17
A_16_P00253394	212320362	212320421	USH2A	0.59	1.69	1.22
A_16_P00253410	212328336	212328395	USH2A	0.23	0.72	0.92
A_16_P15445975	212333817	212333876	USH2A	0.43	0.61	0.82
A_16_P15446012	212344488	212344547	USH2A	-0.12	-0.40	0.74
A_16_P00253457	212351788	212351847	USH2A	0.11	1.00	1.05
A_16_P15446061	212361049	212361108	USH2A	0.26	0.99	0.85
A_16_P00253475	212367408	212367467	USH2A	0.41	0.05	1.02
A_16_P00253490	212375545	212375604	USH2A	1.09	1.41	1.13
A_16_P00253507	212387834	212387893	USH2A	0.77	0.51	1.09
A_16_P35464940	212400345	212400404	USH2A	0.80	1.65	1.11
A_16_P35464956	212405996	212406055	USH2A	0.58	0.70	1.08
A_16_P00253541	212412252	212412311	USH2A	1.07	0.74	1.05
A_14_P118417	212422616	212422675	USH2A	0.36	1.23	1.01
A_16_P15446208	212429350	212429409	USH2A	0.12	0.67	0.94
A_16_P35465049	212440463	212440522	USH2A	0.76	1.60	0.96
A_16_P00253601	212450645	212450704	USH2A	1.02	1.27	1.21
A_16_P15446315	212461934	212461993	USH2A	0.54	1.06	0.92
A_16_P15446348	212472343	212472402	USH2A	0.61	0.78	0.93
A_16_P35465169	212478897	212478956	USH2A	0.65	1.17	1.10
A_16_P35465198	212487257	212487316	USH2A	0.56	1.37	1.09
A_16_P35465217	212493039	212493098	USH2A	0.51	1.11	1.02
A_16_P15446446	212502591	212502650	USH2A	0.45	0.74	0.94
A_16_P15446464	212509250	212509309	USH2A	0.45	0.30	0.82
A_16_P15446478	212518737	212518796	USH2A	0.30	1.03	0.89
A_16_P00253744	212527041	212527100	USH2A	0.65	0.41	0.95
A_14_P123638	212532510	212532569	USH2A	0.47	1.30	0.88
A_16_P15446553	212546300	212546359	USH2A	0.59	0.90	0.79
A_16_P15446578	212554719	212554778	USH2A	0.30	1.35	1.17
A_16_P00253806	212560711	212560770	USH2A	0.20	1.53	0.90
A_16_P35465451	212579606	212579665	USH2A	0.51	1.37	0.92
A_16_P00253847	212589268	212589327	USH2A	0.95	0.93	1.21
A_16_P00253864	212600901	212600960	USH2A	0.27	1.02	0.99
A_16_P15446731	212610238	212610297	USH2A	0.07	1.12	0.88
A_16_P00253895	212616297	212616356	USH2A	0.75	1.02	1.04
A_16_P15446774	212623997	212624056	USH2A	0.55	0.84	0.99
A_16_P15446792	212631789	212631848	USH2A	0.43	1.16	0.99
A_16_P00253944	212639971	212640030	USH2A	0.50	0.47	0.78
A_14_P137296	212647519	212647578	USH2A	0.66	0.69	1.12
A_16_P00253984	212658810	212658869	USH2A	0.29	0.82	0.66
A_16_P15446907	212665111	212665170	USH2A	0.29	0.78	0.71
A_16_P00254003	212673128	212673187	USH2A	0.70	0.10	0.96
A_16_P35465776	212683311	212683370	USH2A	0.44	0.45	0.82
A_16_P15446974	212690194	212690253	USH2A	0.37	1.42	0.93
A_16_P15446985	212695708	212695767	USH2A	-0.20	0.80	0.77
A_16_P15447015	212705246	212705305	USH2A	0.33	0.55	1.02

A_16_P15447024	212715221	212715280	USH2A	0.79	1.42	0.93
A_16_P35465857	212724156	212724215	USH2A	0.27	1.30	0.69
A_16_P15447054	212731126	212731185	USH2A	0.05	1.43	1.00
A_16_P15447085	212741377	212741436	USH2A	0.61	1.00	1.10
A_16_P35465948	212751907	212751966	USH2A	0.76	1.63	1.05
A_14_P135641	212761622	212761681	USH2A	0.70	1.10	1.01
A_16_P00254164	212768989	212769048	USH2A	0.68	1.44	0.87
A_16_P35466027	212779262	212779321	USH2A	0.66	0.46	0.93
A_16_P35466048	212787018	212787077	USH2A	0.28	0.59	0.72
A_16_P00254210	212796829	212796888	USH2A	0.77	0.70	0.96
A_16_P35466117	212808276	212808335	USH2A	0.85	1.58	1.02
A_16_P15447318	212821407	212821466	USH2A	0.36	1.55	0.89
A_16_P15447327	212831401	212831460	USH2A	1.06	1.62	1.19
A_16_P15447350	212839969	212840028	USH2A	0.62	0.30	0.83
A_14_P108872	212854004	212854061	USH2A	-0.31	1.51	1.21
A_16_P15447415	212865864	212865923	USH2A	0.57	1.21	1.02
A_16_P35466277	212880589	212880648	USH2A	0.46	1.07	0.91
A_14_P108240	212887020	212887079	USH2A	-0.85	0.85	0.56
A_16_P15447477	212892831	212892890	USH2A	0.38	0.86	0.87
A_16_P15447483	212900396	212900455	USH2A	0.05	1.56	0.80
A_16_P15447505	212919987	212920046	USH2A	0.36	0.88	0.73
A_16_P15447540	212932529	212932588	USH2A	0.84	0.60	1.10
A_16_P35466422	212947468	212947527	USH2A	0.47	0.93	0.78
A_16_P15447612	212958432	212958491	USH2A	0.24	0.74	0.72
A_16_P35466468	212968302	212968361	USH2A	0.51	1.56	0.99
A_16_P00254457	212980366	212980425	USH2A	0.82	1.42	0.89
A_16_P35466547	212992791	212992850	chr1:212992791-212992850	0.38	0.42	0.94
A_16_P35466568	213000487	213000546	chr1:213000487-213000546	0.28	0.73	0.77
A_16_P15447786	213030459	213030518	chr1:213030459-213030518	0.38	1.46	0.76
A_16_P00254563	213045449	213045508	chr1:213045449-213045508	0.43	0.75	0.75
A_14_P201532	213062779	213062838	chr1:213062779-213062838	0.19	0.57	0.60
A_14_P110748	213066274	213066333	ESRRG	0.33	0.57	1.06
A_16_P00254596	213073202	213073261	ESRRG	0.57	0.93	0.83
A_16_P15447889	213080344	213080403	ESRRG	0.63	1.56	1.09
A_16_P15447915	213087421	213087480	ESRRG	0.33	1.54	0.92
A_16_P00254650	213097202	213097261	ESRRG	0.92	1.12	1.00
A_16_P15447966	213103535	213103594	ESRRG	0.15	-0.04	0.76
A_16_P15448014	213117022	213117081	ESRRG	0.66	-0.11	0.98
A_16_P15448038	213124526	213124585	ESRRG	0.52	0.89	0.91
A_16_P15448074	213134398	213134457	ESRRG	0.24	1.02	0.85
A_16_P00254752	213145338	213145397	ESRRG	0.54	0.30	0.80
A_16_P35467019	213159851	213159910	ESRRG	0.37	0.92	0.93
A_16_P15448192	213169565	213169624	ESRRG	0.53	1.14	1.05
A_16_P15448210	213175360	213175419	ESRRG	0.37	1.67	0.90
A_16_P15448233	213181692	213181751	ESRRG	-0.14	0.83	0.81
A_16_P15448263	213192003	213192062	ESRRG	0.64	0.82	0.91
A_16_P00254876	213203545	213203604	ESRRG	0.78	1.32	0.99
A_14_P117754	213212693	213212747	ESRRG	0.75	0.92	0.80

A_16_P15448374	213224694	213224753	ESRRG	0.22	0.43	0.85
A_16_P15448391	213230334	213230393	ESRRG	0.23	0.74	0.65
A_16_P35467292	213239904	213239963	ESRRG	0.62	0.80	1.00
A_16_P00254967	213245429	213245488	ESRRG	0.68	0.75	1.04
A_16_P00254984	213253263	213253322	ESRRG	0.31	0.89	0.57
A_16_P35467372	213263432	213263491	ESRRG	0.65	0.57	0.95
A_16_P15448539	213276542	213276601	ESRRG	0.11	0.63	0.71
A_14_P104196	213285070	213285129	ESRRG	0.11	1.11	0.71
A_16_P15448597	213292169	213292228	ESRRG	-0.19	0.54	0.91
A_16_P15448622	213300902	213300961	ESRRG	0.68	0.86	0.91
A_16_P15448641	213306361	213306420	ESRRG	0.82	1.57	0.95
A_16_P15448663	213312772	213312831	ESRRG	0.12	0.64	0.82
A_16_P35467572	213319862	213319921	ESRRG	0.76	0.95	0.85
A_16_P15448726	213332233	213332292	ESRRG	0.40	0.95	0.90
A_16_P15448750	213339596	213339655	ESRRG	0.09	0.67	0.71
A_16_P35467659	213346574	213346633	ESRRG	0.52	1.22	0.95
A_16_P15448795	213354869	213354928	ESRRG	1.05	1.76	0.89
A_16_P15448824	213365152	213365211	ESRRG	0.83	0.80	1.08
A_16_P35467736	213373149	213373208	ESRRG	-0.07	1.11	0.90
A_16_P35467764	213381169	213381228	ESRRG	0.53	1.79	0.85
A_14_P123548	213390597	213390656	ESRRG	1.02	1.59	1.02
A_16_P00255292	213407420	213407479	ESRRG	0.19	0.96	0.72
A_16_P00255320	213420226	213420285	ESRRG	0.57	1.38	0.90
A_16_P15449008	213426748	213426807	ESRRG	0.42	0.43	0.94
A_16_P15449021	213438171	213438230	ESRRG	0.75	1.05	1.04
A_16_P15449052	213447721	213447780	ESRRG	0.37	0.97	0.89
A_16_P15449094	213459233	213459292	ESRRG	0.39	0.44	1.02
A_16_P35468025	213472646	213472705	ESRRG	-0.04	1.24	0.76
A_16_P15449160	213481207	213481266	ESRRG	0.53	0.19	0.90
A_16_P00255440	213491685	213491744	ESRRG	0.88	1.26	1.02
A_14_P131927	213501314	213501373	ESRRG	-0.31	2.42	0.94
A_16_P00255469	213508326	213508385	ESRRG	0.40	0.67	0.82
A_16_P15449272	213518374	213518433	ESRRG	0.09	0.74	0.67
A_16_P15449306	213533820	213533879	ESRRG	0.55	1.20	0.82
A_16_P35468228	213544366	213544425	ESRRG	0.41	1.18	0.87
A_16_P35468262	213555943	213556002	ESRRG	0.64	0.48	0.82
A_16_P35468291	213564835	213564894	ESRRG	0.31	0.70	0.83
A_16_P35468320	213572910	213572969	ESRRG	0.21	0.61	0.75
A_16_P15449448	213578756	213578815	ESRRG	0.32	1.08	0.83
A_16_P00255619	213590107	213590166	ESRRG	0.69	0.98	0.76
A_16_P15449518	213601626	213601685	ESRRG	1.01	0.86	1.00
A_16_P00255650	213607905	213607964	ESRRG	0.33	1.04	1.16
A_14_P110080	213618317	213618376	ESRRG	0.59	2.35	1.06
A_16_P15449610	213630558	213630617	ESRRG	0.53	1.28	0.75
A_16_P35468524	213637405	213637464	ESRRG	-0.17	-0.34	0.88
A_16_P15449669	213648592	213648651	ESRRG	0.41	0.94	0.91
A_16_P00255769	213666919	213666978	BX647076	-0.07	0.62	0.64
A_16_P35468663	213681127	213681186	BX647076	0.07	1.36	1.05

A_16_P15449795	213690121	213690179	BX647076	0.53	1.41	0.97
A_16_P15449847	213705007	213705066	chr1:213705007-213705066	0.33	1.50	0.96
A_14_P117687	213726453	213726512	chr1:213726453-213726512	0.82	1.27	0.85
A_16_P35468946	213802266	213802325	chr1:213802266-213802325	0.36	0.88	0.78
A_16_P35469082	213857243	213857302	chr1:213857243-213857302	0.81	0.33	0.87
A_16_P15450260	213893986	213894045	chr1:213893986-213894045	0.40	1.72	1.03
A_16_P00256160	213936376	213936435	chr1:213936376-213936435	0.89	1.62	1.17
A_16_P00256210	213970331	213970390	chr1:213970331-213970390	0.60	0.17	1.08
A_16_P15450491	213982209	213982268	chr1:213982209-213982268	0.60	1.14	0.93
A_14_P115849	213992427	213992486	GPATC2	0.84	1.35	0.95
A_16_P15450550	214006843	214006902	GPATC2	0.40	0.69	1.12
A_16_P00256298	214019975	214020032	GPATC2	0.31	-0.13	0.84
A_16_P35469537	214035551	214035610	GPATC2	0.58	1.45	0.94
A_14_P131469	214053378	214053437	GPATC2	0.11	0.91	0.63
A_16_P35469594	214065883	214065942	GPATC2	0.39	0.72	1.07
A_16_P15450746	214082257	214082316	GPATC2	0.23	1.78	0.88
A_16_P15450788	214097672	214097731	GPATC2	0.45	1.26	0.90
A_16_P15450809	214103733	214103792	GPATC2	0.60	0.55	0.96
A_16_P15450833	214113941	214114000	GPATC2	0.49	0.79	0.83
A_16_P15450851	214120205	214120264	GPATC2	0.30	0.06	0.85
A_16_P15450884	214130969	214131028	GPATC2	0.35	0.96	0.84
A_14_P101314	214137045	214137104	GPATC2	0.46	0.95	0.90
A_16_P35469836	214151481	214151540	GPATC2	0.79	1.38	0.98
A_16_P15450957	214157551	214157610	GPATC2	0.52	1.27	0.95
A_16_P00256535	214163706	214163765	GPATC2	0.73	0.84	1.17
A_14_P114359	214169901	214169960	GPATC2	0.37	0.80	0.96
A_16_P35469945	214181588	214181647	GPATC2	0.40	0.44	0.79
A_16_P15451052	214187067	214187126	GPATC2	0.34	0.58	1.00
A_14_P111752	214193228	214193286	SPATA17	0.31	1.21	0.98
A_16_P15451095	214200418	214200477	SPATA17	0.20	0.97	1.00
A_14_P117151	214210676	214210732	SPATA17	-0.26	1.16	0.81
A_16_P35470061	214221540	214221599	SPATA17	0.74	1.47	0.86
A_16_P15451178	214230809	214230868	SPATA17	-0.15	0.76	0.57
A_16_P35470116	214238669	214238728	SPATA17	0.21	1.53	0.98
A_16_P15451223	214245084	214245143	SPATA17	0.42	0.78	0.98
A_16_P35470152	214251488	214251547	SPATA17	0.19	1.32	0.70
A_16_P00256709	214259629	214259688	SPATA17	0.68	1.57	-0.08
A_16_P15451256	214271913	214271972	SPATA17	0.68	1.29	1.05
A_16_P15451283	214282037	214282096	SPATA17	0.54	0.61	1.07
A_16_P15451315	214291828	214291887	SPATA17	0.47	1.18	0.93
A_16_P15451349	214303919	214303978	SPATA17	0.96	0.26	0.89
A_16_P35470286	214311211	214311270	SPATA17	0.81	0.97	0.88
A_16_P15451396	214318601	214318660	SPATA17	-0.15	0.19	0.68
A_16_P00256810	214324144	214324203	SPATA17	0.30	0.78	1.25
A_16_P00256825	214336051	214336110	SPATA17	0.05	1.39	0.82
A_16_P15451451	214342916	214342975	SPATA17	0.45	0.22	1.00
A_16_P35470398	214354011	214354070	SPATA17	0.14	1.22	0.88
A_14_P135206	214363493	214363552	SPATA17	-0.04	1.50	0.76

A_16_P15451522	214370986	214371045	SPATA17	0.61	0.99	1.06
A_16_P00256893	214381733	214381792	SPATA17	0.39	0.31	1.10
A_16_P15451563	214388427	214388486	SPATA17	0.26	0.95	0.81
A_14_P132509	214394916	214394975	SPATA17	0.15	1.09	0.87
A_16_P15451631	214414457	214414516	SPATA17	0.41	1.34	1.09
A_16_P35470589	214428777	214428836	SPATA17	0.46	1.31	0.91
A_14_P127049	214444151	214444210	chr1:214444151-214444210	0.46	1.48	0.82
A_16_P15451754	214460705	214460764	BC040896	0.69	0.56	0.86
A_16_P15451834	214489427	214489486	chr1:214489427-214489486	0.70	0.57	1.00
A_16_P35470833	214515609	214515668	chr1:214515609-214515668	0.79	1.86	0.99
A_16_P15451977	214550344	214550403	chr1:214550344-214550403	0.39	0.99	0.91
A_16_P15452046	214592252	214592309	chr1:214592252-214592309	0.86	0.80	0.12
A_14_P109765	214611903	214611962	BC039113	0.49	1.31	1.01
A_16_P00257266	214639065	214639124	chr1:214639065-214639124	0.81	0.80	1.05
A_16_P15452229	214666142	214666201	chr1:214666142-214666201	0.79	1.08	0.94
A_16_P15452286	214700870	214700929	chr1:214700870-214700929	0.54	1.29	1.05
A_16_P00257354	214722716	214722775	chr1:214722716-214722775	0.80	1.56	0.99
A_14_P102457	214749887	214749946	chr1:214749887-214749946	0.31	1.39	0.80
A_16_P15452543	214790880	214790939	chr1:214790880-214790939	0.34	0.45	1.15
A_16_P15452643	214823281	214823340	chr1:214823281-214823340	0.39	1.28	1.12
A_16_P35471578	214836088	214836147	chr1:214836088-214836147	0.57	1.11	1.03
A_14_P134449	214847087	214847141	CGI-115	0.75	0.80	1.15
A_16_P15452702	214855791	214855850	CGI-115	0.63	1.09	0.99
A_14_P103284	214869236	214869295	CGI-115	0.11	1.67	1.21
A_16_P15452765	214876959	214877018	CGI-115	0.40	1.04	0.89
A_16_P15452785	214883459	214883518	CGI-115	0.02	0.77	0.93
A_16_P00257655	214892830	214892889	CGI-115	0.38	1.13	1.11
A_16_P15452858	214908271	214908330	TGFB2	0.85	0.88	1.16
A_16_P15452907	214921600	214921659	TGFB2	0.49	1.11	1.09
A_14_P106726	214933792	214933851	TGFB2	0.34	1.42	0.82
A_16_P35471900	214944594	214944653	TGFB2	1.19	1.38	1.13
A_16_P15452997	214951794	214951853	TGFB2	0.76	0.94	0.66
A_16_P15453021	214960085	214960144	TGFB2	0.78	1.03	1.09
A_14_P114581	214975773	214975832	TGFB2	1.14	1.17	1.20
A_16_P15453105	214987849	214987908	TGFB2	0.96	1.07	1.14
A_16_P35472047	214996017	214996076	TGFB2	0.62	1.22	1.13
A_14_P120374	215003297	215003356	TGFB2	0.37	1.23	1.22
A_16_P00257888	215010055	215010114	chr1:215010055-215010114	0.73	0.91	1.17
A_16_P15453216	215024502	215024561	chr1:215024502-215024561	0.03	1.08	0.78
A_16_P15453288	215051151	215051210	chr1:215051151-215051210	0.21	0.71	1.24
A_14_P131185	215081885	215081944	chr1:215081885-215081944	0.79	1.61	0.46
A_16_P00258055	215108554	215108613	chr1:215108554-215108613	0.65	0.31	0.91
A_16_P00258092	215133470	215133529	chr1:215133470-215133529	0.31	1.14	1.00
A_16_P15453574	215165364	215165423	chr1:215165364-215165423	-0.19	1.73	1.42
A_16_P15453652	215199726	215199785	chr1:215199726-215199785	1.04	1.01	1.13
A_14_P105238	215229422	215229481	chr1:215229422-215229481	-0.07	1.37	0.87
A_16_P15453777	215246689	215246748	chr1:215246689-215246748	1.04	0.86	1.04
A_16_P15453826	215265498	215265557	chr1:215265498-215265557	0.83	1.55	1.02

A_16_P35472832	215293850	215293909	chr1:215293850-215293909	0.67	1.41	0.90
A_16_P15453992	215334073	215334132	chr1:215334073-215334132	0.96	1.52	0.98
A_16_P15454051	215356531	215356590	chr1:215356531-215356590	0.57	1.37	1.01
A_14_P112543	215394348	215394407	chr1:215394348-215394407	0.26	-1.33	0.85
A_16_P15454245	215439518	215439577	chr1:215439518-215439577	0.70	1.08	1.06
A_16_P35473253	215468035	215468094	chr1:215468035-215468094	0.49	0.83	1.20
A_16_P35473317	215493250	215493309	chr1:215493250-215493309	0.46	0.29	1.15
A_16_P15454470	215523302	215523361	chr1:215523302-215523361	0.71	0.46	0.83
A_16_P00258710	215552811	215552870	chr1:215552811-215552870	0.26	0.48	0.92
A_16_P15454579	215574219	215574278	chr1:215574219-215574278	0.24	0.99	0.80
A_16_P35473518	215599356	215599415	chr1:215599356-215599415	-0.55	0.65	0.31
A_16_P35473541	215614193	215614252	chr1:215614193-215614252	0.39	0.83	1.13
A_16_P15454676	215638100	215638159	chr1:215638100-215638159	0.16	1.57	0.95
A_16_P15454704	215649847	215649906	BC086863	0.67	0.70	1.25
A_16_P15454707	215662277	215662336	BC086863	0.79	0.80	1.19
A_16_P15454713	215672022	215672081	BC086863	0.76	1.50	0.81
A_16_P00258833	215687884	215687943	BC086863	0.82	1.21	1.00
A_14_P118664	215705732	215705791	BC086863	0.57	1.35	1.09
A_16_P15454811	215718441	215718500	BC086863	0.37	0.92	0.86
A_16_P35473739	215726528	215726587	BC086863	0.29	0.94	0.89
A_16_P00258909	215733302	215733361	BC086863	0.76	1.38	1.07
A_14_P132275	215740862	215740921	LYPLAL1	0.28	1.09	0.85
A_16_P00258940	215754831	215754890	LYPLAL1	0.39	1.34	0.88
A_16_P35473844	215763534	215763593	LYPLAL1	0.58	0.97	1.02
A_14_P127881	215772273	215772332	LYPLAL1	0.20	1.18	0.78
A_16_P15454975	215778757	215778816	chr1:215778757-215778816	0.49	0.54	1.00
A_16_P15454999	215787250	215787309	chr1:215787250-215787309	0.71	1.06	0.96
A_16_P15455034	215798868	215798927	chr1:215798868-215798927	-0.07	2.37	0.90
A_14_P129970	215842111	215842170	chr1:215842111-215842170	0.38	1.20	1.14
A_16_P00259109	215864100	215864159	chr1:215864100-215864159	0.62	0.84	1.06
A_16_P15455306	215903765	215903824	chr1:215903765-215903824	0.44	0.58	0.99
A_16_P35474311	215944917	215944976	chr1:215944917-215944976	0.82	0.76	0.94
A_16_P35474354	215967244	215967303	chr1:215967244-215967303	0.43	0.64	0.83
A_16_P35474406	215985242	215985301	chr1:215985242-215985301	-0.16	0.61	0.83
A_16_P15455545	216001186	216001245	chr1:216001186-216001245	0.63	0.76	0.99
A_14_P105353	216024591	216024650	chr1:216024591-216024650	0.34	1.13	1.05
A_16_P35474590	216050427	216050486	chr1:216050427-216050486	0.21	0.09	1.01
A_16_P35474687	216086426	216086485	chr1:216086426-216086485	0.97	1.17	1.20
A_16_P15455851	216110763	216110822	chr1:216110763-216110822	1.09	0.80	1.10
A_16_P00259547	216129600	216129659	chr1:216129600-216129659	0.19	0.89	0.89
A_16_P00259576	216151537	216151589	chr1:216151537-216151589	-0.19	0.95	0.50
A_16_P00259581	216175292	216175351	chr1:216175292-216175351	-0.33	1.66	-0.53
A_14_P108375	216188985	216189044	chr1:216188985-216189044	0.46	1.06	-0.30
A_16_P35475013	216201515	216201574	chr1:216201515-216201574	0.48	0.64	-0.40
A_16_P00259647	216217911	216217970	chr1:216217911-216217970	0.87	0.83	-0.30
A_16_P15456201	216240127	216240186	chr1:216240127-216240186	0.21	1.17	-0.48
A_16_P00259716	216255261	216255320	AK097467	0.63	0.96	-0.32
A_16_P15456284	216271401	216271460	AK097467	0.86	1.06	-0.34

A_16_P15456318	216294466	216294525	AK097467	0.95	1.40	-0.25
A_16_P15456360	216309254	216309313	AK097467	0.35	-0.10	-0.16
A_16_P15456404	216325951	216326010	AK097467	0.78	0.76	-0.29
A_16_P15456416	216336283	216336342	AK097467	1.29	1.25	-0.20
A_14_P136554	216352157	216352216	AK097467	0.89	1.35	-0.23
A_16_P00259853	216363467	216363526	AK097467	0.66	1.19	-0.38
A_16_P15456535	216379391	216379450	AK097467	0.68	0.98	-0.37
A_16_P15456570	216388968	216389027	AK097467	0.45	0.68	-0.52
A_16_P00259913	216398475	216398534	AK097467	1.01	1.50	-0.39
A_16_P15456592	216407972	216408031	AK097467	0.49	0.76	-0.32
A_16_P15456600	216421624	216421683	AK097467	0.43	1.02	-0.56
A_16_P35475530	216435355	216435414	AK097467	0.19	1.19	-0.55
A_16_P35475563	216444080	216444139	AK097467	0.31	1.09	-0.23
A_14_P103370	216459708	216459767	AK097467	1.00	0.83	-0.21
A_16_P00259984	216473796	216473855	AK097467	0.52	2.80	-0.21
A_14_P106815	216477653	216477708	SLC30A10	0.92	0.93	-0.02
A_16_P00260014	216488831	216488890	SLC30A10	0.43	-0.04	-0.23
A_16_P35475704	216497683	216497742	AK097467	0.64	0.25	-0.26
A_16_P35475716	216502650	216502709	AK097467	1.10	1.07	-0.27
A_16_P15456794	216508479	216508538	AK097467	0.50	0.98	-0.22
A_16_P00260051	216514153	216514212	AK097467	0.53	0.51	-0.51
A_16_P15456820	216519741	216519800	AK097467	0.14	0.48	-0.56
A_16_P00260082	216533804	216533863	EPRS	0.09	0.58	-0.53
A_16_P35475827	216545930	216545989	EPRS	0.48	1.08	-0.49
A_16_P00260118	216551630	216551689	EPRS	-0.18	1.08	-0.38
A_14_P126747	216559029	216559087	EPRS	0.05	1.41	-0.46
A_16_P00260143	216566963	216567022	EPRS	0.20	0.94	-0.51
A_16_P35475912	216572637	216572694	EPRS	0.30	0.89	-0.38
A_16_P00260165	216581718	216581777	EPRS	0.69	0.82	-0.37
A_16_P15457033	216596617	216596676	EPRS	0.51	0.77	-0.18
A_14_P129713	216607379	216607438	EPRS	0.13	0.29	-0.46
A_14_P123254	216619553	216619612	BPNT1	0.20	0.84	-0.63
A_14_P123290	216620566	216620625	BPNT1	1.03	0.96	-0.31
A_16_P15457113	216631079	216631138	BPNT1	0.02	1.29	-0.68
A_16_P00260245	216645703	216645762	BPNT1	0.31	0.81	-0.46
A_14_P108159	216651182	216651241	BPNT1	0.62	0.92	-0.48
A_16_P00260260	216657845	216657904	IARS2	0.71	1.81	-0.56
A_16_P35476158	216665165	216665224	IARS2	0.13	0.82	-0.36
A_16_P00260279	216668780	216668839	IARS2	0.68	1.29	-0.31
A_16_P00260284	216672634	216672693	IARS2	1.14	0.97	-0.32
A_16_P00260289	216676159	216676218	IARS2	0.55	1.44	-0.39
A_16_P15457225	216680837	216680896	IARS2	1.03	0.75	-0.34
A_16_P15457231	216683138	216683197	IARS2	0.41	1.45	-0.39
A_14_P102979	216686469	216686528	IARS2	0.99	1.36	-0.13
A_16_P15457250	216689248	216689307	IARS2	0.68	1.07	-0.36
A_16_P00260316	216698612	216698671	IARS2	0.56	0.80	-0.29
A_16_P00260323	216701905	216701964	IARS2	0.06	0.84	-0.60
A_16_P00260330	216705468	216705527	IARS2	0.32	0.96	-0.52

A_14_P126537	216710157	216710216	AK021928	0.44	0.97	-0.25
A_16_P35476303	216714878	216714937	RAB3GAP2	0.72	0.49	-0.34
A_16_P15457348	216726478	216726537	RAB3GAP2	0.49	1.08	-0.48
A_16_P15457365	216732554	216732613	RAB3GAP2	0.28	1.18	-0.55
A_14_P128185	216744543	216744602	RAB3GAP2	0.33	0.65	-0.60
A_16_P00260402	216751798	216751857	RAB3GAP2	0.61	1.14	-0.36
A_16_P00260416	216757966	216758025	RAB3GAP2	0.77	1.01	-0.41
A_16_P00260424	216764007	216764066	RAB3GAP2	0.01	0.73	-0.33
A_14_P101981	216774646	216774705	RAB3GAP2	0.43	0.82	-0.10
A_16_P00260469	216787608	216787667	RAB3GAP2	1.35	0.88	-0.20
A_16_P00260480	216794509	216794568	RAB3GAP2	0.50	1.48	-0.48
A_14_P127570	216801431	216801490	RAB3GAP2	0.85	0.71	-0.35
A_16_P15457571	216814005	216814064	RAB3GAP2	0.28	0.27	-0.70
A_14_P129325	216826308	216826367	RAB3GAP2	0.09	0.81	-0.47
A_16_P00260521	216836171	216836230	chr1:216836171-216836230	0.64	1.33	-0.53
A_16_P15457644	216845930	216845989	chr1:216845930-216845989	0.48	1.46	-0.43
A_16_P00260585	216882213	216882270	chr1:216882213-216882270	0.81	0.63	-0.22
A_14_P125217	216907266	216907325	chr1:216907266-216907325	0.76	1.18	-0.49
A_16_P15457901	216945373	216945432	chr1:216945373-216945432	1.07	0.92	-0.19
A_16_P15457955	216975689	216975748	chr1:216975689-216975748	0.75	0.57	-0.53
A_16_P15458068	217020744	217020803	chr1:217020744-217020803	1.00	1.22	-0.48
A_14_P114579	217066040	217066099	chr1:217066040-217066099	0.16	-0.17	-0.34
A_16_P15458232	217081692	217081751	chr1:217081692-217081751	0.38	1.89	-0.54
A_16_P00260916	217094327	217094386	MARK1	0.05	0.55	-0.58
A_14_P113855	217108912	217108971	MARK1	0.29	1.79	-0.34
A_16_P35477345	217118869	217118928	MARK1	0.32	0.93	-0.62
A_16_P35477357	217125813	217125872	MARK1	0.83	1.07	-0.30
A_16_P00260951	217131737	217131796	MARK1	0.09	1.08	0.47
A_14_P117920	217141185	217141242	MARK1	1.21	1.02	-0.34
A_16_P15458385	217147191	217147250	MARK1	0.41	0.79	-0.64
A_16_P00260995	217154601	217154660	MARK1	1.03	1.12	-0.21
A_14_P122571	217165775	217165834	MARK1	0.36	1.41	-0.59
A_16_P15458454	217176641	217176700	MARK1	-0.05	0.77	-0.68
A_16_P35477520	217183998	217184057	MARK1	0.24	1.20	-0.61
A_16_P00261048	217189834	217189892	MARK1	0.62	1.30	-0.31
A_14_P137871	217201924	217201983	MARK1	0.41	0.52	-0.53
A_16_P35477585	217211486	217211545	MARK1	0.83	1.26	-0.37
A_14_P100335	217223823	217223882	MARK1	0.87	0.34	-0.43
A_16_P00261117	217235272	217235331	chr1:217235272-217235331	0.09	0.06	-0.49
A_16_P00261123	217239783	217239842	chr1:217239783-217239842	0.31	1.17	-0.52
A_16_P15458634	217245486	217245545	chr1:217245486-217245545	0.61	1.14	-0.29
A_14_P135863	217258565	217258624	C1orf115	0.71	1.34	-0.55
A_16_P15458686	217263055	217263114	chr1:217263055-217263114	1.07	1.16	-0.39
A_16_P15458697	217268715	217268774	chr1:217268715-217268774	-0.12	3.37	-0.66
A_16_P00261179	217274909	217274968	chr1:217274909-217274968	0.63	0.81	-0.35
A_16_P00261191	217293620	217293679	chr1:217293620-217293679	0.42	0.78	-0.42
A_16_P35477797	217306394	217306453	chr1:217306394-217306453	0.53	0.50	-0.48
A_14_P103747	217312598	217312657	MOSC2	0.28	0.95	-0.73

A_16_P00261226	217323475	217323534	MOSC2	0.69	0.16	-0.31
A_14_P115271	217336800	217336859	MOSC2	0.52	1.10	-0.49
A_14_P131287	217346415	217346474	BC010366	0.24	1.34	-0.65
A_16_P15458892	217359556	217359615	MOSC1	1.24	0.67	-0.05
A_16_P00261300	217365187	217365246	MOSC1	0.96	0.82	-0.48
A_14_P118184	217375895	217375954	MOSC1	0.44	1.02	-0.29
A_16_P00261335	217381923	217381982	chr1:217381923-217381982	0.50	0.97	-0.28
A_16_P00261361	217395427	217395486	chr1:217395427-217395486	0.42	0.53	-0.63
A_16_P00261381	217416088	217416147	chr1:217416088-217416147	0.48	1.06	-0.17
A_16_P15459050	217435373	217435432	chr1:217435373-217435432	0.70	0.89	-0.57
A_14_P108178	217446670	217446716	HLX1	0.88	0.36	0.29
A_16_P15459113	217453577	217453636	chr1:217453577-217453636	0.72	0.77	-0.40
A_16_P35478207	217458320	217458364	chr1:217458320-217458364	0.78	0.78	0.15
A_16_P15459132	217463806	217463865	chr1:217463806-217463865	0.56	1.19	-0.22
A_16_P00261463	217472440	217472499	chr1:217472440-217472499	0.11	1.23	-0.69
A_16_P00261494	217487039	217487098	chr1:217487039-217487098	0.58	1.14	-0.45
A_14_P104878	217516749	217516808	chr1:217516749-217516808	0.51	0.94	-0.22
A_16_P35478440	217548550	217548609	chr1:217548550-217548609	0.78	0.51	-0.28
A_16_P15459471	217593945	217594004	chr1:217593945-217594004	0.42	1.29	-0.78
A_16_P35478615	217630485	217630544	chr1:217630485-217630544	0.65	1.42	-0.43
A_16_P00261752	217663362	217663421	chr1:217663362-217663421	0.56	0.70	-0.39
A_14_P116203	217717502	217717561	chr1:217717502-217717561	0.94	1.74	-0.21
A_16_P15459856	217747611	217747670	chr1:217747611-217747670	0.92	1.31	-0.37
A_16_P00261937	217792400	217792459	chr1:217792400-217792459	0.32	0.96	-0.61
A_16_P00261979	217828044	217828103	chr1:217828044-217828103	0.33	1.16	-0.65
A_16_P15460078	217853121	217853180	chr1:217853121-217853180	0.83	0.63	-0.62
A_16_P15460111	217869121	217869180	chr1:217869121-217869180	0.73	1.79	-0.49
A_16_P00262040	217878396	217878455	chr1:217878396-217878455	0.54	0.84	-0.35
A_16_P00262049	217885966	217886025	chr1:217885966-217886025	0.78	1.32	-0.56
A_16_P35479251	217898008	217898067	chr1:217898008-217898067	0.82	1.29	-0.52
A_14_P117478	217905466	217905525	chr1:217905466-217905525	0.24	0.73	0.18
A_16_P35479302	217914695	217914754	chr1:217914695-217914754	0.42	1.22	-0.81
A_16_P00262114	217933890	217933949	chr1:217933890-217933949	1.34	1.10	-0.26
A_16_P15460379	217997100	217997159	chr1:217997100-217997159	0.56	0.58	-1.25
A_14_P122199	218042729	218042788	chr1:218042729-218042788	1.08	1.31	-0.34
A_16_P35479637	218081017	218081076	chr1:218081017-218081076	0.81	0.79	-0.26
A_16_P00262400	218135726	218135785	chr1:218135726-218135785	0.83	0.98	-0.52
A_16_P15460775	218171130	218171189	chr1:218171130-218171189	0.62	0.54	-0.41
A_14_P120333	218206255	218206314	chr1:218206255-218206314	1.24	0.89	-0.43
A_16_P35479964	218235044	218235103	chr1:218235044-218235103	0.50	1.52	-0.43
A_16_P00262510	218251751	218251810	chr1:218251751-218251810	0.32	0.75	-0.43
A_14_P126288	218263439	218263498	DUSP10	0.88	2.40	-0.54
A_16_P35480079	218278270	218278329	DUSP10	0.35	0.82	-0.51
A_14_P115785	218287399	218287458	DUSP10	0.67	1.25	-0.36
A_16_P00262598	218299112	218299171	DUSP10	0.52	0.59	-0.43
A_16_P15461116	218312072	218312131	chr1:218312072-218312131	0.84	0.83	-0.28
A_16_P15461154	218323046	218323105	chr1:218323046-218323105	0.45	1.00	-0.59
A_14_P136237	218336312	218336371	chr1:218336312-218336371	-0.23	0.59	-0.28

A_16_P35480366	218371280	218371339	chr1:218371280-218371339	0.73	1.48	-0.55
A_16_P15461383	218409380	218409439	chr1:218409380-218409439	0.75	1.33	-0.42
A_14_P120542	218446914	218446973	chr1:218446914-218446973	0.67	0.68	-0.52
A_16_P00262885	218464276	218464335	chr1:218464276-218464335	0.55	1.29	-0.14
A_16_P00262935	218500298	218500357	chr1:218500298-218500357	0.32	1.24	-0.64
A_16_P00262946	218525816	218525875	chr1:218525816-218525875	0.92	1.48	-0.53
A_16_P00262952	218541687	218541746	chr1:218541687-218541746	0.92	0.14	-0.32
A_16_P15461723	218565746	218565805	chr1:218565746-218565805	0.28	0.30	-0.76
A_16_P15461820	218600687	218600746	chr1:218600687-218600746	0.87	1.06	-0.41
A_16_P00263071	218619066	218619125	chr1:218619066-218619125	0.53	0.94	-0.54
A_16_P35481007	218645234	218645293	chr1:218645234-218645293	0.55	1.31	-0.47
A_14_P110009	218663191	218663250	BC023608	1.10	1.17	-0.32
A_16_P15462025	218676736	218676795	BC023608	0.78	1.14	-0.51
A_16_P35481127	218693108	218693167	BC023608	1.00	1.46	-0.28
A_16_P15462072	218713669	218713728	BC023608	0.20	0.99	-0.62
A_16_P15462478	218890498	218890557	BC023608	0.68	1.63	-0.35
A_16_P35482235	219178531	219178575	chr1:219178531-219178575	0.95	0.68	0.19
A_14_P115492	219238579	219238638	C1orf80	0.30	0.97	-0.50
A_16_P15463513	219300768	219300827	FLJ43505	0.81	0.19	-0.16
A_16_P00263218	219311634	219311693	FLJ43505	-0.19	0.76	-0.34
A_14_P127398	219503635	219503694	DISP1	0.87	0.73	0.99
A_14_P132961	219679851	219679898	chr1:219679851-219679898	0.70	1.29	-0.13
A_16_P00263233	219892814	219892873	SUSD4	0.86	0.96	-0.25
A_16_P35484084	219918538	219918597	chr1:219918538-219918597	1.32	1.20	-0.16
A_16_P35484092	219924777	219924836	chr1:219924777-219924836	0.90	0.89	-0.25
A_16_P35484107	219932436	219932495	chr1:219932436-219932495	0.41	1.54	-0.39
A_14_P133449	219940496	219940555	C1orf65	0.63	0.72	0.14
A_16_P15464976	219947115	219947174	chr1:219947115-219947174	0.71	1.32	-0.44
A_16_P15464985	219955012	219955071	chr1:219955012-219955071	0.96	1.02	-0.30
A_16_P15464995	219963364	219963423	chr1:219963364-219963423	0.94	1.25	-0.14
A_16_P00263308	219987037	219987096	chr1:219987037-219987096	0.76	0.15	-0.13
A_16_P00263331	220010695	220010754	chr1:220010695-220010754	0.83	1.07	-0.41
A_16_P00263409	220052836	220052895	chr1:220052836-220052895	0.85	1.53	-0.31
A_16_P00263448	220079917	220079976	chr1:220079917-220079976	0.50	2.21	-0.69
A_14_P138107	220151574	220151633	chr1:220151574-220151633	0.46	0.90	-0.17
A_16_P15465417	220177890	220177949	chr1:220177890-220177949	0.11	0.60	-0.55
A_16_P15465440	220192752	220192811	chr1:220192752-220192811	1.21	1.45	-0.30
A_16_P15465470	220208873	220208932	CAPN2	1.08	0.96	0.12
A_14_P137507	220216339	220216398	CAPN2	1.34	1.75	-0.05
A_16_P15465510	220222072	220222131	CAPN2	0.70	0.61	-0.20
A_16_P15465520	220228115	220228174	CAPN2	0.74	1.09	-0.05
A_16_P15465548	220237339	220237398	CAPN2	1.00	1.29	-0.28
A_14_P125883	220246482	220246530	CAPN2	0.31	0.68	0.14
A_16_P35484785	220260765	220260824	CAPN2	1.21	0.98	-0.17
A_16_P15465642	220269354	220269413	CAPN2	-0.70	1.40	-0.46
A_14_P130957	220275107	220275166	TP53BP2	0.33	1.48	-0.30
A_16_P35484855	220283382	220283441	TP53BP2	0.73	1.30	-0.30
A_16_P15465722	220295100	220295159	TP53BP2	0.97	0.42	-0.27

A_16_P00263725	220301344	220301403	TP53BP2	1.03	0.70	-0.23
A_16_P35484939	220309584	220309643	TP53BP2	0.97	0.86	-0.22
A_14_P128426	220315751	220315810	TP53BP2	0.55	0.91	-0.14
A_14_P106753	220333160	220333219	TP53BP2	0.47	0.67	-0.19
A_14_P201484	220340998	220341057	chr1:220340998-220341057	-0.06	0.37	-0.31
A_16_P00263794	220348726	220348785	chr1:220348726-220348785	1.03	1.29	-0.04
A_16_P00263801	220354866	220354925	chr1:220354866-220354925	0.26	0.35	-0.30
A_16_P15465899	220376689	220376748	chr1:220376689-220376748	1.02	0.48	-0.21
A_16_P15465945	220396240	220396289	chr1:220396240-220396289	0.47	0.75	-0.64
A_16_P35485535	220559941	220560000	chr1:220559941-220560000	0.46	1.09	0.17
A_16_P15466373	220580197	220580256	chr1:220580197-220580256	0.76	1.06	0.01
A_16_P00263899	220590925	220590984	chr1:220590925-220590984	0.65	0.61	0.03
A_16_P00263912	220604232	220604291	chr1:220604232-220604291	0.07	-0.03	-0.02
A_14_P133109	220611046	220611105	FBXO28	-0.07	1.34	0.06
A_16_P35485656	220619457	220619516	FBXO28	0.95	0.87	-0.21
A_16_P00263947	220628579	220628638	FBXO28	0.69	1.07	-0.25
A_16_P15466482	220640889	220640948	FBXO28	0.87	0.43	0.01
A_16_P35485719	220647572	220647631	FBXO28	0.36	0.72	-0.18
A_14_P129511	220656393	220656452	AB007952	0.45	0.24	-0.03
A_16_P00263994	220663888	220663947	chr1:220663888-220663947	0.53	0.48	-0.31
A_16_P00264001	220672081	220672140	CR602386	0.59	0.85	-0.27
A_14_P115850	220678560	220678619	DEGS1	0.53	0.83	-0.18
A_14_P127409	220680261	220680320	DEGS1	0.68	1.13	-0.25
A_14_P118522	220683574	220683633	DEGS1	-0.08	1.45	-0.43
A_16_P00264021	220690551	220690610	CR623303	1.18	1.35	-0.12
A_16_P00264031	220699885	220699944	chr1:220699885-220699944	0.83	1.14	0.10
A_16_P15466637	220705622	220705681	BC008027	0.64	1.93	-0.32
A_16_P15466652	220712470	220712529	chr1:220712470-220712529	0.39	0.52	-0.38
A_16_P15466671	220721829	220721888	NVL	0.21	0.95	-0.25
A_14_P111697	220730928	220730985	NVL	0.78	1.55	-0.28
A_16_P35485947	220742643	220742702	NVL	0.81	0.77	-1.02
A_16_P35485958	220751093	220751152	NVL	0.31	1.04	-0.44
A_14_P117996	220761080	220761139	NVL	0.87	1.05	-0.12
A_16_P15466767	220769724	220769783	NVL	1.20	1.15	-0.16
A_16_P15466795	220780568	220780627	NVL	0.67	0.76	-0.37
A_16_P35486050	220788655	220788714	NVL	0.55	0.98	-0.36
A_16_P00264151	220796341	220796400	NVL	0.56	0.75	-0.32
A_16_P00264161	220802485	220802544	NVL	0.77	1.07	-0.09
A_16_P15466869	220812306	220812365	NVL	0.33	1.16	-0.20
A_14_P130313	220820832	220820891	NVL	0.18	1.66	-0.25
A_16_P35486133	220827890	220827949	chr1:220827890-220827949	0.53	0.88	-0.45
A_16_P35486144	220840492	220840551	chr1:220840492-220840551	0.82	1.23	-0.15
A_16_P00264188	220845192	220845251	chr1:220845192-220845251	-0.19	0.34	-0.26
A_16_P00264190	220850378	220850437	chr1:220850378-220850437	1.21	1.26	-0.24
A_14_P129352	220858675	220858734	CNIH4	-0.15	1.07	-0.01
A_16_P15466940	220865681	220865740	CNIH4	0.21	0.82	-0.63
A_16_P35486205	220870175	220870234	CNIH4	0.03	0.91	-0.35
A_16_P15466972	220877699	220877758	chr1:220877699-220877758	0.78	0.85	-0.26

A_14_P137830	220883184	220883243	WDR26	0.38	1.04	-0.38
A_16_P35486279	220892487	220892546	WDR26	0.15	1.63	-0.40
A_16_P00264278	220900317	220900376	WDR26	0.61	1.37	-0.15
A_16_P00264286	220905910	220905969	WDR26	1.23	1.69	0.03
A_16_P00264295	220912679	220912738	WDR26	0.79	0.84	-0.38
A_14_P129346	220918950	220919003	WDR26	0.68	1.24	-0.26
A_16_P15467118	220925894	220925953	WDR26	0.55	1.07	-0.27
A_16_P15467143	220932912	220932971	CR625980	0.19	1.28	-0.26
A_16_P15467163	220942934	220942993	CR625980	0.61	1.23	-0.31
A_16_P00264364	220953057	220953116	CR625980	0.70	0.90	-0.03
A_16_P15467195	220967350	220967409	CR625980	0.46	1.09	-0.32
A_16_P15467202	220980286	220980345	CR625980	0.71	1.29	-0.33
A_16_P35486532	220999817	220999874	CR625980	0.37	1.52	-0.28
A_14_P124984	221009492	221009551	CR625980	0.76	0.37	-0.08
A_16_P15467329	221027159	221027218	chr1:221027159-221027218	0.66	1.14	-0.26
A_16_P00264468	221041378	221041437	chr1:221041378-221041437	0.38	0.81	0.12
A_16_P00264485	221059238	221059297	chr1:221059238-221059297	0.46	0.20	-0.29
A_16_P35486701	221078751	221078810	chr1:221078751-221078810	0.89	0.84	-0.18
A_16_P15467469	221098708	221098767	chr1:221098708-221098767	0.28	0.71	-0.21
A_14_P103068	221113209	221113268	CNIH3	1.08	1.05	-0.23
A_16_P35486802	221120804	221120863	CNIH3	0.55	0.60	-0.33
A_16_P00264575	221126858	221126917	CNIH3	0.02	0.51	-0.56
A_16_P15467560	221133223	221133282	CNIH3	0.14	-0.06	-0.62
A_16_P15467587	221143206	221143265	CNIH3	0.77	1.04	-0.25
A_16_P15467606	221149156	221149215	CNIH3	0.56	-0.07	-0.30
A_16_P00264622	221155378	221155437	CNIH3	0.08	0.54	-0.53
A_14_P124923	221162472	221162531	CNIH3	0.60	0.88	-0.30
A_16_P15467656	221169521	221169580	CNIH3	0.83	1.63	-0.62
A_16_P15467667	221175359	221175418	CNIH3	0.40	0.93	-0.13
A_14_P110914	221182673	221182732	CNIH3	-0.04	0.97	-0.03
A_16_P00264682	221192218	221192277	CNIH3	0.78	1.07	-0.25
A_16_P00264700	221203828	221203887	CNIH3	0.34	0.53	-0.42
A_14_P136150	221209415	221209474	CNIH3	0.43	1.18	-0.08
A_16_P00264722	221215912	221215971	CNIH3	1.08	1.67	-0.11
A_14_P100311	221234929	221234988	CNIH3	-0.12	0.30	-0.30
A_16_P35487157	221243573	221243632	chr1:221243573-221243632	0.91	1.75	-0.13
A_14_P108911	221259667	221259726	chr1:221259667-221259726	0.83	1.44	-0.13
A_16_P15468032	221305790	221305849	chr1:221305790-221305849	0.47	0.67	-0.46
A_16_P00264911	221334011	221334070	chr1:221334011-221334070	1.09	1.04	-0.32
A_16_P15468146	221356454	221356513	chr1:221356454-221356513	-0.18	1.08	-0.48
A_16_P00264967	221397007	221397066	chr1:221397007-221397066	0.48	0.81	-0.36
A_16_P00264981	221406660	221406719	chr1:221406660-221406719	0.35	1.42	-0.55
A_16_P35487532	221414591	221414650	chr1:221414591-221414650	-0.04	0.56	-0.37
A_16_P00264998	221421757	221421816	chr1:221421757-221421816	0.83	0.82	0.02
A_16_P00265003	221434169	221434228	BC042869	0.98	0.58	-0.23
A_16_P15468282	221446579	221446638	BC042869	0.54	0.69	-0.42
A_16_P35487595	221454719	221454778	BC042869	0.37	0.87	-0.39
A_14_P104058	221463207	221463266	BC042869	0.60	1.11	-0.49

A_16_P15468339	221476865	221476920	BC042869	0.98	1.61	0.43
A_16_P15468340	221495795	221495854	BC042869	0.52	-0.35	-0.02
A_16_P15468357	221501509	221501568	BC042869	0.23	1.08	-0.22
A_16_P35487660	221511624	221511683	BC042869	0.47	1.59	-0.47
A_16_P35487662	221518162	221518221	BC042869	0.33	1.42	-0.51
A_16_P35487670	221524231	221524290	chr1:221524231-221524290	0.74	1.40	-0.28
A_16_P35487690	221538990	221539049	chr1:221538990-221539049	0.42	1.57	-0.11
A_16_P00265084	221557056	221557115	chr1:221557056-221557115	0.93	1.46	-0.16
A_16_P15468422	221572923	221572982	chr1:221572923-221572982	0.38	0.51	-0.39
A_16_P15468443	221579512	221579571	chr1:221579512-221579571	1.29	1.45	-0.20
A_16_P15468461	221594855	221594914	chr1:221594855-221594914	0.37	1.03	-0.48
A_16_P35487777	221614118	221614177	chr1:221614118-221614177	0.58	1.01	-0.54
A_16_P35487786	221626190	221626249	chr1:221626190-221626249	1.00	1.01	-0.01
A_16_P35487790	221633825	221633884	chr1:221633825-221633884	0.67	1.06	-0.23
A_14_P100301	221646448	221646507	chr1:221646448-221646507	0.59	1.42	-0.22
A_16_P15468539	221653710	221653769	chr1:221653710-221653769	0.65	0.96	-0.24
A_16_P15468550	221661160	221661219	chr1:221661160-221661219	0.52	0.95	-0.27
A_16_P15468556	221676682	221676741	chr1:221676682-221676741	0.81	1.68	0.12
A_16_P35487874	221687187	221687246	chr1:221687187-221687246	0.44	1.66	-0.36
A_16_P15468590	221704017	221704076	chr1:221704017-221704076	0.74	1.38	-0.41
A_16_P15468592	221724697	221724756	chr1:221724697-221724756	0.63	0.80	-0.26
A_16_P15468609	221737494	221737553	CR936804	-0.01	0.35	-0.47
A_16_P35487947	221752978	221753037	CR936804	0.13	1.37	-0.44
A_16_P35487979	221766076	221766135	CR936804	0.78	0.93	-0.22
A_16_P00265245	221776615	221776662	CR936804	0.65	0.41	-0.11
A_16_P15468696	221784636	221784695	CR936804	0.59	1.30	-0.47
A_16_P00265249	221793893	221793952	CR936804	0.29	0.73	-0.51
A_16_P15468716	221799375	221799434	CR936804	0.59	1.00	-0.54
A_16_P15468730	221812776	221812835	CR936804	0.22	0.15	-0.35
A_16_P15468752	221819538	221819597	CR936804	0.35	0.23	-0.47
A_14_P104728	221833100	221833159	CR936804	0.60	1.15	-0.17
A_16_P15468825	221851918	221851977	CR936804	0.67	0.85	-0.34
A_16_P35488189	221861906	221861965	CR936804	1.04	0.60	-0.19
A_16_P35488210	221871533	221871592	CR936804	0.36	1.49	-0.47
A_16_P15468889	221877658	221877717	CR936804	0.82	0.47	-0.50
A_16_P15468894	221882946	221883005	CR936804	0.62	0.45	-0.31
A_16_P15468929	221896564	221896623	LBR	0.24	0.77	-0.30
A_14_P135836	221907880	221907939	LBR	-0.30	1.25	-0.29
A_14_P130714	221909704	221909763	LBR	0.69	0.80	-0.31
A_14_P125613	221923211	221923260	LBR	1.30	0.78	0.00
A_16_P00265464	221932917	221932976	chr1:221932917-221932976	0.80	0.84	-0.28
A_16_P15469066	221940365	221940424	chr1:221940365-221940424	0.97	1.56	0.10
A_16_P00265508	221959836	221959894	chr1:221959836-221959894	1.16	0.04	-0.01
A_16_P00265517	221965776	221965827	chr1:221965776-221965827	0.86	1.06	-0.05
A_16_P00265526	221971669	221971728	chr1:221971669-221971728	0.39	0.89	-0.24
A_16_P15469189	221987175	221987234	ENAH	0.71	1.50	-0.35
A_16_P00265580	221999425	221999484	ENAH	0.38	1.22	-0.19
A_16_P35488631	222007255	222007314	ENAH	0.95	1.18	-0.13

A_16_P15469295	222019223	222019282	ENAH	0.62	1.17	-0.29
A_14_P118546	222024967	222025026	ENAH	-0.11	2.79	-0.32
A_16_P35488730	222038023	222038082	ENAH	0.50	0.87	-0.57
A_16_P00265680	222049490	222049549	ENAH	0.70	0.81	-0.11
A_16_P35488787	222061629	222061688	ENAH	0.87	1.49	-0.40
A_16_P15469428	222071661	222071720	ENAH	0.62	0.96	-0.33
A_16_P15469448	222085283	222085342	ENAH	1.29	1.19	-0.10
A_16_P15469466	222091533	222091592	ENAH	0.63	0.92	-0.28
A_16_P15469476	222097615	222097674	ENAH	0.43	0.88	-0.27
A_16_P35488878	222104953	222105012	ENAH	0.29	0.56	-0.39
A_16_P15469493	222110931	222110990	ENAH	0.05	0.82	-0.45
A_16_P00265761	222122411	222122470	ENAH	0.77	0.95	-0.38
A_16_P15469563	222134834	222134893	ENAH	0.78	0.96	-0.29
A_16_P15469588	222144531	222144590	ENAH	0.60	0.59	-0.09
A_16_P15469620	222155273	222155332	chr1:222155273-222155332	1.21	0.94	-0.23
A_16_P15469648	222168273	222168332	chr1:222168273-222168332	0.83	1.00	-0.09
A_16_P15469666	222175787	222175846	chr1:222175787-222175846	0.87	-0.04	-0.09
A_16_P00265856	222184746	222184805	chr1:222184746-222184805	0.13	0.64	-0.26
A_16_P15469703	222190379	222190438	chr1:222190379-222190438	0.85	1.39	-0.27
A_16_P00265873	222198041	222198100	AK124056	0.54	0.73	-0.32
A_14_P137085	222203907	222203966	AK124056	1.31	1.22	0.00
A_16_P00265885	222216583	222216638	AK124056	1.19	0.69	-0.22
A_16_P15469758	222224963	222225022	AK124056	0.89	1.29	-0.27
A_16_P00265903	222233709	222233768	AK124056	1.14	1.15	-0.21
A_16_P15469808	222245915	222245974	AK124056	1.10	0.85	-0.26
A_16_P00265937	222261208	222261267	chr1:222261208-222261267	0.69	0.28	-0.33
A_16_P00265948	222265676	222265735	chr1:222265676-222265735	1.00	0.85	-0.33
A_16_P15469870	222270444	222270503	chr1:222270444-222270503	1.17	1.18	-0.40
A_16_P00265967	222275361	222275420	SRP9	0.92	0.71	-0.23
A_14_P101132	222284702	222284761	SRP9	-0.31	3.16	-0.86
A_16_P35489314	222298075	222298134	chr1:222298075-222298134	0.78	0.18	-0.23
A_16_P15469940	222310055	222310114	CR593306	1.13	1.49	-0.21
A_16_P15469954	222314965	222315024	CR593306	1.01	1.35	-0.20
A_14_P103570	222320472	222320529	EPHX1	0.13	0.45	-0.25
A_16_P00266023	222326351	222326410	EPHX1	1.18	0.75	-0.30
A_16_P15470001	222332264	222332323	EPHX1	0.81	1.49	-0.13
A_14_P138423	222339964	222340018	EPHX1	0.31	0.83	-0.17
A_16_P00266056	222346368	222346422	TMEM63A	1.00	1.13	-0.21
A_16_P00266066	222351689	222351748	TMEM63A	0.72	0.39	-0.37
A_16_P00266084	222360766	222360825	TMEM63A	0.77	0.85	-0.28
A_16_P15470119	222368806	222368865	TMEM63A	1.07	0.24	-0.20
A_16_P00266111	222374303	222374361	TMEM63A	1.18	0.85	-0.05
A_14_P133987	222382826	222382879	LEFTY1	1.06	1.27	0.00
A_16_P15470175	222387642	222387701	chr1:222387642-222387701	0.66	0.87	-0.56
A_16_P35489608	222392436	222392495	chr1:222392436-222392495	0.49	-2.19	-0.39
A_16_P00266142	222401279	222401338	chr1:222401279-222401338	1.01	1.09	-0.10
A_16_P00266159	222409363	222409422	chr1:222409363-222409422	0.61	1.55	-0.35
A_14_P104424	222416967	222417020	PYCR2	-0.42	0.78	-0.54

A_16_P15470262	222422514	222422573	chr1:222422514-222422573	0.97	1.57	-0.67
A_16_P00266184	222426362	222426417	chr1:222426362-222426417	0.66	0.49	-0.20
A_16_P15470271	222429630	222429688	chr1:222429630-222429688	0.69	1.02	-0.17
A_14_P114781	222433606	222433657	LEFTY2	1.68	0.64	0.09
A_16_P35489736	222440497	222440556	chr1:222440497-222440556	1.16	1.14	-0.14
A_16_P00266211	222448432	222448491	chr1:222448432-222448491	0.67	0.78	-0.48
A_16_P00266225	222456704	222456759	chr1:222456704-222456759	1.09	1.52	-0.09
A_16_P15470358	222462213	222462272	chr1:222462213-222462272	0.41	0.67	-0.49
A_16_P15470377	222469871	222469930	chr1:222469871-222469930	0.51	1.08	-0.29
A_16_P15470384	222474377	222474436	chr1:222474377-222474436	0.40	1.29	-0.14
A_14_P116712	222479642	222479701	C1orf55	-0.18	2.10	0.48
A_16_P00266270	222489779	222489838	C1orf55	0.81	0.73	-0.32
A_16_P15470446	222497870	222497929	chr1:222497870-222497929	0.37	1.16	-0.14
A_16_P15470456	222502684	222502743	chr1:222502684-222502743	0.08	0.90	-0.33
A_16_P15470491	222517002	222517061	chr1:222517002-222517061	0.73	1.08	-0.10
A_16_P15470519	222527174	222527233	chr1:222527174-222527233	0.68	0.98	-0.24
A_16_P00266344	222543619	222543678	chr1:222543619-222543678	1.06	1.00	0.04
A_16_P00266353	222549007	222549066	chr1:222549007-222549066	0.87	1.49	-0.13
A_14_P138929	222558577	222558636	H3F3A	0.48	1.73	-0.20
A_16_P35490062	222566910	222566969	chr1:222566910-222566969	0.48	1.70	-0.37
A_16_P35490074	222577033	222577092	chr1:222577033-222577092	0.94	0.21	-0.17
A_16_P35490085	222585009	222585068	chr1:222585009-222585068	0.78	0.58	-0.34
A_16_P35490105	222594391	222594450	chr1:222594391-222594450	0.93	1.24	-0.76
A_16_P15470694	222615436	222615495	chr1:222615436-222615495	0.62	0.53	-0.35
A_16_P00266436	222626684	222626734	chr1:222626684-222626734	1.21	0.22	0.04
A_16_P35490191	222639325	222639384	ACBD3	0.48	1.01	-0.19
A_16_P35490236	222653536	222653595	ACBD3	0.58	1.19	-0.26
A_14_P101169	222659341	222659390	ACBD3	0.47	0.84	0.64
A_16_P15470817	222664981	222665040	ACBD3	0.56	0.87	-0.01
A_16_P35490287	222671964	222672023	ACBD3	0.88	1.48	-0.09
A_16_P15470846	222679560	222679619	ACBD3	0.36	1.42	-0.21
A_14_P118904	222685975	222686034	BC033346	0.67	1.12	-0.03
A_16_P15470881	222696394	222696453	chr1:222696394-222696453	0.67	0.27	-0.03
A_16_P15470893	222703585	222703644	chr1:222703585-222703644	0.72	0.09	-0.17
A_16_P15470907	222707736	222707795	chr1:222707736-222707795	0.17	0.98	-0.05
A_16_P35490379	222712731	222712790	chr1:222712731-222712790	0.78	1.29	-0.09
A_14_P103862	222718789	222718848	MIXL1	0.88	1.19	0.00
A_16_P35490423	222727468	222727527	LIN9	0.21	0.35	-0.33
A_16_P00266583	222733379	222733438	LIN9	-0.25	0.79	-0.63
A_14_P122482	222745264	222745323	LIN9	0.33	1.39	-0.23
A_16_P15471003	222759917	222759976	LIN9	0.62	0.85	-0.19
A_14_P112823	222772316	222772375	LIN9	0.64	0.74	-0.46
A_16_P35490515	222780826	222780885	LIN9	0.26	1.03	-0.69
A_14_P126247	222795606	222795665	LIN9	0.25	0.51	-0.39
A_16_P35490569	222804472	222804531	chr1:222804472-222804531	0.61	0.96	-0.27
A_16_P15471117	222817111	222817170	chr1:222817111-222817170	0.59	0.59	-0.05
A_16_P35490606	222830201	222830260	chr1:222830201-222830260	0.90	0.86	-0.25
A_14_P116284	222848186	222848245	chr1:222848186-222848245	1.28	1.61	0.03

A_16_P00266702	222855713	222855772	PARP1	0.66	1.05	-0.23
A_14_P109774	222868634	222868693	PARP1	0.90	0.89	-0.15
A_14_P120707	222875449	222875508	PARP1	1.32	1.58	-0.23
A_14_P130390	222891364	222891423	PARP1	0.65	0.91	-0.13
A_14_P130390	222891364	222891423	PARP1	0.63	0.97	-0.21
A_14_P130390	222891364	222891423	PARP1	0.75	0.85	-0.27
A_16_P00266778	222898559	222898618	PARP1	0.84	1.21	-0.29
A_16_P15471348	222912268	222912327	chr1:222912268-222912327	0.31	1.24	-0.36
A_16_P15471381	222925948	222926007	chr1:222925948-222926007	0.92	1.30	-0.04
A_16_P00266851	222949229	222949288	chr1:222949229-222949288	0.66	1.16	-0.19
A_14_P110905	222984518	222984577	chr1:222984518-222984577	0.96	0.60	-0.19
A_16_P15471551	223010541	223010600	chr1:223010541-223010600	0.73	0.73	-0.32
A_16_P15471591	223024836	223024895	chr1:223024836-223024895	1.08	1.30	-0.21
A_16_P15471609	223035726	223035785	AK055856	-0.10	0.34	-0.01
A_16_P00266960	223045405	223045464	C1orf95	0.59	0.77	-0.34
A_16_P00266976	223055192	223055251	C1orf95	0.64	0.56	-0.29
A_16_P35491165	223061166	223061225	C1orf95	0.83	1.23	-0.25
A_16_P00267004	223070828	223070887	C1orf95	1.30	-0.40	-0.26
A_16_P15471717	223077806	223077865	C1orf95	0.65	1.21	-0.13
A_16_P15471745	223085518	223085577	C1orf95	0.54	0.65	-0.13
A_16_P35491250	223091706	223091765	C1orf95	0.73	1.08	-0.19
A_16_P00267059	223098995	223099052	C1orf95	1.08	0.42	-0.01
A_16_P00267080	223113337	223113396	chr1:223113337-223113396	0.51	1.27	-0.18
A_14_P133622	223128936	223128995	ITPKB	1.29	1.47	-0.28
A_16_P15471875	223134569	223134628	ITPKB	0.54	1.01	-0.45
A_16_P00267138	223144695	223144754	ITPKB	1.11	0.30	-0.19
A_16_P15471937	223151641	223151700	ITPKB	1.23	0.74	-0.19
A_16_P15471983	223165396	223165455	ITPKB	0.92	0.87	-0.30
A_14_P107068	223178337	223178396	ITPKB	0.83	1.83	-0.08
A_16_P15472049	223186020	223186079	ITPKB	1.40	0.75	-0.07
A_16_P00267232	223191471	223191530	ITPKB	0.86	1.15	-0.18
A_16_P15472087	223198574	223198633	ITPKB	0.71	0.35	-0.43
A_16_P15472123	223209964	223210023	ITPKB	0.75	1.70	-0.25
A_16_P00267279	223216968	223217027	ITPKB	1.15	1.49	-0.24
A_14_P116758	223222668	223222727	ITPKB	0.49	1.47	-0.21
A_16_P15472204	223234900	223234959	chr1:223234900-223234959	0.98	0.02	-0.19
A_16_P15472212	223240949	223241008	chr1:223240949-223241008	0.32	0.12	-0.50
A_16_P35491730	223255965	223256024	chr1:223255965-223256024	0.36	0.85	-0.31
A_16_P15472290	223281986	223282045	chr1:223281986-223282045	1.14	1.07	0.06
A_14_P138248	223316345	223316404	chr1:223316345-223316404	0.19	1.14	-0.47
A_16_P35491916	223334855	223334914	chr1:223334855-223334914	1.11	1.61	-0.25
A_16_P00267445	223356995	223357054	chr1:223356995-223357054	0.88	1.33	-0.03
A_16_P35491943	223365938	223365997	PSEN2	1.44	1.56	-0.25
A_14_P136184	223375097	223375141	PSEN2	0.87	0.56	0.05
A_14_P129743	223377828	223377887	PSEN2	1.17	0.90	-0.16
A_16_P35492032	223387855	223387914	PSEN2	0.65	1.24	-0.32
A_16_P15472548	223397554	223397613	AK123980	0.99	1.31	-0.34
A_16_P35492094	223409682	223409741	AK126466	0.43	0.47	-0.23

A_16_P35492102	223415431	223415490	AK126466	0.94	0.62	-0.09
A_16_P15472608	223428327	223428386	AK126466	-0.01	0.69	-0.45
A_16_P00267557	223437290	223437349	CABC1	0.63	0.64	-0.26
A_14_P102341	223443324	223443383	CABC1	0.61	0.49	0.05
A_16_P00267584	223453535	223453594	CABC1	1.02	1.49	-0.10
A_14_P127632	223461436	223461495	CABC1	0.65	1.54	-0.22
A_16_P35492236	223467240	223467299	CABC1	0.96	1.12	-0.48
A_14_P124660	223479316	223479361	CABC1	1.09	1.41	-0.09
A_14_P104189	223487412	223487471	CDC42BPA	0.89	0.54	-0.22
A_16_P35492355	223501621	223501680	CDC42BPA	0.50	1.49	-0.51
A_16_P00267684	223511392	223511451	CDC42BPA	0.41	0.70	-0.48
A_16_P35492423	223523248	223523307	CDC42BPA	1.19	1.33	-0.32
A_16_P15472911	223529874	223529933	CDC42BPA	0.68	0.68	-0.27
A_16_P15472926	223536218	223536277	CDC42BPA	0.61	0.87	-0.16
A_16_P15472947	223546586	223546645	CDC42BPA	0.78	0.94	-0.36
A_16_P00267764	223555968	223556027	CDC42BPA	1.04	0.93	-0.04
A_16_P35492547	223562355	223562414	CDC42BPA	0.29	1.62	-0.41
A_16_P15473035	223571308	223571367	CDC42BPA	0.67	0.56	-0.49
A_16_P00267816	223583996	223584055	CDC42BPA	0.34	1.73	-0.62
A_16_P35492628	223589818	223589877	CDC42BPA	0.39	0.61	-0.23
A_16_P15473085	223595593	223595652	CDC42BPA	0.44	1.99	-0.30
A_16_P15473106	223607059	223607117	CDC42BPA	-0.05	1.02	-0.31
A_16_P15473123	223614278	223614337	CDC42BPA	-0.08	0.59	-0.06
A_16_P15473143	223621921	223621980	CDC42BPA	0.23	-0.08	-0.28
A_14_P104072	223637384	223637443	CDC42BPA	0.71	0.01	1.03
A_16_P00267885	223645330	223645389	CDC42BPA	0.68	1.61	-0.27
A_16_P35492753	223654933	223654992	CDC42BPA	0.66	0.94	-0.51
A_16_P35492768	223661225	223661284	CDC42BPA	0.99	0.81	-0.13
A_16_P15473230	223667561	223667620	CDC42BPA	0.50	0.73	-0.31
A_16_P15473244	223675413	223675472	CDC42BPA	0.28	0.67	-0.53
A_16_P35492808	223685098	223685157	CDC42BPA	0.63	1.35	-0.38
A_14_P110921	223694001	223694060	CDC42BPA	0.64	1.87	-0.24
A_16_P35492882	223711265	223711324	CDC42BPA	0.46	-0.52	-0.68
A_16_P00267984	223720742	223720801	CDC42BPA	1.01	0.89	0.03
A_16_P15473371	223730742	223730801	CDC42BPA	0.13	0.38	-0.39
A_16_P15473375	223737774	223737833	CDC42BPA	-0.11	0.71	-0.41
A_16_P15473377	223745795	223745854	CDC42BPA	0.66	1.43	-0.47
A_16_P15473395	223754687	223754746	CDC42BPA	0.64	0.70	-0.29
A_16_P15473414	223761097	223761156	CDC42BPA	0.32	0.92	-0.40
A_16_P15473437	223769447	223769506	CDC42BPA	-0.48	1.20	-2.33
A_16_P15473467	223780019	223780078	CDC42BPA	1.09	1.01	-0.42
A_16_P00268056	223785466	223785525	CDC42BPA	0.85	0.45	-0.23
A_16_P00268065	223793191	223793250	CDC42BPA	0.34	1.00	-0.48
A_16_P15473517	223803691	223803750	CDC42BPA	0.66	1.00	-0.31
A_14_P102549	223811433	223811492	CDC42BPA	0.29	1.36	-0.11
A_16_P15473560	223817447	223817506	chr1:223817447-223817506	0.48	1.18	-0.34
A_16_P15473586	223827724	223827783	chr1:223827724-223827783	0.03	0.18	-0.17
A_16_P15473596	223837240	223837299	chr1:223837240-223837299	0.72	1.13	-0.10

A_16_P00268142	223844906	223844965	chr1:223844906-223844965	0.28	0.32	-0.58
A_16_P00268154	223855661	223855720	chr1:223855661-223855720	0.67	1.02	-0.23
A_16_P35493205	223861485	223861544	chr1:223861485-223861544	0.63	0.92	-0.35
A_16_P15473672	223872401	223872460	chr1:223872401-223872460	1.12	1.61	-0.20
A_16_P35493233	223883501	223883560	chr1:223883501-223883560	0.93	0.70	-0.23
A_16_P15473696	223892453	223892512	BC039356	0.45	1.57	-0.34
A_16_P00268190	223905715	223905773	BC039356	1.69	0.50	-0.72
A_16_P15473734	223916997	223917056	BC039356	0.80	0.96	-0.16
A_14_P106483	223924596	223924655	BC039356	1.12	1.16	-0.32
A_16_P35493334	223930988	223931047	chr1:223930988-223931047	0.96	0.79	-0.17
A_16_P00268248	223937304	223937363	chr1:223937304-223937363	0.52	0.87	-0.36
A_16_P00268258	223945609	223945668	chr1:223945609-223945668	0.94	1.27	-0.25
A_16_P15473833	223956168	223956227	chr1:223956168-223956227	0.59	0.77	-0.19
A_16_P15473859	223964184	223964243	chr1:223964184-223964243	0.72	0.40	0.04
A_16_P35493430	223976269	223976328	chr1:223976269-223976328	0.56	0.71	-0.25
A_16_P00268296	223984438	223984497	chr1:223984438-223984497	0.87	1.32	-0.38
A_16_P00268303	223993634	223993693	chr1:223993634-223993693	0.91	0.63	0.00
A_16_P00268315	224008749	224008808	chr1:224008749-224008808	0.18	0.96	-0.22
A_16_P00268324	224018993	224019052	chr1:224018993-224019052	0.52	0.76	-0.02
A_16_P35493517	224027773	224027832	chr1:224027773-224027832	0.29	1.12	-0.35
A_16_P35493535	224033632	224033691	chr1:224033632-224033691	0.54	0.73	-0.58
A_16_P35493567	224043834	224043893	chr1:224043834-224043893	0.82	1.21	-0.24
A_16_P00268354	224050448	224050507	chr1:224050448-224050507	0.35	1.96	-0.31
A_16_P15474035	224061364	224061423	ZNF678	0.49	1.27	-0.30
A_16_P15474047	224067995	224068054	ZNF678	0.04	0.99	-0.29
A_14_P104450	224074751	224074810	ZNF678	0.34	1.04	-0.09
A_16_P15474066	224091482	224091541	ZNF678	0.12	0.53	-0.26
A_16_P35493638	224097011	224097070	ZNF678	0.17	1.26	-0.38
A_16_P00268407	224108032	224108091	ZNF678	1.09	0.89	-0.27
A_14_P127623	224117006	224117065	ZNF678	0.49	0.83	0.05
A_16_P35493689	224126341	224126397	ZNF678	0.34	0.71	-0.28
A_14_P109646	224140920	224140974	ZNF678	0.60	1.54	-0.03
A_14_P200052	224141130	224141189	ZNF678	0.84	1.62	-0.30
A_16_P00268437	224148939	224148998	ZNF678	0.80	0.57	-0.19
A_14_P106480	224155880	224155939	BC007286	0.97	0.89	-0.23
A_16_P35493775	224166147	224166206	chr1:224166147-224166206	-0.06	1.13	1.19
A_16_P35493792	224185601	224185660	chr1:224185601-224185660	1.19	0.88	-0.15
A_16_P35493819	224192477	224192536	chr1:224192477-224192536	0.49	0.18	-0.43
A_14_P129898	224201162	224201221	chr1:224201162-224201221	0.74	0.92	-0.07
A_16_P15474271	224207206	224207265	chr1:224207206-224207265	0.94	1.37	-0.18
A_16_P00268503	224219250	224219309	chr1:224219250-224219309	1.11	1.18	-0.36
A_14_P108070	224227419	224227463	JMJD4	0.93	0.94	0.08
A_14_P119767	224229151	224229199	JMJD4	0.03	1.77	-0.29
A_14_P114678	224233068	224233127	C1orf142	0.75	0.99	-0.34
A_16_P00268529	224237250	224237306	C1orf142	1.12	0.62	-0.28
A_14_P127209	224246076	224246135	C1orf142	1.31	0.34	-0.37
A_16_P35493963	224255808	224255867	C1orf142	0.97	0.39	-0.19
A_16_P35493990	224263835	224263894	C1orf142	0.40	0.65	-0.15

A_14_P134195	224270750	224270809	C1orf142	1.13	1.11	0.01
A_16_P00268592	224276585	224276644	chr1:224276585-224276644	0.79	1.55	-0.12
A_16_P00268605	224285323	224285382	chr1:224285323-224285382	1.06	0.81	-0.15
A_16_P00268618	224292481	224292540	chr1:224292481-224292540	1.10	1.08	-0.33
A_16_P00268620	224300454	224300513	chr1:224300454-224300513	1.02	1.39	-0.25
A_16_P35494095	224313550	224313609	MPN2	0.57	1.53	-0.22
A_16_P15474506	224331148	224331207	MPN2	0.09	1.34	-0.23
A_14_P138727	224333040	224333099	MPN2	0.23	1.01	-0.30
A_16_P15474535	224341279	224341338	chr1:224341279-224341338	0.89	1.37	-0.16
A_16_P00268649	224347414	224347473	chr1:224347414-224347473	0.87	1.08	-0.16
A_16_P00268656	224353784	224353843	chr1:224353784-224353843	0.88	0.84	-0.09
A_16_P15474574	224374107	224374166	chr1:224374107-224374166	0.51	0.98	-0.38
A_16_P00268683	224386550	224386601	chr1:224386550-224386601	0.17	0.55	-0.42
A_16_P00268720	224407664	224407723	chr1:224407664-224407723	0.49	1.59	0.07
A_16_P00268744	224417981	224418034	WNT9A	0.99	0.51	-0.06
A_14_P127657	224430231	224430290	WNT9A	0.67	1.10	-0.22
A_16_P15474765	224439275	224439321	WNT9A	0.88	0.39	-0.02
A_16_P00268789	224447651	224447710	chr1:224447651-224447710	0.60	1.40	-0.42
A_16_P35494397	224455039	224455098	chr1:224455039-224455098	0.71	1.64	-0.19
A_16_P00268797	224461493	224461537	chr1:224461493-224461537	0.42	0.95	0.26
A_16_P35494464	224480359	224480418	chr1:224480359-224480418	-0.09	1.05	-0.75
A_16_P15474883	224494503	224494562	chr1:224494503-224494562	1.01	0.49	-0.23
A_14_P106999	224513834	224513893	WNT3A	0.61	0.85	-0.23
A_16_P00268855	224530526	224530580	WNT3A	0.68	0.15	-0.11
A_14_P110739	224535341	224535400	WNT3A	0.40	0.70	-0.28
A_16_P00268878	224547114	224547173	WNT3A	0.89	0.71	-0.29
A_14_P122078	224555630	224555689	WNT3A	0.79	1.18	0.02
A_16_P00268897	224561211	224561267	chr1:224561211-224561267	1.21	1.31	-0.17
A_14_P108890	224569676	224569722	AL137472	0.89	1.51	0.12
A_14_P111813	224578716	224578775	ARF1	0.64	0.70	-0.29
A_16_P00268934	224582303	224582362	ARF1	-0.33	0.91	-0.32
A_14_P124155	224586284	224586343	ARF1	0.37	2.95	0.33
A_16_P00268951	224590344	224590403	ARF1	0.89	0.96	-0.10
A_14_P115866	224596008	224596067	C1orf35	0.94	0.52	-0.06
A_14_P130561	224601127	224601175	MRPL55	0.28	0.62	0.20
A_16_P00268981	224609357	224609416	chr1:224609357-224609416	-0.48	0.00	-0.17
A_16_P15475209	224616214	224616273	AK094778	0.75	0.68	-0.22
A_16_P00268986	224621102	224621161	chr1:224621102-224621161	0.59	0.52	-0.23
A_16_P00268989	224626062	224626121	chr1:224626062-224626121	0.61	1.11	-0.19
A_16_P15475230	224633227	224633286	chr1:224633227-224633286	0.61	0.74	-0.39
A_14_P107324	224639354	224639413	GUK1	0.74	0.89	-0.38
A_14_P114850	224643340	224643398	GUK1	0.92	0.70	-0.09
A_16_P15475281	224647612	224647666	GJA12	0.31	1.43	-0.10
A_14_P120733	224654202	224654258	GJA12	1.29	0.21	-0.02
A_16_P15475312	224658609	224658660	AK057440	1.19	1.24	-0.16
A_16_P15475325	224663767	224663826	C1orf69	0.73	1.35	-0.25
A_16_P00269054	224673140	224673199	C1orf69	0.70	0.02	-0.08
A_16_P00269057	224684220	224684274	chr1:224684220-224684274	1.21	0.41	-0.09

A_16_P15475365	224690273	224690332	chr1:224690273-224690332	0.51	1.33	-0.26
A_16_P00269065	224694925	224694984	chr1:224694925-224694984	0.96	0.24	-0.10
A_16_P00269072	224698887	224698946	C1orf145	0.15	1.70	-0.15
A_16_P15475403	224704645	224704689	C1orf145	0.74	0.46	-0.05
A_16_P15475429	224712357	224712416	OBSCN	0.26	1.29	-0.24
A_14_P124964	224716912	224716971	OBSCN	0.69	0.82	-0.16
A_16_P00269111	224722412	224722471	OBSCN	1.27	1.25	-0.12
A_16_P15475469	224737109	224737168	OBSCN	0.83	1.75	-0.21
A_16_P15475490	224747662	224747721	OBSCN	0.64	1.04	-0.17
A_16_P15475510	224755338	224755397	OBSCN	0.88	0.77	-0.24
A_14_P104315	224762079	224762138	OBSCN	0.44	0.78	-0.35
A_16_P00269154	224769461	224769509	OBSCN	0.41	0.61	-0.04
A_14_P128270	224779992	224780050	OBSCN	0.57	0.83	-0.38
A_16_P15475614	224787720	224787779	OBSCN	0.38	-0.40	-0.40
A_16_P00269223	224804435	224804494	OBSCN	0.82	0.86	-0.12
A_14_P105271	224817276	224817335	OBSCN	1.18	1.89	-0.25
A_14_P105271	224817276	224817335	OBSCN	1.19	1.87	-0.21
A_14_P105271	224817276	224817335	OBSCN	1.16	1.97	-0.31
A_16_P00269253	224828518	224828577	OBSCN	0.55	1.46	-0.58
A_16_P15475761	224843427	224843486	OBSCN	1.31	1.60	0.17
A_16_P35495496	224851740	224851794	OBSCN	1.04	1.36	-0.08
A_16_P35495496	224851740	224851794	OBSCN	1.10	0.74	-0.10
A_16_P35495496	224851740	224851794	OBSCN	0.98	1.33	-0.14
A_16_P00269311	224861413	224861462	AB046859	1.05	0.86	-0.22
A_16_P00269328	224870818	224870867	AB046859	1.07	1.35	-0.25
A_16_P35495611	224879345	224879404	chr1:224879345-224879404	0.24	1.13	-0.53
A_14_P135284	224888129	224888188	TRIM11	0.45	1.11	-0.17
A_16_P00269358	224895139	224895198	TRIM11	0.52	0.82	-0.17
A_16_P00269365	224898391	224898448	TRIM11	0.35	0.78	-0.30
A_14_P112621	224902480	224902539	TRIM17	0.68	1.23	-0.23
A_14_P127889	224909646	224909699	TRIM17	1.48	1.11	0.16
A_16_P15475963	224914214	224914273	chr1:224914214-224914273	0.56	0.98	-0.44
A_16_P35495718	224917590	224917649	chr1:224917590-224917649	0.99	1.79	-0.22
A_16_P15475987	224925165	224925224	chr1:224925165-224925224	0.63	1.09	-0.07
A_16_P00269405	224930094	224930153	chr1:224930094-224930153	0.95	0.52	-0.40
A_16_P00269411	224936992	224937051	chr1:224936992-224937051	1.14	1.26	-0.17
A_16_P15476026	224945306	224945365	chr1:224945306-224945365	0.62	-0.18	-0.11
A_14_P131868	224951501	224951560	BC001193	0.48	0.76	-0.28
A_14_P116173	224951781	224951830	HIST3H2A	0.60	-1.49	0.02
A_14_P128402	224952998	224953050	AK091220	1.50	0.70	0.05
A_16_P15476069	224959526	224959585	chr1:224959526-224959585	0.72	0.15	-0.37
A_16_P00269454	224966996	224967055	chr1:224966996-224967055	0.97	1.30	-0.19
A_14_P107356	224978969	224979028	chr1:224978969-224979028	0.56	-0.63	-0.33
A_16_P00269481	224988438	224988488	BC012758	1.37	0.83	-0.15
A_16_P00269490	224999307	224999366	chr1:224999307-224999366	1.19	0.81	-0.19
A_16_P00269505	225011345	225011404	chr1:225011345-225011404	1.14	1.80	-0.12
A_16_P15476199	225023450	225023495	chr1:225023450-225023495	0.66	0.72	0.38
A_16_P00269512	225050719	225050768	chr1:225050719-225050768	1.28	1.71	-0.65

A_16_P35496088	225090580	225090624	AK092260	1.11	0.48	0.03
A_14_P115799	225094475	225094534	AK092260	0.85	1.16	-0.02
A_16_P15476371	225112915	225112974	chr1:225112915-225112974	0.74	1.47	-0.28
A_14_P112627	225132511	225132570	chr1:225132511-225132570	0.39	0.69	-0.18
A_16_P15476410	225136505	225136564	chr1:225136505-225136564	0.47	0.40	-0.04
A_16_P15476420	225155458	225155517	chr1:225155458-225155517	0.13	0.96	-0.37
A_16_P15476444	225164557	225164616	chr1:225164557-225164616	0.43	0.62	-0.30
A_16_P15476453	225169563	225169622	chr1:225169563-225169622	-0.10	0.81	-0.53
A_16_P35496214	225174410	225174469	chr1:225174410-225174469	0.56	1.19	-0.35
A_16_P35496227	225179269	225179328	RHOU	0.33	0.08	-0.54
A_14_P130411	225184524	225184583	RHOU	0.37	0.59	-0.32
A_14_P201415	225186793	225186852	RHOU	1.39	1.56	0.06
A_16_P15476529	225194238	225194296	chr1:225194238-225194296	1.12	1.50	-0.08
A_16_P00269633	225201448	225201507	chr1:225201448-225201507	1.12	0.92	-0.14
A_16_P35496341	225213456	225213515	chr1:225213456-225213515	1.49	1.63	0.18
A_16_P00269691	225237271	225237330	chr1:225237271-225237330	0.91	1.74	-0.16
A_16_P15476730	225271599	225271658	chr1:225271599-225271658	0.37	1.07	-0.65
A_16_P35496522	225286953	225287012	chr1:225286953-225287012	0.45	0.62	-0.69
A_16_P00269778	225304564	225304623	chr1:225304564-225304623	0.75	1.45	-0.17
A_16_P00269808	225319563	225319622	chr1:225319563-225319622	1.07	0.69	-0.32
A_16_P00269830	225336944	225337002	AL832503	0.29	-0.14	-0.20
A_16_P35496684	225351694	225351753	AL832503	0.63	0.70	-0.70
A_14_P117384	225375223	225375282	AL832503	0.66	1.06	-0.31
A_16_P15477048	225389006	225389065	AL832503	0.56	1.08	-0.42
A_16_P00269954	225429303	225429362	chr1:225429303-225429362	0.21	-0.35	-0.27
A_16_P00270011	225465391	225465450	chr1:225465391-225465450	0.98	0.69	-0.20
A_14_P114999	225519102	225519161	chr1:225519102-225519161	0.42	1.48	-0.33
A_16_P00270137	225574639	225574698	chr1:225574639-225574698	0.91	1.40	-0.39
A_16_P00270174	225602412	225602471	chr1:225602412-225602471	1.24	1.43	-0.44
A_16_P00270207	225625196	225625255	chr1:225625196-225625255	0.83	1.21	-0.29
A_14_P118718	225645712	225645771	chr1:225645712-225645771	-0.02	1.39	-0.51
A_16_P15477681	225663452	225663511	chr1:225663452-225663511	0.57	1.46	-0.17
A_16_P35497474	225673382	225673441	chr1:225673382-225673441	0.93	0.84	-0.27
A_16_P15477729	225683857	225683916	chr1:225683857-225683916	0.83	1.09	-0.22
A_16_P00270281	225691758	225691817	chr1:225691758-225691817	1.05	0.95	-0.02
A_16_P00270293	225701744	225701803	chr1:225701744-225701803	0.99	0.22	-0.15
A_16_P15477768	225710909	225710967	chr1:225710909-225710967	1.03	0.56	-0.30
A_16_P00270309	225717046	225717105	RAB4A	0.82	0.55	-0.35
A_16_P15477804	225722753	225722811	RAB4A	0.92	0.41	-0.33
A_14_P125512	225731264	225731323	RAB4A	0.61	1.41	-0.07
A_16_P35497622	225741544	225741603	RAB4A	0.24	0.04	-0.16
A_16_P35497631	225746758	225746817	RAB4A	0.57	0.74	-0.38
A_16_P15477888	225756544	225756603	chr1:225756544-225756603	0.28	1.12	-0.42
A_16_P35497682	225766572	225766631	C1orf96	0.36	0.55	-0.43
A_14_P132860	225773601	225773660	C1orf96	-0.02	0.59	-0.17
A_14_P119935	225784048	225784107	C1orf96	1.15	1.23	-0.16
A_16_P35497745	225794333	225794392	chr1:225794333-225794392	1.28	0.92	-0.17
A_16_P00270425	225808991	225809050	chr1:225808991-225809050	0.51	0.59	-0.34

A_14_P139476	225831552	225831611	chr1:225831552-225831611	0.84	0.94	-0.18
A_16_P00270476	225851807	225851865	chr1:225851807-225851865	0.29	1.22	-0.27
A_16_P00270490	225865533	225865592	chr1:225865533-225865592	0.74	0.88	-0.30
A_14_P118185	225873736	225873795	ACTA1	0.49	0.23	-0.29
A_14_P121813	225873837	225873894	ACTA1	-0.25	0.44	0.04
A_16_P15478145	225878122	225878181	chr1:225878122-225878181	0.71	0.78	-0.44
A_16_P35497951	225887368	225887427	NUP133	0.75	0.40	-0.30
A_16_P15478184	225892748	225892807	NUP133	0.54	0.89	-0.30
A_16_P35497983	225900588	225900647	NUP133	0.43	0.93	-0.43
A_14_P123841	225907125	225907184	NUP133	0.32	0.42	-0.50
A_16_P15478233	225912835	225912894	NUP133	0.91	0.99	-0.06
A_16_P35498048	225920251	225920310	NUP133	0.57	1.17	-0.21
A_14_P137787	225926558	225926617	NUP133	0.55	1.34	-0.33
A_16_P15478289	225932441	225932500	NUP133	0.89	0.89	-0.21
A_16_P35498095	225937938	225937997	NUP133	0.50	1.01	-0.41
A_16_P00270633	225948546	225948605	NUP133	1.00	0.62	-0.42
A_16_P15478353	225957650	225957709	chr1:225957650-225957709	0.99	1.04	-0.13
A_14_P111764	225968897	225968956	ABCB10	0.88	1.36	-0.25
A_16_P15478415	225983014	225983073	ABCB10	1.05	0.83	-0.06
A_14_P106164	225996840	225996899	ABCB10	-0.05	0.79	-0.41
A_16_P35498292	226007069	226007128	chr1:226007069-226007128	0.97	1.14	-0.20
A_14_P103179	226020504	226020563	chr1:226020504-226020563	1.21	1.30	-0.28
A_16_P00270747	226036258	226036317	TAF5L	0.96	1.42	-0.18
A_14_P107362	226043570	226043629	TAF5L	0.37	1.60	-0.32
A_16_P00270779	226052323	226052381	TAF5L	0.26	0.36	-0.32
A_16_P35498420	226057129	226057188	TAF5L	0.68	0.36	-0.32
A_14_P103952	226067235	226067294	TAF5L	0.52	0.59	-0.12
A_16_P35498475	226074830	226074889	KIAA0133	0.46	1.39	-0.38
A_14_P138438	226085998	226086049	KIAA0133	0.59	0.29	-0.46
A_16_P15478716	226092498	226092557	KIAA0133	0.82	1.60	-0.37
A_14_P202348	226102025	226102084	KIAA0133	0.82	1.38	-0.24
A_16_P15478778	226111691	226111750	chr1:226111691-226111750	0.63	0.30	-0.02
A_16_P00270902	226122199	226122258	chr1:226122199-226122258	0.65	0.98	0.02
A_16_P15478849	226143555	226143614	chr1:226143555-226143614	0.56	1.36	-0.33
A_16_P35498716	226174144	226174203	chr1:226174144-226174203	1.31	1.10	-0.08
A_14_P136139	226217596	226217655	chr1:226217596-226217655	0.80	0.95	-0.57
A_16_P35498886	226257223	226257282	chr1:226257223-226257282	0.66	1.48	-0.41
A_16_P15479138	226290591	226290650	chr1:226290591-226290650	0.88	0.95	-0.51
A_16_P15479241	226326287	226326346	chr1:226326287-226326346	0.74	1.47	-0.30
A_16_P35499127	226365358	226365417	chr1:226365358-226365417	0.81	0.42	-0.14
A_16_P15479411	226418504	226418563	chr1:226418504-226418563	0.50	0.57	-0.25
A_14_P122102	226448377	226448436	chr1:226448377-226448436	1.15	1.49	-0.22
A_16_P00271304	226471542	226471601	chr1:226471542-226471601	1.12	0.40	-0.39
A_16_P15479554	226486073	226486132	chr1:226486073-226486132	0.76	0.80	-0.02
A_16_P00271329	226497588	226497647	chr1:226497588-226497647	0.39	1.80	-0.03
A_16_P15479601	226506088	226506147	chr1:226506088-226506147	-0.18	1.30	-0.24
A_16_P00271347	226511862	226511921	GALNT2	0.18	0.92	-0.52
A_16_P00271360	226518416	226518474	GALNT2	0.86	0.77	-0.23

A_16_P15479653	226524184	226524243	GALNT2	1.30	1.08	-0.32
A_16_P35499518	226540542	226540601	GALNT2	0.49	1.06	-0.26
A_16_P00271413	226548753	226548812	GALNT2	1.16	0.93	-0.21
A_14_P119766	226556378	226556437	GALNT2	0.66	0.28	-0.27
A_16_P15479757	226563444	226563503	GALNT2	0.72	0.90	-0.33
A_16_P00271451	226571585	226571644	GALNT2	0.88	1.07	-0.23
A_16_P35499631	226580407	226580466	GALNT2	0.58	1.04	-0.40
A_16_P15479833	226586466	226586525	GALNT2	0.76	0.97	-0.22
A_14_P128526	226592172	226592231	GALNT2	0.98	0.48	-0.18
A_16_P00271504	226599448	226599507	GALNT2	0.87	1.23	-0.18
A_16_P15479921	226613962	226614021	GALNT2	0.32	0.26	-0.44
A_14_P103305	226620703	226620759	GALNT2	0.66	0.94	-0.38
A_16_P15479954	226631886	226631945	GALNT2	0.44	1.20	-0.09
A_14_P127128	226638936	226638995	GALNT2	0.61	1.19	0.30
A_16_P35499821	226647034	226647093	GALNT2	0.46	0.39	-0.42
A_16_P35499840	226653361	226653420	GALNT2	0.53	0.67	-0.34
A_16_P15480035	226661611	226661670	GALNT2	0.69	1.79	-0.27
A_16_P00271613	226668203	226668262	GALNT2	1.01	1.02	0.04
A_14_P111939	226679171	226679229	GALNT2	0.65	0.94	-0.09
A_16_P35499923	226685483	226685542	GALNT2	1.10	1.33	-0.27
A_16_P00271661	226695650	226695709	GALNT2	0.25	-0.33	-0.26
A_16_P15480145	226703461	226703520	GALNT2	0.85	1.22	-0.41
A_14_P114221	226712699	226712758	GALNT2	0.51	0.90	-0.06
A_16_P00271697	226719193	226719252	GALNT2	1.28	1.26	-0.11
A_16_P00271713	226726590	226726649	chr1:226726590-226726649	0.74	1.18	-0.24
A_16_P15480235	226733503	226733562	chr1:226733503-226733562	0.82	0.79	-0.35
A_16_P00271741	226746086	226746145	chr1:226746086-226746145	1.07	1.13	-0.22
A_14_P101473	226756960	226757019	U79289	0.95	1.05	-0.06
A_16_P15480321	226764085	226764144	chr1:226764085-226764144	0.64	0.34	-0.45
A_16_P00271787	226772995	226773053	PGBD5	-0.03	-0.65	-0.36
A_14_P116004	226784486	226784545	PGBD5	0.48	0.75	-0.49
A_16_P15480410	226795433	226795492	PGBD5	1.03	1.55	-0.26
A_14_P120083	226803741	226803800	PGBD5	1.20	1.28	-0.21
A_16_P00271858	226811427	226811486	PGBD5	0.93	1.13	-0.20
A_16_P00271890	226826674	226826733	chr1:226826674-226826733	0.63	1.46	-0.49
A_16_P15480543	226839909	226839968	chr1:226839909-226839968	0.61	0.99	-0.25
A_16_P00271934	226850315	226850374	chr1:226850315-226850374	0.51	0.91	-0.43
A_14_P106598	226863875	226863934	chr1:226863875-226863934	1.05	1.19	-0.16
A_16_P00271985	226894690	226894749	chr1:226894690-226894749	0.37	1.67	-0.46
A_16_P15480796	226962236	226962295	chr1:226962236-226962295	0.69	0.76	-0.61
A_16_P35500663	226988404	226988463	chr1:226988404-226988463	0.57	1.15	-0.50
A_16_P00272091	227011070	227011129	chr1:227011070-227011129	0.64	1.05	-0.25
A_16_P35500749	227033716	227033775	chr1:227033716-227033775	0.85	0.89	-0.19
A_16_P00272141	227060603	227060662	chr1:227060603-227060662	1.02	0.22	-0.07
A_16_P15480975	227070661	227070720	chr1:227070661-227070720	0.65	1.09	-0.11
A_14_P113812	227086838	227086897	COG2	0.51	1.10	-0.46
A_16_P00272183	227093512	227093571	COG2	0.28	0.35	-0.40
A_14_P134639	227102023	227102082	COG2	0.87	1.62	-0.13

A_16_P00272219	227117463	227117522	COG2	0.38	0.65	-0.49
A_16_P35500976	227129355	227129414	COG2	0.64	0.83	-0.38
A_16_P15481141	227136240	227136297	COG2	1.04	0.79	0.00
A_14_P128545	227145152	227145211	AGT	0.58	0.79	-0.37
A_16_P00272282	227149782	227149841	AGT	0.86	0.93	-0.16
A_16_P00272300	227161796	227161855	chr1:227161796-227161855	0.41	1.77	-0.12
A_16_P00272308	227168968	227169027	chr1:227168968-227169027	0.54	0.15	-0.11
A_16_P00272323	227181487	227181546	chr1:227181487-227181546	1.05	1.41	-0.16
A_16_P15481274	227191812	227191871	CAPN9	0.78	1.09	-0.21
A_14_P115700	227197795	227197851	CAPN9	0.66	0.76	-0.25
A_16_P15481342	227214755	227214814	CAPN9	0.94	0.68	-0.23
A_14_P136528	227222634	227222693	CAPN9	0.88	1.01	-0.07
A_16_P15481375	227228321	227228377	CAPN9	1.01	1.13	-0.18
A_14_P102951	227234368	227234427	CAPN9	1.04	1.46	-0.19
A_14_P115093	227244241	227244292	CAPN9	0.37	1.29	-0.35
A_16_P15481444	227252773	227252832	chr1:227252773-227252832	0.78	0.97	-0.07
A_16_P00272450	227261748	227261807	chr1:227261748-227261807	0.70	1.25	-0.15
A_16_P00272465	227268189	227268248	chr1:227268189-227268248	0.73	0.33	-0.14
A_16_P15481493	227275420	227275479	chr1:227275420-227275479	0.62	1.24	-0.48
A_16_P15481510	227282178	227282237	C1orf198	0.48	1.21	-0.37
A_14_P128530	227287505	227287564	C1orf198	0.87	0.77	-0.09
A_16_P00272511	227296830	227296889	C1orf198	0.74	0.49	-0.18
A_16_P35501481	227305927	227305986	C1orf198	0.64	0.70	-0.11
A_16_P15481601	227312597	227312656	chr1:227312597-227312656	0.86	0.67	-0.22
A_16_P15481619	227320276	227320335	BC032911	0.64	1.01	-0.29
A_16_P00272570	227331218	227331277	chr1:227331218-227331277	1.01	0.23	-0.15
A_16_P00272586	227340994	227341052	chr1:227340994-227341052	1.28	1.46	-0.17
A_14_P139064	227349187	227349246	TTC13	0.17	1.55	-0.13
A_16_P35501663	227363854	227363913	TTC13	0.49	0.57	-0.50
A_14_P134189	227376230	227376289	TTC13	0.22	0.90	-0.24
A_16_P15481798	227382805	227382864	TTC13	0.77	1.02	-0.24
A_16_P00272696	227396801	227396860	TTC13	0.56	1.06	-0.36
A_16_P00272728	227414674	227414733	TTC13	0.69	0.87	-0.41
A_14_P133391	227426196	227426255	ARV1	1.23	1.17	-0.23
A_16_P15481939	227432553	227432612	ARV1	0.58	0.22	-0.33
A_16_P35501881	227438400	227438459	ARV1	0.15	1.27	-0.43
A_14_P120257	227442893	227442952	ARV1	1.31	1.77	-0.17
A_16_P15481978	227452984	227453043	BC016458	0.62	0.85	-0.30
A_16_P00272788	227457092	227457151	BC016458	0.71	1.02	-0.34
A_16_P00272797	227461734	227461793	FAM89A	0.36	1.23	-0.09
A_16_P00272807	227466765	227466824	FAM89A	0.25	-0.70	-0.30
A_14_P137784	227477546	227477605	FAM89A	1.01	1.35	-0.19
A_16_P15482078	227486196	227486255	BC016458	1.04	1.50	-0.12
A_16_P15482092	227491781	227491840	BC016458	0.81	1.82	-0.14
A_16_P35502054	227504896	227504955	BC016458	0.97	1.27	-0.52
A_16_P15482160	227520499	227520558	BC016458	0.25	-0.99	-0.47
A_16_P15482180	227537308	227537367	BC016458	1.10	1.38	-0.29
A_16_P35502137	227548707	227548766	BC016458	0.05	0.45	-0.37

A_16_P15482228	227566456	227566515	BC016458	0.96	1.18	-0.26
A_16_P00272944	227577326	227577385	BC016458	0.97	1.02	-0.23
A_16_P35502200	227591158	227591217	BC016458	0.54	0.88	-0.28
A_16_P15482281	227599504	227599563	BC016458	0.86	1.13	-0.15
A_16_P00272975	227606982	227607041	TRIM67	0.75	0.83	-0.22
A_16_P15482329	227615419	227615478	TRIM67	0.22	-0.13	-0.44
A_14_P201968	227622486	227622545	TRIM67	0.63	1.02	-0.17
A_14_P137604	227627127	227627186	TRIM67	0.55	1.10	-0.32
A_16_P15482383	227634979	227635038	TRIM67	0.94	1.18	-0.34
A_16_P35502342	227643788	227643847	TRIM67	1.03	0.54	-0.39
A_16_P00273041	227649905	227649964	TRIM67	0.80	1.18	-0.22
A_16_P15482454	227660854	227660913	TRIM67	0.89	0.47	0.05
A_14_P108942	227666355	227666414	C1orf131	-0.06	0.64	-0.59
A_16_P15482490	227674488	227674547	C1orf131	0.14	0.07	-0.29
A_16_P15482498	227678694	227678753	C1orf131	0.63	1.20	-0.52
A_14_P103290	227682985	227683044	C1orf131	0.49	0.89	-0.32
A_16_P35502492	227693512	227693571	GNPAT	0.77	1.28	-0.21
A_16_P15482547	227701571	227701630	GNPAT	0.93	1.33	-0.26
A_14_P125225	227708170	227708229	GNPAT	0.88	1.06	-0.12
A_16_P00273156	227720035	227720094	GNPAT	0.73	1.03	-0.29
A_16_P00273166	227733220	227733279	BC016458	1.10	-0.04	-0.30
A_16_P00273192	227746026	227746085	BC016458	0.95	0.98	-0.40
A_14_P113362	227755876	227755935	BC016458	0.35	0.16	-0.64
A_16_P15482713	227760923	227760982	BC016458	1.41	0.95	-0.04
A_16_P35502700	227770444	227770503	BC016458	0.47	0.60	-0.46
A_14_P111529	227776373	227776432	EXOC8	-0.07	0.98	-0.52
A_14_P134260	227781345	227781404	C1orf124	0.89	1.24	-0.17
A_16_P15482761	227784557	227784616	C1orf124	0.90	0.90	-0.16
A_16_P15482771	227790228	227790287	C1orf124	0.92	0.71	-0.07
A_16_P15482789	227795569	227795628	C1orf124	0.75	0.95	-0.32
A_16_P15482802	227800675	227800734	BC016458	0.50	0.51	-0.21
A_16_P15482826	227808234	227808293	EGLN1	0.27	1.05	0.17
A_16_P35502844	227816780	227816839	EGLN1	0.53	0.94	-0.48
A_16_P15482879	227824703	227824762	EGLN1	0.59	1.23	-0.21
A_14_P123420	227830232	227830291	EGLN1	0.87	1.48	-0.33
A_16_P00273347	227842371	227842430	EGLN1	0.62	0.97	-0.32
A_16_P35502944	227854896	227854955	EGLN1	0.17	1.06	-0.58
A_16_P35502960	227860615	227860674	EGLN1	0.51	1.33	-0.40
A_14_P113236	227866642	227866701	EGLN1	1.00	1.59	-0.25
A_16_P15483001	227875258	227875317	BC016458	0.54	0.77	-0.37
A_16_P15483007	227883438	227883497	BC016458	0.23	0.92	-0.52
A_16_P00273405	227905403	227905462	chr1:227905403-227905462	0.79	1.37	-0.26
A_16_P15483030	227913150	227913209	chr1:227913150-227913209	1.07	1.15	-0.27
A_16_P00273426	227921505	227921564	chr1:227921505-227921564	1.13	0.69	-0.17
A_16_P15483070	227928097	227928156	chr1:227928097-227928156	0.75	0.53	-0.35
A_14_P134237	227935937	227935996	chr1:227935937-227935996	0.20	0.49	-0.31
A_16_P15483094	227944756	227944815	chr1:227944756-227944815	0.34	0.58	-0.42
A_16_P15483122	227959975	227960034	chr1:227959975-227960034	1.22	0.55	-0.23

A_16_P15483137	227968560	227968619	chr1:227968560-227968619	0.96	1.20	-0.17
A_16_P35503181	227984911	227984970	TSNAX	0.32	1.20	-0.46
A_14_P130208	227992141	227992200	TSNAX	0.92	0.90	-0.15
A_16_P35503224	227999211	227999270	TSNAX	0.40	0.62	-0.24
A_16_P15483246	228011538	228011597	chr1:228011538-228011597	0.39	0.63	-0.46
A_16_P00273557	228031682	228031741	chr1:228031682-228031741	1.29	0.99	-0.02
A_14_P119376	228044661	228044720	chr1:228044661-228044720	0.69	0.79	-0.25
A_16_P15483344	228056424	228056483	chr1:228056424-228056483	0.31	0.03	-0.41
A_16_P00273617	228073158	228073217	DISC1	1.05	1.12	-0.34
A_16_P00273638	228086632	228086691	DISC1	1.13	0.85	-0.27
A_16_P15483430	228095042	228095101	DISC1	0.67	0.96	-0.30
A_16_P00273652	228110192	228110251	DISC1	0.77	1.19	-0.28
A_16_P00273666	228120656	228120715	DISC1	0.95	1.60	-0.21
A_16_P15483485	228127195	228127254	DISC1	1.03	0.80	-0.28
A_16_P15483501	228134096	228134155	DISC1	1.04	1.56	-0.60
A_16_P00273699	228138956	228139015	DISC1	0.22	1.44	-0.19
A_14_P117145	228144051	228144110	DISC1	0.50	1.81	-0.51
A_16_P15483545	228149006	228149065	DISC1	0.83	0.98	-0.25
A_16_P15483559	228153618	228153677	DISC1	0.86	0.76	-0.29
A_16_P15483562	228157842	228157901	DISC1	0.18	0.34	-0.52
A_14_P116361	228166425	228166477	DISC1	0.95	0.93	-0.07
A_16_P15483613	228173476	228173535	DISC1	0.20	0.61	-0.42
A_16_P00273771	228182747	228182806	DISC1	0.67	0.99	-0.38
A_16_P15483659	228198654	228198713	DISC1	0.32	-0.28	-0.57
A_16_P15483682	228206012	228206071	DISC1	0.28	1.08	-0.63
A_16_P15483719	228217595	228217654	DISC1	1.01	1.16	-0.01
A_16_P15483733	228224064	228224123	DISC1	0.32	0.49	-0.50
A_16_P15483764	228235537	228235596	DISC1	0.42	1.26	-0.43
A_16_P00273871	228244182	228244241	DISC1	1.19	1.20	-0.36
A_16_P15483819	228254040	228254099	DISC1	0.71	1.28	-0.43
A_16_P35503867	228262430	228262489	DISC1	0.50	1.11	-0.33
A_16_P15483873	228278308	228278367	DISC1	0.71	1.34	-0.20
A_16_P35503917	228284854	228284913	DISC1	0.62	0.93	-0.12
A_16_P15483906	228292115	228292174	DISC1	0.24	0.25	-0.25
A_16_P00273956	228301129	228301188	DISC1	0.76	1.31	-0.51
A_14_P202097	228309225	228309284	DISC1	0.49	1.54	-0.60
A_14_P109642	228309285	228309340	DISC1	0.55	0.88	-0.35
A_16_P00273990	228317420	228317479	DISC1	0.87	0.76	-0.24
A_16_P00274008	228327619	228327678	DISC1	0.49	1.47	-0.09
A_16_P15484066	228340922	228340981	DISC1	1.02	1.51	-0.19
A_16_P00274041	228348848	228348907	DISC1	0.29	1.30	-0.35
A_16_P00274055	228361860	228361919	DISC1	0.35	0.51	-0.21
A_16_P00274070	228371857	228371916	DISC1	1.11	0.90	-0.26
A_14_P115749	228379667	228379726	DISC1	-0.09	1.05	-0.37
A_16_P00274104	228395818	228395877	DISC1	0.40	0.46	-0.61
A_16_P00274111	228401361	228401420	DISC1	0.46	0.21	-0.37
A_16_P00274133	228414357	228414416	DISC1	0.96	0.41	-0.50
A_16_P00274144	228422777	228422836	DISC1	0.84	1.53	-0.46

A_16_P15484289	228434431	228434490	DISC1	0.97	1.39	-0.18
A_16_P15484322	228452554	228452613	DISC1	1.25	0.87	-0.15
A_16_P00274205	228460478	228460537	DISC1	0.19	-0.73	-0.14
A_16_P15484383	228471156	228471215	DISC1	0.02	1.09	-0.21
A_16_P15484415	228481844	228481903	DISC1	0.43	0.04	-0.38
A_14_P126778	228498557	228498616	chr1:228498557-228498616	1.03	0.64	-0.18
A_16_P00274293	228510124	228510183	chr1:228510124-228510183	0.75	1.16	-0.16
A_16_P00274320	228540149	228540208	chr1:228540149-228540208	1.16	0.56	-0.26
A_16_P15484649	228589609	228589668	chr1:228589609-228589668	0.95	0.78	-0.04
A_14_P126052	228615544	228615603	chr1:228615544-228615603	0.40	0.18	0.23
A_16_P00274455	228636679	228636738	chr1:228636679-228636738	0.74	1.06	-0.32
A_16_P00274472	228660883	228660942	chr1:228660883-228660942	0.79	0.50	-0.38
A_14_P123588	228690639	228690698	chr1:228690639-228690698	0.38	1.14	-0.28
A_16_P15484968	228725103	228725162	chr1:228725103-228725162	1.07	1.20	-0.19
A_16_P15485067	228759448	228759507	chr1:228759448-228759507	0.80	1.44	-0.30
A_16_P35505221	228804605	228804664	chr1:228804605-228804664	0.39	1.40	-0.35
A_16_P35505272	228823973	228824032	chr1:228823973-228824032	0.62	1.03	-0.38
A_16_P00274741	228832739	228832798	chr1:228832739-228832798	0.78	1.48	-0.35
A_16_P35505327	228844821	228844880	SIPA1L2	0.74	0.73	-0.32
A_16_P15485318	228851785	228851844	SIPA1L2	-0.42	1.39	-0.66
A_16_P15485339	228857830	228857883	SIPA1L2	0.69	0.98	-0.03
A_16_P35505398	228864018	228864077	SIPA1L2	0.81	1.19	-0.38
A_16_P15485389	228872898	228872957	SIPA1L2	0.72	1.45	-0.37
A_16_P15485411	228879552	228879611	SIPA1L2	0.52	0.73	-0.36
A_16_P00274856	228890941	228891000	SIPA1L2	1.09	1.19	-0.21
A_16_P15485479	228899187	228899246	SIPA1L2	1.16	1.22	-0.26
A_16_P15485505	228906695	228906754	SIPA1L2	0.51	1.29	-0.40
A_16_P15485535	228914985	228915044	SIPA1L2	0.28	0.25	-0.60
A_16_P00274933	228925123	228925182	SIPA1L2	0.85	0.85	-0.61
A_16_P00274958	228936098	228936157	SIPA1L2	0.82	1.47	-0.29
A_14_P136424	228950287	228950346	SIPA1L2	0.77	0.95	-0.25
A_16_P00274994	228956782	228956841	SIPA1L2	0.71	0.80	-0.26
A_16_P35505773	228971783	228971842	chr1:228971783-228971842	0.42	0.68	-0.55
A_16_P15485752	228981770	228981827	chr1:228981770-228981827	1.11	1.86	-0.23
A_16_P15485826	229003633	229003692	chr1:229003633-229003692	1.01	0.94	-0.18
A_16_P15485870	229023379	229023438	chr1:229023379-229023438	1.06	1.14	-0.33
A_14_P120996	229053043	229053102	chr1:229053043-229053102	0.94	0.26	-0.38
A_16_P00275226	229079705	229079764	chr1:229079705-229079764	1.00	0.38	-0.32
A_16_P15486185	229132023	229132082	chr1:229132023-229132082	0.82	1.43	0.16
A_16_P15486297	229177950	229178009	AK093200	0.53	0.48	-0.66
A_16_P15486322	229193472	229193531	chr1:229193472-229193531	0.43	0.18	-0.42
A_16_P35506450	229215120	229215179	chr1:229215120-229215179	0.60	0.43	-0.35
A_16_P00275463	229230694	229230753	chr1:229230694-229230753	1.00	0.02	-0.10
A_14_P119094	229249019	229249078	BC109122	0.96	1.01	-0.41
A_14_P122894	229251385	229251444	AB037804	0.86	1.07	-0.29
A_16_P15486511	229257363	229257422	chr1:229257363-229257422	0.42	1.00	-0.42
A_16_P15486525	229264296	229264355	chr1:229264296-229264355	0.34	0.22	-0.51
A_16_P35506597	229272251	229272310	chr1:229272251-229272310	0.47	0.90	-0.08

A_14_P136151	229288266	229288325	chr1:229288266-229288325	0.03	0.83	0.08
A_16_P00275589	229314910	229314969	chr1:229314910-229314969	0.69	0.98	-0.24
A_16_P15486691	229331495	229331554	chr1:229331495-229331554	0.23	0.29	-0.54
A_16_P15486739	229351207	229351266	chr1:229351207-229351266	0.92	0.37	-0.11
A_16_P00275672	229369284	229369343	chr1:229369284-229369343	0.95	0.83	-0.24
A_16_P00275689	229383343	229383402	chr1:229383343-229383402	1.11	-0.04	-0.30
A_16_P00275698	229390287	229390346	chr1:229390287-229390346	0.08	1.20	-0.44
A_14_P121687	229398038	229398095	C1orf57	1.05	1.18	-0.47
A_16_P15486885	229408721	229408780	C1orf57	0.81	1.00	-0.15
A_16_P00275743	229417067	229417126	C1orf57	0.65	0.29	-0.40
A_14_P132905	229424054	229424113	AK094245	0.49	0.49	-0.36
A_16_P00275774	229430685	229430744	PCNXL2	0.39	1.20	-0.28
A_16_P35507057	229444178	229444237	PCNXL2	0.93	0.98	-0.27
A_16_P15487018	229453181	229453240	PCNXL2	0.70	0.67	-0.20
A_14_P121638	229459581	229459640	PCNXL2	0.67	2.07	0.13
A_16_P15487067	229468024	229468083	PCNXL2	0.90	0.74	-0.17
A_16_P00275847	229473789	229473848	PCNXL2	1.00	2.17	-0.71
A_16_P15487092	229480884	229480943	PCNXL2	0.55	0.90	-0.55
A_14_P135594	229499689	229499744	PCNXL2	-0.08	0.76	-0.72
A_16_P35507222	229507809	229507868	PCNXL2	0.21	0.89	-0.43
A_16_P35507234	229520534	229520593	PCNXL2	0.86	0.89	-0.17
A_16_P00275926	229531954	229532013	PCNXL2	0.24	0.82	-0.51
A_16_P15487227	229540877	229540936	PCNXL2	0.86	0.95	-0.47
A_16_P35507315	229548315	229548374	PCNXL2	0.80	0.74	-0.36
A_16_P00275963	229555377	229555433	PCNXL2	1.06	1.38	0.01
A_16_P15487263	229566302	229566361	PCNXL2	0.80	1.42	-0.36
A_16_P00275995	229582272	229582331	PCNXL2	0.32	1.07	-0.54
A_16_P00276004	229589056	229589115	PCNXL2	0.56	0.34	-0.34
A_16_P00276027	229601256	229601315	PCNXL2	0.69	1.82	-0.15
A_16_P35507477	229615464	229615523	PCNXL2	0.81	0.97	-0.37
A_16_P15487421	229622712	229622771	PCNXL2	1.21	1.44	-0.21
A_16_P15487441	229628785	229628844	PCNXL2	0.63	0.62	-0.38
A_14_P110648	229642619	229642678	PCNXL2	1.13	0.20	-0.30
A_16_P35507592	229654229	229654288	PCNXL2	1.28	0.75	-0.20
A_16_P15487526	229660408	229660467	PCNXL2	0.71	0.70	-0.35
A_16_P35507656	229676955	229677014	PCNXL2	0.22	1.15	-0.28
A_16_P00276178	229693265	229693324	PCNXL2	0.59	1.06	-0.60
A_16_P00276178	229693265	229693324	PCNXL2	0.48	0.93	-0.34
A_16_P00276178	229693265	229693324	PCNXL2	0.51	0.74	-0.33
A_14_P108586	229700662	229700721	PCNXL2	0.64	0.90	-0.18
A_16_P15487687	229712396	229712455	AK092353	0.95	1.13	-0.68
A_16_P15487715	229723773	229723832	AK092353	0.50	0.61	-0.42
A_14_P107880	229729550	229729609	AK092353	0.88	1.42	-0.29
A_16_P35507828	229737029	229737088	AK092353	0.55	1.09	-0.36
A_16_P15487768	229743501	229743560	chr1:229743501-229743560	0.30	0.73	-0.43
A_16_P15487788	229757354	229757413	chr1:229757354-229757413	0.46	1.13	-0.32
A_16_P15487822	229773497	229773556	KIAA1804	0.38	0.78	-0.36
A_16_P00276297	229779315	229779374	KIAA1804	1.01	1.22	-0.48

A_14_P126089	229788950	229789005	KIAA1804	0.54	1.28	-0.10
A_16_P00276325	229797404	229797463	KIAA1804	0.55	0.56	-0.41
A_16_P35507995	229803495	229803554	KIAA1804	0.44	2.21	0.17
A_14_P129957	229814364	229814423	KIAA1804	1.01	1.20	-0.47
A_14_P102858	229815908	229815967	KIAA1804	0.00	-0.49	-0.39
A_14_P133535	229818357	229818416	KIAA1804	0.44	1.22	-0.43
A_16_P15487969	229825151	229825210	KIAA1804	0.80	0.99	-0.24
A_16_P35508110	229842327	229842386	chr1:229842327-229842386	0.58	1.17	-0.35
A_14_P115516	229849489	229849548	chr1:229849489-229849548	0.50	1.16	-0.30
A_16_P15488050	229861387	229861446	chr1:229861387-229861446	0.83	1.06	-0.23
A_16_P35508162	229878120	229878179	chr1:229878120-229878179	0.61	0.88	-0.24
A_16_P15488104	229892982	229893041	chr1:229892982-229893041	0.30	0.96	-0.52
A_16_P15488180	229929040	229929099	chr1:229929040-229929099	0.72	1.37	-0.29
A_14_P133160	229955847	229955906	chr1:229955847-229955906	0.64	0.90	-0.33
A_16_P15488297	229989980	229990039	chr1:229989980-229990039	0.70	1.07	-0.46
A_16_P15488348	230015405	230015464	chr1:230015405-230015464	1.24	1.58	-1.89
A_16_P35508464	230029994	230030053	chr1:230029994-230030053	0.32	1.48	-0.32
A_16_P15488382	230041311	230041370	chr1:230041311-230041370	0.90	0.58	-0.40
A_16_P00276624	230052163	230052222	chr1:230052163-230052222	0.52	0.56	-0.45
A_16_P15488435	230063097	230063156	KCNK1	0.46	0.70	-0.34
A_14_P102476	230074867	230074926	KCNK1	0.31	1.59	-0.39
A_16_P15488495	230083737	230083796	KCNK1	0.55	0.32	-0.38
A_14_P114583	230094347	230094406	KCNK1	0.61	0.85	-0.49
A_16_P15488534	230102988	230103047	KCNK1	0.92	0.77	-0.37
A_14_P115075	230114723	230114782	BC018051	0.60	1.20	-0.42
A_14_P202239	230114860	230114919	chr1:230114860-230114919	0.05	1.59	0.09
A_16_P35508722	230125837	230125896	chr1:230125837-230125896	0.97	1.35	-0.08
A_16_P15488618	230136118	230136177	chr1:230136118-230136177	0.50	0.59	-0.17
A_16_P15488646	230148446	230148505	chr1:230148446-230148505	0.74	0.90	-0.31
A_16_P00276796	230163825	230163884	chr1:230163825-230163884	0.75	0.83	-0.39
A_16_P35508850	230189267	230189326	chr1:230189267-230189326	0.81	1.36	-0.29
A_16_P00276849	230220325	230220384	chr1:230220325-230220384	0.92	1.01	-0.37
A_14_P118872	230250952	230251011	chr1:230250952-230251011	0.96	0.80	-0.28
A_16_P15488919	230287178	230287237	chr1:230287178-230287237	0.46	0.48	-0.33
A_16_P00276949	230304993	230305052	chr1:230304993-230305052	0.64	0.90	0.13
A_16_P15488987	230320756	230320815	chr1:230320756-230320815	0.46	1.34	-0.44
A_16_P15489019	230332384	230332443	chr1:230332384-230332443	1.01	1.53	-0.29
A_16_P00276992	230340254	230340313	chr1:230340254-230340313	0.67	0.65	-0.33
A_16_P15489049	230349681	230349740	SLC35F3	0.41	0.46	-0.39
A_16_P15489069	230357599	230357658	SLC35F3	0.74	1.10	-0.46
A_14_P115529	230376766	230376825	SLC35F3	0.94	1.49	-0.28
A_16_P15489119	230383002	230383061	SLC35F3	1.13	1.40	-0.43
A_16_P15489153	230394761	230394820	SLC35F3	0.30	1.24	-0.68
A_16_P15489186	230405232	230405291	SLC35F3	1.19	0.95	-0.31
A_16_P15489203	230411607	230411666	SLC35F3	0.59	0.50	-0.25
A_16_P35509364	230425571	230425630	SLC35F3	1.04	0.94	-0.31
A_16_P00277131	230434692	230434751	SLC35F3	0.73	1.34	-0.42
A_16_P15489287	230444220	230444279	SLC35F3	0.59	0.99	-0.35

A_16_P15489308	230451822	230451881	SLC35F3	0.92	1.33	-0.39
A_16_P15489325	230465215	230465274	SLC35F3	0.63	1.34	-0.34
A_14_P134269	230472983	230473042	SLC35F3	0.37	0.81	-0.33
A_16_P00277192	230485566	230485625	SLC35F3	0.84	0.87	-0.29
A_16_P15489368	230498552	230498611	SLC35F3	1.03	1.15	-0.27
A_16_P35509504	230510666	230510725	SLC35F3	1.10	1.02	-0.15
A_16_P00277214	230517239	230517298	SLC35F3	1.05	1.45	-0.05
A_16_P00277225	230526130	230526189	SLC35F3	0.83	0.65	-0.22
A_16_P15489453	230536639	230536698	SLC35F3	0.85	1.06	-0.25
A_16_P35509593	230542307	230542366	SLC35F3	0.27	0.83	-0.24
A_16_P15489489	230553221	230553280	SLC35F3	0.77	0.81	-0.05
A_16_P15489510	230560690	230560749	SLC35F3	1.03	1.17	-0.42
A_16_P15489541	230571358	230571417	SLC35F3	1.07	0.91	-0.16
A_14_P108748	230580507	230580566	SLC35F3	0.38	0.97	-0.20
A_16_P35509719	230592272	230592331	SLC35F3	0.60	0.50	-0.58
A_16_P15489619	230603393	230603452	SLC35F3	0.77	1.14	-0.50
A_16_P00277365	230613445	230613504	SLC35F3	0.12	1.50	-0.29
A_16_P35509803	230620473	230620532	SLC35F3	0.21	1.13	-0.73
A_16_P00277392	230628349	230628408	SLC35F3	0.89	0.87	-0.23
A_16_P00277399	230640376	230640435	SLC35F3	0.52	1.10	-0.32
A_16_P00277410	230647461	230647520	SLC35F3	0.54	1.66	-0.12
A_16_P00277423	230655770	230655829	SLC35F3	1.14	0.03	-0.15
A_16_P00277438	230662804	230662863	SLC35F3	0.56	0.82	-0.39
A_16_P35509932	230672719	230672778	SLC35F3	0.63	0.71	-0.50
A_16_P15489840	230682511	230682570	SLC35F3	0.97	1.02	-0.29
A_16_P00277495	230692688	230692747	SLC35F3	0.79	1.44	-0.27
A_16_P15489880	230699636	230699695	SLC35F3	0.25	0.82	-0.47
A_16_P15489901	230706608	230706667	SLC35F3	1.05	1.09	-0.34
A_14_P122693	230717945	230718004	SLC35F3	0.67	1.05	-0.12
A_16_P00277548	230730234	230730293	SLC35F3	0.84	0.63	-0.23
A_16_P00277560	230735876	230735935	SLC35F3	0.59	0.58	-0.38
A_16_P00277570	230741585	230741644	SLC35F3	1.20	0.99	-0.36
A_16_P00277574	230747138	230747197	SLC35F3	0.89	1.26	-0.21
A_16_P15490017	230753332	230753391	SLC35F3	1.00	0.59	-0.34
A_14_P109167	230766961	230767020	SLC35F3	0.99	1.55	-0.07
A_16_P35510215	230772688	230772747	chr1:230772688-230772747	0.50	1.26	-0.51
A_16_P35510234	230785795	230785854	chr1:230785795-230785854	0.50	1.28	-0.43
A_16_P15490113	230798275	230798334	chr1:230798275-230798334	0.03	0.39	-0.25
A_16_P35510264	230803192	230803251	chr1:230803192-230803251	0.75	1.35	-0.20
A_16_P35510275	230810906	230810965	chr1:230810906-230810965	0.97	1.18	-0.26
A_14_P126554	230817273	230817332	C1orf31	1.00	1.62	-0.23
A_16_P00277670	230822763	230822822	C1orf31	0.67	1.19	-0.11
A_16_P35510322	230826703	230826762	CR602593	0.75	0.44	-0.10
A_16_P00277683	230834906	230834963	TARBP1	0.00	1.50	0.17
A_14_P108250	230843649	230843708	TARBP1	0.75	0.87	-0.26
A_16_P15490240	230851560	230851619	TARBP1	1.03	1.31	-0.22
A_16_P00277733	230863230	230863289	TARBP1	1.09	1.49	-0.17
A_14_P118479	230872046	230872105	TARBP1	1.23	1.32	-0.21

A_16_P00277755	230875904	230875963	TARBP1	0.52	1.10	-0.79
A_14_P115081	230892897	230892956	TARBP1	0.42	0.33	-0.28
A_16_P00277787	230900126	230900185	TARBP1	0.54	1.03	-0.01
A_14_P123862	230910028	230910087	TARBP1	0.02	1.42	-0.91
A_16_P35510558	230915158	230915217	TARBP1	0.67	1.24	-0.26
A_16_P15490423	230930917	230930976	chr1:230930917-230930976	1.53	1.01	-0.21
A_16_P35510635	230945978	230946037	chr1:230945978-230946037	1.40	0.94	-0.09
A_14_P127165	230972236	230972295	BC040195	0.22	-0.02	-0.41
A_16_P00277941	231001191	231001250	chr1:231001191-231001250	1.61	1.25	-4.24
A_16_P35510833	231019063	231019122	chr1:231019063-231019122	0.41	1.14	-0.39
A_16_P35510871	231030247	231030306	chr1:231030247-231030306	0.59	1.23	-0.39
A_16_P35510891	231036924	231036983	chr1:231036924-231036983	0.64	0.93	-0.40
A_16_P15490744	231045020	231045079	chr1:231045020-231045079	0.61	0.85	-0.26
A_14_P100465	231049352	231049411	BC020516	1.25	1.01	-0.12
A_14_P100465	231049352	231049411	BC020516	1.31	0.59	-0.22
A_14_P100465	231049352	231049411	BC020516	1.33	0.63	-0.16
A_14_P129127	231049586	231049642	IRF2BP2	0.62	1.22	-0.33
A_16_P00278035	231054132	231054191	chr1:231054132-231054191	0.64	0.45	-0.14
A_16_P00278052	231061656	231061715	chr1:231061656-231061715	0.57	1.09	-0.35
A_16_P15490822	231067740	231067799	chr1:231067740-231067799	0.77	0.64	-0.34
A_14_P115515	231081460	231081519	chr1:231081460-231081519	0.75	1.26	-0.54
A_16_P35511059	231092522	231092581	BC032040	0.50	-1.81	-0.26
A_16_P00278117	231103526	231103585	AK058184	0.69	1.11	-0.22
A_16_P35511121	231116367	231116426	AK127758	0.86	1.22	-0.21
A_16_P35511147	231127236	231127295	AK127758	0.43	1.13	-0.42
A_16_P00278165	231139619	231139678	chr1:231139619-231139678	0.84	0.71	-0.26
A_16_P35511282	231174167	231174226	chr1:231174167-231174226	0.57	1.64	-0.42
A_16_P00278259	231188620	231188679	chr1:231188620-231188679	0.98	0.71	-0.27
A_16_P35511354	231200213	231200272	AF193050	0.96	0.90	-0.28
A_16_P35511381	231211008	231211067	chr1:231211008-231211067	1.07	0.80	-0.15
A_16_P35511431	231225171	231225230	chr1:231225171-231225230	1.37	0.90	0.41
A_16_P00278370	231280249	231280308	chr1:231280249-231280308	0.49	0.92	-0.24
A_16_P35511649	231304810	231304869	chr1:231304810-231304869	1.05	0.75	-0.22
A_16_P35511724	231331694	231331753	chr1:231331694-231331753	0.91	1.13	-0.31
A_14_P106810	231353119	231353178	chr1:231353119-231353178	0.67	0.58	-0.31
A_16_P00278527	231374599	231374658	chr1:231374599-231374658	1.00	0.83	-0.13
A_16_P00278606	231426387	231426446	chr1:231426387-231426446	0.79	0.17	-0.13
A_16_P15491890	231462548	231462607	chr1:231462548-231462607	1.17	1.68	0.03
A_16_P35512090	231472115	231472174	chr1:231472115-231472174	0.68	1.55	-0.45
A_16_P00278667	231568431	231568490	chr1:231568431-231568490	0.92	1.38	-0.06
A_16_P35512238	231585826	231585885	chr1:231585826-231585885	0.45	0.78	-0.40
A_14_P201046	231593585	231593644	chr1:231593585-231593644	1.18	0.89	-0.36
A_16_P15492102	231606150	231606209	TOMM20	0.78	0.39	-0.47
A_14_P114410	231651051	231651106	AL133010	0.77	0.64	-0.31
A_16_P15492213	231656907	231656966	ARID4B	0.11	1.04	-0.17
A_16_P00278738	231664512	231664571	ARID4B	1.05	0.90	-0.21
A_14_P117467	231671224	231671283	ARID4B	1.02	1.86	-0.31
A_16_P15492280	231683384	231683443	ARID4B	1.02	1.60	-0.12

A_16_P15492305	231691826	231691885	ARID4B	-0.21	0.51	-0.48
A_16_P35512545	231697544	231697603	ARID4B	0.69	0.56	-0.17
A_16_P35512563	231703390	231703449	ARID4B	0.80	0.89	-0.24
A_14_P119624	231709204	231709263	ARID4B	0.41	1.54	-0.06
A_16_P00278839	231723774	231723833	ARID4B	0.07	0.63	-0.38
A_16_P00278861	231734456	231734515	ARID4B	0.90	1.11	-0.09
A_16_P35512674	231740158	231740217	ARID4B	0.45	1.05	-0.27
A_14_P117779	231746492	231746551	ARID4B	0.71	1.01	-0.12
A_16_P15492482	231752740	231752799	ARID4B	0.09	-0.46	-0.35
A_16_P35512745	231761492	231761551	ARID4B	0.56	1.15	-0.13
A_16_P00278924	231771796	231771855	ARID4B	1.04	1.33	-0.01
A_16_P15492547	231778255	231778314	ARID4B	0.42	0.42	-0.20
A_16_P15492571	231788100	231788159	ARID4B	0.49	0.72	-0.19
A_14_P128006	231794700	231794759	ARID4B	0.89	0.74	0.02
A_16_P15492600	231802977	231803036	ARID4B	0.59	0.30	-0.51
A_16_P15492612	231809007	231809066	ARID4B	0.97	0.92	-0.13
A_16_P15492629	231815596	231815655	ARID4B	0.89	1.30	-0.14
A_14_P123801	231824603	231824662	GGPS1	0.70	1.56	-0.21
A_16_P15492678	231831713	231831772	GGPS1	0.89	0.66	-0.15
A_16_P15492701	231841227	231841286	chr1:231841227-231841286	0.94	1.61	-0.16
A_16_P35512958	231846458	231846517	BC041948	0.92	0.98	-0.12
A_16_P35512970	231851407	231851455	chr1:231851407-231851455	1.08	0.90	-0.28
A_16_P00279032	231857195	231857254	TBCE	1.20	1.29	-0.03
A_16_P35512997	231869599	231869658	TBCE	0.49	1.29	-0.15
A_14_P124853	231877348	231877407	TBCE	0.62	0.67	-0.25
A_16_P00279052	231892283	231892342	TBCE	1.17	0.74	-0.22
A_16_P00279061	231903828	231903887	TBCE	0.74	1.39	-0.30
A_16_P00279077	231916498	231916557	TBCE	0.71	-0.08	-0.38
A_16_P00279086	231923594	231923653	TBCE	0.96	0.93	-0.09
A_16_P15492849	231932201	231932260	TBCE	0.88	1.24	-0.53
A_14_P112430	231938227	231938286	TBCE	0.66	1.73	-0.14
A_16_P00279137	231947928	231947987	B3GALNT2	0.41	0.75	-0.51
A_16_P15492912	231955410	231955469	B3GALNT2	0.07	0.34	-0.78
A_14_P102632	231965390	231965449	B3GALNT2	0.65	0.43	-0.19
A_16_P15492960	231973455	231973514	B3GALNT2	0.39	1.00	-0.25
A_14_P115107	231985197	231985256	B3GALNT2	0.61	0.86	-0.40
A_16_P15493037	232000259	232000318	chr1:232000259-232000318	0.94	0.39	-0.13
A_14_P119602	232007342	232007401	chr1:232007342-232007401	0.16	0.60	-0.54
A_16_P15493089	232019213	232019272	chr1:232019213-232019272	0.57	0.16	-0.39
A_14_P117105	232040994	232041052	GNG4	-0.24	0.33	-0.52
A_16_P35513460	232055352	232055411	GNG4	0.99	1.00	-0.35
A_16_P35513476	232065773	232065832	GNG4	0.04	0.36	-0.71
A_16_P00279311	232075492	232075551	GNG4	0.97	1.38	-0.26
A_14_P132505	232085167	232085226	GNG4	0.84	1.37	0.09
A_16_P15493249	232090762	232090821	GNG4	0.79	0.74	-0.26
A_16_P15493273	232100857	232100916	GNG4	1.37	1.20	-0.08
A_14_P127898	232107790	232107849	GNG4	0.58	1.12	0.14
A_16_P00279360	232114059	232114118	GNG4	0.78	1.32	-0.23

A_16_P00279377	232123243	232123302	GNG4	0.22	0.08	-0.19
A_16_P00279385	232129811	232129870	GNG4	1.01	0.69	-0.27
A_16_P15493358	232136603	232136662	GNG4	0.55	1.31	-0.41
A_14_P116315	232145254	232145313	chr1:232145254-232145313	0.28	0.95	-0.29
A_16_P00279425	232152051	232152110	LYST	0.56	0.83	-0.34
A_16_P35513713	232165329	232165388	LYST	0.53	0.87	-0.43
A_16_P35513743	232176024	232176083	LYST	0.85	0.58	-0.31
A_16_P00279470	232186415	232186474	LYST	0.37	0.40	-0.60
A_14_P133092	232201481	232201540	LYST	0.20	0.49	-0.54
A_16_P35513840	232210000	232210059	LYST	0.89	1.18	-0.25
A_16_P15493563	232220354	232220413	LYST	0.70	0.98	-0.49
A_16_P35513923	232235643	232235702	LYST	0.46	0.78	-0.51
A_14_P121822	232244894	232244953	LYST	0.66	0.48	-0.52
A_16_P15493661	232252165	232252224	LYST	0.32	0.12	-0.72
A_16_P15493687	232260851	232260910	LYST	0.45	1.19	-0.33
A_16_P15493722	232271310	232271369	LYST	0.59	1.11	-0.20
A_14_P139783	232278082	232278141	LYST	0.74	0.34	-0.41
A_16_P15493770	232289673	232289732	LYST	0.57	1.16	-0.30
A_16_P00279674	232299374	232299433	LYST	0.48	-0.13	-0.39
A_16_P35514182	232312763	232312822	LYST	0.48	1.33	-0.50
A_14_P126472	232322906	232322965	LYST	0.08	0.90	-0.35
A_16_P00279713	232330487	232330546	LYST	0.95	0.75	-0.28
A_16_P00279722	232342263	232342322	LYST	1.07	0.49	-0.41
A_16_P15493913	232351246	232351305	LYST	0.85	0.99	-0.38
A_16_P15493939	232363045	232363104	LYST	0.34	0.60	-0.44
A_14_P135983	232375425	232375484	chr1:232375425-232375484	0.31	1.13	-0.08
A_16_P15494007	232391819	232391878	chr1:232391819-232391878	0.64	1.45	-0.34
A_16_P15494036	232403461	232403520	chr1:232403461-232403520	0.85	1.00	-0.33
A_16_P15494101	232431071	232431130	chr1:232431071-232431130	0.82	1.68	-0.63
A_16_P00279869	232446725	232446784	chr1:232446725-232446784	0.76	1.23	-0.32
A_14_P104787	232465481	232465539	BC045606	0.62	0.92	-0.38
A_16_P00279911	232476547	232476606	NID1	0.91	1.26	-0.25
A_14_P121736	232488029	232488088	NID1	0.17	0.97	-0.26
A_16_P35514604	232494771	232494830	NID1	0.58	1.17	-0.34
A_14_P133253	232506565	232506614	NID1	0.72	1.04	-0.31
A_16_P00279972	232515108	232515167	NID1	0.14	2.03	-0.48
A_16_P15494309	232521191	232521250	NID1	0.78	0.73	-0.14
A_14_P123639	232534912	232534966	NID1	0.82	0.30	-0.47
A_16_P00280018	232540841	232540900	NID1	1.17	0.98	-0.26
A_16_P00280030	232550121	232550180	NID1	0.78	1.03	-0.36
A_16_P15494402	232557371	232557430	chr1:232557371-232557430	1.10	1.50	-0.36
A_16_P00280054	232573499	232573558	chr1:232573499-232573558	1.29	1.12	-0.23
A_16_P00280054	232573499	232573558	chr1:232573499-232573558	1.18	1.17	-0.20
A_16_P00280054	232573499	232573558	chr1:232573499-232573558	1.16	1.14	-0.15
A_16_P15494474	232593090	232593149	chr1:232593090-232593149	0.74	1.29	-0.30
A_16_P15494527	232612020	232612079	chr1:232612020-232612079	0.78	1.09	-0.27
A_16_P35514943	232624854	232624913	chr1:232624854-232624913	0.97	1.32	-0.17
A_16_P00280135	232636055	232636114	GPR137B	0.76	1.81	-0.53

A_16_P15494603	232643073	232643132	GPR137B	1.08	1.00	-0.03
A_16_P00280157	232649444	232649503	GPR137B	0.97	1.21	-0.31
A_14_P132660	232658040	232658098	GPR137B	0.69	0.64	-0.18
A_16_P00280185	232666810	232666868	GPR137B	1.29	1.52	0.11
A_16_P00280195	232673196	232673255	GPR137B	0.17	1.26	-0.61
A_16_P35515079	232684239	232684298	GPR137B	0.32	0.58	-0.53
A_14_P124080	232698211	232698270	GPR137B	0.36	0.96	-0.18
A_16_P15494732	232706448	232706507	CR749523	0.71	0.51	-0.20
A_14_P135973	232715732	232715791	ERO1LB	0.56	2.03	-0.21
A_16_P15494798	232726390	232726449	ERO1LB	0.21	0.26	-0.42
A_16_P00280283	232732749	232732808	ERO1LB	-0.23	0.02	-0.47
A_16_P15494831	232739195	232739254	ERO1LB	-0.10	-0.40	-0.37
A_16_P35515248	232744911	232744970	ERO1LB	1.06	1.45	-0.25
A_16_P15494860	232758671	232758730	ERO1LB	0.19	1.12	-0.37
A_14_P119239	232768304	232768363	ERO1LB	0.17	0.66	-0.61
A_16_P15494898	232777879	232777938	chr1:232777879-232777938	0.39	0.56	-0.42
A_16_P00280343	232786317	232786376	chr1:232786317-232786376	0.28	0.73	-0.37
A_16_P15494955	232797621	232797680	chr1:232797621-232797680	0.61	0.10	-0.22
A_16_P00280402	232833147	232833206	chr1:232833147-232833206	0.84	0.72	-0.38
A_16_P15495066	232857524	232857583	chr1:232857524-232857583	0.96	0.87	-0.33
A_16_P15495076	232874337	232874396	chr1:232874337-232874396	0.39	1.55	-0.53
A_14_P114517	232880860	232880919	chr1:232880860-232880919	0.79	1.19	-0.31
A_16_P15495105	232888368	232888427	EDARADD	0.20	0.65	-0.35
A_14_P100752	232898579	232898638	EDARADD	0.84	1.43	-0.08
A_14_P130611	232916733	232916792	EDARADD	0.58	0.78	-0.15
A_16_P15495187	232923282	232923341	EDARADD	1.07	0.90	-0.42
A_16_P15495207	232936987	232937046	EDARADD	0.50	0.03	-0.41
A_16_P00280512	232945769	232945828	EDARADD	1.25	0.51	-0.14
A_14_P139557	232957560	232957611	EDARADD	0.39	0.48	-0.34
A_16_P15495288	232965604	232965663	EDARADD	0.43	0.66	-0.28
A_16_P15495312	232974363	232974422	chr1:232974363-232974422	0.53	1.33	0.08
A_16_P00280570	232983960	232984019	chr1:232983960-232984019	1.33	1.44	-0.25
A_16_P15495365	232994911	232994970	chr1:232994911-232994970	0.07	1.03	-0.42
A_16_P15495378	233000540	233000599	chr1:233000540-233000599	0.24	0.02	-0.22
A_16_P15495397	233007817	233007876	LGALS8	1.00	1.57	-0.15
A_14_P124152	233015339	233015398	LGALS8	0.04	0.87	-0.49
A_16_P15495431	233021029	233021088	LGALS8	0.76	0.19	-0.29
A_16_P00280631	233026725	233026784	LGALS8	0.52	1.10	-0.16
A_14_P136607	233032250	233032302	LGALS8	1.01	0.84	-0.06
A_14_P126950	233040135	233040194	HEATR1	0.73	0.67	-0.02
A_16_P00280675	233045423	233045482	HEATR1	0.75	1.05	-0.13
A_14_P129735	233055340	233055399	HEATR1	-0.06	0.20	-0.45
A_16_P35515998	233065684	233065743	HEATR1	0.56	1.32	-0.29
A_14_P134520	233074146	233074205	HEATR1	0.51	1.14	-0.41
A_16_P00280749	233081396	233081455	HEATR1	0.67	1.39	-0.26
A_16_P00280760	233087220	233087279	HEATR1	0.58	0.92	-0.42
A_16_P35516114	233098827	233098886	chr1:233098827-233098886	0.69	1.32	-0.22
A_16_P15495690	233108712	233108771	chr1:233108712-233108771	0.38	0.64	-0.38

A_16_P15495755	233138999	233139058	chr1:233138999-233139058	0.90	1.42	-0.20
A_16_P15495792	233152596	233152655	chr1:233152596-233152655	0.61	2.21	-0.31
A_16_P15495811	233163363	233163422	chr1:233163363-233163422	0.92	1.64	-0.35
A_14_P107547	233178297	233178356	ACTN2	1.08	1.70	-0.11
A_16_P15495867	233185817	233185876	ACTN2	0.65	0.83	-0.17
A_16_P15495887	233191474	233191533	ACTN2	0.49	1.36	-0.35
A_16_P00280918	233200341	233200398	ACTN2	0.99	0.16	-0.23
A_16_P00280932	233209498	233209557	ACTN2	0.15	0.75	-0.65
A_14_P105077	233215937	233215996	ACTN2	0.72	1.56	-0.28
A_16_P00280971	233227914	233227973	ACTN2	0.97	1.44	-0.43
A_14_P107463	233233941	233233988	ACTN2	0.49	0.70	-0.37
A_16_P15496032	233240739	233240798	ACTN2	0.67	0.88	-0.18
A_16_P15496071	233252596	233252655	ACTN2	0.89	1.07	-0.19
A_16_P15496084	233258467	233258526	chr1:233258467-233258526	0.21	0.61	-0.78
A_16_P00281033	233264183	233264242	chr1:233264183-233264242	0.46	1.09	-0.46
A_16_P15496122	233276255	233276314	chr1:233276255-233276314	-0.10	0.45	-0.56
A_14_P104849	233287372	233287431	MTR	0.63	1.07	-0.36
A_16_P15496187	233298258	233298317	MTR	0.47	0.35	-0.59
A_14_P125222	233304858	233304917	MTR	0.80	1.51	-0.22
A_16_P15496242	233318595	233318654	MTR	0.53	0.98	-0.14
A_14_P100845	233324996	233325055	MTR	1.25	1.27	-0.05
A_16_P00281151	233339818	233339877	MTR	0.71	1.39	-0.40
A_16_P15496343	233355051	233355110	MTR	0.69	1.00	-0.46
A_14_P135133	233363053	233363112	MTR	-0.09	1.63	-0.19
A_16_P35516876	233370518	233370577	MTR	0.36	0.43	-0.39
A_16_P35516912	233380409	233380468	MTR	0.61	1.52	-0.23
A_14_P132244	233386990	233387049	MTR	0.37	1.23	0.51
A_16_P00281251	233394165	233394224	chr1:233394165-233394224	-0.10	0.98	-0.37
A_16_P15496480	233403708	233403767	chr1:233403708-233403767	0.56	0.74	-0.37
A_16_P15496488	233412902	233412961	chr1:233412902-233412961	-0.13	0.80	-0.47
A_16_P15496538	233434591	233434650	chr1:233434591-233434650	0.91	1.22	-0.44
A_14_P122734	233453323	233453382	chr1:233453323-233453382	0.65	0.12	-0.30
A_16_P15496609	233461643	233461702	chr1:233461643-233461702	0.57	0.44	-0.49
A_16_P00281343	233467976	233468034	chr1:233467976-233468034	0.98	1.12	-0.15
A_16_P00281354	233475055	233475114	chr1:233475055-233475114	0.60	0.45	-0.65
A_16_P15496641	233480610	233480665	chr1:233480610-233480665	0.91	1.10	-0.49
A_16_P00281357	233487264	233487323	chr1:233487264-233487323	0.61	0.67	-0.33
A_16_P15496691	233501979	233502038	chr1:233501979-233502038	0.46	0.90	-0.65
A_16_P15496706	233518360	233518419	chr1:233518360-233518419	0.53	1.06	-0.33
A_16_P15496743	233531086	233531145	chr1:233531086-233531145	0.76	0.96	-0.39
A_16_P00281429	233538475	233538534	RYR2	1.26	0.96	-0.32
A_16_P00281455	233549650	233549709	RYR2	0.45	0.38	-0.52
A_16_P00281458	233557308	233557367	RYR2	0.22	1.36	-0.51
A_14_P100572	233574057	233574116	RYR2	-0.05	1.25	-0.13
A_16_P15496878	233583106	233583165	RYR2	1.03	1.14	-0.25
A_16_P00281513	233591743	233591802	RYR2	0.74	0.92	-0.49
A_16_P35517447	233605074	233605133	RYR2	0.53	0.46	-0.35
A_16_P00281551	233614481	233614540	RYR2	1.09	0.71	-0.27

A_16_P15496977	233622830	233622889	RYR2	0.34	1.13	-0.23
A_16_P00281572	233631658	233631717	RYR2	0.07	0.74	-0.19
A_16_P35517552	233649215	233649274	RYR2	0.28	1.11	-0.57
A_16_P15497090	233661417	233661476	RYR2	0.73	0.87	-0.46
A_16_P15497110	233668687	233668746	RYR2	0.17	0.53	-0.80
A_16_P15497136	233677289	233677348	RYR2	0.47	0.62	-0.58
A_16_P35517667	233688346	233688405	RYR2	0.81	0.98	-0.40
A_16_P15497193	233699656	233699715	RYR2	0.44	-0.05	-0.69
A_16_P15497218	233707914	233707973	RYR2	0.07	1.64	-0.42
A_16_P00281719	233718116	233718175	RYR2	0.73	1.17	-0.22
A_16_P15497265	233725748	233725807	RYR2	0.39	1.08	-0.49
A_16_P15497290	233736270	233736329	RYR2	0.43	1.14	-0.48
A_16_P35517810	233744389	233744448	RYR2	0.64	1.12	-0.48
A_16_P00281773	233752442	233752501	RYR2	0.42	1.11	-0.56
A_14_P109818	233759924	233759973	RYR2	0.06	0.53	-0.31
A_16_P15497364	233765990	233766049	RYR2	0.13	1.22	-0.50
A_16_P15497387	233772841	233772900	RYR2	0.25	0.54	-0.56
A_16_P00281819	233787455	233787514	RYR2	0.45	0.55	-0.42
A_16_P15497434	233795673	233795732	RYR2	-0.19	-0.72	-0.74
A_16_P35517950	233805708	233805767	RYR2	0.50	0.47	-0.69
A_16_P35517964	233811854	233811913	RYR2	0.55	1.31	-0.48
A_16_P15497504	233823013	233823072	RYR2	0.75	1.42	-0.44
A_16_P35518036	233833805	233833864	RYR2	0.47	0.79	-0.53
A_16_P00281911	233845662	233845721	RYR2	0.29	0.81	-0.49
A_16_P15497580	233851617	233851676	RYR2	0.32	0.94	-0.50
A_16_P35518102	233857082	233857141	RYR2	0.20	0.81	-0.61
A_16_P15497637	233869230	233869289	RYR2	0.06	1.08	-0.65
A_16_P15497693	233885402	233885461	RYR2	1.02	1.52	-0.41
A_16_P35518212	233894931	233894990	RYR2	0.48	0.86	-0.40
A_16_P00282017	233906885	233906944	RYR2	-0.28	0.56	-0.03
A_16_P35518260	233912588	233912647	RYR2	0.03	1.25	-0.54
A_14_P137572	233930710	233930769	RYR2	0.65	1.05	-0.53
A_16_P15497823	233936641	233936700	RYR2	-0.09	0.21	-0.69
A_16_P15497865	233950507	233950566	RYR2	0.27	0.73	-0.45
A_16_P15497894	233959620	233959679	RYR2	0.63	1.36	-0.48
A_16_P15497937	233972464	233972523	RYR2	0.64	0.74	-0.51
A_16_P15497974	233985796	233985855	RYR2	0.38	0.83	-0.54
A_16_P00282182	233996063	233996122	RYR2	0.53	0.62	-0.48
A_16_P35518547	234002626	234002685	RYR2	-0.03	1.17	-0.67
A_16_P15498038	234012376	234012435	RYR2	-0.01	0.50	-0.55
A_16_P15498048	234019703	234019762	RYR2	0.65	0.74	-0.46
A_16_P00282222	234033091	234033150	RYR2	0.58	1.20	-0.50
A_16_P00282236	234040001	234040060	RYR2	-0.30	1.02	-0.12
A_16_P35518649	234054139	234054198	RYR2	0.42	0.55	-0.30
A_16_P15498154	234062404	234062463	RYR2	-0.38	1.20	-0.78
A_16_P15498170	234069060	234069119	RYR2	-0.09	-0.24	-0.71
A_16_P00282292	234079298	234079357	RYR2	0.25	0.71	-0.60
A_16_P15498218	234091208	234091267	RYR2	0.50	1.08	-0.49

A_16_P15498225	234098167	234098226	RYR2	0.11	0.81	-0.42
A_16_P00282323	234104190	234104249	RYR2	1.04	0.68	-0.31
A_16_P15498260	234113140	234113197	RYR2	0.35	1.03	-0.25
A_16_P15498288	234120875	234120934	RYR2	0.91	-0.39	-0.39
A_16_P15498323	234132622	234132681	RYR2	0.10	1.23	-0.48
A_14_P131985	234143705	234143764	RYR2	0.29	0.92	-0.58
A_16_P00282412	234150123	234150182	RYR2	0.16	1.22	-0.87
A_16_P00282427	234157239	234157298	RYR2	0.03	0.77	-0.71
A_16_P00282454	234169796	234169855	RYR2	0.01	0.29	-0.46
A_16_P00282468	234176813	234176872	RYR2	0.58	1.13	-0.58
A_16_P00282487	234188289	234188348	RYR2	0.17	0.97	-0.74
A_16_P15498536	234198152	234198211	RYR2	0.06	0.58	-0.81
A_16_P35519149	234212482	234212541	RYR2	1.39	0.93	-0.45
A_16_P15498620	234223188	234223247	RYR2	0.42	1.58	-0.50
A_16_P15498655	234236242	234236301	RYR2	0.02	0.91	-0.56
A_16_P00282610	234249093	234249152	RYR2	0.48	0.75	-0.56
A_16_P15498725	234255607	234255666	RYR2	0.01	0.04	-0.44
A_16_P00282643	234262949	234263008	RYR2	0.31	1.31	-0.52
A_16_P35519359	234270853	234270912	RYR2	-0.05	1.11	-0.70
A_16_P15498810	234280738	234280797	RYR2	0.59	0.88	-0.39
A_16_P15498830	234287509	234287568	RYR2	0.07	1.18	-1.01
A_16_P00282712	234295497	234295556	RYR2	0.63	1.32	-0.58
A_16_P00282740	234308348	234308407	RYR2	0.73	1.33	-0.38
A_16_P15498902	234316520	234316579	RYR2	0.23	0.34	-0.24
A_14_P129287	234322138	234322197	RYR2	0.19	0.35	-0.02
A_16_P35519532	234335017	234335076	chr1:234335017-234335076	0.28	1.23	-0.56
A_16_P15498951	234342651	234342710	chr1:234342651-234342710	0.49	1.23	-0.44
A_16_P00282791	234354532	234354591	AL122093	0.13	0.48	-0.88
A_16_P00282800	234360937	234360996	AL122093	0.48	1.11	-0.45
A_16_P00282810	234366762	234366821	AL122093	-0.41	0.00	-0.70
A_14_P131826	234372147	234372206	ZP4	0.12	0.52	-0.39
A_16_P15499041	234376206	234376265	ZP4	0.60	0.88	-0.56
A_16_P00282848	234385718	234385777	AL122093	0.29	0.94	-0.33
A_16_P15499081	234390315	234390374	AL122093	0.79	1.06	-0.49
A_16_P00282866	234395720	234395779	AL122093	1.03	1.62	-0.31
A_16_P15499136	234414898	234414957	AL122093	0.30	0.66	-0.61
A_14_P134444	234442054	234442113	chr1:234442054-234442113	0.98	1.48	-0.49
A_16_P15499357	234489634	234489693	chr1:234489634-234489693	0.34	1.28	-0.55
A_16_P15499415	234510380	234510439	chr1:234510380-234510439	0.72	1.76	-0.33
A_16_P15499465	234550361	234550420	chr1:234550361-234550420	0.54	0.61	-0.36
A_16_P35520103	234575608	234575667	chr1:234575608-234575667	0.58	1.40	-0.41
A_14_P105214	234610264	234610323	chr1:234610264-234610323	0.21	1.68	-0.49
A_16_P15499695	234642500	234642559	chr1:234642500-234642559	0.22	1.12	-0.57
A_16_P15499788	234694492	234694551	chr1:234694492-234694551	0.07	0.87	-0.72
A_16_P15499872	234737816	234737875	chr1:234737816-234737875	0.63	0.57	-0.62
A_16_P35520498	234750815	234750874	chr1:234750815-234750874	0.10	-0.24	-0.67
A_16_P00283349	234760702	234760761	chr1:234760702-234760761	0.34	1.49	-0.59
A_16_P15499950	234776770	234776829	chr1:234776770-234776829	0.46	1.83	-0.58

A_16_P15499996	234796612	234796671	chr1:234796612-234796671	0.12	0.69	-0.57
A_16_P15500108	234840462	234840521	chr1:234840462-234840521	0.02	0.32	-0.66
A_14_P118567	234901433	234901492	chr1:234901433-234901492	-0.13	0.81	-0.69
A_16_P15500291	234927550	234927609	chr1:234927550-234927609	0.43	1.00	-0.51
A_16_P15500388	234958478	234958537	chr1:234958478-234958537	0.33	-0.41	-0.52
A_16_P35521023	234976939	234976998	AK091229	0.60	0.63	-0.53
A_16_P15500457	234985064	234985123	AK091229	0.33	0.84	-0.66
A_16_P00283659	234991538	234991597	AK091229	0.25	0.87	-0.71
A_16_P35521081	235002293	235002352	AK091229	0.26	0.57	-0.50
A_16_P15500549	235024713	235024772	AK091229	0.25	0.46	-0.84
A_16_P15500623	235050155	235050214	chr1:235050155-235050214	0.45	0.34	-0.64
A_16_P00283794	235079469	235079528	chr1:235079469-235079528	0.80	1.29	-0.69
A_14_P131114	235107559	235107618	chr1:235107559-235107618	-0.14	0.91	-0.49
A_16_P00283881	235143510	235143569	chr1:235143510-235143569	0.11	0.66	-0.61
A_16_P15500942	235191300	235191359	chr1:235191300-235191359	-0.22	1.22	-0.77
A_14_P110149	235214911	235214970	chr1:235214911-235214970	0.28	1.78	-0.88
A_16_P15501031	235243219	235243278	chr1:235243219-235243278	0.28	0.73	-0.62
A_16_P15501126	235288177	235288236	chr1:235288177-235288236	0.68	0.52	-0.42
A_16_P35521744	235314463	235314522	chr1:235314463-235314522	0.09	0.63	-0.62
A_14_P139812	235337043	235337102	chr1:235337043-235337102	0.05	1.42	-0.66
A_16_P15501328	235382961	235383020	chr1:235382961-235383020	0.64	1.54	-0.14
A_16_P15501376	235405226	235405285	chr1:235405226-235405285	0.31	1.03	-0.44
A_16_P15501461	235443923	235443982	chr1:235443923-235443982	0.50	0.50	-0.60
A_16_P15501532	235474858	235474917	chr1:235474858-235474917	0.70	1.08	-0.38
A_16_P15501656	235517432	235517491	chr1:235517432-235517491	0.75	1.42	-0.52
A_16_P15501731	235552121	235552180	chr1:235552121-235552180	-0.16	0.96	-0.55
A_16_P35522314	235575241	235575300	chr1:235575241-235575300	0.30	1.13	-0.66
A_14_P129837	235618094	235618153	chr1:235618094-235618153	-0.25	1.08	-0.57
A_16_P15501997	235665599	235665658	chr1:235665599-235665658	0.18	0.57	-0.27
A_16_P15502061	235701499	235701558	chr1:235701499-235701558	0.33	0.71	-0.69
A_16_P15502132	235738402	235738461	AL162037	0.39	0.88	-0.56
A_14_P120118	235785790	235785849	chr1:235785790-235785849	0.44	0.58	-0.68
A_16_P00284772	235822988	235823047	chr1:235822988-235823047	0.74	0.53	-0.67
A_16_P35522984	235846802	235846861	chr1:235846802-235846861	0.60	0.32	-0.57
A_16_P00284884	235884391	235884450	AK056349	0.52	0.69	-0.40
A_16_P15502584	235907259	235907318	AK056349	0.44	1.11	-0.80
A_16_P15502615	235916087	235916146	AK056349	0.46	0.48	-0.47
A_14_P123686	235930233	235930292	AK056349	0.50	1.01	-0.74
A_16_P15502699	235941737	235941796	AK056349	0.65	0.56	-0.47
A_16_P15502733	235955021	235955080	AK056349	0.80	1.37	-0.40
A_16_P15502758	235965863	235965922	AK056349	0.20	0.99	-0.52
A_16_P00285051	235975496	235975555	AK056349	0.76	0.95	-0.64
A_16_P15502810	235986302	235986361	AK056349	1.15	1.14	-0.35
A_16_P00285087	236001129	236001188	AK056349	0.16	0.74	-0.26
A_16_P15502856	236010273	236010332	AK056349	0.51	1.63	-0.47
A_16_P15502880	236019078	236019137	AK056349	0.54	0.18	-0.46
A_16_P15502909	236029590	236029649	AK056349	0.13	0.93	-0.39
A_16_P15502969	236047771	236047830	AK056349	0.68	1.00	-0.57

A_16_P35523580	236061866	236061925	AK056349	0.35	0.05	-0.83
A_16_P15503053	236073259	236073318	AK056349	0.51	0.16	-0.54
A_16_P15503083	236083003	236083062	AK056349	0.66	0.02	-0.58
A_16_P15503124	236097390	236097449	AK056349	0.28	0.72	-0.32
A_16_P00285282	236106689	236106748	AK056349	0.54	0.73	-0.50
A_14_P127307	236121182	236121241	CHRM3	0.02	1.08	-0.67
A_16_P00285310	236134663	236134722	CHRM3	0.50	0.34	-0.56
A_16_P15503233	236147009	236147068	CHRM3	0.27	0.76	-0.59
A_16_P00285353	236158201	236158260	CHRM3	0.84	1.07	-0.46
A_14_P135163	236167559	236167616	CHRM3	0.32	0.62	-0.35
A_16_P00285399	236181305	236181364	CHRM3	0.82	0.43	-0.20
A_16_P15503375	236191196	236191255	CHRM3	0.62	0.49	-0.65
A_16_P35523954	236196757	236196816	CHRM3	0.12	0.19	-0.61
A_16_P35523996	236211405	236211464	CHRM3	0.28	1.06	-0.57
A_16_P00285474	236221445	236221504	CHRM3	0.32	0.53	-0.52
A_16_P15503498	236232320	236232379	CHRM3	0.31	1.07	-0.45
A_16_P15503516	236238142	236238201	CHRM3	0.02	0.80	-0.60
A_16_P15503549	236248413	236248472	CHRM3	0.10	0.17	-0.36
A_14_P122908	236253984	236254043	CHRM3	0.39	0.23	-0.62
A_16_P15503583	236259768	236259827	CHRM3	0.47	0.96	-0.34
A_16_P00285559	236266412	236266471	CHRM3	0.42	1.88	-0.49
A_16_P15503617	236274333	236274392	CHRM3	0.17	1.56	-0.52
A_16_P15503627	236280081	236280138	CHRM3	0.36	1.62	-0.46
A_16_P00285587	236285731	236285790	CHRM3	0.69	0.94	-0.48
A_16_P00285599	236292294	236292353	CHRM3	0.36	1.14	-0.44
A_16_P00285613	236298203	236298262	CHRM3	0.48	0.97	-0.55
A_16_P15503730	236309060	236309119	CHRM3	-0.03	0.64	-0.34
A_14_P121189	236316659	236316718	CHRM3	-0.08	0.67	-0.78
A_16_P15503779	236326597	236326656	CHRM3	0.35	0.33	-0.46
A_16_P15503822	236338780	236338839	CHRM3	0.19	0.86	-0.69
A_16_P15503842	236344659	236344718	CHRM3	0.37	1.25	-0.29
A_16_P15503866	236353099	236353158	CHRM3	1.11	1.25	-0.39
A_16_P15503894	236362007	236362066	CHRM3	0.38	0.73	-0.56
A_16_P15503933	236376930	236376989	CHRM3	0.47	0.80	-0.48
A_16_P35524518	236389415	236389474	CHRM3	0.53	0.72	-0.38
A_14_P125847	236397499	236397558	CHRM3	0.50	1.46	-0.42
A_14_P201069	236402753	236402812	AK095715	0.85	0.88	-0.38
A_16_P00285819	236410240	236410299	chr1:236410240-236410299	0.54	0.65	-0.37
A_16_P35524619	236429679	236429738	chr1:236429679-236429738	0.21	0.92	-0.93
A_16_P15504145	236460825	236460884	chr1:236460825-236460884	0.51	1.14	-0.06
A_16_P00285902	236479301	236479360	chr1:236479301-236479360	0.73	0.33	-0.75
A_16_P00285912	236489022	236489081	chr1:236489022-236489081	0.40	1.05	-0.32
A_16_P35524784	236495811	236495870	chr1:236495811-236495870	0.69	1.22	-0.55
A_16_P00285946	236509868	236509927	chr1:236509868-236509927	0.54	1.09	-0.25
A_16_P15504291	236517516	236517575	chr1:236517516-236517575	0.29	0.96	-0.55
A_16_P35524877	236536926	236536985	chr1:236536926-236536985	0.25	0.57	-0.61
A_16_P00286015	236557732	236557791	chr1:236557732-236557791	0.37	1.25	-0.40
A_16_P00286023	236569883	236569942	chr1:236569883-236569942	0.75	1.34	-0.27

A_16_P15504433	236580715	236580774	chr1:236580715-236580774	0.93	1.37	-0.34
A_16_P15504464	236589334	236589393	FMN2	-0.03	-0.54	-0.66
A_16_P15504485	236595554	236595613	FMN2	0.54	1.03	-0.31
A_16_P35525044	236603176	236603235	FMN2	0.66	1.96	-0.67
A_16_P00286089	236613917	236613976	FMN2	0.30	-0.04	-0.36
A_16_P00286103	236621107	236621166	FMN2	1.20	1.22	-0.23
A_16_P00286116	236629728	236629787	FMN2	0.55	0.71	-0.62
A_14_P112344	236639745	236639804	FMN2	0.01	0.45	-0.81
A_16_P15504624	236648090	236648149	FMN2	0.13	0.48	-0.48
A_16_P00286154	236655148	236655207	FMN2	1.05	1.00	-0.40
A_16_P15504650	236662071	236662130	FMN2	0.23	0.52	-0.45
A_16_P15504679	236672037	236672096	FMN2	0.68	1.16	-0.28
A_16_P15504709	236680852	236680911	FMN2	0.51	0.89	-0.59
A_16_P00286235	236700534	236700593	FMN2	0.31	-0.48	-0.32
A_16_P15504799	236708811	236708870	FMN2	0.21	0.94	-0.38
A_16_P00286257	236714603	236714660	FMN2	0.86	0.61	-0.24
A_16_P15504834	236726467	236726526	FMN2	0.41	0.57	-0.54
A_16_P15504860	236733785	236733844	FMN2	0.08	1.42	-0.55
A_16_P00286305	236747316	236747375	FMN2	-0.04	0.22	-0.63
A_16_P35525436	236753091	236753150	FMN2	0.73	1.01	-0.48
A_16_P35525454	236762165	236762224	FMN2	0.44	0.58	-0.67
A_16_P15504927	236769018	236769077	FMN2	0.50	1.67	-0.24
A_16_P15504959	236779605	236779664	FMN2	-0.07	0.38	-0.90
A_16_P00286368	236785567	236785626	FMN2	-0.07	-0.04	-0.38
A_16_P15504991	236792047	236792102	FMN2	0.40	0.15	-0.53
A_16_P35525544	236799553	236799612	FMN2	0.68	1.18	-0.47
A_16_P15505020	236805149	236805208	FMN2	0.43	0.86	-0.39
A_16_P15505049	236817124	236817183	FMN2	0.63	0.80	-0.42
A_16_P00286429	236823235	236823294	FMN2	-0.40	0.10	-0.73
A_16_P35525648	236833659	236833718	FMN2	0.41	0.71	-0.45
A_16_P35525678	236845111	236845170	FMN2	0.27	1.46	-0.55
A_16_P15505159	236854359	236854418	FMN2	0.11	0.80	-0.49
A_16_P15505177	236864897	236864956	FMN2	0.46	0.51	-0.49
A_16_P00286509	236881872	236881931	FMN2	0.64	0.82	-0.42
A_16_P15505216	236887913	236887972	FMN2	0.56	0.92	-0.51
A_16_P35525795	236897019	236897078	FMN2	0.86	1.21	-0.69
A_16_P15505265	236903885	236903944	FMN2	0.51	1.14	-0.54
A_16_P00286562	236914909	236914968	FMN2	0.46	1.15	-0.89
A_16_P15505308	236925246	236925305	FMN2	1.00	0.82	-0.52
A_16_P35525879	236931853	236931912	FMN2	0.48	1.26	-0.31
A_16_P15505347	236937318	236937377	FMN2	0.07	1.03	-0.61
A_16_P15505370	236944769	236944828	FMN2	0.03	0.53	-0.43
A_16_P00286624	236954463	236954522	FMN2	0.03	1.08	-0.67
A_14_P200826	236964184	236964243	FMN2	0.03	1.18	-0.44
A_16_P15505456	236972844	236972903	chr1:236972844-236972903	0.56	0.78	-0.50
A_14_P126309	236980841	236980900	GREM2	1.01	1.37	-0.37
A_16_P15505496	236987523	236987582	GREM2	-0.15	0.80	-0.58
A_16_P35526093	237001956	237002015	GREM2	0.37	0.42	-0.66

A_14_P136171	237007506	237007565	GREM2	0.74	-0.63	-0.72
A_16_P15505577	237016470	237016529	GREM2	-0.17	0.30	-0.54
A_16_P35526163	237027223	237027282	GREM2	0.32	0.78	-0.48
A_16_P15505656	237042779	237042838	GREM2	0.35	1.02	-0.74
A_14_P119050	237050563	237050622	GREM2	0.10	0.94	-0.33
A_16_P00286800	237059890	237059949	GREM2	0.31	0.66	0.11
A_16_P15505710	237066422	237066481	GREM2	0.05	0.46	-0.01
A_16_P15505727	237072850	237072909	GREM2	0.36	0.08	-0.62
A_14_P124100	237081820	237081879	GREM2	0.17	1.65	-0.57
A_16_P00286856	237092655	237092714	GREM2	0.30	0.62	-0.58
A_14_P139701	237099373	237099432	GREM2	0.53	1.17	-0.49
A_16_P15505834	237110716	237110775	chr1:237110716-237110775	0.98	1.09	-0.39
A_16_P00286895	237117930	237117989	chr1:237117930-237117989	0.34	1.25	-0.33
A_16_P15505882	237137098	237137157	chr1:237137098-237137157	0.46	0.77	-0.42
A_16_P15506041	237197316	237197375	chr1:237197316-237197375	0.42	1.32	-0.57
A_14_P132226	237226763	237226822	chr1:237226763-237226822	0.20	0.33	-0.55
A_16_P00287104	237258401	237258460	chr1:237258401-237258460	-0.05	0.86	-0.34
A_14_P137062	237265039	237265098	RGS7	0.32	1.36	-0.75
A_16_P15506223	237270584	237270643	RGS7	0.81	0.46	-0.33
A_16_P35526804	237281108	237281167	RGS7	0.59	0.93	-0.31
A_16_P00287164	237290787	237290846	RGS7	0.18	1.70	-0.74
A_16_P00287179	237297028	237297087	RGS7	0.61	0.27	-0.50
A_16_P00287192	237304082	237304141	RGS7	0.06	1.16	-0.65
A_16_P15506345	237309718	237309777	RGS7	0.49	1.43	-0.34
A_16_P00287217	237316457	237316515	RGS7	0.78	1.35	-0.47
A_16_P15506406	237330388	237330447	RGS7	-0.22	-0.24	-0.69
A_16_P00287256	237342839	237342898	RGS7	-0.11	0.85	-0.47
A_16_P35527018	237351709	237351768	RGS7	0.17	0.33	-0.57
A_14_P106422	237357977	237358036	RGS7	0.47	1.21	-0.45
A_16_P15506509	237368545	237368604	RGS7	0.65	1.36	-0.28
A_16_P15506527	237375975	237376034	RGS7	0.89	1.20	-0.30
A_16_P15506544	237382549	237382608	RGS7	0.87	0.88	-0.20
A_16_P15506554	237391456	237391515	RGS7	0.68	0.87	-0.59
A_16_P15506580	237398833	237398892	RGS7	0.39	-0.28	-0.39
A_16_P35527166	237413064	237413123	RGS7	0.39	1.54	-0.36
A_16_P15506625	237420109	237420168	RGS7	1.09	1.22	-0.54
A_16_P35527203	237425891	237425950	RGS7	0.77	0.39	-0.39
A_16_P00287401	237437629	237437688	RGS7	0.43	0.86	-0.33
A_16_P15506685	237445350	237445409	RGS7	0.60	0.82	-0.49
A_16_P15506705	237454465	237454524	RGS7	0.47	1.67	-0.48
A_16_P35527292	237461227	237461286	RGS7	0.31	1.64	-0.18
A_16_P15506764	237472418	237472477	RGS7	0.26	1.56	-0.62
A_16_P35527357	237480019	237480078	RGS7	-0.05	0.18	-0.64
A_16_P15506802	237489855	237489914	RGS7	-0.31	1.20	-0.52
A_16_P15506816	237495470	237495529	RGS7	0.52	1.16	-0.28
A_16_P15506837	237504102	237504161	RGS7	0.26	1.63	-0.54
A_16_P15506849	237510869	237510928	RGS7	0.23	0.54	-0.44
A_16_P15506877	237523603	237523662	RGS7	0.28	0.20	-0.25

A_16_P00287516	237529376	237529435	RGS7	0.92	1.14	-0.62
A_16_P15506914	237536763	237536822	RGS7	0.25	1.14	-0.59
A_16_P15506929	237543487	237543546	RGS7	0.58	1.32	-0.44
A_16_P35527509	237551722	237551781	RGS7	0.15	0.41	-0.48
A_16_P15506984	237565700	237565759	RGS7	0.17	0.61	-0.68
A_16_P15507006	237572736	237572795	RGS7	0.32	-0.34	-0.46
A_14_P126683	237588050	237588109	RGS7	-0.23	-0.48	0.18
A_16_P00287626	237598230	237598289	RGS7	0.61	0.55	-0.38
A_16_P15507112	237606547	237606606	RGS7	0.22	0.78	-0.54
A_16_P15507147	237619565	237619624	RGS7	0.63	0.43	-0.50
A_16_P00287678	237629134	237629193	RGS7	0.17	1.00	-0.51
A_16_P15507197	237637004	237637063	RGS7	0.20	0.59	-0.45
A_16_P15507232	237648753	237648812	RGS7	0.44	1.11	0.03
A_16_P15507245	237654227	237654286	RGS7	0.93	0.64	-0.35
A_16_P00287728	237663311	237663370	RGS7	-0.12	1.11	-0.73
A_16_P35527851	237669045	237669104	RGS7	0.36	0.45	-0.46
A_16_P15507313	237683252	237683311	RGS7	0.71	1.65	-0.47
A_16_P00287765	237693605	237693664	RGS7	0.68	0.63	-0.38
A_16_P00287782	237706848	237706907	RGS7	0.77	0.89	-0.25
A_16_P15507408	237720424	237720483	RGS7	0.11	1.11	-0.50
A_16_P15507447	237736557	237736616	RGS7	0.43	0.52	-0.26
A_16_P00287833	237742539	237742598	RGS7	0.98	1.25	-0.39
A_16_P15507493	237752023	237752082	RGS7	0.88	1.36	-0.50
A_16_P15507508	237758451	237758510	RGS7	0.70	0.29	-0.30
A_14_P138338	237765962	237766021	RGS7	-0.15	1.61	-0.59
A_16_P15507544	237776483	237776542	RGS7	0.77	0.84	-0.34
A_16_P00287905	237787257	237787316	RGS7	1.25	1.34	-0.27
A_16_P15507599	237797652	237797711	RGS7	0.56	1.51	-0.19
A_16_P15507623	237808280	237808339	RGS7	0.22	0.14	-0.70
A_16_P15507656	237818449	237818508	RGS7	0.08	1.15	-0.10
A_16_P35528234	237823955	237824014	RGS7	0.77	1.21	-0.33
A_16_P00287975	237830448	237830507	RGS7	0.27	1.46	-0.38
A_14_P130762	237848072	237848131	chr1:237848072-237848131	0.86	1.62	-0.39
A_16_P00288016	237857547	237857606	chr1:237857547-237857606	1.42	1.11	-0.47
A_16_P15507788	237868752	237868811	chr1:237868752-237868811	0.12	0.59	-0.62
A_16_P15507873	237902435	237902494	chr1:237902435-237902494	0.13	0.69	-0.41
A_16_P15507931	237934226	237934285	chr1:237934226-237934285	0.50	0.80	-0.45
A_16_P15507960	237955764	237955823	chr1:237955764-237955823	0.68	1.10	-0.19
A_16_P35528585	237977315	237977374	chr1:237977315-237977374	0.25	0.98	-0.38
A_14_P120277	237984427	237984486	chr1:237984427-237984486	1.08	1.65	-0.39
A_16_P15508062	237995306	237995365	FH	0.26	1.54	-0.43
A_14_P127149	238006592	238006651	FH	0.37	0.77	-0.48
A_16_P15508118	238014872	238014931	chr1:238014872-238014931	0.54	1.05	-0.39
A_16_P00288225	238021752	238021811	KMO	0.45	0.92	-0.40
A_14_P115134	238030826	238030885	KMO	0.74	1.12	-0.17
A_16_P35528761	238040261	238040320	KMO	0.72	1.24	-0.42
A_14_P120282	238055850	238055909	KMO	0.20	1.39	0.04
A_16_P35528821	238073082	238073141	KMO	-0.21	1.11	-0.65

A_14_P110695	238083225	238083284	KMO	0.65	0.53	-0.28
A_16_P00288330	238093585	238093644	OPN3	0.82	0.76	-0.36
A_16_P00288354	238106739	238106798	OPN3	0.67	0.88	-0.38
A_16_P15508364	238114458	238114517	OPN3	0.38	1.50	-0.41
A_16_P35528979	238119984	238120043	OPN3	0.51	0.74	-0.49
A_14_P100448	238125127	238125186	OPN3	0.89	1.33	-0.10
A_16_P00288408	238135841	238135900	chr1:238135841-238135900	0.82	0.96	-0.57
A_16_P15508449	238144738	238144797	AK122927	0.30	0.43	-0.46
A_16_P15508461	238150380	238150439	AK122927	0.42	0.74	-0.20
A_16_P15508496	238163327	238163386	AK122927	1.03	0.53	-0.30
A_16_P00288473	238176825	238176884	WDR64	0.14	1.07	-0.55
A_16_P35529148	238184041	238184100	WDR64	0.62	1.17	-0.33
A_16_P15508554	238190432	238190491	WDR64	0.38	0.85	-0.48
A_16_P00288499	238198723	238198782	WDR64	0.17	0.67	-0.54
A_16_P15508597	238205402	238205461	WDR64	0.69	0.43	-0.34
A_16_P15508611	238211873	238211932	WDR64	-0.06	0.84	-0.30
A_16_P35529249	238221821	238221880	WDR64	0.87	0.50	-0.26
A_16_P15508670	238230911	238230970	WDR64	0.35	0.77	-0.30
A_16_P15508694	238239798	238239857	WDR64	0.66	1.31	-0.29
A_16_P15508706	238246592	238246651	WDR64	0.39	0.32	-0.39
A_14_P130699	238262106	238262165	WDR64	1.22	0.98	-0.26
A_16_P35529400	238277118	238277177	WDR64	0.76	0.47	-0.35
A_16_P35529400	238277118	238277177	WDR64	0.78	0.34	-0.30
A_16_P35529400	238277118	238277177	WDR64	0.71	0.39	-0.33
A_16_P15508806	238285549	238285608	WDR64	0.72	1.12	-0.41
A_16_P35529453	238298063	238298122	chr1:238298063-238298122	0.44	0.76	-0.44
A_16_P15508848	238304747	238304806	chr1:238304747-238304806	0.06	1.19	-0.48
A_14_P103442	238314315	238314374	chr1:238314315-238314374	0.44	3.05	-0.35
A_16_P15508901	238322547	238322606	chr1:238322547-238322606	0.53	1.28	-0.36
A_16_P15508929	238331514	238331573	chr1:238331514-238331573	0.37	1.05	-0.52
A_14_P120822	238338324	238338383	EXO1	0.53	1.05	-0.44
A_14_P137216	238349959	238350018	EXO1	0.07	1.92	-0.26
A_16_P00288760	238364218	238364277	EXO1	0.20	0.61	-0.31
A_14_P136592	238371238	238371297	EXO1	-0.17	1.32	-0.40
A_16_P35529688	238379001	238379060	EXO1	0.12	1.34	-0.28
A_16_P00288810	238392679	238392738	chr1:238392679-238392738	0.38	1.25	-0.44
A_14_P132920	238420936	238420995	chr1:238420936-238420995	0.51	1.26	0.08
A_16_P00288853	238431864	238431923	chr1:238431864-238431923	0.13	0.80	-0.33
A_16_P15509207	238445612	238445671	chr1:238445612-238445671	1.33	1.14	-0.12
A_16_P15509221	238456482	238456541	chr1:238456482-238456541	0.91	1.15	0.00
A_16_P15509234	238462617	238462676	chr1:238462617-238462676	0.25	0.82	-0.46
A_16_P00288884	238467453	238467512	chr1:238467453-238467512	0.75	0.76	-0.30
A_16_P15509246	238471979	238472038	chr1:238471979-238472038	0.09	0.65	-0.41
A_16_P00288897	238477630	238477689	chr1:238477630-238477689	0.96	1.01	-0.18
A_16_P00288903	238482587	238482632	chr1:238482587-238482632	0.90	0.56	0.04
A_16_P15509285	238488166	238488225	MAP1LC3C	0.70	1.45	-0.59
A_16_P00288922	238499123	238499182	chr1:238499123-238499182	0.98	1.08	-0.30
A_16_P15509324	238507862	238507921	chr1:238507862-238507921	0.78	-0.05	-0.35

A_16_P00288948	238522971	238523030	chr1:238522971-238523030	0.45	1.38	-0.12
A_16_P35530014	238531296	238531343	chr1:238531296-238531343	0.57	0.44	-0.06
A_16_P15509372	238549271	238549321	chr1:238549271-238549321	1.00	0.21	-0.51
A_16_P35530043	238559055	238559114	chr1:238559055-238559114	0.33	0.75	-0.28
A_16_P00288983	238566211	238566270	chr1:238566211-238566270	0.74	1.01	-0.30
A_14_P124263	238578201	238578260	AK091691	-0.16	1.56	-0.45
A_16_P00289024	238590049	238590108	PLD5	0.37	1.19	-0.33
A_16_P00289039	238596969	238597028	PLD5	0.35	1.06	-0.55
A_16_P00289051	238603299	238603358	PLD5	-0.31	0.10	-0.54
A_14_P124163	238613805	238613864	PLD5	0.55	1.07	-0.35
A_16_P15509583	238626825	238626884	PLD5	0.37	1.11	-0.26
A_16_P15509610	238635597	238635656	PLD5	0.35	0.85	-0.53
A_16_P15509620	238651073	238651132	PLD5	0.45	1.37	-0.35
A_16_P00289121	238667792	238667851	PLD5	0.24	1.71	-0.08
A_16_P15509661	238675010	238675069	PLD5	0.44	1.00	-0.46
A_16_P00289148	238683249	238683308	PLD5	0.15	-0.11	-0.59
A_16_P15509703	238690311	238690370	PLD5	0.40	0.55	-0.48
A_16_P35530384	238697905	238697964	PLD5	0.91	0.67	-0.42
A_16_P35530401	238710572	238710631	PLD5	1.36	0.88	-0.16
A_16_P35530421	238717409	238717468	PLD5	0.62	0.97	-0.26
A_16_P00289198	238724605	238724664	PLD5	0.68	0.64	-0.50
A_14_P123812	238730140	238730199	PLD5	0.35	1.76	-0.41
A_16_P35530505	238747201	238747247	PLD5	0.15	0.95	0.22
A_16_P00289236	238756044	238756100	PLD5	0.46	1.34	0.51
A_16_P00289240	238768139	238768198	PLD5	0.33	0.67	-0.48
A_16_P00289246	238776783	238776842	PLD5	-0.19	0.71	-0.53
A_16_P00289255	238796201	238796260	PLD5	-0.56	0.40	-0.38
A_16_P00289259	238803290	238803346	PLD5	0.61	0.29	-0.29
A_16_P15510015	238812507	238812566	PLD5	-0.29	0.93	-0.47
A_16_P15510052	238825151	238825210	PLD5	-0.40	0.19	-0.76
A_16_P15510091	238838948	238839005	PLD5	0.79	1.13	-0.25
A_16_P15510099	238848898	238848957	PLD5	0.54	0.46	-0.28
A_16_P15510126	238859201	238859260	PLD5	0.78	0.59	-0.37
A_16_P35530820	238869177	238869236	PLD5	0.01	1.09	-0.62
A_16_P35530842	238880862	238880921	PLD5	0.41	1.42	-0.16
A_16_P15510187	238887731	238887790	PLD5	0.48	0.72	0.18
A_14_P109328	238899912	238899971	PLD5	0.35	1.77	-0.35
A_16_P00289359	238909334	238909393	PLD5	0.32	0.62	-0.52
A_16_P00289380	238921604	238921663	PLD5	0.25	0.91	-0.59
A_16_P15510296	238929805	238929864	PLD5	1.14	0.73	-0.30
A_16_P15510338	238942881	238942940	PLD5	0.07	-0.03	-0.50
A_16_P35531014	238949805	238949864	PLD5	0.97	0.31	-0.30
A_16_P15510354	238956678	238956737	PLD5	-0.17	-0.01	-0.66
A_16_P00289443	238964837	238964896	PLD5	0.69	0.37	-0.39
A_14_P133225	238982501	238982560	PLD5	0.29	1.06	-0.61
A_14_P133225	238982501	238982560	PLD5	0.24	0.48	-0.62
A_14_P133225	238982501	238982560	PLD5	0.42	0.49	-0.58
A_16_P15510426	238991704	238991763	PLD5	0.32	1.34	-0.20

A_16_P00289487	238999444	238999503	PLD5	0.94	0.92	0.04
A_16_P00289498	239007513	239007572	PLD5	0.69	0.74	-0.59
A_16_P15510508	239020095	239020154	chr1:239020095-239020154	0.38	1.05	-0.60
A_16_P00289546	239029738	239029797	chr1:239029738-239029797	0.65	0.72	-0.64
A_16_P35531259	239055515	239055574	chr1:239055515-239055574	0.72	1.41	-0.49
A_14_P125293	239098296	239098355	chr1:239098296-239098355	0.32	0.51	-0.35
A_16_P15510745	239133727	239133786	chr1:239133727-239133786	0.82	0.94	-1.00
A_16_P15510800	239156984	239157043	chr1:239156984-239157043	0.43	0.76	-0.45
A_16_P15510872	239190321	239190380	chr1:239190321-239190380	0.31	0.14	-0.63
A_16_P15510942	239215651	239215710	chr1:239215651-239215710	0.41	0.63	-0.60
A_16_P35531659	239245483	239245542	chr1:239245483-239245542	0.14	0.70	-0.65
A_16_P15511059	239274053	239274112	chr1:239274053-239274112	1.00	0.39	-0.40
A_14_P134894	239313778	239313837	chr1:239313778-239313837	0.77	0.69	-0.49
A_16_P35531871	239337733	239337792	chr1:239337733-239337792	-0.11	0.85	-1.16
A_16_P15511253	239364269	239364328	chr1:239364269-239364328	0.94	0.69	-0.43
A_16_P00289944	239391358	239391405	chr1:239391358-239391405	0.58	0.55	-0.31
A_16_P15511366	239421919	239421973	chr1:239421919-239421973	0.32	0.41	-0.73
A_16_P00289959	239447109	239447168	chr1:239447109-239447168	1.03	1.34	-0.28
A_16_P00289969	239517083	239517142	chr1:239517083-239517142	0.45	1.03	-0.03
A_16_P00289979	239544570	239544614	chr1:239544570-239544614	0.73	0.70	0.22
A_16_P00289994	239630032	239630091	CEP170	0.12	-0.01	-0.94
A_14_P110070	239635092	239635150	CEP170	-0.13	-0.64	-0.73
A_16_P00290002	239643577	239643636	CEP170	-0.14	-0.24	-1.42
A_16_P00290015	239663268	239663327	CEP170	0.44	0.42	-1.32
A_16_P15512065	239701509	239701568	CEP170	0.15	-0.10	-0.09
A_16_P15512080	239708851	239708910	CEP170	0.14	0.68	-0.40
A_16_P15512107	239718643	239718702	BC050722	0.68	1.22	-0.26
A_16_P15512125	239725921	239725980	BC050722	0.59	1.07	-0.35
A_16_P00290081	239731436	239731495	BC050722	0.99	1.33	-0.30
A_16_P00290081	239731436	239731495	BC050722	0.97	1.22	-0.48
A_16_P00290081	239731436	239731495	BC050722	0.93	1.23	-0.44
A_14_P108031	239745243	239745287	chr1:239745243-239745287	0.02	1.25	0.31
A_16_P00290128	239753549	239753608	SDCCAG8	0.79	0.93	-0.43
A_16_P15512251	239765038	239765097	SDCCAG8	0.35	0.55	-0.35
A_14_P123374	239782416	239782475	SDCCAG8	0.46	1.41	-0.68
A_16_P15512288	239794094	239794153	SDCCAG8	0.60	0.85	-0.49
A_16_P00290191	239806186	239806245	SDCCAG8	0.00	0.77	-0.61
A_16_P00290199	239812643	239812700	SDCCAG8	0.82	1.21	-0.55
A_16_P35533067	239819825	239819884	SDCCAG8	0.05	2.15	-0.93
A_16_P00290222	239830356	239830415	SDCCAG8	0.44	0.74	-0.50
A_16_P00290238	239837025	239837084	SDCCAG8	0.52	0.73	-0.47
A_16_P15512423	239842982	239843041	SDCCAG8	0.90	1.68	-0.28
A_16_P15512448	239852965	239853024	SDCCAG8	0.27	0.95	-0.61
A_16_P15512473	239859778	239859837	SDCCAG8	0.50	1.39	-0.58
A_14_P135470	239868061	239868120	SDCCAG8	0.32	1.16	-0.58
A_16_P15512540	239881558	239881617	SDCCAG8	0.24	0.94	-0.43
A_16_P15512576	239892379	239892438	SDCCAG8	0.57	0.96	-0.41
A_16_P00290349	239897895	239897954	SDCCAG8	0.49	0.64	-0.40

A_14_P134161	239907379	239907438	SDCCAG8	0.03	0.17	-0.44
A_16_P15512661	239923550	239923609	SDCCAG8	0.61	0.05	-0.49
A_16_P15512698	239934992	239935051	SDCCAG8	1.15	0.75	-0.36
A_14_P117891	239942768	239942827	SDCCAG8	0.61	0.81	-0.30
A_16_P35533469	239951694	239951753	SDCCAG8	0.99	1.07	-0.47
A_16_P35533495	239960229	239960288	SDCCAG8	0.60	0.29	-0.41
A_16_P15512818	239971202	239971261	SDCCAG8	0.99	0.69	0.16
A_16_P00290510	239982793	239982852	SDCCAG8	0.87	0.74	-0.40
A_14_P108488	239989104	239989154	AKT3	0.51	1.58	-0.44
A_14_P107841	239992656	239992715	AKT3	0.18	-0.39	0.11
A_16_P15512931	240003647	240003706	AKT3	0.39	0.67	-0.49
A_16_P15512937	240010086	240010145	AKT3	0.57	0.93	-0.61
A_16_P15512956	240020775	240020834	AKT3	-0.04	1.08	-0.75
A_16_P15512963	240027011	240027070	AKT3	0.48	1.33	-0.56
A_16_P00290583	240034834	240034893	AKT3	0.14	0.45	-0.55
A_16_P00290594	240042100	240042159	AKT3	0.64	1.52	-0.52
A_16_P00290615	240051001	240051060	AKT3	0.32	-0.98	-0.58
A_14_P129004	240062287	240062342	AKT3	0.49	1.83	-0.39
A_16_P35533790	240068737	240068796	AKT3	0.24	1.38	-0.50
A_16_P15513075	240076432	240076491	AKT3	0.38	0.95	-0.59
A_16_P15513081	240086773	240086832	AKT3	0.22	-0.17	-0.77
A_16_P35533809	240092341	240092400	AKT3	0.28	0.63	-0.37
A_16_P00290674	240104419	240104478	AKT3	0.16	1.40	-0.76
A_16_P15513144	240112030	240112089	AKT3	0.48	0.49	-0.46
A_16_P35533883	240126952	240127011	AKT3	0.43	0.89	-0.44
A_14_P123877	240135234	240135293	AKT3	0.62	1.33	-0.20
A_16_P15513212	240154091	240154150	AKT3	0.26	1.47	-0.66
A_16_P35533965	240166567	240166626	AKT3	0.27	0.61	-0.63
A_16_P15513272	240175686	240175745	AKT3	0.19	0.65	-0.57
A_16_P00290772	240184962	240185021	AKT3	0.57	0.69	-0.33
A_16_P35534058	240197448	240197507	AKT3	0.52	0.62	-0.43
A_16_P15513371	240206140	240206199	AKT3	0.31	0.62	-0.50
A_16_P35534124	240216982	240217041	AKT3	0.46	1.23	-0.34
A_16_P00290853	240227455	240227514	AKT3	-0.17	1.52	-0.73
A_14_P135952	240239509	240239568	AKT3	-0.08	-0.18	-0.49
A_16_P35534205	240245080	240245139	AKT3	0.74	0.81	-0.40
A_16_P15513501	240253480	240253539	AKT3	-0.01	1.23	-0.64
A_16_P15513518	240259942	240260001	AKT3	-0.14	0.57	-0.32
A_16_P35534249	240265477	240265536	AKT3	0.43	0.89	-0.64
A_16_P15513555	240275972	240276031	AKT3	0.39	0.62	-0.60
A_16_P15513575	240288002	240288061	AKT3	0.18	1.23	-0.60
A_16_P15513598	240295570	240295629	AKT3	0.51	1.30	-0.36
A_16_P00290953	240303921	240303980	AKT3	0.68	-0.03	-0.61
A_16_P15513633	240320275	240320334	AKT3	0.32	0.87	-0.61
A_14_P108181	240332470	240332520	AKT3	0.93	0.93	-0.57
A_16_P15513698	240344557	240344616	chr1:240344557-240344616	0.53	0.93	-0.39
A_16_P15513739	240359839	240359898	chr1:240359839-240359898	-0.12	0.36	-0.38
A_16_P15513739	240359839	240359898	chr1:240359839-240359898	-0.22	0.93	-0.29

A_16_P15513739	240359839	240359898	chr1:240359839-240359898	-0.26	0.47	-0.46
A_14_P117696	240398127	240398186	chr1:240398127-240398186	0.90	1.23	-0.33
A_16_P35534625	240426687	240426746	BC046644	0.37	0.05	-0.57
A_16_P00291148	240444303	240444362	BC046644	0.49	0.53	-0.36
A_16_P00291187	240464148	240464207	BC046644	1.11	1.12	-0.28
A_16_P15514038	240473011	240473070	BC046644	0.40	0.70	-0.51
A_16_P15514073	240486172	240486231	BC046644	0.32	1.55	-0.51
A_16_P15514096	240500491	240500550	BC046644	0.72	1.34	-0.54
A_16_P15514116	240508950	240509009	BC046644	0.61	1.13	-0.45
A_14_P125181	240520548	240520607	BC046644	0.29	1.25	-0.48
A_16_P35534928	240532956	240533015	BC046644	0.47	0.70	-0.49
A_16_P35534944	240536754	240536813	chr1:240536754-240536813	0.51	1.00	-0.47
A_16_P00291309	240540752	240540811	ZNF238	0.88	1.55	-0.29
A_14_P127771	240546328	240546387	ZNF238	0.41	0.59	-0.37
A_16_P15514254	240551291	240551350	chr1:240551291-240551350	0.53	0.25	-0.41
A_16_P15514267	240555154	240555213	LOC440742	0.05	0.44	-0.16
A_16_P00291358	240562236	240562295	chr1:240562236-240562295	1.21	0.02	-0.30
A_16_P00291369	240569847	240569906	chr1:240569847-240569906	0.41	0.66	-0.54
A_16_P00291388	240582854	240582913	chr1:240582854-240582913	1.10	0.68	-0.38
A_16_P35535148	240598967	240599026	chr1:240598967-240599026	0.65	1.11	-0.54
A_16_P00291464	240619312	240619371	chr1:240619312-240619371	0.45	1.08	-0.44
A_16_P00291497	240637737	240637796	chr1:240637737-240637796	0.78	0.71	-0.22
A_16_P15514557	240647625	240647684	chr1:240647625-240647684	0.65	1.18	-0.28
A_16_P15514585	240657611	240657670	chr1:240657611-240657670	0.59	0.88	-0.41
A_16_P00291552	240667869	240667928	chr1:240667869-240667928	0.52	0.09	-0.38
A_16_P00291597	240691473	240691532	chr1:240691473-240691532	-0.01	0.72	-0.31
A_16_P00291621	240701103	240701162	chr1:240701103-240701162	0.32	-0.09	-0.29
A_16_P00291641	240711565	240711624	chr1:240711565-240711624	0.59	0.76	-0.31
A_16_P00291660	240721959	240722018	chr1:240721959-240722018	0.72	0.76	-0.37
A_16_P00291679	240733168	240733227	chr1:240733168-240733227	0.83	0.93	-0.59
A_16_P15514874	240760763	240760822	chr1:240760763-240760822	0.87	1.20	-0.31
A_16_P00291745	240778027	240778086	chr1:240778027-240778086	0.48	-0.65	-0.32
A_14_P108990	240790159	240790218	chr1:240790159-240790218	1.17	1.41	-0.32
A_16_P15514962	240802777	240802836	chr1:240802777-240802836	0.31	0.09	-0.36
A_16_P15514985	240812144	240812203	chr1:240812144-240812203	-0.18	1.16	0.17
A_16_P00291789	240828247	240828306	chr1:240828247-240828306	1.13	1.05	-0.24
A_16_P00291800	240835432	240835491	chr1:240835432-240835491	0.59	0.96	-0.34
A_16_P15515044	240842126	240842185	C1orf100	0.33	1.45	-0.47
A_16_P15515055	240848335	240848394	C1orf100	-0.09	0.89	-0.76
A_16_P15515066	240853856	240853915	C1orf100	0.47	0.85	-0.57
A_16_P35535863	240867763	240867822	C1orf100	0.71	1.05	-0.43
A_16_P35535882	240878139	240878198	C1orf100	0.65	1.22	-0.57
A_16_P15515130	240883803	240883862	chr1:240883803-240883862	1.26	1.32	-0.36
A_14_P130420	240898016	240898075	ADSS	0.49	0.75	-0.35
A_16_P15515174	240906916	240906975	ADSS	0.63	0.90	-0.41
A_16_P15515205	240915839	240915898	ADSS	0.41	0.76	-0.57
A_16_P15515224	240922679	240922738	ADSS	0.27	0.19	-0.44
A_14_P107574	240935701	240935760	ADSS	0.48	0.70	-0.32

A_16_P15515285	240941755	240941814	chr1:240941755-240941814	0.46	1.23	-0.44
A_16_P15515295	240952155	240952214	C1orf101	0.41	0.99	-0.38
A_16_P00291973	240967281	240967340	C1orf101	0.10	1.35	-0.44
A_16_P15515342	240979733	240979792	C1orf101	0.56	0.69	-0.52
A_16_P15515357	240988413	240988472	C1orf101	0.62	1.03	-0.18
A_14_P106543	241007976	241008035	C1orf101	0.09	1.49	-0.45
A_16_P35536159	241015904	241015954	C1orf101	0.98	0.55	-0.18
A_16_P00292002	241033120	241033179	C1orf101	0.48	1.09	-0.32
A_16_P15515392	241041687	241041746	C1orf101	0.73	1.66	-0.22
A_14_P120433	241050337	241050396	C1orf101	0.66	1.36	-0.11
A_16_P35536197	241061614	241061673	C1orf101	-0.51	1.00	-1.05
A_16_P35536216	241071466	241071525	C1orf101	0.76	0.42	-0.34
A_14_P114330	241082734	241082793	C1orf101	0.51	1.12	-0.39
A_16_P35536288	241095041	241095100	C1orf101	0.04	0.84	-0.39
A_16_P00292088	241107678	241107737	C1orf101	0.50	0.77	-0.19
A_16_P35536336	241122799	241122858	C1orf101	0.41	1.06	-0.42
A_14_P128538	241129384	241129443	C1orf101	0.09	0.61	-0.55
A_16_P15515571	241136189	241136248	chr1:241136189-241136248	0.35	0.49	-0.56
A_14_P101445	241145731	241145790	C1orf121	0.63	0.85	-0.29
A_16_P15515619	241157831	241157890	C1orf121	0.61	1.24	-0.30
A_16_P15515637	241166914	241166973	C1orf121	0.37	0.48	-0.35
A_16_P15515672	241181404	241181463	C1orf121	0.60	1.27	-0.32
A_14_P114520	241195262	241195321	C1orf121	0.05	1.86	-0.08
A_16_P15515713	241203852	241203911	chr1:241203852-241203911	1.03	1.46	-0.31
A_16_P00292205	241215451	241215510	chr1:241215451-241215510	0.81	0.41	-0.20
A_16_P00292220	241227367	241227426	chr1:241227367-241227426	0.64	0.58	-0.44
A_14_P109660	241245331	241245390	chr1:241245331-241245390	0.78	0.39	0.15
A_16_P00292243	241261111	241261170	chr1:241261111-241261170	1.60	1.27	-0.21
A_16_P15515817	241275947	241276006	chr1:241275947-241276006	0.50	1.07	-0.28
A_16_P35536648	241295224	241295283	chr1:241295224-241295283	0.67	1.41	-0.30
A_16_P35536657	241312002	241312061	chr1:241312002-241312061	0.55	0.98	-0.37
A_16_P00292286	241319725	241319784	chr1:241319725-241319784	0.94	0.38	-0.24
A_14_P114352	241326387	241326446	FAM36A	0.50	1.13	-0.31
A_16_P35536709	241331609	241331668	FAM36A	0.56	0.39	-0.34
A_16_P00292317	241335900	241335959	AK095297	0.35	0.64	-0.25
A_16_P15515946	241341813	241341872	HNRPU	0.50	1.26	-0.31
A_14_P110500	241345935	241345994	HNRPU	0.64	1.30	0.16
A_14_P127963	241349660	241349719	HNRPU	0.84	1.76	-0.22
A_16_P00292358	241354309	241354368	chr1:241354309-241354368	0.97	1.34	-0.22
A_16_P15516003	241360093	241360152	chr1:241360093-241360152	0.42	0.93	-0.53
A_16_P00292373	241365981	241366040	chr1:241365981-241366040	0.36	0.79	-0.29
A_16_P15516035	241378638	241378697	chr1:241378638-241378697	0.77	0.16	-0.50
A_16_P00292400	241389544	241389603	chr1:241389544-241389603	-0.24	0.73	-0.14
A_16_P15516067	241398867	241398926	chr1:241398867-241398926	0.20	0.95	-0.42
A_16_P15516104	241412731	241412790	chr1:241412731-241412790	0.50	0.79	-0.55
A_16_P00292440	241422008	241422067	chr1:241422008-241422067	0.59	0.00	-0.41
A_16_P15516142	241431979	241432038	chr1:241431979-241432038	0.00	-0.19	-0.61
A_16_P15516157	241440175	241440234	chr1:241440175-241440234	0.61	0.72	-0.37

A_14_P120784	241458138	241458197	AK126649	0.65	1.26	-0.30
A_16_P35537060	241463783	241463842	EFCAB2	0.80	1.33	-0.26
A_16_P35537085	241477942	241478001	EFCAB2	0.47	0.82	-0.42
A_14_P120309	241488339	241488398	EFCAB2	0.60	0.89	-0.62
A_14_P105495	241506606	241506665	EFCAB2	0.57	1.25	-0.32
A_16_P15516347	241523410	241523469	EFCAB2	0.83	1.91	-0.34
A_16_P35537185	241532862	241532921	EFCAB2	0.30	0.81	-0.38
A_16_P35537198	241540161	241540220	EFCAB2	0.85	0.30	-0.21
A_14_P106253	241548709	241548768	EFCAB2	0.24	1.30	-0.47
A_16_P35537237	241556808	241556867	EFCAB2	0.76	0.70	-0.38
A_16_P35537259	241566402	241566461	EFCAB2	0.68	0.90	-0.26
A_14_P103341	241577002	241577061	EFCAB2	0.30	1.15	-0.50
A_16_P00292636	241585549	241585608	AK090927	1.05	1.09	-0.20
A_16_P15516499	241595526	241595585	AK090927	0.57	0.58	-0.48
A_16_P15516517	241602444	241602503	AK090927	0.34	0.44	-0.44
A_16_P15516533	241608581	241608640	AK090927	0.73	1.11	-0.34
A_16_P00292678	241616114	241616173	AK027158	0.73	1.04	-0.28
A_16_P15516577	241628759	241628818	chr1:241628759-241628818	0.81	0.72	-0.36
A_16_P00292701	241635579	241635638	chr1:241635579-241635638	0.39	0.38	-0.35
A_16_P00292714	241647019	241647078	AK125187	0.92	1.40	-0.21
A_16_P00292760	241670441	241670500	AK125187	0.85	0.80	-0.37
A_14_P138460	241689448	241689507	AK125187	1.18	2.12	-0.39
A_16_P15516803	241710474	241710533	AK125187	0.62	0.58	-0.21
A_16_P35537678	241722579	241722638	AK125187	0.79	1.22	-0.26
A_16_P00292874	241736036	241736095	AK125187	0.73	0.52	-0.30
A_16_P15516907	241753108	241753167	AK125187	0.83	0.53	-0.48
A_16_P00292909	241767261	241767320	AK125187	0.83	1.45	-0.20
A_16_P00292931	241783821	241783880	AK125187	0.41	1.52	-0.29
A_16_P15517010	241802158	241802217	AK125187	0.53	1.17	-0.46
A_16_P15517037	241810847	241810906	AK125187	0.11	0.74	-0.60
A_16_P15517112	241832902	241832961	AK125187	0.57	1.28	-0.11
A_16_P15517121	241838953	241839012	AK125187	0.51	1.07	-0.32
A_14_P201708	241848046	241848105	AK125187	0.72	0.92	-0.74
A_14_P113203	241854082	241854141	AK125187	0.13	0.81	-0.16
A_16_P00293092	241870174	241870233	AK125187	0.79	0.90	-0.20
A_16_P35538091	241878929	241878988	AK125187	0.44	1.26	-0.53
A_16_P15517248	241886162	241886221	AK125187	0.65	1.72	-0.43
A_14_P107961	241902597	241902656	AK125187	1.00	1.43	-0.22
A_16_P15517337	241914600	241914659	AK125187	0.62	1.56	-0.22
A_16_P35538230	241928835	241928894	AK125187	0.56	0.90	-0.23
A_16_P15517416	241943066	241943125	AK125187	0.55	0.90	-0.49
A_16_P15517472	241960379	241960438	AK125187	0.13	1.06	-0.88
A_16_P35538355	241973425	241973484	AK125187	0.67	1.03	-0.42
A_16_P00293286	241983282	241983341	AK125187	1.02	0.82	-0.41
A_16_P00293299	241991503	241991562	AK125187	0.81	1.38	-0.39
A_16_P00293319	242000411	242000470	AK125187	0.74	0.93	-0.41
A_16_P00293336	242008027	242008086	AK125187	0.34	-0.06	-0.55
A_16_P15517619	242014554	242014613	AK125187	0.55	1.08	-0.56

A_16_P35538500	242023004	242023063	AK125187	0.23	0.99	-0.30
A_16_P00293384	242032251	242032310	AK125187	0.26	1.25	-0.31
A_16_P00293408	242044016	242044075	AK125187	-0.20	1.08	-0.86
A_14_P133474	242050797	242050856	AK125187	0.65	0.76	-0.28
A_16_P00293449	242066190	242066249	AK125187	0.26	0.45	-0.35
A_16_P35538657	242073765	242073824	AK125187	1.11	1.55	-0.35
A_16_P00293481	242081852	242081911	AK125187	0.91	0.73	-0.14
A_16_P15517851	242093936	242093995	AK125187	0.67	0.73	-0.19
A_16_P00293533	242112003	242112047	AK125187	0.91	1.32	0.15
A_16_P00293534	242131661	242131712	AK125187	0.30	0.82	-0.12
A_16_P00293554	242142346	242142405	AK125187	0.69	0.81	-0.46
A_16_P00293564	242148615	242148674	AK125187	0.08	0.81	-0.38
A_16_P35538837	242154208	242154267	AK125187	0.13	1.29	-0.70
A_16_P35538864	242165719	242165778	AK125187	0.68	1.06	-0.40
A_16_P00293597	242172995	242173053	AK125187	0.98	1.44	-0.23
A_16_P15518043	242180944	242181003	AK125187	0.45	1.35	-0.20
A_16_P00293632	242188939	242188998	AK125187	0.45	1.47	-0.19
A_14_P109963	242196701	242196760	AK095500	0.32	0.90	-0.33
A_16_P35539013	242209630	242209689	chr1:242209630-242209689	0.29	0.49	-0.55
A_16_P15518142	242228666	242228725	chr1:242228666-242228725	1.00	1.87	-0.24
A_14_P108127	242238722	242238780	SMYD3	0.57	0.91	-0.28
A_16_P15518177	242244330	242244386	SMYD3	0.27	0.45	0.32
A_16_P15518187	242251663	242251722	SMYD3	1.28	1.81	-0.37
A_16_P15518226	242262506	242262565	SMYD3	1.07	1.59	-0.21
A_16_P15518249	242274664	242274723	SMYD3	0.78	1.05	-0.33
A_16_P00293752	242282253	242282312	SMYD3	0.90	1.19	-0.28
A_16_P15518310	242292432	242292491	SMYD3	0.44	0.95	-0.06
A_16_P15518328	242298484	242298543	SMYD3	0.28	1.83	-0.15
A_16_P15518353	242312117	242312176	SMYD3	0.26	0.77	-0.45
A_16_P15518369	242317764	242317823	SMYD3	0.57	1.08	-0.34
A_16_P15518385	242324289	242324348	SMYD3	-4.28	1.38	-0.36
A_16_P00293837	242331550	242331609	SMYD3	0.44	0.74	-0.50
A_16_P15518445	242344429	242344488	SMYD3	0.97	1.24	-0.36
A_14_P121648	242353152	242353210	SMYD3	1.12	1.21	-0.23
A_16_P00293908	242368340	242368399	SMYD3	0.83	0.91	-0.15
A_16_P15518545	242377309	242377368	SMYD3	0.36	0.86	-0.34
A_16_P35539455	242390044	242390103	SMYD3	0.78	0.24	-0.28
A_16_P00293944	242407249	242407308	SMYD3	0.63	0.74	-0.34
A_16_P00293970	242419536	242419595	SMYD3	0.67	1.02	-0.29
A_16_P00293982	242427030	242427089	SMYD3	0.59	-0.18	0.14
A_16_P35539544	242436266	242436325	SMYD3	0.70	1.17	-0.43
A_14_P109363	242442565	242442624	SMYD3	0.33	1.01	-0.50
A_16_P00294013	242450648	242450707	SMYD3	0.60	1.19	-0.38
A_16_P15518742	242469203	242469262	SMYD3	0.52	-0.25	-0.34
A_16_P35539668	242483333	242483392	SMYD3	1.31	2.34	-0.25
A_16_P00294079	242491385	242491444	SMYD3	0.93	1.42	-0.28
A_16_P35539726	242500010	242500069	SMYD3	0.40	0.19	-0.29
A_16_P00294111	242508806	242508865	SMYD3	0.75	1.24	-0.25

A_16_P15518888	242520588	242520647	SMYD3	0.52	1.22	-0.41
A_14_P130594	242527482	242527541	SMYD3	0.37	0.99	-0.07
A_16_P35539821	242533627	242533686	SMYD3	0.66	0.64	-0.28
A_16_P15518933	242539187	242539246	SMYD3	0.71	0.71	-0.28
A_16_P15518947	242545876	242545935	SMYD3	0.63	1.04	-0.47
A_16_P15518963	242552245	242552304	SMYD3	0.39	0.14	-0.39
A_16_P15518994	242560808	242560867	SMYD3	-0.15	0.11	-0.46
A_16_P35539909	242569089	242569148	SMYD3	0.71	1.10	-0.35
A_16_P15519028	242576951	242577010	SMYD3	0.64	0.91	-0.24
A_16_P15519060	242587054	242587113	SMYD3	0.86	0.88	-0.43
A_16_P35540000	242600295	242600354	SMYD3	0.57	1.10	-0.37
A_16_P15519120	242606468	242606527	SMYD3	0.36	1.30	-0.23
A_16_P15519159	242620705	242620764	SMYD3	0.56	0.84	-0.27
A_16_P00294304	242627580	242627639	SMYD3	0.66	0.53	-0.20
A_16_P00294320	242635335	242635394	SMYD3	0.70	1.16	-0.17
A_16_P15519224	242641859	242641918	SMYD3	0.73	1.57	-0.23
A_16_P15519243	242647488	242647547	SMYD3	0.80	1.02	-0.30
A_16_P00294364	242655988	242656047	SMYD3	0.24	1.35	-0.18
A_16_P15519297	242667000	242667059	SMYD3	0.73	0.52	-0.13
A_14_P104128	242676719	242676778	SMYD3	0.58	0.41	-0.55
A_16_P00294415	242684172	242684231	SMYD3	0.30	1.10	-0.35
A_16_P15519371	242691646	242691705	SMYD3	0.19	0.49	-0.48
A_16_P35540292	242698399	242698458	SMYD3	0.88	1.26	-0.11
A_16_P00294458	242704805	242704864	SMYD3	0.54	1.02	-0.42
A_16_P15519427	242711311	242711370	SMYD3	0.71	1.62	-0.36
A_16_P15519447	242718193	242718252	SMYD3	0.00	0.52	-0.64
A_16_P15519473	242725418	242725477	SMYD3	0.34	0.08	-0.49
A_16_P15519485	242733118	242733177	SMYD3	0.39	1.40	0.08
A_16_P15519504	242739091	242739150	SMYD3	0.66	0.90	0.04
A_16_P15519533	242754982	242755041	SMYD3	0.57	1.28	0.10
A_16_P15519550	242763930	242763989	SMYD3	0.50	0.35	-0.03
A_16_P35540480	242776752	242776811	SMYD3	0.27	1.57	-0.01
A_16_P15519606	242784906	242784965	SMYD3	0.60	1.15	-0.36
A_16_P15519625	242790473	242790532	SMYD3	0.53	1.39	-0.29
A_16_P35540585	242808787	242808846	SMYD3	0.77	0.77	-0.29
A_16_P35540619	242819787	242819846	SMYD3	-0.07	0.40	-0.53
A_16_P15519732	242827674	242827733	SMYD3	0.70	1.43	-0.41
A_14_P101454	242844344	242844403	SMYD3	-0.15	1.63	-0.10
A_16_P15519788	242859245	242859304	SMYD3	0.79	1.51	-0.30
A_16_P00294694	242869381	242869440	SMYD3	0.61	0.38	-0.13
A_16_P15519828	242876242	242876301	SMYD3	0.37	1.27	-0.29
A_16_P35540769	242889046	242889105	SMYD3	0.52	0.70	-0.48
A_16_P15519897	242905215	242905274	SMYD3	0.39	0.70	-0.43
A_16_P00294746	242916155	242916214	AB057595	0.21	0.34	-0.50
A_16_P15519953	242932738	242932797	AB057595	0.88	1.09	-0.15
A_16_P00294779	242939288	242939347	AB057595	0.68	0.71	-0.11
A_16_P35540900	242946290	242946349	AB057595	1.10	0.08	-0.06
A_14_P132848	242952336	242952395	AB057595	0.99	1.16	-0.31

A_16_P15520035	242966328	242966387	AB057595	0.43	0.17	-0.49
A_16_P15520078	242981398	242981457	AB057595	0.49	1.21	-0.40
A_16_P15520103	242991896	242991955	AB057595	0.82	-1.15	-0.20
A_16_P00294870	243004283	243004342	chr1:243004283-243004342	0.89	0.69	0.02
A_16_P00294886	243016713	243016768	chr1:243016713-243016768	0.97	0.59	-0.09
A_14_P135018	243030504	243030563	TFB2M	0.76	1.26	-0.35
A_16_P35541139	243039115	243039174	TFB2M	0.66	0.49	-0.27
A_14_P114977	243046700	243046758	TFB2M	0.57	1.09	0.05
A_14_P127503	243057553	243057612	C1orf71	0.15	1.21	-0.20
A_16_P15520296	243071557	243071615	C1orf71	0.32	0.76	-0.49
A_16_P00294968	243080845	243080904	C1orf71	0.53	1.45	-0.30
A_14_P135786	243094569	243094628	C1orf71	0.79	1.45	-0.04
A_16_P15520367	243104163	243104222	C1orf71	0.37	0.47	-0.24
A_16_P35541323	243110693	243110752	C1orf71	0.92	1.27	-0.21
A_16_P00295013	243118229	243118286	C1orf71	0.89	1.11	-0.34
A_16_P15520417	243123841	243123900	C1orf71	0.65	1.49	-0.41
A_14_P116888	243131399	243131458	C1orf71	0.47	0.24	-0.09
A_16_P15520448	243137202	243137261	C1orf71	0.74	0.80	-0.31
A_16_P35541435	243151259	243151318	C1orf71	0.90	0.94	-0.29
A_14_P128563	243157053	243157112	AL834246	0.47	0.89	-0.21
A_16_P15520527	243166941	243167000	chr1:243166941-243167000	0.57	1.21	-0.44
A_16_P35541487	243174525	243174584	chr1:243174525-243174584	0.98	1.51	-0.21
A_16_P15520571	243182856	243182915	chr1:243182856-243182915	1.41	0.52	-0.16
A_16_P15520589	243189422	243189481	chr1:243189422-243189481	1.13	1.65	-0.20
A_14_P111342	243196544	243196603	chr1:243196544-243196603	0.75	0.85	-0.28
A_16_P15520611	243203030	243203084	chr1:243203030-243203084	0.50	0.21	-0.24
A_14_P129642	243216209	243216268	SCCPDH	0.63	1.20	-0.60
A_16_P00295144	243225309	243225368	SCCPDH	0.57	0.70	-0.13
A_16_P35541601	243234289	243234348	SCCPDH	0.46	0.58	-0.32
A_16_P15520683	243249254	243249313	SCCPDH	0.31	1.02	-0.35
A_14_P103238	243257092	243257151	SCCPDH	0.95	1.15	-0.39
A_16_P15520726	243266882	243266941	AK022825	0.62	-0.90	-0.42
A_16_P15520749	243273661	243273720	AK022825	0.83	0.94	-0.18
A_16_P00295221	243280107	243280166	LOC149134	0.74	1.00	-0.38
A_16_P15520785	243284988	243285045	chr1:243284988-243285045	0.89	1.08	-0.20
A_16_P00295237	243288977	243289036	chr1:243288977-243289036	0.85	0.83	0.03
A_16_P15520813	243297213	243297272	chr1:243297213-243297272	0.72	1.20	-0.24
A_16_P15520830	243304623	243304682	chr1:243304623-243304682	0.42	0.64	-0.64
A_14_P104647	243310512	243310571	chr1:243310512-243310571	0.89	0.89	-0.34
A_16_P00295265	243318708	243318767	chr1:243318708-243318767	0.58	0.95	-0.22
A_16_P15520856	243327540	243327599	chr1:243327540-243327599	1.01	1.65	-0.35
A_14_P113808	243335625	243335684	AHCTF1	0.79	1.09	-0.29
A_16_P15520900	243341162	243341221	AHCTF1	0.74	0.89	-0.25
A_16_P15520953	243356305	243356364	AHCTF1	0.58	0.81	-0.33
A_14_P136431	243366473	243366532	AHCTF1	0.60	0.94	-0.31
A_16_P15520996	243373589	243373648	AHCTF1	0.53	0.88	-0.29
A_16_P35542002	243381572	243381631	AHCTF1	0.39	0.60	-0.35
A_16_P35542048	243393369	243393428	AHCTF1	0.47	0.73	-0.31

A_16_P35542067	243400442	243400501	AHCTF1	1.03	1.08	-0.01
A_16_P15521109	243410990	243411049	AY157619	0.54	1.49	-0.33
A_16_P35542125	243420173	243420232	AY157619	0.57	0.72	-0.39
A_16_P35542151	243427799	243427858	chr1:243427799-243427858	0.46	0.67	-0.31
A_16_P00295436	243434938	243434997	ZNF695	0.40	0.63	-0.35
A_16_P15521207	243445048	243445107	ZNF695	0.39	0.59	-0.34
A_16_P00295458	243450514	243450573	ZNF695	0.21	0.62	-0.50
A_14_P103460	243462852	243462911	ZNF695	0.17	1.56	-0.19
A_14_P111042	243477704	243477763	ZNF695	0.43	0.67	-0.31
A_16_P35542272	243487878	243487937	ZNF695	1.03	1.72	-0.32
A_14_P117067	243495592	243495651	ZNF695	0.75	0.69	-0.16
A_16_P15521301	243506710	243506769	CR597038	0.59	0.89	-0.37
A_16_P15521315	243513944	243514003	CR597038	0.84	1.23	-0.18
A_16_P15521317	243519593	243519652	CR597038	-0.09	0.89	-0.40
A_14_P200053	243526552	243526611	ZNF670	0.47	0.30	-0.45
A_14_P132103	243529049	243529108	ZNF670	0.94	0.60	-0.57
A_16_P15521354	243543014	243543066	ZNF670	0.47	1.09	-0.19
A_14_P130459	243552376	243552435	ZNF670	0.95	1.36	-0.13
A_16_P15521373	243560984	243561043	ZNF670	1.25	0.95	-0.03
A_16_P15521390	243569213	243569271	chr1:243569213-243569271	0.72	1.03	-0.31
A_16_P35542400	243576511	243576570	chr1:243576511-243576570	1.02	0.40	-0.19
A_16_P00295574	243581775	243581834	chr1:243581775-243581834	0.28	1.03	-0.49
A_16_P15521419	243585876	243585935	chr1:243585876-243585935	1.06	2.27	0.25
A_14_P135266	243590741	243590800	ZNF669	-0.06	0.85	-0.52
A_16_P35542436	243592299	243592358	ZNF669	0.38	0.85	-0.35
A_16_P00295590	243603285	243603337	chr1:243603285-243603337	0.47	1.53	0.14
A_16_P00295601	243612095	243612154	BC032514	1.42	1.01	-0.14
A_16_P15521490	243619716	243619775	BC032514	0.38	1.14	-0.25
A_16_P00295610	243633055	243633114	BC032514	0.78	0.88	-0.49
A_16_P00295614	243637678	243637737	BC032514	0.43	0.15	-0.42
A_16_P15521515	243642129	243642188	BC032514	0.34	1.08	-0.47
A_14_P136021	243647013	243647072	ZNF124	0.49	1.27	-0.18
A_16_P15521541	243656701	243656760	ZNF124	0.70	1.45	-0.28
A_16_P15521561	243665675	243665734	chr1:243665675-243665734	-0.20	1.01	-0.66
A_14_P108387	243674828	243674887	chr1:243674828-243674887	0.32	1.20	0.12
A_16_P15521612	243683074	243683133	chr1:243683074-243683133	-0.01	1.09	-0.44
A_16_P15521625	243691053	243691112	chr1:243691053-243691112	0.66	0.46	-0.34
A_16_P00295693	243699103	243699162	chr1:243699103-243699162	0.54	1.10	-0.38
A_16_P15521652	243706605	243706664	chr1:243706605-243706664	0.70	1.33	-0.20
A_16_P15521654	243711629	243711678	chr1:243711629-243711678	0.43	1.43	-0.03
A_16_P15521660	243716503	243716562	chr1:243716503-243716562	0.10	1.25	-0.39
A_16_P35542677	243721086	243721132	chr1:243721086-243721132	0.70	1.23	0.15
A_16_P15521681	243727635	243727694	chr1:243727635-243727694	1.10	1.73	-0.31
A_16_P15521699	243735042	243735101	chr1:243735042-243735101	0.86	1.29	-0.28
A_16_P00295724	243742440	243742499	chr1:243742440-243742499	0.36	1.18	-0.39
A_14_P127091	243745726	243745785	VN1R5	0.80	1.01	-0.34
A_16_P35542728	243751073	243751132	chr1:243751073-243751132	-0.14	0.14	-0.40
A_16_P00295734	243764309	243764368	chr1:243764309-243764368	1.01	1.34	-0.12

A_16_P15521739	243775390	243775449	chr1:243775390-243775449	0.78	0.49	-0.30
A_16_P00295745	243785097	243785156	chr1:243785097-243785156	0.75	1.48	-0.20
A_16_P15521771	243792226	243792280	ZNF496	0.74	1.73	-0.16
A_14_P110030	243799752	243799806	ZNF496	1.15	0.95	-0.12
A_16_P00295787	243815666	243815725	ZNF496	0.40	0.32	-0.29
A_16_P00295805	243826627	243826686	chr1:243826627-243826686	0.92	1.84	-0.20
A_16_P15521882	243837796	243837855	chr1:243837796-243837855	1.31	1.49	-0.03
A_16_P00295819	243850430	243850489	chr1:243850430-243850489	0.80	0.77	-0.34
A_16_P15521903	243868941	243869000	chr1:243868941-243869000	0.30	0.63	-0.50
A_16_P15521910	243878604	243878663	chr1:243878604-243878663	1.09	1.55	0.32
A_16_P15521930	243887092	243887151	chr1:243887092-243887151	-0.19	-0.10	-0.48
A_16_P35542954	243894126	243894185	chr1:243894126-243894185	0.61	1.16	-0.31
A_14_P105324	243907505	243907564	CIAS1	0.66	-0.33	-0.54
A_16_P15522006	243919983	243920042	CIAS1	0.71	0.41	-0.41
A_14_P127104	243931046	243931105	CIAS1	0.79	1.29	0.13
A_16_P15522058	243940317	243940376	chr1:243940317-243940376	1.15	1.01	-0.29
A_16_P00295920	243944763	243944822	chr1:243944763-243944822	1.03	0.69	-0.17
A_16_P15522093	243950715	243950774	chr1:243950715-243950774	-0.32	1.48	-0.45
A_16_P00295938	243956660	243956719	chr1:243956660-243956719	0.18	1.80	-0.60
A_16_P15522125	243964226	243964285	chr1:243964226-243964285	0.34	0.69	-0.42
A_16_P15522135	243970327	243970386	chr1:243970327-243970386	1.00	1.22	-0.27
A_16_P00295963	243976211	243976270	chr1:243976211-243976270	0.63	0.87	-0.42
A_16_P00295978	243982993	243983052	chr1:243982993-243983052	0.94	1.19	0.13
A_16_P00295992	243996230	243996289	chr1:243996230-243996289	0.42	1.43	-0.11
A_16_P15522208	244005772	244005831	AK124520	0.46	0.64	-0.44
A_16_P15522222	244009968	244010027	AK124520	0.16	1.00	-0.30
A_16_P15522243	244017407	244017466	AK124520	0.36	0.74	-0.68
A_14_P118208	244021734	244021792	OR2C3	1.09	1.24	-0.31
A_16_P00296028	244030126	244030185	AK124520	0.16	1.12	-0.29
A_16_P00296037	244033781	244033840	AK124520	-0.19	0.62	-0.27
A_14_P106879	244038512	244038563	C1orf150	0.75	1.72	-0.29
A_16_P15522308	244044589	244044648	C1orf150	0.45	1.59	-0.53
A_16_P00296067	244052931	244052990	C1orf150	0.33	0.00	-0.69
A_14_P112778	244063856	244063915	C1orf150	0.81	1.51	-0.28
A_16_P35543378	244069950	244070009	chr1:244069950-244070009	0.44	1.65	-0.21
A_16_P15522354	244073609	244073668	chr1:244073609-244073668	0.26	0.94	-0.75
A_16_P35543387	244077743	244077802	OR2G2	0.47	1.49	-0.28
A_16_P35543401	244081303	244081362	chr1:244081303-244081362	0.23	0.83	-0.36
A_16_P15522382	244088506	244088565	chr1:244088506-244088565	0.57	1.10	-0.31
A_16_P00296100	244092520	244092579	chr1:244092520-244092579	0.23	0.66	-0.48
A_16_P35543428	244095837	244095896	chr1:244095837-244095896	0.19	0.41	-0.53
A_16_P15522407	244102451	244102510	chr1:244102451-244102510	0.44	0.90	-0.52
A_16_P15522429	244111998	244112057	chr1:244111998-244112057	0.62	0.45	-0.52
A_16_P35543469	244119151	244119210	chr1:244119151-244119210	0.06	0.78	-0.68
A_16_P00296132	244127104	244127163	chr1:244127104-244127163	0.74	1.35	-0.40
A_16_P35543506	244137193	244137252	BC036260	0.92	1.42	-0.37
A_16_P15522492	244144446	244144505	BC036260	0.49	1.29	-0.46
A_16_P15522507	244157059	244157118	BC036260	0.63	-0.12	-0.52

A_16_P00296179	244160926	244160985	BC036260	0.32	-0.09	-0.53
A_16_P15522558	244172274	244172333	BC036260	-0.21	1.49	-0.55
A_16_P15522563	244182577	244182636	BC036260	-0.26	-0.01	-1.13
A_16_P15522572	244186577	244186636	BC036260	1.18	1.26	-0.38
A_16_P15522583	244193955	244194014	BC036260	0.37	0.79	-0.40
A_16_P35543621	244201104	244201160	BC036260	0.76	1.22	-0.12
A_14_P137657	244205967	244206026	BC036260	0.11	0.33	-0.66
A_16_P15522618	244212054	244212113	BC036260	0.26	0.30	-0.52
A_16_P35543665	244218615	244218674	BC036260	0.08	0.49	-0.65
A_16_P15522646	244227927	244227986	BC036260	0.80	1.26	-0.46
A_16_P15522659	244231455	244231514	BC036260	0.44	1.29	-0.13
A_16_P35543717	244236273	244236332	BC036260	0.14	0.65	-0.68
A_16_P00296272	244246745	244246804	chr1:244246745-244246804	0.87	1.34	-0.46
A_16_P15522708	244253275	244253334	chr1:244253275-244253334	0.26	0.88	-0.76
A_16_P15522718	244259016	244259075	chr1:244259016-244259075	0.08	1.32	-0.62
A_16_P00296304	244264241	244264300	AY358215	0.70	0.54	-0.49
A_16_P15522750	244269339	244269398	AY358215	0.09	0.83	-0.51
A_16_P00296315	244285252	244285311	chr1:244285252-244285311	0.42	1.13	-0.44
A_16_P15522773	244292656	244292715	chr1:244292656-244292715	0.25	0.79	-0.42
A_16_P35543822	244296502	244296561	chr1:244296502-244296561	0.35	0.62	-0.42
A_16_P15522789	244301582	244301641	chr1:244301582-244301641	0.21	-0.16	-0.44
A_16_P15522809	244307178	244307237	chr1:244307178-244307237	-0.29	0.79	-0.56
A_16_P15522816	244312348	244312407	chr1:244312348-244312407	0.70	0.76	-0.34
A_16_P35543872	244316732	244316791	chr1:244316732-244316791	0.71	0.93	-0.54
A_16_P15522846	244325069	244325128	chr1:244325069-244325128	0.23	0.78	-0.54
A_14_P115958	244330009	244330068	chr1:244330009-244330068	-0.13	0.42	-0.54
A_16_P35543916	244336019	244336078	chr1:244336019-244336078	0.33	0.82	-0.32
A_16_P15522886	244341184	244341243	chr1:244341184-244341243	0.68	0.64	-0.77
A_16_P00296397	244344544	244344603	chr1:244344544-244344603	0.82	1.07	-0.34
A_14_P120939	244347924	244347983	TRIM58	0.10	1.48	-0.56
A_16_P15522924	244353155	244353213	TRIM58	0.95	1.04	-0.27
A_14_P106678	244357171	244357217	TRIM58	0.64	1.00	0.20
A_16_P15522949	244362720	244362777	TRIM58	0.86	0.75	-0.29
A_14_P105489	244366498	244366557	TRIM58	-0.12	1.34	-0.63
A_16_P00296444	244373479	244373538	BX537432	0.60	1.25	-0.46
A_16_P15522983	244377265	244377324	BX537432	0.51	0.66	-0.43
A_14_P121873	244386401	244386460	BX537432	0.28	1.12	0.01
A_16_P00296462	244397508	244397567	chr1:244397508-244397567	0.25	1.43	-0.60
A_16_P15523005	244401981	244402040	chr1:244401981-244402040	0.45	0.72	-0.51
A_16_P35544061	244405939	244405998	chr1:244405939-244405998	0.49	0.85	-0.59
A_16_P15523027	244409714	244409773	chr1:244409714-244409773	0.74	1.18	-0.50
A_16_P35544095	244415685	244415744	chr1:244415685-244415744	0.66	1.03	-0.46
A_16_P15523057	244419870	244419929	chr1:244419870-244419929	0.25	0.68	-0.75
A_16_P35544120	244424319	244424378	chr1:244424319-244424378	0.48	1.17	-0.57
A_16_P35544136	244428449	244428508	OR2L13	0.25	1.17	-0.48
A_16_P00296519	244437136	244437195	OR2L13	0.50	1.13	-0.51
A_16_P00296525	244442266	244442325	OR2L13	0.13	1.41	-0.17
A_16_P00296529	244450748	244450807	OR2L13	1.05	0.76	-0.36

A_14_P115257	244457394	244457453	OR2L13	0.01	1.34	-0.68
A_16_P15523152	244460836	244460895	OR2L13	0.71	1.22	-0.48
A_16_P15523165	244467764	244467823	OR2L13	0.38	1.59	-0.38
A_16_P15523186	244473682	244473741	OR2L13	0.24	0.45	-1.00
A_16_P15523209	244484980	244485039	OR2L13	0.55	0.64	-0.59
A_16_P35544280	244490772	244490831	OR2L13	0.07	0.53	-0.91
A_16_P00296596	244496652	244496711	OR2L13	0.39	-0.24	-0.57
A_14_P124575	244505096	244505155	OR2L13	-0.28	1.67	-1.00
A_16_P35544334	244515511	244515570	OR2L13	0.63	1.40	-0.54
A_16_P35544356	244526367	244526426	OR2L13	0.30	1.04	-0.32
A_14_P119862	244529304	244529363	OR2L13	0.29	1.46	-0.56
A_16_P15523330	244536115	244536174	OR2L13	-0.19	0.35	-0.48
A_16_P00296647	244539577	244539636	OR2L13	0.23	0.62	-0.71
A_16_P00296649	244547636	244547695	OR2L13	0.46	0.61	-0.69
A_14_P131046	244554065	244554124	OR2L13	0.38	0.23	-0.81
A_16_P15523370	244563262	244563321	OR2L13	0.23	0.14	-0.46
A_16_P15523371	244568620	244568679	OR2L13	0.27	0.88	-0.37
A_16_P35544442	244574215	244574274	OR2L13	0.37	-1.39	-0.70
A_16_P00296679	244582846	244582905	OR2L13	0.88	1.15	-0.39
A_14_P116408	244588695	244588754	OR2L13	0.43	1.33	-0.47
A_14_P136390	244604052	244604111	chr1:244604052-244604111	-0.40	0.48	-0.81
A_16_P35544516	244612276	244612335	chr1:244612276-244612335	0.50	1.68	-0.49
A_16_P15523458	244617610	244617669	chr1:244617610-244617669	0.12	1.01	-0.55
A_16_P00296712	244627520	244627579	chr1:244627520-244627579	0.30	1.09	-0.64
A_16_P15523492	244634239	244634296	chr1:244634239-244634296	0.40	0.97	-0.26
A_16_P35544557	244642962	244643021	chr1:244642962-244643021	0.13	1.74	-0.38
A_16_P15523512	244650259	244650318	chr1:244650259-244650318	0.32	0.66	-0.68
A_16_P15523526	244654854	244654899	chr1:244654854-244654899	0.66	0.66	0.09
A_16_P35544587	244662048	244662107	chr1:244662048-244662107	0.09	1.19	-0.55
A_16_P15523546	244665981	244666040	chr1:244665981-244666040	0.21	0.48	-0.40
A_16_P00296760	244676774	244676833	chr1:244676774-244676833	0.49	0.60	0.11
A_14_P103402	244686946	244687005	chr1:244686946-244687005	0.46	0.98	-0.46
A_16_P00296776	244693374	244693433	chr1:244693374-244693433	0.44	0.86	-0.57
A_16_P35544658	244702116	244702175	chr1:244702116-244702175	0.12	0.58	-0.67
A_16_P00296785	244707779	244707838	chr1:244707779-244707838	0.04	0.68	-0.50
A_16_P15523615	244711518	244711573	chr1:244711518-244711573	0.07	0.11	-0.35
A_16_P35544687	244723414	244723473	chr1:244723414-244723473	0.60	0.94	-0.46
A_16_P00296802	244728879	244728938	OR2M4	1.11	1.22	-0.37
A_16_P15523651	244735042	244735101	chr1:244735042-244735101	0.13	0.83	-0.68
A_16_P35544721	244741016	244741075	chr1:244741016-244741075	0.15	1.60	-0.08
A_16_P15523667	244754692	244754751	chr1:244754692-244754751	0.16	0.80	-0.49
A_16_P35544748	244764447	244764506	chr1:244764447-244764506	0.60	1.07	-0.33
A_16_P35544757	244767489	244767548	chr1:244767489-244767548	0.29	0.75	-0.53
A_16_P15523708	244775642	244775701	chr1:244775642-244775701	0.07	-0.05	-0.54
A_16_P35544770	244781566	244781625	chr1:244781566-244781625	0.11	-0.33	-0.46
A_16_P00296851	244789613	244789672	chr1:244789613-244789672	0.32	0.71	-0.66
A_16_P15523755	244797256	244797315	chr1:244797256-244797315	0.41	1.49	-0.59
A_14_P108926	244806791	244806850	chr1:244806791-244806850	0.40	0.50	-0.52

A_16_P35544829	244815842	244815901	chr1:244815842-244815901	0.19	1.04	-0.51
A_16_P15523786	244821282	244821341	chr1:244821282-244821341	-0.27	-0.37	-0.48
A_16_P15523787	244832583	244832642	chr1:244832583-244832642	0.82	1.85	-0.13
A_16_P00296894	244838154	244838213	OR5BF1	0.27	0.77	-0.40
A_16_P35544878	244841278	244841337	chr1:244841278-244841337	0.05	1.29	-0.75
A_16_P35544896	244847328	244847387	chr1:244847328-244847387	0.42	0.27	-0.47
A_16_P35544917	244852552	244852611	chr1:244852552-244852611	0.52	0.45	-0.55
A_16_P00296933	244857272	244857331	chr1:244857272-244857331	-0.07	0.53	-0.31
A_16_P15523883	244865778	244865837	chr1:244865778-244865837	0.05	0.96	-0.50
A_16_P15523900	244870377	244870436	chr1:244870377-244870436	0.28	0.48	-0.50
A_16_P35544976	244874847	244874906	chr1:244874847-244874906	0.42	2.02	-0.43
A_16_P15523935	244881634	244881689	chr1:244881634-244881689	1.01	0.83	-0.42
A_16_P35545003	244889818	244889877	chr1:244889818-244889877	0.30	0.97	-0.47
A_16_P15523964	244895428	244895487	OR2T1	-0.03	0.62	-0.54
A_16_P15523973	244899416	244899475	chr1:244899416-244899475	-0.01	0.44	-0.57
A_16_P15523984	244908381	244908440	chr1:244908381-244908440	0.58	1.07	-0.35
A_16_P35545051	244918438	244918497	chr1:244918438-244918497	-0.45	-0.49	-0.74
A_16_P35545077	244930595	244930639	chr1:244930595-244930639	0.57	1.10	-0.39
A_16_P00297017	244936269	244936328	chr1:244936269-244936328	0.48	1.82	0.20
A_16_P35545117	244942436	244942481	OR2T2	0.78	0.90	-0.22
A_16_P00297021	244947984	244948043	chr1:244947984-244948043	0.47	1.10	-0.53
A_16_P35545145	244955806	244955865	chr1:244955806-244955865	0.46	1.27	-0.62
A_16_P00297029	244961779	244961838	chr1:244961779-244961838	1.02	2.14	-0.47
A_16_P00297031	244970723	244970782	chr1:244970723-244970782	0.23	1.05	-0.44
A_16_P00297032	244972775	244972834	chr1:244972775-244972834	0.92	2.35	0.25
A_16_P15524187	245009770	245009829	chr1:245009770-245009829	-0.47	-0.35	-0.65
A_16_P15524204	245014643	245014702	chr1:245014643-245014702	0.19	0.23	-0.44
A_16_P15524269	245053940	245053999	chr1:245053940-245053999	-1.22	0.58	-0.99
A_16_P35545330	245054641	245054700	chr1:245054641-245054700	-1.60	-0.67	-1.30
A_16_P35545350	245064939	245064998	chr1:245064939-245064998	-0.76	-1.02	-0.93
A_16_P15524307	245071941	245072000	chr1:245071941-245072000	0.31	1.58	-0.56
A_16_P15524319	245082256	245082315	OR2T10	-1.01	0.04	-0.54
A_16_P35545405	245090799	245090858	chr1:245090799-245090858	-0.82	0.16	-0.46
A_16_P15524360	245098539	245098598	chr1:245098539-245098598	-0.53	0.16	-0.40
A_16_P15524371	245107236	245107295	chr1:245107236-245107295	-1.09	-0.29	-0.66
A_16_P35545438	245111544	245111603	chr1:245111544-245111603	-0.54	0.13	-0.38
A_16_P35545459	245118238	245118297	chr1:245118238-245118297	-0.25	0.04	-0.47
A_16_P15524410	245121447	245121506	chr1:245121447-245121506	-1.22	-0.02	-0.35
A_16_P00297115	245134410	245134469	chr1:245134410-245134469	0.04	0.50	-1.48
A_16_P35545529	245140268	245140327	chr1:245140268-245140327	0.09	0.17	-0.82
A_16_P15524494	245150122	245150181	chr1:245150122-245150181	0.92	1.66	-0.19
A_16_P15524510	245161888	245161944	chr1:245161888-245161944	0.73	1.24	-0.34
A_16_P35545596	245170669	245170728	chr1:245170669-245170728	0.24	0.91	-0.39
A_16_P15524555	245176200	245176259	chr1:245176200-245176259	0.41	1.46	-0.35
A_16_P15524588	245187872	245187931	chr1:245187872-245187931	0.50	1.66	-0.37
A_16_P15524604	245197794	245197853	chr1:245197794-245197853	0.99	1.26	-0.15
A_16_P35545672	245210791	245210850	BC033943	0.86	1.15	-0.21
A_16_P00297193	245275177	245275236	chr1:245275177-245275236	0.74	1.13	-0.20

A_16_P00297210	245291091	245291150	chr1:245291091-245291150	0.77	0.84	0.05
A_16_P15524658	245299678	245299732	chr1:245299678-245299732	-0.14	1.71	-0.23
A_16_P15524669	245308564	245308623	chr1:245308564-245308623	1.05	1.04	-0.10
A_14_P200712	245314354	245314405	chr1:245314354-245314405	0.75	0.75	-0.23
A_14_P105106	245314409	245314467	SH3BP5L	0.80	0.17	-0.16
A_16_P15524705	245319342	245319401	SH3BP5L	0.94	0.48	-0.40
A_16_P35545780	245323107	245323166	SH3BP5L	0.90	0.95	-0.31
A_14_P134962	245328092	245328151	SH3BP5L	0.77	1.19	-0.46
A_16_P15524752	245333557	245333616	chr1:245333557-245333616	0.72	0.57	-0.04
A_16_P15524765	245338252	245338311	chr1:245338252-245338311	0.77	0.67	-0.20
A_16_P00297283	245341555	245341614	chr1:245341555-245341614	0.54	1.14	-0.47
A_16_P15524788	245345172	245345231	ZNF672	0.70	0.73	-0.39
A_14_P103141	245348547	245348602	ZNF672	0.79	1.37	-0.08
A_16_P00297306	245353600	245353659	chr1:245353600-245353659	0.35	0.28	-0.28
A_14_P120601	245361556	245361610	ZNF692	1.06	0.20	0.02
A_16_P00297329	245368527	245368586	chr1:245368527-245368586	0.99	0.60	-0.15
A_14_P107408	245382561	245382620	chr1:245382561-245382620	0.97	1.12	-0.10
A_16_P15524897	245395200	245395259	chr1:245395200-245395259	0.95	0.74	-0.27
A_16_P15524902	245407454	245407513	chr1:245407454-245407513	0.90	0.89	-0.14
A_16_P00297364	245412988	245413045	PGBD2	1.22	0.42	-0.14
A_16_P00297372	245418024	245418083	PGBD2	1.55	1.45	-0.01
A_14_P105372	245422360	245422419	PGBD2	0.62	1.41	-0.34
A_16_P00297384	245428484	245428543	chr1:245428484-245428543	0.05	0.14	-0.40
A_16_P00297387	245433846	245433898	chr1:245433846-245433898	0.81	0.86	-0.12
