# Supplementary material for: Genetic alterations of histone lysine methyltransferases and their significance in breast cancer
Source: Oncotarget. 2014 Dec 11;6(4):2466–82. doi: 10.18632/oncotarget.2967 (PMC4385864; doi:10.18632/oncotarget.2967)
Supplement: Supplementary file 6 [file oncotarget-06-2466-s006.pdf]

Table S5. Summary of log-rank statistical analysis of overall survival for 44 HMT CNAs in breast cancer

Gene	Three Group Analysis		Diploid vs Gain/Amp				Loss vs Diploid			
	Chi square	P value	Chi square	P value	Ratio	95% CI of ratio	Chi square	P value	Ratio	95% CI of ratio
ASH1L	0.185	0.9116	0.1854	0.6668	0.8968	0.5526 to 1.460	0.07655	0.782	1.321	0.1417 to 13.55
DOT1L	1.064	0.5874	0.02245	0.8809	0.9445	0.4370 to 2.032	1.06	0.3033	1.277	0.7957 to 2.095
EHMT1	2.428	0.2971	2.702	0.1002	0.603	0.2628 to 1.117	0.5102	0.4751	1.202	0.7159 to 2.053
EHMT2	1.612	0.4466	1.622	0.2028	0.731	0.4276 to 1.197	0.3471	0.5558	1.234	0.5871 to 2.727
EZH1	1.471	0.4793	1.137	0.2863	0.7324	0.3894 to 1.319	1.09	0.2964	1.299	0.7925 to 2.151
EZH2	5.829	0.0542	1.925	0.1653	1.487	0.8600 to 2.436	2.444	0.118	1.545	0.8842 to 3.081
KMT2A	5.257	0.0722	2.439	0.1183	0.5478	0.1793 to 1.204	4.37	0.0366	1.621	1.036 to 2.611
KMT2C	7.482	0.0237	3.002	0.0831	1.66	0.9427 to 2.684	2.503	0.1136	1.528	0.8952 to 2.931
KMT2E	4.636	0.0985	1.192	0.2748	1.34	0.8021 to 2.185	2.653	0.1034	1.644	0.8918 to 3.761
MECOM	0.1624	0.922	0.1718	0.6785	0.9054	0.5598 to 1.458	0.009883	0.9208	1.044	0.4408 to 2.477
NSD1	4.29	0.117	3.218	0.0728	0.6288	0.3344 to 1.042	2.495	0.1142	1.552	0.8909 to 3.051
PRDM1	7.622	0.0221	7.179	0.0074	0.4869	0.2091 to 0.7730	0.7083	0.4	1.245	0.7392 to 2.146
PRDM10	4.419	0.1098	3.002	0.0832	0.5434	0.1972 to 1.097	2.605	0.1066	1.46	0.9240 to 2.358
PRDM11	0.2544	0.8805	0.05891	0.8082	0.9371	0.5481 to 1.597	0.2223	0.6373	1.167	0.5952 to 2.347
PRDM12	3.122	0.21	3.033	0.0816	0.5849	0.2493 to 1.078	1.176	0.2782	1.317	0.7907 to 2.281
PRDM15	1.136	0.5666	1.134	0.2869	0.7598	0.4347 to 1.277	0.2819	0.5954	1.176	0.6332 to 2.220
PRDM16	3.737	0.1543	1.878	0.1706	0.6289	0.2664 to 1.259	3.06	0.0802	1.511	0.9522 to 2.511
PRDM2	3.196	0.2023	2.829	0.0926	0.5297	0.1665 to 1.138	1.105	0.2932	1.278	0.8053 to 2.061
PRDM4	1.805	0.4055	0.2602	0.61	0.857	0.4554 to 1.585	1.807	0.1788	1.427	0.8373 to 2.614
PRDM5	6.533	0.0381	7.088	0.0078	0.434	0.1329 to 0.7294	0.9707	0.3245	1.279	0.7758 to 2.157
PRDM6	2.051	0.3585	2.128	0.1446	0.6405	0.2876 to 1.186	0.4488	0.5029	1.189	0.7080 to 2.025
PRDM8	10.53	0.0052	12.52	0.0004	0.3579	0.09002 to 0.4915	1.34	0.2469	1.337	0.8095 to 2.284
SETD1A	0.2456	0.8844	0.1922	0.6611	1.108	0.6941 to 1.786	0.1402	0.7081	0.8582	0.3889 to 1.885
SETD1B	2.391	0.3026	0.3547	0.5514	0.8381	0.4496 to 1.530	2.411	0.1205	1.501	0.8900 to 2.767
SETD2	8.708	0.0129	0.3531	0.5523	1.295	0.5800 to 2.777	7.007	0.0081	1.825	1.188 to 3.124
SETD3	0.8668	0.6483	0.3764	0.5395	0.8242	0.4227 to 1.568	0.8331	0.3614	1.255	0.7628 to 2.120
SETD4	4.518	0.1044	4.501	0.0339	0.5956	0.3273 to 0.9536	0.2444	0.6211	1.174	0.6061 to 2.319
SETD5	4.483	0.1063	4.281	0.0385	0.5938	0.3148 to 0.9679	0.9816	0.3218	1.375	0.7073 to 2.886
SETD6	0.38	0.827	0.2284	0.6327	0.8357	0.3774 to 1.796	0.3475	0.5556	1.165	0.7076 to 1.905
SETD7	4.009	0.1347	4.567	0.0326	0.4969	0.1702 to 0.9187	0.5376	0.4634	1.198	0.7324 to 1.983
SETD8	2.752	0.2525	2.156	0.142	0.6655	0.3445 to 1.162	1.66	0.1976	1.414	0.8227 to 2.584
SETDB1	0.7877	0.6745	0.007148	0.9326	0.9794	0.6040 to 1.588	1.293	0.2555	2.24	0.4258 to 26.57
SETDB2	8.797	0.0123	4.022	0.0449	0.4752	0.1456 to 0.9733	8.109	0.0044	1.919	1.261 to 3.202
SETMAR	6.486	0.0391	5.01	0.0252	0.5615	0.2858 to 0.9188	3.715	0.0539	1.742	0.9916 to 3.787
SMYD2	2.582	0.275	0.02046	0.8863	1.038	0.6210 to 1.736	2.524	0.1121	2.269	0.7875 to 14.79
SMYD3	3.29	0.1931	1.003	0.3166	1.286	0.7739 to 2.216	1.276	0.2586	1.721	0.6152 to 6.453
SMYD4	0.8805	0.6439	0.005207	0.9425	0.9655	0.3651 to 2.549	0.8297	0.3623	1.247	0.7813 to 1.974
SMYD5	2.114	0.3475	0.03514	0.8513	1.055	0.6045 to 1.841	1.932	0.1646	0.5595	0.3152 to 1.210
SUV39H1	0.733	0.6932	0.0008186	0.9772	1.008	0.5678 to 1.791	0.6667	0.4142	1.268	0.6988 to 2.401
SUV39H2	3.296	0.1924	2.728	0.0986	0.6767	0.4025 to 1.078	1.873	0.1712	1.633	0.7789 to 4.324
SUV420H1	1.949	0.3773	1.841	0.1748	0.7223	0.4304 to 1.164	0.01142	0.9149	0.962	0.4738 to 1.952
SUV420H2	0.1318	0.9362	0.02887	0.8651	0.9567	0.5688 to 1.606	0.1533	0.6954	1.129	0.6015 to 2.148
WHSC1	4.866	0.0878	5.32	0.0211	0.4579	0.1355 to 0.8411	0.4168	0.5185	1.169	0.7225 to 1.907
WHSC1L1	1.986	0.3705	1.791	0.1808	0.708	0.4313 to 1.169	0.2317	0.6302	1.159	0.6320 to 2.135
