# Supplementary material for: Genetic alterations of histone lysine methyltransferases and their significance in breast cancer
Source: Oncotarget. 2014 Dec 11;6(4):2466–82. doi: 10.18632/oncotarget.2967 (PMC4385864; doi:10.18632/oncotarget.2967)
Supplement: Supplementary file 7 [file oncotarget-06-2466-s007.pdf]

Table S6. Summary of log-rank statistical analysis of overall survival for 44 HMT expressions in breast cancer

Gene	Chi square	P value	Hazard Ratio	95% CI of ratio
SETD7	9.455	0.0021	1.944	1.303 to 3.196
WHSC1L1	5.16	0.0231	1.659	1.074 to 2.571
SETD5	3.995	0.0456	1.548	1.013 to 2.455
SUV39H2	3.822	0.0506	1.559	1.001 to 2.394
ASH1L	3.552	0.0595	1.507	0.9890 to 2.393
KMT2A	3.42	0.0644	1.495	0.9827 to 2.368
NSD1	3.281	0.0701	1.483	0.9730 to 2.370
SETD8	3.128	0.0769	0.6788	0.4307 to 1.040
EHMT2	2.741	0.0978	0.696	0.4424 to 1.067
PRDM2	2.214	0.1368	1.385	0.9021 to 2.194
SETMAR	2.038	0.1534	0.7309	0.4662 to 1.124
DOT1L	1.8	0.1797	1.349	0.8727 to 2.086
PRDM15	1.637	0.2008	1.327	0.8608 to 2.060
PRDM4	1.447	0.2289	1.303	0.8455 to 2.039
EZH2	1.439	0.2303	0.7668	0.4945 to 1.183
PRDM10	1.29	0.256	1.284	0.8325 to 2.006
SMYD4	1.235	0.2664	1.277	0.8290 to 1.991
SETDB1	1.155	0.2824	0.7883	0.5084 to 1.217
SMYD3	1.126	0.2887	0.7902	0.5104 to 1.220
EHMT1	0.9746	0.3235	0.804	0.5185 to 1.240
PRDM11	0.9489	0.33	0.8059	0.5203 to 1.244
PRDM16	0.8648	0.3524	1.228	0.7966 to 1.905
KMT2C	0.7791	0.3774	1.213	0.7874 to 1.896
EZH1	0.6071	0.4359	0.8418	0.5434 to 1.299
SUV39H1	0.4489	0.5029	1.161	0.7511 to 1.795
SUV420H1	0.4019	0.5261	1.151	0.7450 to 1.781
PRDM5	0.383	0.536	1.146	0.7418 to 1.781
PRDM12	0.3552	0.5512	0.8763	0.5664 to 1.353
SETD1B	0.3029	0.5821	0.8847	0.5718 to 1.368
SETD4	0.2989	0.5846	0.8859	0.5702 to 1.372
SETD1A	0.2819	0.5955	0.8891	0.5697 to 1.379
SETD6	0.2424	0.6225	1.115	0.7219 to 1.725
SMYD5	0.2231	0.6367	1.112	0.7168 to 1.726
SETD2	0.1903	0.6627	1.101	0.7126 to 1.708
KMT2E	0.1788	0.6724	1.097	0.7109 to 1.701
PRDM6	0.1119	0.738	0.9283	0.5995 to 1.436
SETD3	0.076	0.7828	0.9407	0.6078 to 1.454
SMYD2	0.03921	0.843	0.9571	0.6189 to 1.479
MECOM	0.01139	0.915	1.024	0.6573 to 1.598
PRDM8	0.009362	0.9229	1.021	0.6599 to 1.583
SUV420H2	0.005481	0.941	0.9837	0.6350 to 1.523
PRDM1	0.002798	0.9578	1.012	0.6528 to 1.569
SETDB2	0.002532	0.9599	1.011	0.6542 to 1.563
WHSC1	0.0001057	0.9918	1.002	0.6484 to 1.549
