# Supplementary material for: Genetic alterations of histone lysine methyltransferases and their significance in breast cancer
Source: Oncotarget. 2014 Dec 11;6(4):2466–82. doi: 10.18632/oncotarget.2967 (PMC4385864; doi:10.18632/oncotarget.2967)
Supplement: Supplementary file 8 [file oncotarget-06-2466-s008.pdf]

Table S7. Summary of multivariate analysis of overall survival for 44 HMT CNAs in breast cancer

Multivariate	Age	ER.Status	PR.Status	HER2.Final.Status	Tumor Size	Lymph Node	Metastasis	PAM50 Subtypes	ASH1L Gain/Amp
Hazard Ratio	1.03907	0.6248	0.295	0.74393	1.03466	1.99736	2.65425	0.71938	0.57027
P-value	0.0031	0.4211	0.0066	0.6204	0.9346	0.0583	0.0601	0.5653	0.1473
95% Conf. Interval	1.013	0.1987	0.1223	0.2308	0.4584	0.976	0.9593	0.2341	0.2668
	1.0658	1.9652	0.7118	2.3983	2.3354	4.0874	7.3439	2.2105	1.219
Multivariate	Age	ER.Status	PR.Status	HER2.Final.Status	Tumor Size	Lymph Node	Metastasis	PAM50 Subtypes	DOT1L Gain/Amp
Hazard Ratio	1.04431	0.6562	0.27676	0.20121	0.92906	2.28451	1.42365	0.60682	0.79275
P-value	0.00989	0.6021	0.03637	0.14288	0.88097	0.06554	0.63502	0.55747	0.66819
95% Conf. Interval	1.01047	0.13466	0.08311	0.02356	0.35464	0.94825	0.33113	0.11435	0.27411
	1.0793	3.1977	0.9217	1.7186	2.4339	5.5038	6.1208	3.2203	2.2927
Multivariate	Age	ER.Status	PR.Status	HER2.Final.Status	Tumor Size	Lymph Node	Metastasis	PAM50 Subtypes	DOT1L Loss
Hazard Ratio	1.03028	0.91118	0.27906	0.77937	1.14227	1.73638	2.36883	0.74048	0.91264
P-value	0.03613	0.87393	0.00588	0.67195	0.77107	0.16409	0.101	0.6203	0.80253
95% Conf. Interval	1.0019	0.2888	0.1125	0.2459	0.4663	0.7982	0.8452	0.2256	0.4458
	1.059	2.875	0.692	2.471	2.798	3.778	6.639	2.431	1.868
Multivariate	Age	ER.Status	PR.Status	HER2.Final.Status	Tumor Size	Lymph Node	Metastasis	PAM50 Subtypes	EHMT1 Gain/Amp
Hazard Ratio	1.038881	0.497019	0.440502	0.467239	1.001355	2.356177	2.937554	0.473001	1.697166
P-value	0.0106	0.3211	0.1351	0.2731	0.9978	0.0563	0.0883	0.3133	0.2481
95% Conf. Interval	1.0089	0.1249	0.1503	0.1198	0.3853	0.9774	0.8508	0.1104	0.6916
	1.07	1.977	1.291	1.822	2.603	5.68	10.143	2.027	4.165
Multivariate	Age	ER.Status	PR.Status	HER2.Final.Status	Tumor Size	Lymph Node	Metastasis	PAM50 Subtypes	EHMT1 Loss
Hazard Ratio	1.02048	1.37813	0.22144	0.84235	1.15788	1.51207	2.7902	0.83089	1.05203
P-value	0.18847	0.62618	0.00356	0.79723	0.76034	0.30432	0.05408	0.77489	0.89707
95% Conf. Interval	0.99011	0.37917	0.08034	0.22758	0.45143	0.68698	0.98218	0.23343	0.48782
	1.0518	5.0089	0.6103	3.1178	2.9699	3.3281	7.9265	2.9575	2.2688
Multivariate	Age	ER.Status	PR.Status	HER2.Final.Status	Tumor Size	Lymph Node	Metastasis	PAM50 Subtypes	EHMT2 Gain/Amp
Hazard Ratio	1.03101	0.74717	0.21985	0.5947	1.03384	2.04938	2.46841	0.62579	1.07771
P-value	0.02938	0.62823	0.00142	0.44144	0.94006	0.0687	0.11182	0.41026	0.84255
95% Conf. Interval	1.00307	0.22965	0.08671	0.15835	0.43427	0.94648	0.81042	0.20506	0.51497
	1.0597	2.431	0.5574	2.2335	2.4612	4.4374	7.5184	1.9098	2.2554
Multivariate	Age	ER.Status	PR.Status	HER2.Final.Status	Tumor Size	Lymph Node	Metastasis	PAM50 Subtypes	EHMT2 Loss
Hazard Ratio	1.03915	1.05564	0.20528	0.44376	1.13439	2.34848	1.206	0.1906	1.58645
P-value	0.00727	0.93597	0.00272	0.24512	0.80702	0.05642	0.74261	0.05546	0.33382
95% Conf. Interval	1.01042	0.28176	0.0729	0.11276	0.41246	0.97693	0.39433	0.03495	0.62224
	1.069	3.955	0.578	1.746	3.12	5.646	3.688	1.039	4.045

Multivariate	Age	ER.Status	PR.Status	HER2.Final.Status	Tumor Size	Lymph Node	Metastasis	PAM50 Subtypes	EZH1 Gain/Amp
Hazard Ratio	1.02245	0.26674	1.24037	1.9144	0.81229	2.34995	2.3034	1.07817	1.69515
P-value	0.2123	0.2127	0.8195	0.3501	0.6843	0.0656	0.2977	0.9231	0.2916
95% Conf. Interval	0.98739	0.03338	0.19497	0.49034	0.29819	0.94632	0.47903	0.23372	0.63573
	1.059	2.132	7.891	7.474	2.213	5.836	11.076	4.974	4.52

Multivariate	Age	ER.Status	PR.Status	HER2.Final.Status	Tumor Size	Lymph Node	Metastasis	PAM50 Subtypes	EZH1 Loss
Hazard Ratio	1.026226	0.900758	0.235052	0.499446	1.013802	1.593546	2.57992	0.647686	0.998725
P-value	0.07043	0.8697	0.00795	0.34679	0.97591	0.24805	0.08883	0.48939	0.99743
95% Conf. Interval	0.99784	0.25839	0.08069	0.11759	0.41645	0.72276	0.86596	0.18903	0.45894
	1.0554	3.1401	0.6847	2.1213	2.468	3.5135	7.6862	2.2192	2.1734

Multivariate	Age	ER.Status	PR.Status	HER2.Final.Status	Tumor Size	Lymph Node	Metastasis	PAM50 Subtypes	EZH2 Gain/Amp
Hazard Ratio	1.02484	1.16012	0.19015	0.5411	1.02961	1.89995	2.4124	0.50866	0.7183
P-value	0.09822	0.82097	0.00175	0.43318	0.95124	0.13391	0.09286	0.29276	0.39751
95% Conf. Interval	0.99546	0.32052	0.06724	0.11649	0.40404	0.82081	0.86377	0.14438	0.33376
	1.0551	4.1991	0.5378	2.5134	2.6238	4.3978	6.7376	1.792	1.5459

Multivariate	Age	ER.Status	PR.Status	HER2.Final.Status	Tumor Size	Lymph Node	Metastasis	PAM50 Subtypes	EZH2 Loss
Hazard Ratio	1.03661	0.47417	0.39501	0.47481	2.47797	1.27935	1.48702	0.51928	1.65065
P-value	0.0203	0.2669	0.0958	0.2218	0.0939	0.535	0.5519	0.2965	0.2711
95% Conf. Interval	1.0056	0.127	0.1324	0.1437	0.8572	0.5874	0.4024	0.1517	0.6761
	1.069	1.77	1.179	1.568	7.164	2.786	5.496	1.777	4.03

Multivariate	Age	ER.Status	PR.Status	HER2.Final.Status	Tumor Size	Lymph Node	Metastasis	PAM50 Subtypes	KMT2A Gain/Amp
Hazard Ratio	1.04495	0.44698	0.56167	0.37186	0.94625	1.67471	2.82063	0.66977	1.75812
P-value	0.0296	0.4066	0.4953	0.374	0.9265	0.33	0.1711	0.6403	0.3559
95% Conf. Interval	1.00437	0.06674	0.107	0.04199	0.2926	0.59339	0.63899	0.12471	0.53066
	1.087	2.994	2.948	3.293	3.06	4.726	12.451	3.597	5.825

Multivariate	Age	ER.Status	PR.Status	HER2.Final.Status	Tumor Size	Lymph Node	Metastasis	PAM50 Subtypes	KMT2A Loss
Hazard Ratio	1.04116	0.71522	0.27017	0.50177	0.93879	2.45541	2.08458	0.76453	1.78219
P-value	0.0044	0.58481	0.00433	0.30771	0.88545	0.02482	0.14025	0.65132	0.08242
95% Conf. Interval	1.0127	0.2149	0.11	0.1334	0.3976	1.1205	0.7854	0.2386	0.9284
	1.0705	2.3801	0.6639	1.8879	2.2168	5.3806	5.5331	2.4496	3.421

Multivariate	Age	ER.Status	PR.Status	HER2.Final.Status	Tumor Size	Lymph Node	Metastasis	PAM50 Subtypes	KMT2E Gain/Amp
Hazard Ratio	1.03017	0.84305	0.22679	0.61485	1.09144	1.77523	2.47873	0.554	0.82079
P-value	0.03166	0.7806	0.00386	0.46484	0.84197	0.14352	0.08548	0.30465	0.58264
95% Conf. Interval	1.00261	0.25357	0.08289	0.16685	0.4618	0.82278	0.88091	0.17938	0.40583
	1.0585	2.8029	0.6204	2.2657	2.5796	3.8302	6.9747	1.711	1.6601

Multivariate	Age	ER.Status	PR.Status	HER2.Final.Status	Tumor Size	Lymph Node	Metastasis	PAM50 Subtypes	KMT2E Loss
Hazard Ratio	1.03183	0.95046	0.3349	0.63777	1.95766	1.31855	1.81098	0.58787	0.94504
P-value	0.0514	0.9429	0.0635	0.4544	0.2151	0.5139	0.454	0.4812	0.9215
95% Conf. Interval	0.9998	0.2366	0.1055	0.1963	0.6768	0.5747	0.3827	0.1341	0.3068
	1.065	3.818	1.063	2.072	5.662	3.025	8.571	2.578	2.911

Multivariate	Age	ER.Status	PR.Status	HER2.Final.Status	Tumor Size	Lymph Node	Metastasis	PAM50 Subtypes	MECOM Gain/Amp
Hazard Ratio	1.04443	0.5825	0.25206	0.53747	1.24925	1.92323	2.00174	0.41997	1.55914
P-value	0.0012	0.358	0.00303	0.29068	0.61404	0.08259	0.17293	0.14213	0.21074
95% Conf. Interval	1.0173	0.184	0.1014	0.1699	0.5261	0.919	0.7378	0.1319	0.7777
	1.0723	1.844	0.6268	1.7003	2.9667	4.0246	5.4308	1.3375	3.1257

Multivariate	Age	ER.Status	PR.Status	HER2.Final.Status	Tumor Size	Lymph Node	Metastasis	PAM50 Subtypes	NSD1 Gain/Amp
Hazard Ratio	1.02717	0.67181	0.57875	1.1569	0.69214	2.06364	3.9023	0.54844	2.65694
P-value	0.0724	0.6299	0.3544	0.815	0.4209	0.097	0.027	0.4672	0.0153
95% Conf. Interval	0.9976	0.1332	0.1819	0.3412	0.2825	0.877	1.1672	0.1086	1.2064
	1.058	3.389	1.841	3.923	1.696	4.856	13.046	2.77	5.852

Multivariate	Age	ER.Status	PR.Status	HER2.Final.Status	Tumor Size	Lymph Node	Metastasis	PAM50 Subtypes	PRDM10 Gain/Amp
Hazard Ratio	1.04681	0.45673	0.5419	0.37583	0.91971	1.4376	3.35921	0.66776	2.31939
P-value	0.0292	0.4259	0.4785	0.3804	0.8808	0.4783	0.1146	0.6457	0.1346
95% Conf. Interval	1.00465	0.06635	0.09957	0.04221	0.30796	0.52712	0.74558	0.11937	0.7705
	1.091	3.144	2.949	3.346	2.747	3.921	15.135	3.736	6.982

Multivariate	Age	ER.Status	PR.Status	HER2.Final.Status	Tumor Size	Lymph Node	Metastasis	PAM50 Subtypes	PRDM10 Loss
Hazard Ratio	1.04169	0.73251	0.27395	0.50584	0.90029	2.53331	2.02838	0.69225	2.07262
P-value	0.00428	0.61643	0.00462	0.31162	0.82374	0.02657	0.15906	0.54284	0.0345
95% Conf. Interval	1.0129	0.2167	0.1118	0.1351	0.3573	1.1141	0.758	0.2117	1.0546
	1.0713	2.476	0.6711	1.8939	2.2688	5.7604	5.428	2.2634	4.0734

Multivariate	Age	ER.Status	PR.Status	HER2.Final.Status	Tumor Size	Lymph Node	Metastasis	PAM50 Subtypes	PRDM11 Gain/Amp
Hazard Ratio	1.03058	1.10621	0.31982	0.78576	0.92243	1.77151	2.09054	0.9668	1.48181
P-value	0.0246	0.8704	0.0185	0.6823	0.8459	0.1262	0.1681	0.9548	0.2488
95% Conf. Interval	1.0039	0.3292	0.1238	0.2477	0.4085	0.8513	0.7326	0.3009	0.7595
	1.058	3.718	0.826	2.492	2.083	3.686	5.965	3.107	2.891

Multivariate	Age	ER.Status	PR.Status	HER2.Final.Status	Tumor Size	Lymph Node	Metastasis	PAM50 Subtypes	PRDM11 Loss
Hazard Ratio	1.04309	0.88606	0.20869	0.3484	1.02219	1.75454	1.71106	0.22783	1.33291
P-value	0.00785	0.85351	0.0017	0.19248	0.96633	0.22886	0.41578	0.04562	0.6191
95% Conf. Interval	1.01114	0.24535	0.07845	0.07135	0.36892	0.70221	0.4693	0.05342	0.4293
	1.076	3.1999	0.5551	1.7011	2.8323	4.3839	6.2386	0.9716	4.1385

Multivariate	Age	ER.Status	PR.Status	HER2.Final.Status	Tumor Size	Lymph Node	Metastasis	PAM50 Subtypes	PRDM12 Gain/Amp

Hazard Ratio	1.02895	0.39352	0.47897	0.27388	0.66053	3.17934	4.05322	0.58086	2.46299
P-value	0.0686	0.1826	0.1847	0.1188	0.3976	0.0147	0.033	0.4241	0.0532
95% Conf. Interval	0.99783	0.09984	0.16142	0.0538	0.25267	1.25515	1.11959	0.15332	0.98748
	1.061	1.551	1.421	1.394	1.727	8.053	14.674	2.201	6.143
Multivariate	Age	ER.Status	PR.Status	HER2.Final.Status	Tumor Size	Lymph Node	Metastasis	PAM50 Subtypes	PRDM12 Loss
Hazard Ratio	1.02539	1.14851	0.26206	1.3329	1.53273	1.4356	2.79795	0.67445	1.2252
P-value	0.1053	0.8452	0.0143	0.6275	0.4169	0.3764	0.0518	0.5693	0.5971
95% Conf. Interval	0.99474	0.28619	0.08979	0.41755	0.54659	0.64427	0.99179	0.17369	0.57692
	1.057	4.6091	0.7649	4.2549	4.298	3.1989	7.8933	2.619	2.602
Multivariate	Age	ER.Status	PR.Status	HER2.Final.Status	Tumor Size	Lymph Node	Metastasis	PAM50 Subtypes	PRDM15 Gain/Amp
Hazard Ratio	1.0252	0.63932	0.31664	0.31423	1.03145	1.47372	3.20451	0.47365	1.45943
P-value	0.0772	0.4683	0.0177	0.1544	0.9427	0.3225	0.0676	0.2077	0.3281
95% Conf. Interval	0.99729	0.19083	0.12238	0.06388	0.44353	0.68357	0.91917	0.14812	0.68408
	1.0539	2.1419	0.8193	1.5457	2.3987	3.1773	11.172	1.5147	3.1136
Multivariate	Age	ER.Status	PR.Status	HER2.Final.Status	Tumor Size	Lymph Node	Metastasis	PAM50 Subtypes	PRDM15 Loss
Hazard Ratio	1.039352	1.067885	0.37037	1.622133	0.574495	2.56458	1.674155	1.30179	1.002781
P-value	0.0268	0.9263	0.0967	0.4475	0.2686	0.0361	0.4248	0.7198	0.9952
95% Conf. Interval	1.0044	0.2653	0.1147	0.4656	0.2152	1.0632	0.4723	0.308	0.4037
	1.075	4.298	1.196	5.651	1.534	6.186	5.934	5.501	2.491
Multivariate	Age	ER.Status	PR.Status	HER2.Final.Status	Tumor Size	Lymph Node	Metastasis	PAM50 Subtypes	PRDM16 Gain/Amp
Hazard Ratio	1.02473	0.41519	0.4321	0.30098	1.02782	1.8862	1.43041	0.29192	2.52797
P-value	0.174	0.286	0.309	0.162	0.959	0.168	0.712	0.106	0.14
95% Conf. Interval	0.98924	0.08272	0.08594	0.05601	0.35861	0.76467	0.21354	0.06559	0.73853
	1.061	2.084	2.173	1.617	2.946	4.653	9.582	1.299	8.653
Multivariate	Age	ER.Status	PR.Status	HER2.Final.Status	Tumor Size	Lymph Node	Metastasis	PAM50 Subtypes	PRDM16 Loss
Hazard Ratio	1.03222	1.20561	0.30153	1.11925	0.73882	1.8672	1.81447	0.60409	1.52446
P-value	0.0284	0.7921	0.0272	0.8504	0.5051	0.125	0.3066	0.4753	0.2642
95% Conf. Interval	1.0034	0.3001	0.104	0.347	0.3033	0.8409	0.5791	0.1514	0.7272
	1.0619	4.843	0.8739	3.6102	1.7995	4.1461	5.6853	2.4102	3.1958
Multivariate	Age	ER.Status	PR.Status	HER2.Final.Status	Tumor Size	Lymph Node	Metastasis	PAM50 Subtypes	PRDM2 Loss
Hazard Ratio	1.04037	1.01065	0.24127	0.9028	0.96776	1.92496	1.3618	0.71187	1.25997
P-value	0.00445	0.98615	0.00477	0.86262	0.93988	0.08778	0.63131	0.56013	0.51558
95% Conf. Interval	1.01238	0.30564	0.08988	0.2835	0.41288	0.90759	0.3858	0.22693	0.6278
	1.0691	3.3419	0.6477	2.8749	2.2683	4.0827	4.807	2.2331	2.5287
Multivariate	Age	ER.Status	PR.Status	HER2.Final.Status	Tumor Size	Lymph Node	Metastasis	PAM50 Subtypes	PRDM4 Gain/Amp
Hazard Ratio	1.022746	0.931653	0.309623	1.03932	1.174293	1.022155	3.682068	0.99449	1.429868

P-value	0.1531	0.9154	0.0167	0.9487	0.7468	0.9598	0.0188	0.9934	0.4155
95% Conf. Interval	0.9917	0.2523	0.1185	0.3212	0.4428	0.4361	1.2418	0.2683	0.6046
	1.0548	3.4397	0.8089	3.3625	3.1143	2.396	10.9179	3.6862	3.3815
Multivariate	Age	ER.Status	PR.Status	HER2.Final.Status	Tumor Size	Lymph Node	Metastasis	PAM50 Subtypes	PRDM4 Loss
Hazard Ratio	1.038479	0.691987	0.350778	0.712594	1.006581	2.154298	2.631448	0.497433	1.549467
P-value	0.0113	0.5699	0.0569	0.5914	0.9882	0.0518	0.0948	0.2751	0.2853
95% Conf. Interval	1.0086	0.1943	0.1193	0.2068	0.4229	0.994	0.8458	0.1419	0.694
	1.069	2.464	1.031	2.456	2.396	4.669	8.187	1.743	3.46
Multivariate	Age	ER.Status	PR.Status	HER2.Final.Status	Tumor Size	Lymph Node	Metastasis	PAM50 Subtypes	PRDM6 Gain/Amp
Hazard Ratio	1.03534	0.93914	0.34438	1.1415	0.74349	1.82476	3.21075	0.94485	1.88041
P-value	0.0208	0.9364	0.0477	0.8414	0.5161	0.1507	0.0558	0.9438	0.1401
95% Conf. Interval	1.0053	0.2009	0.1199	0.3123	0.3039	0.8034	0.9715	0.195	0.8127
	1.0663	4.3892	0.9893	4.1724	1.8189	4.1445	10.611	4.5788	4.3506
Multivariate	Age	ER.Status	PR.Status	HER2.Final.Status	Tumor Size	Lymph Node	Metastasis	PAM50 Subtypes	PRDM6 Loss
Hazard Ratio	1.02878	0.62375	0.20467	0.19973	1.64468	1.51541	2.74528	0.31368	1.43238
P-value	0.06142	0.45802	0.00408	0.05951	0.32418	0.31746	0.07019	0.08447	0.42769
95% Conf. Interval	0.99864	0.17932	0.06933	0.0374	0.61166	0.6708	0.92011	0.08404	0.58942
	1.0598	2.1697	0.6042	1.0667	4.4223	3.4235	8.1909	1.1708	3.4809
Multivariate	Age	ER.Status	PR.Status	HER2.Final.Status	Tumor Size	Lymph Node	Metastasis	PAM50 Subtypes	SETD1A Gain/Amp
Hazard Ratio	1.03021	0.87751	0.2494	0.52727	0.93144	2.3328	2.12991	0.64945	1.03449
P-value	0.02727	0.83333	0.00233	0.33333	0.86839	0.02943	0.13369	0.49823	0.9317
95% Conf. Interval	1.0033	0.2598	0.102	0.1442	0.4021	1.0884	0.7929	0.1862	0.4764
	1.0578	2.9634	0.6096	1.9283	2.1578	5.0002	5.7213	2.2647	2.2463
Multivariate	Age	ER.Status	PR.Status	HER2.Final.Status	Tumor Size	Lymph Node	Metastasis	PAM50 Subtypes	SETD1A Loss
Hazard Ratio	1.07027	0.25543	0.09222	0.06067	1.56922	2.17137	24.06061	0.06905	1.33697
P-value	0.00606	0.17748	0.00422	0.03439	0.54	0.19966	0.00988	0.00872	0.68885
95% Conf. Interval	1.019605	0.03514	0.018021	0.004523	0.371394	0.66394	2.147601	0.009369	0.322683
	1.1235	1.8567	0.472	0.8138	6.6303	7.1013	269.5626	0.5089	5.5394
Multivariate	Age	ER.Status	PR.Status	HER2.Final.Status	Tumor Size	Lymph Node	Metastasis	PAM50 Subtypes	SETD1B Gain/Amp
Hazard Ratio	1.02461	0.93727	0.27525	0.96217	1.14868	1.15277	2.83076	0.86236	1.50943
P-value	0.12659	0.92134	0.00873	0.94957	0.78402	0.73967	0.07317	0.81835	0.33171
95% Conf. Interval	0.9931	0.2591	0.105	0.2913	0.4263	0.4984	0.907	0.2437	0.6573
	1.0571	3.3909	0.7219	3.1784	3.0952	2.6664	8.8353	3.0516	3.4663
Multivariate	Age	ER.Status	PR.Status	HER2.Final.Status	Tumor Size	Lymph Node	Metastasis	PAM50 Subtypes	SETD1B Loss
Hazard Ratio	1.03869	0.74447	0.34046	0.79599	0.90292	2.22655	2.73552	0.44532	1.70241
P-value	0.00981	0.65431	0.0523	0.71888	0.819	0.05212	0.08234	0.21956	0.17416

95% Conf. Interval	1.0092	0.2046	0.1147	0.2298	0.3765	0.9927	0.879	0.1224	0.7903
	1.069	2.709	1.011	2.757	2.165	4.994	8.514	1.62	3.667
Multivariate	Age	ER.Status	PR.Status	HER2.Final.Status	Tumor Size	Lymph Node	Metastasis	PAM50 Subtypes	SETD3 Gain/Amp
Hazard Ratio	1.03594	2.15927	0.17048	1.10362	0.87004	2.64952	1.22298	2.02734	0.75382
P-value	0.03837	0.333	0.00225	0.88573	0.75314	0.02006	0.77993	0.35302	0.5319
95% Conf. Interval	1.0019	0.4544	0.0548	0.2876	0.3654	1.1654	0.298	0.4563	0.3108
	1.0712	10.2597	0.5304	4.2343	2.0717	6.0238	5.0192	9.0083	1.8285
Multivariate	Age	ER.Status	PR.Status	HER2.Final.Status	Tumor Size	Lymph Node	Metastasis	PAM50 Subtypes	SETD3 Loss
Hazard Ratio	1.03635	0.61274	0.34869	0.53184	0.93397	2.23452	3.32146	0.42264	1.40031
P-value	0.0119	0.4439	0.0523	0.3428	0.8841	0.0504	0.0372	0.1866	0.401
95% Conf. Interval	1.0079	0.1748	0.1203	0.1443	0.3727	0.9986	1.074	0.1177	0.6382
	1.066	2.147	1.011	1.96	2.34	5	10.272	1.517	3.072
Multivariate	Age	ER.Status	PR.Status	HER2.Final.Status	Tumor Size	Lymph Node	Metastasis	PAM50 Subtypes	SETD4 Gain/Amp
Hazard Ratio	1.02479	0.65771	0.33797	0.42475	1.02519	1.54407	3.21931	0.54006	1.61249
P-value	0.0774	0.4837	0.0241	0.2089	0.9531	0.25	0.0611	0.2848	0.2075
95% Conf. Interval	0.9973	0.2036	0.1317	0.1117	0.4478	0.7366	0.9471	0.1747	0.7671
	1.053	2.1246	0.8673	1.615	2.347	3.2368	10.9433	1.6699	3.3895
Multivariate	Age	ER.Status	PR.Status	HER2.Final.Status	Tumor Size	Lymph Node	Metastasis	PAM50 Subtypes	SETD4 Loss
Hazard Ratio	1.0331	1.12029	0.36034	1.28505	0.60871	2.19323	1.93653	0.95462	0.80806
P-value	0.0674	0.8791	0.0909	0.7173	0.3292	0.0907	0.3195	0.953	0.6825
95% Conf. Interval	0.9977	0.2594	0.1104	0.3306	0.2246	0.8828	0.5271	0.2035	0.291
	1.07	4.839	1.177	4.994	1.65	5.449	7.115	4.477	2.244
Multivariate	Age	ER.Status	PR.Status	HER2.Final.Status	Tumor Size	Lymph Node	Metastasis	PAM50 Subtypes	SETD5 Gain/Amp
Hazard Ratio	1.02693	0.87293	0.27517	0.68885	0.89327	2.07378	2.52117	0.5783	1.77954
P-value	0.0563	0.8306	0.0113	0.5353	0.7883	0.063	0.103	0.3388	0.0911
95% Conf. Interval	0.9993	0.2513	0.1014	0.212	0.3919	0.9611	0.8297	0.1883	0.9118
	1.0553	3.0328	0.7464	2.2382	2.0362	4.4745	7.6614	1.7762	3.4729
Multivariate	Age	ER.Status	PR.Status	HER2.Final.Status	Tumor Size	Lymph Node	Metastasis	PAM50 Subtypes	SETD5 Loss
Hazard Ratio	1.02177	1.11033	0.37333	0.37875	0.71168	1.41775	3.66749	1.53698	0.74272
P-value	0.196	0.8808	0.1006	0.3639	0.5009	0.4578	0.0478	0.5067	0.5732
95% Conf. Interval	0.98895	0.28266	0.11515	0.04658	0.26431	0.56421	1.01295	0.43222	0.26388
	1.056	4.362	1.21	3.08	1.916	3.562	13.279	5.465	2.09
Multivariate	Age	ER.Status	PR.Status	HER2.Final.Status	Tumor Size	Lymph Node	Metastasis	PAM50 Subtypes	SETD6 Gain/Amp
Hazard Ratio	0.990858	0.030422	0.942441	0.046738	5.255441	2.873304	38.512697	0.040551	0.92557
P-value	0.680516	0.027573	0.942093	0.050303	0.274745	0.280324	0.000809	0.019228	0.900591
95% Conf. Interval	0.948473	0.001361	0.190359	0.002176	0.267589	0.422868	4.548096	0.002771	0.275016

95% Conf. Interval	1.0351	0.6799	4.6659	1.0041	103.2168	19.5235	326.1206	0.5935	3.115
Multivariate	Age	ER.Status	PR.Status	HER2.Final.Status	Tumor Size	Lymph Node	Metastasis	PAM50 Subtypes	SETD6 Loss
Hazard Ratio	1.04457	0.68171	0.2602	0.46703	1.20512	2.11817	1.53253	0.68792	1.25411
P-value	0.00148	0.53563	0.00776	0.25385	0.66164	0.05107	0.4381	0.54018	0.59779
95% Conf. Interval	1.01686	0.20281	0.09658	0.1263	0.52256	0.99651	0.52091	0.20783	0.54078
	1.073	2.291	0.701	1.727	2.779	4.502	4.509	2.277	2.908
Multivariate	Age	ER.Status	PR.Status	HER2.Final.Status	Tumor Size	Lymph Node	Metastasis	PAM50 Subtypes	SETD7 Gain/Amp
Hazard Ratio	1.009088	0.757839	0.557851	0.916159	0.52173	2.271097	3.560902	1.36478	1.6622
P-value	0.583	0.738	0.393	0.915	0.19	0.089	0.113	0.669	0.401
95% Conf. Interval	0.977	0.1495	0.1461	0.182	0.197	0.8823	0.7398	0.3284	0.5077
	1.042	3.841	2.13	4.611	1.382	5.846	17.14	5.672	5.442
Multivariate	Age	ER.Status	PR.Status	HER2.Final.Status	Tumor Size	Lymph Node	Metastasis	PAM50 Subtypes	SETD7 Loss
Hazard Ratio	1.02954	1.004401	0.226261	0.555459	0.957915	1.524303	2.929361	0.586777	1.428348
P-value	0.03958	0.99424	0.00137	0.40013	0.92477	0.29533	0.04279	0.37098	0.31103
95% Conf. Interval	1.00139	0.30489	0.09106	0.1412	0.39241	0.69215	1.03553	0.18249	0.71661
	1.0585	3.3088	0.5622	2.1851	2.3384	3.3569	8.2868	1.8867	2.847
Multivariate	Age	ER.Status	PR.Status	HER2.Final.Status	Tumor Size	Lymph Node	Metastasis	PAM50 Subtypes	SETD8 Gain/Amp
Hazard Ratio	1.02573	0.89733	0.31502	1.2162	1.01074	0.97797	3.05789	0.86087	2.0676
P-value	0.107	0.8664	0.0184	0.7541	0.9823	0.9592	0.0545	0.8101	0.078
95% Conf. Interval	0.9945	0.2539	0.1205	0.3574	0.3944	0.4168	0.9788	0.2536	0.9217
	1.0579	3.1707	0.8234	4.1385	2.5902	2.2948	9.5531	2.9218	4.638
Multivariate	Age	ER.Status	PR.Status	HER2.Final.Status	Tumor Size	Lymph Node	Metastasis	PAM50 Subtypes	SETD8 Loss
Hazard Ratio	1.03355	1.1935	0.27268	0.84868	0.83218	2.8846	2.63678	0.6514	1.50631
P-value	0.0301	0.8039	0.0223	0.7936	0.6918	0.0135	0.097	0.5439	0.3133
95% Conf. Interval	1.00318	0.29537	0.08947	0.24827	0.33552	1.24483	0.83891	0.16318	0.67935
	1.0648	4.8226	0.8311	2.9011	2.064	6.6844	8.2877	2.6003	3.3399
Multivariate	Age	ER.Status	PR.Status	HER2.Final.Status	Tumor Size	Lymph Node	Metastasis	PAM50 Subtypes	SETDB1 Gain/Amp
Hazard Ratio	1.04329	0.63718	0.26705	0.79176	1.0325	2.0512	2.25263	0.76121	0.50121
P-value	0.0013	0.43788	0.00356	0.6963	0.93858	0.0498	0.1448	0.63338	0.075
95% Conf. Interval	1.0167	0.2041	0.1099	0.2451	0.4577	1.0006	0.7562	0.2481	0.2343
	1.0706	1.9897	0.6488	2.5574	2.3293	4.2048	6.7106	2.3357	1.0721
Multivariate	Age	ER.Status	PR.Status	HER2.Final.Status	Tumor Size	Lymph Node	Metastasis	PAM50 Subtypes	SMYD2 Gain/Amp
Hazard Ratio	1.04184	0.54456	0.38734	0.77774	0.9118	2.34032	2.3621	0.73768	0.52385
P-value	0.00231	0.34008	0.06201	0.68047	0.82703	0.02691	0.11053	0.61024	0.08933
95% Conf. Interval	1.0147	0.1562	0.1431	0.2351	0.3983	1.102	0.8219	0.229	0.2485
	1.07	1.898	1.049	2.573	2.087	4.97	6.789	2.376	1.104

Multivariate	Age	ER.Status	PR.Status	HER2.Final.Status	Tumor Size	Lymph Node	Metastasis	PAM50 Subtypes	SMYD3 Gain/Amp
Hazard Ratio	1.0416	0.43517	0.36277	0.60896	0.94199	2.75139	2.95237	0.67263	0.30789
P-value	0.00209	0.20049	0.03294	0.47381	0.88687	0.01054	0.03208	0.51886	0.00206
95% Conf. Interval	1.0149	0.1217	0.1429	0.1567	0.4135	1.2668	1.0971	0.2016	0.1455
	1.069	1.5556	0.9211	2.366	2.1458	5.9758	7.9452	2.244	0.6514

Multivariate	Age	ER.Status	PR.Status	HER2.Final.Status	Tumor Size	Lymph Node	Metastasis	PAM50 Subtypes	SMYD4 Loss
Hazard Ratio	1.03582	0.61153	0.35768	0.52289	0.96077	1.77827	1.98257	0.53825	1.68998
P-value	0.00855	0.41968	0.03219	0.28732	0.92527	0.12286	0.20261	0.28237	0.15132
95% Conf. Interval	1.009	0.1852	0.1396	0.1584	0.4163	0.8559	0.6919	0.174	0.8253
	1.0633	2.0191	0.9163	1.7263	2.2172	3.6947	5.6811	1.6652	3.4606

Multivariate	Age	ER.Status	PR.Status	HER2.Final.Status	Tumor Size	Lymph Node	Metastasis	PAM50 Subtypes	SMYD5 Gain/Amp
Hazard Ratio	1.03808	0.6913	0.26131	0.56102	1.28007	1.85042	1.98336	0.6273	0.72752
P-value	0.01021	0.51792	0.00421	0.34106	0.5762	0.10718	0.22184	0.41108	0.50699
95% Conf. Interval	1.0089	0.2257	0.1042	0.1707	0.5385	0.8752	0.6611	0.2063	0.2843
	1.068	2.117	0.655	1.844	3.043	3.912	5.95	1.907	1.862

Multivariate	Age	ER.Status	PR.Status	HER2.Final.Status	Tumor Size	Lymph Node	Metastasis	PAM50 Subtypes	SMYD5 Loss
Hazard Ratio	1.03106	0.78378	0.35969	0.74464	0.80972	2.10009	1.68534	0.94256	0.59809
P-value	0.0292	0.6954	0.0464	0.6565	0.6381	0.0649	0.3766	0.9215	0.3458
95% Conf. Interval	1.0031	0.2315	0.1315	0.203	0.3361	0.9552	0.5299	0.2909	0.2054
	1.0598	2.654	0.9836	2.7309	1.951	4.6175	5.3604	3.0544	1.7414

Multivariate	Age	ER.Status	PR.Status	HER2.Final.Status	Tumor Size	Lymph Node	Metastasis	PAM50 Subtypes	SUV39H1 Gain/Amp
Hazard Ratio	1.0285	0.94333	0.26353	0.41827	0.66487	1.85339	4.02466	0.78317	1.07791
P-value	0.0703	0.9337	0.0251	0.2733	0.3718	0.1402	0.0102	0.7066	0.8557
95% Conf. Interval	0.99768	0.23874	0.08203	0.08795	0.27149	0.81635	1.39171	0.21941	0.48019
	1.0603	3.7275	0.8466	1.9892	1.6283	4.2078	11.6389	2.7955	2.4197

Multivariate	Age	ER.Status	PR.Status	HER2.Final.Status	Tumor Size	Lymph Node	Metastasis	PAM50 Subtypes	SUV39H1 Loss
Hazard Ratio	1.02385	0.56666	0.33417	0.55494	1.21257	2.12112	1.83975	0.61349	1.28567
P-value	0.0918	0.3445	0.0278	0.3836	0.6954	0.0742	0.3195	0.3895	0.5345
95% Conf. Interval	0.9962	0.1745	0.1259	0.1475	0.462	0.929	0.554	0.2016	0.5818
	1.0523	1.8397	0.8872	2.0875	3.1826	4.843	6.1093	1.8667	2.8409

Multivariate	Age	ER.Status	PR.Status	HER2.Final.Status	Tumor Size	Lymph Node	Metastasis	PAM50 Subtypes	SUV39H2 Gain/Amp
Hazard Ratio	1.02514	1.11365	0.33048	0.928	0.8607	2.09544	2.76447	0.87308	1.3978
P-value	0.0886	0.8629	0.027	0.9024	0.7593	0.0684	0.0624	0.8352	0.4213
95% Conf. Interval	0.9963	0.3283	0.1239	0.2812	0.3296	0.9456	0.9485	0.2431	0.618
	1.0549	3.778	0.8817	3.0624	2.2473	4.6433	8.057	3.1356	3.1616

Multivariate	Age	ER.Status	PR.Status	HER2.Final.Status	Tumor Size	Lymph Node	Metastasis	PAM50 Subtypes	SUV39H2 Loss
Hazard Ratio	1.03188	1.51397	0.34131	0.76756	1.09114	1.56921	1.90281	1.1975	1.97371
P-value	0.0998	0.623	0.0702	0.7369	0.8693	0.3384	0.4373	0.8244	0.177
95% Conf. Interval	0.994	0.2897	0.1066	0.1641	0.3862	0.6238	0.3753	0.2438	0.7355
	1.071	7.912	1.093	3.591	3.083	3.948	9.647	5.882	5.296

Multivariate	Age	ER.Status	PR.Status	HER2.Final.Status	Tumor Size	Lymph Node	Metastasis	PAM50 Subtypes	SUV420H1 Gain/Amp
Hazard Ratio	1.04335	0.81656	0.36928	1.02714	0.89383	1.64068	2.64862	1.04419	1.23941
P-value	0.00307	0.74765	0.04592	0.96432	0.80194	0.20961	0.06325	0.94428	0.55138
95% Conf. Interval	1.0144	0.2376	0.1388	0.3178	0.3719	0.757	0.9477	0.3106	0.6117
	1.0731	2.8064	0.9821	3.3202	2.1485	3.5557	7.4027	3.5104	2.5114

Multivariate	Age	ER.Status	PR.Status	HER2.Final.Status	Tumor Size	Lymph Node	Metastasis	PAM50 Subtypes	SUV420H1 Loss
Hazard Ratio	1.05092	0.64071	0.16851	0.14059	1.18683	2.00755	2.22662	0.4061	1.68336
P-value	0.00362	0.51	0.00368	0.06942	0.73329	0.12176	0.31143	0.12674	0.27643
95% Conf. Interval	1.01634	0.17041	0.05068	0.01691	0.44312	0.83044	0.47261	0.12774	0.65898
	1.0867	2.4089	0.5604	1.1688	3.1788	4.8531	10.4903	1.291	4.3001

Multivariate	Age	ER.Status	PR.Status	HER2.Final.Status	Tumor Size	Lymph Node	Metastasis	PAM50 Subtypes	SUV420H2 Gain/Amp
Hazard Ratio	1.036388	1.268687	0.243693	0.970495	0.888301	1.855195	2.143728	0.83158	1.004049
P-value	0.0129	0.71649	0.00714	0.96076	0.77974	0.10431	0.22939	0.76969	0.99186
95% Conf. Interval	1.0076	0.35119	0.08712	0.2943	0.38729	0.88012	0.61821	0.24194	0.46191
	1.066	4.5832	0.6816	3.2004	2.0374	3.9106	7.4337	2.8582	2.1825

Multivariate	Age	ER.Status	PR.Status	HER2.Final.Status	Tumor Size	Lymph Node	Metastasis	PAM50 Subtypes	SUV420H2 Loss
Hazard Ratio	1.03182	0.59002	0.25559	0.5577	1.22308	1.60417	1.95831	0.45855	1.12029
P-value	0.0454	0.3866	0.0045	0.3993	0.7018	0.2888	0.2523	0.2669	0.8068
95% Conf. Interval	1.00065	0.17874	0.09972	0.14346	0.43623	0.66986	0.61972	0.11577	0.4509
	1.064	1.9477	0.6551	2.168	3.4292	3.8416	6.1883	1.8162	2.7834

Multivariate	Age	ER.Status	PR.Status	HER2.Final.Status	Tumor Size	Lymph Node	Metastasis	PAM50 Subtypes	WHSC1 Gain/Amp
Hazard Ratio	1.007946	0.980533	0.502311	1.414945	0.670273	2.030183	5.679511	1.301132	0.881347
P-value	0.636	0.983	0.352	0.675	0.445	0.153	0.025	0.741	0.866
95% Conf. Interval	0.9754	0.1679	0.1179	0.2797	0.2402	0.7687	1.2431	0.2739	0.2035
	1.042	5.725	2.139	7.159	1.87	5.362	25.949	6.181	3.817

Multivariate	Age	ER.Status	PR.Status	HER2.Final.Status	Tumor Size	Lymph Node	Metastasis	PAM50 Subtypes	WHSC1 Loss
Hazard Ratio	1.03233	0.93085	0.27358	0.69063	0.90546	1.87159	2.86633	0.74042	1.17057
P-value	0.01788	0.90369	0.00534	0.55	0.81834	0.10147	0.04699	0.62183	0.66142
95% Conf. Interval	1.0055	0.2916	0.1099	0.2052	0.388	0.884	1.0141	0.2243	0.5785
	1.0599	2.9715	0.6809	2.3245	2.113	3.9625	8.1014	2.444	2.3687

Multivariate	Age	ER.Status	PR.Status	HER2.Final.Status	Tumor Size	Lymph Node	Metastasis	PAM50 Subtypes	WHSC1L1 Gain/Amp

Hazard Ratio	1.02258	0.34223	0.37451	0.72677	0.72084	2.62661	1.87068	0.51415	1.59521
P-value	0.165	0.152	0.14	0.699	0.516	0.033	0.302	0.336	0.261
95% Conf. Interval	0.99087	0.07886	0.10156	0.14438	0.26827	1.08117	0.57008	0.13259	0.7071
	1.055	1.485	1.381	3.658	1.937	6.381	6.138	1.994	3.599
Multivariate	Age	ER.Status	PR.Status	HER2.Final.Status	Tumor Size	Lymph Node	Metastasis	PAM50 Subtypes	WHSC1L1 Loss
Hazard Ratio	1.05441	1.52317	0.15408	0.41597	1.07698	2.22996	2.0667	0.54308	1.94973
P-value	0.0052	0.57376	0.00173	0.21948	0.90021	0.10907	0.3662	0.4361	0.1386
95% Conf. Interval	1.01594	0.35156	0.04783	0.10257	0.3379	0.83614	0.428	0.11684	0.80579
	1.0943	6.5993	0.4964	1.6869	3.4327	5.9472	9.9796	2.5242	4.7176
Multivariate	Age	ER.Status	PR.Status	HER2.Final.Status	Tumor Size	Lymph Node	Metastasis	PAM50 Subtypes	SETD2 Gain/Amp
Hazard Ratio	1.02956	1.90649	0.3228	1.05882	0.98593	1.94972	2.76899	1.14656	1.01625
P-value	0.132	0.511	0.173	0.943	0.978	0.168	0.15	0.874	0.976
95% Conf. Interval	0.99125	0.27789	0.06354	0.22401	0.35269	0.75489	0.69243	0.21098	0.35148
	1.069	13.079	1.64	5.005	2.756	5.036	11.073	6.231	2.938
Multivariate	Age	ER.Status	PR.Status	HER2.Final.Status	Tumor Size	Lymph Node	Metastasis	PAM50 Subtypes	SETD2 Loss
Hazard Ratio	1.03035	0.62666	0.32378	0.47803	0.84961	1.61408	1.92798	0.62512	1.54876
P-value	0.0263	0.4216	0.0219	0.2734	0.7227	0.2132	0.2761	0.4086	0.2422
95% Conf. Interval	1.0035	0.2005	0.1235	0.1276	0.3454	0.7595	0.5916	0.2051	0.744
	1.0579	1.959	0.8491	1.7909	2.0898	3.4303	6.2835	1.9052	3.2239
Multivariate	Age	ER.Status	PR.Status	HER2.Final.Status	Tumor Size	Lymph Node	Metastasis	PAM50 Subtypes	SETDB2 Gain/Amp
Hazard Ratio	1.01758	1.302	0.26179	0.50743	0.61582	1.83172	6.43834	1.43865	1.53409
P-value	0.4233	0.7914	0.0744	0.565	0.4424	0.2863	0.0112	0.705	0.4725
95% Conf. Interval	0.97508	0.18425	0.06005	0.05033	0.17873	0.60209	1.5282	0.2189	0.47737
	1.062	9.201	1.141	5.116	2.122	5.573	27.125	9.455	4.93
Multivariate	Age	ER.Status	PR.Status	HER2.Final.Status	Tumor Size	Lymph Node	Metastasis	PAM50 Subtypes	SETDB2 Loss
Hazard Ratio	1.03841	0.68049	0.36045	0.66314	0.86325	2.37642	1.37239	0.44937	2.28877
P-value	0.0088	0.5573	0.0572	0.4935	0.743	0.0301	0.5728	0.2113	0.0289
95% Conf. Interval	1.0095	0.1881	0.1259	0.2047	0.3584	1.0868	0.4568	0.1282	1.0887
	1.068	2.462	1.032	2.149	2.079	5.197	4.123	1.575	4.812
Multivariate	Age	ER.Status	PR.Status	HER2.Final.Status	Tumor Size	Lymph Node	Metastasis	PAM50 Subtypes	SETMAR Gain/Amp
Hazard Ratio	1.0316	0.71382	0.21671	0.39901	1.01729	2.24926	1.70978	0.33996	1.95611
P-value	0.029	0.60013	0.00326	0.17326	0.96908	0.04111	0.38311	0.06752	0.05632
95% Conf. Interval	1.00319	0.20238	0.07825	0.10634	0.42751	1.03327	0.51225	0.10693	0.9821
	1.0608	2.5177	0.6002	1.4972	2.4207	4.8963	5.7069	1.0809	3.8961
Multivariate	Age	ER.Status	PR.Status	HER2.Final.Status	Tumor Size	Lymph Node	Metastasis	PAM50 Subtypes	SETMAR Loss
Hazard Ratio	1.02582	0.9768	0.43743	0.84802	0.60038	1.78191	3.11971	1.76921	1.19215

P-value	0.1203	0.9725	0.1637	0.8389	0.3176	0.2275	0.0822	0.3797	0.7082
95% Conf. Interval	0.9934	0.2567	0.1366	0.173	0.2208	0.6973	0.8648	0.4953	0.4749
	1.059	3.716	1.4	4.156	1.633	4.553	11.254	6.319	2.993
Multivariate	Age	ER.Status	PR.Status	HER2.Final.Status	Tumor Size	Lymph Node	Metastasis	PAM50 Subtypes	KMT2C Gain/Amp
Hazard Ratio	1.02008	1.27265	0.18113	0.30375	1.24799	1.82009	2.37173	0.47617	0.62403
P-value	0.1886	0.7531	0.0028	0.256	0.6613	0.1776	0.0988	0.3066	0.2526
95% Conf. Interval	0.99029	0.28338	0.05908	0.03887	0.46331	0.76199	0.85056	0.11482	0.27818
	1.0508	5.7155	0.5553	2.3739	3.3616	4.3475	6.6134	1.9748	1.3998
Multivariate	Age	ER.Status	PR.Status	HER2.Final.Status	Tumor Size	Lymph Node	Metastasis	PAM50 Subtypes	KMT2C Loss
Hazard Ratio	1.03532	0.45656	0.42146	0.45507	1.97965	1.37676	1.53781	0.60614	1.74748
P-value	0.0235	0.2327	0.1234	0.2053	0.1885	0.4246	0.519	0.3996	0.2141
95% Conf. Interval	1.0047	0.126	0.1404	0.1346	0.7155	0.6281	0.4157	0.1891	0.7244
	1.067	1.655	1.265	1.539	5.478	3.018	5.689	1.943	4.215
Multivariate	Age	ER.Status	PR.Status	HER2.Final.Status	Tumor Size	Lymph Node	Metastasis	PAM50 Subtypes	PRDM1 Gain/Amp
Hazard Ratio	1.04351	0.9476	0.18439	0.35461	1.2185	2.34455	1.77212	0.4584	2.80259
P-value	0.00895	0.93658	0.00545	0.14413	0.68312	0.03872	0.38586	0.27329	0.02251
95% Conf. Interval	1.01071	0.25165	0.05597	0.08822	0.47179	1.04519	0.48623	0.11355	1.15637
	1.0774	3.5681	0.6075	1.4254	3.147	5.2593	6.4587	1.8505	6.7924
Multivariate	Age	ER.Status	PR.Status	HER2.Final.Status	Tumor Size	Lymph Node	Metastasis	PAM50 Subtypes	PRDM1 Loss
Hazard Ratio	1.02236	0.46091	0.33148	0.25744	0.75207	2.72418	2.38984	0.39529	1.23125
P-value	0.138	0.2704	0.036	0.2026	0.5791	0.0409	0.1143	0.1552	0.5882
95% Conf. Interval	0.99292	0.11625	0.11809	0.03193	0.27478	1.04231	0.81056	0.10992	0.57989
	1.0527	1.8275	0.9305	2.0756	2.0584	7.1199	7.0462	1.4215	2.6143
Multivariate	Age	ER.Status	PR.Status	HER2.Final.Status	Tumor Size	Lymph Node	Metastasis	PAM50 Subtypes	PRDM5 Gain/Amp
Hazard Ratio	1.01172	0.77119	0.53976	0.90819	0.56109	2.13624	3.36226	1.29628	1.57769
P-value	0.472	0.752	0.365	0.906	0.235	0.101	0.128	0.72	0.445
95% Conf. Interval	0.9801	0.1535	0.1423	0.1822	0.2163	0.8628	0.7052	0.3143	0.4896
	1.044	3.875	2.047	4.527	1.455	5.289	16.03	5.347	5.084
Multivariate	Age	ER.Status	PR.Status	HER2.Final.Status	Tumor Size	Lymph Node	Metastasis	PAM50 Subtypes	PRDM5 Loss
Hazard Ratio	1.029431	0.983359	0.230642	0.545071	0.994696	1.47941	2.924637	0.581298	1.404228
P-value	0.03984	0.97791	0.00157	0.38472	0.99061	0.33669	0.04348	0.36381	0.34444
95% Conf. Interval	1.00135	0.29976	0.09287	0.13874	0.41018	0.66542	1.03193	0.18027	0.69468
	1.0583	3.2259	0.5728	2.1414	2.4122	3.2892	8.2888	1.8745	2.8385
Multivariate	Age	ER.Status	PR.Status	HER2.Final.Status	Tumor Size	Lymph Node	Metastasis	PAM50 Subtypes	PRDM8 Gain/Amp
Hazard Ratio	1.01202	1.48615	0.32535	1.57386	0.57354	1.86919	2.56815	1.0867	2.28539
P-value	0.459	0.636	0.108	0.521	0.245	0.175	0.221	0.903	0.129

95% Conf. Interval	0.98053<br>1.045	0.28814<br>7.665	0.08262<br>1.281	0.39452<br>6.279	0.22467<br>1.464	0.75684<br>4.616	0.56712<br>11.63	0.28664<br>4.12	0.78513<br>6.652
Multivariate	Age	ER.Status	PR.Status	HER2.Final.Status	Tumor Size	Lymph Node	Metastasis	PAM50 Subtypes	PRDM8 Loss
Hazard Ratio	1.02608	0.71171	0.2334	0.29653	1.16929	1.5079	3.00128	0.47882	1.36177
P-value	0.07589	0.58753	0.00193	0.13793	0.74748	0.32468	0.04042	0.2439	0.40155
95% Conf. Interval	0.99732<br>1.0557	0.20826<br>2.4322	0.09305<br>0.5854	0.05951<br>1.4775	0.45127<br>3.0297	0.66589<br>3.4146	1.04914<br>8.5857	0.13876<br>1.6524	0.66187<br>2.8018
