# Supplementary material for: Genetic alterations of histone lysine methyltransferases and their significance in breast cancer
Source: Oncotarget. 2014 Dec 11;6(4):2466–82. doi: 10.18632/oncotarget.2967 (PMC4385864; doi:10.18632/oncotarget.2967)
Supplement: Supplementary file 9 [file oncotarget-06-2466-s009.pdf]

Table S8. Summary of multivariate analysis of overall survival for 44 HMT expressions in breast cancer

Multivariate	Age	ER.Status	PR.Status	HER2.Final.Status	Tumor Size	Lymph Node	Metastasis	PAM50 subtype	ASH1L
Hazard Ratio	1.03312	0.78622	0.27763	0.66047	1.05469	1.97754	2.40323	0.649	1.24159
P-value	0.0134	0.68114	0.00555	0.48917	0.89936	0.06324	0.08571	0.43596	0.50979
95% Conf. Interval	1.0068	0.2496	0.1122	0.2039	0.4621	0.9631	0.8841	0.2187	0.6524
	1.0601	2.4761	0.6868	2.1397	2.4072	4.0606	6.5328	1.9258	2.3627
Multivariate	Age	ER.Status	PR.Status	HER2.Final.Status	Tumor Size	Lymph Node	Metastasis	PAM50 subtype	DOT1L
Hazard Ratio	1.03424	0.72125	0.28643	0.60624	1.02259	1.97228	2.09738	0.57747	1.22744
P-value	0.0091	0.57174	0.00615	0.40176	0.95751	0.06371	0.14755	0.34203	0.52766
95% Conf. Interval	1.0084	0.2324	0.1171	0.1882	0.4496	0.962	0.7697	0.186	0.6498
	1.0607	2.2384	0.7006	1.9531	2.3258	4.0435	5.715	1.7924	2.3184
Multivariate	Age	ER.Status	PR.Status	HER2.Final.Status	Tumor Size	Lymph Node	Metastasis	PAM50 subtype	EHMT1
Hazard Ratio	1.03447	0.73795	0.27732	0.59332	1.05606	1.9341	2.39796	0.57951	0.77567
P-value	0.00904	0.59685	0.00528	0.38002	0.89574	0.07005	0.08528	0.33412	0.43775
95% Conf. Interval	1.0085	0.2393	0.1126	0.185	0.4671	0.9474	0.8856	0.1915	0.4084
	1.061	2.275	0.683	1.903	2.388	3.948	6.493	1.753	1.473
Multivariate	Age	ER.Status	PR.Status	HER2.Final.Status	Tumor Size	Lymph Node	Metastasis	PAM50 subtype	EHMT2
Hazard Ratio	1.02961	0.80379	0.32213	0.637	1.05657	1.94417	2.48091	0.90525	0.53692
P-value	0.0248	0.7049	0.0145	0.4432	0.8963	0.0681	0.0777	0.8647	0.0669
95% Conf. Interval	1.0037	0.2595	0.1299	0.2011	0.4618	0.9518	0.9041	0.288	0.2761
	1.0562	2.4895	0.7986	2.0175	2.4173	3.9711	6.8079	2.8455	1.0443
Multivariate	Age	ER.Status	PR.Status	HER2.Final.Status	Tumor Size	Lymph Node	Metastasis	PAM50 subtype	EZH1
Hazard Ratio	1.03381	0.77341	0.27574	0.67881	1.08555	1.96477	2.44798	0.68447	1.26215
P-value	0.01024	0.65626	0.00546	0.52248	0.84568	0.06371	0.08298	0.49864	0.49599
95% Conf. Interval	1.0079	0.2495	0.1111	0.2071	0.4749	0.9622	0.8897	0.2283	0.6457
	1.0604	2.3977	0.6841	2.2253	2.4812	4.0119	6.7355	2.0525	2.4671
Multivariate	Age	ER.Status	PR.Status	HER2.Final.Status	Tumor Size	Lymph Node	Metastasis	PAM50 subtype	EZH2
Hazard Ratio	1.03479	0.70643	0.29994	0.60561	0.97764	1.99142	2.29769	0.55517	1.23871
P-value	0.00811	0.54744	0.00855	0.39732	0.95846	0.06077	0.10048	0.32466	0.56025
95% Conf. Interval	1.0089	0.2277	0.1222	0.1896	0.4174	0.9693	0.8515	0.1721	0.6027
	1.0613	2.1918	0.7359	1.9344	2.2897	4.0912	6.2001	1.7906	2.5457
Multivariate	Age	ER.Status	PR.Status	HER2.Final.Status	Tumor Size	Lymph Node	Metastasis	PAM50 subtype	KMT2A

Hazard Ratio	1.03231	0.73562	0.25045	0.61364	1.14649	1.83413	2.69995	0.47017	1.83856
P-value	0.01332	0.5927	0.00303	0.41008	0.74144	0.09509	0.05038	0.19681	0.07379
95% Conf. Interval	1.0066	0.2388	0.1003	0.192	0.509	0.8997	0.9984	0.1495	0.9431
	1.0586	2.2659	0.6256	1.9612	2.5825	3.7391	7.3016	1.4789	3.5843
Multivariate	Age	ER.Status	PR.Status	HER2.Final.Status	Tumor Size	Lymph Node	Metastasis	PAM50 subtype	KMT2C
Hazard Ratio	1.03377	0.71549	0.29039	0.62761	1.03229	1.96467	2.29374	0.62398	1.12489
P-value	0.0116	0.5611	0.0066	0.4342	0.9394	0.064	0.1006	0.3994	0.714
95% Conf. Interval	1.0074	0.2313	0.119	0.1953	0.4549	0.9614	0.8515	0.2084	0.5995
	1.0608	2.2129	0.7087	2.0171	2.3425	4.015	6.1784	1.8687	2.1106
Multivariate	Age	ER.Status	PR.Status	HER2.Final.Status	Tumor Size	Lymph Node	Metastasis	PAM50 subtype	KMT2E
Hazard Ratio	1.03526	0.71155	0.29585	0.60993	1.03833	1.9777	2.23328	0.63247	0.96189
P-value	0.00874	0.56432	0.00845	0.40891	0.92866	0.06237	0.10886	0.41074	0.90414
95% Conf. Interval	1.0088	0.2237	0.1195	0.1887	0.4557	0.9654	0.8363	0.2123	0.5111
	1.0624	2.2632	0.7324	1.9719	2.3657	4.0516	5.9635	1.8841	1.8102
Multivariate	Age	ER.Status	PR.Status	HER2.Final.Status	Tumor Size	Lymph Node	Metastasis	PAM50 subtype	MECOM
Hazard Ratio	1.03488	0.7208	0.29233	0.61498	1.05112	1.95152	2.286	0.63487	1.03761
P-value	0.0078	0.56891	0.00667	0.41275	0.90656	0.07949	0.12189	0.41303	0.91366
95% Conf. Interval	1.0091	0.2337	0.1202	0.1921	0.4572	0.9244	0.8019	0.2139	0.5323
	1.0614	2.2233	0.7108	1.9684	2.4167	4.1201	6.5168	1.8842	2.0224
Multivariate	Age	ER.Status	PR.Status	HER2.Final.Status	Tumor Size	Lymph Node	Metastasis	PAM50 subtype	NSD1
Hazard Ratio	1.03456	0.7132	0.28359	0.60992	1.08397	1.88511	2.41431	0.58647	1.42249
P-value	0.00803	0.55786	0.00542	0.40611	0.84579	0.08418	0.07798	0.34433	0.25676
95% Conf. Interval	1.0089	0.2303	0.1167	0.19	0.481	0.918	0.906	0.1941	0.7737
	1.0609	2.2088	0.6893	1.9582	2.4429	3.871	6.4336	1.7725	2.6155
Multivariate	Age	ER.Status	PR.Status	HER2.Final.Status	Tumor Size	Lymph Node	Metastasis	PAM50 subtype	PRDM1
Hazard Ratio	1.03392	0.69275	0.30307	0.57698	1.06583	1.84695	2.32276	0.59784	1.31466
P-value	0.0092	0.52743	0.00855	0.36233	0.87877	0.10043	0.09576	0.36128	0.39341
95% Conf. Interval	1.0083	0.2219	0.1245	0.1767	0.4698	0.8883	0.8617	0.1981	0.7014
	1.0602	2.1629	0.7378	1.8839	2.4181	3.8403	6.2611	1.804	2.4641
Multivariate	Age	ER.Status	PR.Status	HER2.Final.Status	Tumor Size	Lymph Node	Metastasis	PAM50 subtype	PRDM2
Hazard Ratio	1.03307	0.73456	0.26543	0.59325	1.06027	1.88116	2.60765	0.59792	1.50843
P-value	0.01107	0.58861	0.00428	0.38283	0.8877	0.0834	0.06118	0.35887	0.19939

95% Conf. Interval	1.0075	0.2402	0.1068	0.1836	0.4706	0.9199	0.9561	0.1993	0.8051
	1.0593	2.2466	0.6594	1.9166	2.3886	3.8468	7.1124	1.7938	2.8261

Multivariate	Age	ER.Status	PR.Status	HER2.Final.Status	Tumor Size	Lymph Node	Metastasis	PAM50 subtype	PRDM4
Hazard Ratio	1.03616	0.62108	0.29302	0.67241	1.01741	1.87063	2.57537	0.65512	1.57659
P-value	0.00557	0.4113	0.00657	0.50193	0.96751	0.09375	0.06128	0.44182	0.17048
95% Conf. Interval	1.0105	0.1994	0.1209	0.2111	0.4434	0.8993	0.9562	0.223	0.8222
	1.0625	1.9346	0.7102	2.1417	2.3345	3.8911	6.936	1.9248	3.0231

Multivariate	Age	ER.Status	PR.Status	HER2.Final.Status	Tumor Size	Lymph Node	Metastasis	PAM50 subtype	PRDM5
Hazard Ratio	1.036156	0.684609	0.278098	0.570637	1.009437	2.056683	1.829439	0.553325	0.734028
P-value	0.00617	0.51004	0.00542	0.35039	0.98208	0.04972	0.27035	0.30161	0.36729
95% Conf. Interval	1.0101	0.2217	0.1128	0.1758	0.4449	1.0009	0.625	0.18	0.3748
	1.0628	2.1136	0.6854	1.8524	2.2906	4.2262	5.3549	1.7008	1.4376

Multivariate	Age	ER.Status	PR.Status	HER2.Final.Status	Tumor Size	Lymph Node	Metastasis	PAM50 subtype	PRDM6
Hazard Ratio	1.03538	0.63347	0.33116	0.63638	0.98265	2.06082	2.42107	0.55409	0.75746
P-value	0.00728	0.45296	0.02189	0.4477	0.96687	0.05002	0.08714	0.3161	0.45844
95% Conf. Interval	1.0094	0.1923	0.1287	0.1982	0.4303	0.9999	0.8791	0.1747	0.3634
	1.062	2.087	0.852	2.044	2.244	4.247	6.668	1.758	1.579

Multivariate	Age	ER.Status	PR.Status	HER2.Final.Status	Tumor Size	Lymph Node	Metastasis	PAM50 subtype	PRDM8
Hazard Ratio	1.03448	0.71994	0.29426	0.61111	1.06122	1.92261	2.30104	0.62131	1.11873
P-value	0.00855	0.56903	0.00711	0.40771	0.88754	0.0798	0.10074	0.39479	0.7239
95% Conf. Interval	1.0087	0.2324	0.1207	0.1905	0.4658	0.9253	0.8506	0.2076	0.6003
	1.061	2.2307	0.7172	1.9609	2.418	3.9949	6.2249	1.8594	2.0848

Multivariate	Age	ER.Status	PR.Status	HER2.Final.Status	Tumor Size	Lymph Node	Metastasis	PAM50 subtype	PRDM10
Hazard Ratio	1.03326	0.74197	0.29111	0.62292	1.07644	1.95907	2.40085	0.64448	1.1889
P-value	0.01384	0.60204	0.00658	0.42508	0.86255	0.06755	0.0889	0.42914	0.59274
95% Conf. Interval	1.0067	0.2417	0.1195	0.1947	0.4675	0.9526	0.8753	0.2169	0.6306
	1.061	2.278	0.709	1.993	2.478	4.029	6.585	1.915	2.241

Multivariate	Age	ER.Status	PR.Status	HER2.Final.Status	Tumor Size	Lymph Node	Metastasis	PAM50 subtype	PRDM11
Hazard Ratio	1.03558	0.69692	0.28703	0.63215	1.07326	1.97137	2.24538	0.62277	1.1806
P-value	0.00684	0.53334	0.00589	0.44298	0.86604	0.06274	0.10359	0.39773	0.59815
95% Conf. Interval	1.0097	0.2238	0.1181	0.1959	0.472	0.9646	0.8478	0.2078	0.6368
	1.0622	2.1706	0.6978	2.0403	2.4402	4.029	5.9469	1.8663	2.1889

Multivariate	Age	ER.Status	PR.Status	HER2.Final.Status	Tumor Size	Lymph Node	Metastasis	PAM50 subtype	PRDM12
Hazard Ratio	1.03381	0.7773	0.28203	0.63387	1.0581	1.93298	2.29638	0.64692	0.86176
P-value	0.01095	0.67249	0.00607	0.44135	0.89288	0.07241	0.09692	0.43465	0.64467
95% Conf. Interval	1.0077	0.2417	0.1142	0.1986	0.4651	0.9418	0.8605	0.2169	0.4579
	1.0606	2.4995	0.6965	2.0233	2.4073	3.9673	6.1281	1.9292	1.6218

Multivariate	Age	ER.Status	PR.Status	HER2.Final.Status	Tumor Size	Lymph Node	Metastasis	PAM50 subtype	PRDM15
Hazard Ratio	1.03277	0.69458	0.29628	0.58875	1.03184	1.93594	2.2716	0.5329	1.34292
P-value	0.01361	0.53163	0.00775	0.3794	0.94014	0.07181	0.10321	0.2914	0.37288
95% Conf. Interval	1.0067	0.2217	0.121	0.1807	0.4553	0.9431	0.8467	0.1655	0.7021
	1.0596	2.176	0.7253	1.9184	2.3383	3.9739	6.0943	1.7157	2.5686

Multivariate	Age	ER.Status	PR.Status	HER2.Final.Status	Tumor Size	Lymph Node	Metastasis	PAM50 subtype	PRDM16
Hazard Ratio	1.03524	0.64617	0.31008	0.61465	1.15264	1.77655	3.17237	0.66602	1.7311
P-value	0.00598	0.45423	0.00948	0.41799	0.73216	0.11737	0.03512	0.46655	0.10657
95% Conf. Interval	1.01	0.2059	0.128	0.1893	0.511	0.8654	1.0839	0.223	0.889
	1.061	2.028	0.751	1.996	2.6	3.647	9.285	1.989	3.371

Multivariate	Age	ER.Status	PR.Status	HER2.Final.Status	Tumor Size	Lymph Node	Metastasis	PAM50 subtype	SETD1A
Hazard Ratio	1.03529	0.70405	0.29874	0.58825	1.03357	1.93902	2.34615	0.65021	0.85659
P-value	0.00733	0.54455	0.00778	0.37807	0.93674	0.06961	0.09315	0.44234	0.63044
95% Conf. Interval	1.0094	0.2263	0.1227	0.1808	0.4573	0.9483	0.867	0.2168	0.4559
	1.0619	2.1905	0.7273	1.914	2.336	3.9648	6.3486	1.9498	1.6094

Multivariate	Age	ER.Status	PR.Status	HER2.Final.Status	Tumor Size	Lymph Node	Metastasis	PAM50 subtype	SETD1B
Hazard Ratio	1.03499	0.72101	0.29066	0.61326	1.04403	1.97495	2.22687	0.63	1.02654
P-value	0.0079	0.56893	0.00734	0.41169	0.91802	0.0626	0.11244	0.41264	0.93324
95% Conf. Interval	1.0091	0.234	0.1178	0.1908	0.4596	0.9648	0.8287	0.2086	0.5562
	1.0616	2.222	0.7173	1.9708	2.3715	4.0426	5.9843	1.9028	1.8947

Multivariate	Age	ER.Status	PR.Status	HER2.Final.Status	Tumor Size	Lymph Node	Metastasis	PAM50 subtype	SETD2
Hazard Ratio	1.034	0.73194	0.27855	0.63307	1.05499	1.94892	2.36644	0.61944	1.21735
P-value	0.0096	0.58897	0.00574	0.44226	0.89773	0.06772	0.08862	0.39113	0.53537
95% Conf. Interval	1.0082	0.236	0.1125	0.1972	0.4663	0.9526	0.878	0.2073	0.6536
	1.0605	2.2702	0.6899	2.0319	2.3868	3.9875	6.3784	1.8509	2.2673

Multivariate	Age	ER.Status	PR.Status	HER2.Final.Status	Tumor Size	Lymph Node	Metastasis	PAM50 subtype	SETD3

Hazard Ratio	1.03339	0.66817	0.27517	0.58532	0.96488	1.92439	3.16371	0.46524	0.54199
P-value	0.00967	0.48585	0.00448	0.36975	0.932	0.07437	0.03351	0.1909	0.08938
95% Conf. Interval	1.008	0.215	0.113	0.1816	0.4245	0.9376	1.0941	0.1478	0.2673
	1.059	2.077	0.67	1.887	2.193	3.95	9.149	1.464	1.099
Multivariate	Age	ER.Status	PR.Status	HER2.Final.Status	Tumor Size	Lymph Node	Metastasis	PAM50 subtype	SETD4
Hazard Ratio	1.03842	0.59258	0.28807	0.61794	0.98911	1.9497	2.82474	0.56827	1.92037
P-value	0.0038	0.3753	0.00562	0.43005	0.97892	0.06857	0.04188	0.32114	0.04678
95% Conf. Interval	1.0122	0.1864	0.1194	0.1869	0.439	0.9504	1.0389	0.1861	1.0093
	1.0653	1.8842	0.6951	2.0426	2.2287	3.9996	7.6806	1.7356	3.6537
Multivariate	Age	ER.Status	PR.Status	HER2.Final.Status	Tumor Size	Lymph Node	Metastasis	PAM50 subtype	SETD5
Hazard Ratio	1.03739	0.65522	0.27415	0.62724	0.96749	1.99102	3.16256	0.43043	2.79394
P-value	0.00503	0.46337	0.00531	0.43108	0.9361	0.0602	0.02699	0.13588	0.00231
95% Conf. Interval	1.0111	0.2117	0.1104	0.1964	0.4313	0.9709	1.14	0.1422	1.4426
	1.064	2.028	0.681	2.003	2.17	4.083	8.773	1.303	5.411
Multivariate	Age	ER.Status	PR.Status	HER2.Final.Status	Tumor Size	Lymph Node	Metastasis	PAM50 subtype	SETD6
Hazard Ratio	1.03471	0.69742	0.30184	0.5947	1.03456	1.93859	2.29877	0.60467	1.09825
P-value	0.00831	0.54205	0.01054	0.39348	0.93547	0.07491	0.1032	0.39097	0.79163
95% Conf. Interval	1.0088	0.219	0.1205	0.1803	0.4545	0.9357	0.8447	0.1916	0.548
	1.0613	2.2212	0.7559	1.962	2.3548	4.0166	6.2559	1.9085	2.2011
Multivariate	Age	ER.Status	PR.Status	HER2.Final.Status	Tumor Size	Lymph Node	Metastasis	PAM50 subtype	SETD7
Hazard Ratio	1.03399	0.72015	0.25709	0.55166	1.06859	1.8992	2.55723	0.74277	1.97415
P-value	0.01036	0.5686	0.00352	0.31883	0.87367	0.07903	0.06084	0.60127	0.03906
95% Conf. Interval	1.0079	0.233	0.1032	0.1713	0.4717	0.9283	0.9582	0.2435	1.0347
	1.0608	2.2263	0.6402	1.7766	2.4207	3.8854	6.8248	2.2658	3.7665
Multivariate	Age	ER.Status	PR.Status	HER2.Final.Status	Tumor Size	Lymph Node	Metastasis	PAM50 subtype	SETD8
Hazard Ratio	1.03474	0.72088	0.29751	0.63049	1.11493	1.97425	2.37566	0.68819	0.83129
P-value	0.00805	0.5662	0.00737	0.43737	0.80216	0.06188	0.08942	0.51331	0.58901
95% Conf. Interval	1.0089	0.2356	0.1226	0.1969	0.476	0.9668	0.8753	0.2244	0.4253
	1.0612	2.2054	0.7221	2.0194	2.6113	4.0317	6.4481	2.1101	1.625
Multivariate	Age	ER.Status	PR.Status	HER2.Final.Status	Tumor Size	Lymph Node	Metastasis	PAM50 subtype	SETDB1
Hazard Ratio	1.03518	0.58339	0.34842	0.55509	1.06781	1.99686	2.51067	0.7617	0.59245
P-value	0.00715	0.38071	0.02404	0.332	0.87294	0.05571	0.0724	0.64134	0.12394

95% Conf. Interval	1.0094	0.1748	0.1394	0.169	0.4778	0.9833	0.9197	0.2423	0.3041
	1.0616	1.9465	0.8706	1.8232	2.3862	4.0552	6.8537	2.3943	1.1542
Multivariate	Age	ER.Status	PR.Status	HER2.Final.Status	Tumor Size	Lymph Node	Metastasis	PAM50 subtype	SETDB2
Hazard Ratio	1.03476	0.72369	0.29267	0.61927	1.04748	1.97475	2.24506	0.63829	1.01694
P-value	0.00974	0.57324	0.00678	0.42846	0.91363	0.06262	0.10748	0.425	0.96027
95% Conf. Interval	1.0083	0.2349	0.1203	0.1891	0.453	0.9648	0.8386	0.2118	0.5252
	1.0619	2.2297	0.7123	2.0278	2.4221	4.042	6.0105	1.9233	1.9692
Multivariate	Age	ER.Status	PR.Status	HER2.Final.Status	Tumor Size	Lymph Node	Metastasis	PAM50 subtype	SETMAR
Hazard Ratio	1.03455	0.70356	0.28822	0.61107	1.04512	2.00053	2.19933	0.60479	0.86526
P-value	0.00843	0.54189	0.00624	0.40676	0.91562	0.05749	0.11463	0.37563	0.65435
95% Conf. Interval	1.0087	0.2273	0.1182	0.1909	0.462	0.9782	0.8262	0.1988	0.4592
	1.061	2.1776	0.7029	1.9564	2.3644	4.0913	5.8547	1.8397	1.6304
Multivariate	Age	ER.Status	PR.Status	HER2.Final.Status	Tumor Size	Lymph Node	Metastasis	PAM50 subtype	SMYD2
Hazard Ratio	1.03508	0.73065	0.29163	0.63089	1.0386	2.01264	2.2253	0.66332	0.93079
P-value	0.00784	0.58501	0.00651	0.44604	0.92808	0.06378	0.1095	0.48857	0.83545
95% Conf. Interval	1.0091	0.2369	0.12	0.1929	0.4563	0.9607	0.8355	0.2076	0.4731
	1.0617	2.2536	0.7085	2.0629	2.3639	4.2164	5.9267	2.1195	1.8313
Multivariate	Age	ER.Status	PR.Status	HER2.Final.Status	Tumor Size	Lymph Node	Metastasis	PAM50 subtype	SMYD3
Hazard Ratio	1.03943	0.43227	0.47171	0.66747	0.93735	2.1065	2.7126	0.46508	0.35402
P-value	0.00262	0.17688	0.11865	0.5016	0.87396	0.03873	0.05604	0.18983	0.00414
95% Conf. Interval	1.0136	0.128	0.1836	0.2053	0.4214	1.0393	0.9746	0.1481	0.1741
	1.0659	1.4602	1.2121	2.1703	2.0848	4.2694	7.55	1.4606	0.7199
Multivariate	Age	ER.Status	PR.Status	HER2.Final.Status	Tumor Size	Lymph Node	Metastasis	PAM50 subtype	SMYD4
Hazard Ratio	1.03499	0.70455	0.2906	0.64794	1.0416	1.9181	2.39029	0.60107	1.21052
P-value	0.00735	0.54537	0.00647	0.46983	0.92225	0.07715	0.08817	0.37041	0.55586
95% Conf. Interval	1.0093	0.2264	0.1194	0.1997	0.4594	0.9315	0.8779	0.1973	0.641
	1.0613	2.1921	0.7074	2.1019	2.3614	3.9496	6.5082	1.8309	2.286
Multivariate	Age	ER.Status	PR.Status	HER2.Final.Status	Tumor Size	Lymph Node	Metastasis	PAM50 subtype	SMYD5
Hazard Ratio	1.03401	0.70958	0.28839	0.62627	1.02804	1.97568	2.39303	0.6296	0.85649
P-value	0.0097	0.55216	0.00603	0.42995	0.94732	0.0621	0.09158	0.40657	0.62866
95% Conf. Interval	1.0081	0.229	0.1187	0.1959	0.4527	0.9661	0.8684	0.2111	0.4572
	1.0605	2.1989	0.7005	2.0019	2.3348	4.0401	6.5945	1.8776	1.6047

Multivariate	Age	ER.Status	PR.Status	HER2.Final.Status	Tumor Size	Lymph Node	Metastasis	PAM50 subtype	SUV39H1
Hazard Ratio	1.03552	0.71533	0.29633	0.52734	0.95104	1.95833	1.8602	0.46658	1.74412
P-value	0.0067	0.56207	0.00802	0.28591	0.90502	0.06748	0.23231	0.19102	0.11933
95% Conf. Interval	1.0097	0.2305	0.1206	0.1628	0.4169	0.9528	0.6718	0.1488	0.8661
	1.062	2.22	0.7283	1.7081	2.1693	4.025	5.151	1.4628	3.5121

Multivariate	Age	ER.Status	PR.Status	HER2.Final.Status	Tumor Size	Lymph Node	Metastasis	PAM50 subtype	SUV39H2
Hazard Ratio	1.03649	0.6787	0.31061	0.50416	1.01087	2.00185	1.83857	0.45387	1.75061
P-value	0.00611	0.50602	0.01123	0.26523	0.97952	0.06195	0.24266	0.1828	0.12123
95% Conf. Interval	1.0103	0.2166	0.1258	0.1511	0.4429	0.9659	0.6619	0.142	0.8622
	1.0634	2.1268	0.7669	1.682	2.3074	4.1489	5.1068	1.451	3.5545

Multivariate	Age	ER.Status	PR.Status	HER2.Final.Status	Tumor Size	Lymph Node	Metastasis	PAM50 subtype	SUV420H1
Hazard Ratio	1.03479	0.71832	0.29465	0.63163	1.04829	1.96648	2.25561	0.64003	1.08993
P-value	0.00809	0.56261	0.0071	0.44392	0.91069	0.06519	0.10305	0.42085	0.7848
95% Conf. Interval	1.0089	0.2344	0.121	0.1948	0.4599	0.9583	0.8483	0.2159	0.5875
	1.0613	2.2015	0.7172	2.0478	2.3897	4.0351	5.9976	1.8971	2.0222

Multivariate	Age	ER.Status	PR.Status	HER2.Final.Status	Tumor Size	Lymph Node	Metastasis	PAM50 subtype	SUV420H2
Hazard Ratio	1.03644	0.71011	0.28572	0.60622	1.02426	2.01432	2.06767	0.60465	1.16544
P-value	0.00713	0.55434	0.00624	0.40166	0.95466	0.05848	0.17137	0.3753	0.64288
95% Conf. Interval	1.0098	0.2283	0.1164	0.1882	0.4483	0.9752	0.7302	0.1988	0.6101
	1.0638	2.2089	0.7012	1.9527	2.3401	4.1607	5.8552	1.8388	2.2261

Multivariate	Age	ER.Status	PR.Status	HER2.Final.Status	Tumor Size	Lymph Node	Metastasis	PAM50 subtype	WHSC1
Hazard Ratio	1.03227	0.68056	0.29982	0.65793	0.98468	1.94941	2.53259	0.50792	1.56208
P-value	0.01531	0.50898	0.00889	0.4838	0.97101	0.07345	0.07394	0.24229	0.18153
95% Conf. Interval	1.0061	0.2172	0.1216	0.2038	0.4284	0.9386	0.914	0.1632	0.812
	1.0591	2.1324	0.7392	2.1238	2.2634	4.0489	7.0177	1.5811	3.0051

Multivariate	Age	ER.Status	PR.Status	HER2.Final.Status	Tumor Size	Lymph Node	Metastasis	PAM50 subtype	WHSC1L1
Hazard Ratio	1.03252	0.646727	0.288934	0.690364	1.002898	1.892351	2.141124	0.582225	1.548182
P-value	0.01426	0.45439	0.00652	0.5375	0.99445	0.08147	0.13013	0.33706	0.19717
95% Conf. Interval	1.0064	0.2065	0.1181	0.2126	0.4438	0.9234	0.7989	0.193	0.7968
	1.0593	2.0259	0.7068	2.2418	2.2662	3.8782	5.7384	1.7567	3.0082
