# Supplementary material for: Genetic alterations of histone lysine methyltransferases and their significance in breast cancer
Source: Oncotarget. 2014 Dec 11;6(4):2466–82. doi: 10.18632/oncotarget.2967 (PMC4385864; doi:10.18632/oncotarget.2967)
Supplement: Supplementary file 10 [file oncotarget-06-2466-s010.pdf]

Table S9. Copy number of four HMTs, SETDB1, ASH1l, SMYD2 and SMYD3 in 88 TCGA basal breast cancer samples

CASE ID	SETDB1	ASH1L	SMYD2	SMYD3
TCGA-A1-A0SK	1	1	-1	-1
TCGA-A2-A04P	1	1	1	1
TCGA-A2-A04Q	1	1	1	1
TCGA-A2-A04T	2	2	1	2
TCGA-A2-A04U	2	-1	1	1
TCGA-A2-A0CM	2	2	1	0
TCGA-A2-A0D0	2	1	1	2
TCGA-A2-A0D2	1	1	1	1
TCGA-A2-A0ST	1	1	1	1
TCGA-A2-A0SX	1	1	1	1
TCGA-A2-A0T0	1	1	1	1
TCGA-A2-A0T2	2	1	0	0
TCGA-A2-A0YE	1	1	1	1
TCGA-A2-A0YJ	1	1	1	1
TCGA-A2-A0YM	1	1	1	1
TCGA-A7-A0CE	2	2	2	2
TCGA-A7-A0DA	2	2	1	2
TCGA-A7-A13D	2	2	1	2
TCGA-A7-A13E	1	1	1	1
TCGA-A8-A07O	2	2	1	2
TCGA-A8-A07R	2	1	1	1
TCGA-A8-A07U	1	1	1	1
TCGA-A8-A08H	0	0	0	0
TCGA-A8-A08R	1	1	1	1
TCGA-AN-A04D	1	1	1	2
TCGA-AN-A0AL	1	1	-1	1
TCGA-AN-A0AR	0	0	1	-1
TCGA-AN-A0AT	0	0	-1	1
TCGA-AN-A0FJ	1	2	0	0
TCGA-AN-A0FL	1	1	1	1
TCGA-AN-A0FX	2	1	1	1
TCGA-AN-A0XU	1	1	1	0
TCGA-AO-A0J4	2	2	2	2
TCGA-AO-A0J6	1	1	0	0
TCGA-AO-A0JL	1	1	1	2
TCGA-AO-A124	1	1	1	2
TCGA-AO-A128	1	1	1	1
TCGA-AO-A129	2	2	1	2
TCGA-AO-A12F	1	1	2	2
TCGA-AQ-A04J	2	2	0	2
TCGA-AR-A0TS	1	1	1	1
TCGA-AR-A0U0	1	1	1	1
TCGA-AR-A1AH	2	2	2	2

TCGA-AR-A1AI	1	1	-1	1
TCGA-AR-A1AJ	1	1	1	1
TCGA-AR-A1AQ	1	1	0	0
TCGA-AR-A1AR	1	1	1	0
TCGA-AR-A1AY	1	1	1	1
TCGA-B6-A0I2	1	1	1	1
TCGA-B6-A0IJ	2	2	2	1
TCGA-B6-A0IQ	2	2	2	2
TCGA-B6-A0RE	2	2	1	1
TCGA-B6-A0RT	2	2	0	2
TCGA-B6-A0RU	1	1	1	2
TCGA-B6-A0WX	2	2	2	2
TCGA-B6-A0X1	1	1	0	1
TCGA-BH-A0AV	2	2	-1	1
TCGA-BH-A0B3	2	2	1	2
TCGA-BH-A0B9	1	1	1	0
TCGA-BH-A0BG	1	1	1	1
TCGA-BH-A0BL	2	2	1	2
TCGA-BH-A0BW	2	2	0	2
TCGA-BH-A0DL	1	1	1	1
TCGA-BH-A0E0	2	2	0	0
TCGA-BH-A0E6	1	1	1	1
TCGA-BH-A0RX	1	1	1	1
TCGA-BH-A0WA	0	1	1	-1
TCGA-BH-A18G	1	1	1	1
TCGA-BH-A18K	1	1	1	2
TCGA-BH-A18Q	1	1	1	2
TCGA-BH-A18V	2	2	1	1
TCGA-BH-A1F0	1	1	1	1
TCGA-C8-A12K	0	1	1	0
TCGA-C8-A12V	1	1	1	1
TCGA-C8-A131	1	1	1	1
TCGA-C8-A134	0	0	0	0
TCGA-D8-A142	1	1	1	1
TCGA-D8-A143	1	1	1	1
TCGA-D8-A147	2	2	0	2
TCGA-E2-A14N	1	1	1	1
TCGA-E2-A14R	2	0	-2	1
TCGA-E2-A14X	1	1	1	1
TCGA-E2-A14Y	1	1	1	1
TCGA-E2-A150	1	1	1	1
TCGA-E2-A158	1	2	0	1
TCGA-E2-A159	1	1	1	1
TCGA-E2-A1AZ	2	1	1	1
TCGA-E2-A1B5	0	0	1	1
